# The International Headache Congress – IHS and EHF joint congress 2021: Virtual. 8-12 September 2021

**DOI:** 10.1186/s10194-021-01293-9

**Published:** 2021-09-07

## AL01 Estimating the Probability of Reported Versus Theoretical Drug-Drug Interactions in Headaches Medicine

### V. Kaytser, I. Hakkinen, P. Zhang

#### Rutgers Robert Wood Johnson Medical School, New Brunswick, NJ, United States

##### **Correspondence:** V. Kaytser; I. Hakkinen; P. Zhang

Background: This project aims to compare the likelihood of a theoretical drug-drug interaction between a number of abortives and preventives using DrugBank"s application programing interface (API) versus the empirically reported interactions using the FDA"s Adverse Event Reporting System (FAERS) API.

Methods: We included, as input, abortive and preventive drugs from the *AHS Position Statement on Integrating New Migraine Treatments into Clinical Practice*, as well as Szperka"s, *Migraine Care in the Era of COVID-19*. All combinations of up to 3 abortives and/or preventives are screened for interactions through DrugBank and FAERS. If at least one interaction, of any type, is listed, then it is included here and compared across the two databases.

Results: We included 38 abortives and 23 preventives. We downloaded DrugBank data on August 26, 2020 and included FAERS data from October 2012 to March 2020. Table 1 contains the number of interactions for a given number of medications. Due to hardware limitations, 3 abortives vs. 3 preventives was not analyzed.

Conclusion: The likelihood of an interaction increases as the number of combinations of abortives and preventives increases. Per DrugBank, the chance of an interaction is >99% once more than 3 drugs are used in combination. Whereas, the reported interaction is actually less, 60%, per FAERS. This data may help providers to use more rational polypharmacy.

**Table 1 (abstract AL01).**
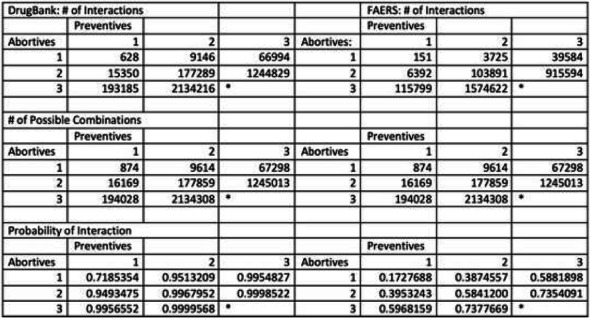
See text for description

## AL02 An AI-enabled ECG Algorithm Predicts Higher Subclinical Atrial Fibrillation Risk in Patients with Migraine with Aura Compared to Migraine without Aura

### N. Chhabra^1^, C. J. Chao^2^, N. Zhang^3^, E. Lim^3^, H. Wang^4^, Z. Attia^5^, P. Friedman^5^, P. Noseworthy^2^, C. C. Chiang^6^

#### ^1^Mayo Clinic, Neurology, Scottsdale, AZ, United States; ^2^Mayo Clinic, Cardiology, Scottsdale, AZ, United States; ^3^Mayo Clinic, Quantitative Health Research, Scottsdale, AZ, United States; ^4^Mayo Clinic, Neurology, Mankato, MN, United States; ^5^Mayo Clinic, Cardiology, Rochester, MN, United States; ^6^Mayo Clinic, Neurology, Rochester, MN, United States

##### **Correspondence:** N. Chhabra

Objective: Migraine with aura (MwA) is associated with a 2-fold risk of ischemic stroke. Higher incidence of atrial fibrillation (AF) has been demonstrated in MwA compared to Migraine without aura (MwoA) in longitudinal cohort studies. The Mayo Clinic Cardiology team developed an artificial intelligence-enabled ECG (AI-ECG) algorithm that predicts probability of AF in ECGs interpreted as normal sinus rhythm (NSR). We aim to assess the probability of AF predicted by the AI-ECG algorithm in patients with MwA and MwoA.

Methods: Adult patients with a MwA or MwoA diagnosis and at least one NSR ECG within the past 5 years at Mayo Clinic were identified. Patients with AF and inconsistent diagnoses of migraine types were excluded. The AI-ECG data with the highest predicted AF probability was used to compare the MwA and MwoA groups.

Results: The analysis included 676 MwA and 1124 MwoA patients. The MwA group was significantly older than MwoA (50.2 vs. 46.6, p <0.001). After adjusting for age and sex, MwA patients had a higher mean probability of AF compared to MwoA (7.6%±0.5% vs. 5.9%± 0.4% p =0.003). The difference of AF probability between MwA and MwoA was significant in men, but not in women.

Conclusions: The probability of AF predicted by an AI-enabled ECG algorithm is significantly higher in patients with MwA compared to MwoA. This supports that AF-mediated cardioembolism plays a significant role in the MwA-stroke association.

**Table 1 (abstract AL02).**
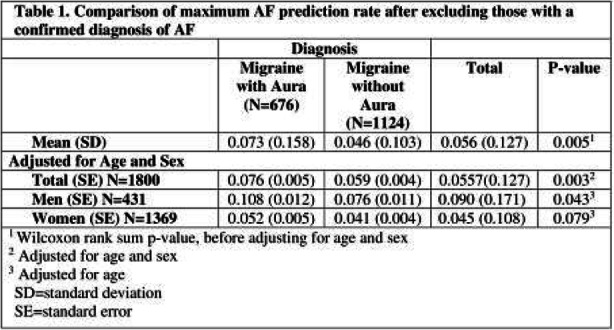
See text for description

## AL03 Gene networks reveal functional distinct mechanisms segregating in families with clustering of migraine

### A. Rasmussen^1^, L. J. A. Kogelman^1^, D. M. Kristensen^1^, M. A. Chalmer^1^, J. Olesen^1^, T. F. Hansen^1,2^

#### ^1^Copehagen University Hospital, Danish Headache Center, Glostrup, Denmark; ^2^Copenhagen University, Novo Nordic Foundation Center for Protein Research, Copenhagen, Denmark

##### **Correspondence:** T. F. Hansen

Background: Migraine has complex polygenic origins with a heritability of estimated 40–70%. Both common and rare genetic variants are believed to underlie the pathophysiology of the prevalent types of migraine, migraine with typical aura and migraine without aura. However, only common variants have been identified so far.

Methods: We utilize a systems genetics approach and integrate RNA sequencing data from brain and vascular tissues knwon to be involved in mgriane, and assessed whole genome sequencing of 117 families with clustering of migraine. We then use a whole genome sequenced cohort of clinical and urelated patients with migraine.

Results: A gene module in the visual cortex, based on single nuclei RNA sequencing data, that had increased rare mutations in the migraine families and replicated this in a second independent cohort of 1930 patients. This module was mainly expressed by interneurons, pyramidal CA1, and pyramidal SS cells, and pathway analysis showed association with hormonal signalling (thyrotropin-releasing hormone and oxytocin receptor), Alzheimer"s disease pathway, serotonin receptor pathway and general heterotrimeric G-protein signalling pathways.

Conlcusion: We demonstrate that rare functional gene variants are strongly implicated in the pathophysiology of migraine. Furthermore, we our results may explain some of the missing heritability and thus mechanisms behind migraine.

## AL04 Genome-wide analysis of 102,084 migraine cases identifies 123 risk loci and subtype-specific risk alleles

### H. Hautakangas^1^, B. S. Winsvold^2,3,4^, S. E. Ruotsalainen^1^, G. Bjornsdottir^5^, A. V. E. Harder^6,7^, L. J. A. Kogelman^8^, L. F. Thomas^3,9,10,11^, R. Noordam^12^, H. Stefánsson^5^, K. Stefansson^5^, A. van den Maagdenberg^6,7^, T. F. Hansen^8,13^, S. Ripatti^1,14,15^, J. A. Zwart^2,3,16^, A. Palotie^1,17,18^, M. Pirinen^1,15,19^

#### ^1^Institute for Molecular Medicine Finland (FIMM), Helsinki Institute of Life Science (HiLIFE), University of Helsinki, Helsinki, Finland; ^2^Department of Research, Innovation and Education, Division of Clinical Neuroscience, Oslo University Hospital, Oslo, Norway; ^3^K. G. Jebsen Center for Genetic Epidemiology, Department of Public Health and Nursing, Faculty of Medicine and Health Sciences, Norwegian University of Science and Technology, Trondheim, Norway; ^4^Department of Neurology, Oslo University Hospital, Oslo, Norway; ^5^deCODE genetics/Amgen Inc., Reykjavik, Iceland; ^6^Leiden University Medical Center, Department of Neurology, Leiden, Netherlands; ^7^Department of Human Genetics, Leiden University Medical Center, Leiden, Netherlands; ^8^Danish Headache Center, Copenhagen, Denmark; ^9^Department of Clinical and Molecular Medicine, Norwegian University of Science and Technology, Trondheim, Norway; ^10^BioCore - Bioinformatics Core Facility, Norwegian University of Science and Technology, Trondheim, Norway; ^11^Clinic of Laboratory Medicine, St.Olavs Hospital, Trondheim University Hospital, Trondheim, Norway; ^12^Department of Internal Medicine, Section of Gerontology and Geriatrics, Leiden University Medical Center, Leiden, Netherlands; ^13^Novo Nordic Foundation Center for Protein Research, Copenhagen University, Copenhagen, Denmark; ^14^Broad Institute of MIT and Harvard, Cambridge, MA, United States; ^15^Department of Public Health, University of Helsinki, Helsinki, Finland; ^16^Institute of Clinical Medicine, Faculty of Medicine, University of Oslo, Oslo, Norway; ^17^Analytic and Translational Genetics Unit, Department of Medicine, Department of Neurology and Department of Psychiatry Massachusetts General Hospital, Boston, MA, United States; ^18^The Stanley Center for Psychiatric Research and Program in Medical and Population Genetics, The Broad Institute of MIT and Harvard, Cambridge, MA, United States; ^19^Department of Mathematics and Statistics, University of Helsinki, Helsinki, Finland

##### **Correspondence:** H. Hautakangas

Objective: We set out to conduct the largest genome-wide analysis of migraine to date and further evaluated shared and distinct genetic components in the two main migraine subtypes: migraine with aura (MA) and migraine without aura (MO).

Methods: Our analysis included 102,084 migraine cases and 771,257 controls. We used LDSC to evaluate whether the polygenic migraine signal was enriched near genes that were active in certain tissue or cell types.

Results: We identified 123 risk loci of which 86 are novel. A stratification of the risk loci by subtypes indicated 3 risk variants that appear specific for MA (in *HMOX2*, *CACNA1A* and *MPPED2*), 2 that appear specific for MO (near *SPINK2* and near *FECH*), and 9 that increase susceptibility for migraine regardless of subtype. The new risk loci include genes encoding recent migraine-specific drug targets, namely calcitonin gene-related peptide (*CALCA/CALCB*) and serotonin 1F receptor (*HTR1F*). We report enrichment of migraine signal in 5 central nervous system and 3 cardiovascular cell types, and in single cell types of digestive system, musculoskeletal/connective tissue and ovary at FDR 5%.

Conclusion: New risk loci include migraine-specific drug targets. We provided a concrete view to the homogeneity and heterogeneity in the genetic background of migraine subtypes. Genomic annotations among migraine-associated variants supported that neurovascular mechanisms underlie migraine pathophysiology.

## AL05 Dry eye disease in migraine: A case control study

### B. Chowdhury^1^, P. Dubey^1^, D. Chowdhury^2^

#### ^1^North DMC Medical College & Hindu Rao Hospital, Ophthalmology, Delhi, India; ^2^G B Pant Institute of Post graduate Medical Education and Research, NEUROLOGY, Delhi, India

##### **Correspondence:** B. Chowdhury

Objective: To study the prevalence of dry eye disease (DED) in migraine patients and compare it with non-migraine controls.

Method: This was a cross-sectional, observational hospital-based study. Sixty preventive drug-naive migraineurs diagnosed by ICHD-3 and 60 controls (patients presenting for refractive error without any migraine), aged 18-65 years were studied. Patients with comorbidities that can cause DED were excluded. Tear film break-up time (TBUT), Fluorescein staining, Schirmer"s-I test, and ocular surface disease index (OSDI) scores were generated. Severe DED was diagnosed using the ODISSEY algorithm. Only severely affected eye was used for comparison.

Result: The migraineurs and the controls were age and sex-matched (33.5 ± 7.1; M: F=46:14 vs 33.8 ± 7.4; M: F=46:14). The mean TBUT (11.65±6.33 vs 14.30±4.67 seconds) and fluorescein scores (1.125±0.81 vs 0.692±0.63) were significantly worse in cases compared to controls (p=0.010). The prevalence of DED and severe DED was found to be higher in migraine patients (46.7% and 16.7%) as compared to controls (18.3% and 1.7%) (Table1). Among the migraineurs, only mean pain severity was significantly associated with the presence of DED (Table 2). Migraineurs with severe DED had both higher frequency and severity of headache attacks.

Conclusion: DED was more common in migraine patients compared to non-migraineurs. Severe DED in migraineurs was associated with increased frequency and severity of headache.

**Table 1 (abstract AL05).**
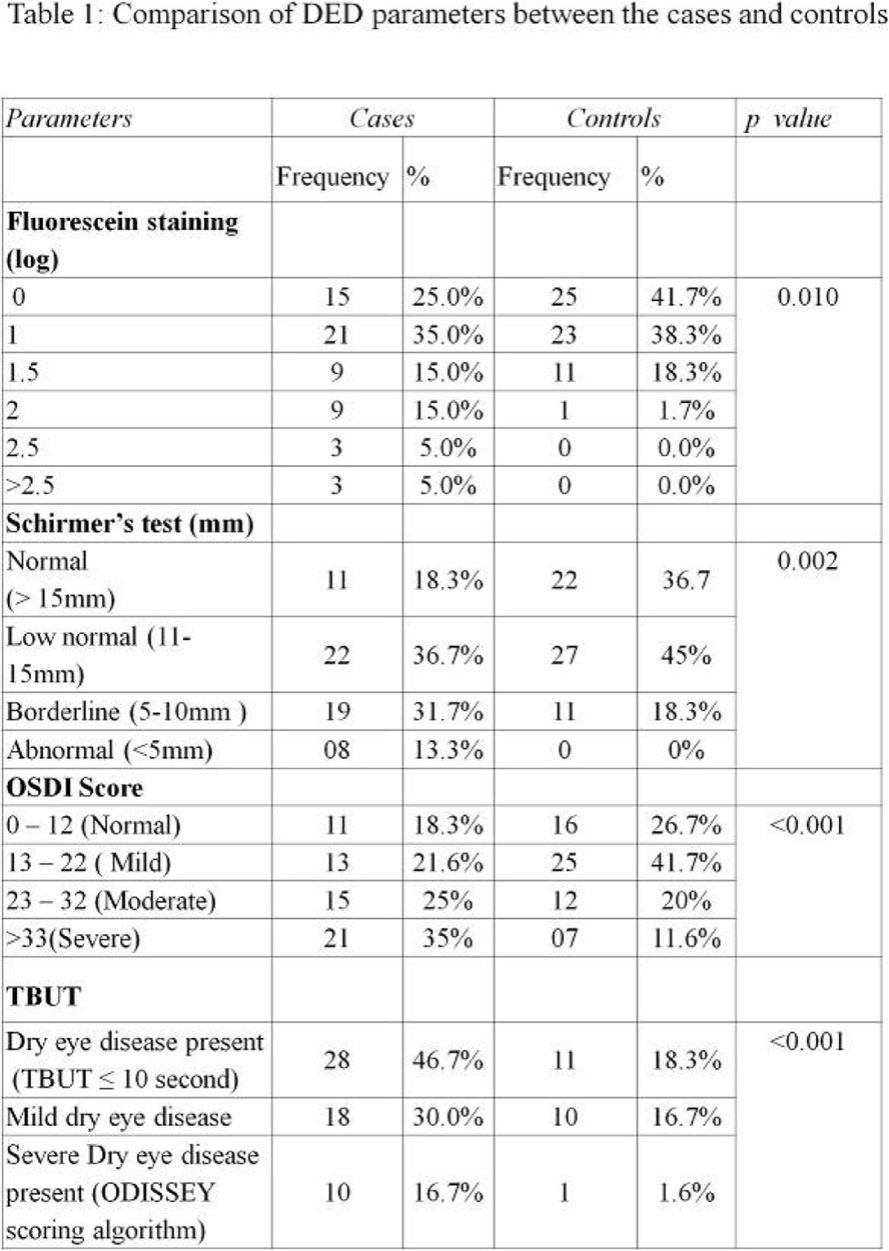
See text for description

**Table 2 (abstract AL05).**
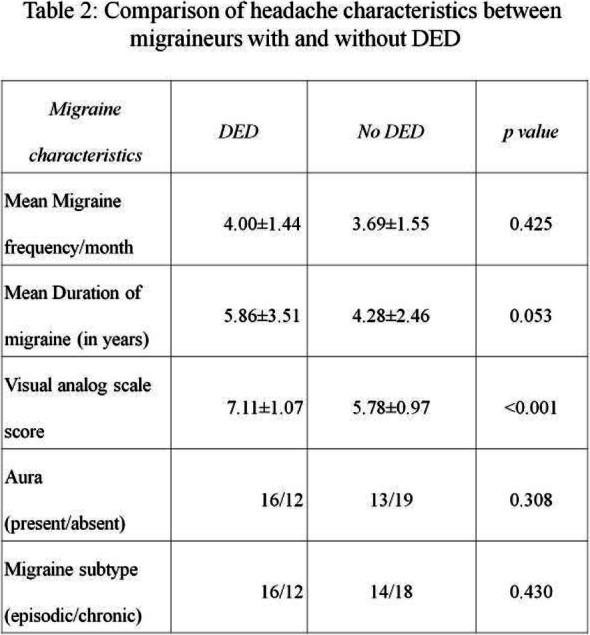
See text for description

## AL06 Cortical morphological changes in cluster headache between bouts: voxel- and surface-based analyses

### L. Lai^1,2^, Y. T. Hsiao^3^, W. K. Chan^3^, S. J. Wang^3,1,2^

#### ^1^National Yang Ming Chiao Tung University, Neurology, Taipei City, Taiwan; ^2^National Yang Ming Chiao Tung University, Brain Research Center, Taipei City, Taiwan; ^3^Taipei Veterans General Hospital, Department of Neurology, Taipei City, Taiwan

##### **Correspondence:** L. Lai

Objective: Previous structural imaging studies in cluster headache (CH) used either volume- (VBM) or surface-based morphometry (SBM) to evaluate related morphological changes. A study combining both methods may provide further insights.

Methods: 94 CH (47 during in-bout [CH-in], and 47 during out-of-bout period [CH-out]) and 47 healthy controls (CTL) were analyzed. VBM and SBM were applied to investigate between-group differences. Averaged volumes or cortical thicknesses from regions showing group differences were correlated with clinical parameters.

Results: Compared with CTL, VBM showed reduction of gray matter volume (GMV) in multiple areas, confined to the pain matrix, in patients with CH (CH-all), CH-in and CH-out. Increased GMV at bil putamen was observed in CH-all and CH-in, but not CH-out, suggesting this effect may be derived from CH-in. SBM revealed a reduction of cortical thickness in ant/mid cingulate, and bil insula cortices in CH-all and CH-in, but not CH-out, suggesting CH-in contributed to this effect. Additionally, the cortical thickness at right insula correlated negatively with disease duration in CH-in group.

Conclusion: CH-in and CH-out showed distinct morphological changes. Both groups showed reduced GMV in regions within the pain-processing network, while CH-in additionally presented with reduced cortical thickness over bil insular and cingulate cortices, and increased volume in bil putamen. These changes may be related to trait- and state-dependent effects.

**Fig. 1 (abstract AL06).**
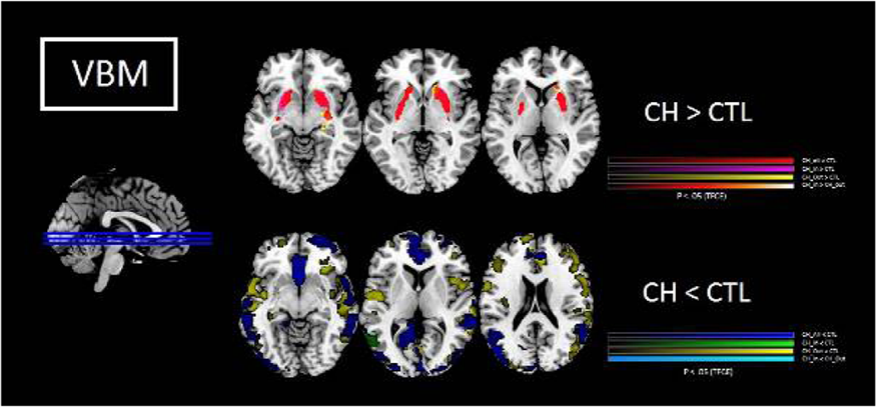
See text for description

**Fig. 2 (abstract AL06).**
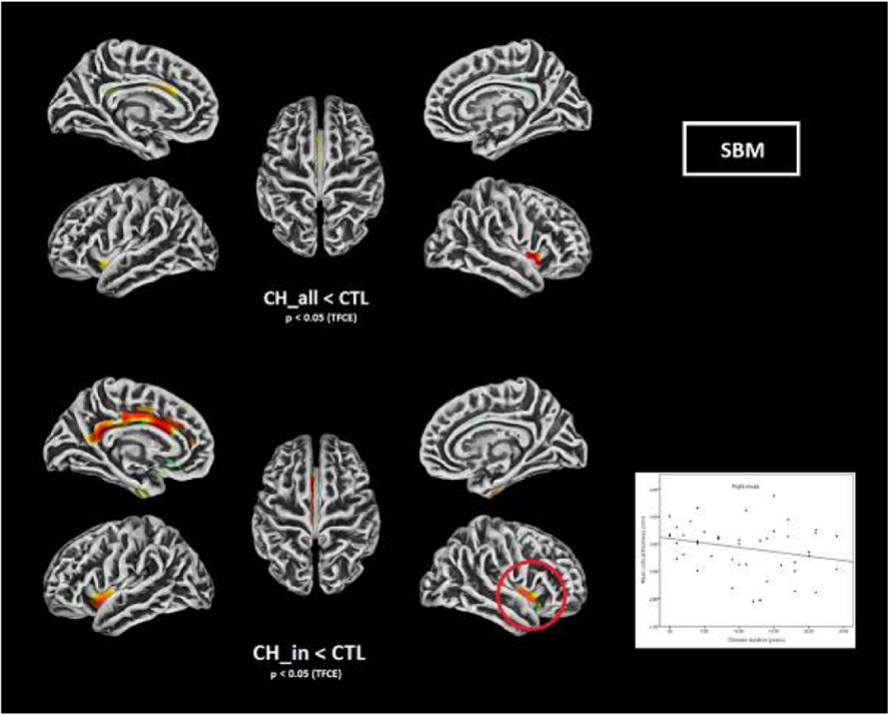
See text for description

## AL07 I stay at home with headache. A survey to investigate the effects of lockdown on headache in Italian children

### L. Papetti^1^, L. Grazzi^2^, V. Guidetti^3,4^, V. Sciruicchio^5,6,7^, C. Termine^8^, I. Toldo^9^, E. Tozzi^10^, P. Verdecchia^4^, S. Tarantino^1^, R. Moavero^1^, F. Ursitti^1^, M. A. N. Ferilli^1^, G. Sforza^1^, M. Valeriani^1^

#### ^1^Bambino Gesù Children's Hospital, Neuroscience, Rome, Italy; ^2^IRCCS Foundation “Carlo Besta” Neurological Institute, Headache Center, Neuroalgology Department, Rome, Italy; ^3^Sapienza University of Rome, Neuroscience, Rome, Italy; ^4^Department of Human Neuroscience, Section of Child and Adolescent Neuropsychiatry, “Sapienza” University, Rome, Italy; ^5^Ospedale Sant'Andrea, Università Sapienza di Roma, Rome, Italy; ^6^Ismep - ARNAS Civico, Child Neuropsychiatry Unit -, Palermo, Italy; ^7^PO, San Paolo ASL (Azienda Sanitaria Locale), Children Epilepsy and EEG center, Rome, Italy; ^8^Department of Medicine and Surgery, University of Insubria and ASST dei Sette Laghi, Varese, Italy; ^9^Dipartimento di Salute della Donna e del Bambino, Università degli Studi, Azienda Ospedaliera di Padova,, Centro Cefalee per l'età Evolutiva, Padova, Italy; ^10^Università degli Studi dell'Aquila, Dipartimento di Medicina Clinica, Sanità Pubblica, Scienze della Vita e dell'ambiente, L'Aquila, Italy

##### **Correspondence:** L. Papetti

Objective: The present Italian multicenter study aimed at investigating whether the course of primary headache disorders in children and adolescents was changed during the lockdown necessary to contain the COVID-19 emergency in Italy.

Methods: During the lockdown, we submitted an online questionnaire to patients already diagnosed with primary headache disorders. Questions explored the course of headache, daily habits, psychological factors related to COVID-19, general mood and school stress.

Results: We collected the answers of 707 patients. In the multivariate analysis, we found that reduction of school effort and anxiety was the main factor explaining the improvement in the subjective trend of headache and the intensity and frequency of the attacks (p < 0.001). The greater the severity of headache, the larger was the clinical improvement (p < 0.001). Disease duration was negatively associated with the improvement (p < 0.001). It is noteworthy that clinical improvement was independent of prophylaxis (p > 0.05), presence of chronic headache disorders (p > 0.05) and geographical area (p > 0.05).

Conclusions: Our study showed that lifestyle modification represents the main factor impacting the course of primary headache disorders in children and adolescents. In particular, reduction in school-related stress during the lockdown was the main factor explaining the general headache improvement in our population.

**Fig. 1 (abstract AL07).**
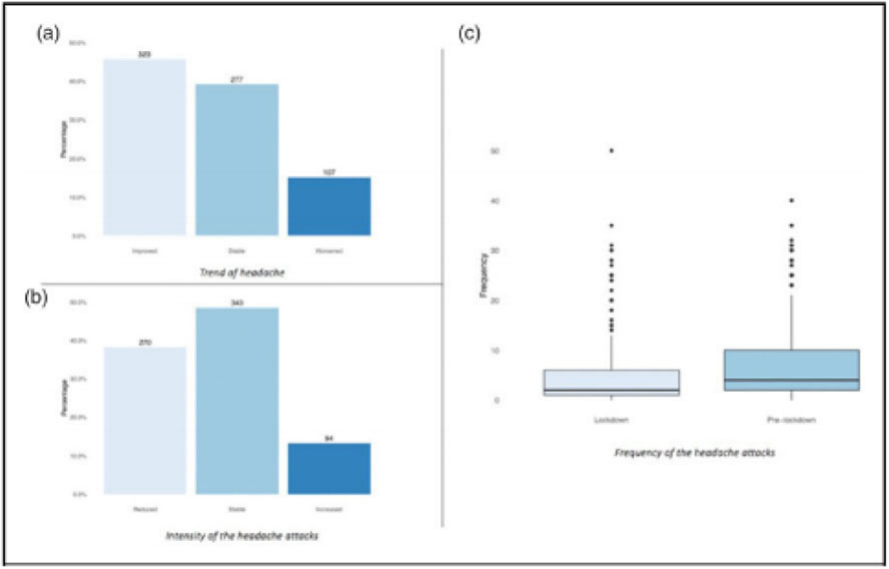
See text for description

**Fig. 2 (abstract AL07).**
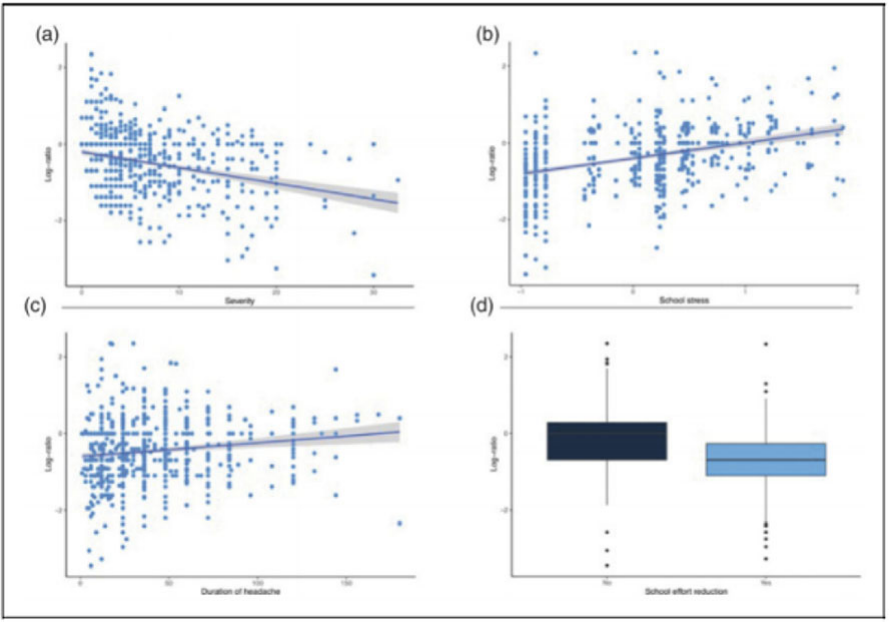
See text for description

## AL08 Patient Experience of Telemedicine for Headache Care during the COVID-19 Pandemic: an American Migraine Foundation Survey Study

### C. C. Chiang^1^, R. Halker Singh^2^, N. Lalvani^3^, K. Stein^4^, D. Lorenz^5^, C. Lay^6^, D. W. Dodick^2^, L. Newman^7^

#### ^1^Mayo Clinic, Neurology, Rochester, MN, United States; ^2^Mayo Clinic, Neurology, Scottsdale, AZ, United States; ^3^American Migraine Foundation, New York, NY, United States; ^4^Northwell Health, Neurology, Hempstead, NY, United States; ^5^Lorenz & Kopf LLC, Scottsdale, AZ, United States; ^6^University of Toronto, Neurology, Toronto, Canada; ^7^New York University Langone Medical Center, Neurology, New York, NY, United States

##### **Correspondence:** C. C. Chiang

Objective: We sought to investigate the patient experience of telemedicine for headache care during the COVID-19 pandemic.

Methods: The American Migraine Foundation designed a standardized electronic questionnaire to assess the patient experience of telemedicine for headache care between March and September 2020. The questionnaire was distributed electronically to more than 100,000 members through social media platforms and email database.

Results: A total of 1172 patients responded to our electronic questionnaire, with 1098 complete responses. 93.8% patients had a previous headache diagnosis. 648 patients reported they had used telemedicine for headache care during. 85.5% patients used it for follow up and 14.5% for new patient visits. During the telemedicine encounters, 43.7% were evaluated by headache specialists, 34.4% by general neurologists, 30.7% by primary care providers, 11.3% by headache nurse practitioners. 7.4% patients received a new headache diagnosis, and a new treatment was prescribed for 52.3% patients. 82.8% of patients rated the telemedicine headache care experience as "very good" or "good". 89.8% patients indicated that they would continue to use telemedicine for their headache care.

Conclusions: Our study evaluating the patient perspective demonstrated that telemedicine facilitated headache care for many patients during the COVID-19 pandemic, resulting in high patient satisfaction rates, and a desire to continue to utilize telemedicine for future headache care.

**Fig. 1 (abstract AL08).**
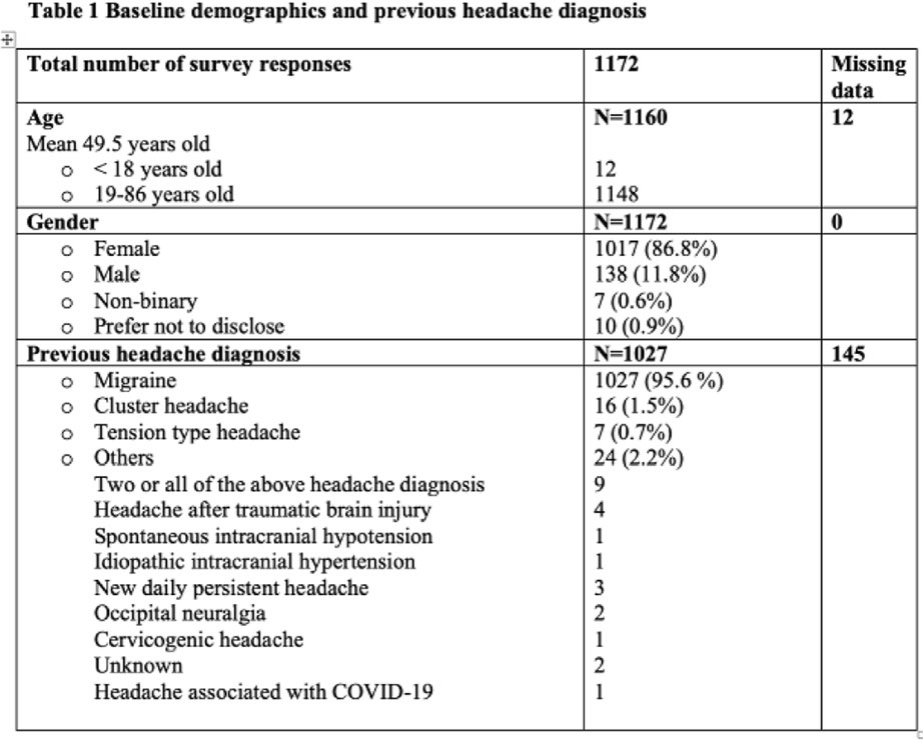
See text for description

**Fig. 2 (abstract AL08).**
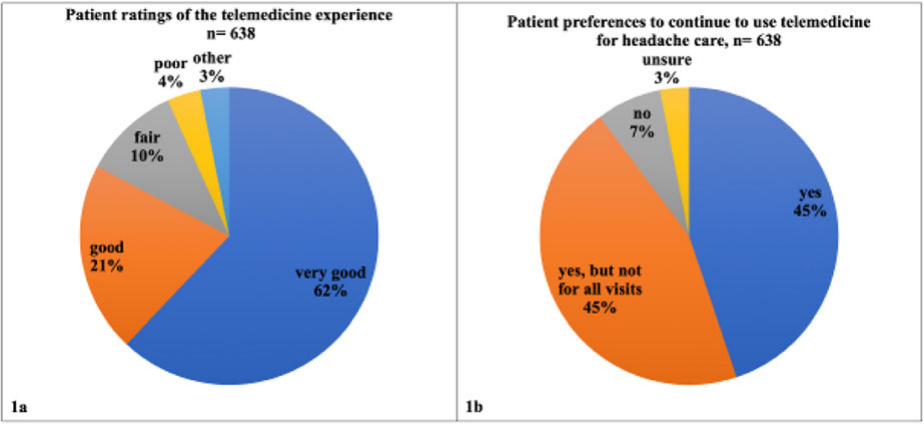
See text for description

## AL09 Cerebral venous thrombosis (CVT) associated with COVID-19 infection; A multi-center study

### S. H. Shaikh

#### Aga Khan University, Neurology, Karachi, Pakistan

Background: Coronavirus disease 2019 (COVID-19) has an increased propensity for systemic hypercoagulability and thromboembolism. An association of cerebrovascular diseases especially CVT has been reported among these patients. The objective of the present study is to identify risk factors, presentation, and outcome of CVT in COVID-19 patients.

Methods: It is a multicenter and multinational prospective observational study. Ten centers in four countries participated in this study. Study included patients (aged > 18 years) with symptomatic CVT and recent COVID-19 infection.

Results: 20 patients (70% men) were included. Mean age was 42.4 years with a male to female ratio of 2.3:1. Headache (85%) and seizures (65%) were the common neurological features with a mean admission Glasgow Coma Score (GCS) of 13. Respiratory symptoms were absent in 45% of the patients. The most common MRI finding was infarction (65%). Superior sagittal sinus (65%) was the most common site for thrombosis. Acute inflammatory markers were raised. Homocysteine was elevated in half of the cases. Mortality rate was 20%. A good functional outcome was seen in the surviving patients with a mRS at discharge was 1.3.

Conclusion: COVID-19 patients are at high risk for CVT secondary to the high incidence of systemic thromboembolism. CVT should be suspected in COVID-19 patients presenting with headache or seizures. Mortality is high but the functional neurological outcome is good among survivors.

## AL010 The characteristics of COVID-19 vaccine-related headache

### E. Ekizoglu^1^, H. Gezegen^1^, P. Yalınay Dikmen^2^, E. Kocasoy Orhan^1^, M. Ertaş^1^, B. Baykan^1^

#### ^1^Istanbul University, Istanbul Faculty of Medicine, Department of Neurology, Istanbul, Turkey; ^2^Acibadem Mehmet Ali Aydinlar University School of Medicine, Department of Neurology, Istanbul, Turkey

##### **Correspondence:** E. Ekizoglu

Objective: Headache is the most common neurological symptom during COVID-19 and a frequent adverse event after viral vaccines. We aimed to investigate the frequency and clinical associations of COVID-19 vaccine-related headache.

Methods: We developed a detailed web-based questionnaire screening headache following vaccination in healthcare professionals who received at least one dose of COVID-19 vaccine. We investigated the associations of this headache with primary headache disorders, main comorbid conditions, headache history during COVID-19 or following influenza vaccine.

Results: A total of 1247 participants (mean age, 47.6±12.3 years;860 females) contributed to the survey;131 (10.5%) had been infected with COVID-19, being asymptomatic or mildly symptomatic in 111 (84.7%). Nearly one-third of all participants (386;31%) had headache after vaccination; 99(25.6%) experienced headache lasting more than two days. The diagnosis of primary headache disorders and migraine were significantly more frequent in participants having COVID-19 vaccine-related headache (p<0.000; p<0.000). The rates of headache during COVID-19 or following influenza vaccine were also significantly higher (p=0.003 and p<0.000). Thyroid diseases showed also a significant association(p=0.001).

Conclusion: Headache is a frequent adverse event following the COVID-19 vaccine and mostly experienced by people with primary headache disorders, having headache during COVID-19 or headache related to other viral vaccines.

## AL011 Gray matter cortical changes in patients with persistent headache after COVID-19 infection: an exploratory study

### Á. Planchuelo-Gómez^1^, D. García-Azorín^2,3^, Á. L. Guerrero Peral^2,3,4^, S. Aja-Fernández^1^, M. Rodríguez^5^, R. Moro^5^, R. de Luis-García^1^

#### ^1^Universidad de Valladolid, Imaging Processing Laboratory, Valladolid, Spain; ^2^Hospital Clínico Universitario de Valladolid, Headache Unit, Valladolid, Spain; ^3^Institute for Biomedical Research (IBSAL), Salamanca, Spain; ^4^Universidad de Valladolid, Department of Medicine, Valladolid, Spain; ^5^Hospital Clínico Universitario de Valladolid, Department of Radiology, Valladolid, Spain

##### **Correspondence:** Á. Planchuelo-Gómez

Objective: To evaluate gray matter alterations in patients with persistent headache after COVID-19 resolution.

Methods: Exploratory case-control study. High-resolution 3D brain T1-weighted Magnetic Resonance Imaging data were acquired in patients with persistent headache after COVID-19 infection and healthy controls (HC). FreeSurfer (version 6.0) was employed to segment the T1-weighted images and extract the mean values of the cortical curvature (CC) and thickness (CT), surface area (SA) and gray matter volume (GMV) of 68 cortical regions. GMV comparisons were adjusted for intracranial volume. Significant results were considered with *p* < 0.05 (False Discovery Rate corrected).

Results: Ten patients with persistent headache after COVID-19 (mean age: 53.8 ± 7.8 years; nine women) and 10 HC balanced for age and sex (mean age: 53.1 ± 7.0 years; nine women) were included in the study. Significant higher mean SA and GMV values were found in patients with persistent headache compared to HC in the bilateral medial orbitofrontal cortex, left rostral middle frontal gyrus, and right pars opercularis and superior frontal gyrus. In the patients, significant higher GMV in the right caudal anterior cingulate gyrus and SA values in five temporal, frontal and parietal regions were observed. No CC or CT changes were found.

Conclusions: Persistent headache after COVID-19 infection is related to gray matter cortical changes defined by higher GMV and SA values mainly localized in frontal regions.

## AL012 Comparison of quantitative headache parameters of headache after vaccination against COVID-19 (Coronavirus SARS-CoV-2) with AZD1222, BNT162b2, mRNA-1273 and BBIBP-CorV vaccines

### C. Göbel^1,2^, A. Heinze^2^, S. Karstedt^1,2^, M. Morscheck^2^, L. Tashiro^2^, A. Cirkel^1,2^, Q. Hamid^3^, R. Halwani^3^, M. H. Temsah^4^, M. Ziemann^5^, S. Görg^5^, T. Münte^1^, H. Göbel^2^

#### ^1^University Hospital Schleswig-Holstein, Department of Neurology, Lübeck, Germany; ^2^Kiel Headache and Pain Centre, Kiel, Germany; ^3^University of Sharjah, College of Medicine, Sharjah, United Arab Emirates; ^4^King Saud University, College of Medicine, Riyadh, Saudi Arabia; ^5^University Hospital Schleswig-Holstein, Institute of Transfusion Medicine, Lübeck, Germany

##### **Correspondence:** C. Göbel

Background: It is not yet known whether the phenotypes of headache after of vaccination against COVID-19 differ with the various available vaccines. This study aims to compare quantitative headache parameters of headache after COVID-19 vaccination with AZD1222 (AstraZeneca), BNT162b2 (BioNTech/Pfizer), mRNA-1273 (Moderna) and BBIBP-CorV (Sinopharm).

Methods: The study is a continuous prospective multicenter observational cohort study taking place during the Covid-19 vaccination campaign. With a publicly available online questionnaire, specific aspects of the headache phenotype, the vaccine used and related variables were collected globally. This is an interim analysis after vaccination with AZD1222 (n=2464), BNT162b2 (n=3285), mRNA-1273 (n=583) and BBIBP-CorV (n=252).

Findings: Headache intensity on the VRS (0-5) was as follows: AZD1222 (3.58±0.90), BNT162b2 (3.37±0.86), mRNA-1273 (3.46±0.84) and BBIBP-CorV (3.06±0.88). The headache intensity after vaccination with AZD1222 was found to be significantly higher than that of the other vaccines. The latency of headache onset after vaccination with AZD1222 was found to be significantly less than that of the other vaccines. The headache duration after vaccination with AZD1222 was found to be significantly longer compared to BBIBP-CorV.

Interpretation: Quantitative parameters of headache after Covid-19 vaccination are strongest after vaccination with AZD1222 compared to other vaccines, and least pronounced with BBIBP-CorV.

## AL013 Heat Pain Threshold and Mechanical Punctate Pain Threshold Predict Treatment Outcomes of Patients with Chronic Migraine

### L. L. Pan^1^, K. L. Lai^1,2,3^, Y. F. Wang^1,2,3^, S. P. Chen^1,2,3^, W. T. Chen^1,2,3^, S. J. Wang^1,2,3^

#### ^1^National Yang Ming Chiao Tung University, Brain Research Center, Taipei City, Taiwan; ^2^Taipei Veterans General Hospital, Department of Neurology, Neurological Institute, Taipei City, Taiwan; ^3^National Yang Ming Chiao Tung University, School of Medicine, Taipei City, Taiwan

##### **Correspondence:** L. L. Pan

Objectives: We aimed to investigate whether the quantitative sensory testing (QST) was associated with treatment outcomes in patients with chronic migraine (CM.)

Methods: Treatment-naïve CM patients were prospectively recruited from the headache clinic. The subjects underwent the QST assessments, focusing on thermal and mechanical pain thresholds over the supraorbital area (V1) and medial forearm (T1) at baseline. They were asked to keep headache diaries for four weeks as baseline before routine preventive treatment and at 3-month follow-up. The responders were defined as at least a 50% reduction in average headache days of the 3 months after intervention from baseline headache days.

Results: Eighty-four CM patients (Table 1) finished the study. Significant differences were found between responders and non-responders in several baseline QST parameters, including heat pain threshold (HPT) and mechanical punctate pain threshold (MPT). Multiple logistic regression showed that V1 HPT (OR: 1.28, *p* = 0.002), V1 MPT (OR: 1.02, *p* = 0.014), and T1 warm detection threshold (OR: 0.33, *p* = 0.013) were associated with the treatment outcomes and overall prediction percentage was 83.3% with Nagelkerke R^2^ of 40.4%. (Table 2)

Conclusions: CM patients who were less sensitive to heat and punctate stimuli were more likely to improve in headache days after 3-month treatment.

**Table 1 (abstract AL013).**
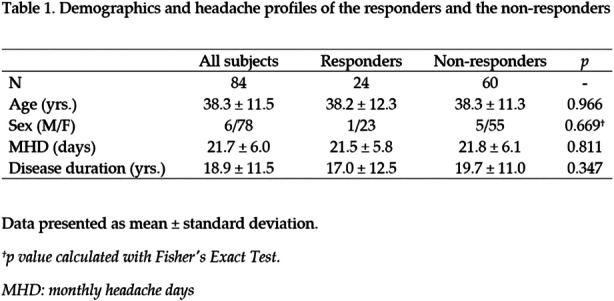
See text for description

**Table 2 (abstract AL013).**
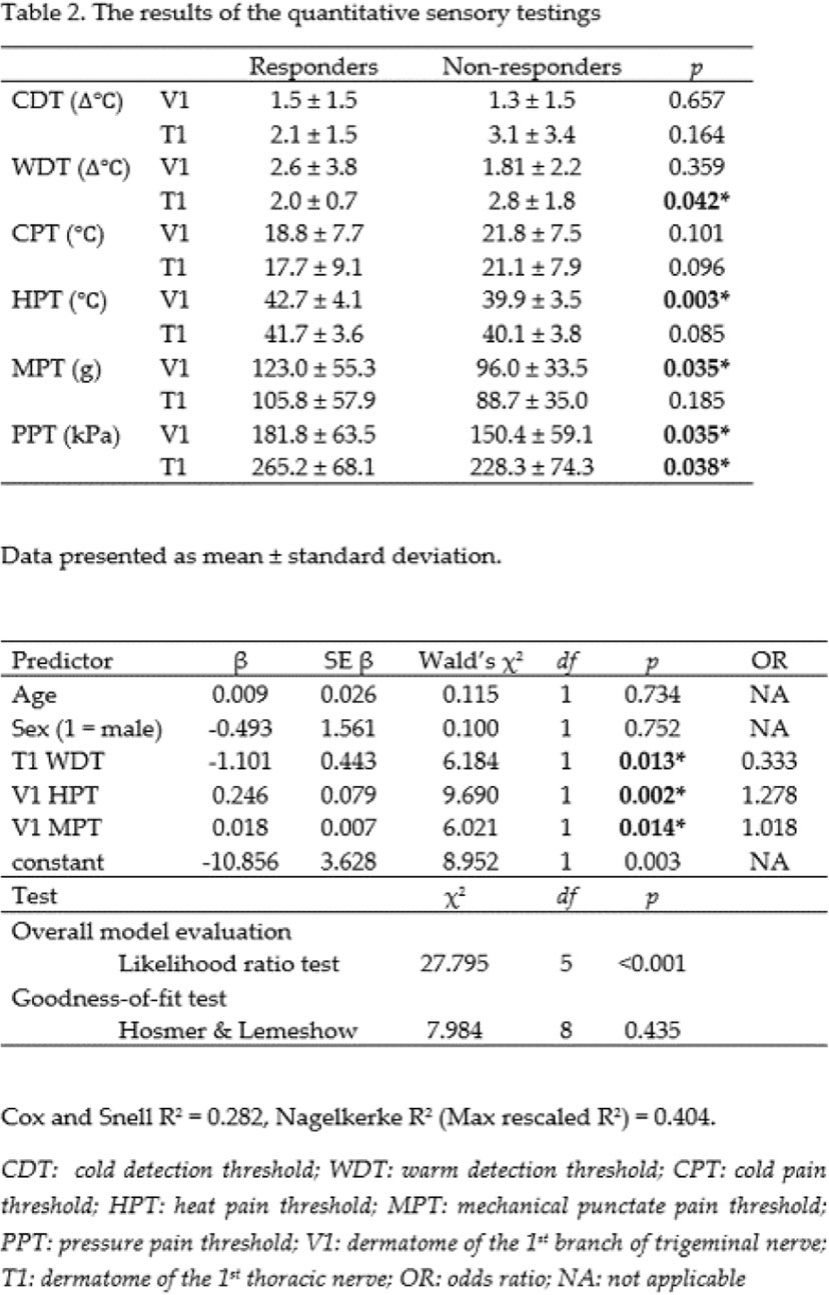
See text for description

## AL014 Migraine-Related Absenteeism is Associated with Total Healthcare and Pharmaceutical Costs – A US-Based Real World Longitudinal Analysis

### L. Harris^1^, G. L'Italien^1^, A. Kumar^2^, P. Seelam^2^, C. LaVallee^3^, R. Croop^1^, V. Coric^1^

#### ^1^Biohaven Pharmaceuticals, New Haven, CT, United States; ^2^Decision Resources Group, Bangalore, India; ^3^Decision Resources Group, Boston, MA, United States

##### **Correspondence:** L. Harris

Objective: Use real-world data to assess absenteeism and healthcare utilization and cost among adults with migraine.

Methods: Claims and EMR data (January 2016-June 2019) for US adults with a diagnosis of migraine and ≥1 Migraine Disability Assessment (MIDAS) score were analyzed. Subjects were classified by treatment setting (primary care or specialist) and categorized by MIDAS score range (0, 1-5, 6-10, 11-30, and >30) into 5 levels of absenteeism from work or school. The association between absenteeism and total healthcare costs (office visits and treatments) was assessed with a series of GLM models adjusted for sex, Charlson comorbidity index, payer channel, and patient comorbidity and migraine flags.

Results: The study population (N=7662) had a mean age of 50 years; 79% were female, and 63% were commercially insured. Migraine-related absenteeism was observed in 69% of the cohort; 41% had at least a moderate level of absenteeism (Figure 1). Absenteeism was positively correlated with medical claims and pharmacy costs regardless of treatment setting (Table 1). Frequent absenteeism and specialist treatment was associated with the highest costs, mainly due to higher costs for medications.

Conclusion: Migraine-related absenteeism was common and directly associated with healthcare costs. The relationship was strongest in subjects with MIDAS scores exceeding 30 who had received specialty care. Improvements in migraine treatment may reduce rates of absenteeism and healthcare costs.

**Fig. 1 (abstract AL014).**
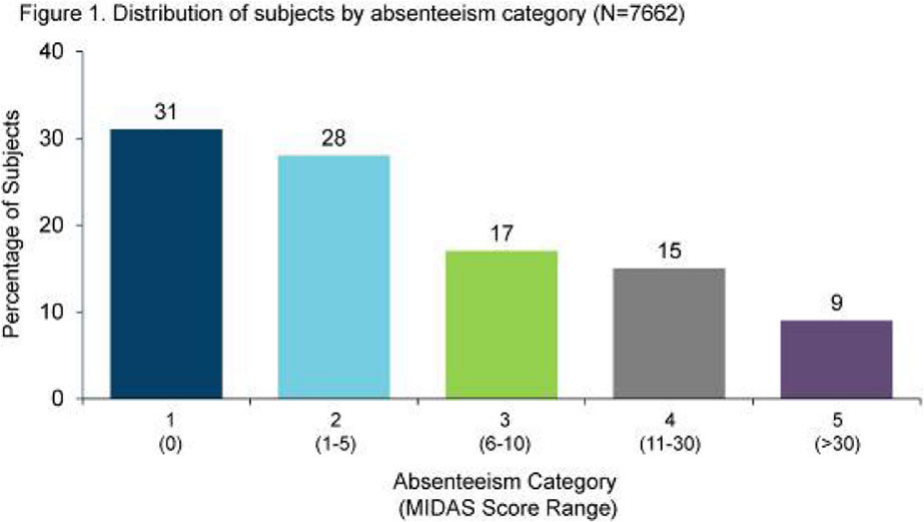
See text for description

**Table 1 (abstract AL014).**
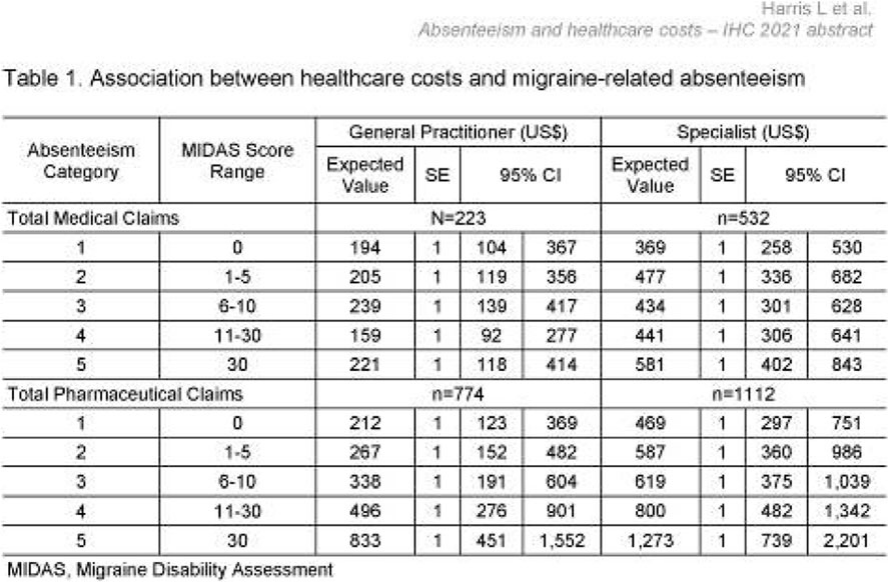
See text for description

## AL015 Association between suicidal risks and medication-overuse headache in chronic migraine: a cross-sectional study

### C. C. Yu^1^, Y. F. Wang^1,2,3^, A. S. Kuan^2,3,4^, S. P. Chen^1,5,3,4^, S. J. Wang^1,3,4^

#### ^1^Taipei Veterans General Hospital, Department of Neurology, Taipei City, Taiwan; ^2^Taipei Veterans General Hospital, Department of Neurosurgery, Taipei City, Taiwan; ^3^National Yang Ming Chiao Tung University, Institute of Public Health, Taipei City, Taiwan; ^4^National Yang Ming Chiao Tung University, School of Medicine, Taipei City, Taiwan; ^5^Taipei Veterans General Hospital, Department of Medical Research, Taipei City, Taiwan

##### **Correspondence:** Y. F. Wang

Objectives: To determine whether medication-overuse headache (MOH), like other substance use disorders, is associated with an increased risk for suicide.

Methods: This prospective cross-sectional study enrolled newly diagnosed chronic migraine (CM) patients with or without coexisting MOH. Headache diagnoses were made through face-to-face interviews by headache specialists, and a specifically designed questionnaire was used to collect clinical characteristics. Suicidal ideation and prior suicide attempt were specifically questioned.

Results: In total, 603 CM patients (485F/118M, mean age 42.03±12.18 years) were recruited, including 320 with MOH (257F/63M, mean age 42.8±11.7 years) (53.1%), and 214 (35.5%) and 81 (13.4%) had suicidal ideation and prior suicide attempt, respectively. Among CM patients, the presence of MOH increased the risks of suicidal ideation (odds ratio [OR]=1.75 [95% CI=1.20-2.56], p=0.004) and prior suicide attempt (OR=1.88 [1.09-3.24], p=0.024), after controlling for demographics, headache profile, disabilities, symptoms of anxiety and depression, and sleep quality.

Conclusions: In CM patients, MOH is associated with an increased risk for suicidal ideation and prior suicide attempt, which deserves attention for clinicians taking care of headache patients. However, further studies are needed to determine the causal relationship, as well as the underlying pathophysiology.

## AL016 Migraine is not Associated with Incident Stroke: The Rotterdam Study

### C. Acarsoy^1^, M. K. Ikram^1,2^, D. Bos^1,3^

#### ^1^Erasmus University Medical Center, Epidemiology, Rotterdam, Netherlands; ^2^Erasmus University Medical Center, Neurology, Rotterdam, Netherlands; ^3^Erasmus University Medical Center, Radiology, Rotterdam, Netherlands

##### **Correspondence:** C. Acarsoy

Objective: A (causal) link between migraine and stroke has been implicated, however so far there is no conclusive answer. In this study, we determined the association between migraine and the risk of stroke.

Methods: This study is based on the on-going prospective Rotterdam Study. Between 2006 and 2011, we included 6925 (mean age 65.7 years, 57.8 % women) participants who did not suffer from previous stroke. At baseline, migraine was assessed with a structured interview based on ICHD-2 criteria. After a median follow-up of 6.4 years, 195 participants developed a stroke. The association between migraine and risk of stroke were analyzed using Cox proportional-hazards regression models. The models were adjusted for age and additionally for cardiovascular, metabolic, lifestyle and psychological risk factors and stratified by sex.

Results: At baseline 1030 (14.9%) participants were diagnosed with migraine. We found no association between migraine and stroke (hazard ratio [HR] 1.40, 95% confidence interval [CI] 0.94-2.09). The results for the different subtypes of migraine were similar (active vs non-active, HR 1.59, CI 0.82-3.05; aura present vs absent, HR 1.45, CI 0.64-3.30). With respect to stroke subtypes, we found no association with ischemic stroke (HR 1.45, CI 0.94-2.24).

Conclusion: Our findings suggest that migraine is not associated with stroke in neither men nor women.

## AL017 Physical inactivity and headache disorders in the ELSA-Brasil cohort: A cross-sectional analysis

### A. Oliveira^1,2,3^, J. Mercante^1,2,3^, M. Peres^2,3^, M. D. C. Molina^4,5^, P. Lotufo^1^, I. Benseñor^1^, A. Goulart^1^

#### ^1^Universidade de São Paulo, Center for Clinical and Epidemiological Research, São Paulo, Brazil; ^2^Hospital Israelita Albert Einstein, Instituto do Cérebro, São Paulo, Brazil; ^3^Universidade de São Paulo, Instituto de Psiquiatria, São Paulo, Brazil; ^4^Universidade Federal de Ouro Preto, Nutrição e Saúde, Ouro Preto, Brazil; ^5^Universidade Federal do Espírito Santo, Vitória, Brazil

##### **Correspondence:** A. Oliveira

Objective: To test the associations between headache disorders and physical inactivity in the ELSA-Brasil cohort.

Methods: In a cross-sectional analysis, logistic regression models computed the odds ratio (OR) for headache disorders according to physical activity levels in the leisure-time (LTPA) and commuting (CPA).

Results: Of 15,105 participants, 14,847 (54.4 % women) provided data on physical activity levels and headache. In the adjusted models, LTPA-physical inactivity associated with definite migraine [OR: 1.32 (1.10-1.57)] and probable migraine [OR: 1.33 (1.17-1.50)] in the whole cohort. In women, it associated with definite migraine [OR: 1.29 (1.04-1.59)] and probable migraine [OR: 1.29 (1.04-1.59)]. In men, LTPA-physical inactivity only associated with probable migraine [OR: 1.40 (1.15-1.69)]. CPA-physical inactivity associated with probable tension-type headache in men [OR: 1.33 (1.01-1.75)], while it inversely associated with definite migraine [OR: 0.79 (0.64-0.98)] and probable migraine [OR: 0.80 (0.67-0.96)] in women. LTPA-vigorous physical inactivity associated with definite migraine [OR: 1.36 (1.13-1.65)] and probable migraine [OR: 1.37 (1.20-1.57)]. There was a strong linear trend for the association between physical inactivity and headache attack frequency (p-trend < 0.001).

Conclusion: Physical inactivity is associated with headache with distinct associations regarding headache subtype, sex, physical activity domain, intensity, and headache frequency.

## AL018 Direct costs for headache disorders. Data from a brazilian health maintenance organization

### M. Peres^1,2^, J. Brito^3^, R. Pires^3^, A. Oliveira^4,5^

#### ^1^IPq-HCFMUSP, São Paulo, Brazil; ^2^Hospital Albert Einstein, São Paulo, Brazil; ^3^NotreDame Intermedica, São Paulo, Brazil; ^4^ABRACES, São Paulo, Brazil; ^5^Universidade de Sao Paulo, Center for Clinical and Epidemiological Research, São Paulo, Brazil

##### **Correspondence:** M. Peres

Background: Primary and secondary headache disorders are significant players in direct and inderect costs worldwide. People who experience migraine have increased healthcare use. Limited information about this scenario is available in Brazil. Objective. To analyze direct costs related to headache disorders in a Brazilian healthcare maintenance organization (HMO).

Methods: Data from the HMO NotreDame Intermedica (Sao Paulo, Brazil) claims related to health care costs due to headache disorders (ICD-10 G43-44, G50, R51) were retrieved, from 2018 to 2020. Results. Outpatient medical consultations were in average 14,148 per year. Emergency room patients were 29,035, emergency room visits were 39,149, with a total cost of R$ 3,442,586 (USD 611,960). Diagnostic exames 19,328 cost R$ 913,764.00 (US$ 162,432.00). 532.584 patients accessed one headache health care provider, 10.8% of the total population insured in 2018, 12.1% in 2019, and 11.4% in 2020, in total R$ 38,994,440.00 (USD 6,931,729.00). Most headaches were coded R 51 (headache not otherwise specified).

Conclusion: Headache disorders are significant contributors for the economic burden in health maintenance organization.

## AL019 Is behaviour of neck pain related to cervical musculoskeletal dysfunction (CMD) or hypersensitivity in migraine?

### Z. Liang^1^, L. Thomas^1^, G. Jull^1^, H. Zareie^2^, J. Treleaven^1^

#### ^1^The University of Queensland, School of Health and Rehabilitation Sciences, St Lucia, Australia; ^2^Royal Brisbane & Women's Hospital, Neurology, Herston, Australia

##### **Correspondence:** Z. Liang

Objective: To investigate if temporal behaviour of neck pain relates to presence of CMD or hypersensitivity.

Methods: Participants (n=108) completed daily online surveys for a month, recording the presence of headache (usual migraine or not) and neck pain. Hypersensitivity was assessed using Allodynia Symptom Checklist (ASC12) and pressure pain thresholds (PPTs). Presence of CMD was determined previously by overall performance across eight cervical measures using cluster analysis for migraineurs, individuals with idiopathic neck pain and healthy controls. Fisher Exact test was used to determine if temporal behaviour categories of neck pain was related to presence of CMD.

Results: In the month, 16 did not experience migraine or neck pain leaving 92 participants (46 usual migraine only, 46 usual migraine plus another headache). All participants reported ictal neck pain. Temporal behaviour of neck pain in participants was categorised as ictal only (n=42), infrequent interictal (n=26), frequent interictal (n=17), undecipherable pattern (n=7). CMD was present in 43% and was unrelated to temporal behaviour of neck pain in all migraineurs. Temporal behaviour of neck pain was also not associated with ASC12 and PPTs. Results were similar for all migraineurs, with or without another headache.

Conclusions: Temporal behaviour of neck pain does not indicate if CMD is present in migraine. Overlapping peripheral and central mechanisms are likely to explain neck pain behaviour.

## AL020 Withdrawn

## AL021 Sex differences in the migraine attack burden and the genetics of migraine

### M. A. Chalmer, I. Callesen, L. J. A. Kogelman, J. Olesen, T. F. Hansen

#### Rigshospitalet-Glostrup, Danish Headache Center, Glostrup, Denmark

##### **Correspondence:** M. A. Chalmer

Objectives & Background: Migraine affects 2-3 times more women than men. Sex differences in the migraine attacks are important for a full understanding of the burden of the migraine attacks in the two sexes. Genetic differences might explain the high attack burden in women. First, we quantitated sex differences in attack frequency, severity, and other clinical parameters, to get the full picture of the migraine attack burden in the two sexes. Secondly, we assessed if genetics might contribute to such differences.

Methods: Cohort of 62,672 individuals (9,212 women, 3,446 men with migraine) from the Danish Blood Donor Study. Migraine diagnosis was made by an extensive questionnaire (specificity and a sensitivity: 93%).

Results: The male-female ratio was 1:2.7. Women did not have a higher migraine attack frequency, but their attacks had a higher severity of pain, longer duration, more often unilateral, pulsating, exacerbated by physical activity, more often accompanied by nausea, vomiting, phonophobia, osmophobia, and allodynia. Women had more headache days unspecified, some of which might have been migraine. Among the 123 genome-wide migraine risk variants, 3 migraine risk variants were significant among women only.

Conclusion: The migraine attack burden per woman is higher than per man. Our genetic analyses show that genetic variants have different effects in women vs men, this may explain the burden difference between sexes. We are currently working on more genetic results.

## AL022 Primary Headache Disorders and Acute Medications Associated With Medication Overuse Headache: Analysis of US Claims Data

### A. H. Ahn, K. J. Shulman, R. G. Iyer, A. Rubin, L. J. Krasenbaum, S. Reshef, S. Lim

#### Teva Branded Pharmaceutical Products R&D, Inc., West Chester, PA, United States

##### **Correspondence:** A. H. Ahn

Objective: Medication overuse headache (MOH), a secondary headache (HA) disorder, arises from frequent acute HA medication use for primary HA disorders (migraine or tension-type HA [TTH]). This retrospective, cross-sectional cohort study using data from the IBM/Watson MarketScan® medical claims database characterized patients (pts) with MOH, their primary HA, and prescribed acute HA medication.

Methods: Pts with MOH were categorized by the following primary HA diagnoses: migraine only, TTH only, other primary HA only, multiple primary HA diagnoses, and "no primary HA specified." Demographic characteristics and prescribed acute HA medication classes (triptans, ergotamines, barbiturates, opioids, non-opioid analgesics, combination analgesics, multiple medications) were evaluated.

Results: Among patients (n=29,124) with a MOH diagnosis, mean age was 41.4 years; 78.8% were female. Single primary HA diagnoses were identified in about half of MOH pts: migraine (45.7%), TTH (5.1%), and "other primary HA" (1.9%). Multiple primary HA diagnoses were found for 18.3% of pts; 29.1% had "no primary HA specified." Among pts overusing acute HA medications in the MOH population (34%), overuse of multiple acute medications (78%) and combination analgesics (33%) were most common (not mutually exclusive).

Conclusions: These results confirm migraine and TTH to be common primary HA disorders for pts with MOH; yet, nearly half present with multiple primary or "no specified" primary HA diagnosis.

## AL023 Sex differences in the pharmacological role of TRPM3 channels and NMDA receptors in human isolated coronary arteries

### E. Rivera-Mancilla^1^, T. de Vries^1^, A. van den Bogaerdt^2^, A. H. J. Danser^1^, C. M. Villalón^3^, A. Maassen van den Brink^1^

#### ^1^Erasmus University Medical Center, Division of Vascular Medicine and Pharmacology, Department of Internal Medicine, Rotterdam, Netherlands; ^2^ETB-BISLIFE, Heart Valve Department, Beverwijk, Netherlands; ^3^Cinvestav-Coapa, Pharmacobiology, Mexico City, Mexico

##### **Correspondence:** E. Rivera-Mancilla

Background: Pregnenolone sulfate (PregS) activates transient receptor potential (TRP) channels and N‑methyl‑D‑aspartate (NMDA) receptors, which are involved in the regulation of the vascular tone and in the pathophysiology of migraine. We investigated the PregS‑induced vasoactive effects and the possible mechanisms involved in human isolated coronary arteries (HCAs).

Methods: In HCAs from both women (n=9, 54±5 years) and men (n=11, 46±5 years), the vasodilatory responses to PregS (0.01-100 μM) were evaluated in the absence or presence of isosakuranetin (TRPM3 antagonist, 5 μM); olcegepant (CGRP receptor antagonist, 1 μM); or MK-801 (NMDA receptor antagonist, 10 μM) to obtain the maximum contractile response (E_max_).

Results: PregS induced concentration-dependent relaxation in HCAs. The E_max_ to PregS was higher in women than in men (E_max_ 73±8% *vs.* E_max_ 46±5%, respectively; p<0.05), which was significantly reduced (p<0.05) in the presence of isosakuranetin (E_max_ 31±6% and E_max_ 17±8%) or olcegepant (E_max_ 31±8% and E_max_ 20±5%). In contrast, the E_max_ to PregS was reduced by MK-801 in women (E_max_ 19±8%, p<0.05) but not in men (E_max_ 45±12%).

Conclusion: (i) PregS-induced relaxation in HCAs is mediated by TRPM3 channels and the CGRP receptor; (ii) there is a differential vasoactive effect of PregS and in the role of NMDA receptors in women and in men. This may provide new therapeutic options to target the sex dimorphism in migraine and its related cardiovascular events.

## AL024 Response to onabotulinumtoxinA in men and women - Results from a multicenter retrospective study

### R. Ornello^1^, F. Ahmed^2^, A. Negro^3^, A. M. Miscio^4^, A. Santoro^4^, A. Alpuente^5^, A. Russo^6^, M. Silvestro^6^, S. Cevoli^7^, N. Brunelli^8^, F. Vernieri^8^, L. Grazzi^9^, C. Baraldi^10^, S. Guerzoni^10^, A. P. Andreou^11^, G. Lambru^11^, K. Kamm^12^, R. Ruscheweyh^12^, M. Russo^13^, P. Torelli^14^, E. Fiatova^15^, N. Latysheva^15^, A. Gryglas-Dworak^16^, M. Straburzyński^17^, C. Butera^18^, B. Colombo^18^, M. Filippi^18^, P. Pozo-Rosich^5^, P. Martelletti^3^, S. Sacco^1^

#### ^1^University of L'Aquila, L'Aquila, Italy; ^2^Hull University Teaching Hospital, Hull, United Kingdom; ^3^Sapienza University of Rome, Rome, Italy; ^4^Fondazione IRCCS "Casa Sollievo della Sofferenza", San Giovanni Rotondo, Italy; ^5^Vall d'Hebron Universiy Hospital, Headache Unit, Neurology Department, Barcelona, Spain; ^6^University of Campania "Luigi Vanvitelli", Naples, Italy; ^7^IRCCS Istituto delle Scienze Neurologiche di Bologna, Bologna, Italy; ^8^Campus Biomedico University, Rome, Italy; ^9^Fondazione IRCCS Istituto Neurologico Carlo Besta, Milan, Italy; ^10^University of Modena and Reggio Emilia, Modena, Italy; ^11^Guy's and St Thomas' Hospital NHS Foundation Trust, London, United Kingdom; ^12^Ludwig Maximilians University, Munich, Germany; ^13^Azienda USL di Reggio Emilia, Reggio Emilia, Italy; ^14^University of Parma, Parma, Italy; ^15^Sechenov University, Moscow, Russian Federation; ^16^Headache Center, Wroclaw, Poland; ^17^Terapia Neurologiczna Samodzielni, Warsaw, Poland; ^18^IRCSS San Raffaele, Milan, Italy

##### **Correspondence:** R. Ornello

Objectives: We aimed to provide data on the effectiveness of onabotulinumtoxinA (BT-A) for chronic migraine (CM) in men compared with women.

Methods: We performed a retrospective analysis on patients with CM treated with BT-A in 16 European centers. We reported the proportion of patients with a ≥50% decrease in monthly headache days (MHDs) – "responders" – during the first 3 BT-A cycles compared with baseline. We also recorded the absolute numbers of MHDs. We then performed exact propensity score matching between men and women, considering age, CM duration, MHDs at baseline, and medication overuse as matching variables.

Results: We included 522 men and 2357 women; men were older than women (47.8±13.2 vs 46.3±12.1 years; P=0.024). The proportion of responders in men was comparable to women during the 1st BT-A cycle (27.7% vs 26.6%; P=0.611), while it was lower during the 2nd (29.2% vs 33.7%; P=0.044) and the 3rd cycle (35.6% vs 41.2%; P=0.018). MHD decrease during treatment was significant in both sexes; however, during the 3rd cycle, mean MHDs were higher in men than in women (15.1±9.7 vs 14.0±8.7; P=0.022). After propensity score matching (84 men vs 113 women), men had more MHDs than women during the 2nd (18.8±10.2 vs 15.6±9.3; P=0.027) and the 3rd cycle (17.7±11.2 vs 13.7±9.4; P=0.010).

Conclusion: Our data suggest that response to BT-A might be lower in men than in women, although significant in both sexes. Sex-specific response to CM treatments merits further study.

## AL025 Opening of BKCa Channels Causes Migraine Attacks: A New Downstream Target for the Treatment of Migraine

### M. A. Al-Karagholi^1^, H. Ghanizada^1^, C. A. Nielsen^1^, C. Skandarioon^1^, J. Snellman^2^, C. Lopez-Lopez^2^, J. M. Hansen^1^, M. Ashina^1^

#### ^1^Danish Headache Center, Copenhagen, Denmark; ^2^Novartis Pharma AG, Basel, Switzerland

##### **Correspondence:** M. A. Al-Karagholi

Potassium channel opening may cause migraine, and we therefore examined the migraine-inducing effect of MaxiPost, a large (big)-conductance calcium-activated potassium (BKCa) channel opener, on migraine induction and cephalic vasodilation in individuals with migraine.

Twenty-six migraine without aura patients were randomly allocated to receive an infusion of MaxiPost or placebo on two study days separated by at least one week. The primary endpoint was the difference in incidence of migraine attacks after MaxiPost compared to placebo. The secondary endpoints were the difference in incidence of headaches and the difference in area under the curve (AUC) for headache intensity scores (0–12 hours), for middle cerebral artery blood flow velocity (VMCA) (0-2 hours), and for superficial temporal artery (STA) and radial artery (RA) diameter.

Twenty-two patients completed the study. Twenty-one of 22 (95%) developed migraine attacks after MaxiPost compared with none after placebo (*P* ˂ 0.0001); the difference of incidence is 95% [95% confidence interval (CI) 86–100%]. The incidence of headache over the 12 hours observation period was higher after MaxiPost day (n= 22) than after placebo (n= 7) (*P* ˂ 0.0001). We found a significant increase of middle cerebral artery blood flow velocity and superficial temporal and radial arteries diameter. Because BKCa channel opening initiate migraine attacks, we suggest that BKCa channel blockers could be potential candidates for novel anti-migraine drugs.

## AL026 Alterations of resting-state periaqueductal gray matter connectivity in tension-type headache

### K. Gecse^1,2^, D. Dobos^1,2^, D. Baksa^1,2^, C. S. Aranyi^3^, M. Emri^3^, G. Kökönyei^1,2,4^, G. Bagdy^1,5^, G. Juhász^1,2^

#### ^1^Semmelweis University, Faculty of Pharmacy, Department of Pharmacodynamics, Budapest, Hungary; ^2^Semmelweis University, SE-NAP2 Genetic Brain Imaging Migraine Research Group, Budapest, Hungary; ^3^University of Debrecen, Faculty of Medicine, Department of Medical Imaging, Division of Nuclear Medicine and Translational Imaging, Debrecen, Hungary; ^4^ELTE Eötvös Lóránd University, Institute of Psychology, Budapest, Hungary; ^5^Semmelweis University, NAP-2-SE New Antidepressant Target Research Group, Budapest, Hungary

##### **Correspondence:** K. Gecse

Background and objective: Tension-type headache (TTH) is the most prevalent type of headache in the world, however its pathophysiology is underexplored. Periaqueductal gray matter (PAG), playing a pivotal role in pain modulation network, may contribute to increased pain sensitivity observed in TTH. Our aim was to determine functional connectivity (FC) alterations of PAG in tension-type headache.

Methods: Using 6-minute resting-state functional MRI, we compared PAG-FC between 32 TTH subjects (23 females), during pain-free state and 32 healthy controls (21 females) using Statistical Parametric Mapping (SPM12) toolbox in MATLAB.

Results: Increased FC correlation was found between PAG and clusters in the superior medial part of frontal gyrus (family-wise error corrected p: pFWE<0.001) and right triangular part of inferior frontal gyrus (pFWE<0.001) in TTH patients compared to controls. In addition, decreased FC correlation was revealed between PAG and cuneus (pFWE<0.001) and left lingual gyrus (pFWE<0.036) in TTH patients compared to controls.

Conclusions: Our results suggest a disrupted PAG-FC with regions that modulate pain-related information integration and affective dimension of pain in TTH patients compared to non-headache controls. These alterations could be a consequence of increased pain sensitivity induced by repeated headaches, however the effect of headache frequency should be further investigated.

## AL027 Hypersensitivity to Calcitonin Gene–Related Peptide in Post-Traumatic Headache

### H. Ashina, A. Iljazi, H. Al-Khazali, C. Christensen, F. M. Amin, M. Ashina, H. Schytz

#### Danish Headache Center, Neurology, Copenhagen, Denmark

##### **Correspondence:** H. Ashina

Objective: To demonstrate that calcitonin gene–related peptide (CGRP) induces headache exacerbation with migraine-like features in patients with persistent post-traumatic headache (PTH) attributed to mild traumatic brain injury (TBI).

Methods: A randomized, double-blind, placebo-controlled, two-way crossover study was conducted. Analyses were intention-to-treat. Eligible patients were aged 18 to 65 years and had a history of persistent PTH after mild TBI for at least 12 months. Patients were randomized to receive an intravenous infusion of 1.5μg/min of CGRP or placebo (isotonic saline) over 20 minutes on two separate experimental days. A 12-hour observational period was used to evaluate the following outcomes: (1) difference in incidence of headache exacerbation with migraine-like features and (2) difference in area under the curve for headache intensity scores.

Results: Thirty patients were randomized and completed the study. During the 12-hour observational period, 21 of 30 patients (70%) developed headache exacerbation with migraine-like features after CGRP, compared with 6 patients (20%) after placebo (p < 0.001). The baseline-corrected area under the curve for headache intensity scores was significantly larger after CGRP, compared with placebo (p < 0.001).

Conclusions: Patients with persistent PTH are hypersensitive to CGRP, which underscores its pathophysiological importance.

**Fig. 1 (abstract AL027).**
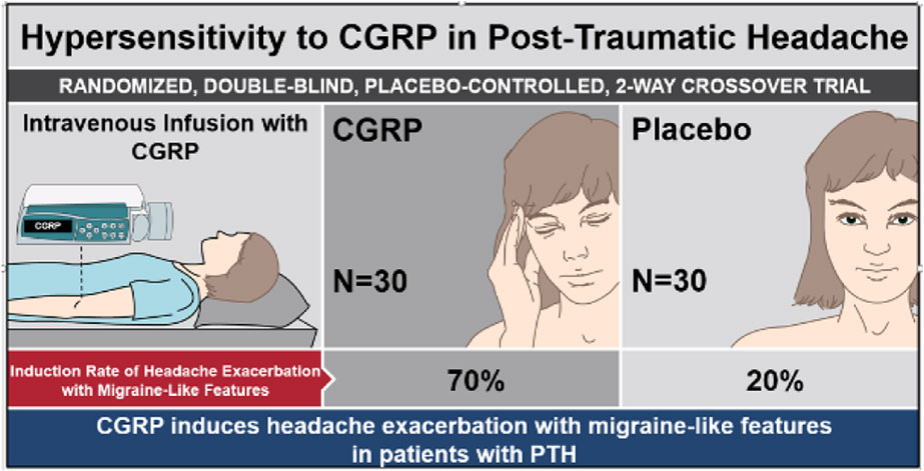
See text for description

## AL028 Exploring the role of the pons and hypothalamus in migraine progression

### R. Messina^1^, M. A. Rocca^1^, P. Valsasina^2^, P. Misci^2^, B. Colombo^3^, I. Cetta^1^, M. Filippi^1^

#### ^1^San Raffaele Scientific Institute, Neuroimaging Research Unit and Neurology Unit, Milan, Italy; ^2^San Raffaele Scientific Institute, Neuroimaging Research Unit, Milan, Italy; ^3^San Raffaele Scientific Institute, Neurology Unit, Milan, Italy

##### **Correspondence:** R. Messina

Objective: The hypothalamus and dorsal pons could be putative drivers of migraine attacks. Here, we explored whether longitudinal hypothalamic and pontine resting state (RS) functional connectivity (FC) changes might influence migraine progression over time.

Methods: Ninety-one headache-free episodic migraine patients and 73 controls underwent RS functional magnetic resonance imaging. Twenty-three patients and 23 controls were re-examined after 4 years. A seed-based correlation approach was used to study hypothalamic and pontine RS FC changes, separately.

Results: After 4 years, migraine patients developed an increased FC between the hypothalamus and orbitofrontal gyrus (OFG), bilaterally, as well as between the left pons and left cerebellum. They also experienced decreased RS FC between the right hypothalamus and ipsilateral lingual gyrus. At baseline, the decreased hypothalamic-lingual gyrus RS FC correlated with higher migraine attack frequency. At follow-up, higher hypothalamic-OFG RS FC correlated with lower migraine attack frequency and higher pontine-cerebellar RS FC correlated with an increased number of migraine attacks over the years. A significant negative association between the pontine-cerebellar RS FC and the hypothalamic-lingual RS FC was found in migraine patients.

Conclusion: Our findings support the presence of a common functional framework comprising the hypothalamic, pontine, cerebellar and visual networks that might influence migraine progression.

## AL029 Stability of functional MRI-based migraine diagnosis: an internal validation study

### B. Y. Park^1^, S. Cho^2^, C. S. Chung^3^, M. J. Lee^3^

#### ^1^Department of Data Science, Department of Data Science, Incheon, South Korea; ^2^Uijeongbu Eulji Medical Center, Neurology, Uijeongbu, South Korea; ^3^Samsung Medical Center, Sungkyunkwan University School of Medicine, Neurology, Seoul, South Korea

##### **Correspondence:** B. Y. Park

Objective: To test the reliability of functional MRI (fMRI)-based diagnosis of migraine using an internal validation set which was followed up with 1-year interval.

Methods: We prospectively recruited 50 patients with episodic migraine and 50 age-sex-matched healthy controls (HCs). Participants underwent resting-state fMRI at baseline and after 1 year. Interictal pairs (patients and matched HCs) at baseline were used to build a diagnostic model using regularized graph-based functional connectivity measures and non-linear classifier. We tested the model with 5-fold nested cross-validation and repeated 100 times with different training/test dataset. The diagnostic performance was validated using 1-year follow-up data of the same pairs and interictal pairs.

Results: Among 100 participants, 46 patients and 43 HCs completed the follow-up fMRI scan. The training set included 16 pairs (n=32) and showed a diagnostic accuracy of 79.4 ± 4.60% across repetitions. Nine interictal patients at baseline were switched to peri-ictal/ictal at follow up, and the diagnostic accuracy decreased to 55.2%. When 21 patients who were interictal at follow up and matched HCs were tested, the diagnostic accuracy was 66.7%.

Conclusions: Whole-brain connectivity obtained from resting-state fMRI may not be stable over time, and the accuracy of fMRI-based diagnosis is subject to migraine phases. A rigorous validation should be performed before the fMRI can be used as a diagnostic modality for migraine.

## AL030 T2* Contrast Changes in Post-traumatic Headache

### S. Nikolova^1^, T. J. Schwedt^1^, C. Chong^1^, G. Dumkrieger^1^, T. Wu^2^, V. Berisha^2^, J. Li^3^, K. Ross^4^

#### ^1^Mayo Clinic, Neurology, Phoenix, AZ, United States; ^2^Arizona State University, Phoenix, AZ, United States; ^3^Georgia Tech, Atlanta, GA, United States; ^4^PVAHCS, Phoenix, AZ, United States

##### **Correspondence:** S. Nikolova

Background and Objective: To compare brain T2* contrast, commonly associated with iron accumulation, between individuals with post traumatic headache (PTH) attributed to mild traumatic brain injury (mTBI), and healthy age-balanced controls (HC). Additionally, we aimed to interrogate whether in subjects with PTH changes in T2* contrast associates with headache frequency and number of mTBIs.

Methods: We included 20 individuals with PTH (mean age=41.3; SD=11.9) following mTBI and 20 healthy controls (mean age= 40.5; SD= 12.4). Between group differences on T2* were evaluated within SPM12 using cluster volume thresholding (>25 mm3; p< 0.01)

Results: Compared to HC, subjects with PTH had less T2* contrast in the right hippocampus, right amygdala, right insula, the anterior commissure, the pons as well as the right supramarginal, left temporal and left occipital areas. In the PTH group T2* contrast in the right supplemental motor area, bilateral precuneus and bilateral insula negatively correlated with headache frequency and right supramarginal T2* contrast negatively correlated with number of mTBIs.

Conclusion: PTH was associated with lower T2* contrast indicating higher iron accumulation in cortical, limbic and brainstem areas. For subjects with PTH, there was a negative association between T2* contrast with headache frequency and number of mTBIs suggesting that increased headache burden and number of mTIBs associate with accumulative iron load.

**Table 1 (abstract AL030).**
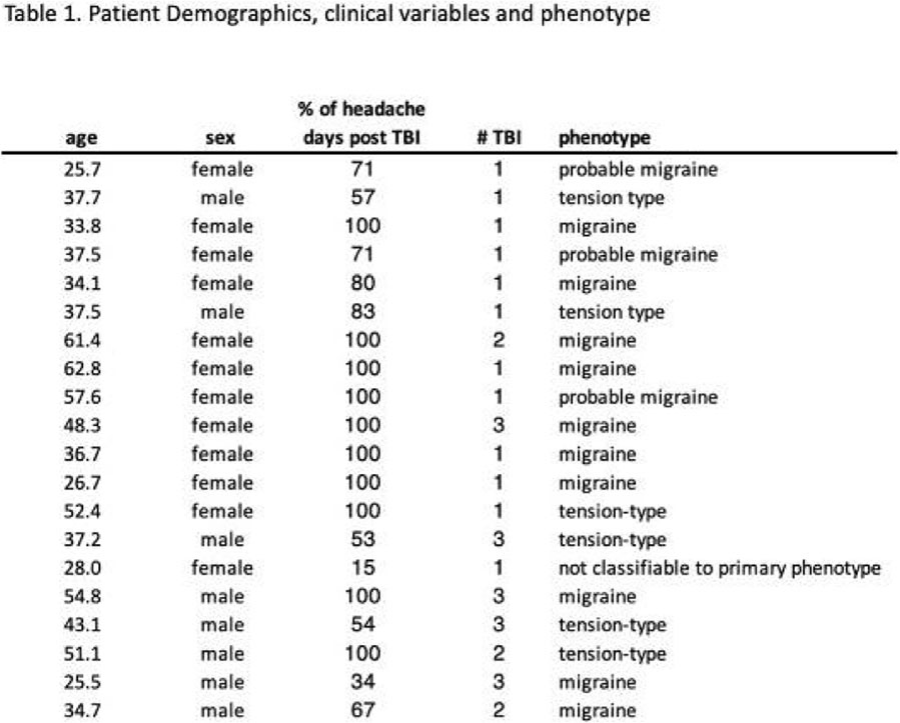
See text for description

**Fig. 2 (abstract AL030):**
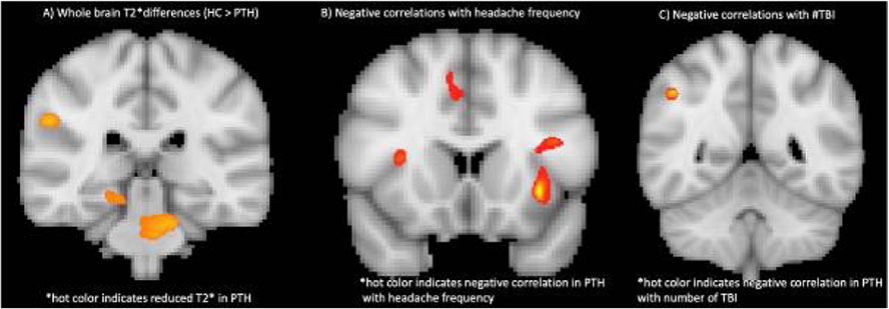
. See text for description

## AL031 Part I: The impact of diet-induced obesity on intracranial pressure in a rodent model of non-traumatic intracranial hypertension

### I. Israelsen, C. James Westgate, R. H. Jensen, S. Eftekhari

#### Danish Headache Center, Copenhagen, Denmark

##### **Correspondence:** I. Israelsen

Objectives: Idiopathic intracranial hypertension (IIH) is a disorder characterized by increased intracranial pressure (ICP) and strongly linked to obesity. We aimed to explore the impact of diet-induced obesity (DIO) on ICP with the dual goal of developing a model for non-traumatic raised ICP.

Methods: 28 female Sprague-Dawley rats received high-fat diet (60% fat) or control diet for 17 weeks. Selected rats were implanted with telemetric probe for continuous ICP recordings for 30 days. At baseline body composition was measured with dual energy x-ray absorptiometry. Molecular analysis of aquaporin 1 (AQP1) and Na-K-2Cl-1 cotransporter (NKCC1) was studied in choroid plexus (CP) from the implanted rat after 30 days.

Results: Mean ICP was raised by 55 % in the DIO rats over 14 days (2.32±1.65 vs 4.57±1.60 mmHg, P=0.019). ICP was also correlated with abdominal fat percentage (r=0.54, P=0.016). We observed an increase in the spectral power of ICP wavelengths at 0-0.25Hz representing non-respiratory slow ICP waves (0.24±0.09 vs 1.09±0.3 mmHg2, P=0.02) seven days after surgery. The DIO rats also exhibited a tendency to increased protein expression in the ratio of glycosylated AQP1 to total expression (0.46±0.03 vs 0.59±0.06 AU, P=0.084). NKCC1 protein expression was also changed by 1.3-fold (3.4±0.78 vs 4.7±0.92 AU, P=0.43).

Conclusions: DIO leads to raised ICP in rats. This may serve as model for non-traumatic raised ICP to expand the knowledge regarding the pathophysiology of IIH.

## AL032 Part III: Neuroretinal changes in a rodent model of diet-induced obesity with raised intracranial pressure

### S. Hagen^1,2,3^, C. S. J. Westgate^2^, I. Israelsen^2,3^, S. Hamann^1,3^, R. H. Jensen^2,3^, S. Eftekhari^2^

#### ^1^Rigshospitalet-Glostrup, Department of Ophthalmology, Glostrup, Denmark; ^2^Rigshospitalet-Glostrup, Danish Headache Center, Department of Neurology, Glostrup, Denmark; ^3^University of Copenhagen, SUND, Copenhagen, Denmark

##### **Correspondence:** S. Hagen

Objectives: The mechanisms driving increased intracranial pressure (ICP) and neuroretinal degeneration have not been clarified. We evaluated the structural changes of the peripapillary retina in a newly developed rat model with of non-traumatic raised ICP using diet-induced obesity (DIO) mimicking key features of IIH.

Methods: 14 DIO and 10 control Sprague-Dawley rats were included for dual energy x-ray absorptiometry (DEXA, evaluating abdominal fat percentage) and spectral domain optical coherence tomography (OCT) scans prior to implantation of ICP telemeter. OCT-scans were analyzed using a semi-automatic segmentation software and the retinal nerve fiber layer (RNFL) thickness was measured in a blinded design. Histology and measurement of retinal nerve fiber bundles (RNFB) thickness in the anterior preliminary region of the optic cup were performed.

Results: DIO animals with raised ICP (4.40±0.85 mmHg) had significant thicker RNFL compared to control animals (28.82±0.61 vs 24.85±1.09 μm, p=.003). We found positive correlations between RNFL thickness and ICP (r=0.64, P=.006), body weight (r=0.57, p=.005) and DEXA abdominal fat percentage (r=0.47, p=.021). DIO animals showed thinner RNFB compared to control animals in histological sections of the optic nerve head (p=.014).

Conclusion: DIO rats with elevated ICP develop peripapillary RNFL swelling followed by neuroretinal degeneration as seen in acute IIH patients with papilledema, illustrating the high risk of permanent damage.

## AL033 Amylin analog pramlintide induces migraine-like attacks in patients

### H. Ghanizada

#### Danish Headache Center, Neurology, Taastrup, Denmark

Objective: Migraine is a prevalent and disabling neurological disease. Its genesis is poorly understood and there remains unmet clinical need. We aimed to identify mechanisms and thus novel therapeutic targets for migraine using human models of migraine and translational models in animals, with emphasis on amylin, a close relative of calcitonin gene-related peptide (CGRP)

Methods: Thirty-six migraine without aura patients were enrolled in a randomized, double-blinded, two-way, cross-over, positive-controlled clinical trial study to receive infusion of an amylin analogue pramlintide or human αCGRP on two different experimental days. Furthermore, translational studies in cells and mouse models, and rat and human tissue samples were conducted.

Results: Thirty patients (88%) developed headache after pramlintide infusion, compared to thirty-three (97%) after CGRP (*p* = 0.375). Fourteen patients (41%) developed migraine-like attacks after pramlintide infusion, compared to nineteen patients (56%) after CGRP (*p* = 0.180). The pramlintide-induced migraine-like attacks had similar clinical characteristics to those induced by CGRP. There were differences between treatments in vascular parameters.

Interpretation: Our findings propose amylin receptor agonism as a novel contributor to migraine pathogenesis. Greater therapeutic gains could therefore be made for migraine patients through dual amylin and CGRP receptor antagonism, rather than selectively targeting the canonical CGRP receptor.

## AL034 New daily persistent headache: clinical characteristics and treatment responses in 366 patients

### S. Cheema^1^, A. Stubberud^1,2,3^, K. Rantell^4^, P. Nachev^2^, E. Tronvik^3^, M. Matharu^1^

#### ^1^UCL Queen Square Institute of Neurology, Headache and Facial Pain Group, London, United Kingdom; ^2^UCL Queen Square Institute of Neurology, High Dimensional Neurology Group, London, United Kingdom; ^3^NTNU Norwegian University of Science and Technology, Department of Neuromedicine and Movement Sciences, Trondheim, Norway; ^4^UCL Queen Square Institute of Neurology, Education Unit, London, United Kingdom

##### **Correspondence:** S. Cheema

Objective: To describe clinical characteristics and treatment responses in a large cohort of patients with new daily persistent headache (NDPH).

Methods: Descriptive analysis of data extracted from routinely collected clinical records in consecutive patients with primary NDPH seen in a secondary and tertiary headache clinic between 2007 and 2019.

Results: 366 patients met inclusion criteria, mean age 37.9 years, 62.6% female. 140 (38.8%) had an identifiable precipitant, most commonly a flu-like viral illness. 23 (6.3%) had a thunderclap headache at onset.

According to ICHD-3 criteria, 63.7% had characteristics of chronic migraine (CM), 27.6% of chronic tension type headache (CTTH), and 8.7% met neither criteria.

The most common comorbid diagnoses were depression, anxiety, and joint hypermobility disorder. 87.1% and 87.2% were within the severely disabled range on the Headache Impact Test-6 and Migraine Disability Assessment scores respectively.

Response to the majority of acute, preventive, and injectable treatments was poor, with only a small proportion of patients experiencing >30% improvement in either headache severity or frequency. The most effective preventive treatments were doselupin in 37 patients (45.7% responders, 95% CI 34.8-56.5%) and onabotulinumtoxinA in 55 patients (34.2% responders, 95% CI 26.8-41.5%).

Conclusions: NDPH is a highly disabling disorder, which may have features of CM or CTTH, but is often refractory to treatments used in CM and CTTH.

**Fig. 1 (abstract AL034).**
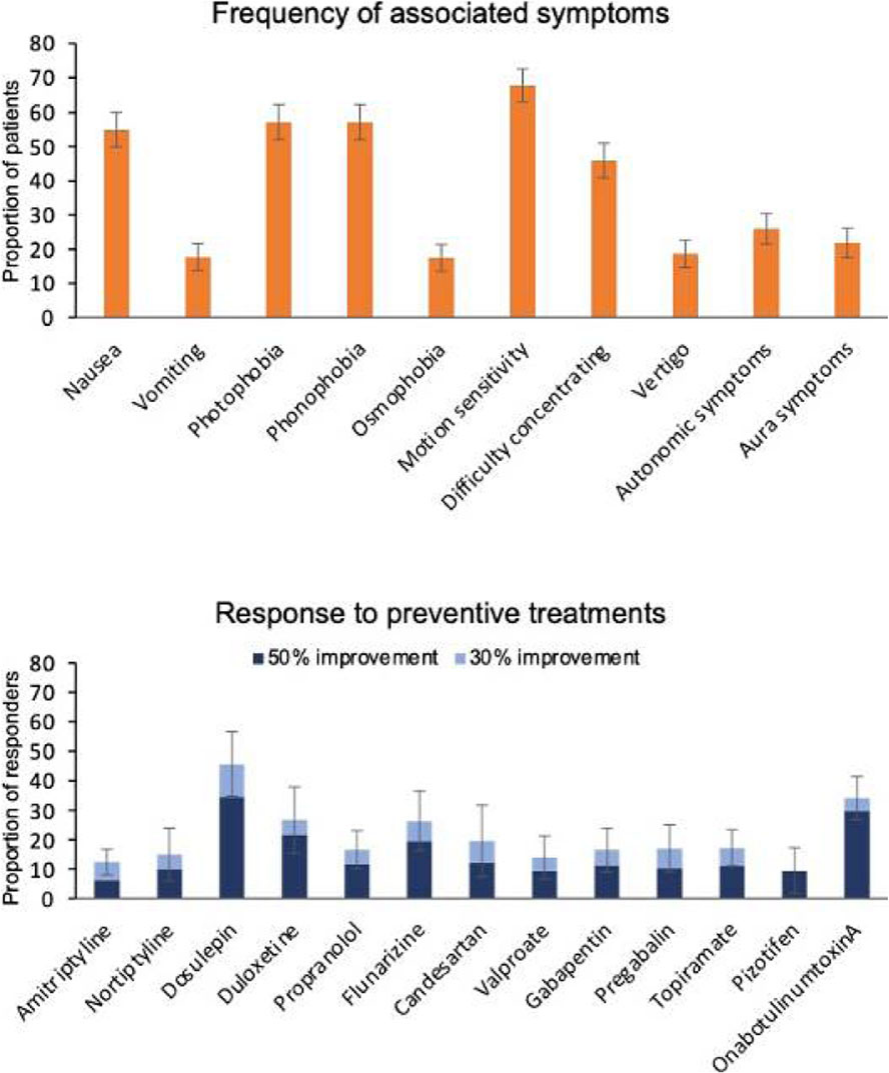
See text for description

## AL035 Using Optical Coherence Tomography as a Surrogate of Measurements of Intracranial Pressure in Idiopathic Intracranial Hypertension

### S. P. Mollan^1^, V. Vijay^2^, J. Mitchell^2^, E. Bilton^1^, Z. Alimajstorovic^2^, K. Markey^2^, A. Fong^1^, J. Walker^1^, H. Lyons^1^, A. Yiangou^2^, G. Tsermoulas^3^, K. Brock^4^, A. J. Sinclair^2^

#### ^1^University Hospitals Birmingham, Neuro-ophthalmology, Birmingham, United Kingdom; ^2^University of Birmingham, Metabolic neurology, Birmingham, United Kingdom; ^3^University Hospitals Birmingham, Department of Neurosurgery, Birmingham, United Kingdom; ^4^University of Birmingham, Institute of Cancer and Genomic Sciences, Birmingham, United Kingdom

##### **Correspondence:** J. Mitchell

Background: To determine whether Optical Coherence Tomography (OCT) of the optic nerve head in papilloedema could act as a surrogate measure of intracranial pressure (ICP).

Methods: This is a longitudinal cohort study using data collected from three randomised controlled trials, between from April 1^st^, 2014 to August, 1^st^ 2019. OCT imaging and automated perimetry was followed immediately by ICP measurement on the same day. Cohort 1 utilised continuous sitting telemetric ICP monitoring (Raumedic Neurovent P-tel device) on one visit. Cohort 2 were evaluated at baseline, 3, 12 and 24 months and underwent lumbar puncture assessment of ICP.

Results: 104 patients were recruited. Amongst cohort 1 (n=15), the range of OCT protocols were evaluated and optic nerve head central thickness was found to be most closely associated with ICP (right eye: p=0.017, r = 0.60; left eye: p = 0.002; r = 0.73). Subsequently, cohort 2 (n=89) confirmed the correlation between central thickness and ICP longitudinally (12 and 24 months). Finally, bootstrap surrogacy analysis noted a positive association between central thickness and ICP treatment effects at all time points (e.g. at 12 months, an decrease in central thickness of 50μm predicted a decrease in ICP of 5 cmCSF).

Conclusion: OCT optic nerve head volume measures reproducibly correlates with ICP and surrogacy analysis demonstrated its ability to inform ICP changes. This data suggests that OCT can non-invasively predict ICP.

## AL036 CGRP and the AMY1 receptor: Identifying Targets for Migraine

### T. Rees^1,2^, S. O'Carroll^3^, C. Le Foll^4^, T. Lutz^4^, C. Walker^1,2^, D. Hay^1,2,5^

#### ^1^University of Auckland, School of Biological Sciences, Auckland, New Zealand; ^2^Maurice Wilkins Centre for Molecular discovery, Auckland, New Zealand; ^3^University of Auckland, Faculty of Medical and Health Sciences, Auckland, New Zealand; ^4^University of Zurich, Institute of Veterinary Physiology, Zurich, Switzerland; ^5^University of Otago, Department of Pharmacology and Toxicology, North Dunedin, New Zealand

##### **Correspondence:** T. Rees

Objective: Calcitonin gene-related peptide (CGRP) has been proposed to act in an auto-regulatory manner in the trigeminal ganglia (TG), to increase CGRP expression and responsiveness. However, in the TG, CGRP and its canonical receptor, the CGRP receptor, are reportedly not co-expressed. The AMY1 receptor, a dual receptor for both CGRP and amylin, is also reported to be expressed in the TG. Therefore, we aimed to compare the relative distribution of CGRP and amylin with the AMY1 receptor component, calcitonin receptor (CTR), in the TG.

Methods: CGRP, amylin and CTR antibodies were thoroughly validated for specificity and cross-reactivity. Mouse, rat, and human TG sections were then double or triple-immunostained with the lead antibodies and neuronal markers, β tubulin III and NF200.

Results: Anti-CGRP antibodies displayed good specificity in immunoblots and immunohistochemistry. The lead CTR antibodies strongly detected CTR in cell models and displayed a marked loss of staining in KO mouse models. CTR and CGRP immunoreactivity frequently colocalised in rodent TG sections, primarily in C-fibre neurons, and infrequently in A-fibre neurons. Interestingly, anti-amylin antibodies frequently displayed cross-reactivity with CGRP, and lead antibodies demonstrated a lack of amylin-like immunoreactivity.

Conclusions: In TG C-fibre neurons, CGRP could be acting via the AMY1 receptor in an autocrine manner. Circulating amylin may also activate this receptor in the TG.

## AL037 The Effects of P2X7 Antagonism on Neuroinflammation Following Optogenetically-Triggered Cortical Spreading Depression

### B. Uzay^1^, B. Donmez-Demir^1^, S. Yilmaz-Ozcan^1^, E. Eren-Kocak^1,2^, M. Yemisci^1,3^, T. Dalkara^1^, H. Karatas^1^

#### ^1^Hacettepe University, Institute of Neurological Sciences and Psychiatry, Ankara, Turkey; ^2^Hacettepe University, Faculty of Medicine, Dep of Psychiatry, Ankara, Turkey; ^3^Hacettepe University, Faculty of Medicine, Dep of Neurology, Ankara, Turkey

##### **Correspondence:** B. Uzay; H. Karatas

Cortical spreading depression(CSD) is the electro-physiological correlate of migraine aura that causes opening of pannexin-1 megachannels and ATP release.CSD triggers parenchymal neurogenic inflammation(PNI) preceding meningeal inflammation which is the cause of migraine headache.P2X7-receptors are purinergic receptors activating pro-inflammatory cascades when extracellular ATP increased.We aim to investigate the effects of purinergic P2X7-receptors on CSD-induced PNI using a potent, selective and BBB permeable P2X7 antagonist(JNJ-47965567). Experiments were performed on Thy1-ChR2 transgenic mice and CSD is optogenetically triggered via a 450nm laser source without craniotomy. In the experimental group 30mg/kg JNJ-47965567(or its vehicle) was intraperitoneally administered 15-min prior to CSD induction. After CSD, animals were perfused with 4% PFA, brains cryosectioned. Sections were immune-stained with NF-kappa B-p65 and P2X7, costained with S100b and/or NeuN; imaged with confocal microscope.In cortex and subcortical structures (thalamus, hypothalamus, striatum, hippocampus) following CSD, NF-kB-p65 is translocated to the nucleus of the astrocytes and P2X7 receptor signal is increased particularly in neurons.These effects of CSD, indicating PNI are all reversed by P2X7 antagonist. P2X7 receptors play a role in neurogenic inflammation in the cortex and subcortical structures following CSD.P2X7 receptors can be used as a target in the prophylaxis and treatment of migraine headache.

## AL039 Insights into the natural history of spontaneous intracranial hypotension from infusion testing

### J. Beck^1^, C. Fung^1^, D. Cipriani^1^, T. Dobrocky^2^, A. Raabe^3^, E. I. Piechowiak^2^, L. Häni^3^

#### ^1^University of Freiburg, Germany, Neurosurgery, Freiburg, Germany; ^2^University of Bern, Neuroradiology, Bern, Switzerland; ^3^University of Bern, Neurosurgery, Bern, Switzerland

##### **Correspondence:** J. Beck

Objective

To assess the pathophysiologic changes in spontaneous intracranial hypotension

(SIH) based on measures of CSF dynamics and on the duration of symptoms.

Methods: We included consecutive patients from 2012 to 2018. CSF leak was confirmed if extrathecal contrast spillage was seen on imaging after intrathecal contrast application, or dural breach was detected intraoperatively. We divided patients with a CSF leak into 3 groups depending on the symptom duration: ≤10, 11–52, and >52 weeks. Clinical characteristics and measures of CSF fluid dynamics obtained by computerized lumbar infusion testing (LIT) were analyzed over time.

Results: Among the 137 patients included, 69 had a confirmed CSF leak. Whereas 93.1% with <10 weeks of symptoms displayed typical orthostatic headache, only 62.5% with >10 weeks of symptoms did (p = 0.004). LIT revealed differences between groups with different symptom duration for CSF outflow resistance (p < 0.001), lumbar baseline pressure (p = 0.013), lumbar plateau pressure (p < 0.001), pressure–volume index (p = 0.001), elastance (p < 0.001), and CSF production rate (p = 0.001). Compared to the reference population, only patients with acute symptoms showed a significantly altered CSF dynamics profile.

Conclusion: A CSF leak dramatically alters CSF dynamics in the acute phase of SIH, but these dynamics normalize with long lasting symptoms. There was an association between the clinical presentation and changes in CSF dynamics.

## AL040 IIH Pressure Med: A randomised, sequential, trial of the effect on intracranial pressure of five drugs commonly used in Idiopathic Intracranial Hypertension

### J. Mitchell^1^, S. P. Mollan^2^, J. Walker^1^, H. Lyons^1^, A. Yiangou^1^, Z. Alimajstorovic^1^, O. Grech^1^, B. R. Wakerley^3^, G. Tsermoulas^4^, K. Brock^5^, A. J. Sinclair^1^

#### ^1^University of Birmingham, Metabolic Neurology, Birmingham, United Kingdom; ^2^University Hospitals Birmingham, Ophthalmology, Birmingham, United Kingdom; ^3^University Hospitals Birmingham, Neurology, Birmingham, United Kingdom; ^4^University Hospitals Birmingham, Neurosurgery, Birmingham, United Kingdom; ^5^University of Birmingham, Birmingham, United Kingdom

##### **Correspondence:** J. Mitchell

Background: Limited data exists to guide treatment of idiopathic intracranial hypertension (IIH). We examined the effects of therapeutics on reducing intracranial pressure (ICP) in IIH.

Methods: Randomised, sequential, open label trial in women with active IIH. Participants received 2 weeks of acetazolamide (2g), amiloride (10mg), furosemide (80mg), spironolactone (200mg) and topiramate (100mg). Treatment order was randomised, minimum 1 week drug washout between rounds. ICP was recorded before and after with telemetric, intraparenchymal ICP monitors (Raumedic, Hembrechts, Germany). Headache frequency and severity were recorded by diary. Analysis was by hierarchical regression.

Results: 14 participants were recruited. BMI 38.1(6.2) kg/m2, ICP 30.6(5.1) cmCSF at baseline. ICP fell significantly with 4 drugs acetazolamide mean -3.31mmHg(SE 0.95), p=0.0009, furosemide -3.03(0.88), 0.0011, spironolactone -2.71(0.88), 0.0033, topiramate -2.29(0.85), 0.0095. There was no significant difference between drugs. There was no significant improvement in headache. Side-effects were common with acetazolamide (92%) and topiramate (92%).

Conclusions: Acetazolamide, furosemide, spironolactone and topiramate marginally reduced ICP, but there was no statistical difference between treatments and no improvement in headache. There were significant side-effects, especially with acetazolamide and topiramate. Therapeutics with greater efficacy and less side effects are an unmet need in IIH.

## AL041 Characterizing Preventive Treatment Gaps in Migraine: Results From the CaMEO Study

### S. J. Nahas^1^, D. C. Buse^2^, S. Hutchinson^3^, M. L. Reed^4^, K. M. Fanning^4^, B. Dabruzzo^5^, R. B. Lipton^2^

#### ^1^Thomas Jefferson University, Jefferson Headache Center, Philadelphia, PA, United States; ^2^Albert Einstein College of Medicine, Bronx, NY, United States; ^3^Orange County Migraine and Headache Center, Irvine, CA, United States; ^4^Vedanta Research, Chapel Hill, NC, United States; ^5^AbbVie, Madison, NJ, United States

##### **Correspondence:** S. J. Nahas

Objective: To characterize self-reported use of preventive medications for migraine and treatment gaps in a representative US sample.

Methods: CaMEO was a web-based survey (Sept 2012-Nov 2013) of people who met modified migraine criteria consistent with *International Classification of Headache Disorders, 3rd edition.* Potentially preventive-eligible respondents for traditional oral preventive medications (≥4 monthly headache days [MHDs]) were categorized by oral preventive use status, and prespecified treatment gaps were characterized.

Results: Among respondents with ≥4 MHDs, 80.2% (5275/6579) reported never using, 9.8% (642/6579) reported currently using, and 10.1% (662/6579) reported previous but not current use of a daily oral migraine preventive. Among never users, 61.8% (3259/5275) were interested in trying a daily oral prescription preventive. Among current users, 26.0% (167/642) reported that their preventive medication did not work at all, or only prevented a few attacks. Additionally, 85.7% (550/642) were somewhat/very interested in trying a different daily oral preventive medication. Among discontinued users, factors contributing to discontinuation included safety and tolerability concerns (44.6% [295/662]) and insufficient efficacy (34.8% [263/662]).

Conclusion: Less than 10% of potentially preventive-eligible respondents were currently using a migraine preventive. Discontinuations were largely attributed to safety and tolerability concerns, and lack of efficacy.

## AL042 Treatment with calcitonin gene-related peptide antibodies modifies brainstem excitability and habituation to nociceptive trigeminal stimulation in migraineurs

### A. Thiele^1^, L. Klehr^1^, S. Strauß^1^, A. Angermaier^1^, M. Kronenbuerger^2^, R. Fleischmann^1^

#### ^1^University Medicine Greifswald, Department of Neurology, Greifswald, Germany; ^2^Medical School OWL, Department of Neurology, Bielefeld, Germany

##### **Correspondence:** R. Fleischmann

Objective: Calcitonin gene-related peptide ligand/receptor (CGRP) antibodies effectively reduce headache frequency in migraineurs. It is understood that they act peripherally, which raises the question whether treatment merely interferes with the last stage of headache generation or, alternatively, causes secondary adaptations in the central nervous system and is thus disease modifying.

Methods**:** This interim analysis includes fifteen episodic migraineurs (14 female, 48±13 years old), who completed all study visits until March 2021 and received assessments of the nociceptive blink reflex (R2 component, 10 trials, 6 stimuli/trial) before (V0) and three months (V1) after treatment with CGRP antibodies started. The R2 area (R2a) and habituation (R2h; gradient of R2a against stimulus order) of the stimulated/non-stimulated side (_s/_ns) following repeated supraorbital stimulation provide a direct readout of brainstem excitability and habituation as key mechanisms in migraine.

Results**:** All patients showed a substantial reduction of headache days/month (V0: 12.3±3.7, V1: 5.9±4.0) and disability (HIT-6, V0: 65.1±2.9, V1: 55.2±8.6). R2a significantly decreased (R2a_s: -46%, *p*=.038; R2a_ns: -39%, *p*=.014) and R2h significantly increased (R2h_s: β=-.33, *p*=.016; R2h_ns: β=-2.6, *p*=.041) from V0 to V1.

Conclusion: We provide novel evidence that treatment with CGRP antibodies is disease modifying. The nociceptive blink reflex may provide a biomarker to monitor central disease activity.

## AL043 Long-term Fremanezumab Treatment Over 6 to 12 Months Shows No Effect on Blood Pressure in Migraine Patients

### S. Nägel^1^, J. M. Cohen^2^, Y. Kessler^2^, V. Ramirez Campos^2^, X. Ning^2^, S. Barash^2^, S. J. Nahas^3^

#### ^1^Department of Neurology, Martin Luther University Halle, University Hospital Halle (Saale), Halle, Germany; ^2^Teva Branded Pharmaceutical Products R&D, Inc., West Chester, PA, United States; ^3^Thomas Jefferson University, Philadelphia, PA, United States

##### **Correspondence:** S. Nägel

Objective: To assess long-term effects of fremanezumab on blood pressure (BP) in clinical trial participants (CTPs) from a 12-month (mo) long-term extension study (HALO LTS) and 6-mo study (FOCUS).

Methods: In HALO LTS, CTPs received quarterly (QTY; 675mg) or monthly (MLY; 225mg) fremanezumab for 12 mo. In FOCUS, CTPs received QTY or MLY fremanezumab or placebo (PBO) for a 3-mo double-blind (DB) period; all CTPs received MLY fremanezumab during a subsequent 3-mo open-label (OL) extension. Adverse events (AEs) for BP changes and changes in systolic BP (SBP; in mmHg) and diastolic BP (DBP; in mmHg) over time (in CTPs with hypertension [HTN] or taking anti-HTN concomitant medications in HALO LTS) were evaluated.

Results: In HALO LTS, AEs of increased DBP and decreased SBP were reported for 1 CTP each (QTY group). Mean BP values were comparable or lower during treatment than at baseline (BL) in CTPs with a history of HTN (SBP: BL, 128.2; end of treatment [EOT], 128.0; DBP: BL, 82.3; EOT, 81.6) and concomitant anti-HTN treatment (SBP: BL, 131.0; EOT, 128.6; DBP: BL, 84.0; EOT, 82.3). In FOCUS, 1 CTP (QTY group) had an AE of decreased BP during the DB period and 1 had increased BP during the OL extension. Overall, BP was maintained or decreased slightly during treatment (mean SBP: BL, 122.7; EOT, 122.7; mean DBP: BL, 78.6; EOT, 78.3).

Conclusion: With long-term fremanezumab treatment in HALO LTS and FOCUS, there were few BP-related AEs, and SBP and DBP changed minimally with treatment.

## AL044 Erenumab vs galcanezumab in a very difficult-to-treat migraine population. Efficacy and safety

### D. I. Samuel, C. Nieves Castellanos, L. L. Mireya, P. G. Julia, N. M. María José

#### Hospital Universitari i Politècnic La Fe, Headache Unit - Neurology, Valencia, Spain

##### **Correspondence:** D. I. Samuel

Objective: To evaluate the efficacy and safety of erenumab (E) vs galcanezumab (G) as preventive treatments in a very difficult-to-treat migraine population.

Methods: Post-authorization study, phase IV, no financial support. Prospective registry of patients using E or G since its approval in our centre. Measures of efficacy: MHD (migraine Headache Days), MtuD (Monthly triptan-use Days), MOH rates, VAS and PRO as MIDAS, HIT-6, PCS and MsQol are evaluated at baseline, and each 3 months. Adverse events are reported.

Results: 220 patients reached at least 3 months. Age 47"46 years, women 81"55%, 89% Chronic Migraine, 15 years with CM. Have failed 5"74 previous preventive treatments. Baseline: 20"57 MHD, 17"13 MtuD, 87"73% MOH; MIDAS 93, HIT-6 68"86, PCS 32"74, MsQol 29"2. 111 patients used E, 109 G. MHD with E at baseline, 3, 6 months: 20"94, 17"28, 10"93; with G: 20"19, 16"28, 10"83. Rest of measures will be presented and are very similar in treated with E or G, achieving the best improvements in MsQol and MOH rates. Adverse events: constipation the most reported, near 25% in both E and G treated patients.

Conclusion: Erenumab and galcanezumab are effective when used as preventive treatments in a very difficult-to-treat migraine population. Results are even better when measured in terms of Quality of Life. Constipation is the most usual adverse event, greater in real life than in clinical trials. The results of both are very similar in terms of efficacy and safety.

## AL045 Long term (48-weeks) effectiveness, safety and tolerability of erenumab in the prevention of high-frequency episodic and chronic migraine in real-world: the EARLY 2 study

### C. Aurilia^1^, S. Cevoli^2^, G. Egeo^1^, L. Fofi^1^, R. Messina^3^, A. Salerno^4^, P. Torelli^5^, M. Albanese^6^, A. Carnevale^7^, F. Bono^8^, D. D'Amico^9^, M. Filippi^3^, C. Altamura^10^, F. Vernieri^10^, B. Colombo^3^, F. Frediani^11^, B. Mercuri^4^, F. D'Onofrio^12^, L. Grazzi^9^, M. Aguggia^13^, V. Favoni^2^, C. Finocchi^14^, P. Di Fiore^11^, C. M. Costa^10^, N. Brunelli^10^, A. Fallacara^10^, D. Bertuzzo^13^, M. Zucco^15^, L. Di Clemente^15^, M. Trimboli^16^, A. Pascarella^8^, L. Manzo^8^, P. Barbanti^1^

#### ^1^IRCCS San Raffaele, Headache and Pain Unit, Rome, Italy; ^2^IRCCS Istituto delle Scienze Neurologiche, Bologna, Italy; ^3^IRCCS San Raffaele Scientific Institute, Vita-Salute San Raffaele University, Neurology Unit, Milan, Italy; ^4^San Giovanni Addolorata Hospital, Rome, Italy; ^5^University of Parma, Department of Medicine and Surgery, Headache Center, Unit of Neurology, Parma, Italy; ^6^University Hospital of Rome "Tor Vergata",, Neurophysiopathology, Rome, Italy; ^7^San Filippo Neri Hospital, Rome, Italy; ^8^A.O.U. Mater Domini, Center for Headache and Intracranial Pressure Disorders, Neurology Unit, Catanzaro, Italy; ^9^Neurological Institute C. Besta IRCCS Foundation, Headache and Neuroalgology Unit, Milan, Italy; ^10^Headache and Neurosonology Unit, Policlinico Universitario Campus Bio- Medico, Rome, Italy; ^11^Headache Center, ASST Santi Paolo Carlo, Milan, Italy; ^12^AOSG Moscati, Avellino, Italy; ^13^Cardinal Massaia Hospital, Asti, Italy; ^14^San Martino Hospital, Genova, Italy; ^15^San Camillo Hospital, Rome, Italy; ^16^San Carlo Hospital, Potenza, Italy

##### **Correspondence:** C. Aurilia

Objective: To assess the long-term effectiveness, safety and tolerability of erenumab in real-world.

Methods: In 48-week, multicenter (n=15) longitudinal cohort real life study, all consecutive adult patients with high-frequency episodic migraine (HFEM) or chronic migraine (CM) received erenumab 70 mg monthly. Change in monthly migraine days (MMD) at weeks 45-48 compared to baseline was the primary efficacy endpoint. Secondary endpoints encompassed change in monthly analgesic intake (MAI), ≥50%, ≥75%, or 100% response rates, VAS and HIT-6 scores. Results: Of the 242 patients treated with >1 dose, 221 received erenumab for >48 weeks. Patients had >3 prior preventive treatments failures. Most subjects received 140 mg. From baseline to weeks 45-48, erenumab reduced MMD by 4.3 days in HFEM and 12.8 in CM. VAS and HIT-6 were decreased by 1.8 and 12.3 in HFEM, and 3.0 and 13.1 in CM. MAI passed from 11 to 5 in HFEM and from 20 to 6 in CM.>50% responders were 56.1% in HFEM and 75.6% in CM, >75% were 31.6% and 44.5%, and 100% responders 8.8% and 1.2% respectively. Erenumab was safe. Responsiveness predictors were allodynia (p=0.009) in HFEM and male gender (p=0.044) and baseline migraine frequency (p=0.001) in CM. Negative predictors in CM were psychiatric comorbidities (p=0.023) and prior treatment failures (p=0.004).

Conclusions: Long-term erenumab treatment provides sustained effectiveness, safety and tolerability in HFEM or CM patients with >3 prior preventive treatment failures.

## AL046 Chronic Migraine with Medication Overuse Headache: is detoxification still necessary in the era of new prophylaxes?

### D. Mascarella, E. Matteo, G. M. Asioli, U. Pensato, V. Favoni, G. Andrini, G. Pierangeli, S. Cevoli, P. Cortelli

#### University of Bologna, Bologna, Italy

##### **Correspondence:** D. Mascarella

Objectives: To estimate the effectiveness of detoxification in multi-resistant chronic migraine (CM) with medication overuse headache (MOH) patients who start prophylaxes with Botulinum Toxin A(BTA) or Anti-CGRP mAb.

Method: Prospective analysis of all CM with MOH patients with at least 28 monthly headache days (MHD) who started a prophylaxis with either BTA or Anti-CGRP, with or without detoxification at the beginning of treatment, at Bologna Headache Center. We evaluated CM remission and MHD at three months.

Results: 89 patients were included; 50 started BTA and 39 Anti-CGRP.

In the BTA group, 29 patients started prophylaxis immediately after detoxification (PAD) and 21 prophylaxis alone (PA). At 3-months, we observed conversion to episodic migraine in two (9.5%) of the 21 PA patients and in six (20.6%) of the 29 PAD patients (p 0.28). Mean MHD at three months were 26.9 in the PA group versus 22.7 in the PAD group (p 0.03)

In the Anti-CGRP group, 13 patients started PAD and 26 PA. At 3-months we observed clinical conversion to episodic migraine in five of the 26 PA patients (19.2%) and five of the 13 PAD (38.4%) (p 0.1). Mean MHD were 22.2 in the PA group and 16.3 in the PAD group (p 0.11)

Conclusion: Detoxification seems to maintain a key role in preventive treatment of CM patients with MOH, also in the era of new prophylaxes. Larger samples are warranted to obtain definitive results.

**Fig. 1 (abstract AL046).**
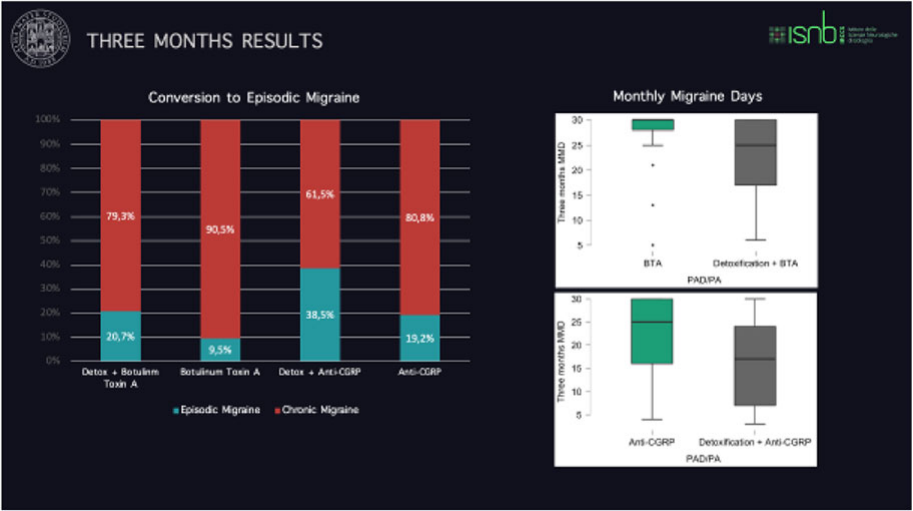
See text for description

## AL047 Effectiveness of prophylactic and acute migraine treatment in rare migraine syndromes

### I. de Boer, Y. Su, A. Wilms, J. Simons, N. Pelzer, G. M. Terwindt

#### Leiden University Medical Center, Neurology, Leiden, Netherlands

##### **Correspondence:** A. Wilms

Objective: Rare migraine syndromes have provided insight into migraine mechanisms. Little is known on treating migraine in these disorders. Physicians seem reluctant to start triptans fearing (vascular) side-effects. We evaluated migraine treatment in Hemiplegic Migraine (HM), Cerebral Autosomal Dominant Arteriopathy with Subcortical Infarcts and Leukoencephalopathy (CADASIL) and Retinal Vasculopathy with Cerebral Leukoencephalopathy and Systemic manifestations (RVCL-S).

Methods: A retrospective cohort study was performed in HM, CADASIL and RVCL-S patients diagnosed between 2009-2020. Treatment effectiveness and side-effects were assessed.

Results: We included 77 HM (median follow up years (FUY):1 IQR 1-5), 114 CADASIL (FUY:1 IQR 0-3) and 28 RVCL-S (FUY:7 IQR 4-11) patients. Migraine prevalence was 53% in CADASIL and 43% in RVCL-S. Prophylactics were prescribed in 53 (69%) HM, 9 (15%) CADASIL, and 3 (25%) RVCL-S patients. In 80% of HM, 90% of CADASIL, and 66% of RVCL-S patients treatment was effective. Most effective prophylactics with the least side-effects for HM were: 1) lamotrigine, 2) valproate, 3) topiramate, 4) acetazolamide. Valproate appeared most effective in CADASIL. Acetazolamide and propranolol showed efficacy in RVCL-S. Triptans were used by 86 patients without severe side-effects.

Conclusion: Lamotrigine, valproate, acetazolamide and topiramate are effective in HM. Valproate seems effective for migraine in CADASIL. No severe side-effects of triptans were reported.

## AL048 Impact of Baseline Characteristics on the Efficacy and Safety of Eptinezumab in Patients With Migraine: Subgroup Analyses of PROMISE-1 and PROMISE-2

### V. Martin^1^, A. J. Nagy^2^, M. Janelidze^3^, G. Giorgadze^4^, J. Hirman^5^, R. Cady^6^, L. Mehta^7^, D. C. Buse^8,9^

#### ^1^UC Headache and Facial Pain Center, Cincinnati, OH, United States; ^2^Nevada Headache Institute, Las Vegas, NV, United States; ^3^Tbilisi State Medical University, Tbilisi, Georgia; ^4^Aversi Clinic, Tbilisi, Georgia; ^5^Pacific Northwest Statistical Consulting, Inc., Woodinville, WA, United States; ^6^Lundbeck La Jolla Research Center, San Diego, CA, United States; ^7^Lundbeck Seattle BioPharmaceuticals, Inc. (Former Employee), Bothell, WA, United States; ^8^Albert Einstein College of Medicine, Bronx, NY, United States; ^9^Vector Psychometric Group, LLC, Chapel Hill, NC, United States

##### **Correspondence:** R. Cady

Objective: To evaluate the efficacy of eptinezumab for migraine prevention across patient subgroups defined by patient self-reported intrinsic factors.

Methods: PROMISE-1 and PROMISE-2 randomized adults with episodic and chronic migraine, respectively, to eptinezumab or placebo. Data from the studies were pooled, with clinical efficacy evaluated using the predefined ≥50% migraine responder rate (MRR) over weeks 1–12 endpoint. Intrinsic factors included select demographics, medical history, and migraine characteristics at baseline. No formal statistical testing was performed due to the post hoc nature of the analysis.

Results: Demographics and baseline characteristics were balanced across the 100-mg, 300-mg, and placebo groups. Intrinsic factors defined by migraine characteristics (age at migraine diagnosis, duration of migraine diagnosis, baseline monthly headache days, and baseline MMDs) did not impact ≥50% MRRs with eptinezumab, with a similar efficacy profile across both dose levels. A dose-response trend was noted for eptinezumab 100 mg and 300 mg for gender (male), obesity (class I, class II), and race (nonwhite), with numerically higher ≥50% MRRs for 300 mg vs 100 mg. Intrinsic factors had no impact on safety outcomes, with no new safety signals identified.

Conclusion: This exploratory analysis found that eptinezumab demonstrated a clinical response (ie, ≥50% MRR vs placebo) across a wide range of demographic factors and disease characteristics at baseline.

## AL049 Consistent efficacy and safety of erenumab over time in patients with episodic migraine who completed a 5-year, open-label extension study

### M. Ashina^1^, P. J. Goadsby^2,3^, U. Reuter^4^, S. D. Silberstein^5^, D. W. Dodick^6^, F. Zhang^7^, F. Xui^7^, S. Cheng^7^, D. E. Chou^7^, G. Paiva Da Silva Lima^7^

#### ^1^University of Copenhagen, Copenhagen, Denmark; ^2^King's College London, London, United Kingdom; ^3^University of California, Los Angeles, CA, United States; ^4^Charité University Hospital Berlin, Berlin, Germany; ^5^Thomas Jefferson University, Jefferson Headache Center, Philadelphia, PA, United States; ^6^Mayo Clinic, Scottsdale, AZ, United States; ^7^Amgen Inc, Thousand Oaks, CA, United States

##### **Correspondence:** M. Ashina

Erenumab demonstrated significant reduction in migraine frequency in short-term studies. Here, we report the long-term efficacy and safety of erenumab in episodic migraine patients who completed a 5-year open-label treatment phase (OLTP; NCT01952574).

Following a 12-week placebo-controlled, double-blind treatment period (DBTP), 383 patients continued into the OLTP, receiving erenumab 70mg every 4 weeks, and increasing to 140mg after a protocol amendment (after ~2 years in OLTP). Overall, 214 patients completed the 5-year OLTP; 138 patients had efficacy data at Week 268 (end of 5-year OLTP) and were included in this analysis.

At Week 268, the mean(SD) change from the DBTP baseline in monthly migraine days (MMD) and monthly acute migraine-specific medication (AMSM) days was −5.3(3.9) and −4.4(3.3), respectively. Other efficacy results are presented in **Table 1**. Exposure-adjusted patient incidence of adverse events (AEs) and serious AEs during OLTP was 91.6 and 2.8 per 100 subject-years, respectively; this was lower than that observed for erenumab 70mg during DBTP. One fatality occurred during the safety follow-up period when no erenumab was administered and was considered unrelated to study drug by the investigator.

Patients receiving erenumab over 5-years demonstrated consistent and sustained response. Safety was comparable to that observed in patients who received erenumab 70mg during the randomised phase of the trial.

**Table 1 (abstract AL049).**
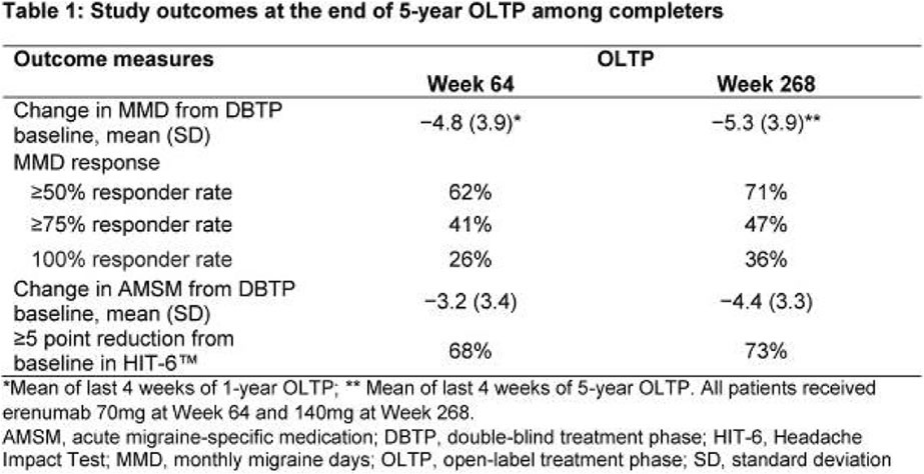
See text for description

## AL050 Real-world healthcare costs and resource utilization (HRU) among patients treated with erenumab in the United States: A retrospective claims database study

### S. J. Tepper^1^, T. J. Schwedt^2^, P. Vo^3^, P. Joshi^4^, M. Glassberg^5^, A. Abdrabboh^5^, M. Ferraris^3^, S. Tiwari^4^, J. Thompson^6^

#### ^1^Geisel School of Medicine, Dartmouth-Hitchcock Department of Neurology, Dartmouth, NH, United States; ^2^Mayo Clinic, Phoenix, AZ, United States; ^3^Novartis Pharma AG, Basel, Switzerland; ^4^Novartis Healthcare Private Limited, Value and Access, Hyderabad, India; ^5^Novartis Pharmaceuticals Corporation, East Hanover, NJ, United States; ^6^Kantar Health, New York, NY, United States

##### **Correspondence:** S. J. Tepper

Objective: To evaluate costs and HRU among migraine patients treated with erenumab in the US.

Methods: Adults with ≥3 consecutive monthly claims for erenumab (11/01/2017–09/01/2019) were identified from the Komodo Health database (index date=first erenumab claim). Mean monthly migraine-related and all-cause healthcare costs ($2019) during 180 days pre-index were compared over varied follow-up periods to assess the short- (180 days post-index), mid- (91-270 days post-index), and longer-term (maximum available follow-up time) impact of the treatment. HRU was compared over 180 days pre- vs. 180 days post-index periods. Outcomes were adjusted for patient characteristics.

Results: Overall, 1,839 patients were included (mean age 47 years; 86% females). Following erenumab initiation, a reduction in mean monthly migraine-related (P<0.0001) and all-cause medical costs (P=0.07) during the 180-day post-index period was observed, which was associated with significant increase in migraine-related (P<0.0001) and all-cause prescription costs (P<0.0001). However, with increase in follow-up time, up to 98% of the increased migraine-related and >100% of the all-cause prescription costs were offset by the reduced medical costs (Fig. 1). A significant reduction in HRU during the 180-day post-index period was observed (Table 1).

Conclusion: Erenumab treatment has an entrance cost that gets mitigated by reduced medical cost over a long-term follow-up suggesting an improved disease management.

**Fig. 1 (abstract AL050).**
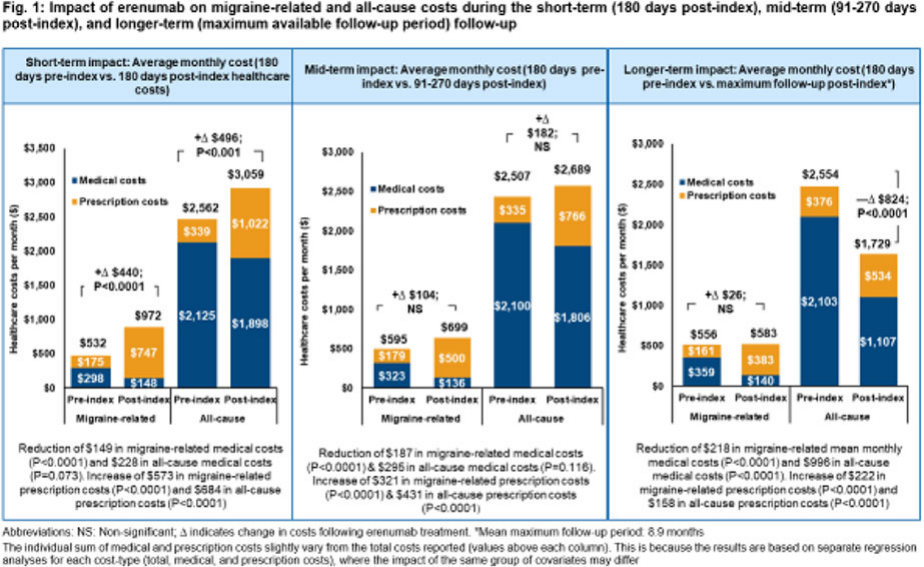
See text for description

**Table 1 (abstract AL050).**
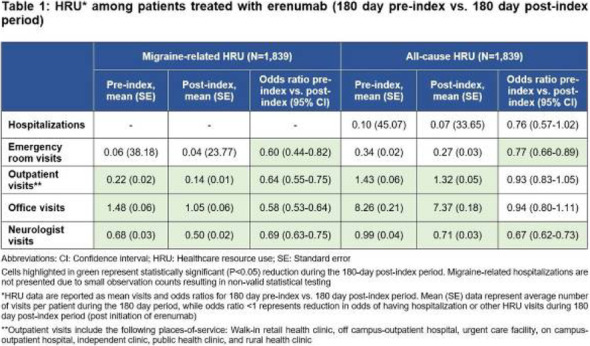
See text for description

## AL051 Efficacy of Fremanezumab in Migraine Patients With Pain or Psychiatric Comorbidities and Documented Inadequate Response to 2-4 Prior Migraine Preventive Medication Classes

### E. L. H. Spierings^1^, X. Ning^2^, J. M. Cohen^2^, V. Ramirez Campos^2^, S. Barash^2^

#### ^1^Boston Headache Institute, Boston PainCare, Waltham, MA, United States; ^2^Teva Branded Pharmaceutical Products R&D, Inc., West Chester, PA, United States

##### **Correspondence:** E. L. H. Spierings

Objective: To evaluate efficacy of fremanezumab during the 12-week double-blind (DB) period (DBP) and 12-week open-label extension (OLE) of the phase 3b FOCUS study in patients (pts) with pain or psychiatric comorbidities and inadequate response to 2-4 prior migraine preventive medication classes.

Methods: Pts were randomized to quarterly (QTY) or monthly (MLY) fremanezumab or placebo (PBO) for DBP. All pts completing DBP entered the OLE and received MLY fremanezumab. OLE outcomes are summarized by DB randomization group. Changes from baseline (BL) in monthly migraine days (MMD) and monthly headache days of at least moderate severity (MHD) were evaluated in pt subgroups with pain or psychiatric comorbidities.

Results: For pts with pain comorbidities (n=95) in the PBO, QTY, and MLY fremanezumab groups, respectively, least-squares mean (LSM) changes from BL in MMD were 0.3, −1.2, −1.5 (*P*≥0.13 vs PBO) during DBP, and mean changes were −3.1, −5.9, and −3.7 during OLE. For pts with psychiatric comorbidities (n=207) in the PBO, QTY, and MLY fremanezumab groups, LSM changes from BL in MMD were −0.9, −3.7, and −4.2 (*P*<0.001 vs PBO) during DBP, and mean changes were −4.6, −4.0, and −5.1 during OLE. Similar reductions in MHD were observed in pts with pain or psychiatric comorbidities.

Conclusion: Fremanezumab demonstrated efficacy over up to 6 months of treatment in migraine pts with pain or psychiatric comorbidities and inadequate response to 2-4 prior preventive medication classes.

## AL052 Changes in the Number of Non-headache Days and Functioning on Those Days With Fremanezumab Treatment in Patients With Migraine: A Pooled Analysis

### J. VanderPluym^1^, J. M. Cohen^2^, X. Ning^2^, V. Ramirez Campos^2^, L. Janka^2^, D. C. Buse^3^

#### ^1^Mayo Clinic, Phoenix, AZ, United States; ^2^Teva Branded Pharmaceutical Products R&D, Inc., West Chester, PA, United States; ^3^Albert Einstein College of Medicine, Bronx, NY, United States

##### **Correspondence:** J. VanderPluym

Objective: This pooled analysis assessed number of non-headache (HA) days and functioning on non-HA days from three phase 3, double-blind trials (HALO CM, HALO EM, and FOCUS).

Methods: In all 3 studies, patients (pts) were randomized 1:1:1 to quarterly (QTY) fremanezumab, monthly (MLY) fremanezumab, or placebo (PBO) over 12 weeks. A HA day was defined as a day with ≥4 consecutive hours of HA or acute migraine-specific medication use. Changes from baseline (BL) in monthly non-HA days were evaluated in the overall population; diary questions about functioning on non-HA days were evaluated in a subgroup with ≥1 non-HA day.

Results: In the overall population (N=2,823), mean change in monthly non-HA days from BL during the 12-week double-blind period (DBP) with QTY and MLY fremanezumab and PBO was 4.7, 4.9, and 2.9 days, respectively. During month 3 in the subgroup with ≥1 non-HA day across (n=2,749), few pts reported poor functioning on non-HA days across the QTY and MLY fremanezumab and PBO groups (≥50% impaired work/studying ability, <1%; feeling bad, <1%; difficulty concentrating most of the time, 3%; tired/asleep/drained most of the time, 6%; ≥50% impaired ability to perform household chores, 2%; not engaged in partner's/children's activities, 3-4%; not interested in daily activities, 2%).

Conclusion: Both QTY and MLY fremanezumab increased non-HA days versus PBO. Pt responses about daily functionality on non-HA days were comparable in the fremanezumab and PBO groups at month 3.

## AL053 Onset of Migraine Preventive Effects With Rimegepant in a Phase 2/3, Randomized, Double-Blind, Placebo-Controlled Trial

### R. B. Lipton^1^, D. Kudrow^2^, T. Smith^3^, R. Croop^4^, C. M. Jensen^4^, L. Kamen^4^, A. Thiry^4^, V. Coric^4^, P. J. Goadsby^5,6^

#### ^1^Albert Einstein College of Medicine, Bronx, NY, United States; ^2^California Medical Clinic for Headache, Santa Monica, CA, United States; ^3^StudyMetrix Research, St Louis, MO, United States; ^4^Biohaven Pharmaceuticals, New Haven, CT, United States; ^5^NIHR-Wellcome Trust King’s Clinical Research Facility, King’s College Hospital/SLaM Biomedical Research Centre, King’s College, London, Germany; ^6^University of California, Los Angeles, Neurology, Los Angeles, CA, United States

##### **Correspondence:** P. J. Goadsby

Objective: Assess the onset of migraine preventive treatment efficacy with rimegepant, an oral small molecule CGRP receptor antagonist, during each of the first 4 weeks of treatment with an every other day dosing regimen.

Methods: Multicenter, randomized, double-blind, placebo-controlled trial (NCT03732638) enrolled adults with a history of 4-18 monthly migraine attacks of moderate to severe pain intensity. After a 4-week observation period (OP), subjects were randomized to rimegepant 75 mg or placebo every other day for 12 weeks. This post-hoc analysis assessed mean changes from the OP in number of weekly migraine days during Weeks 1 through 4. *P* values are uncorrected for multiple comparisons.

Results: In total, 741 subjects were treated with study medication (rimegepant n=370, placebo n=371). Mean age was 41.2 years; 82.7% of subjects were female, and 23.3% had chronic migraine. Rimegepant and placebo-treated subjects, respectively, had 2.6 and 2.5 mean weekly migraine days at baseline. Rimegepant was more effective than placebo for mean change in weekly migraine days as early as Week 1 (−0.7 vs −0.3, *p*=0.0003); mean percentage change (95% CI) from the OP in weekly migraine days during Week 1 was −30% (−36.1, −23.9) for rimegepant and −9.4% (−17.1, −1.8) for placebo. Weekly migraine day reductions through Week 4 are shown in Table 1 and Figure 1.

Conclusions: Oral rimegepant taken every other day demonstrated migraine preventive effects within the first week of treatment.

**Table 1 (abstract AL053).**
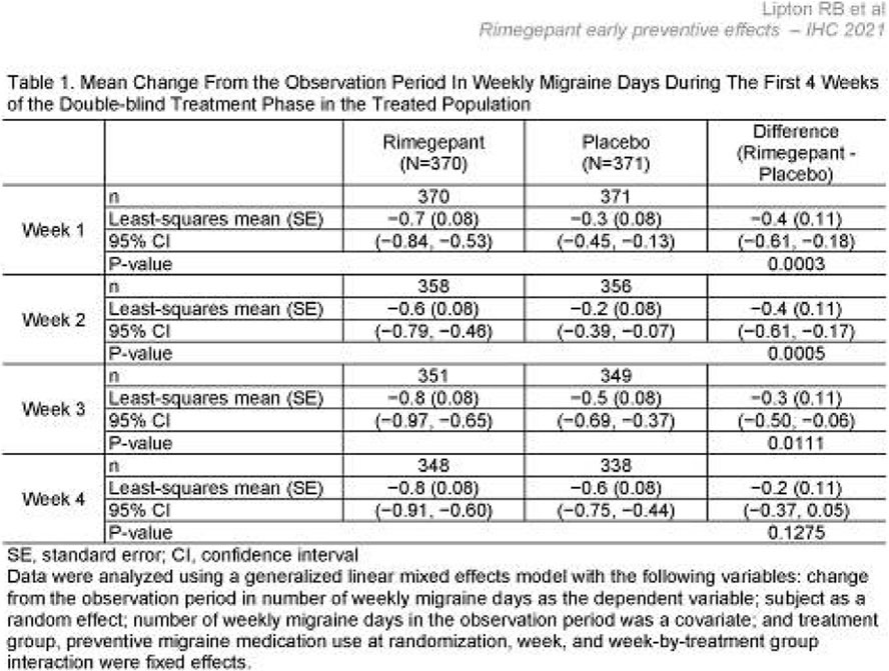
See text for description

**Fig. 2 (abstract AL053).**
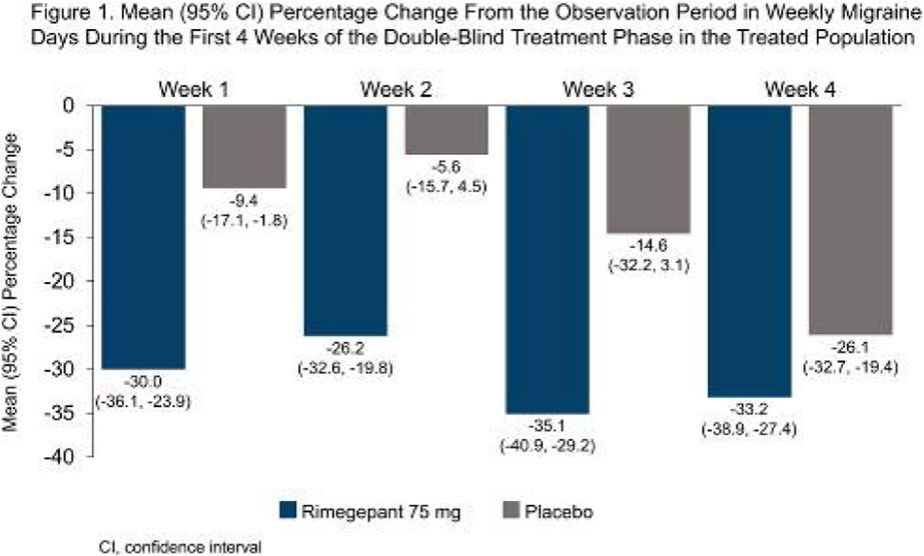
See text for description

## AL054 A Phase 2/3, Randomized, Double-blind, Placebo-controlled Study to Evaluate the Efficacy and Safety of Rimegepant for the Preventive Treatment of Migraine

### R. Croop^1^, R. B. Lipton^2^, D. Kudrow^3^, D. A. Stock^1^, L. Kamen^1^, C. M. Conway^1^, E. G. Stock^1^, V. Coric^1^, P. J. Goadsby^4,5^

#### ^1^Biohaven Pharmaceuticals, New Haven, CT, United States; ^2^Albert Einstein College of Medicine, Bronx, NY, United States; ^3^California Medical Clinic for Headache, Santa Monica, CA, United States; ^4^NIHR-Wellcome Trust King’s Clinical Research Facility, King’s College Hospital/SLaM Biomedical Research Centre, King’s College, London, Germany; ^5^University of California, Los Angeles, Neurology, Los Angeles, CA, United States

##### **Correspondence:** R. Croop

Objective: Compare the efficacy, safety, and tolerability of rimegepant — an orally administered, small molecule calcitonin gene-related peptide receptor antagonist with demonstrated efficacy in the acute treatment of migraine – with placebo for the preventive treatment of migraine.

Methods: Randomized, double-blind, placebo-controlled trial (NCT03732638) in adults with a history of 4-18 moderate-severe migraine attacks/month. After a 4-week baseline observation period, subjects were randomized to oral rimegepant 75 mg or placebo every other day for 12 weeks. The primary efficacy endpoint was change from the 4-week observation period in the mean number of migraine days per month (MMD) during Weeks 9-12.

Results: In total, 741 subjects were treated (rimegepant n=370 placebo n=371; mean age 41.2 years, 82.7% female, 81.5% white, 23.3% chronic migraine), and 695 were evaluated for efficacy (rimegepant n=348 placebo n=347). Rimegepant was superior to placebo for the primary endpoint and secondary endpoints of ≥50% reduction in the mean number of moderate or severe MMDs during Weeks 9-12 and change in the mean number of total MMDs during Weeks 1-12 (Table 1). The incidence of adverse events was similar in the rimegepant and placebo groups (35.9% vs 35.8%; Table 2).

Conclusions: Rimegepant 75 mg taken every other day was effective for the preventive treatment of migraine. Tolerability was similar to placebo, with no unexpected or serious safety issues.

**Table 1 (abstract AL054).**
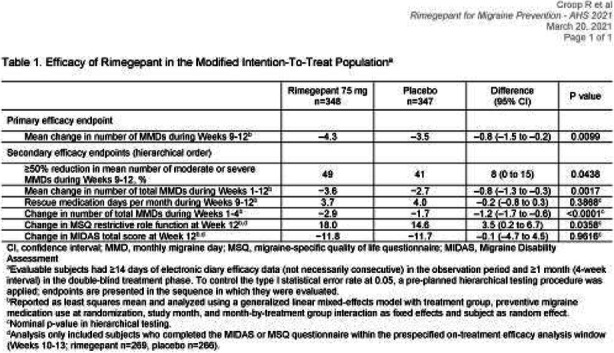
See text for description

**Table 2 (abstract AL054).**
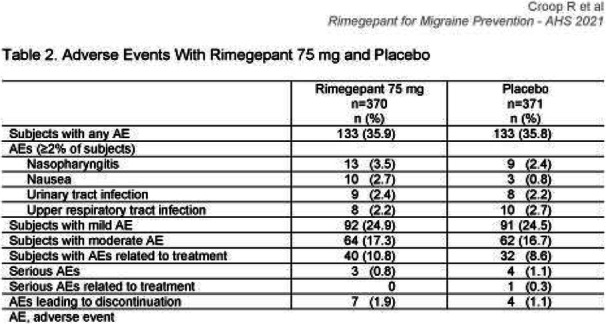
See text for description

## AL055 Efficacy and Safety of AXS-07 (MoSEIC Meloxicam-Rizatriptan) for the Acute Treatment of Migraine: Results from the MOMENTUM Phase 3, Randomized, Double-blind, Active- and Placebo-controlled Trial

### C. O'Gorman^1^, A. Jones^1^, S. J. Tepper^2^, C. Askew^1^, R. B. Lipton^3^, H. Tabuteau^1^

#### ^1^Axsome Therapeutics, New York, NY, United States; ^2^Geisel School of Medicine at Dartmouth, Hanover, NH, United States; ^3^Albert Einstein College of Medicine, New York, NY, United States

##### **Correspondence:** C. O'Gorman, A. Jones

Background and objective: The study aimed to evaluate the efficacy & safety of AXS-07, a novel, oral, multi-mechanistic investigational medicine for the acute treatment of migraine in patients with a history of inadequate response to prior therapy.

Methods: MOMENTUM was a Phase 3, double-blind, controlled study that randomized 1,594 patients (2:1:2:2) to treatment with AXS-07, placebo (Pbo), MoSEIC meloxicam (Mlx), or rizatriptan (Riz).

Results: AXS-07 resulted in a greater percentage of patients vs. Pbo achieving pain freedom (PF) (19.9% vs. 6.7%, p<0.001) and absence of most bothersome symptom (36.9% vs 24.4%, p=0.002), at 2 hours. AXS-07 demonstrated faster time to pain relief (PR) of 1.5hrs vs. Pbo (12hrs, p<0.001), Mlx & Riz (both 4hrs, p<0.001); AXS-07 PR rates were greater at 30mins & thereafter. 48-hour sustained PF and PR were achieved by a significantly greater proportion of AXS-07 patients vs. Pbo, Mlx & Riz (p<0.001 to p=0.003). AXS-07 patients achieved greater global symptom improvement (PGI-C) & rate of normal functioning at 24hrs vs. Pbo, Mlx & Riz (p<0.001 to 0.027). A significantly lower proportion of AXS-07 patients used rescue meds vs. control (all p<0.001). AXS-07 reduced pain relapse >50% vs. Riz (p=0.001). AXS-07 was safe & well-tolerated.

Conclusions: AXS-07 produced rapid, substantial, and sustained efficacy vs. Pbo, Mlx & Riz in the acute treatment of migraine in patients with a history of inadequate response.

## AL057 Withdrawn

## AL058 Erenumab versus topiramate for the prevention of migraine: Results of a post-hoc efficacy analysis

### M. Ehrlich^1^, M. Maier-Peuschel^1^, C. Sieder^1^, C. Hentschke^1^, U. Reuter^2^

#### ^1^Novartis Pharma GmbH, Nürnberg, Germany; ^2^Charité University Hospital Berlin, Berlin, Germany

##### **Correspondence:** U. Reuter

Objective: In this post-hoc analysis of the HER-MES trial, we compared the efficacy of erenumab vs. topiramate using multiple imputation.

Background: HER-MES is the first study to compare a CGRP-AB to one of the most commonly used migraine prophylactic drugs in a randomized, controlled trial.

Design/Methods: HER-MES is the first head-to-head double-blind, double-dummy trial comparing the tolerability and efficacy of erenumab to topiramate in a German cohort ofpatients with at least 4 monthly migraine days (MMD). HER-MES comprised a 24-week treatment epoch (DBTE) in which patients received (1) either 70 mg or 140 mg subcutaneous erenumab/ oral placebo or (2) s.c. placebo/maximally tolerated dose of topiramate (50-100 mg/daily). This post-hoc analysis compares the efficacy of erenumab and topiramate over months 4, 5, and 6 regarding the 50 % responder rate (RR) and change in monthly migraine days from baseline using a multiple imputation model.

Results: For both outcomes, 50 % RR and change in MMD from baseline in month 4, 5, and 6 erenumab proved to be superior to topiramate.

Conclusion: This analysis displays a hypothetical scenario in which all patients stayed on drug throughout the 24-weeks treatment phase, despite AE and ineffective response. The results of this post-hoc analysis complement the efficacy results from the HERMES primary analysis and further support the benefits of erenumab over topiramate in the prevention of migraine.

## AL059 A Phase 3 randomized, double-blind, sham-controlled Trial of e-TNS for the Acute treatment of Migraine (TEAM)

### D. Kuruvilla^1^, A. Starling^2^, S. J. Tepper^3^, J. Mann^4^, G. Panza^5^, M. Johnson^5^

#### ^1^Westport Headache Institute, Neurology, Headache, Westport, CT, United States; ^2^Mayo Clinic, Neurology, Phoenix, AZ, United States; ^3^Dartmouth-Hitchcock Medical Center, Neurology, Lebanon, NH, United States; ^4^Rochester Clinical Research, Rochester, MN, United States; ^5^Cefaly, Seraing, Belgium

##### **Correspondence:** D. Kuruvilla

Background: The CEFALY device is a non-invasive neuromodulation treatment which stimulates the bilateral supraorbital nerves transcutaneously to provide pain relief by targeting the trigeminal nerve.

Methods: We conducted a randomized, double blind, sham-controlled, multicenter study at 10 sites in the United States. 538 adults diagnosed with 2-8 migraine headache days per month were randomized to active or sham stimulation. Neurostimulation was applied for a 2-hour, continuous session. Migraine pain levels and most bothersome migraine-associated symptom (MBS) were recorded at baseline, 2 hours and 24 hours using a paper diary. The primary endpoints for the study were pain freedom at 2 hours and freedom from the MBS at 2 hours. The secondary endpoints were pain relief at 2 hours, absence of all most bothersome migraine-associated symptoms (MBSs) at 2 hours, acute medication use within 24 hours after treatment, sustained pain freedom at 24 hours and sustained pain relief at 24 hours.

Results: Active stimulation was more effective than sham stimulation in achieving pain freedom at 2 hours with a therapeutic gain of 7.2% (25.5% versus 18.3%, p=0.043). MBS freedom at 2 hours was also higher in the activegroup compared to the sham group (56.4% versus 42.3%, p= 0.001).

Conclusion: External trigeminal nerve stimulation with the CEFALY device was found to be superior to a sham device in providing pain freedom and freedom from the MBS at 2 hours

## AL060 Ubrogepant users' experience - Patients on Ubrogepant, characteristics and outcomes (UNIVERSE STUDY)

### A. R. Shewale^1^, W. Poh^2^, M. L. Reed^3^, S. Manthena^1^, F. Cadiou^2^, K. Burslem^1^, R. B. Lipton^4^

#### ^1^AbbVie, North Chicago, IL, United States; ^2^Healint Pte. Ltd, Singapore, Singapore; ^3^Vedanta Research, Chapel Hill, NC, United States; ^4^Albert Einstein College of Medicine, Bronx, NY, United States

##### **Correspondence:** A. R. Shewale

Objective: To examine the real-world effectiveness of ubrogepant for acute treatment of migraine using the Migraine Buddy application.

Methods: This is a observational cross-sectional study of US adult Migraine Buddy users who have self-reported using at least 4 doses of ubrogepant. Participants indicating at least one dose in the preceding 14 days completed a 29-question self-reported survey that assessed patient characteristics, treatment patterns, and satisfaction with ubrogepant.

Results: Results are based on planned interim analysis of 84 participants (mean age: 43.2 years, 87% female) out of a planned sample of 300 participants. Majority reported being satisfied with ubrogepant for pain relief at 2-hrs (67.9%), at 4-hrs (84.5%) and at 24-hrs (81.0%) post-dose (Fig:1). Participants reported very high satisfaction for ability to think clearly (84.5%), return to normal function (86.9%), and 90.5% reported they were likely to continue using ubrogepant. Analyses of prior and current acute medication use with ubrogepant suggest reductions in opioids (-80%), barbiturates (-58%), ergots (-93%), triptans (-65%), NSAIDs (-47%), and other acute medications (-32%) use.

Conclusions: Ubrogepant users reported high satisfaction with pain relief, ability to think clearly, return to normal function and most indicated that they were likely to continue its use. Ubrogepant use was also associated with reductions in opioid and barbiturate use suggesting additional clinical benefits for users.

**Fig. 1 (abstract AL060).**
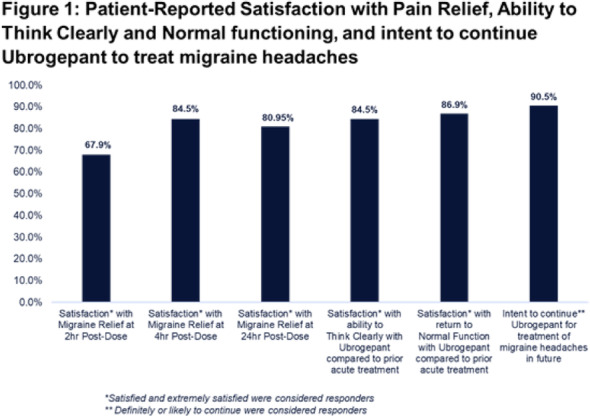
See text for description

## P01 Clinical profile of chronic cluster headache (CCH) in a regional headache center in Japan

### S. Kikui^1^, J. Miyahara^1^, H. Sugiyama^1^, M. Kohashi^1^, K. Ota^1^, D. Danno^1^, Y. Kashiwaya^1^, T. Takeshima^1^

#### ^1^Tominaga Hospital, Department of Neurology, Headache Center, Osaka, Japan

##### **Correspondence:** S. Kikui

Background: CCH is a refractory headache that lowers quality of life, but little is known about the characteristics of CCH in Japan.

Object & Methods: 19 consecutive patients with CCH visiting at a tertiary headache center (Tominaga hospital) from February 2011 to July 2020. Patients with CCH were interviewed using standardised questionnaires during a consultation.

Results: Patients with CCH accounted for 4.2% (19/420) of CH. The demographic characteristics of the study participants are shown in Table 1. Patients with CCH in Japan had later age of CH onset. Nine (47.4%) patients had CCH from onset of CH (primary CCH), and in the remaining 10 (52.6%) patients, CCH had evolved from episodic CH (secondary CCH). There were more smokers in the secondary CCH group. In one primary CCH patient, CH attacks had disappeared. Two secondary CCH patients migrated to episodic CH. Seven patients have persistent CCH. The detailed treatment results are provided in Tables 2. In 6 cases, quality of life has been improved by the combined use of HOT and subcutaneous sumatriptan injection.

Conclusions: prevalence of CCH in our study is low as in other Asian regions. Patients with CCH in Japan had later age of CH onset of and the duration of evolution in patients with secondary CCH is a long interval after CH onset. There are many cases of CCH that can be controlled with HOT and sumatriptan subcutaneous injection. Drug control may be better in many CCH cases in Japan.

**Table 1 (abstract P01).**
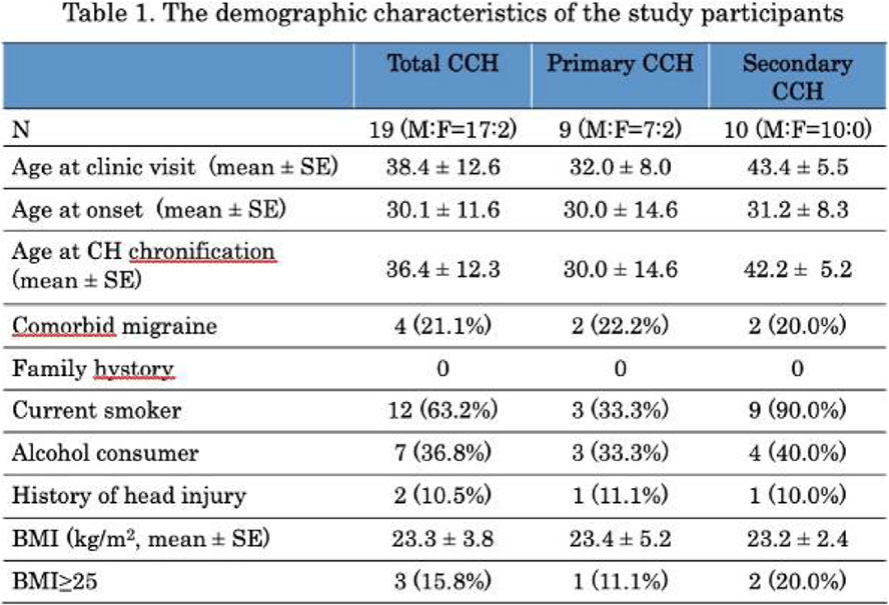
See text for description

**Table 2 (abstract P01).**
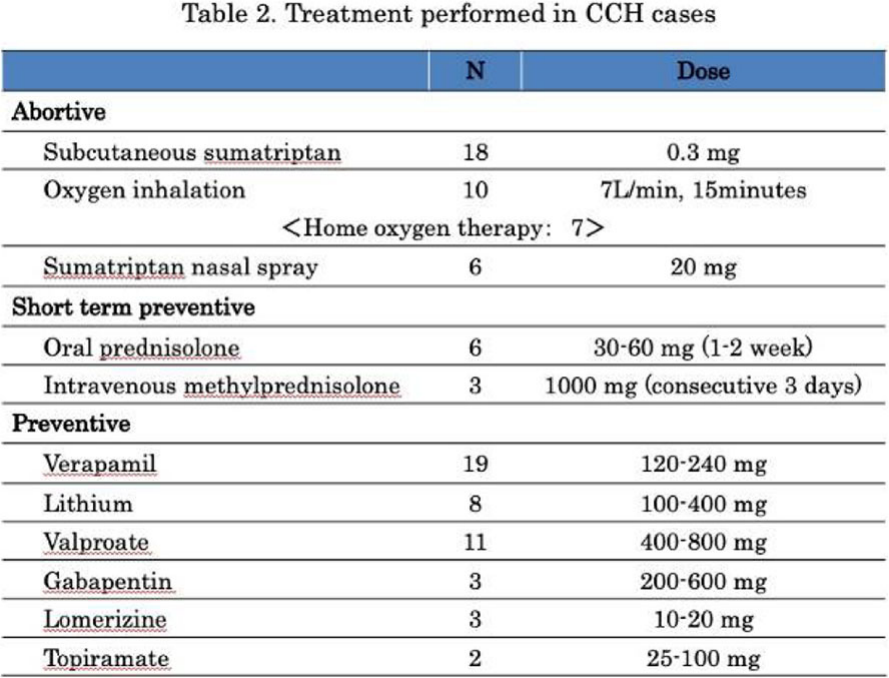
See text for description

## P02 Clinical characteristics and burden of a large series with cluster headache from Turkey

### P. Yalınay Dikmen^1^, C. ARI^2^, E. Sahin^3^, M. Ertaş^3^, F. Mayda Domac^4^, E. Ilgaz Aydinlar^1^, A. Sahin^1^, A. Özge^5^, H. OZGUNER^5^, O. Karadas^6^, J. Shafiyev^6^, D. Vuralli^7^, E. OGUZ AKARSU^8^, N. Karli^8^, M. Zarifoglu^8^, H. Bolay^9^, E. Ekizoglu^3^, E. Kocasoy Orhan^3^, B. Baykan^3^

#### ^1^Acibadem University School of Medicine, Neurology, Istanbul, Turkey; ^2^Siirt State Hospital, Neurology, Siirt, Turkey; ^3^Istanbul University, school of Medicine, Neurology, Istanbul, Turkey; ^4^University of Health Sciences, Erenköy Training and Research Hospital for Psychiatric and Neurological Disorders, Neurology, Istanbul, Turkey; ^5^Mersin University School of Medicine, Neurology, Mersin, Turkey; ^6^University of Health Science, Gulhane School of Medicine, Neurology, Ankara, Turkey; ^7^Gazi University Faculty of Medicine, Algology and Neurology, Ankara, Turkey; ^8^Uludag University, Neurology, Bursa, Turkey; ^9^Gazi University Faculty of Medicine, Neuropsychiatry Center, Ankara, Turkey

##### **Correspondence:** P. Yalınay Dikmen

Objective: To present results from a cluster headache (CH) survey from Turkey regarding clinical characteristics, diagnostic delay, triggers, treatment and personal burden.

Methods: The survey was composed of 76 questions. Participants diagnosed with CH according to IHS criteria were recruited from headache centers.

Results: A total of 209 individuals with a mean age of 39.8 (11.3) completed the survey (176 males; 188 episodic, 21 chronic). The mean age at onset was 28.6 (10.2). Diagnostic delay was 4.9 years. Incorrect diagnosis before CH was 57.9%. Of participants, 9.1% reported a positive family history for CH and 54.5% had a history of current/prior tobacco exposure. Strikingly, 26.8% noted an aura before a CH attack. The most common cranial autonomic symptoms were lacrimation in 79.9 %, followed by nasal congestion 55%, agitation 55 %, eyelid swelling 50.2 %. Of episodic CH patients, 72.7% had ≥ 1 bout per year. The mean duration of CH attack with and without medication was 40.9 (31.6) and 91.8 (58) minutes, respectively. A positive response to high-flow oxygen was observed in 67% of the participants. The most commonly used prophylactic agent was verapamil (72.7%). In this study, 48% of CH patients reported significant personal burden (episodic 47.3% vs. chronic 62.5%; p=0.80).

Conclusion: Diagnostic delay and incorrect diagnosis were still frequent before a proper diagnosis. The significant burden was reported by patients regardless of chronicity.

## P03 Sphenopalatine Ganglion Volume in Cluster Headache: From Symptoms Laterality toward Treatment Prediction

### J. W. Wu^1,2^, S. T. Chen^1^, Y. F. Wang^1,2^, K. L. Lai^1,2^, S. J. Wang^1,2^

#### ^1^Taipei Veterans General Hospital, Taipei City, Taiwan; ^2^National Yang Ming Chiao Tung University, Taipei City, Taiwan

##### **Correspondence:** J. W. Wu

Objectives: This study aimed to identify the role of sphenopalatine ganglion (SPG) in symptoms laterality and treatment response in patients with cluster headache (CH).

Methods: We prospectively recruited patients with side-locked episodic CH from our clinic and collected their medical records, headache questionnaires, and data of treatment response. All patients received brain MRI including specialized protocol focusing on SPG during the in-bout period. We compared the SPG volume between pain and non-pain sides and analyzed the association between SPG volume and acute treatment response to sumatriptan nasal spray (NS).

Results: In this study, 34 in-bout CH patients underwent brain MRI. The SPG volume (mean ± SD) was larger at the pain side (pain side vs. non-pain side:36.0±15.5 vs. 30.0±13.2μL, p=0.011). Responders to sumatriptan NS tended to have a larger SPG volume at the pain side (responder vs. non-responder: 43.4±18.3 vs. 31.9±12.4μL, p=0.037). Patients with SPG volume ≥40μL have a higher response rate to sumatriptan NS (≥40 vs. <40μL: 61.5% vs. 19.0%, p=0.012).

Conclusion: Using the specialized protocol for measurement of SPG volume, our study showed the CH patients had a larger SPG over the ipsilateral side. In addition, larger SPG volume predicted a higher response rate to sumatriptan NS, suggesting the potential "direct" effect of sumatriptan on the SPG. The SPG volumetry provides insights for future research in the pathophysiology and treatment of cluster headache.

**Fig. 1 (abstract P03).**
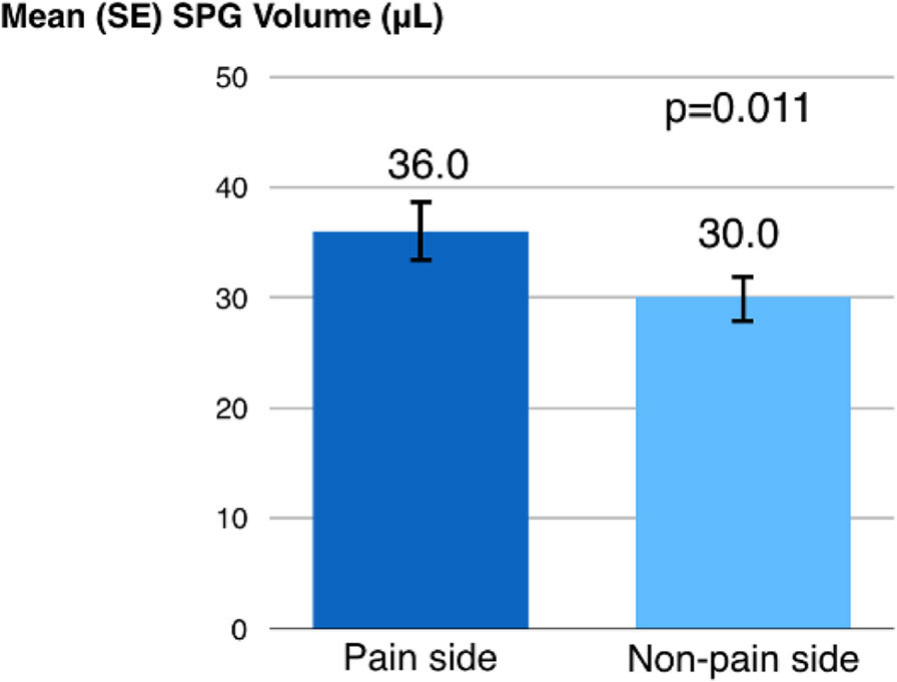
See text for description

**Fig. 2 (abstract P03).**
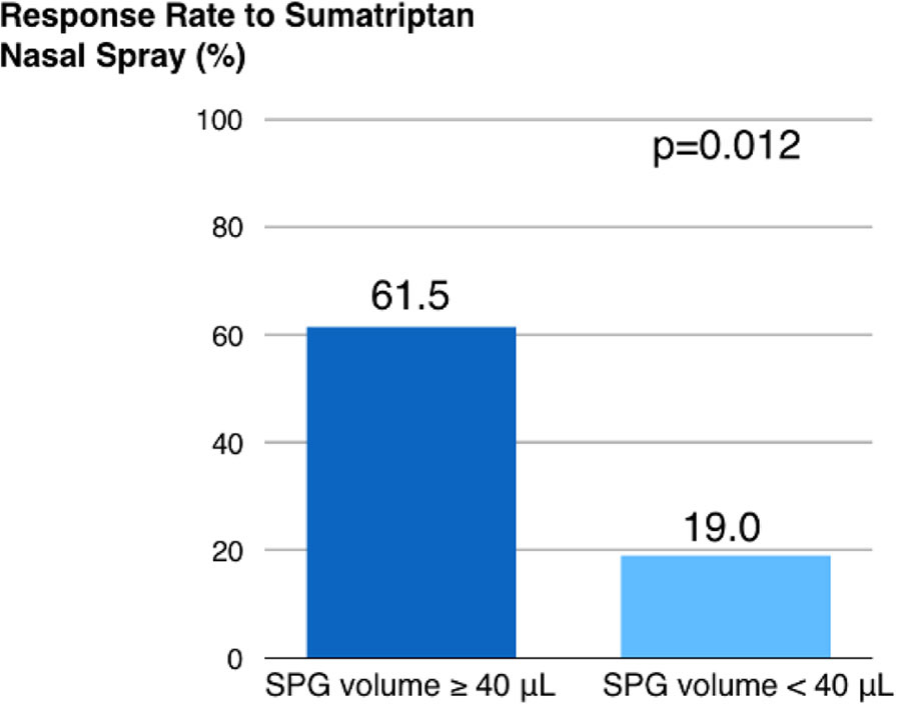
See text for description

## P04 Effect of Caffeine and Caffeine Cessation on Cerebrovascular Reactivity in Patients with Migraine

### Y. E. Gil^1^, S. Cho^2^, C. S. Chung^1^, M. J. Lee^1^

#### ^1^Samsung Medical Center, Sungkyunkwan University School of Medicine, Neurology, Seoul, South Korea; ^2^Uijeongbu Eulji Medical Center, Neurology, Uijeongbu, South Korea

##### **Correspondence:** Y. E. Gil

Background and objective: Studies regarding cerebrovascular reactivity (CVR) using vasodilatory stimuli in patients with migraine have yielded conflicting results. We aimed to investigate the effect of chronic caffeine use and caffeine cessation on CVR in patients with migraine.

Methods: We prospectively recruited patients with episodic migraine who were 18 – 50 years of age and free of vascular risk factors at the Samsung Medical Center. Patients were classified into caffeine users and non-users at baseline, and caffeine users were instructed to discontinue caffeine intake. We measured transcranial Doppler (TCD) breath-holding index (BHI) in all the included patients at baseline and followed up after 3 months. We compared BHIs in cerebral arteries between caffeine users and non-users, and analyzed BHI changes according to the caffeine cessation.

Results: Among 84 patients completed the study protocol, the baseline PCA-BHI was lower in caffeine users (n=56) than that in non-users (n=28, p=0.030). In the longitudinal analysis, caffeine quitters showed a significant improvement in the PCA-BHI (p=0.034), whereas continuous users and non-users did not. Multivariable analysis showed an independent effect of caffeine cessation on the changes in the PCA-BHI (unstandardized beta=0.294, 95% CI 0.047–0.541, p=0.020).

Conclusions: In patients with migraine, caffeine use is associated with reduced CVR in the PCA, and caffeine cessation might be beneficial in improving the CVR.

## P05 Patient-physician interactive data sharing enhances patient engagement in digital migraine applications

### T. Kelderman^1^, V. Keereman^2,3^, M. Vandenheede^4^, O. Langa^5^, P. Dedeken^6^, J. Schreiber^5^, J. Versijpt^7^

#### ^1^Sint-Augustinus Hospital, Wilrijk, Belgium; ^2^AZ Maria Middelares, Ghent, Belgium; ^3^MEDISIP, Ghent University, Ghent, Belgium; ^4^Clinique CHC MontLégia, Liège, Belgium; ^5^Neuroventis, Brussels, Belgium; ^6^Heilig Hart Lier Hospitaal, Lier, Belgium; ^7^UZ Brussel, Brussels, Belgium

##### **Correspondence:** T. Kelderman

Objectives: Digital health applications have the potential to improve patient empowerment and quality of care, though retention is challenging. Six-month retention rates of good-in-class solutions range between 30 and 40%. We evaluated the impact of interactive data sharing in the MigraineManager® solution (Neuroventis, Belgium) between patients connected with their physician compared to patients who did not connect.

Methods: Belgian patients with headache or migraine having signed up to the app between 01-Jan-2019 and 31-Aug-2020 were enrolled. We compared retention rates at three and 6 months and reported headache days per 100 active app days per patient (unconnected versus connected).

Results: 1007 patients were enrolled. Three and 6 month-retention rates for standalone headache app users were 34% and 20% compared to 67% and 50% for connected patients, respectively. Up to 90% of connected patients reported at least 1 headache episode, compared to 75% unconnected patients. Side effects were reported by 10% of connected users, 3.3 times more frequent than unconnected ones. The average number of reported headaches per 100 active app days was 21 for connected and 7 for standalone users.

Conclusion: Interactive data sharing in MigraineManager® increased retention rates and exceeded observations of good-in-class solutions. Future research needs to address how this patient-physician connection impacts outcomes or reflects differences in demographics or headache characteristics.

## P06 Body Mass Index and Migraine Characteristics and Comorbidities in a Large Academic Headache Center

### D. Krashin^1^, M. Dyess^2^, M. Chan-Goh^2^, M. Bigal^2^, A. Cuneo^2^, S. Belaskova^3^, N. Murinova^2^

#### ^1^Puget Sound VA, Pain and Psychiatry, Seattle, WA, United States; ^2^University of Washington, Neurology, Seattle, WA, United States; ^3^Masaryk University, Institute of Mathematics and Statistics, Brno, Czech Republic

##### **Correspondence:** N. Murinova

Background: Obese individuals have an increased risk of migraine chronification and headache comorbidities. This study aims to quantify the Body Mass Index (BMI) of new university headache patients and analyze if high BMI is associated with increased migraine chronification and other common comorbidities.

Methods: Patients referred to our clinic complete a detailed intake questionnaire prior to their first visit. This questionnaire asks about headache characteristics, sleep, depression, anxiety, and stress. All patient data are analyzed by headache providers, BMI and ICHD-3 headache diagnosis are added.

Results: Our study shows 3611 unique patients were diagnosed with migraine. Statistical analysis shows that BMI is higher in chronic migraine. Patient with BMI ≥ 30 have more headache days per month and greater headache severityhigher perception of stress scores (P<0.0001) that correlates with higher anxiety, have higher PHQ4 (P<0.0001) that correlates with depression than patients with a normal BMI. Patients reporting sleep problems have higher BMI than patients not reporting sleep problems.

Conclusion: Data suggest that BMI ≥ 30 correlates with increased migraine chronification, headache days per month, and headache severity. BMI ≥ 30 also significantly correlates with measures of migraine comorbidities, such as anxiety, depression, and difficulty with sleep. Normalizing BMI may be protective against migraine chronification and improve all migraine comorbidities.

## P07 Increased visual sensitivity in cluster headache, as quantified by the Leiden Visual Sensitivity Scale (L-VISS): a cross-sectional study

### R. Brandt^1^, V. Cnossen^1^, P. Doesborg^1^, I. de Coo^1^, T. Perenboom^1^, J. A. Carpay^2^, R. Meilof^1^, M. Ferrari^1^, G. M. Terwindt^1^, R. Fronczek^1^

#### ^1^Leiden University Medical Centre, Neurology, Leiden, Netherlands; ^2^Tergooi Hospital, Department of Neurology, Hilversum, Netherlands

##### **Correspondence:** R. Brandt

Background and objective: Abnormal sensitivity to light and patterns is typically associated with migraine. Increased visual sensitivity has also been reported in cluster headache, contributing to confusion with migraine, sometimes delaying the diagnosis. We aim to asses visual sensitivity in episodic (ECH) and chronic cluster headache (CCH).

Methods: Participants filled out the Leiden Visual Sensitivity Scale (L-VISS), assessing their visual sensitivity during an attack, between attacks, and - in ECH - outside a bout. Data were analyzed using a linear mixed model and one-way ANOVA with sex and age included as covariates.

Results: Higher L-VISS scores were observed: (i) in all CH patients *during* attacks vs *between* attacks (ECH: 11.9 vs. 5.2, CCH: 13.7 vs 5.6; *p* = .000); (ii) in ECH patients between attacks *inside* a bout vs *outside* a bout (5.2 vs 3.7, *p* = .000); (iii) in all CH patients *during* and *between* attacks vs healthy controls (12.6 vs 5.3 vs 3.6, *p* = 0.000). Subjective visual hypersensitivity was reported by 110/121 (91%) cluster headache patients; in 70/110 (64%) patients (mostly) unilateral and, in all but one case, ipsilateral to the pain.

Conclusions: Patients with CH have an increased visual sensitivity during and between attacks that is in almost two third of the cases ipsilateral to the pain. This is an important clinical realization that might contribute to a reduced diagnostic delay.

## P08 Insights into real-world treatment of Cluster Headache through a large Italian database: prevalence, prescription patterns and costs

### V. Favoni^1^, P. Carlo^2^, G. Ronconi^2^, L. Dondi^2^, S. Calabria^2^, A. Pedrini^2^, A. P. Maggioni^2^, I. Esposito^3^, A. Addesi^3^, G. Pierangeli^1,4^, P. Cortelli^1,4^, S. Cevoli^1^, N. Martini^2^

#### ^1^IRCCS delle Scienze Neurologiche di Bologna, Bologna, Italy; ^2^Fondazione ReS (Ricerca e Salute) – Research and Health Foundation, Casalecchio di Reno (Bologna), Italy; ^3^Drugs and Health, Rome, Italy; ^4^University of Bologna, Department of Biomedical and Neuromotor Sciences, Bologna, Italy

##### **Correspondence:** V. Favoni

Objective: To estimate the prevalence of treated cluster headache (CH), to describe prescription patterns and direct costs paid by the National Health System (NHS).

Methods: Cross-sectional and longitudinal analyses were performed by using the ReS database collecting healthcare administrative data of a large sample of Italian population. Adult patients with an acute treatment for CH (sumatriptan subcutaneous or oxygen) associated with a preventive therapy (verapamil or lithium) were selected. Cross-sectional analysis was used to describe the 2013-2017 annual prevalence. Longitudinal analysis of patients selected in 2013-2015 and followed for 2 years was performed to provide a picture of prescription patterns.

Results: The annual prevalence of treated CH increased from 6.4 per 100,000 adults in 2013 to 6.7 in 2017. In 2013-2015, we found 570 treated CH (80.7% M; mean age 46) out of >7 million subjects. The identifying treatment was sumatriptan subcutaneous+verapamil in the 50.4% of cases. During follow-up, >1/3 modified the preventive drug and mainly stopped it, although acute treatments were still prescribed. The mean annual cost paid by the NHS per patient ranged from €2,956 to €2,267; drugs expenditures represent the 56.4% and 57.3%, respectively.

Conclusions: We found an important unmet need among CH patients with an important economic impact. This becomes crucial in view of the Calcitonin-Gene-Related-Peptide antibodies incoming that could modify the approach to CH.

## P09 Sleep Disorders in Pediatric Migraine: a questionnaire-based study

### A. Voci^1^, M. A. N. Ferilli^2^, L. Papetti^2^, F. Ursitti^2^, G. Sforza^2^, S. Tarantino^2^, F. Vigevano^2^, M. Valeriani^2,3^, R. Moavero^1,2^

#### ^1^Child Neurology and Psychiatry Unit, Tor Vergata University of Rome, Rome, Italy; ^2^Headache Center, Child Neurology Unit, Neuroscience Department, Bambino Gesù Children’s Hospital, IRCCS, Rome, Italy; ^3^Center for Sensory Motor Interaction, Aalborg University, Aalborg, Denmark

##### **Correspondence:** A. Voci

This study aimed to analyze the relationship between headache and sleep in pediatric migraine. We evaluated differences in migraine frequency and intensity, presence of migraine equivalents, use of attack and prophylactic medications in subjects with and without sleep disorders based on the results of standardized sleep assessment questionnaires. The parents of 140 children and adolescents with migraine completed the Children's Sleep Habits Questionnaire (CSHQ) and the Epworth Sleepiness Scale for Children and Adolescents (ESS-CHAD) and answered questions about headache characteristics in their children. The CSHQ revealed a sleep disturbance in 72.9% of subjects, but only 5.0% had already received a diagnosis of sleep disorder. We found statistically significant higher headache frequency (p=0.002) and prevalence of migraine equivalents (p=0.007) in patients with sleep disorders. A higher CSHQ total score was associated with higher severe attacks frequency (p=0.012) and lower acute drug efficacy (p=0.003). Significant positive correlations of sleep onset delay, sleep duration and nightwakings subscales with migraine frequency emerged. Our findings indicate that sleep disorders are highly prevalent in pediatric migraine and frequently associated with a higher headache severity, but remain underdiagnosed in many cases. Given the relationship between sleep and migraine characteristics, improving sleep quality could help to reduce migraine intensity and disability and vice versa.

## P010 Characteristics of pre-cluster symptoms in cluster headache

### S. Cho^1^, S. J. Cho^2^, M. J. Lee^3^, J. W. Park^4^, M. K. Chu^5^, H. S. oon^6^, P. W. Chung^6^, J. H. Sohn^7^, B. K. Kim^8,9^, D. Ki^10^, J. M. Kim^10^, J. M. Chung^11^, K. Oh^12^, J. Y. Ahn^13^, C. S.Chung^3^

#### ^1^Uijeongbu Eulji Medical Center, Neurology, Uijeongbu, South Korea; ^2^Dongtan Sacred Heart Hospital, Neurology, Hwaseong, South Korea; ^3^Samsung Medical Center, Neurology, Seoul, South Korea; ^4^Uijeongbu St. Mary’s Hospital, Neurology, Uijeongbu, South Korea; ^5^Severance Hospital, Neurology, Seoul, South Korea; ^6^Kangbuk Samsung Hospital, Neurology, Seoul, South Korea; ^7^Chuncheon Sacred Heart Hospital, Neurology, Chuncheon, South Korea; ^8^Bundang Jesaeng General Hospital, Neurology, Seongnam, South Korea; ^9^Nowon Eulji Medical Center, Neurology, Seoul, South Korea; ^10^Chungnam National University College of Medicine, Neurology, Daejeon, South Korea; ^11^Inje University College of Medicine, Neurology, Seoul, South Korea; ^12^Korea University College of Medicine, Neurology, Seoul, South Korea; ^13^Seoul Medical Center, Neurology, Seoul, South Korea

##### **Correspondence:** S. Cho

Background: In this study, we investigated characteristics of pre-cluster symptoms in patients with cluster headache.

Methods: In this multi-center study, 190 patients with cluster headache patients (184 episodic and 6 chronic cluster headache) were recruited between October 2018 and December 2020. Patients were asked about the prediction of upcoming cluster bout. For the characteristics of pre-cluster symptoms, we selected the 20 relevant symptoms and signs. If patients have had other symptoms which were not included in the list, they could describe them in their own words.

Results: Pre-cluster symptoms were predictable in 36.8%. When present, pre-cluster symptoms occurred at a median of 7 days (IQR 2.3 to 14) before the onset of cluster bout. The most frequent symptom in the pre-cluster symptoms was painful symptom (25.8%). Patients with pre-cluster symptoms had higher female proportion, prevalence of pre-attack symptom and seasonal rhythmicity, frequency of cluster headache attack per day, and total number of cluster bouts than patients without pre-cluster symptoms. In univariable and multivariable logistic regression analysis, being female was associated with the predictability of pre-cluster symptoms (OR=2.026, *p*=0.039).

Conclusions: Pre-cluster symptoms were predictable in about two-fifth of patients with cluster headache, which may allow earlier preventive treatment and help understanding pathophysiology.

## P011 Treatment pattern and response of cluster headache in Korea

### M. J. Lee^1^, S. J. Cho^2^, J. W. Park^3^, M. K. Chu^4^, H. S. Moon^5^, P. W. Chung^5^, J. M. Chung^6^, J. H. Sohn^7^, B. K. Kim^8,9^, S. K. Kim^10^, T. J. Song^11^, Y. J. Choi^12^, K. Y. Park^13^, K. Oh^14^, J. Y. Ahn^15^, K. S. Lee^16^, C. S. Chung^1^

#### ^1^Samsung Medical Center, Sungkyunkwan University School of Medicine, Neurology, Seoul, South Korea; ^2^Dongtan Sacred Heart Hospital, Neurology, Hwaseong, South Korea; ^3^Uijeongbu St.Mary’s Hospital, Neurology, Uijeongbu, South Korea; ^4^Severance Hospital, Yonsei University College of Medicine, Neurology, Seoul, South Korea; ^5^Kangbuk Samsung Hospital, Seoul, South Korea; ^6^Inje University College of Medicine, Seoul, South Korea; ^7^Chuncheon Sacred Heart Hospital, Chuncheon, South Korea; ^8^Eulji University School of Medicine, Seoul, South Korea; ^9^Bundang Jesaeng General Hospital, Seongnam, South Korea; ^10^Gyeongsang National University College of Medicine, Jinju, South Korea; ^11^Ewha Womans University College of Medicine, Seoul, South Korea; ^12^Presbyterian Medical Center, Jeonju, South Korea; ^13^Chung-Ang University Hospital, Seoul, South Korea; ^14^Korea University College of Medicine, Seoul, South Korea; ^15^Seoul Medical Center, Seoul, South Korea; ^16^Seoul St. Mary’s Hospital, Seoul, South Korea

##### **Correspondence:** M. J. Lee

Background and objective: No data regarding treatment status and response of cluster headache have been reported from Asian population.

Methods: In this multicenter study, patients with cluster headache were recruited between September 2016 and January 2019 from 16 hospitals in Korea. At baseline visit, we surveyed the patients about previous experience of CH treatment, and acute and/or preventive treatments were prescribed by the physician"s discretion. Treatment response was prospectively evaluated using a structured case report form.

Results: Among 295 patients recruited, 262 within the active bout was included in this analysis. An experience of disease-specific treatments was reported by only one third of patients. At the baseline visit, oral triptans (73.4%), verapamil, (68.3%), and systemic steroids (55.6%) were the top three most common treatments prescribed by the investigators. For the acute treatment, oral triptans and oxygen were effective in 90.1% and 86.8%, respectively. For the preventive treatment, verapamil, lithium, systemic steroids, and suboccipital steroid injection were effective in 85.5%, 75.0%, 91.8%, and 80.6%, respectively.

Conclusions: Our data provide the first prospective analysis of treatment response in Asian population. Patients well responded to treatments despite of a limited availability of treatment options. Most patients were undertreated previously, suggesting a need of raising awareness of CH among primary physicians.

## P012 The Gut, the Brain and Migraine: When Pills Dont Work

### S. Aurora^1^, S. Shrewsbury^1^, L. Nguyen^2^, N. Hindiyeh^3^

#### ^1^Impel NeuroPharma, Medical Affairs, Seattle, WA, United States; ^2^Stanford University, Gastroenterology, Redwood City, CA, United States; ^3^Stanford University, Neurology, Stanford, CA, United States

##### **Correspondence:** S. Aurora

Objective: Migraine is often complicated by GI conditions such as gastroparesis, functional dyspepsia - both associated with delayed gastric emptying, and cyclic vomiting syndrome. For example, GI comorbidity was reported in 38.4% of 354 subjects enrolled in STOP 301, with 20.3% reporting GERD. Here we review the current state of scientific evidence that exists linking migraine and gastric stasis.

Methods: Key words, gastric stasis, migraine, autonomic dysfunction were used to obtain relevant studies in a literature search of EMBASE and PubMED.

Results: Delayed aspirin absorption was reported in 19 out of 42 patients during a spontaneous attack, but not interictally. However, scintigraphy studies showed that gastric emptying after an induced migraine attack was delayed 78% ictally, and 80% interictally. Delayed emptying during spontaneous migraine attacks was reported as well but others have reported contradictory results. Compared to migraine patients, subjects with functional dyspepsia had more delayed emptying and others reported delayed ictal, but not interictal, emptying in patients compared to controls. An NIH Gastroparesis consortium survey reported migraine as the most common extra-GI comorbidity (36.6%) and was associated with more severe gastroparesis symptoms.

Conclusion: The association between gastroparesis and migraine may be important if patients have GI symptoms and do not experience migraine relief with oral abortive treatment.

## P013 Sleep assessment in patients with Cluster headache – self reported vs observed data

### C. Ran^1^, C. Fourier^1^, F. Jennysdotter Olofsgård^1^, C. Wirth^1^, A. Steinberg^2,3^, C. Sjöstrand^2^, E. Waldenlind^2,3^, A. Dahlgren^2^, A. Carmine Belin^1^

#### ^1^Karolinska Institutet, Department of Neuroscience, Stockholm, Sweden; ^2^Karolinska Institutet, Department of Clinical Neuroscience, Stockholm, Sweden; ^3^Karolinska University Hospital, Department of Neurology, Stockholm, Sweden

##### **Correspondence:** C. Ran

Objective: Cluster Headache (CH) is a primary headache disorder often characterized by a circadian timing of headache attacks. The hypothalamus is reported to be activated during attacks, and several genes involved in the regulation of the molecular clock have been linked to CH. To investigate this further, we analyzed sleep patterns in CH patients compared to controls and in relation to active period and remission.

Methods: 92 individuals were recruited for sleep assessment, 42 controls and 50 patients. Sleep was recorded during a two-week period using MotionWatch 8 actigraphs (CamNTech) containing an accelerometer recording physical movement. Study participants were instructed to wear the unit during rest and sleep and to fill out a short version of the Karolinska Sleep Diary in order to compare recorded sleep data with perceived sleep.

Results: 77 individuals have completed the study, two individuals discontinued the study because of technical difficulties, and six because of personal reasons or health problems. Preliminary results from the sleep diary suggests that CH patients take significantly longer time to fall asleep compared to controls, 30 min. vs 15 min., and CH patients remain in bed for a longer time in the morning compared to controls, 40 min. vs 20 min.

Conclusion: Our preliminary data suggest that sleep is affected in CH patients, manifesting in prolonged sleep latency and increased time in bed. These data will be verified using actigraphy.

## P014 Prevalence of pre-cluster symptoms in episodic cluster headache: Is it possible to predict an upcoming bout?

### A. S. Pedersen^1^, A. Snoer^1^, M. Barloese^1^, A. S. Petersen^1^, R. H. Jensen^1^

#### ^1^Danish Headache Center, Glostrup, Denmark

##### **Correspondence:** A. S. Pedersen

Background: Early symptoms prior to a cluster headache bout have been reported to occur days or weeks before the actual beginning of the cluster headache bouts. This study aimed to describe the prevalence of pre-cluster (premonitory) symptoms and examine the predictability of an upcoming cluster headache bout.

Methods: 100 patients with episodic cluster headache were included in this retrospective cross-sectional study. All patients underwent a semi-structured interview including 25 questions concerning pre-cluster symptoms.

*Results:* Pre-cluster symptoms were reported by 86% of patients with a mean of 6.8 days (interquartile range 3-14) preceding the bout. An ability to predict an upcoming bout was reported by 57% with a mean 4.6 days (interquartile range 2-7) before the bout. Occurrence of shadow attacks was associated with increased predictability (odds ratio: 3.06, confidence interval: 1.19-7.88, p-value=0.020). In remission periods, 58% of patients reported mild cluster headache symptoms and 53% reported occurrence of single shadow attacks.

Conclusions: The majority of episodic cluster headache patients experienced pre-cluster symptoms, and more than half could predict an upcoming bout, suggesting a significant potential of early intervention. Furthermore, the experience of mild cluster headache symptoms and infrequent shadow attacks in remission periods is common and suggest an underlying pathophysiology extending beyond the cluster headache bouts.

**Fig. 1 (abstract P014).**
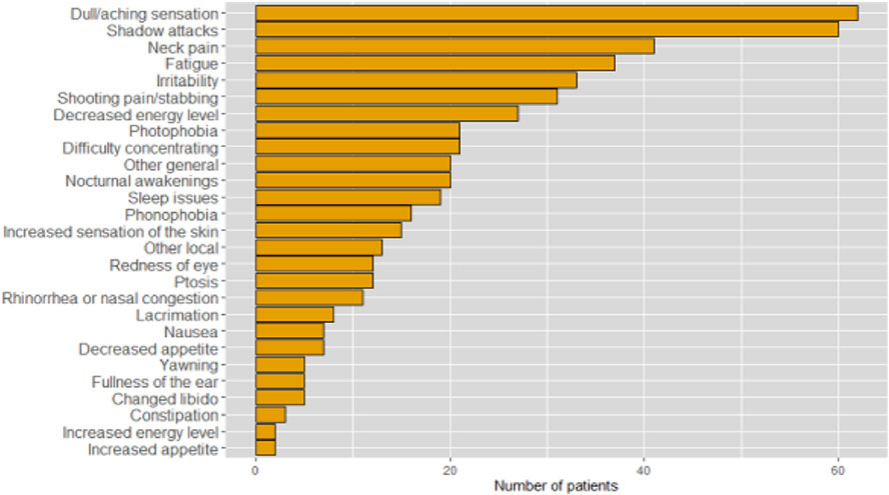
See text for description

**Fig. 2 (abstract P014).**
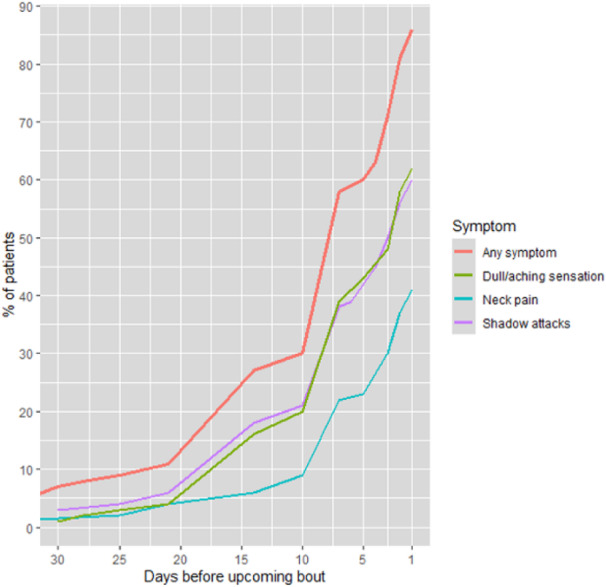
See text for description

## P015 Clock gene expression amongst a population of cluster headache patients

### R. Oliveira^1^, A. Neves-Costa^2^, D. Pedroso^2^, A. B. Barros^2^, L. F. Moita^2^, R. Gil-Gouveia^1^

#### ^1^Hospital da Luz Lisboa, Neurology, Lisbon, Portugal; ^2^Instituto Gulbenkian de Ciência, Oeiras, Portugal, Lisbon, Portugal

##### **Correspondence:** R. Oliveira

Objective: To measure the expression of CLOCK gene (CLOCK) in a population of cluster headache (CH) patients and matched controls.

Methods: CH patients were sampled 2 to 4 times over one year, both in or outside bouts, one week after each solstice and equinox. Expression of CLOCK was quantified in the peripheral blood by quantitative RT-PCR.

Results: A total of 50 patients (84% males, average age of 44.6 years) of which 45(90%) suffered from episodic CH, and 58 controls were included. We had 159 samples, 36(22.6%) coinciding with CH bouts. CLOCK expression was not significantly different between bout and non-bout samples– Spring(average expression of 1.03 vs 1.24, p=0.475); Summer(average expression of 1.44 vs 1.18, p=0.268) and Fall(average expression of 1.35 vs 1.32, p=0.815). Furthermore, multivariate analysis with individual factors (age, sex, circadian chronotype, smoking and coffee habits, and history of migraine) did not show any differences between non-bout and bout across seasons. In Winter, CLOCK expression was lower in non-bout CH patients than controls (average expression of 0.63 vs 1.17, p=0.01). Comparing the expression levels of patient"s season by season, the expression of CLOCK was significantly lower in Winter in comparison with Fall(p<0.01), Spring(p<0.01) and Summer(p<0.01)

Conclusion: CLOCK expression in CH patients varies along the seasons being lower in the December solstice. Bout activity does not seem to influence CLOCK expression.

## P016 Evaluating the real-world burden of migraine using Migraine Buddy

### P. J. Goadsby^1^, C. Amand^2^, L. Constantin^2^, S. Hitier^2^

#### ^1^KCL, London, United Kingdom; ^2^Sanofi, Gentilly, France

##### **Correspondence:** P. J. Goadsby

Objective: To use data from a mobile phone application to study real-world burden of self-diagnosed headache and describe its impact on daily life in headache sufferers not routinely seek medical advice.

Methods: This retrospective, non-interventional, cross-sectional study analysed self-reported data from the "Migraine Buddy" app. Self-reported characteristics of headache and migraine, such as triggers, duration, frequency; treatment patterns and impact on daily activity in headache sufferers from Australia, Brazil, France, Germany, Japan were described. Demographics, self-diagnosed episode type: headache/migraine, duration, potential triggers, and impact on daily activity are reported. All analyses were exploratory and performed per country.

Results: Self-reported data were collected from 60,474 users between August 2016–August 2018. Of users ~90% were females; >60% was aged 24–45 years. Over one-third of users reported having 2–5 episodes of headache or migraine per month; impact included impaired concentration, being slower and missing work or social activities. Variation across countries were observed; within countries, episode characteristics were very similar for self-diagnosed headache vs migraine.

Conclusions: Headache disorders present a range of important issues for patients that deserve more study and reinforce the need for better approaches to management. Big data can provide directions and potential insights to help improve headache management broadly.

## P017 Add on treatment with galacanezumab improved refractory cluster headache in 5 out of 6 tested cases

### G. Karagiorgis^1^, E. Kasioti^1^, E. Mitropoulou^1^, S. Tsanoula^1^, T. Mavridis^1^, D. Mitsikostas^1^

#### ^1^Eginition Hospital, 1st Department of Neurology, Athens, Greece

##### **Correspondence:** G. Karagiorgis

Objective: To observe whether galcanezumab, a monoclonal antibody targeting the calcitonin gene-related peptide (CGRP), improves cluster headache (CH) as add-on treatment in a real-world setting.

Methods: We prospectively collected data from 6 refractory CH patients (3 with episodic CH and 3 with chronic CH) at weeks 1 through 4, following the first dose of galcanezumab (120mg, sc).

Results: The average number of previous treatments with limited or no response was 3.6 (range 2-5). At baseline the average number of attacks per week was 22.7. After adding galcanezumab the frequency of attacks decreased by 17.1 across weeks 1 through 4. One patient became headache free; in 2 patients a more than 75% and in other 2 a more than 50% reduction of attacks was recorded. One patient with episodic CH did not report changes in headache frequency. A significant reduction in days of acute medication use was noted in all cases (vs. baseline). Reduction in attack frequency started at week 1 and was consistent throughout the observation period. No adverse effect was noted.

Conclusion: Galcanezumab was effective and safe in 5 out of our 6 CH patients, supporting individual off-label treatment attempts with anti-CGRP/R antibodies in refractory and disabled CH patients with poor outcomes.

## P018 Cluster Headache Impact Questionnaire (CHIQ) – A tool for assessing disability in cluster headache patients

### K. Kamm^1^, A. Straube^1^, R. Ruscheweyh^1^

#### ^1^Ludwig Maximilians University, Department of Neurology, Munich, Germany

##### **Correspondence:** K. Kamm

Objective: Cluster headache (CH) is a severe, highly disabling primary headache disorder. The aim of this study was to develop a CH-specific, short questionnaire to assess disability due to CH.

Methods: Based on a literature review and semi-structured interviews with CH patients and headache experts, the 8-item Cluster Headache Impact Questionnaire (CHIQ) was developed and pretested. Subsequently, the CHIQ was administered online or on paper to CH patients visiting our headache center or via a German patient group. Reliability and validity were evaluated.

Results: Active episodic (n = 85) and chronic (n = 111) CH patients (65.3% male, 47.21 ± 11.64 years) were included. The CHIQ showed good internal consistency (Cronbach"s α = 0.88) and factor analysis identified a single factor. Test-retest reliability was adequate (ICC 0.82, n = 38). Convergent validity was shown by significant correlations with the Headache Impact Test™ (HIT-6™, r = 0.62, p < 0.01), subscales of the depression, anxiety and stress scales (DASS, r = 0.46 - 0.59; p < 0.01) and with CH attack frequency (r = 0.39; p < 0.01).

Conclusion: The CHIQ is a short, CH-specific questionnaire for the assessment of the impact of CH. The questionnaire is reliable, valid, and easy to administer which makes it a useful tool for clinical use and research.

## P019 Telencephalic cortical thickness in chronic cluster headache

### L. Giani^1^, G. Demichelis^2^, C. Pinardi^2^, J. P. Medina^2^, R. Gianeri^2^, M. G. Bruzzone^2^, B. Becker^3^, A. Proietti Cecchini^1^, L. Chiapparini^2^, S. Ferraro^2,3^, A. Nigri^2^, M. Leone^1^

#### ^1^Fondazione IRCCS Istituto Neurologico Carlo Besta, Neuroalgology, Milan, Italy; ^2^Fondazione IRCCS Istituto Neurologico Carlo Besta, Neuroradiology, Milan, Italy; ^3^University of Electronic Science and Technology of China, School of Life Science and Technology, MOE Key Laboratory for Neuroinformation, Chengdu, China

##### **Correspondence:** L. Giani

Objective: Previous studies on brain morphologiy in chronic cluster headache (CCH) revealed inconsistent findings, maybe due to limitations of VBM. We investigated telencephalic cortical thickness in CCH patients employing a highly robust state-of-the-art approach for thickness estimation (Freesurfer).

Methods: CCH patients (n=28; 23 males; age 45±11.7) and sex- and age-matched healthy individuals were scanned with a 3T-MRI for 3D-T1 images. No other pain, vascular or psychiatric comorbidities were admitted. We used Freesurfer 6 to obtain surface-based individual telencephalic cortical thickness estimates. CCH and controls were compared with a vertex-wise between-group analysis. Results were considered significant with a vertex-wise threshold of p<0.001 and a cluster-wise threshold of 50 mm2.

Results: CCH patients showed significant cortical thinning in the right midcingulate cortex (MCC), the left posterior insula (postIC), the left superior temporal sulcus (STS) and the left collateral/lingual sulcus (CLS) (p<0.001 for all).

Conclusions: CCH patients show abnormalities in regions belonging to the spino-thalamo-cortical tract, involved in sensory-motivational aspects of nociception (MCC, postIC), and in areas involved in social cognition (STS, CLS), a possible expression of behavioral/psychological vulnerability of CCH patients.

Acknowledgements: This work was supported by the Italian Ministry of Health (RF-2016-02364909)

## P020 Dopaminergic system abnormalities in chronic cluster headache patients

### L. Giani^1^, S. Ferraro^2,3^, A. Nigri^2^, M. G. Bruzzone^2^, C. Pinardi^2^, G. Demichelis^2^, L. Chiapparini^2^, A. Proietti Cecchini^1^, M. Leone^1^

#### ^1^Fondazione IRCCS Istituto Neurologico Carlo Besta, Neuroalgology, Milan, Italy; ^2^Fondazione IRCCS Istituto Neurologico Carlo Besta, Neuroradiology, Milan, Italy; ^3^University of Electronic Science and Technology of China, School of Life Science and Technology, MOE Key Laboratory for Neuroinformation, Chengdu, China

##### **Correspondence:** L. Giani

Objective: Diencephalic-mesencephalic structures have been implicated in the pathogenesis of cluster headache (CH). Among them, the ventral tegmental area (VTA) is part of the mesocorticolimbic dopaminergic system, which is involved in rewarding, avoidance, chronic pain. We hypothesized structural abnormalities in elements of the mesocorticolimbic system in patients with chronic CH (CCH).

Methods: Patients with CCH (n=28; 5 females; age 45±11.7) and age- and sex- matched healthy controls were scanned for volumetric T1-w image. We segmented cortical and subcortical areas of the mesocorticolimbic system with FreeSurfer: ventral diencephalon (comprising basal forebrain, VTA, hypothalamus), hippocampus, amygdala, n. accumbens, frontal pole and pars orbitalis frontal cortex. We assessed the association between the volume of each region with CCH diagnosis by univariate logistic regression.

Results: Higher volumes of bilateral n. accumbens (p=0.008 left, p=0.033 right), ventral diencephalon (p=0.024 left, p=0.040 right), left pars orbitalis (p=0.035), bilateral frontal pole (p=0.047 left, p=0.039 right), right hippocampus (p=0.025) were associated with CCH.

Conclusions: CCH patients showed increased volumes in structures of the mesocorticolimbic system, opposite to what commonly seen in other chronic pain conditions. CCH seems to have a peculiar alteration in systems involved in affection and motivation.

Acknowledgements: Supported by the Italian Ministry of Health (RF-2016-02364909).

## P021 Co-occurring neck-pain in patients with episodic migraine and analgesic intake

### O. Dubenko^1^, A. Chernenko^1^

#### ^1^Kharkiv Medical Academy of Postgraduate Education, Neurology and neurosurgery, Kharkiv, Ukraine

##### **Correspondence:** A. Chernenko

Objective: Comorbid and co-occurring diseases are risk factors for the progression of episodic migraine to chronic migraine. Overuse of analgesics is common problem in patients with primary headache. The aim - to estimate the influence of co-occurring neck pain on the number of analgesics taken in patients with episodic migraine.

Methods: The study included 92 patients (male 24, female 68, mean age 42.5±15.5). Three groups were identify: 1) both episodic migraine and neck pain (31); 2) episodic migraine only (30); 3) neck pain only (31). Visual analogue scale (VAS), Migraine Disability Assessment (MIDAS) and numbers days with analgesics intake were assessment. The disc herniation and root compression were excluded.

Results: In patients group with co-occurring migraine and neck pain number of days with headache for 3 months was significantly greater (p=0.000052), the intensity of pain during a migraine attack on the VAS was higher (p=0.003750) and the disability according to the MIDAS was more significant versus migraine only (p=0.00048). Number days with analgesic intake per month was greater in first group – 7.06±0.96 and 6 (19.35%) of them had sigh of medication overuse, in migraine only – 1.20±0.35, in neck pain only – 3.19±0.83 (p=0.000003).

Conclusion: In patients with combined episodic migraine and neck-pain observed increase number days with analgesic intake that may be risk for chronification headache.

## P022 Differences in sensory nucleus of the trigeminal nerve, dorso-lateral pons, and somatosensory cortex between migraine patients and healthy controls. An MRI post-processing study

### N. Morollón^1,2^, R. Belvís^1,2^, G. Garcia^3^, N. Mas^2^, R. Ramos^4^, J. Kulisevsky^1,2^

#### ^1^Hospital de la Santa Creu i Sant Pau, Neurology, Barcelona, Spain; ^2^Hospital Universitario Quirón Dexeus, Neurology, Barcelona, Spain; ^3^Hospital Quirón Valencia, Bioengineering, Valencia, Spain; ^4^Hospital Universitario Quirón Dexeus, Radiology, Barcelona, Spain

##### **Correspondence:** N. Morollón

Background: Neuroimaging studies have been carried out to analyze whether there are microstructural alterations in white matter and gray substance in patients with migraine.

Methods: This is a single-center case-control study based on structural magnetic resonance image (MRI) description in migraine to compare the thickness and volume of brain gray matter and the diffusivity and anisotropy of brain white matter regions involved in the pathophysiology of migraine in patients and controls. Images were collected using 1.5T MRI. Post-processing of the cortical morphometry images (Statistical Parametric Mapping-12 and Freesurfer) and microstructural analysis in white matter (diffusion tensor image) of regions of interest (somatosensory cortex, visual areas (V3, MT+), hypothalamus, caudal portion of the sensory nucleus of the trigeminal nerve and dorsolateral pons) were extracted.

Results: 128 patients with migraine (69 without aura, 46 with aura) and 48 controls were included. The statistically significant differences (p<0.05) found were a volume and thickness increase in the gray matter of somatosensory cortex that is influenced by disease duration, a reduction in gray matter volume in the caudal portion of the sensitive nucleus of trigeminal nerve, as well as a reduction in the fractional anisotropy of the dorso-lateral pons in patients with migraine.

Conclusions: Patients with migraine present micro-structural changes in regions of interest related to the pathophysiology of migraine. Our results suggests an altered anatomical substrate may correlate with the transmission, modulation, and perception of pain.

**Fig. 1 (abstract P022).**
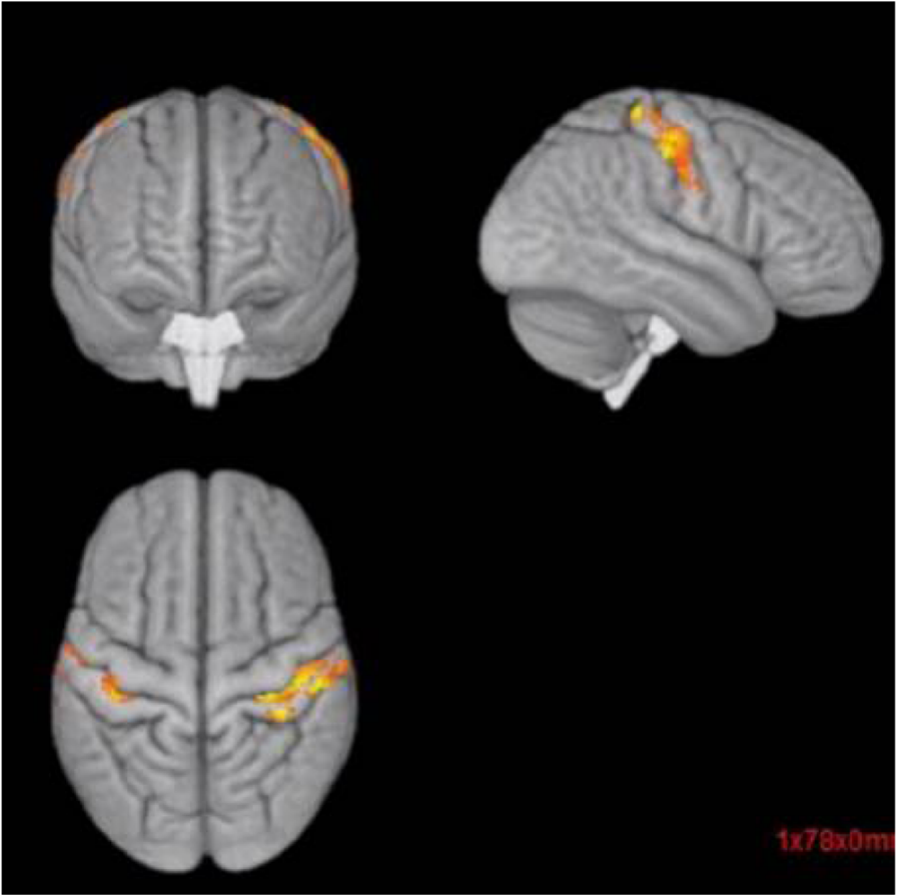
See text for description

**Fig. 2 (abstract P022).**
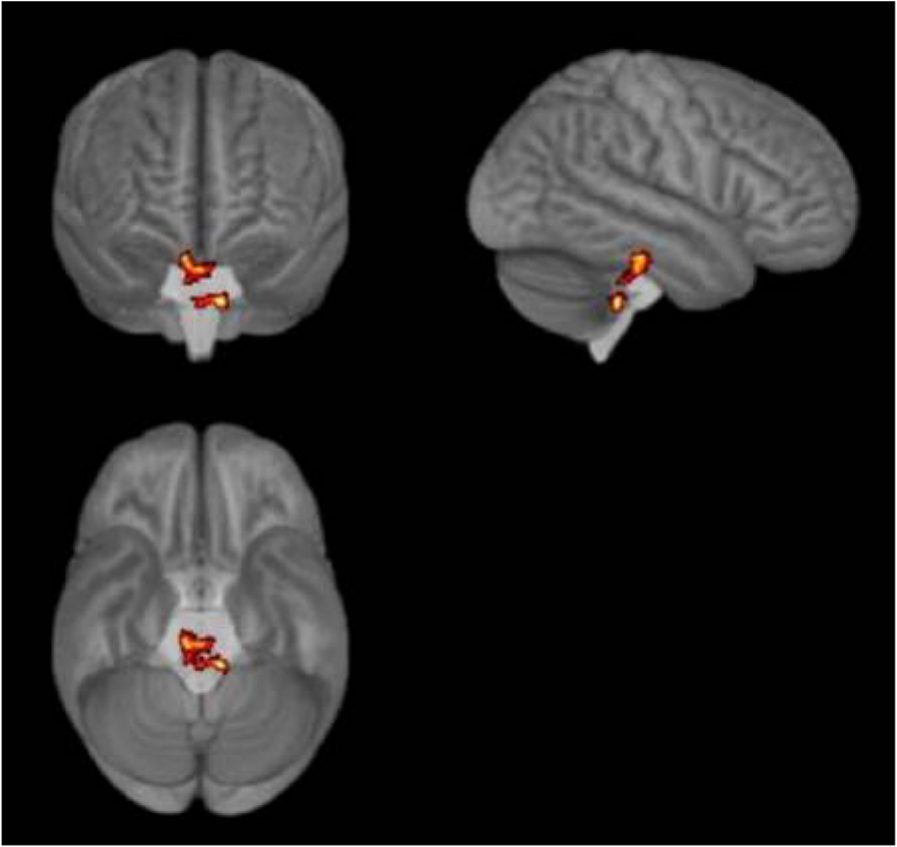
See text for description

## P023 Nasopharyngeal swab may trigger migraine by peripheral stimulation of trigeminal nerve

### J. Madera Fernández^1^, E. M. Rodríguez Rodríguez^1^, V. González-Quintanilla^1^, S. Pérez-Pereda^1^, J. Pascual^1^

#### ^1^Hospital Universitario Marqués de Valdecilla, Neurology, Santander, Spain

##### **Correspondence:** J. Madera Fernández

Background: Despite there is no doubt that certain structures of the central nervous system, such as the hypothalamus or some brainstem nuclei, play a key role in migraine generation, migraine attacks can also be triggered by peripheral trigeminal nerve stimuli. Our aim was to study the possible role of nasopharyngeal swab (NS) performed for SARS-CoV-2 determination as a potential trigger of migraine attacks.

Methods: Descriptive and retrospective study through an online survey conducted among healthcare professionals of our hospital. *Primary objective:* To determine the possible role of the NS as a trigger for migraine attacks (MA), comparing the percentage of participants who presented a MA in the following 24h after performing the PCR test in migraineurs vs non migraineurs. *Secondary objective:* To evaluate the characteristics of post-PCR headache in migraineurs.

Results: A total of 309 people were included. 36,6% were migraineurs. 47 participants (15,2%) reported MA in the next 24h after the PCR test. The percentage of headache after PCR test was 37.2% in migraineurs vs 2.6% in non-migraineurs (X^2^=66.6 p<0.00) with an odds ratio to develop a MA in the next 24h after the test in migraineurs vs non migraineurs of 22,6 (95% CI[8.6-59.4]). Characteristics of post-PCR headache, including migraine aura,didn't differ from previous migraine attacks.

Conclusions: NS may induce MA in migraineurs, confirming the claim that purely peripheral stimuli could induce MA in these patients.

**Fig. 1 (abstract P023).**
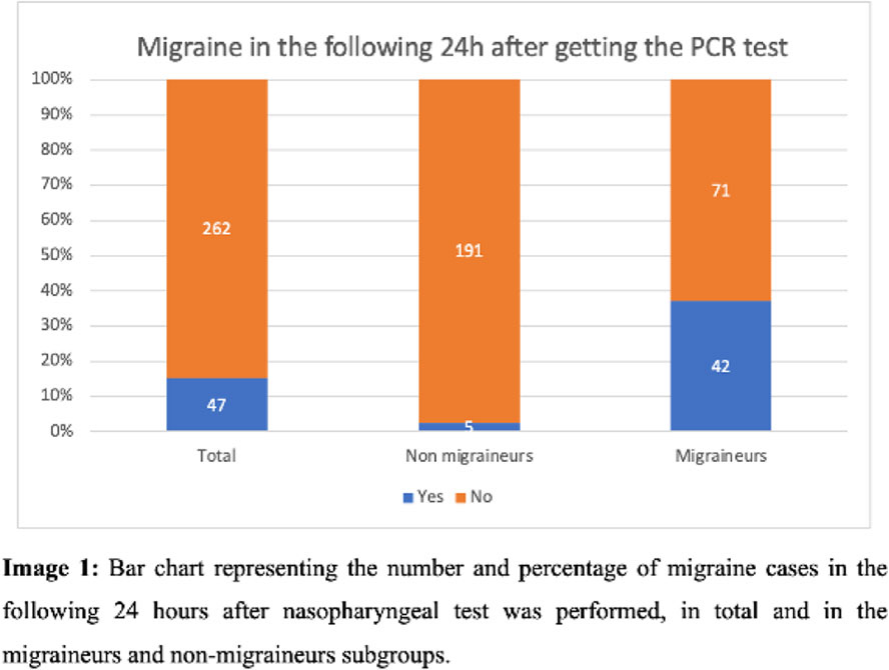
See text for description

**Table 1 (abstract P023).**
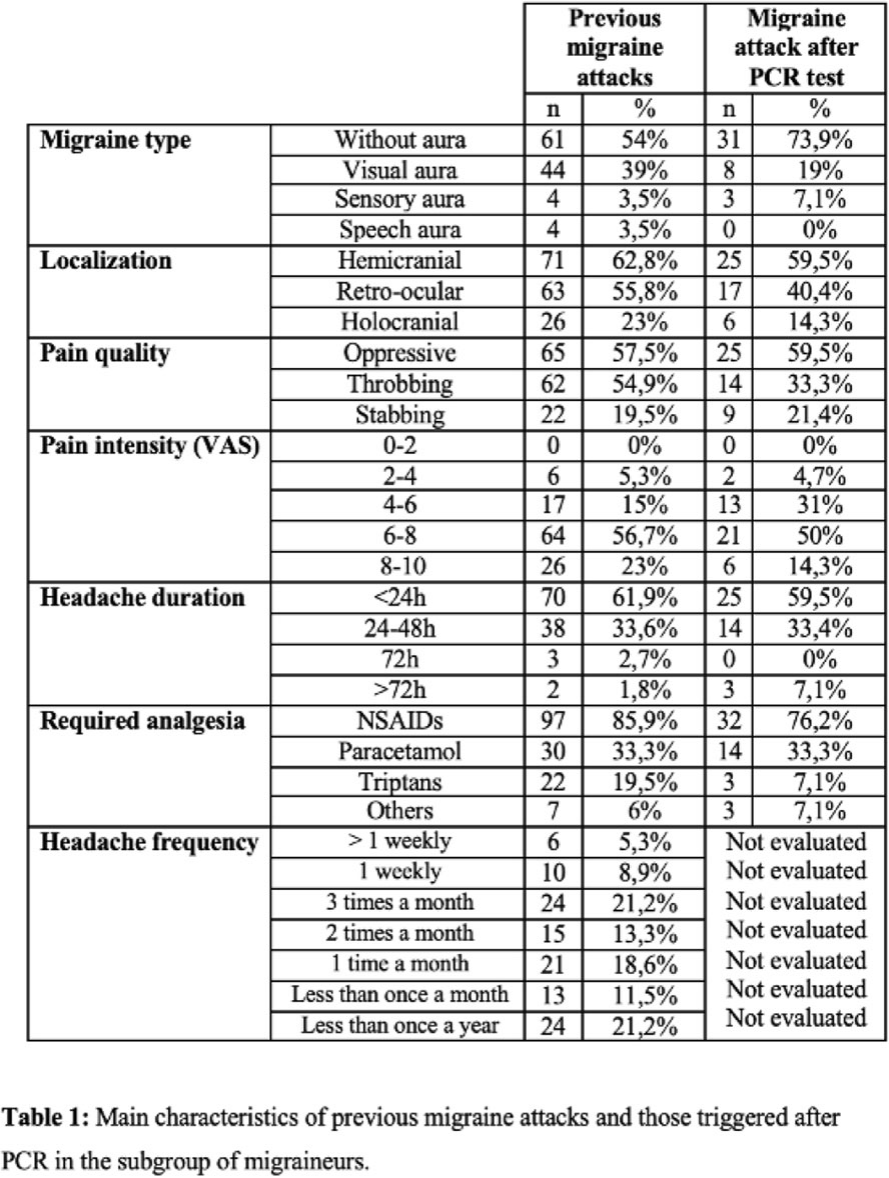
See text for description

## P024 Impact of COVID-19 lockdown in Argetinean patients with migraine

### M. V. Nagel^1^, M. T. Goicochea^1^, L. Bonamico^1^, N. Larripa^1^, T. Gutierrez^1^, M. Olivier^1^, M. Grandinetti^1^, D. Calvo^1^

#### ^1^FLENI, Neurology, Buenos Aires, Argentina

##### **Correspondence:** M. V. Nagel

Objective: COVID-19 lockdown may change habits in migraine patients. Such modifications can impact the frequency and/or severity of migraine. The objective of this work is to evaluate the impact of lockdown in patients with migraine.

Methods: Descriptive, retrospective study. We reviewed electronical medical records of patients evaluated during lockdown in headache clinic in a neurological center between May 26 and June 30, 2020. Diagnoses, evolution of headache, current workplace and different factors reported by patients were considered. Study approved by institution's ethics committee.

Results: 304 patients were evaluate: 88% women, mean age 41 years. 40% were evaluated by telemedicine. Episodic migraine represented 52%. 50% worsened their headache, 29% remained stable and 21% improved. Among those who worsened, main causes were work (29%) and mood changes (24%). Patients who improved associated it with pharmacological preventive therapies (35%) and causes related to work (28%). In all groups, most of the patients modified their work activity incorporating home office (61%).

Conclusions: Half of the patients perceived a worsening, but a significant percentage improved. Reasons related to work were the main cause of worsening, followed by mood changes. Improvement was attributed to the pharmacological preventive therapies and work modifications. Home office was the most frequent job change. This way of working can impact differently on each patient.

## P025 Hormonal changes as an aetiological factor in trigeminal neuralgia in women

### M. Noory^1,2^, A. S. S. Andersen^1^, N. Noory^1^, T. B. Heinskou^1^, L. Bendtsen^1^, D. M. Kristensen^1,3^, C. S. J. Westgate^1^, S. Maarbjerg^1^

#### ^1^Rigshospitalet-Glostrup, Department of Neurology, Danish Headache Center, Glostrup, Denmark; ^2^University of Copenhagen, Medicine, Copenhagen, Denmark; ^3^Université de Rennes, Inserm, EHESP, Irset, Rennes, France

##### **Correspondence:** M. Noory, S. Maarbjerg

Objective: Previous studies have shown TN to be significantly more prevalent and to have an earlier age of onset in women than in men, and that women less frequently have a neurovascular contact with morphological changes of the trigeminal nerve as the cause of TN. These findings are to date not explained. This case-control study investigates the possible role of sex hormones in women with primary trigeminal neuralgia (TN).

Methods: Women with TN below the age of 60 years were included consecutively and interviewed with a semi-structured questionnaire. Patients were then age-matched with healthy controls.

Results: A total of 76 patients with TN and 76 healthy controls were included. We found that women with TN did not differ from healthy controls with respect to age of menarche and (peri)menopause or to the number of pregnancies and duration of breastfeeding prior to TN onset. Patients with TN were more likely to suffer from other headache disorders, other chronic pain conditions and psychiatric disease compared to healthy controls.

Conclusions: We did not find any differences between patients with TN and controls with respect to major natural events leading to changes in the female sex hormones. Our findings do not exclude that the possible significance of sex hormones in TN may be related to an individual heightened sensitivity to natural hormonal fluctuations possibly contributing to dysmyelination of the trigeminal nerve.

## P027 The neurological manifestations of COVID-19: a retrospective single-center clinical study in the Republic of Dagestan, Russia

### Z. Gadzhieva^1^, Z. Khamidova^1^, T. Magomedova^1^, Z. Kaplanova^1^

#### ^1^GBU RD RDC, Neurology, Makhachkala, Russian Federation

##### **Correspondence:** Z. Gadzhieva

Background, objective: Numerous studies have demonstrated that patients with COVID-19 may develop neurological complications, including headaches. Despite this, the available data on the clinical characteristics of affected patients remain limited. The purpose of the study was to analyze the characteristics of headaches in COVID-19 patients.

Methods: A retrospective study of the patients with confirmed COVID-19 was conducted from 1st May to 30th June 2020.Epidemiological, demographic, clinical, laboratory and radiological data were collected and analyzed. The study was approved by the local ethics committee.

Results: 175 COVID-19 patients were enrolled. The mean age was 49,8±12,3years (64%females).

The leading neurological signs were fatigue (81,2%), headache (64,6%), anosmia/ageusia (54,8%/52,0%), anxiety/depression (58,8%/57,7%). The main features of headache were bilateral localization (72.5%), a pressing quality (69.1%), onset in the frontal and periorbital regions (60.2%) and spontaneous regression after the acute phase of the disease. 57,5% of the patients had no previous history of any primary headache. Headache intensity was significantly higher in patients with more severe lung damage (p=0.033), probably due to hypoxia.

Conclusions*:* Most of the neurological manifestations were comparable in frequency to those reported in the literature. The incidence of headache in our population was higher than reported, possibly due to the higher rate of primary headache history in our sample of patients.

## P028 Genome-wide Analyses Identify the Genetic Landscape and Polygenic Risk Model of Reversible Cerebral Vasoconstriction Syndrome

### S. P. Chen^1,2^, Y. F. Wang^1,2^, C. L. Hsu^3^, J. L. Fuh^1,2^, Y. H. Ling^1,2^, L. L. Pan^2^, C. S. Fann^3^, S. J. Wang^1,2^

#### ^1^Taipei Veterans General Hospital, Department of Neurology, Taipei City, Taiwan; ^2^National Yang Ming Chiao Tung University, Taipei City, Taiwan; ^3^Academia Sinica, Taipei City, Taiwan

##### **Correspondence:** S. P. Chen

Background and Objective: Reversible cerebral vasoconstriction syndrome (RCVS) is a complex neurovascular disorder with unclear pathogenesis. The objective of this study is to identify potential genetic determinants of RCVS.

Methods: We performed a two-stage genome-wide association study (GWAS) in totally 544 RCVS patients and 2,370 population-based healthy controls. We also developed a genome-wide polygenic risk score (GPS) to differentiate patients from controls and to identify patients at risks of complications.

Results: We identified three risk variants for RCVS including rs8015178 in *SLC24A4* (odds ratio (OR) = 0.378 [95% confidence interval (CI)], *p* = 1.54×10-27); rs10460143 in *WDR7* (OR = 2.969 [95% CI], *p* = 1.73×10-15); and rs78378504 in *PLD5* (OR = 2.225 [95% CI ], *p* = 2.27×10-12), with the latter two also significantly associated with BBB disruption (*p* < 1×10-15). The GRS well differentiates patients from controls with an area under the receiver operating characteristic curve 0.825 (*p* = 1.01×10-94). A GRS above the 95 percentile of controls also identified patients with 9.3-fold risk of ischemic stroke, 3.8-fold risk of overall complications, and 2.7-fold risk of BBB disruption.

Conclusions: Our study revealed the genetic landscape of RCVS and provides mechanistic insights for its pathogenesis. In addition, GPS may be useful to identify patients at the worse end of the disease spectrum.

## P029 Impact of Lockdown during Covid-19 Pandemic in India on the Disease Activity and Quality of Life in migraine patients: A Web-Based Survey

### D. Chowdhury^1^, D. Datta^1^, A. Duggal^1^, A. Krishnan^2^

#### ^1^G B Pant Institute of Post graduate Medical Education and Research, Neurology, Delhi, India; ^2^AIIMS, Department of Community Medicine, Delhi, India

##### **Correspondence:** D. Chowdhury

Objective: To study the impact of COVID-19 pandemic related lockdown on Indian migraine patients

Methods: This cross-sectional, internet-based study recruited participants aged 18 years and above from 27th April to 31st July 2020, using a specifically designed questionnaire. Previously physician-diagnosed migraineurs and those fulfilling 2 out of 3 criteria (limitation of activities for a day or more, associated nausea or vomiting, and photophobia or phonophobia) were diagnosed as migraineurs. The primary outcome measure was change in the quality of life (QOL).

Results: 5694 persons registered and 4078 completed the full survey. 984 had migraine (347 males, 635 females, and 2 transgender; mean age 35.32 ±11.16). Increased last month attack frequency, headache days, attack duration, and headache severity was reported by 79-89% of the migraineurs. Overall, 57.4% of migraineurs reported ≥50% worsening in their headache, and 75.4% related it to lockdown. The reasons for worsening included anxiety due to the COVID-19 pandemic (72.9%); inability to access healthcare (56.5%), and financial worries (68.4%). Only 28.3% of migraineurs could access a doctor during the lockdown. A greater proportion of migraineurs reported a bad QOL compared to non-migraineurs (26.83% versus 7.37%; p<o.ooo1) and 61.4% of migraineurs reported that migraine affected their QOL.

Conclusion: COVID-19 pandemic-related lockdown greatly impacted Indian migraine patients and significantly reduced their QOL.

**Fig. 1 (abstract P029).**
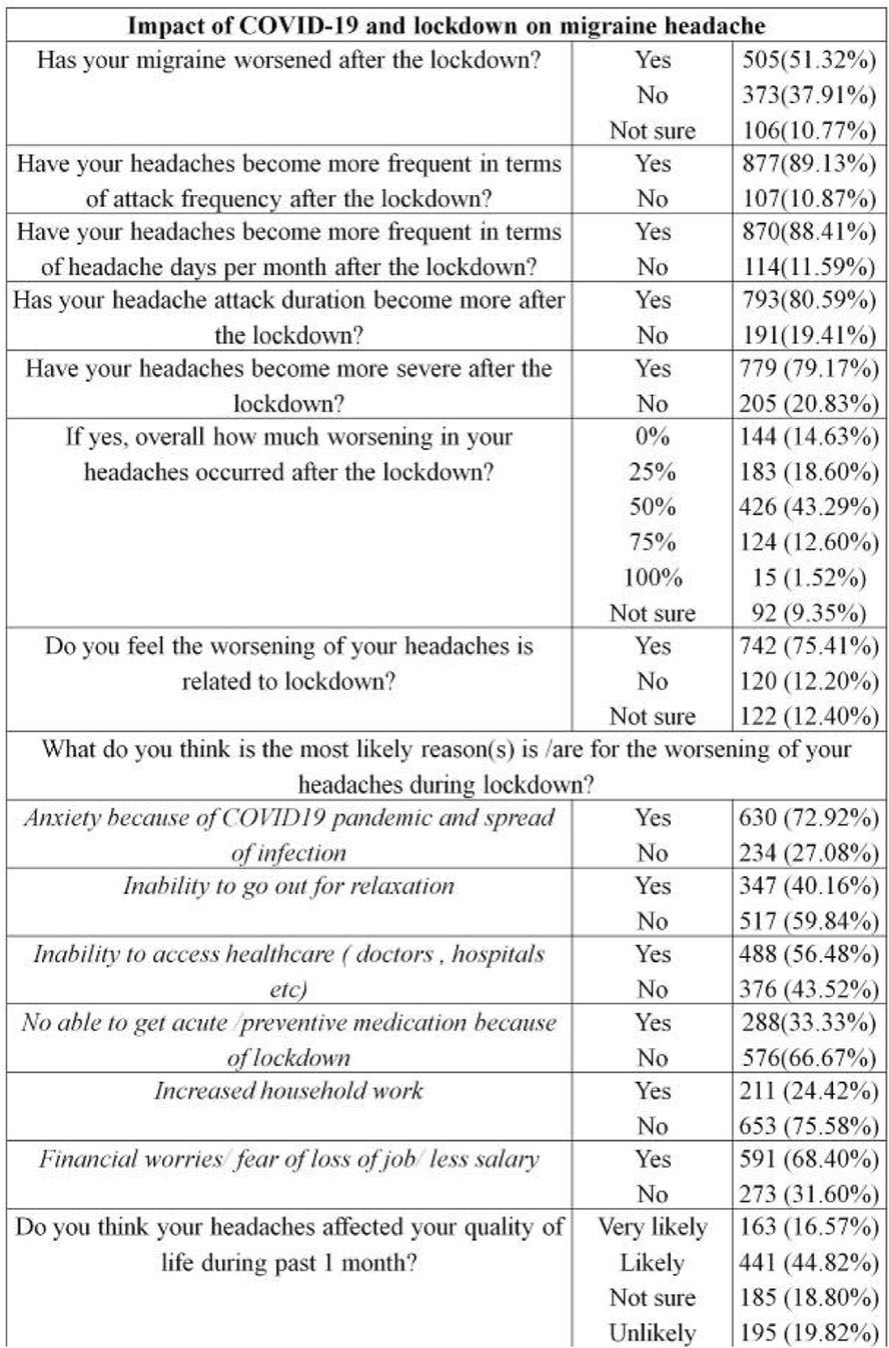
See text for description

## P030 Hypermethylation of exon 1 of MTDH/AEG1 is associated with chronic migraine. A case-control study with GWAS-discovered migraine genes

### S. Pérez-Pereda^1^, M. Toriello^2^, G. Garate^2^, V. González-Quintanilla^2^, A. Oterino^3^

#### ^1^University Hospital Marqués de Valdecilla and Cantabria University, Neurology, Santander, Spain; ^2^University Hospital Marqués de Valdecilla and IDIVAL, Neurology, Santander, Spain; ^3^University Hospital Central de Asturias, Neurology, Oviedo, Spain

##### **Correspondence:** S. Pérez-Pereda

Background and objective: Migraine is a multifactorial disease. Our aim was to investigate epigenetic DNA modifications and its relation to the risk for chronic (CM) and episodic migraine (EM).

Methods: We performed a case-control study using 3 age and sex-matched clinical groups (CM, EM and healthy control, HC). We analyzed the peripheral blood methylation level of some of the genes previously associated with migraine in GWAS through Real Time Quantitative Methylation Specific PCR.

Results: 296 subjects (101 CM, 98 EM and 97 HC) were included. After adjustment for confounding factors, only the methylation level on exon 1 of MTDH/AEG1 conferred an increased risk for CM vs EM [OR 1.675(1.1-2.6)] and vs HC [OR 1.350(1.08-1.68)]. The risk for medication overuse headache (MOH) also increased with higher MTDH/AEG1 methylation [OR 1.708(1.1-2.6)]. MTDH/AEG1 methylation level showed a positive correlation with MIDAS score (rho=0.264, p=0.002) and number of headache days/90 days (rho=0.197, p=0.027).

Conclusions: Hypermethylation on exon 1 of MTDH/AEG1, a gene involved in glutamate homeostasis, is associated with CM and MOH, and correlates with some variables of severity and impact of migraine. Hypermethylation on this region could represent and adaptive epigenetic mechanism for migraine.

**Fig. 1 (abstract P030).**
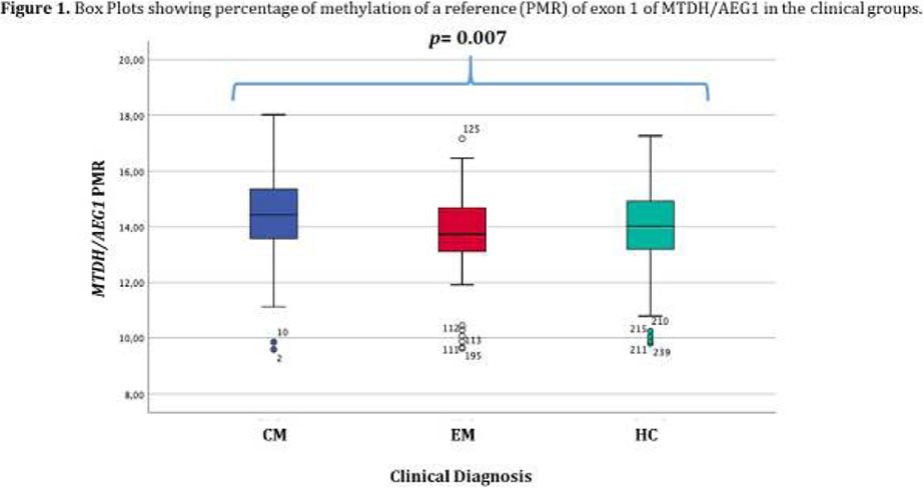
See text for description

**Table 1 (abstract P030).**
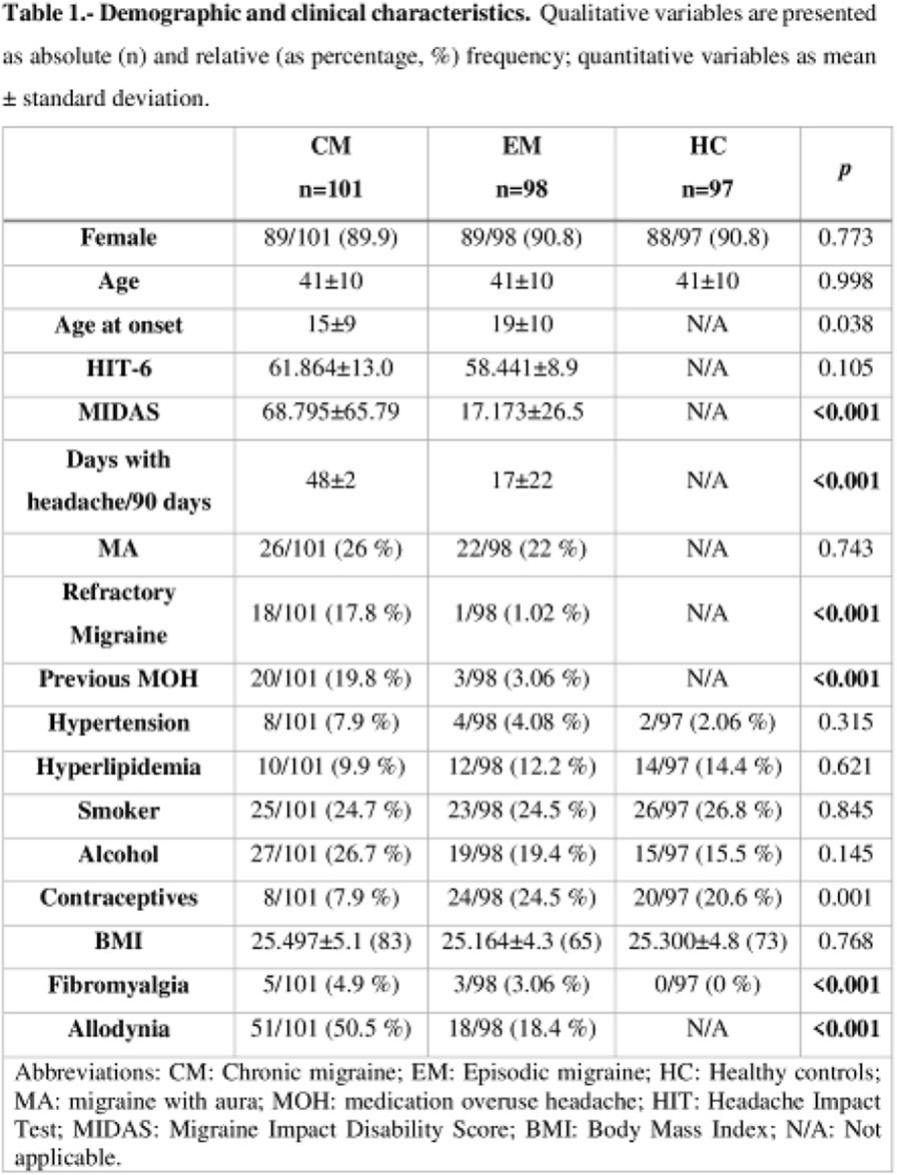
See text for description

## P031 White matter microstructural alterations in patients with persistent headache after COVID-19 infection: an exploratory study

### Á. Planchuelo-Gómez^1^, D. García-Azorín^2,3^, Á. L. Guerrero Peral^2,3,4^, S. Aja-Fernández^1^, M. Rodríguez^5^, R. Moro^5^, R. de Luis-García^1^

#### ^1^Universidad de Valladolid, Imaging Processing Laboratory, Valladolid, Spain; ^2^Hospital Clínico Universitario de Valladolid, Headache Unit, Valladolid, Spain; ^3^Institute for Biomedical Research (IBSAL), Salamanca, Spain; ^4^Universidad de Valladolid, Department of Medicine, Valladolid, Spain; ^5^Hospital Clínico Universitario de Valladolid, Department of Radiology, Valladolid, Spain

##### **Correspondence:** Á. Planchuelo-Gómez

Objective: To evaluate white matter alterations in patients with persistent headache after COVID-19 resolution.

Methods: Exploratory case-control study. High-resolution brain diffusion Magnetic Resonance Imaging data were acquired in patients with persistent headache after COVID-19 infection and healthy controls (HC). Tract-Based Spatial Statistics was used to compare fractional anisotropy (FA), axial diffusivity (AD), mean diffusivity (MD), radial diffusivity (RD) and the return-to-axial (RTAP), return-to-origin (RTOP) and return-to-plane probability (RTPP) between the groups. RTAP, RTOP and RTPP were obtained with a new approach called AMURA (https://www.lpi.tel.uva.es/AMURA). Significant results were considered with *p* < 0.05 (Family-Wise Error corrected) and region size larger than 30 mm^3^.

Results: Ten patients with persistent headache after COVID-19 (mean age: 53.8 ± 7.8 years; nine women) and 10 HC balanced for age and sex (mean age: 53.1 ± 7.0 years; nine women) were included in the study. Significant higher AD and lower RTPP values were found in patients with persistent headache compared to HC in five regions from the corona radiata, and the external and internal capsule. In the patients, significant lower RTPP values were identified in six additional areas from the same tracts and the superior longitudinal fasciculus. No additional changes were found.

Conclusions: White matter axonal alterations are present in patients with persistent headache after COVID-19 infection.

## P032 Evaluation of Headache Caused by the Use of Protective Equipment in Hospital Personnel during COVID-19 Pandemic

### E. Jafari^1^, M. Togha^1^, H. Kazemizadeh^1^, S. Haghighi^1^, S. Nasergivehchi^1^, M. Saatchi^1^, S. Ariyanfar^1,2^

#### ^1^Tehran University of Medical Sciences, Headache Department, Tehran, Iran; ^2^Shahid Beheshti University of Medical Scienes, Clinical Nutrition and Dietetics, Tehran, Iran

##### **Correspondence:** E. Jafari

Introduction: The coronavirus 19 disease (COVID-19) pandemic has created new conditions for medical staff, forcing them to use personal protective equipment (PPE) for an extended duration of time. Headache is one of the most commonly associated side effects of the use of such equipment among healthcare workers.

Method: In this cross-sectional study 243 healthcare workers in frontline of three referral hospitals for COVID 19 were evaluated in terms of having headache following the use of PPE. Further, its relationship with various factors including blood gas parameters was assessed.

Results: The average age of the participants was 36 ± 8 years, among whom75% were women. The prevalence of headache after using masks was 72.4%, with the N95 mask being the most common cause of headache (41%). Among patients with headache, 25.1% developed external pressure headache,22.2% migraine and 15.2% tension type headache. Female gender and increased heart rate were significantly associated with headache due to mask use (P-value: 0.024 and 0.001 respectively). The mean heart rate was 97.7±13.68 in participants with headache, compared to 65.8±35.63 in those without headache. No significant relationship was found between headache and venous blood gas parameters including oxygen and carbon dioxide partial pressure.

Conclusion: Headache due to PPE can decrease the efficiency of hospital staff performance. Hence, it is necessary to reduce the associated risk factors of this type of headache.

## P033 Headache during the COVID- 19 first wave: a survey on admissions' frequency, diagnosis and management in Emergency Department

### L. D'Acunto^1^, F. Pasquin^1^, A. Buoite Stella^1^, S. Olivo^1^, A. Granato^1^, F. Cominotto^2^, P. Manganotti^1^

#### ^1^University Hospital and Health Services of Trieste - ASUGI, University of Trieste, Clinical Unit of Neurology, Headache Centre, Department of Medicine, Surgery and Health Sciences, Trieste, Italy; ^2^University Hospital and Health Services of Trieste - ASUGI, University of Trieste, Emergency Department, Trieste, Italy

##### **Correspondence:** L. D'Acunto

Objective: The aim of this study was to analyze how the first Italian lockdown impacted on Emergency Department"s (ED) attendances due to headache as the principal presenting symptom in the tertiary-care University Hospital of Trieste.

Methods: We retrospectively evaluated frequency, features and management of ED attendances for headache during the lockdown period (from 8th March to the 31^st^ May 2020) comparing it with the pre lock down period (January-February 2020) and the first five months of 2019.

Results: A reduction of headache ED attendances was observed in the first five months of 2020 compared to the same period of 2019 (174 and 339 respectively; -49%). During the lockdown only a reduction of female ED access rate (p= 0.03) was found, while no significant variation was detected in repeaters prevalence, diagnostic assessment and acute treatment. The ratio of Not Otherwise Specified (NOS), Secondary and Primary Headaches remained unchanged during the lockdown period, in comparison to control periods, being NOS headache the prevalent discharge diagnosis (48.4%). Primary and secondary headache represented the 21.0% and 30.6 % respectively of the sample.

Conclusions: COVID-19 pandemic impacted the number of ED attendances for headache but not their management, discharge diagnosis distribution and rate of repeaters. During this period probably a portion of secondary dangerous headaches did not arrive in ED.

## P034 Clinical characteristics of headache after vaccination against COVID-19 (Coronavirus SARS-CoV-2) with the BNT162b2 mRNA vaccine: a prospective multicentre observational cohort study

### C. Göbel^1,2^, A. Heinze^2^, S. Karstedt^1,2^, M. Morscheck^2^, L. Tashiro^2^, A. Cirkel^1,2^, Q. Hamid^3^, R. Halwani^3^, M. H. Temsah^4^, M. Ziemann^5^, S. Görg^5^, T. Münte^1^, H. Göbel^2^

#### ^1^University Hospital Schleswig-Holstein, Department of Neurology, Lübeck, Germany; ^2^Kiel Headache and Pain Centre, Kiel, Germany; ^3^University of Sharjah, College of Medicine, Sharjah, United Arab Emirates; ^4^King Saud University, College of Medicine, Riyadh, Saudi Arabia; ^5^University Hospital Schleswig-Holstein, Institute of Transfusion Medicine, Lübeck, Germany

##### **Correspondence:** C. Göbel

Background: The novel coronavirus SARS-CoV-2 causes the infectious disease Covid-19. Newly developed mRNA vaccines can prevent the spread of the virus. Headache is the most common neurological symptom in over 50% of those vaccinated. Detailed information about the clinical characteristics of this new form of headache has not yet been described. The aim of the study is to examine in detail the clinical characteristics of headaches occurring after vaccination against Covid-19 with the BNT162b2 mRNA Covid-19 vaccine for the first time.

Methods: In a prospective multicenter observational cohort study data on the clinical features and corresponding variables were recorded using a standardized online questionnaire. The questionnaire was circulated to 12,000 residential care homes of the elderly as well as tertiary university hospitals in Germany and the United Arab Emirates.

Findings: A total of 2349 participants reported headaches after vaccination with the BNT162b2 mRNA Covid-19 vaccine. Headaches occur an average of 18.0 ± 27.0 hours after vaccination and last an average duration of 14.2 ± 21.3 hours. Only 9.7% of those affected also report headaches resulting from previous vaccinations. In 66.6% of the participants headache occurs as a single episode. 73.1% of participants indicate a bilateral location.

Interpretation: Headaches after Covid-19 vaccination show concise clinical characteristics. The constellation of accompanying symptoms together with the temporal and spatial headache characteristics delimit a distinctive headache phenotype.

## P035 Resting-state functional alterations in patients with persistent headache after COVID-19 infection: an exploratory study

### Á. Planchuelo-Gómez^1^, D. García-Azorín^2,3^, Á. L. Guerrero Peral^2,3,4^, S. Aja-Fernández^1^, M. Rodríguez^5^, R. Moro^5^, R. de Luis-García^1^

#### ^1^Universidad de Valladolid, Imaging Processing Laboratory, Valladolid, Spain; ^2^Hospital Clínico Universitario de Valladolid, Headache Unit, Valladolid, Spain; ^3^Institute for Biomedical Research (IBSAL), Salamanca, Spain; ^4^Universidad de Valladolid, Department of Medicine, Valladolid, Spain; ^5^Hospital Clínico Universitario de Valladolid, Department of Radiology, Valladolid, Spain

##### **Correspondence:** Á. Planchuelo-Gómez

Objective: To evaluate resting-state functional alterations in patients with persistent headache after COVID-19 resolution.

Methods: Exploratory case-control study. High-resolution brain resting-state functional Magnetic Resonance Imaging data were acquired in patients with persistent headache after COVID-19 infection and healthy controls (HC). CONN toolbox (version 17) was employed to assess the resting-state functional connectivity between 84 cortical and subcortical gray matter regions of interest. Significant results were considered with *p* < 0.05 (Family Discovery Rate and seed-level corrected).

Results: Ten patients with persistent headache after COVID-19 (mean age: 53.8 ± 7.8 years; nine women) and 10 HC balanced for age and sex (mean age: 51.9 ± 6.6 years; nine women) were included in the study. Statistically significant higher functional connectivity was observed in the patients with persistent headache compared to HC in 10 connections. These connections were composed of an occipital region and another region that included the isthmus cingulate gyrus, a frontal or a parietal area. In the patients, significant lower functional connectivity was found in 12 connections between the cingulate and hippocampal gyri, parietal, temporal and frontal regions.

Conclusions: Patients with persistent headache after COVID-19 infection present strengthened functional connectivity with occipital regions and weakened functional connectivity between frontal, temporal and parietal regions.

## P036 Facemask headache: a new nosographic entity among healthcare professionals in COVID-19 era

### L. Rapisarda^1^, F. Fortunato^1^, A. De Martino^1^, O. Marsico^1^, G. Demonte^1^, A. Augimeri^2^, A. Labate^1^, A. Gambardella^1^, M. Trimboli^1^

#### ^1^AOU Mater Domini - Magna Graecia University, Department of Medical and Surgical Sciences, Institute of Neurology, Catanzaro, Italy; ^2^Biotecnomed S.CaR.L., Catanzaro, Italy

##### **Correspondence:** L. Rapisarda, M. Trimboli

Background: Mild neurological disturbances such as headache have been related to the extensive utilization of facemask. This study aims to examine headache variations related to the intensive utilization of facemask among a cohort of healthcare professionals in a setting of low-medium risk of exposure to SARS-CoV-2.

Methods: This is a cross-sectional study amongst healthcare providers from different hospital and clinics in Italy. Each participant completed a specifically-designed self-administered questionnaire. Headache features and outcome measures" change from baseline were evaluated over a four-months period, in which wearing facemask has become mandatory for Italian healthcare workers.

Results: A total of 400 healthcare providers completed the questionnaire, 383 of them met the inclusion criteria. The majority were doctors, with a mean age of 33.4±9.2 years old. Amongst 166/383 subjects, who were headache free at baseline, 44 (26.5%) developed *de novo* headache. Furthermore, 217/383 reported a previous diagnosis of primary headache disorder: 137 were affected by migraine and 80 had tension-type headache. A proportion (31.3%) of these primary headache sufferers experienced worsening of their pre-existing headache disorder.

Conclusions: Our data showed the appearance of *de novo* associated facemask headache in previous headache-free subjects and an exacerbation of pre-existing primary headache disorders, mostly experienced by people with migraine disease.

**Fig. 1 (abstract P036).**
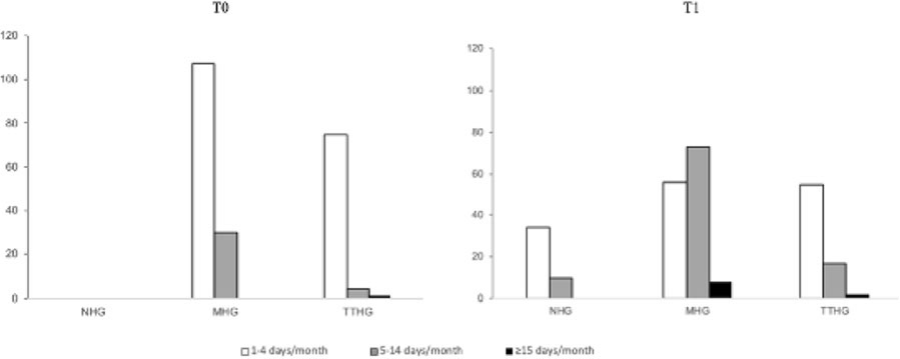
See text for description

**Fig. 2 (abstract P036).**
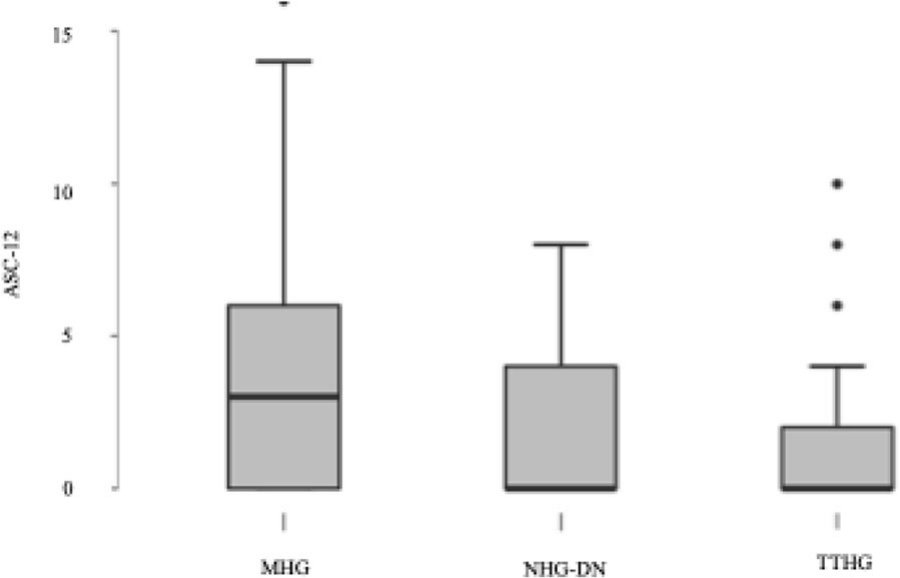
See text for description

## P039 Clinical predictors of persistent post-COVID-19 headache

### D. García-Azorín^1^, A. Gonzalez-Martinez^2^, Á. Sierra Mencía^1^, J. Trigo López^1^, A. Alberdi^3^, M. Blanco^3^, I. Calcerrada^3^, A. Cornejo^3^, M. Cubero^3^, A. Gil^3^, C. García^3^, A. G. Lozano^3^, C. Martínez Badillo^3^, C. Montilla^3^, M. Mora^3^, G. Nuñez^3^, M. Paniagua^3^, C. Perez^3^, M. Rojas^3^, M. Ruiz^3^, L. Sierra^3^, L. Hurtado^3^, Á. L. Guerrero Peral^1^

#### ^1^Hospital Clinico Universitario de Valladolid, Headache Unit, Department of Neurology, Valladolid, Spain; ^2^Hospital Universitario de la Princesa & Instituto de Investigación Sanitaria de la Princesa, Headache Unit, Neurology, Madrid, Spain; ^3^Gerencia de Atención Primaria Valladolid Este, Valladolid, Spain

##### **Correspondence:** D. García-Azorín

Objective: We aimed to evaluate which demographic and clinical variables were associated with a more prolonged duration of headache attributed to Covid-19.

Methods: We conducted prospective study including all hospitalized patients during the first wave of the pandemic, followed-up until April 2021. We used log-rank test to evaluate the association of 48 different variables with the duration of the headache and created a multivariable Cox"s proportional hazard model. We used False Discovery Rate to adjust for multiple comparisons.

Results: 138/576 (23.9%) patients had headache, with a median duration of 30 days (inter-quartile range 19-66). In the univariate analysis, the variables that were associated with a more prolonged duration of the headache were prior history of immunosuppression, unilateral pain and worse baseline situation. The variables that were associated with a shorter duration of the headache included worsening by physical activity, presence of dyspnea, prior history of tension-type headache, higher intensity of the headache, pressing quality, bilateral pain and photophobia. In the multivariate analysis, worsening of the headache by physical activity (Hazard ratio (HR): 0.48; 95% confidence intervals (CI): 0.30-0.78) and prior history of immunosuppression (HR: 2.9; 95% CI: 1.02-8.22) remained statistically significant.

Conclusion: Long-term duration of headache might be related with individual predisposition and the clinical phenotype of the headache.

## P040 Persistence of headache post-COVID-19: A multicentric prospective study of 9-months follow-up

### D. García-Azorín^1^, A. Layos-Romero^2^, J. Porta-Etessam^3^, J. A. Membrilla^4^, E. Caronna^5^, A. Gonzalez-Martinez^6^, Á. Sierra Mencía^1^, T. Segura^2^, N. González García^3^, J. Díaz de Terán Velasco^4^, V. J. Gallardo^5^, A. B. Gago Veiga^6^, A. Ballvé^5^, J. Trigo López^1^, M. Sastre-Real^4^, A. Llauradó^5^, A. Cornejo^7^, I. de Lorenzo^4^, Á. L. Guerrero Peral^1^, P. Pozo-Rosich^5^

#### ^1^Hospital Clinico Universitario de Valladolid, Headache Unit, Department of Neurology, Valladolid, Spain; ^2^Department of Neurology. Hospital General de Albacete. Albacete, Spain., Albacete, Spain; ^3^Headache Unit, Department of Neurology. Hospital San Carlos, Madrid., Spain. Instituto de Investigación Sanitaria San Carlos (IdISSC), Madrid, Spain.Universidad Complutense de Madrid, Madrid, Spain, Madrid, Spain; ^4^La Paz Institute for Health Research (IdiPAZ), Madrid, Spain; ^5^Headache Unit, Neurology Department, Hospital Universitari Vall d’Hebron, Barcelona, Spain. Headache Research Group, Vall d’Hebron Research Institute, Universitat Autónoma de Barcelona, Barcelona Spain., Barcelona, Spain; ^6^Hospital Universitario de la Princesa & Instituto de Investigación Sanitaria de la Princesa, Headache Unit, Neurology, Madrid, Spain; ^7^Gerencia de Atención Primaria Valladolid Este, Valladolid, Spain

##### **Correspondence:** D. García-Azorín

Objective: Headache is within the most frequent symptoms of coronavirus disease 2019 (COVID-19). We aim to evaluate the long-term duration of headache attributed to COVID-19 in six cohorts that studied patients with headache during the first wave of the pandemic.

Methods: We conducted an observational prospective study, including patients from six different centers that were studied during the first wave of the pandemic and completed at least 9 months of follow-up since headache onset. All six cohorts have already published data regarding the acute phase of the headache. We harmonized the databases and analyzed all common data elements. We present the data as percentage or median and inter-quartile range [IQR].

Results: We included 905 patients who presented headache during the acute phase of COVID-19, aged 51 [IQR 45-65], 66.5% female, with prior history of headache 52.7%. Patients had pneumonia in 47.2% cases and were hospitalized in 50.5% cases. Headache onset was after one [IQR 0-3] days after the onset of COVID-19 symptoms. The median duration of headache was 14 [6-39] days. Headache persisted after two months in 21.4% cases, after three months in 18.9%, after six months in 17.0% and after nine months in 15.9%.

Conclusion: The median duration of headache attributed to COVID-19 is two weeks, but in approximately a fifth of patients it becomes persistent and follows a chronic pattern.

## P041 Clinical characteristics of headache after vaccination against COVID-19 (Coronavirus SARS-CoV-2) with the COVID-19 Vaccine AstraZeneca: a prospective multicentre observational cohort study

### C. Göbel^1,2^, A. Heinze^2^, S. Karstedt^1,2^, M. Morscheck^2^, L. Tashiro^2^, A. Cirkel^1,2^, Q. Hamid^3^, R. Halwani^3^, M. H. Temsah^4^, M. Ziemann^5^, S. Görg^5^, T. Münte^1^

#### ^1^University Hospital Schleswig-Holstein, Department of Neurology, Lübeck, Germany; ^2^Kiel Headache and Pain Centre, Kiel, Germany; ^3^University of Sharjah, College of Medicine, Sharjah, United Arab Emirates; ^4^King Saud University, College of Medicine, Riyadh, Saudi Arabia; ^5^University Hospital Schleswig-Holstein, Institute of Transfusion Medicine, Lübeck, Germany

##### **Correspondence:** C. Göbel

Background: Headache at a frequency of 52.6 % is the most common neurological symptoms in those vaccinated with the COVID-19 Vaccine AstraZeneca. The aim of the study is to examine in detail the clinical characteristics of headaches occurring after vaccination against Covid-19 with the COVID-19 Vaccine AstraZeneca for the first time.

Methods: The study is a continuous prospective multicenter observational cohort study taking place during the Covid-19 vaccination campaign. With a publicly available online questionnaire, specific aspects of the headache phenotype and related variables are collected. A total of 12,000 residential care homes in Germany were contacted. In addition, the departments responsible for organizing the vaccinations at university hospitals in Germany and the United Arab Emirates were contacted.

Findings: A total of 2464 participants reported headaches after vaccination with the COVID-19 Vaccine AstraZeneca. The mean age of the participants was 39.8 ± 12.7 years. 92.6% stated that they had not experienced any headaches with any other vaccination. Headaches occur an average of 14.5 ± 21.6 hours after vaccination and last an average duration of 16.3 ± 30.0 hours. In 67.4% of the participants headache occurs as a single episode. 75.8% of participants indicate a bilateral location.

Interpretation: Headaches after Covid-19 vaccination with the COVID-19 Vaccine AstraZeneca show concise clinical characteristics.

## P042 Impact of personal protective equipment use in migraine patients during the COVID-19 pandemic

### R. Oliveira^1^, M. Plácido^2^, L. Pereira^3^, S. Machado^4^, E. Parreira^4^, R. Gil-Gouveia^1^

#### ^1^Hospital da Luz Lisboa, Neurology, Lisbon, Portugal; ^2^MiGRA Portugal, Lisbon, Portugal; ^3^Hospital Garcia de Orta, Lisbon, Portugal; ^4^Hospital Professor Doutor Fernando da Fonseca, Lisbon, Portugal

##### **Correspondence:** R. Oliveira

Objective: To analyze the impact of personal protective equipment (PPE) use in migraine patients.

Methods: National web-based survey between September-December 2020 to explore the occurrence of PPE-related headaches in the general population.

Results: Of 5064 participants, 2547(50.3%) had migraine, 2412(94.7%) were women, average age of 36.7±10.2 years. Surgical and cloth masks were the most common PPE type used. Forty-four percent (1118) reported *de novo* headaches(dnH) after the onset of the COVID-19 pandemic, most participants (888, 79.4%) attributing it to PPE use. Comparing to previous headaches, dnH group had less photophobia (94.5%vs71.3%), nausea (92.3%vs55.9%), and aggravation by routines (90.8%vs57.8%). Participants with dnH wore PPE for longer periods of time (7 ± 3h20 vs 6.1 ± 3h30 min per day, P<0.001). Longer mean duration of PPE use (OR of 1.1, 95% CI 1-1.2) was predictor of developing dnH in multivariate analysis. Most migraine patients reported aggravation of pre-existing headaches with PPE use with more patients fulfilling chronic migraine criteria (5.9% vs 14.4%). Duration of PPE usage was also determinant for exacerbation of previous headaches (7 ± 2h30 vs 6 ± 3h10 min per day, P<0.05).

Conclusions: Almost all participants with migraine reported worse outcomes, and almost half developed dnH with PPE use, which had less migrainous features than previous headaches. Duration of PPE usage was the strongest predictor of both dnH and exacerbation of previous headaches.

## P043 Classification of migraine subtypes and its characteristics by latent class analysis: a population-based study

### W. Lee^1^, M. K. Chu^1^, I. K. Min^1^

#### ^1^Yonsei University, Neurology, Seoul, South Korea

##### **Correspondence:** M. K. Chu

Background and objectives: Identifying the natural subgroups of migraine may facilitate biological and genetic characterization. This study is aimed to investigate the natural subgroups of migraine based on the ICHD-3 criteria using latent class analysis modeling.

Methods: We used the data of the Korea Sleep-Headache Study which was a nation-wide population-based survey on headache and sleep. Optimal number of subgroups was determined by Akaike information criterion (AIC) and Bayesian information criterion (BIC).

Results: Of total 2501 participants, 125 participants had migraine. AIC was lowest in four latent class (LC) models while BIC was lowest in two LC models. We selected the three LC models in the present study. 53, 50, and 22 participants with migraine belonged to LC1, LC2 and LC3, respectively. LC1 had intermediate headache frequency (median and interquartile range, 1.0 [0.3-4.0] attacks per month), and more photophobia (79.3%) and phonophobia (100.0%). LC2 showed infrequent headache frequency (0.5 [0.3-2.0] attacks per month), unilateral headache (100.0%) and no aggravation by routine physical activity (0.0%). LC3 had more frequent headache (2.0 [0.5-7.0] attacks per month), and more aggravation by routine physical activity (77.3%).

Conclusions: Three subtypes of migraine were identified in LCA modeling. These subtypes showed difference in clinical features.

## P044 Pediatric headaches in Benin a sub-Saharan African country: Epidemiology and Burden

### M. Agbetou^1^, W. Tchuenga Fokom^1^, N. Dovoedo^1^, T. Adoukonou^1^

#### ^1^University of Parakou, Neurology, Parakou, Benin

##### **Correspondence:** M. Agbetou

Objective: To study the epidemiology and burden of headache among children and adolescents in Benin

Method: A cross-sectional study with descriptive and analytical purposes was performed from April to June 2019 in 16 primary and secondary schools of the North and South in Republic of Benin. The sampling technique was a 3-stage random survey. We included 2319 children and adolescents respectively aged 6 to 11 years enrolled in an elementary school class and 12 to 17 years enrolled in a secondary school class. Their assent and the consent or non-opposition of the parents or guardian have been obtained. The headache attributed restriction disability, social handicap and impaired participation (HARDSHIP) questionnaire adapted for children had been used; analysis was performed with IBM SPSS Statistics 21 and STATA MP14 software

Results: The overall prevalence of headache was 88.54% CI [87.23-89.84] with 89.08% among children and 88.06% among adolescent. Migraine prevalence was 14.95%; tension headache 7.58%; probable headache drug abuse 1.53%. School absenteeism (31.79%), disruption of parents" activities (30,33%), lack of concentration (79.68%) and difficulty to study were the most common impacts and disabilities. Factors associated with headache were area of residence (p=0.000) and level of study (p=0.011) in children; gender (p=0.021) and ethnicity (p=0.043) in adolescents.

Conclusion: Headache is frequent among school-age children and adolescents in Benin with a social impact.

## P045 Atypical auras in pediatric migraine: Clinical series and pathophysiological correlations

### G. M. Nocera^1^, V. Raieli^2^

#### ^1^Università degli studi di Palermo, Neuropsichiatria Infantile, Palermo, Italy; ^2^P.O. Di Cristina - ARNAS Civico Palermo, Palermo, Italy

##### **Correspondence:** G. M. Nocera

Objectives: Cortical Spreading Depression (CSD) has been suggested as the most plausible underlying pathophysiological mechanism of migraine Aura. However, recent reports raised doubt against the concept that CSD could account for all presentations of migraine aura. Here, we show a series of atypical paediatric aura hardly explainable by the CSD, suggesting partly different pathophysiological mechanisms.

Methods: We selected retrospectively our patients who had atypical aura on the basis of the following criteria:

1. the spreading wave appears to be not related to the CSD model
2. the chronological sequence and *homunculus* are not respected by the sequence and characters of aura symptoms
3. time intervals between symptoms onset not justified by CSD theory
4. atypical clinical symptoms not accountable by CSD
5. atypical correlation with pain onset and pain side

Results: We collected 15 cases (5 M/10 F, range age 9-16 ys). All subjects underwent EEG and Brain MRI. They were divided according to the criteria described above: 4 subjects met criterion 1, 5 the second, 5 the third, 5 the fourth, 1 the fifth, some children met multiple criteria.

Conclusion: Our cases show that the current CSD theory cannot fully explain the modalities of the aura presentation in some subjects. Therefore, some aspects need further investigation and reassessment on the basis of clinical practice. Moreover, we underline how accurate exploration of migraine aura can provide useful insight on pathophysiological aspects.

## P046 Recurrent headache in Internet-addicted Central Siberia urban adolescents: a school-based study

### S. Tereshchenko^1^, L. Evert^1^, N. Gorbacheva^1^, M. Shubina^1^

#### ^1^Scienific Research Institute of Medical Problems of the North Department of child's physical and mental health, Kranyarsk, Russian Federation

##### **Correspondence:** S. Tereshchenko

Psychosomatic symptoms prevalence and types in Internet-addicted (IA) adolescents are not studied well. We aimed to investigate IA comorbidity with recurrent headache in Central Siberia urban adolescents.

Methods: 2950 urban Siberian (Krasnoyarsk) school-based adolescents (aged 12-18; boys/girl ratio 1348/1602) were tested with Chen Internet Addiction Scale (CIAS). Based on the CIAS, score Internet users were categorized into three groups: adaptive Internet users (AIU-1) (scoring 27–42); maladaptive Internet users (MIU) (scoring 43–64); and pathological Internet users (PIU) (scoring ≥65). Adolescents were also asked about headache presence/frequency and according to answer were divided into three groups: (1) No headache group, (2) frequent episodic headache with episodes frequency 1-15 per month, and (3) chronic headache with episodes frequency >15 per month. A Chi-square test was used.

Results: The prevalence of AIU, MIU, and PIU were 50.4%, 42.8%, and 6.8%, respectively. Significant positive associations were detected between CIAS scores and headache, especially for the chronic headache group (р1-2=0,0047, р1-3<0,0001, р2-3=0,0008, where 1-AIU, 2-MIU, 3-PIU; Fig. 1).

Conclusion: The Internet addiction group have significantly higher headache frequency that may be explained by the presence of common risk factors such as emotional stress, depression, and anxiety.

The reported study was funded by RFBR according to the research project № 18-29-22032.

**Fig. 1 (abstract P046).**
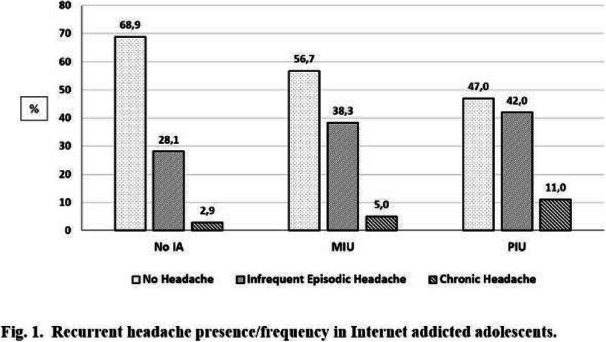
See text for description

## P047 Dairy intake and odds of peadiatric migraine: A case control study

### S. Ariyanfar^1,2^, S. Razeghi Jahromi^1^, N. Rezaeimanesh^1^, M. Togha^2^, Z. Ghorbani^2,3^

#### ^1^Shahid Beheshti University of Medical Scienes, Clinical Nutrition, Tehran, Iran; ^2^Tehran University of Medical Sciences, Headache Department, Tehran, Iran; ^3^Guilan University of Medical Sciences, Department of Cardiology, Guilan, Iran

##### **Correspondence:** S. Ariyanfar

Objective: Migraine is recognized as a disease with various pathophysiologic pathways, which are not fully understood. This study was designed following the relation between dairy intake and various chronic conditions in children and also the paucity of data on the probable role of dairy intake on pediatrics" odds of migraine.

Methods: The present study was a population based case – control design and included 290 children. Definite diagnosis of migraine was performed by a neurologist, with respect to the 2018 international classification of headache disorder 3(ICHD3) criteria. The usual dietary intake of participants was evaluated, using a validated semi-quantitative food frequency questionnaire (FFQ).

Result: In the second regression model, odds of migraine were 48% (OR:0.52;95%CI:0.27-1.00) diminished in the second tertile, and 53%(OR:0.47;95%CI:0.24-0.92) in the third tertile of low-fat dairy intake (P-trend:0.03). In fully adjusted model, the migraine ORs were 0.48 (95% CI:0.240.95) in the second tertile and 0.46(95%CI:0.21-0.96) in the third tertile (P-trend:0.04), respectively. Children with more high-fat dairy intake, also consumed higher amounts of energy, pastries, simple sugar, and hydrogenated oil(P<0.05).

Conclusion: Greater amount of low-fat dairy intake may attenuates the odds of having migraine attacks in pediatrics, who might be at risk of headache. It can be attributed to the micronutrient and bioactive content of these dietary components.

## P048 Multi-omics to predict changes during cold pressor test during interictal phase

### L. J. A. Kogelman^1^, M. Ernst^2^, G. Mazzoni^3^, J. Courraud^2^, L. P. Lundgren^2^, S. S. Laursen^2^, A. Cohen^2^, J. Olesen^1^, T. F. Hansen^1^

#### ^1^Copehagen University Hospital, Danish Headache Center, Glostrup, Denmark; ^2^Statens Serum Institut, Danish Center for Neonatal Screening, Copenhagen, Denmark; ^3^Copenhagen University, Novo Nordic Foundation Center for Protein Research, Copenhagen, Denmark

##### **Correspondence:** T. F. Hansen

Background: Molecular mechanisms of pain are complex and difficult to entangle, but important to understand to treat pain disorders. The cold pressor test (CPT) is used as pain provocation test in pain research. We hypothesize, that performing multi-omic analyses during CPT gives the opportunity to home in on molecular mechanisms involved.

Methods: Twenty-two females diagnosed with migraine were phenotypically assessed before and after a CPT, and blood samples were taken interictal. RNA-Sequencing, steroid profiling and untargeted metabolomics were performed. Each "omic level was analyzed separately at both single-feature and systems-level (e.g. principal component and partial least squares regression analysis) and all "omic levels were combined using an integrative multi-omics approach, all using the paired-sample design.

Results: We showed that unsupervised methods were not able to discriminate time points, while supervised clustering did significantly distinguish time points using metabolomics and/or transcriptomic data, but not using conventional physiological measures. Transcriptomic and metabolomic data revealed at feature-, systems- and integrative- level biologically relevant processes involved during CPT, e.g. lipid metabolism and stress response.

Conclusion: Multi-omics strategies should be exploited in pain research to gain knowledge on the biological mechanisms involved in pain.

## P049 Combined Oral Contraceptive was associated to protection for severe allodynia

### A. Vitali da Silva^1^, L. Hessmann Gonzalez^1^, R. Schmidt Alves Ferreira Galvão^1^, I. Gaspar Vuolo^1^, R. Célia Poli Frederico^1^, V. Aparecida Bello^1^, S. H. dos Santos Gajardoni Farges^1^

#### ^1^Pontifícia Universidade Católica do Paraná, Escola de Medicina, Londrina, Brazil

##### **Correspondence:** A. Vitali da Silva

Objective: To assess the effect of combined hormonal contraceptive (CHC) use on the prevalence of severe allodynia in women with migraine.

Methods: study composed by women with migraine with or without aura, who were not pregnant, lactating or in menopause. The research was developed through a digital platform. Clinical features and contraceptive method were registered. In sequence, the participants answered to the validated self-applicable questionnaires: Migraine Disability Assessment, Allodynia Symptom Checklist, Generalized Anxiety Disorder and Beck Depression Inventory. To determine variables associated with severe allodynia, 2 binary logistic regression models were used, the first which included all forms of exposure to estrogen and the second included oral CHC.

Results: 440 women were included at the study. An amount of 176 women were taking estrogen by taking CHC, and 164 of these were taking it by oral pills. Severe allodynia was identified in 126 participants (29.2%). In multivariate analysis, severe allodynia was independently associated with the presence of aura (OR=2.57 IC95%1.43-4.61; p=0.002), depression (OR= 1.67 IC95%0.97-2.86; p=0.062), migraine-related disability (OR=3.08 IC95%11.82-5.2; p<0.001), and estrogen in the form of oral CHC (OR=0.56 IC95%0,33-0.95; p=0.030). Age, BMI, smoking, menstrual migraine, and anxiety were not related to the presence of severe allodynia.

Conclusion: the oral CHC was shown to be a protective factor to severe allodynia.

**Table 1 (abstract P049).**
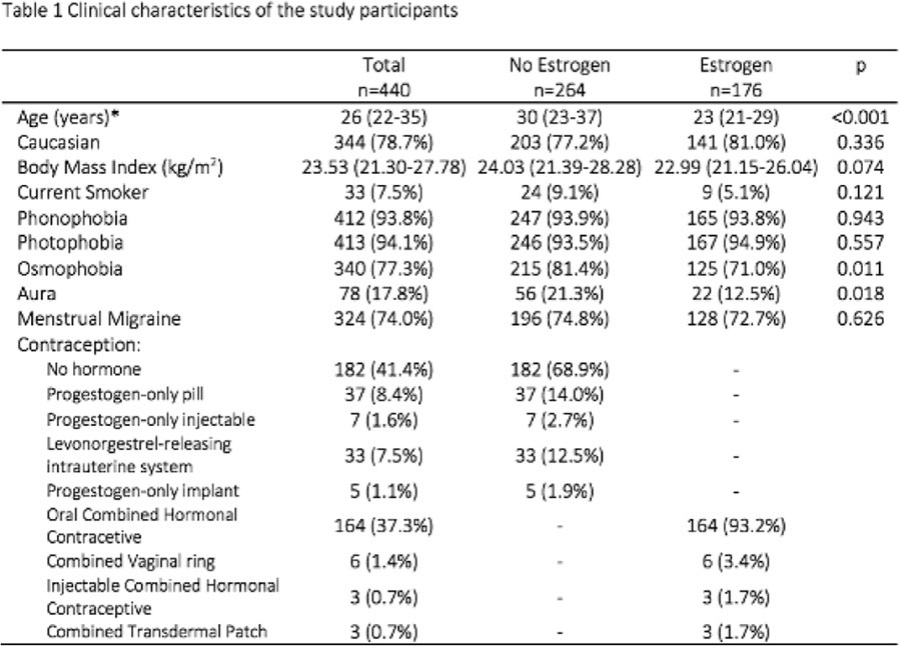
See text for description

**Table 2 (abstract P049).**
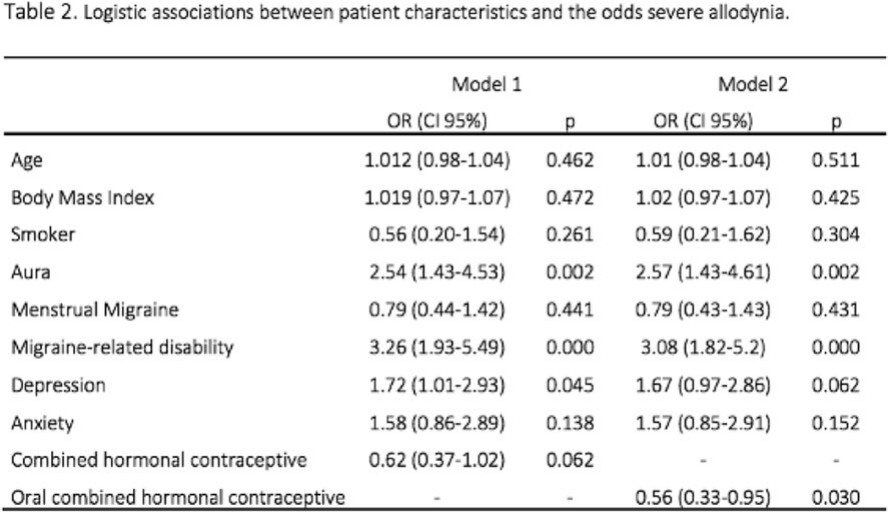
See text for description

## P050 Family-based exome sequencing disclose associated genes in primary headaches

### A. Dias^1,2^, J. Pereira Monteiro^1^, J. Sequeiros^1,2^, M. Paço^3^, T. Pinho^3^, A. Sousa^1,2^, C. Lemos^1,2^, M. Alves-Ferreira^1,2^

#### ^1^i3S – Instituto de Investigação e Inovação em Saúde, UnIGENe, Porto, Portugal; ^2^ICBAS - Instituto Ciências Biomédicas Abel Salazar, Universidade do Porto, Porto, Portugal; ^3^IINFACTS, CESPU – Instituto de Investigação e Formação Avançada em Ciências e Tecnologias da Saúde, Gandra, Portugal

##### **Correspondence:** A. Dias

During the last 20 years, our group clinically characterized more than thousand individuals with migraine (with/without aura – MA/MO) or cluster headache (CH). We found several genetic variants involved in vascular component, trigeminal nociceptive plasticity, neurogenic inflammation and in neurotransmitters release. Despite all advances, genetic basis of primary headaches remains unknown. Whole-exome sequencing (WES) is a powerful approach to explore coding regions, particularly low-frequency variants.

Objective: To perform a WES focusing on variants with a predicted high impact to study transmission intra- and inter-families with migraine and CH.

Methods: We performed a WES in 20 DNA samples from 3 families.

Results: We found common and rare variants in genes already associated with migraine subtypes as CACNA1A and PRRT2 and in new genes that may open new pathways of study.

Conclusions: These preliminary results need to be further explored and variants interactions studied to deepen the pathophysiological pathways, leading to the development of more effective and better-tolerated therapeutics.

Acknowledgments: This work was funded by IINFACTS, CESPU and FEDER Regional funds (COMPETE 2020 - Operacional Programme for Competitiveness and Internationalisation (POCI), Portugal 2020) and through FCT - Fundação para a Ciência e a Tecnologia/Ministério da Ciência, Tecnologia e Ensino Superior in the framework of the project POCI-01-0145-FEDER- 029486 (PTDC/MEC-NEU/29486/2017).

## P051 Diagnosis and Classification of Headache Associated with Sexual Activity Using a Composite Algorithm

### P. T. Lin^1^, Y. F. Wang^1^, J. L. Fuh^1^, J. F. Lirng^1^, Y. H. Ling^1^, S. P. Chen^1^, S. J. Wang^1^

#### ^1^Taipei Veterans General Hospital, Department of Neurology, Taipei City, Taiwan

##### **Correspondence:** P. T. Lin

Objective: To differentiate primary headache associated with sexual activity (HSA) from other devastating secondary causes.

Methods: In the prospective cohort, we recruited consecutive patients with at least 2 attacks of HSA from the headache clinics or emergency department of a national medical center. Detailed interview, neurological examination, and serial thorough neuroimaging including brain MRI/ magnetic resonance angiography scans were performed on registration and during follow-ups.

Results: Overall, 245 patients with HSA were enrolled. Our clinic-radiologic composite algorithm diagnosed and classified all patients into four groups, including 38 (15.5%) with primary HSA, 174 (71.0%) with reversible cerebral vasoconstriction syndrome (RCVS), 26 (10.6%) with probable RCVS, and 7 (2.9%) with other secondary causes (aneurysmal subarachnoid hemorrhage (n=4), right internal carotid artery dissection (n=1), Moyamoya disease (n=1), and meningioma with hemorrhage (n=1)). These four groups shared similar clinical profiles, except 26% of the patients with primary HSA had a 3 times greater chance of running a chronic course (≥ 1 year) than patients with RCVS. Of note, the RCVS2 score could not differentiate RCVS from other groups.

Conclusion: Our composite clinic-radiological diagnostic algorithm successfully classified repeated HSA, which was predominantly secondary and of vascular origin, and predicted the prognosis. Primary HSA and RCVS may be of the same disease spectrum.

**Fig. 1 (abstract P051).**
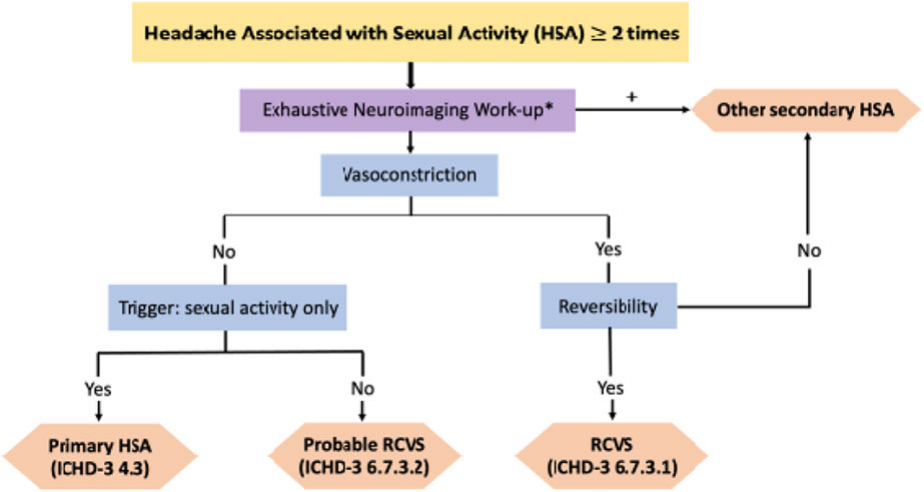
See text for description

## P052 Physical Activity and Migraine According to Aura Symptoms in the ELSA-Brasil cohort: A cross-sectional study

### A. Oliveira^1,2,3^, M. Peres^2,3^, J. Mercante^1,2,3^, M. D. C. Molina^4,5^, P. Lotufo^1^, I. Benseñor^1^, A. Goulart^1^

#### ^1^Universidade de São Paulo, Center for Clinical and Epidemiological Research, São Paulo, Brazil; ^2^Hospital Israelita Albert Einstein, Instituto do Cérebro, São Paulo, Brazil; ^3^Universidade de São Paulo, Instituto de Psiquiatria, São Paulo, Brazil; ^4^Universidade Federal de Ouro Preto, Nutrição e Saúde, Ouro Preto, Brazil; ^5^Universidade Federal do Espírito Santo, Vitória, Brazil

##### **Correspondence:** A. Oliveira

Objective: To evaluate the associations between physical activity in the leisure-time (LTPA) and commuting time (CPA) domains, migraine with aura (MA), and migraine without aura (MO) in the ELSA-Brasil cohort.

Methods: In this cross-sectional analysis, logistic regression models computed the odds ratio (OR) for the associations between LTPA and CPA levels across headache subtypes: no headaches (ref.), MA, MO, and other headaches. The adjusted models were controlled for potential confounders as sociodemographic, BMI, and migraine prophylaxis.

Results: From 4,717 participants (53.6 % women; age: 51.7 SD ±9 years), LTPA was associated with reduced MA [OR: 0.65 (0.48-0.88) p < 0.01] and MO [OR: 0.67 (0.53-0.84), p < 0.001]. In men, vigorous LTPA was associated with reduced MA [OR: 0.65 (0.44-0.94), p < 0.05] and MO [OR: 0.38 (0.20-0.73), p < 0.01]. For women, moderate LTPA was associated with reduced MA [OR: 0.48 (0.24-0.93)]. Vigorous, but not moderate LTPA, was associated with reduced MO [OR: 0.69 (0.48-0.98), p < 0.05]. In the CPA domain, insufficiently active associated with reduced MA [OR: 0.56 (0.32-0.99), p < 0.05]. There was a strong and inverse linear trend for the association between LTPA and MO frequency (p-trend < 0.001), but not MA.

Conclusion: LTPA was associated with reduced migraine, regardless of aura symptoms. In the CPA domain, only insufficient activity level associated with reduced MA. LTPA intensity diverges according to aura, sex, and headache frequency.

## P053 Characterization of erenumab and rimegepant affinity towards calcitonin gene-related peptide and amylin-1 receptors: Possible explanation for constipation by erenumab

### S. H. La Cour^1^, K. Juhler^1^, L. J. A. Kogelman^1^, J. Olesen^1^, D. Klærke^2^, D. M. Kristensen^1,3^, I. Jansen-Olesen^1^

#### ^1^Rigshospitalet Glostrup, Department of Neurology, Danish Headache Center, Glostrup, Denmark; ^2^Copenhagen University, Department of Veterinary and animal sciences, Faculty of Health and Medical Sciences, Frederiksberg C, Denmark; ^3^Copenhagen University, Department of Biology, Copenhagen, Denmark

##### **Correspondence:** I. Jansen-Olesen

Objective: Calcitonin gene-related peptide receptor (CGRP-R) antagonists and monoclonal antibodies (mAB) against CGRP or its receptor have few side effects but erenumab in contrast to ligand binding mAB causes constipation in 40% of cases. CGRP activates both the CGRP-R and the structurally related amylin-1 receptor (AMY_1_-R) which have opposing effects on the GI tract. It is unknown if different affinity to these receptors may be the cause of constipation.

Methods: *Xenopus laevis* oocytes expressing human CGRP-R, human AMY_1_-R or their subunits was examined by two-electrode voltage clamp.

Results: CGRP induced a concentration-dependent increase in current in receptor expressing oocytes with the order of potency CGRP-R>>AMY_1_-R>calcitonin receptor (CTR). There was no effect on single components of the CGRP-R. Amylin was only effective on AMY_1_-R and CTR. Inhibition potencies (pIC_50_) for erenumab on CGRP-induced currents were 10.86 and 9.31 for CGRP-R and AMY_1_-R, respectively. Rimegepant inhibited CGRP-induced currents with pIC_50_ values of 11.30 and 9.91 for CGRP-R and AMY_1_-R, respectively.

Conclusions: Our results show that erenumab and rimegepant are potent inhibitors of CGRP-R and AMY_1_-R with 35- and 25-times preference for the CGRP-R over the AMY_1_-R. Clinically, the unopposed anti-peristaltic AMY_1_-R in the absence of the balancing CGRP-R may explain constipation by erenumab while ligand binding mAB keep the balance and cause no constipation.

## P054 Presentation of migraine in the media – Perception of patients and healthcare workers

### B. Raffaelli^1^, P. Kull^1^, J. Mecklenburg^1^, L. H. Overeem^1^, E. Storch^1^, M. Terhart^1^, L. Neeb^1^, U. Reuter^1^

#### ^1^Charité University Hospital Berlin, Department of Neurology, Berlin, Germany

##### **Correspondence:** B. Raffaelli

Objective: To investigate how patients and healthcare workers perceive stock images of migraine attacks.

Methods: We conducted an anonymous web-based survey among the following two groups: 1) Patients with migraine treated at the Charité Headache Center in 2020 (migraine group); 2) Charité employees and students (healthcare group). We presented ten selected stock pictures (Adobe Stock©) of migraine attacks to all participants. Participants rated on a scale from 0-100% how much each picture corresponds to a realistic migraine attack (*realism score*). We analysed the mean *realism score* for all pictures and in the following categories: male/female actors, younger/older actors, unilateral/bilateral pain pose.

Results: The survey was completed by 367 patients with migraine and 331 employees and students. In both groups, the mean *realism score* was <50% (47.8% ±18.3 in the migraine group and 46.0% ±16.2 in the healthcare group). Both patients and healthcare workers considered pictures with male actors more realistic than pictures with females (p<0.001) and pictures with older actors more realistic than those with younger actors (p<0.001). Only in the healthcare group, a bilateral pain posture was considered more realistic than a unilateral pain posture (p<0.001).

Conclusion: Standard images of migraine attacks are perceived as not realistic by patients and healthcare workers. A better representation in the media could help raise awareness for migraine and reduce the associated stigma.

## P055 Trigeminal, cervical, and widespread sensitization in the 4 phases of the migraine cycle

### M. Castaldo^1^, S. Di Antonio^1^, M. Ponzano^2^, F. Bovis^2^, P. Torelli^3^, C. Finocchi^4^, L. Arendt-Nielsen^1^

#### ^1^School of Medicine, Aalborg University, Denmark., Department of Health Science and Technology, Center for Pain and Neuroplasticity (CNAP), Aalborg, Denmark; ^2^University of Genoa, Department of Health Sciences (DISSAL), Section of Biostatistics, Genova, Italy; ^3^University of Parma, Headache Centre, Department of Medicine and Surgery, Parma, Italy; ^4^Ospedale Policlinico San Martino, Headache Centre, IRCCS, Genova, Italy

##### **Correspondence:** M. Castaldo

Objective: Assess pressure pain threshold (PPT) in trigeminal, cervical, and extra trigeminal/cervical pain-free areas in episodic migraine (EM) patients in all the 4 phases of the migraine cycle

Methods: Multicenter, cross-sectional, observational study. EM patients and healthy controls (HC) (age 18 -65) were included. Temporal summation of pain (10 consecutive stimuli, 50 g von Frey, 1 Hz frequency) in the trigeminal area and PPT over temporalis muscle, neck region, and dominant hand were assessed. A linear regression model using the variable group to predict the results was performed. Age and sex matched healthy controls were used as the reference group

Results: 48 Control, 38 interictal EM, 42 Preictal EM, 30 Ictal EM, and 26 postictal EM were included. Temporal summation was facilitated in Ictal EM compared to HC (p=0.003), with no other difference (p>0.092). In all phases, EM patients had lower PPT in the temporal and cervical area compared to HC (p<0.024; p<0.008). PPT over the dominant hand was reduced only in Preictal EM compared to HC (p=0.009), with no other differences (p>0.108)

Conclusion: EM patients in all phases of the migraine cycle have increased pressure pain sensitization of the trigeminocervical complex, with patients in the ictal phase have further enhanced sensitization. Signs of widespread sensitization are present only in preictal EM patients, and this may reflect an enhanced activation of cortical and subcortical areas in this phase.

**Fig. 1 (abstract P055).**
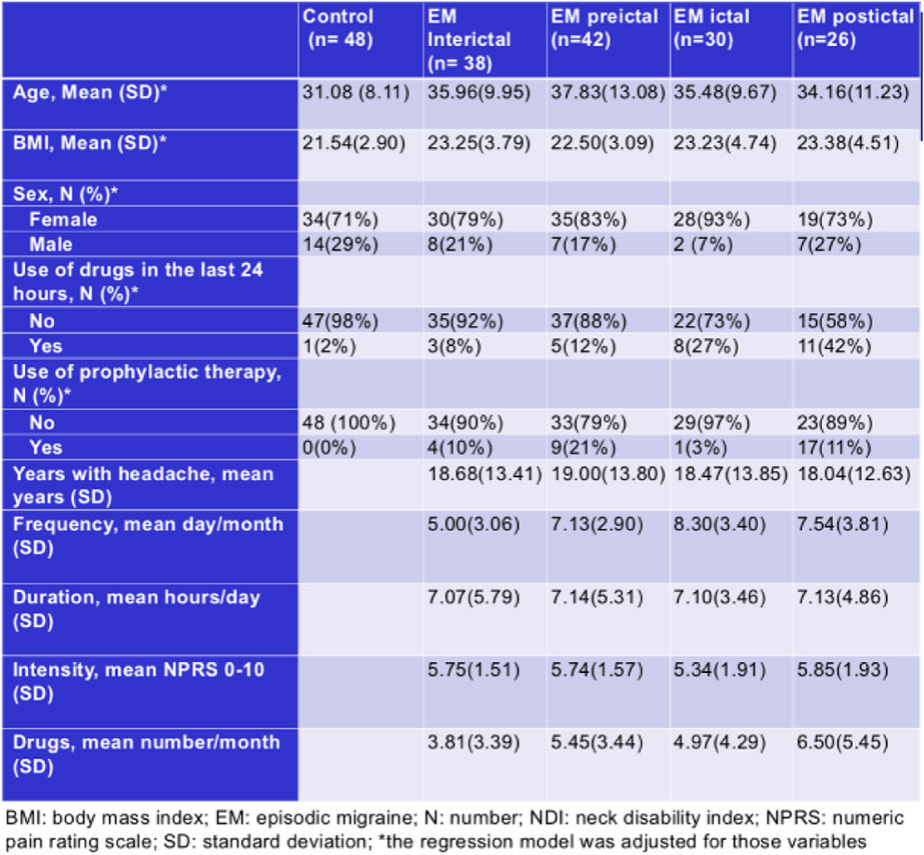
See text for description

**Fig. 2 (abstract P055).**
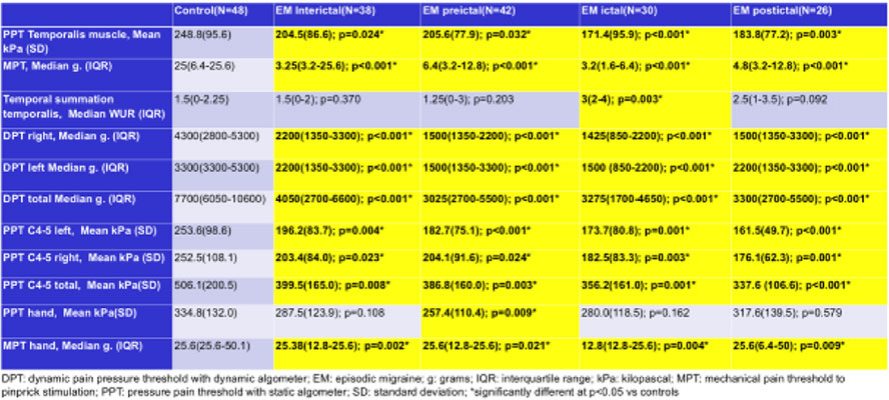
See text for description

## P056 Effects of endogenous PEA in the modulation of migraine pain

### R. Greco^1^, M. Francavilla^1^, C. Demartini^1^, A. M. Zanaboni^1,2^, C. Tassorelli^1,2^

#### ^1^IRCCS Mondino Foundation, Pavia, Italy; ^2^University of Pavia, Department of Brain and Behavioral Sciences, Pavia, Italy

##### **Correspondence:** M. Francavilla

Palmitoylethanolamide (PEA) is degraded preferentially by acylethanolamine acid amidase (NAAA), thus NAAA inhibition might be an amenable drug target for pain and inflammation control.

Objective: To evaluate the potential therapeutic effect of NAAA inhibition in an animal model of migraine.

Methods: Male Sprague-Dawley rats received nitroglycerin (NTG) or vehicle, followed by a NAAA inhibitor (ARN726) or vehicle. Four hours after NTG administration, the expected time of maximal expression of NTG-induced hyperalgesia, we evaluated in the open field the locomotor ability by calculating total distance and anxiety-related behavior by time spent in the central area for 10 minutes, then exposed them to the orofacial formalin test. Rats were then sacrificed to assess gene expression of IL-10 and IL-1beta in the meninges, trigeminal ganglion and medulla pons were collected to assess. CGRP serum levels were analyzed by ELISA kit.

Results: ARN726 significantly reversed NTG-induced trigeminal hyperalgesia, but it did not affect the inactivity induced by NTG injection. ARN726 also reduced IL-1beta mRNA levels in meninges and medulla-pons, as well as CGRP serum levels, while it increased IL-10 mRNA levels in meninges and trigeminal ganglion.

Conclusions: Our data show that NAAA inhibition has an anti-inflammatory effect in the NTG animal model of migraine, where it also prevents NTG-induced hyperalgesia. NAAA inhibition thus represents a potential drug target for migraine treatment.

## P057 Investigation of KATP channel opening and inhibition in different in vivo rodent models of migraine

### S. L. T. Christenen^1^, G. Munro^1^, S. Petersen^1^, A. Shabir^1^, I. Jansen-Olesen^1^, D. M. Kristensen^1^, J. Olesen^1^

#### ^1^Danish Headache Center, Glostrup, Denmark

##### **Correspondence:** S. L. T. Christenen

Objective: KATP channel agonist levcromakalim was shown to induce migraine attacks in migraineurs by a high incidence. To investigate the role of KATP channels in migraine, we first tested efficacy of KATP channel inhibition in two distinct rodent models of migraine. Secondly, using levcromakalim as provoking substance, we tested the inhibitory effect of a CGRP monoclonal antibody.

Methods: Hind paw and periorbital sensitivity to tactile stimulation were used as surrogate markers of migraine pain in three different rodent models: (i) the GTN mouse model of migraine, (ii) the STA (spontaneous trigeminal allodynia) rat model and (iii) a mouse model of levcromakalim induced migraine.

Results: The KATP channel antagonist glibenclamide inhibited the effect of GTN in mice and in STA rats the allodynia was alleviated. Mice injected repeatedly with levcromakalim (1 mg/kg, i.p.) developed a progressive hyperalgesia similar, but milder than that mediated by GTN (10 mg/kg, i.p.). The effect was completely inhibited by glibenclamide, but surprisingly also by a CGRP monoclonal antibody.

Conclusion: Reversal of tactile hypersensitivity in two distinct animal models indicates that KATP channel blockers could be effective drugs in the treatment of migraine. Despite KATP channel opening being a downstream event from CGRP binding to its receptor, we find a secondary release of CGRP *in vivo* after administration of levcromakalim as proven by the efficacy of the CGRP monoclonal antibody.

## P058 Profiling PACAP-responsive receptor pharmacology and agonist-dependent antagonism

### Z. Tasma^1^, D. Hay^1,2^, C. Walker^1^

#### ^1^University of Auckland, Biological Sciences, Auckland, New Zealand; ^2^University of Otago, Pharmacology and Toxicology, Dunedin, New Zealand

##### **Correspondence:** Z. Tasma

Objective: The pituitary adenylate cyclase-activating peptide (PACAP) system has recently been of interest for the treatment of migraine. The PACAP peptide family activate the PAC_1_, VPAC_1_ and VPAC_2_ receptors. Splice variants of the PAC_1_ receptor can differ in their agonist or signalling profiles. To aid development of therapeutics targeting the PACAP system, this study aimed to pharmacologically characterise PACAP-responsive receptor signalling and antagonism.

Methods: The pharmacology of the human PAC_1n_, PAC_1s_, VPAC_1_, VPAC_2_ receptors were examined in transfected Cos7 cells for five signalling molecules. The ability of antagonists to block PACAP-38, PACAP-27 and VIP was also determined.

Results: PACAP-responsive receptors exhibited varied pharmacological profiles but activated signalling in a similar manner. The PAC_1n_ and PAC_1s_ receptors displayed distinct pharmacology where VIP and PHM were more potent at the PAC_1s_ than the PAC_1n_ receptor. PACAP-responsive receptors displayed agonist-dependent antagonism where PACAP-38 was less effectively antagonised than PACAP-27 or VIP.

Conclusion: The distinct pharmacological profile displayed by the PAC1s receptor suggests that it can act as a dual receptor for VIP and PACAP. Furthermore, the effectiveness of blocking a signalling pathway can be influenced by which endogenous PACAP family agonist is present. These behaviours have potential implications for the development and effectiveness of drugs targeting the PACAP system.

## P059 Withdrawn

## P060 Intracellular pathways of calcitonin gene-related peptide-induced relaxation of human coronary arteries

### T. de Vries^1^, S. Labruijere^1^, A. van den Bogaerdt^2^, A. H. J. Danser^1^, A. Maassen van den Brink^1^

#### ^1^Erasmus University Medical Center, Division of Vascular Medicine and Pharmacology, Department of Internal Medicine, Rotterdam, Netherlands; ^2^ETB-BISLIFE, Heart Valve Department, Beverwijk, Netherlands

##### **Correspondence:** T. de Vries

Objective: Calcitonin gene-related peptide (CGRP) is an important neuropeptide in the pathophysiology of migraine and a target for novel anti-migraine medication. While its signaling is assumed to be mediated via increases in cAMP, we focused on actually elucidating intracellular signaling pathways involved in CGRP-induced relaxation of human isolated coronary arteries (HCA).

Methods: Concentration-response curves to CGRP (10 pM–1 μM) were constructed in HCA segments obtained from 11 male and 5 female donors (age 49±4 years), incubated with or without the PKA inhibitor Rp-8-Br-cAMPs (100 μM), the adenylate cyclase (AC) inhibitors SQ22536 (100 μM) and 2′,3′-dideoxyadenosine (DDA, 10 μM), or the guanylate cyclase (GC) inhibitor ODQ (10 μM).

Results: The AC inhibitors SQ22536 and DDA, and the PKA inhibitor Rp-8-Br-cAMPs, did not inhibit the CGRP-induced relaxation of HCA, nor did the GC inhibitor ODQ.

Conclusion: While CGRP signaling is generally assumed to act via cAMP, the CGRP-induced vasodilation in HCA could not be inhibited by targeting this intracellular signaling pathway at different levels. As inhibition of GC also did not affect relaxations to CGRP, it is important to further identify the intracellular signaling cascade after binding of CGRP to its receptor in human arteries. This would ultimately allow novel anti-migraine medication to target specific parts of the intracellular signaling pathway that reduces migraine, while limiting (cardiovascular) side effects.

## P061 The impact of glucose on mitochondrial function in a brain slice model of cortical spreading depression

### O. Grech^1^, D. Fulton^2^, Z. Alimajstorovic^1^, S. Heising^1^, D. Cartwright^1^, B. R. Wakerley^3^, S. P. Mollan^4^, A. J. Sinclair^1,3,5^, G. G. Lavery^1,5^

#### ^1^University of Birmingham, Institute of Metabolism and Systems Research, Birmingham, United Kingdom; ^2^University of Birmingham, Institute of Inflammation and Ageing, Birmingham, United Kingdom; ^3^University Hospitals Birmingham NHS Foundation Trust, Department of Neurology, Birmingham, United Kingdom; ^4^University Hospitals Birmingham NHS Foundation Trust, Birmingham Neuro-ophthalmology Unit, Birmingham, United Kingdom; ^5^Birmingham Health Partners, Centre for Endocrinology, Diabetes and Metabolism, Birmingham, United Kingdom

##### **Correspondence:** O. Grech

Objective: A fundamental mechanism of migraine is cortical spreading depression (CSD), a wave of depolarisation across the cortex. Fasting is noted to trigger and aggravate migraine attacks. Using a mouse brain slice model, we investigated the impact of hypoglycaemia and CSD on mitochondrial function.

Methods: CSD was induced with 1ul 2M KCl (KCl+ vs. KCl-) to cortical regions of acute brain slices (C57BL/6J). 10 minutes after KCl, mitochondrial oxidative respiration was assessed using Oroboros O2k oxygraphy, in the presence or absence of glucose (10mM vs 0mM). CSD was measured with Fluo-4-AM, a fluorescent calcium indicator.

Results: Fluo-4-AM induced a wave of calcium following KCl. In KCl- and KCl+ slices basal respiration is unchanged, however, in the absence of glucose we observed increased oxidative capacity from complex I and II (12.54 vs. 17.94 pmol O2/mg-1/s-1 p = 0.002) and maximal rates of uncoupled respiration (13.60 vs. 19.93 pmol O2/mg-1/s-1, p<0.001, n = 6). This was rescued with glucose (n=6).

Conclusions: In the absence of glucose, CSD increased the oxidative capacity of complex I and II and maximal rates of uncoupled respiration, which was rescued by glucose (10mM). Energetic deficits due to glucose deficiency may trigger upregulated oxidative respiration following CSD, in order to compensate. Adequate glucose appeared to ameliorate CSD disturbances in energy metabolism. This may be relevant to observations that migraine attacks are aggravated by fasting.

## P063 Evaluation of the burden of migraine on the partner´s lifestyle: a multicenter study

### E. M. Fernández Bermejo^1^, Á. Planchuelo-Gómez^2^, S. Quintas^3^, A. González Martínez^3^, D. García-Azorín^4,5^, Á. Sierra Mencía^4^, Á. L. Guerrero Peral^4,5,6^, S. Santos-Lasaosa^7^, M. P. Navarro-Pérez^7^, N. González García^8^, J. Díaz de Terán Velasco^9^, A. B. Gago Veiga^1,3^

#### ^1^Universidad Autónoma de Madrid, Medicine Department, Madrid, Spain; ^2^Universidad de Valladolid, Imaging Processing Laboratory, Valladolid, Spain; ^3^Hospital Universitario de la Princesa & Instituto de Investigación Sanitaria de la Princesa, Headache Unit, Department of Neurology, Madrid, Spain; ^4^Hospital Clínico Universitario de Valladolid, Headache Unit, Department of Neurology, Valladolid, Spain; ^5^Institute for Biomedical Research (IBSAL), Salamanca, Spain; ^6^University of Valladolid, Deparment of Medicine, Valladolid, Spain; ^7^Hospital Clínico Universitario Lozano Blesa & Aragon Institute for Health Research, Headache Unit, Department of Neurology, Zaragoza, Spain; ^8^Hospital Universitario Clínico San Carlos, Headache Unit, Department of Neurology, Madrid, Spain; ^9^Hospital Universitario de La Paz, Headache Unit, Neurology, Madrid, Spain

##### **Correspondence:** E. M. Fernández Bermejo

Objective: Migraine is a highly disabling disease that affects the patient"s life, but its consequences on the patient"s partner have been barely studied. The objective was to analyze these effects on romantic relationship, relationship with their children, friendship and work; as well as to evaluate caregiver burden and the presence of anxiety and/or depression.

Methods: Cross-sectional observational study. An online survey was filled by partners of migraine patients from five Spanish Headache Units. Questions about the four assessed areas and two scales to evaluate anxiety, depression and caregiver burden (Hospital Anxiety and Depression Scale and Zarit scale) were included. The presence of anxiety and depression was compared to the Spanish prevalence (6.7% in both cases).

Results: Out of 176 registered responses, 155 were accepted. The sample included 86.5% of women, with mean age 44.2 ± 10.4 years. Effects on partners were found on love relationship and items concerning children and friendships, with a minor impact at work. Partners showed a significant moderate burden according to the Zarit scale (p = 12/155 = 0.077 [0.041-0.131]; *p* < 0.001) and a higher anxiety rate than the 6.7% national prevalence (p = 23/155 = 0.148 [0.096-0.214]; *p* < 0.001), but similar depression rate.

Conclusion: We found an impact on the patient's partners on the studied areas. Migraine is a disease that implies caregiver burden in the patient's environment with possible effect on anxiety levels.

## P064 Opioid use for acute headache treatment in a Brazilian Emergency Department

### G. Taricani Kubota^1^, M. Nattan Portes Souza^1^, M. Calderaro^1^, A. P. de Sousa Oliveira^1^, G. Kuster^2^, M. Rodrigues Jordao^1^

#### ^1^Hopital Samaritano de São Paulo - unidade Higienópolis, São Paulo, Brazil; ^2^Americas Serviços Médicos, São Paulo, Brazil

##### **Correspondence:** G. Taricani Kubota

Objective: To assess the frequency of opioid use for acute headache treatment in the Emergency Department (ED) of a private Hospital in Brazil.

Methods: Cross-sectional study which included all patients admitted to the ED of the Sao Paulo Samaritano Hospital in 2018, who were diagnosed with International Classification of Diseases codes R51, G43 or G44. The subjects treated with opioids were compared to those who were not for demographical characteristics, ED visit duration and healthcare-related costs.

Results: We identified 3,943 ED visits due to headache, and opioids were used in 11.3% of these. The types of administered opioids were: tramadol (92.4%), morphine (3.9%), tramadol and acetaminophen combination (3.3%) and nalbuphine (0.2%). Subjects who received opioids had greater probability to return to the ED in the same studied year (OR 1.61, 95%CI 1,3-1,99). They also stayed 45.5% longer in the ED than those who did not receive opioids. Average cost *per* visit among opioid-treated subjects was 51.1% greater (95%CI 21.4-42.3%). Subjects who received opioids were more frequently: female (p=0.018) and admitted in the period from 0:00 a.m. to 6:00 a.m (p<0.001).

Conclusion: We found a high frequency of opioid use for acute headache treatment in the ED, however lower than those previously reported by north-american studies. Opioid use was associated with higher healthcare-related costs *per* visit. These data were previously presented at the 33^th^ Brazilian Headache Congress.

## P065 Association of daytime sleepiness with recurrent headache in Russian urban adolescents: a school-based study

### S. Tereshchenko^1^, M. Shubina^1^, N. Gorbacheva^1^, I. Novitckii^1^

#### ^1^Scientific Research Institute of Medical Problems of the North, Department of child's physical and mental health, Kranoyarsk, Russian Federation

##### **Correspondence:** S. Tereshchenko

Objective: Excessive daytime sleepiness (EDS) is one of the most common sleep disorders in adolescents associated with social behaviors patterns and school performance. We aimed to investigate EDS comorbidity with recurrent headache in Central Siberia urban adolescents.

Materials and Methods: 4680 urban Siberian (Krasnoyarsk, Abakan, Kysyl) school-based adolescents (aged 12-18; boys/girl ratio 2190/2490) were tested with Pediatric Daytime Sleepiness Scale (PDSS); cutoffs for EDS were PDSS 95% percentiles for each age group. Adolescents were also asked about headache presence/frequency and pain intensity according to 6-points Visual Analogue Scale (VAS). Clinically relevant recurrent headache was diagnosed at headache frequency ≥ 2 per month and VAS score ≥ 4 points. Chi-square and Mann-Whitney tests were used.

Results: The recurrent headache group exhibited a higher prevalence of EDS in comparison with the non-headache group (1.50% and 4.81%, respectively, p<0.001). PDSS score was higher in headachers (headache group – 14 (10-18), non-headache group 10 (7-14), p<0.001). Also, we found a positive correlation between PDSS and VAS scores (Spearman's r=0.366, p<0.05)

Conclusion: EDS is strongly associated with recurrent headache in Siberian adolescents. The possible explanations of this relation may be night sleep disturbances in headachers and the presence of common pathogenic factors, such as personality characteristics, depression, anxiety.

## P066 Interactions of the neuropeptide galanin with cortical spreading depolarization and cortical neuronal excitability

### F. Gimeno-Ferrer^1^, A. Eitner^1^, R. Bauer^2^, A. Lehmenkühler^3^, H. G. Schaible^1^, F. Richter^1^

#### ^1^University Hospital Jena, Institute of Physiology I/ Neurophysiology, Jena, Germany; ^2^University Hospital Jena, Center of Molecular Biomedicine, Jena, Germany; ^3^Pain Institute & Center for Medical Education, Düsseldorf, Germany

##### **Correspondence:** F. Gimeno-Ferrer

Galanin modulates in hippocampal neurons the release of neurotransmitters, and thereby it influences neuronal excitability. To test its actions on neurons in cerebral cortex, we recorded ongoing brain activity and induced cortical spreading depolarization (CSD) before and after application of galanin.

In spontaneously breathing anesthetized adult rats (sodium thiopentone, 100 mg/kg, i.p.) the electrocorticogram was recorded with arrays of glass microelectrodes in two areas (treated with galanin and untreated) and different depths. CSD was induced by KCl microinjection. CSD-related potential shifts, changes in extracellular potassium concentration and in regional cerebral blood flow were continuously monitored. Galanin at concentrations of 10^-6^, 10^-7^, 10^-8^, 10^-9^, and 10^-10^ M was applied for 3 h and then washed away with artificial cerebrospinal fluid.

Galanin at concentrations of 10^-6^, 10^-7^, and 10^-8^ M increased the threshold for elicitation of CSD, reduced amplitudes of CSD and significantly slowed propagation velocity of CSD. In some rats, during galanin, the CSD propagated only in the untreated area. Lower concentrations of galanin had no significant effects. In the washout phase after these three concentrations of galanin ictal discharging or repetitive seizure activity were observed in nearly 50 % of rats.

We conclude that galanin has the potential to control cortical neuronal activity. This could be a target for brain diseases that involve cortical hyperexcitablity.

## P069 Headache Disability, Lifestyle Factors, Health Perception, and Mental Disorder Symptoms in the 2013 National Health Survey in Brazil: A cross-sectional analysis

### A. Olieira^1,2,3^, J. Mercante^1,2,3^, A. Goulart^1^, I. Benseñor^1^, M. Peres^2,3^

#### ^1^Universidade de São Paulo, Center for Clinical and Epidemiological Research, São Paulo, Brazil; ^2^Hospital Israelita Albert Einstein, Instituto do Cérebro, São Paulo, Brazil; ^3^Universidade de São Paulo, Instituto de Psiquiatria, São Paulo, Brazil

##### **Correspondence:** A. Olieira

Objective: To estimate headache disability and explore its association with lifestyle factors, health perception, and mental disorders symptoms in a national health survey in Brazil.

Methods: In a cross-sectional analysis of the PNS 2013 Survey, logistic regression models computed the associations between headache-related disability (days lost from work, school, or household chores in the past 2 weeks) and lifestyle factors, health perception, and mental disorders symptoms compared to other disease-related disabilities or no day lost group. The adjusted models controlled for the effects of age, sex, income, and educational levels.

Results: In the sample aged ≥ 18 years (n = 145,580), 10,728 (7.4 %) participants reported any disease-related disability in the past 2 weeks [median interquartile range (IQR) for age = 47 (33-59) years, 62 % women], with the median (IQR) days lost = 5 (2-14). Headache disability represented 5.3 % (572/10,728) of all diseases, constituting the 2nd as most prevalent disability (13%) in young people, Headache disability associated with physical inactivity, poorer health perception and mental disorders symptoms.

Conclusion: Headache disability represents a leading cause of disease-related disability in Brazil, and associates with unhealthy lifestyle factors, poorer health perception, and mental disorders symptoms.

## P070 Headache patient analsys in LLC Vidzemes Hospital, Latvia, emergency room

### M. Siliņa^1^, L. Smeltere^1^

#### ^1^University of Latvia, Faculty of medicine, Rīga, Latvia

##### **Correspondence:** M. Siliņa, L. Smeltere

Objective: Headache patients in the emergency room (ER) present multiple challenges, like excluding secondary headache (SH), providing efficient pain relieve for primary headache (PH) patients, provide recommendations for treatment. Research in this field could improve execution of these tasks.

Methods: This prospective research involves quantitative analysis of 50 ER patients with primary complaint of headache over 4-month period. The data of patient questioning, examination are documented in a specially designed questionnaire.

Results: Out of 50 patients, aged 22 to 77 years, 48% were PH patients, whereas 36% were SH, 16% unspecified headache patients. Most common headache types in this research are listed in Table 1. Although 68% of the patients had previously suffered a headache, 14% had sought medical attention, but only 10% are diagnosed with a headache disorder. 54% described their headache at 4 out of 5 points, with 62% experiencing accompanying symptoms, such as nausea (41%), photophobia (24%). Almost all participants (90%) presented with at least one "red flag" symptom for SH, such as unfamiliar headache (26%), meningeal signs (13%). Only 28% needed to be hospitalized.

Conclusion**:** Majority of headache patients in the ER consist of PH patients, who can be treated on outpatient basis. More patients need to seek medical care to be diagnosed and receive treatment. Better education and detailed recommendations are necessary for all patients, to reduce unnecessary ER visits.

**Table 1 (abstract P070).**
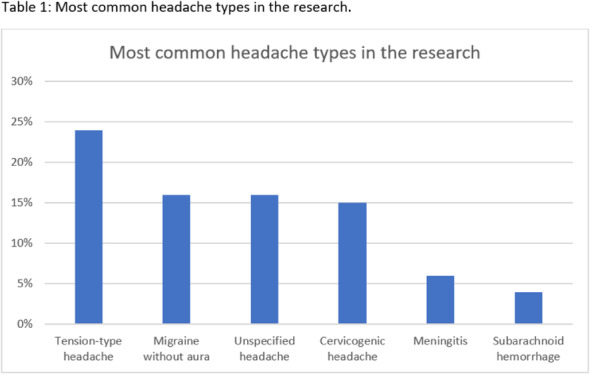
See text for description

## P072 Intoxication profiles in a Dutch migraine cohort

### T. van den Hoek^1^, I. Verhagen^1^, I. de Boer^1^, G. M. Terwindt^1^

#### ^1^Leiden University Medical Center, Neurology, Leiden, Netherlands

##### **Correspondence:** T. van den Hoek

Migraine patients are interested in lifestyle influences on their attacks and 25% have stopped consuming or never consumed alcohol because of presumed trigger effects (Onderwater et al. Eur J Neurol. 2019; 26:588-95). However, it is unknown if they apply restrictions on potential triggers compared to healthy controls and the general Dutch population.

In this cross sectional study in migraine patients and controls from the Leiden LUMINA cohort, we collected data for alcohol, tobacco and illicit drug consumption. Data from the general population (GP) were extracted from the annual health survey (Statistics Netherlands).

From the LUMINA-cohort, n=6228 subjects were included (migraine n=5689, controls n=539). In the migraine group, 5487 subjects provided data to distinguish between episodic (EM) and chronic migraine (CM). From the general population 14,542 subjects were included. Migraine patients used less illicit drugs, tobacco and alcohol compared to GP (p<0.01). For our control group; controls had higher consumption of alcohol and packyears (both p<0.01) compared to migraineurs, but showed reduced illicit drug use compared to GP (p<0.01). CM had lower consumption and less consumers of alcohol (p<0.01) compared to EM, more smokers and packyears (p<0.01), but no difference in illicit drug use.

Migraine patients avoid illicit drugs, tobacco and alcohol compared to the GP. CM report less alcohol use, more smoking, but similar low illicit drug use compared to EM.

## P073 Salivary CGRP can help monitor the different migraine phases: CGRP (in)dependent attacks

### A. Alpuente^1,2^, V. J. Gallardo^2^, L. Asskour^2^, E. Caronna^2^, M. Torres-Ferrus^1^, P. Pozo-Rosich^1,2^

#### ^1^Vall d'Hebron University Hospital, Headache Unit, Neurology Department, Barcelona, Spain; ^2^Autonomous University of Barcelona, Medicine Department, Barcelona, Spain

##### **Correspondence:** A. Alpuente

Background: CGRP plays a key role in the pathogenesis of migraine.

Objective: To assess saliva as a substrate to measure CGRP by comparing interictal levels in episodic migraine (EM) and controls (HC); and to evaluate its temporal profile during migraine attacks.

Methods: This is a prospective observational pilot study in which we monitored salivary CGRP during 30 consecutive days and during migraine attacks. We considered 6 timepoints:interictal (72h headache free), preictal(PRE-24h before), ictal (headache onset, after 2h and 8h), postictal (POST-24h after).

Results: 44 women (22 EM, 22 HC) were recruited. Differences in interictal salivary levels of CGRP between EM and HC (98.0[80.3] (95% CI 56.6, 124.0) vs. 54.3[44.0](95% CI 42.2, 70.1)pg/mL, p=0.034) were found. An increase in CGRP levels during attacks was detected (pre:112.0[130.0](95% CI 58.5,169.0);

headache onset: 162.0[186*.*0](95% CI 105.0,240.0);

after 2h:102.0[131.0](95% CI 46.6,165.0); after 8h: 82.1[154.0](95% CI 47.6,166.0); post: 85.6[122.0](95% CI 46.3,160.0) pg/ml;p<0.001). Patients were classified as having CGRP-dependent (79.6%) and non-CGRP dependent migraine attacks (20.4%) according to the magnitude of change between preictal and ictal. Photophobia and phonophobia were significantly associated to first group.

Conclusions: Salivary CGRP levels, which interictally are elevated in episodic migraine, usually increase during a migraine attack in the majority of patients. However, not every attack is CGRP-dependent.

**Fig. 1 (abstract P073).**
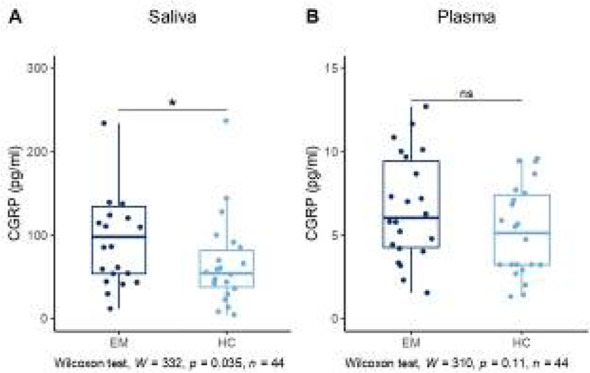
See text for description

**Fig. 2 (abstract P073).**
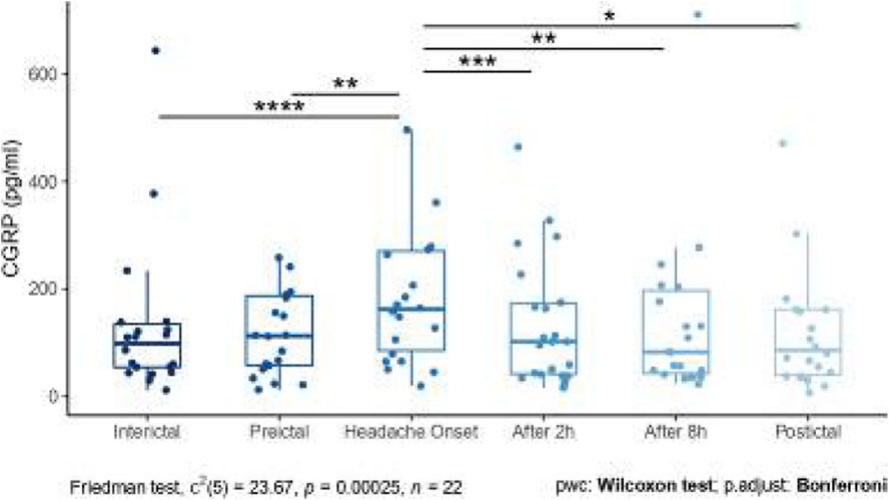
See text for description

## P074 Micro-array analysis of the hypothalamus in an animal migraine model

### R. Abuukar Abdullahi^1,2^, T. Takahashi^2^, D. Chambers^3^, G. Williams^3^, B. Okine^2^, G. Lambru^1^, A. P. Andreou^1,2^, R. Abdullahi^1,2^

#### ^1^Guy's and St Thomas' Hospital NHS Foundation Trust, Headache Centre, London, United Kingdom; ^2^King's College, Headache Research, London, United Kingdom; ^3^King's College, Wolfson centre for age-related diseases, London, United Kingdom

##### **Correspondence:** R. Abuukar Abdullahi

Objective: Migraines can be defined as a cyclical disorder with 4 phases, the premonitory phase reflects the initiation of the migraine and hypothalamic dysfunction has been implicated in it. Infusion of GTN has been shown to trigger migraines and premonitory symptoms similar to those seen in spontaneous attacks. This study aims to investigate early transcriptional responses to GTN-infusion in the mouse hypothalamus in order to elucidate the mechanisms involved in the initiation of migraine attacks.

Methods: Mice were anaesthetized and infused with GTN or vehicle for 30 minutes. Thereafter, the hypothalamus was collected for microarray analysis. Expression patterns of selected genes were confirmed by qPCR. Pathways analysis was carried out using DAVID Bioinformatics Resource.

Results: Differences in gene expression were detected in 45037 genes between treatments, and of those 864 were significantly different (P <0.05). The DAVID analysis demonstrated enrichment of pathways suggesting an increase in circadian rhythm, signal transduction and immune responses.

Conclusion: Several of the pathways known to be involved at later phases in migraine, such as dysregulation of neurotransmission, seem to be initiated in the hypothalamus. Additionally, we found unexpected enrichment in pathways, such as inflammation which previously have not been reported in the premonitory phase of migraine. Further studies are needed to assess their role in the initiation of a migraine attack.

## P075 The influence of calcitonin gene-related peptide on cerebral hemodynamics and calcitonin gene-related peptide-induced headache in migraine with aura

### M. Zaletel^1^, D. Visočnik^1^, M. Zupan^1^, B. Žvan^1^

#### ^1^University Medical Centre Ljubljana, Dept.of Vascular Neurology, Ljubljana, Slovenia

##### **Correspondence:** M. Zaletel

Background: Exogenous calcitonin gene-related peptide (eCGRP) can induce CGRP induced headache (CGRP-IH). In patients with migraine with aura (MA), eCGRP may induce aura attacks. This implies a common pathophysiological mechanism of trigeminovascular sensitization (TVS). We predicted that cerebral hemodynamic detected by TCD and induced by eCGRP differ between migraine without aura (MwA) and MA using TCD.

Methods: Twenty patients with migraine participated in our study Fifteen patients had MwA and 5 patients had MA. We performed a multimodal TCD monitoring during and after eCGRP infusion, recording arterial velocity in the middle (MCA) and posterior cerebral arteries (PCA), end-tidal carbone dioxide (Et-CO2), mean arterial pressure (MAP) and heart rate (HR). We calculated the responses between different time points during the experiment and composed variables vm MCAtot, vm PCAtot, Et-CO2tot, MAPtot, and HRtot.

Results: The CGRP-IH appeared in 5 patients with MA (100%) and in 11 patients with MwA (73.3%) (p=0.530). The difference of changes in vm MCAtot (p = 0.014), and vm PCAtot (p = 0.004) was significant. Logistic regression showed significant association between vm MCAtot and MA (p = 0.023), and vm PCAtot and MA (p = 0.018).

Conclusions: Our study shows that cerebral hemodynamics clearly differ between MwA and MA indicating a higher degree of vasodilatation and TVS in MA. TVS with neurogenic inflammation might be connected with aura.

## **P076 The effect of KATP channel blocker glibenclamide on CGRP-induced headache and hemodynamic in healthy volunteers**

### H. Coskun^1^, F. A. Elbahi^1,2^, M. A. Al-Karagholi^1^, H. Ghanizada^1^, M. Sheykhzade^2^, M. Ashina^1^

#### ^1^Danish Headache Center, Neurology, Glostrup, Denmark; ^2^University of Copenhagen, Drug Design and Pharmacology, Copenhagen, Denmark

##### **Correspondence:** F. A. Elbahi

Background: Calcitonin gene-related peptide (CGRP) dilates cranial arteries and triggers headache. The CGRP signaling pathway is partly dependent on activation of ATP-sensitive potassium (KATP) channels. Here, we investigated the effect of the KATP channel blocker glibenclamide on CGRP-induced headache and vascular changes in healthy volunteers.

Methods: In a randomized, double-blind, placebo-controlled, cross-over study, 20 healthy volunteers were randomly allocated to receive an intravenous infusion of 1.5 μg/min CGRP after oral pretreatment with glibenclamide or placebo. The primary endpoints were the difference in incidence of headache and the difference in AUC for headache intensity scores between glibenclamide and placebo. The secondary endpoints were the difference in AUC for VMCA, STA and RA diameter, facial flushing, HR and MAP between glibenclamide and placebo.

Results: We found no significant difference in the incidence of headache between glibenclamide-CGRP day and placebo-CGRP day *(P=0.06)*. The AUC for headache intensity, VMCA, STA, RA, facial skin blood flow, HR and MAP did not differ between glibenclamide-CGRP day compared to placebo-CGRP day (*P > 0.05*).

Conclusion: Pretreatment with a non-selective KATP channel inhibitor glibenclamide did not attenuate CGRP-induced headache and hemodynamic changes in healthy volunteers. We suggest that CGRP-induced responses could be mediated via activation of specific isoforms of sulfonylurea receptor subunits of KATP channel.

## P077 Diffusion tensor imaging and neurite orientation and dispersion imaging in patients with migraine

### Y. Shibata, S. Ishiyama

#### University of Tsukuba, Neurosurgery, Mito, Ibaraki, Japan

##### **Correspondence:** Y. Shibata

Objective: We examined the diffusion tensor imaging (DTI) parameters and neurite orientation and dispersion imaging (NODDI) in patients with migraine and healthy control.

Materials and Methods: Twenty-six patients with migraines and 24 healthy controls were recruited. All patients underwent DTI and NODDI using 3.0 T MRI. The fractional anisotropy, mean diffusivity, axial diffusivity, radial diffusivity, orientation dispersion index (ODI), the fraction of intracellular volume (ficv), and the fraction of iso-diffusion (fiso) values in the whole brain were analyzed using tract-based spatial statistics.

Results: Twenty-six migraine patients (43.4±13.8 years old) and 23 healthy control (44.9±12.7 years old) were included for the analysis. The mean disease duration of migraine was 21.3±15.9 years. Migraine frequency was episodic for 16 patients, and chronic for 10 patients. Medication overuse headache was associated in 4 migraine patients.

There were no significant differences between migraine patients and healthy control in the DTI and NODDI parameters. The ficv of chronic migraine patients showed slightly lower at right temporal than those of episodic migraine patients.

Conclusion: Our study demonstrated neurite damage in chronic migraine patients. NODDI may be useful to understand the pathophysiology of migraines.

**Fig. 1 (abstract P077).**
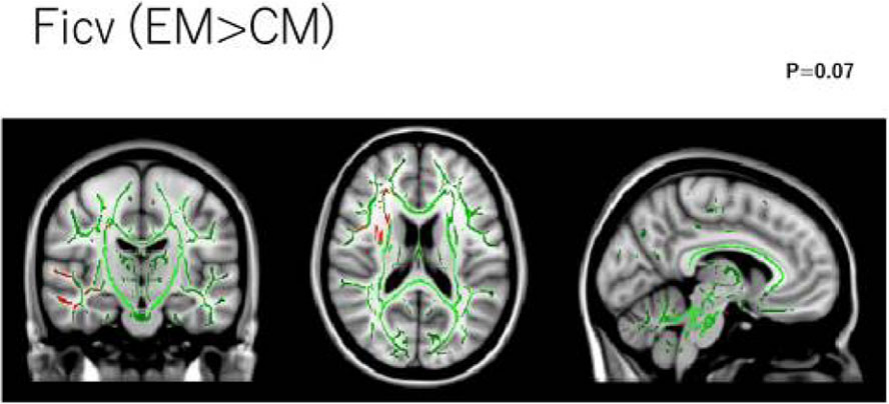
See text for description

## P078 Effect of adrenomedullin on migraine-like attacks in patients with migraine: A randomized crossover study

### H. Ghanizada

#### Danish Headache Center, Neurology, Taastrup, Denmark

Objective: To determine whether the intravenous infusion of adrenomedullin, a potent vasodilator belonging to calcitonin family of peptides, provokes attacks of migraine in patients.

Methods: Twenty migraine without aura patients participated in a placebo-controlled and double-blinded clinical study. In a randomized and crossover design the patients received an intravenous infusion of human adrenomedullin (19.9 picomole/kg/min) or placebo (saline). The primary outcome of the study was predefined as a difference in migraine incidence (0–12 h) and the secondary outcome were the headache intensity score"s area under curve (AUC0-12 h).

Results: Eleven migraine without aura patients (55%) fulfilled migraine attacks criteria after adrenomedullin infusion in comparison to only three patients reported attack (15%) after placebo (*P=* 0.039). We found that patients reported in a period of (0-12 hours) stronger headache intensity after adrenomedullin in comparison to placebo infusion (*P=* 0.035). AUC0-90 min for HR and, flushing (*P* < 0.05) were significant and MAP (*P* = 0.502) remain unchanged. Common adverse events reported were facial flushing, heat sensation and palpitation (*P* <0.001)

Conclusion: Our data implicate adrenomedullin in migraine pathogenesis. This suggests that adrenomedullin and/or its receptors are novel therapeutic targets for the treatment of migraine. However, we cannot discount for the possibility that adrenomedullin may be acting through the canonical CGRP receptor.

## P079 Vasoactive intestinal polypeptide (VIP) induces migraine attacks: A randomized clinical trial

### L. Pellesi^1^, M. A. Al-Karagholi^1^, R. De Icco^2,3^, H. Coskun^1^, F. A. Elbahi^1^, C. Lopez-Lopez^4^, J. Snellman^4^, J. Hannibal^5^, F. M. Amin^1^, M. Ashina^1^

#### ^1^Danish Headache Center, Copenhagen, Denmark; ^2^Headache Science & Neurorehabilitation Center, IRCCS Mondino Foundation, Pavia, Italy; ^3^Department of Brain and Behavioral Sciences, University of Pavia, Pavia, Italy; ^4^Novartis Pharma AG, Basel, Switzerland; ^5^Bispebjerg Frederiksberg Hospital, Department of Clinical Biochemistry, Copenhagen, Denmark

##### **Correspondence:** L. Pellesi

Background and Objective**:** Vasoactive intestinal polypeptide (VIP) and pituitary adenylate cyclase-activating polypeptides (PACAPs) are structurally and functionally related yet different in their migraine-inducing properties. It remains unclear whether the lack of migraine induction can be attributed to the transient vasodilatory response of VIP. We hypothesized that 2-hour infusion of VIP would provoke migraine attacks.

Methods**:** A randomized, double-blind, placebo-controlled, crossover study was conducted at the Danish Headache Center (Denmark). Twenty-one patients (seventeen females and four males) were randomly allocated to receive a two-hour infusion of VIP or placebo on two different days, separated by at least one week (ClinicalTrials.gov, NCT04260035).

Results**:** Fifteen patients (71%) developed migraine attacks after VIP compared to one patient (5%) after placebo (*p* = 0.0005). The VIP-induced migraine attacks mimicked patients´ spontaneous attacks. The area under the curve (AUC) of headache intensity scores (0-12 h), as well as the AUC of the superficial temporal artery diameter (0-180 min) were significantly greater after VIP compared to placebo (AUC0-12h, *p* = 0.0028; AUC0-180min, *p* < 0.0001).

Conclusion**:** Two-hour infusion of VIP caused migraine attacks, suggesting a role of VIP in migraine pathophysiology. The role of VIP and/or a prolonged dilation of cranial arteries is critical in migraine initiation.

## P080 Opening of ATP sensitive potassium channels causes migraine attacks with aura

### M. A. Al-Karagholi, H. Ghanizada, C. A. Nielsen, A. Hougaard, M. Ashina

#### Danish Headache Center, Copenhagen, Denmark

##### **Correspondence:** M. A. Al-Karagholi

The common pathophysiological mechanisms underlying migraine headache and migraine aura are yet to be identified. Based on recent data, we hypothesized that levcromakalim, an ATP-sensitive potassium channel opener, would trigger migraine attacks with aura in migraine with aura patients.

In a randomized, double-blind, placebo-controlled, crossover study, 17 patients aged 21-59 years and diagnosed with migraine with aura exclusively were randomly allocated to receive an infusion of 0.05 mg/minute levcromakalim or placebo (isotonic saline) on two different days (ClinicalTrials.gov, ID: NCT04012047). The primary endpoints were the difference in incidence of migraine attacks with or without aura, headache and the difference in the area under the curve for headache intensity scores (0–12h).

Seventeen patients completed the study. Fourteen of 17 (82%) patients developed migraine attacks with and without aura after levcromakalim compared with 1 of 17 (6%) after placebo (*P<0*.*001*). Ten patients (59%) developed migraine with aura after levcromakalim compared with none after placebo (*P=0.002*). One additional patient reported "possible" aura, only partially fulfilling the criteria.

is likely a novel migraine aura-inducing substance in humans. These findings highlight the ATP-sensitive potassium channel as a shared target in migraine aura and migraine headache. Likely, ATP-sensitive potassium channel opening leads to triggering of aura and headache, respectively, via distinct mechanisms.

## P081 Resting-state Occipital Alpha Power is associated with Treatment Outcome in Patients with Chronic Migraine

### F. J. Hsiao^1^, L. L. Pan^1^, W. T. Chen^1,2^, S. J. Wang^1,2^

#### ^1^National Yang-Ming Chiao-Tung University, Brain research center, Taipei City, Taiwan; ^2^Taipei Veteran General Hospital, Department of Neurology, Neurological Institute, Taipei City, Taiwan

##### **Correspondence:** F. J. Hsiao

Background and Objective: Preventive treatment is crucial for patients with chronic migraine (CM). Present study aimed to associate 3-month preventive treatment outcomes of flunarizine with resting-state cortical oscillations at baseline.

Methods: Treatment naïve CM patients and healthy controls (HC) were recruited. Resting-state EEG data was recorded under eye-closed condition and analyzed over the bilateral primary somatosensory (S1) and visual (V1) cortex. According to the changes of the monthly headache days (MHDs) (3-month vs. baseline), CM patients were arranged into responders (≥50% decrease) and non-responders. The oscillatory powers were compared between groups (CM and HC; responders and non-responders).

Results: The demographic, clinical and resting-state EEG data from 72 CM and 50 HC were analyzed. No significant difference was observed in the demographic data; however, elevated level of anxiety, depression, and stress were noted in CM. Resting-state theta power in bilateral S1 and alpha and gamma power in the right S1 were increased for CM. Regarding the treatment outcome, augmented alpha powers in bilateral V1 were noted in non-responders. The alpha powers exhibited significant correlations with the change of MHDs.

Conclusion: Resting-state occipital alpha activities at baseline determine the 3-month treatment outcome. This EEG measurement might be one of the signatures for conceivable treatment plan towards personalizing migraine medicine.

## P082 Cortical abnormalities in pediatric patients with migraine without aura: analysis of gyrification morphology and cortical thickness

### G. Sforza^1^, L. Papetti^1^, A. Guarnera^2^, R. Moavero^1,3^, F. Ursitti^1^, M. A. N. Ferilli^1^, S. Tarantino^1^, A. Napolitano^4^, D. Longo^2^, M. Valeriani^1,5^

#### ^1^Bambino Gesù Children's Hospital, Neurology, Rome, Italy; ^2^Bambino Gesù Children's Hospital, Neuroradiology Unit, Imaging Department, Rome, Italy; ^3^Tor Vergata University, Child Neurology and Psychiatry Unit, Systems Medicine Department, Rome, Italy; ^4^Bambino Gesù Children's Hospital, Medical Physics Unit, Risk Management Enterprise, Rome, Italy; ^5^Aalborg University, Center for Sensory-Motor Interaction, Aalborg, Denmark

##### **Correspondence:** G. Sforza

Objective: To verify the presence of abnormalities of the morphology of gyrification and cortical thickness (CT) in pediatric patients with migraine without aura Reading View. Premere ALT+MAIUSC+A per visualizzare la Guida per l'accessibilità.and to identify the clinical-radiological correlations.

Materials and Methods: Estimation of CT and gyrification morphology was performed on the 3D T1 MPRAGE sequence without contrast medium of 73 patients and 49 controls (CTR). Permutational statistical analysis for linear models was carried out to evaluate the significance of the results obtained.

Results: Statistically significant data (p<0.05) are related to the reduction of CT in migraine patients <12 years of age compared to CTR, in particular areas involved are the convolutions: superior frontal, middle frontal, pre-central, post-central of the right hemisphere; superior frontal, post-central, superior parietal lobule, precuneus of the left hemisphere. Regarding the gyrification index, statistically significant differences (p<0.05) were found in migraine patients who presented <5 monthly attacks compared to CTR, in particular altered areas are the lateral and medial orbitofrontal cortex of the left hemisphere.

Conclusion: The areas involved are in the networks of nociception, pain processing and of executive functions, and it"s interesting to note that this result is present only in youngest patients who therefore have a more recent history of disease, suggesting that these alterations may be a true biomarker of migraine.

## P083 Neural signatures associated with treatment response in chronic migraine

### H. Y. Liu^1,2^, K. H. Chou^3,4^, P. L. Lee^3,4^, Y. F. Wang^1,2^, S. P. Chen^1,2,4,5^, K. L. Lai^1,2^, L. L. Pan^4^, W. T. Chen^1,2,4^, S. J. Wang^1,2,4^

#### ^1^Taipei Veterans General Hospital, Department of Neurology, Taipei City, Taiwan; ^2^National Yang Ming Chiao Tung University, School of Medicine, Taipei City, Taiwan; ^3^National Yang Ming Chiao Tung University, Institute of Neuroscience, Taipei City, Taiwan; ^4^National Yang Ming Chiao Tung University, Brain Research Center, Taipei City, Taiwan; ^5^Taipei Veterans General Hospital, Division of Translational Research, Taipei City, Taiwan

##### **Correspondence:** H. Y. Liu

Objective: To identify the neural signatures associated with treatment response in patients with chronic migraine.

Methods: We enrolled 20-60 years old newly diagnosed CM patients. All patients were asked to record the headache diary. Clinical data and a brain MRI were obtained at first visit. Based on diary, headache frequency in the 1st month without treatment was baseline frequency. We gave flunarizine as first line treatment, and topiramate as the second line treatment, and longitudinally followed up the patients for another 3 months. Good response was defined as ≥50% reduction at the 4th month headache frequency compared to the baseline frequency. The others were considered as poor response. Gray matter volume of each brain region was obtained using Freesurfer and Desikan-Killany atlas. Analysis of covariance test was used to compare volumes between patients with different responses.

Results: A total of 78 patients with CM were included in the study. Among them, 41 had good response and 37 had poor response to treatments. Patients with good and poor responses were comparable in age, sex, and scores of hospital anxiety and depression scale and migraine disability assessment. Compared to those with good response, patients with poor response had higher baseline headache frequency, and had smaller volumes of the bilateral medial and lateral orbitofrontal, inferior frontal, and temporal pole, right hippocampus, and left post-central gyrus.

Conclusion: Reduced cortical and subcortical volumes are associated with poor response to treatments in patients with CM.

## P084 Investigation of cortical thickness and volume during spontaneous attacks of migraine without aura: a 3-Tesla MRI study

### F. M. Amin^1^, R. De Icco^2^, M. A. Al-Karagholi^1^, J. Raghava^3^, F. Wolfram^4^, H. Larsson^3^, M. Ashina^1^

#### ^1^Rigshospitalet-Glostrup, Danish Headache Center, Glostrup, Denmark; ^2^University of Pavia, Pavia, Italy; ^3^University of Copenhagen, Copenhagen, Denmark; ^4^Herlev Hospital, Department of Radiology, Herlev, Denmark

##### **Correspondence:** F. M. Amin

Aim and hypothesis: The aim of the present study was to investigate transient changes in cortical thickness during spontaneous migraine attacks. We hypothesized that pain-related cortical area would be affected during the attack compared to an inter-ictal phase.

Methods: 25 patients with migraine without aura underwent 3D T1w imaging on a 3T MRI scanner during spontaneous and untreated migraine attacks. Subsequently, 20 patients were scanned in the inter-ictal phase, while 5 patients did not show up for the inter-ictal scan. Four patients were excluded from the analysis because of bilateral migraine pain and another one patient was excluded due to technical error in the imaging. Imaging analysis was done using FreeSurger. ANOVA was used for statistical analysis and he level of significance was set at *p*=0.025.

Results: Cortical thickness of prefrontal (*p*=0.023), pericalcarine (*p*=0.024), and temporal pole (*p*=0.017) cortices during attack compared to the inter-ictal phase. Cortical volume was reduced in prefrontal (*p*=0.018) and pericalcarine (*p*=0.017) cortices as well as the hippocampus (*p*=0.007). No correlations between these findings and clinical parameters were found.

Conclusion: Spontaneous migraine attacks are accompanied by transient reduced cortical thickness and volume in pain-related areas. The findings constitute a fingerprint of acute pain in migraine patients, which can be used as a biomarker to predict antimigraine treatment (e.g. TMS) effect in future studies.

## P085 Results of occipital nerve stimulation for refractory chronic cluster headache in a third-level hospital

### J. A. Membrilla^1^, I. de Lorenzo^1^, J. Roa^1^, M. Lara-Lara^1^, J. Paz-Solís^2^, A. Gil-Martínez^3^, E. Díez-Tejedor^1^, J. Diaz-de-Terán^1^

#### ^1^La Paz University Hospital, Neurology, Madrid, Spain; ^2^La Paz University Hospital, Neurosurgery, Madrid, Spain; ^3^La Paz University Hospital, Physiotherapy, Madrid, Spain

##### **Correspondence:** J. A. Membrilla

Background and objective: Occipital nerve stimulation (ONS) is a surgical treatment proposed for redractory chronic cluster headache (rCCH). Long-term series assessing its efficacy are scarce. Our objective is to share the outcome of rCCH treated with ONS in our unit.

Methods**:** We designed a retrospective observational study with consecutive sampling, evaluating the follow-up of 22 rCCH patients who underwent ONS. Our endpoint was the weekly attacks reduction. We also evaluated the pain intensity scored by the Visual Analogue Scale (VAS), patient overall perceived improvement and decrease in oral medication intake.

Results**:** After a median follow-up of 5.0 years, patients decreased from a median of 30 weekly attacks to 22.5 at 3 months [p=0.012], 7.5 at 1 year [p=0.006] and 15.0 at the end of follow-up [p=0.023]. The VAS decreased from a median of 10.0 to 9.0 at 3 months [p=0.011] and 7.0 at 1 year [p=0.002] and at the end of follow-up [p=0.002]. 23.5% had an overall perceived improvement of ≥70% at 3 months, 41.2% at 1 year and 27.8% at the end of follow-up. Reducing prophylactic oral medication was possible in 59.1% and it was stopped in 13.6%. Triptan use decreased in all the responder patients and 13.6% stopped its intake. 40.9% presented mild adverse events.

Conclusions**:** Our long-term experience shows that ONS is a beneficial treatment which does not entail serious harm and should be offered as the first option for rCCH management.

## P086 Nocebo Response in Human Models of Migraine: A Systematic Review and Meta-Analysis of Randomized, Double-Blind, Placebo-Controlled, 2-Way Crossover Trials in Migraine without Aura and Healthy Volunteers

### H. Ghanizada

#### Danish Headache Center, Neurology, Taastrup, Denmark

Human models of migraine have been used for the past 30 years to test putative "trigger" molecules and ascertain whether they induce migraine attacks in humans. However, nocebo effects using this model have never been systematically explored.

Objective: To assess the nocebo response rate in randomised clinical trials conducted at the Danish Headache Center.

Methods: Studies of human models of migraine with a randomised, double-blind, placebo-controlled, two-way crossover design that included data on the incidence of migraine attacks or headache after infusion of placebo. A total of 943 articles were screened. Of these, 27 studies met the inclusion criteria (1994 and 2020) and were included in the qualitative and quantitative analysis.

Results: 12 studies reported data for adults with migraine (n=182) whereas 16 studies reported data for healthy volunteers (n=210). For adults with migraine, the pooled incidence of migraine attacks after placebo was 8.1% (95% CI = 2.5-15.5%, I2=50.8%). The pooled incidence of delayed headache was 25.9% (95% CI = 18.5-34.1%, I2=18.9%). For healthy volunteers, the pooled incidence of migraine attacks after placebo was 0.5% (95% CI = 0.0-3.6%, I2=0.0%) while the pooled incidence of delayed headache was 10.5% (95% CI = 4.8%-17.6%, I2=45.2%).

Conclusion: The nocebo response in randomised, placebo-controlled two-way crossover trials with intravenous infusions of placebo in migraine is negligible.

## P087 Evaluating the Utility of Patient-Identified Most Bothersome Symptom for Migraine Research

### R. B. Lipton^1^, P. J. Goadsby^2^, D. W. Dodick^3^, J. S. McGinley^4^, C. R. Houts^4^, R. J. Wirth^4^, S. Kymes^5^, A. Ettrup^6^, O. Østerberg^6^, R. Cady^7^, M. Ashina^8^, D. C. Buse^1,4^

#### ^1^Department of Neurology, Albert Einstein College of Medicine, Bronx, NY, United States; ^2^NIHR-Wellcome Trust King’s Clinical Research Facility, King’s College Hospital, UK & Department of Neurology, University of California, Los Angeles, CA, United States; ^3^Mayo Clinic, Phoenix, AZ, United States; ^4^Vector Psychometric Group, LLC, Chapel Hill, NC, United States; ^5^Lundbeck LLC, Deerfield, IL, United States; ^6^H. Lundbeck A/S, Copenhagen, Denmark; ^7^Lundbeck La Jolla Research Center, San Diego, CA, United States; ^8^Danish Headache Center, Copenhagen, Denmark

##### **Correspondence:** R. B. Lipton

Objective: To evaluate a patient-identified most bothersome symptom (PI-MBS) measure from PROMISE-2 as a patient-reported outcome measure (PROM) for the preventive treatment in chronic migraine.

Methods: Rather than selecting from a predefined list, the PROMISE-2 PI-MBS was captured at screening by querying patients using an open-ended question; responses were recorded and then categorized by investigators. Correlations between PI-MBS, monthly migraine days (MMDs), and PROMs at week 12 were calculated. Linear regression models were used to calculate unique effects of PI-MBS controlling for MMD changes.

Results: Patients (N=1072) reported 23 unique PI-MBS, grouped into 3 classes: pain-related (n=462), cardinal non-pain (n=440), and other (n=170). The 3 classes did not significantly differ in week 12 improvement (*P*>0.05), nor in associations among week 12 PROMs (*P*>0.05), supporting pooling over symptom classes for subsequent analyses. PI-MBS significantly correlated with MMDs and all PROMs (all *P*<0.01); PI-MBS correlated highly with Patient Global Impression of Change (r=0.84) and more strongly with headache-related PROMs (r~0.5) vs general PROMs (r=0.21–0.34). Controlling for MMD changes, PI-MBS improvement provided unique effects on PROMs (all *P*<0.01).

Conclusion: These exploratory analyses suggest that the PROMISE-2 PI-MBS may be a unique measure for assessing patient-centered aspects of burden of disease and benefits of treatment.

## P088 Optimal Acute Treatment Is Associated With Productivity Gains in People With Migraine: Results From the Chronic Migraine Epidemiology and Outcomes (CaMEO) Study

### D. C. Buse^1^, S. J. Nahas^2^, W. Stewart^3^, C. E. Armand^1^, M. L. Reed^4^, K. M. Fanning^4^, A. Manack Adams^5^, R. B. Lipton^1^

#### ^1^Albert Einstein College of Medicine, Bronx, NY, United States; ^2^Thomas Jefferson University, Jefferson Headache Center, Philadelphia, PA, United States; ^3^Medcurio, Oakland, CA, United States; ^4^Vedanta Research, Chapel Hill, NC, United States; ^5^AbbVie, Irvine, CA, United States

##### **Correspondence:** D. C. Buse

Objective: To assess the relationship of acute treatment optimization to lost productive time (LPT) and variation in this relationship by number of monthly headache days (MHDs).

Methods: This analysis included CaMEO survey respondents who met modified migraine criteria consistent with *International Classification of Headache Disorders-3*; were current users of acute prescription medications for migraine; and were employed full-time. LPT was defined as the sum of absenteeism and presenteeism days in the prior 3 months. Acute treatment optimization scores based on the Migraine Treatment Optimization Questionnaire (mTOQ-5) (dichotomous scoring) ranged from optimal (5 positive responses) to very poor (0 positive responses). Headache frequency groups included 0–3, 4–7, 8–14, or ≥15 MHDs.

Results: Of 16,789 respondents with migraine, 2455 (14.6%) met inclusion criteria. Positive responses on the mTOQ-5 were associated with less LPT. This relationship was statistically significant in all MHD groups (linear trend test: *P*≤0.001) except 8–14 MHDs. For example, in the ≥15 MHD group, mean 3-month LPT was 30.4 days in the poor/very poor groups (≤1 positive response), but 7.1 days in the optimal group (5 positive responses). Results were similar after controlling for sociodemographic and headache characteristics.

Conclusion: In people with migraine, suboptimal acute treatment optimization was associated with greater LPT. Optimizing acute treatment may mitigate LPT and reduce indirect costs.

## P089 Long-term Safety and Tolerability of Atogepant 60 mg Following Once Daily Dosing Over 1 Year for the Preventive Treatment of Migraine

### M. Ashina^1^, S. J. Tepper^2^, U. Reuter^3^, A. M. Blumenfeld^4^, S. Hutchinson^5^, J. Xia^6^, R. Miceli^6^, L. Severt^6^, M. Finnegan^6^, J. M. Trugman^6^

#### ^1^Danish Headache Center, University of Copenhagen, Glostrup, Denmark; ^2^Geisel School of Medicine at Dartmouth, Hanover, NH, United States; ^3^Charité University Hospital Berlin, Berlin, Germany; ^4^Headache Center of Southern California, Carlsbad, CA, United States; ^5^Orange County Migraine and Headache Center, Irvine, CA, United States; ^6^AbbVie, Madison, NJ, United States

##### **Correspondence:** M. Ashina

To assess the safety and tolerability of atogepant, an oral, calcitonin gene-related peptide (CGRP) receptor antagonist in development for migraine preventive treatment, once daily over 1 year.

Multicenter, open-label trial (NCT03700320). Adults with migraine were randomized 5:2 to atogepant (ato) or oral standard-of-care (SOC) migraine prevention.

744 randomized participants (pts; n=546 atogepant), 739 safety population pts (n=543 ato). Adverse events (AEs) were reported by 67.0% of ato pts; 18.0% of pts had AEs considered related to ato by the investigator. Most commonly reported AEs (≥5% of pts) following ato treatment were upper respiratory tract infection (10.3%), constipation (7.2%), nausea (6.3%), and urinary tract infection (5.2%). 4.4% of ato pts reported serious AEs and included a broad variety of common medical conditions; no event was seen in ≥1 pt and none were ato-related. Two deaths were reported in pts treated with ato (victim of homicide; group A beta-hemolytic streptococcal sepsis [toxic shock syndrome]); both were considered not related. 5.7% of ato pts discontinued due to AEs. Alanine aminotransferase/aspartate aminotransferase (ALT/AST) levels ≥3Xs the upper limit of normal were reported for 2.4% of ato pts (n=13/531) and 3.2% for SOC pts (n=6/190). No cases of potential Hy"s Law were reported.

Long-term, once-daily use of atogepant for the preventive treatment of migraine over 1 year was safe and well-tolerated with no safety concerns identified.

## P090 Can migraine features, hypersensitivity or cervical musculoskeletal dysfunction explain neck disability in migraine?

### Z. Liang, L. Thomas, G. Jull, J. Treleaven

#### The University of Queensland, School of Health and Rehabilitation Sciences, St Lucia, Australia

##### **Correspondence:** Z. Liang

Objective: To investigate the predictors of neck disability in migraine and determine if scores from the Neck Disability Index (NDI) versions (NDI-physical, NDI-mental, NDI-8, NDI-5) are associated with cervical musculoskeletal dysfunction.

Methods: Migraineurs with neck pain (n=104) were assessed on migraine and neck pain features, the Neck Disability Index (NDI), Headache Impact Test (HIT6), Allodynia Symptom Checklist (ASC12) and pressure pain thresholds (PPTs). Cervical dysfunction was previously identified in 45 but not in 59 of these individuals. NDI score was regressed on migraine features, HIT-6, total PPT, ASC12, while accounting for neck pain features and the presence or not of cervical dysfunction. Presence of cervical dysfunction was regressed on the scores of NDI versions.

Results: Neck pain intensity (B=2.26, p<0.001) and frequency (B=5.08, p<0.001), the ASC12 (B=0.71, p=0.018) and HIT6 scores (B=0.42, p=0.049) were significantly predictive of NDI score. Presence of cervical dysfunction and other variables were not predictive of NDI score. No version of NDI was associated with cervical dysfunction (NDI-physical: Χ^2^=0.038, df=1, p=0.85, NDI-mental: Χ^2^=0.246, df=1, p=0.62, NDI-8: Χ^2^=0.010, df=1, p=0.92, NDI-5: Χ^2^=0.274, df=1, p=0.60).

Conclusion: The NDI is a complex measure of neck disability in migraine that is related to headache disability and allodynia, but not necessarily indicative of cervical dysfunction.

## P091 Transcranial sonography in migraine: periaqueductal gray matter (PAG)

### N. Ghiotto, E. Guaschino, R. De Icco, E. Cecconi, C. Tassorelli, G. Sances

#### IRCCS Mondino Foundation, Pavia, Pavia, Italy

##### **Correspondence:** N. Ghiotto

Objective: Periaqueductal gray (PAG) plays an important role in the modulation of descending pain control. Previous MRI studies showed that increased PAG iron levels in both episodic and chronic migraine patients correlated with disease duration. Transcranial sonography (TCS) is an imaging technique that allows visualization of heavy metals in the brain parenchyma as an area of hyperecogenicity. Our aim was to investigate hyperechogenicity of PAG in migraine patients.

Methods: We investigated with TCS 13 patients with episodic migraine (EM), 15 with chronic migraine and medication overuse headache (CM+MOH) and 10 Healthy Controls (HCs). The area of PAG hypercogenicity visualized through the transtemporal window was measured semiautomatically on each side and then calculated as a mean value.

Results: PAG hyperechogenicity was visualized in 100% of the CM+MOH patients, 69% of EM patients and 44% of HCs (p <0.001). No significant difference was found in the hyperechogenic PAG area among the three groups (p=0.295). However PAG hyperecogenicity area correlated with disease duration (p<0.023), pain intensity (p<0.031) and scores of the HIT-6 scale (p<0.043).

Conclusion: These preliminary data suggest that repeated migraine attacks may lead over time to increased oxidative stress and free radicals release, contributing to secondary damage, contextual hyperaemia, and iron deposit in the PAG.

## P092 Association of white matter hyperintensities with different migraine subtypes

### L. Dobrynina^1^, A. Suslina^2^, M. Gubanova^1^, A. Belopasova^1^, A. Sergeeva^2^, S. Evers^3^, M. Krotenkova^2^

#### ^1^Research Center of Neurology, 3rd Neurological Department, Moscow, Russian Federation; ^2^Research Center of Neurology, Neuroradiology Department, Moscow, Russian Federation; ^3^University of Münster, Faculty of Medicine, Münster, Germany

##### **Correspondence:** M. Gubanova

Introduction: White matter hyperintensities (WMH) are frequently detected in migraine patients, however, their significance remains uncertain. Objective: To evaluate the WMH pattern of different migraine subtypes

Methods: A brain MRI (Siemens, Germany, 3T) was performed in 92 otherwise healthy migraine patients with no vascular risk factors (73 females, mean age 34.6±8.9; 61 episodic migraine, 31 chronic migraine, 36 migraine with aura, 56 migraine without aura).

Results: The prevalence of WMH in different types of migraine was similar and ranged from 38.7% to 44.4%. The distribution of focal WMH decreased from the frontal to the parietal and to the temporal lobe. In most cases, WMH were located in the juxtacortical and/or deep white matter; only 2 patients had periventricular WMH. WMH appeared as round or slightly elongated foci with a median size of 2.5 mm [1.5; 3]. Total number, size and prevalence of WMH by lobes and white matter regions were similar between groups, and no interaction with age or sex was found.

Conclusion: Patients with different subtypes of migraine and without vascular risk factors have a similar pattern of WMH and no subclinical infarctions and microbleedings, which indicates the low prognostic value of WMH in identifying a specific migraine subtype or vascular complications of migraine. WMH pattern may be used to differentiate migraine as a primary disorder and other disorders with migraine-like headache and WMH.

## P093 Craniocervical exercises reduce disability in patients with migraine

### M. Tedeschi Benatto^1^, L. Lima Florencio^2^, M. Mendes Bragatto^1^, F. Dach^3^, D. Bevilaqua-Grossi^1^

#### ^1^University of São Paulo, Department of Health Sciences – Ribeirão Preto Medical School, Ribeirão Preto, Brazil; ^2^Universidad Rey Juan Carlos, Department of Physical Therapy, Occupational Therapy, Rehabilitation and Physical Medicine, Alcorcón, Spain; ^3^University of São Paulo, Department of Neurosciences and Behavioral Sciences, Ribeirão Preto, Brazil

##### **Correspondence:** M. Tedeschi Benatto

Objective: to verify if a craniocervical exercises protocol was able to reduce the disability caused by migraine.

Methods: thirty-three women with a diagnosis of migraine with a mean age of 32.5 (SD=8.5) years and a frequency of 9.8 (SD=7.6) days/month were included. All volunteers signed an informed consent form and answered the Migraine Disability Assessment (MIDAS) questionnaire. The craniocervical exercise protocol started after the initial data collection and lasted for 8 weeks. The protocol consisted of active exercises for the deep and superficial flexor and extensor muscles of the cervical spine. The volunteers had once weekly sessions with the physiotherapist to progress the exercises. At the end of the treatment, the MIDAS questionnaire was applied again. For the comparison between pre and post treatment, a paired Student"s t test was used. SPSS version 20.0 software was used and a significance level of 0.05 was adopted. The study was approved by the local ethics committee (6146/2016).

Results: we observed a significant reduction of 14.6 (SD=29.5; 95% CI=4.2 – 25.1; p=0.008) points in the total score of the MIDAS questionnaire after the 8-week treatment. A reduction of 5 points in MIDAS questionnaire is considered a clinically important change and 21 (63.6%) volunteers showed a reduction of ≥5 points in questionnaire.

Conclusion: a craniocervical exercises protocol lasting 8 weeks had a positive effect in reducing the disability in patients with migraine.

## P094 Pharmacological characterisation of mouse calcitonin and calcitonin receptor-like receptors reveals differences compared to human receptors

### M. Garelja^1^, C. Walker^2,3^, D. Hay^1,3^

#### ^1^University of Otago, Pharmacology and Toxicology, Dunedin, New Zealand; ^2^University of Auckland, School of BIological Sciences, Auckland, New Zealand; ^3^Maurice Wilkins Centre for Molecular Biodiscovery, Auckland, New Zealand

##### **Correspondence:** M. Garelja

Objective: The calcitonin receptor family comprises multiple receptors and multiple endogenous peptide ligands, including calcitonin gene-related peptide (CGRP) and amylin. CGRP has been successfully targeted for the treatment of migraine but mechanistic understanding of exactly how CGRP contributes to migraine is still poorly resolved. Mouse models are commonly used to probe CGRP and related peptide biology. However, the pharmacology of mouse calcitonin family receptors is poorly characterised, creating challenges for data interpretation and translation of pre-clinical findings to humans. We therefore investigated the pharmacology of mouse calcitonin family receptors.

Methods: Plasmids encoding mouse receptors were transfected into Cos7 cells. Cells were stimulated with agonists with and without antagonists and cAMP production measured.

Results: The pharmacology of these receptors differed between humans and mice, with mouse receptors generally displaying less discrimination between peptides. This was most apparent for receptors that included the calcitonin receptor. Overall, CGRP had nanomolar potency at four mouse receptors.

Conclusion: Our findings are a framework for interpreting pre-clinical findings. The data reveal challenges in interpreting which receptor may underlie an effect in pre-clinical models, and thus translation of findings from mice to humans. The work also highlights the need for more selective ligands that can differentiate between these receptors.

## P095 Registry-based, Prospective, Observational Studies to Assess Maternal, Fetal, and Infant Outcomes Following Exposure to Migraine Treatments, Including Galcanezumab

### S. Ephross^1^, K. Schroeder^2^, N. Kellier-Steele^2^, A. Graves^1^, M. Bangs^2^, R. Nichols^2^, L. Do^2^, P. Hauck^2^, J. Brandes^3^

#### ^1^Syneos Health, Morrisville, NC, United States; ^2^Eli Lilly and Company, Indiana, IN, United States; ^3^Nashville Neuroscience Group, Vanderbilt University, Nashville, TN, United States

##### **Correspondence:** J. Brandes

Objective: Compare maternal, fetal and infant outcomes among pregnant women with migraine exposed to galcanezumab to those exposed or not exposed to other migraine medications. There is a need to study utilization and safety of these medications before/during pregnancy since data on outcomes of pregnancies exposed to galcanezumab is limited.

Methods: This multidrug pregnancy registry will enroll women with migraine exposed to galcanezumab up to 5 half-lives before/during pregnancy. Pregnant women with migraine (exposed or not exposed to other migraine medications) will be enrolled into comparator groups. Eligible women may enroll or be enrolled by their Health Care Provider by calling the phone number/visiting the website listed in the US Package Insert. Information on mother and fetus/infant (eg, demographics/medical history/exposures/outcomes) will be collected at multiple time points during pregnancy and to 1 year post delivery.

Results: The primary outcome assessed in this pregnancy registry is major congenital malformations. Additional maternal, fetal and infant outcomes (to 1 year of age) will be evaluated.

Conclusions: Real-world studies are needed to evaluate utilization and safety of new migraine medication exposures in pregnancy. This registry is part of a larger effort towards this goal. Sufficient enrollment of pregnant women will enable execution of two comparative safety studies using this registry.

## P096 Mindfulness-based Stress Reduction (MBSR) vs. HA Education: A Randomized Clinical Trial Showing Mindfulness Treats Total Migraine Burden

### R. E. Wells^1^, N. O’Connell^1^, C. R. Pierce^1^, P. Estave^1^, D. B. Penzien^1^, E. Loder^2^, F. Zeidan^3^, T. T. Houle^4^

#### ^1^Wake Forest Baptist, Winston Salem, NC, United States; ^2^Brigham and Women's Hospital, Boston, MA, United States; ^3^University of California-San Diego, San Diego, CA, United States; ^4^Massachusetts General Hospital, Boston, MA, United States

##### **Correspondence:** R. E. Wells

Objective**:** Determine if mindfulness-based stress reduction (MBSR) improves migraine outcomes compared to Headache (HA) Education.

Methods: Randomized clinical trial in adults with 4-20 migraine days/month comparing MBSR vs. HA Education (n=89), both delivered in eight weekly classes. Participants were blinded to active vs. comparator group assignments, and PI/data analysts to group assignments.

Results**:** Participants in both groups had fewer migraine days at 12 weeks (MBSR: -1.6 migraine days/month; 95% CI: [-0.7, -2.5]; HA Education -2.0; [-1.1, -2.9]), without group differences (p=0.51). MBSR participants, compared to HA Education, had improvements from baseline at all follow-up time points on measures of disability (5.92 (95% CI 2.8, 9.0) p<0.001); quality of life (5.1 (1.2, 8.9) p=0.01); self-efficacy (8.2 (0.3, 16.1, p=0.04); pain catastrophizing (5.8 (2.9, 8.8), p<0.001); depression scores (1.6 (0.4, 2.7) p=0.008), and decreased experimentally induced pain intensity and unpleasantness (p= 0.004 and 0.005, respectively, at 36 weeks). One reported adverse event was deemed unrelated to study protocol.

Conclusion: Both MBSR and HA Education improved migraine frequency. MBSR also had clinically meaningful improvements in disability, quality of life, self-efficacy, pain-catastrophizing, and depression out to 36 weeks, with decreased experimentally induced pain suggesting a potential shift in pain appraisal. MBSR may safely help treat total migraine burden.

## P097 Switching Associated with Initiation of Calcitonin Gene-Related Peptide (CGRP) Monoclonal Antibodies (mAbs) Versus non-CGRP mAb Treatments for Prevention of Migraine

### S. A. Foster^1^, J. Manjelievskaia^2^, J. H. Ford^1^, W. Ye^1^, A. Perry^1^, K. Schuh^1^, R. Wenzel^1^

#### ^1^Eli Lilly and Company, Indiana, IN, United States; ^2^IBM Watson Health, Cambrigde, MA, United States

##### **Correspondence:** R. Wenzel

Objective: Comparing switching patterns in patients with migraine initiating CGRP mAbs vs non-CGRP mAbs.

Methods: This retrospective observational cohort study used administrative claims databases. Adults with ≥1 claim (first claim=index) for CGRP mAb (galcanezumab/ erenumab/fremanezumab) or non-CGRP mAb treatment (antidepressants/beta-blockers//neurotoxin) May 2018-June 2019 with continuous enrollment in medical and pharmacy benefits for 12 and 6 months pre-/post-index were included. Chi-square and Student"s t-tests were conducted on study measures.

Results: 12681 CGRP mAb (mean (SD) age 44.3(11.6); 87% female) and 21474 non-CGRP mAb patients (mean (SD) age 41(12.5); 85% female) met criteria. Top 2 prescriber specialties were neurologists (CGRP mAbs: 30%; non-CGRP mAbs: 25.8%) and primary care providers (CGRP mAbs: 25.5%; non-CGRP mAbs: 43%) (p<0.001). Over 6 months (post-index), 31.5% CGRP mAb and 60.2% non-CGRP mAb initiators discontinued therapy(p<0.001). Of those who discontinued, 40.7% CGRP mAb and 17.9% non-CGRP-mAb initiators switched to another therapy(p<0.001). For those who switched, average mean (SD) time to switch after index drug initiation was 104.2 (39.9) and, 97.2 (42.4) days for CGRP mAb and non-CGRP-mAb patients(p<0.001).

Conclusion: Over 6-month post-index period, compared to non-CGRP mAb initiators, CGRP mAb initiators were less likely to discontinue therapy; those who discontinued were more likely to switch; took longer time to switch.

## P099 Real-world Evidence for the Safety and Efficacy of CGRP Monoclonal Antibody Therapy Added to OnabotulinumtoxinA Treatment for Migraine Prevention in Adult Patients with Chronic Migraine

### L. Mechtler^1^, N. Saikali^1^, J. McVige^1^, O. Hughes^2^, A. Traut^3^, A. Adams^3^

#### ^1^DENT Neurologic Institute, Buffalo, NY, United States; ^2^ICON plc, Boston, MA, United States; ^3^AbbVie, Irvine, CA, United States

##### **Correspondence:** L. Mechtler

Objective: Evaluate the real-world safety and efficacy of adding a calcitonin gene-related peptide (CGRP) monoclonal antibody (mAb) to onabotulinumtoxinA (onabotA) for chronic migraine (CM).

Methods: Retrospective, longitudinal chart review from adults (≥18 years) with CM treated with ≥2 consecutive cycles of onabotA before ≥1 month of onabotA + mAb combination therapy. Safety and efficacy (monthly headache days [MHD]) were recorded at first mAb prescription (index) and up to 4 onabotA visits ~3, 6, 9, and 12 months post-index.

Results: Charts were collected for 192 patients; 149 met eligibility criteria. 57% of patients were prescribed erenumab, 42.3% fremanezumab, and 0.7% galcanezumab. Mean (SD) MHD were 20.3 (6.6) prior to onabotA and 14.0 (6.9) prior to the addition of a mAb. There were significant reductions in MHD at the first visit (~3 month) and at all subsequent visits (**Figure 1**). OnabotA was discontinued by 42 (28.2%) patients and a mAb by 50 (33.6%) patients. Most common reasons for discontinuing either treatment were lack of reimbursement (40%) and lack of effect (34%); 14% discontinued a mAb and none onabotA due to safety/tolerability. Adverse events (AEs) were reported by 18 patients (12.1%); no serious AEs were reported.

Conclusions: In this real-world study, onabotA was effective at reducing MHD and the addition of a CGRP mAb was well tolerated and associated with incremental reductions in MHD for those on the combination. No new safety signals were identified.

**Fig. 1 (abstract P099).**
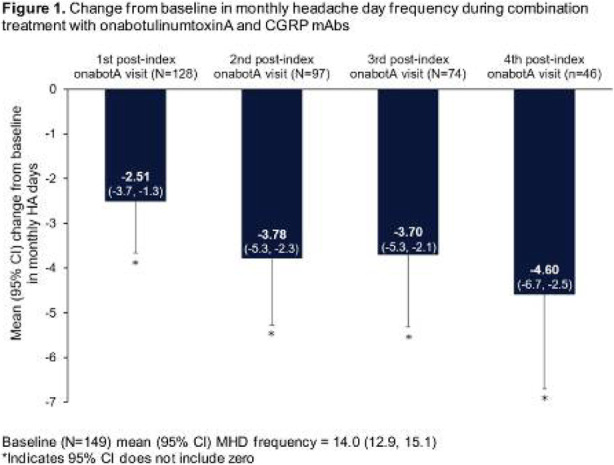
See text for description

## P0100 Effect of Occipital Nerve Stimulation (ONS) on the Orbicularis Oculi Reflex Triggered by a Standardized Air Flow in Patients with Chronic Migraine—A Prospective, Randomized, Interventional Study

### C. Göbel, A. Heinze, S. Karstedt, S. Clasen, H. Göbel

#### Kiel Headache and Pain Centre, Kiel, Germany

##### **Correspondence:** C. Göbel

Introduction: Occipital nerve stimulation (ONS) is a specific form of peripheral neuromodulation used in the treatment of chronic pain disorders. A particular field of application is in the therapy of treatment-refractory headaches, especially of chronic migraine. The precise mode of action is unknown. It is presumed that central and peripheral sensitization are reduced in patients with chronic headache. The aim of this study was to examine the effect of ONS on pain-modulatory mechanisms in the trigeminocervical area in patients with chronic migraine.

Methods: In a balanced repeated measurements design in eight patients with chronic migraine with and without active ONS, we analyzed which effects ONS had on the orbicularis oculi reflex dynamically elicited by corneal air flow.

Results: The orbicularis oculi reflex in active ONS (7.38 ± 20.14 eyelid closures/minute) compared to inactive ONS (18.73 ± 14.30 eyelid closures/minute) is significantly reduced (*p* = 0.021).

Conclusions: The results show that under active ONS compared to inactive ONS in patients with chronic migraine, the orbicularis oculi reflex, dynamically triggered by a standardized air flow, is significantly reduced. This suggests that ONS is able to directly counteract the trigeminally mediated central sensitization in chronic migraine and protectively reduce the effects of aversive peripheral stimulation.

## P0101 Modulation of cortical networks functional connectivity in migraine patients by repetitive transcranial magnetic stimulation

### K. Markin^1^, D. Frunza^1^, D. Tarumov^1^, M. Sorokin^2^, A. Trufanov^1^

#### ^1^Military Medical Academy, St. Petersburg, Russian Federation; ^2^V.M. Bekhterev National Medical Research Center for Psychiatry and Neurology, St. Petersburg, Russian Federation

##### **Correspondence:** K. Markin

Resting-state fMRI studies allow to objectify the effect of treatment. We aimed to determine the differences between large-scale networks functional connectivity (FC) in migraine patients due to repetitive transcranial magnetic stimulation (rTMS) course and their correlation with clinical features.

19 patients with migraine without aura (39.8±11.1 years; 3 men) underwent a 5-day course of rTMS with a 10 Hz frequency of the projection of the ventrolateral prefrontal cortex and trigeminal nerve branches bilaterally. Before and after the course of TMS, each patient was offered a test battery (Numerical Pain Rating Scale, Migraine Disability Assessment Questionnaire, Hospital Anxiety and Depression Scale, Leeds Addiction Questionnaire) and underwent fMRI. FC changes was carried out with paired t-test based on the 10-factors group independent component analysis (ICA) results with the pFDR-correction.

We founded increased FC in Default Mode Network (DMN), decreased FC in SensoriMotor Network, and both decreased and increased FC in Salience Network (Fig. 1). Responders (15) differed from nonresponders in that the higher the reduction of the severity of headache, the increased the strength within DMN connectivity. The reduction of drug dependence was correlated with increased FC between DMN and Visual Network (Fig. 2).

Considering the results of previous neuroimaging studies based on the ICA, our data may indicate a partial restoration of FC alterations as a result of TMS therapy.

**Fig. 1 (abstract P0101).**
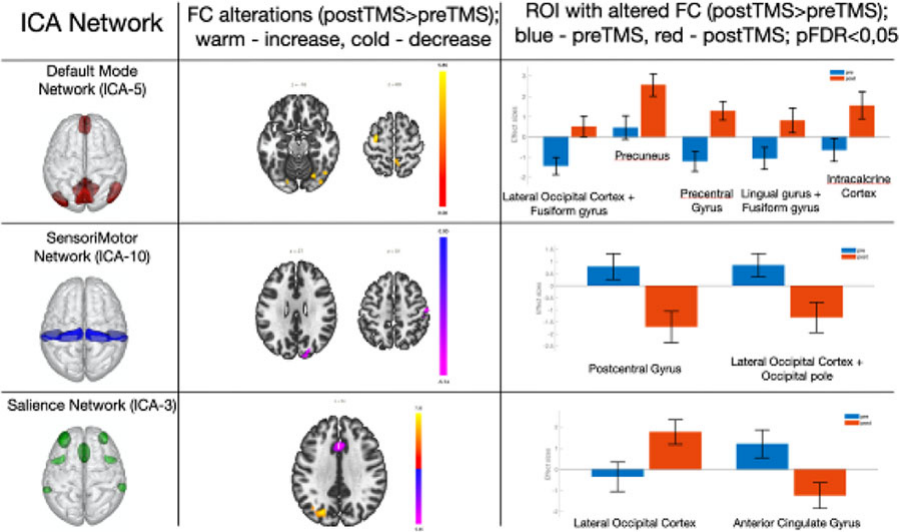
See text for description

**Fig. 2 (abstract P0101).**
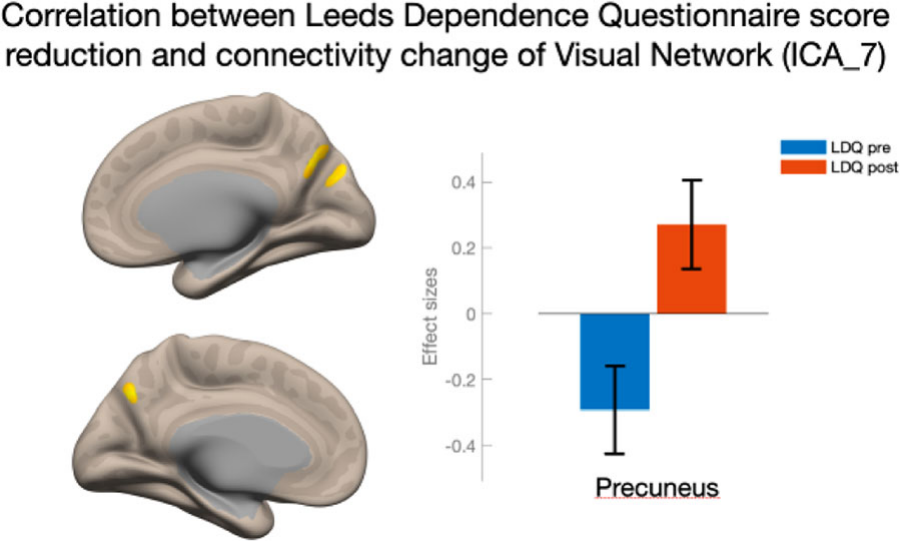
See text for description

## P0102 Abnormal multisensory integration in migraine: a study of concurrent visual and somatosensory stimulation

### G. Sebastianelli, C. Abagnale, F. Casillo, M. Serrao, G. Coppola

#### Sapienza University of Rome, Latina, Italy

##### **Correspondence:** G. Sebastianelli

Background: Merging of sensory information is an important process for all species. Co-application of bi-modal stimulations results in greater neural activation than the sum of each unimodal stimuli delivered independently. Here, we have tested how the multisensory integration take place in episodic migraine patients (MO), by evaluating the potential ability of bi-modal stimulations to affect the mechanisms of habituation.

Methods: We recorded somatosensory evoked potentials (SSEPs) in 20 healthy volunteers (HVs) and in 21 patients with MO before, during, and after simultaneous visual stimulation. 600 sweeps were acquired for each condition and partitioned in 2 blocks of 100 sweeps for the calculation of habituation as the slope of the regression line between the 1st and the 2nd block of averaged N20-P25 SSEP amplitude response.

Results: In both groups the visuo-somesthetic stimulation changed the SSEP N20-P25 habituation seen at baseline, but in opposite way. In HVs the concurrent stimulation provoked a significant loss of habituation. In patients with MO, who had a deficient habituation at baseline, the simultaneous stimulation produced a significant amplitude decrement.

Conclusion: There is ample scientific evidence which sustain that MO patients have an atypical way of processing unimodal information. Our result suggests that also the multisensory integration is affected, and this process, by modifying cortical responsivity, could influence the migraine cycle.

## P0103 The association of cortical thickness at MRI with clinical presentation of migraine aura: a whole brain surface-based morphometry study

### C. Abagnale^1^, A. Di Renzo^2^, E. Tinelli^3^, B. Petolicchio^3^, M. Serrao^1^, V. Parisi^2,4^, M. Fiorelli^3^, F. Caramia^3^, V. Di Piero^3^, G. Coppola^1^

#### ^1^Sapienza, University of Rome, Department of Medico-Surgical Sciences and Biotechnologies, Latina, Italy; ^2^IRCCS-Fondazione Bietti, Rome, Italy; ^3^Sapienza University of Rome, Human neuosciences, Rome, Italy; ^4^IRCCS-Fondazione Bietiti, Rome, Germany

##### **Correspondence:** C. Abagnale

Background: We were aimed to study intracerebral white matter fiber bundles, using a tract-based spatial statistics (TBSS) analysis of diffusion tensor imaging (DTI), and grey matter cortical thickness from structural magnetic resonance imaging data in migraine patients with pure visual auras (MA), and in patients with complex neurological auras (MA+), i.e. with the addition of at least one of sensory and language symptoms.

Methods: 3T MRI data from 20 patients with MA and 15 with MA+ were collected and compared with data from 19 healthy controls (HCs). For everyone, we performed DTI to calculate diffusivity metrics and we obtained cortical thickness maps from structural MRI.

Results: TBSS showed no significant differences in the diffusivity maps between both patients" groups and HCs. As compared to HCs, both patients with MA and MA+ significantly showed tinner temporal cortices, frontal areas, insula, post-central area, and primary and associative visual areas. In the MA group, the high-level visual-information-processing areas, including lingual gyrus, were thicker, in contrast to the MA+ group where they were thinner than in HCs.

Discussion: These findings suggest that clinical heterogeneity of migraine with aura is associated with common cortical surface morphological features as well as with an opposite morphological involvement of the high-level visual-information-processing areas.

## P0104 Erenumab effects at the level of the caudal trigeminal nucleus and on the somatosensory cortex

### F. Casillo^1^, G. Sebastianelli^2^, C. Abagnale^2^, F. Pierelli^2^, G. Coppola^2^

#### ^1^Sapienza University of Rome, Latina, Italy; ^2^Sapienza University of Rome, Department of Medico-Surgical Sciences and Biotechnologies, Latina, Italy

##### **Correspondence:** F. Casillo

Background: Erenumab is a monoclonal antibody against CGRP receptor approved as a prophylactic treatment of migraine. It is not yet clear if its neurophysiological effects are confined to the peripheral trigeminal system or also occur at the cortical level. This study assessed the neurophysiological effects of the drug in migrainous patients unresponsive to ≥2 prophylactic treatments.

Methods: We prospectively enrolled 20 patients. For each patient we recorded the blink reflex (nBR), after stimulation of the right supraorbital nerve with a nociception specific concentric electrode, and the non-noxious somatosensory evoked potentials (SSEPs) after repetitive electrical stimulation of the median nerve. We measured nBR R2 area-under-the-curve (AUC) and habituation, and SSEP N20-P25 amplitude and habituation. Neurophysiological measurements were recorded before and at month-1 (T1) and month-2 (T2) before each monthly erenumab injection.

Results: At T2, erenumab reduced the severity of headache, the mean monthly headache days and tablet intake (all p=<0.001). Compared to baseline, the nBR AUC was significantly reduced at T1. An increase in SSEP habituation, was noted at T1 and, more so, at T2 compared to the baseline (slope baseline =+0.103, T1 =-0.167, T2 =-0.229, p<0.05).

Conclusion: The results of our study show that the clinical improvement induced by Erenumab can be attributed to neurophysiological changes occurring at both the brainstem and cortical levels.

## P0105 Treatment failure with anti-CGRP therapy: should we discontinue the treatment or switch it?

### C. Nieves Castellanos, M. Losada López, M. I. Fabrich Marín, J. Pérez García, S. Díaz Insa

#### Hospital Universitari i Politécnic la Fe de Valencia, Valencia, Spain

##### **Correspondence:** C. Nieves Castellanos

Objective: Since monoclonal antibodies anti-CGRP or its receptor (a-CGRP mAbs) are available, we have been using them in our Headache Unit. Using a second a-CGRP mAbs after the failure of the first one could be interesting.

Methods: We have carried out a real-life study collecting the patients with refractory migraine with a-CGRP mAbs since January 2020. We initiated 220 patients. 52 patients switch the a-CGRP mAb: 37 patients after 3 months with the first one, 8 after 6 months and 7 after 9 months. We present the data (migraine days (MD), headache days (HD)) and scales (HIT-6, MIDAS, MSQ, pain catastrophizing scale (PCS)) and willing of continue with the treatment, collected before and 3 months after the switch.

Results: Collected data from 52 patients. They had failure an average of 6 preventive treatment. Before the switch: 24,2 HD, 22 MD, 98,7 points in MIDAS. 3 months after the switch with a second a-CGRP mAb, 46,15% wanted to continue with the treatment. These patients (n=24) reduced MD from 22 to 16,6 days, use of symptomatic treatment was reduced from 20 days per month to 14,6. The results of the scales are: MIDAS was reduced from 105,4 points to 83,4 points (39,37%), HIT-6 was reduced 4,33 points, and MSQ increased an average of 9,5 points. The 25% responder rate was 36%. 2 patients reduced more than 75% of MD.

## Conclusion: It could be interesting to switch the a-CHGRP mAb after a failure because a percentage of patients improve their rate of MD and quality of life.

## P0106 Efficacy and Safety of Eptinezumab in Patients With Migraine and Self-Reported Aura: Post Hoc Analysis PROMISE-1 and PROMISE-2

### M. Ashina^1^, P. McAllister^2^, R. Cady^3^, J. Hirman^4^, A. Ettrup^5^

#### ^1^Danish Headache Center, Copenhagen, Denmark; ^2^New England Institute for Neurology and Headache, Stamford, CT, United States; ^3^Lundbeck La Jolla Research Center, San Diego, CA, United States; ^4^Pacific Northwest Statistical Consulting, Inc., Woodinville, WA, United States; ^5^H. Lundbeck A/S, Copenhagen, Denmark

##### **Correspondence:** M. Ashina

Objective: To evaluate the efficacy and safety of eptinezumab for migraine prevention in patients with self-reported aura.

Methods: Patients with episodic migraine (EM; PROMISE-1) or chronic migraine (CM; PROMISE-2) and self-reported migraine with aura at screening were included. Symptoms constituting aura were discussed with/explained by investigators to patients to improve accuracy of future symptom capture. In both studies, the primary efficacy outcome was the reduction in monthly migraine days (MMDs) over weeks 1–12.

Results: Of patients with EM, ~75% reported a history of experiencing aura at screening (eptinezumab 100mg, 167/221; eptinezumab 300mg, 173/222; placebo, 167/222); of patients with CM, ~35% reported a history of aura (100mg, 115/356; 300mg, 173/350; placebo, 167/366). In EM patients with aura, mean changes from baseline in MMDs were –4.0 (100mg) and –4.2 (300mg) vs –3.1 (placebo). In CM patients with aura, mean changes were –7.1 (100mg) and –7.6 (300mg) vs –6.0 (placebo). These changes were comparable to the total PROMISE-1 and PROMISE-2 populations. A similar percentage of patients experienced adverse events across treatment groups (100mg, 56.0%; 300mg, 57.4%; placebo, 55.4%).

Conclusion: This subgroup analysis showed efficacy and safety with eptinezumab vs placebo in patients with self-reported migraine with aura, consistent with the full populations, demonstrating the clinical utility of eptinezumab treatment in this subpopulation of patients with migraine.

## P0107 Tardive Dyskinesia Associated with Dopamine Blocking Agents Used in Migraine

### W. Gryc^1^, N. Murinova^2^, D. Krashin^2^, A. Argyropoulos^3^

#### ^1^University of Washington, Neurology, Seattle, WA, United States; ^2^Puget Sound VA, Pain and Psychiatry, Seattle, WA, United States; ^3^Puget Sound VA, Psychiatry, Seattle, WA, United States

##### **Correspondence:** N. Murinova

Background: Anti-nausea medications such as metoclopramide are commonly used in migraine management for control of nausea and emesis. The most commonly used anti-emetics are dopamine blocking agents and have the potential to cause movement disorders that are disabling and permanent.

Design/Methods: The Leaf research database was used to analyze drug induced tardive dyskinesia (TD) patients and migraine n a large medical database. Patients were analyzed based on their use of dopamine-blocking anti-emetics.

Results: 495 patients had subacute dyskinesia and of those, 54 patients had migraine diagnosis. Out of 66,086 patients with migraine, 22,795 used metoclopramide, 2,011 specifically for migraine. 47 patients had both migraine and subacute drug induced dyskinesia. Out of 19 patients with previous use of metoclopramide, 17 carried diagnosis of TD, 5 presumed due to metoclopramide use. Upon detailed chart review only one patient with migraine met TD criteria from use of metoclopramide used for indigestion for 1.5 years.

Conclusions: We conclude that tardive dyskinesia (TD) from metoclopramide in migraine patients is very rare. In analysis of our database of close to 5 million unique patients and detailed chart review, only one patient met the diagnostic criteria for TD metoclopramide use. Dosage is the primary risk for TD in genetically susceptible individuals. Headache providers should be aware of the risk of TD with prolonged anti-emetic therapy.

## P0108 Consecutive Migraine-free Days with Fremanezumab Treatment: Results of the Double-blind, Placebo-controlled FOCUS Study

### H. C. Diener^1^, J. M. Cohen^2^, M. Galic^3^, V. Ramirez Campos^2^, S. Barash^2^, X. Ning^2^, L. Pazdera^4^

#### ^1^Universitätsklinikum Essen, Essen, Germany; ^2^Teva Branded Pharmaceutical Products R&D, Inc., West Chester, PA, United States; ^3^Teva Pharmaceuticals, Amsterdam, Netherlands; ^4^Vestra Clinics, Rychnov nad Kněžnou, Czech Republic

##### **Correspondence:** H. C. Diener

Objective: Use of migraine preventive medication may reduce migraine frequency and increase number of migraine-free days (MFD). Fremanezumab, a fully-humanized monoclonal antibody (IgG2Δa) that selectively targets calcitonin gene-related peptide (CGRP), demonstrated efficacy for migraine prevention in patients with documented inadequate response to 2-4 prior migraine preventive medication classes in the phase 3b FOCUS study. This post hoc analysis evaluated maximum number of consecutive MFD for patients in the FOCUS study.

Methods: For 12 weeks of double-blind treatment in FOCUS, eligible patients were randomized (1:1:1) to quarterly fremanezumab, monthly fremanezumab, or matched placebo. Change from baseline (BL) in monthly average maximum number of consecutive MFD was evaluated.

Results: 838 patients were randomized. At BL, mean (SD) numbers of maximum consecutive MFD were comparable across treatment groups (quarterly fremanezumab, 5.1 [2.84]; monthly fremanezumab, 5.1 [3.11]; placebo, 4.8 [3.03]). Increases from BL in consecutive MFD during 12 weeks were significantly higher for fremanezumab (least-squares mean [SE] change from baseline during 12 weeks: quarterly, 8.3 [0.82]; monthly, 9.6 [0.81]) versus placebo (4.0 [0.81]; both *P*<0.0001).

Conclusions: In migraine patients with inadequate response to 2-4 prior migraine preventive medication classes, patients receiving quarterly or monthly fremanezumab had significantly more consecutive MFD versus placebo during 12 weeks.

## P0109 Efficacy of Galcanezumab for the treatment of migraine in Korea: the first real-world data from an Asian country

### S. Kwon^1^, Y. E. Gil^2^, M. J. Lee^2^

#### ^1^Inha University Hospital, Neurology, Incheon, South Korea; ^2^Samsung Medical Center, Sungkyunkwan University School of Medicine, Neurology, Seoul, South Korea

##### **Correspondence:** M. J. Lee

Background and objective: We aimed to provide the first real-world data of anti-calcitonin gene-related peptide (CGRP) receptor monoclonal antibody in Asians.

Methods: We prospectively recruited patients with migraine who received galcanezumab treatment in a single university hospital from June 2020 and Dec 2020. Treatment response was assessed after 3 consecutive monthly injections. A 50% responder rate was defined as ≥50% o reduction in moderate-to-severe headache days.

Results: A total of 54 patients were eligible for the analysis. Patients were mostly female (81.5%) with a mean age was 41.8±12.0 (range 17–71), had chronic migraine in 42 and medication overuse in 27, and previously failed ≥3 classes of preventive medication in 45 (83.3%). After 3 months of treatment, mean changes of monthly headache days, moderate to severe headache days, crystal clear days, and days of acute medication use were -7.4 ± 8.61, -5.0 ± 11.18, +7.4 ± 8.61, and -4.0 ± 9.40, respectively. The 50% responder rate was 77%, 56%, and 44% in patients who previously failed ≤3, 4, and 5 preventive medication classes. Total 68% patients reported any improvement and satisfaction from the treatment.

Conclusion: In our cohort, efficacy and safety of galcanezumab were comparable to those reported from clinical trials and even better in patients who failed multiple preventive drug classes. Our study provides the first real-world evidence of benefits of galcanezumab treatment in Asian patients with migraine.

## P0110 Safety Findings from CENTURION, a Phase 3 Consistency Study of Lasmiditan for the Acute Treatment of Migraine

### C. Tassorelli^1^, S. Bragg^2^, J. Krege^2^, E. G Doty^2^, P. A Ardayfio^2^, D. Ruff^2^, S. Dowsett^2^, T. J. Schwedt^3^

#### ^1^Headache Science Center and Headache Unit, Neurological Institute C Mondino Foundation, Dept of Brain and Behavioral Sciences, University of Pavia, Pavia, Italy; ^2^Eli Lilly and Company, Indiana, IN, United States; ^3^Mayo Clinic, Phoenix, Phoenix, AZ, United States

##### **Correspondence:** C. Tassorelli

Objective: Present safety findings from the placebo-controlled, double-blind Phase 3 study, of lasmiditan treatment across 4 migraine attacks (CENTURION).

Methods: Patients were randomised 1:1:1 to lasmiditan 200mg (LTN200), LTN100, or a control group (received placebo for 3 attacks and LTN50 for either third or fourth attack (1:1). Safety analyses were conducted for patients who took ≥1 dose of study drug.

Results: In CENTURION, 1471 patients treated 4494 attacks. Incidences of treatment-emergent serious adverse events (SAEs) were placebo, n=2 (0.4%); LTN100, n=1 (0.2%); LTN200, n=2 (0.4%); no specific SAE was reported in more than 1 patient. There were no deaths/major cardiovascular events. Most common treatment-emergent adverse events (TEAEs) with lasmiditan were dizziness/paresthesia/fatigue/nausea/ vertigo/somnolence; the vast majority were mild/ moderate in severity. Incidences of TEAEs were highest during the first attack and decreased during subsequent attacks. Median durations of common TEAEs with lasmiditan ranged from 1.1–5.5 hours and was higher in the first versus fourth attack except for fatigue and somnolence. Findings are tabulated for dizziness (most common TEAE).

Conclusion: In this blinded, controlled, multiple-attack study, lasmiditan was associated with generally mild or moderate CNS-related TEAEs of short duration. TEAEs tended to decrease in frequency across the 4 attacks. There were no new safety findings compared with previous single attack studies.

**Table 1 (abstract P0110).**
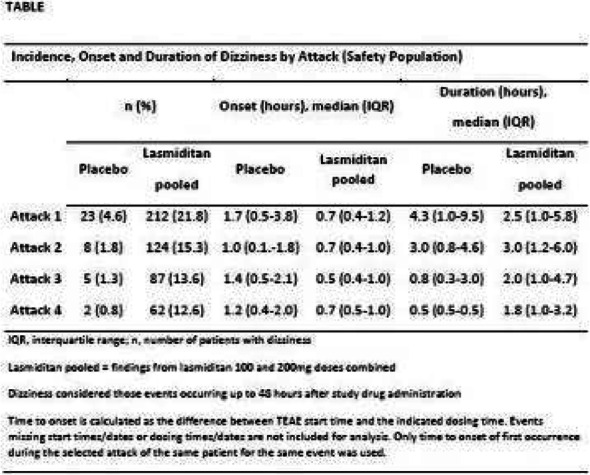
See text for description

## P0111 Haematohidrosis with Headache-A Rare Phenomenon of Sweating Blood: A Case Report

### A. Chowdhury

#### Bangabandhu Sheikh Mujib Medical University, Dhaka, Bangladesh

Haematohidrosis is an extremely rare clinical condition in which the patient experiences sweat mixed with blood. Till date only a few cases of haematohidrosis have been reported in national and international medical journals. Pathogenesis of the condition is not yet established but rupture of the blood vessels of sweat glands due to activation of sympathetic nervous system from stress, anxiety or any other reason have been proposed as the cause of bleeding.

It was aimed to present the case who presented with episodes of sweat mixed with blood from different sites of her body. A case of haematohidrosis who experiences bloody sweat which comes with episodes of headache, was studied during in Bangabandhu Sheikh Mujib Medical University, Dhaka.

Interestingly, it was to be found that a child of the patient, who is a 4 year old boy and a nephew of her, are suffering from the same condition. No family history was found in any of the previous cases. All other history and the investigations were insignificant. It was to be diagnosed the headache as migraine. The patient was treated with propranolol and paracetamol. Her bloody sweat decreased significantly in both severity and frequency with the treatment.

**Fig. 1 (abstract P0111).**
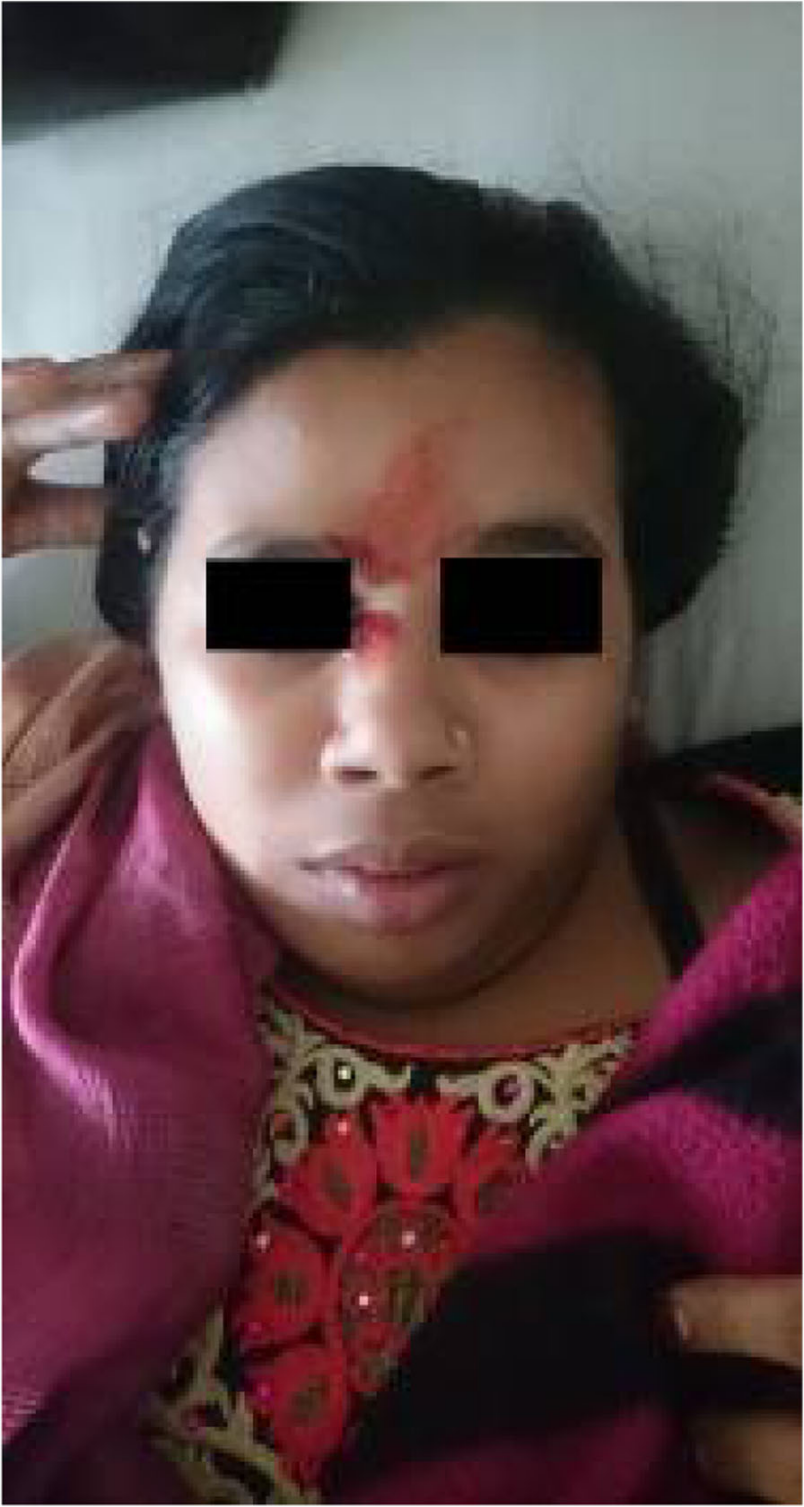
See text for description

**Fig. 2 (abstract P0111).**
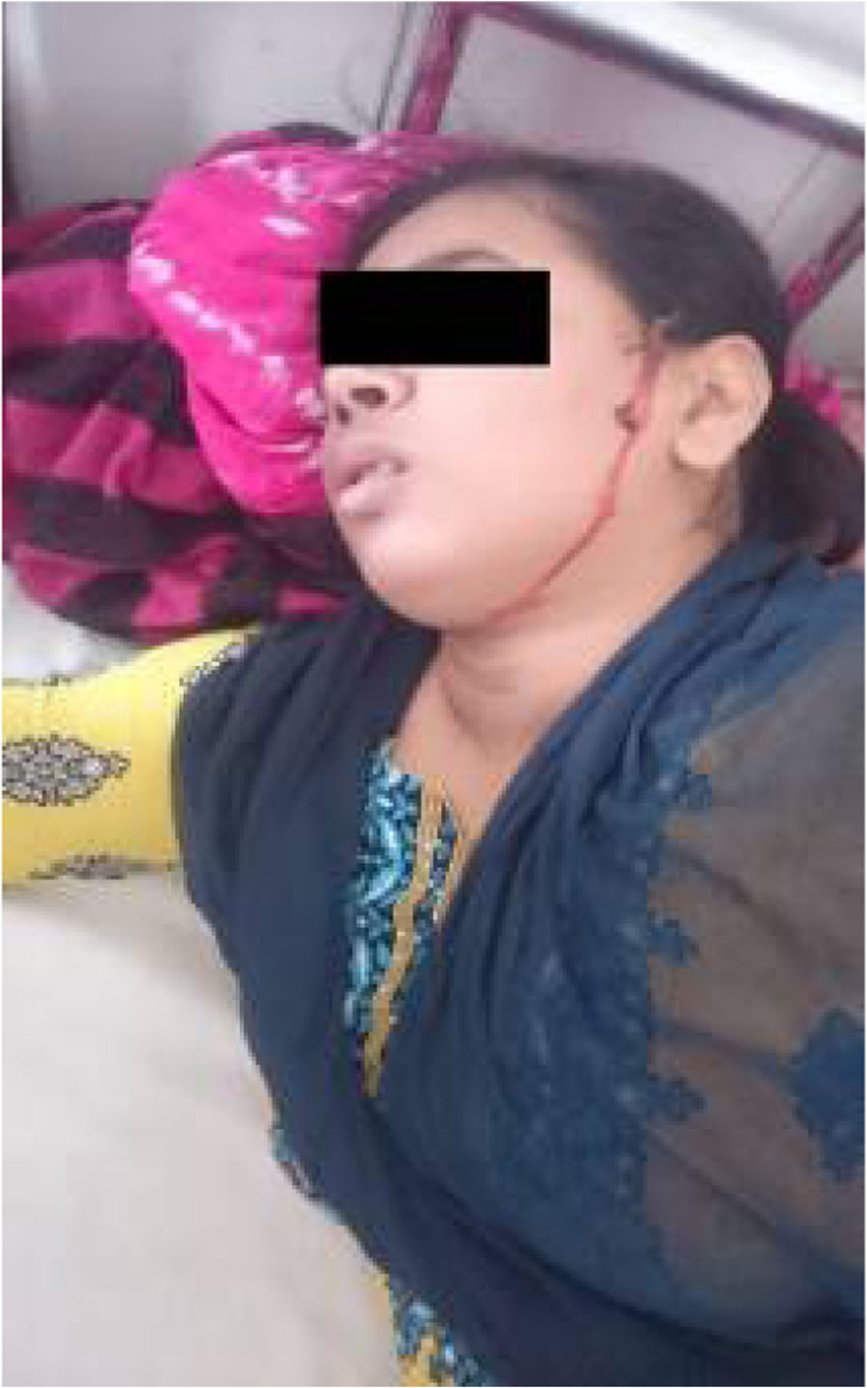
See text for description

## P0112 Preventive oral migraine treatment utilization patterns (POLARIS STUDY): A retrospective claims data analysis

### J. Ailani^1^, A. R. Shewale^2^, D. Oliveri^3^, L. Wilson^3^, K. Burslem^2^, R. B. Lipton^4^

#### ^1^MedStar Georgetown University Hospital, Washington, DC, United States; ^2^AbbVie, North Chicago, IL, United States; ^3^Genesis Research, LLC, Hoboken, NJ, United States; ^4^Albert Einstein College of Medicine, Bronx, NY, United States

##### **Correspondence:** J. Ailani

Objective: To examine the real-world treatment patterns of oral migraine preventive medication (OMPM) by pharmacologic class in episodic migraine (EM) patients and in all migraine patients.

Methods: Adults with ≥1 OMPM claim in 2017(earliest claim date = index date) were identified from the IBM® MarketScan® Commercial database. Patients were required to have ≥1 migraine diagnosis and no claims for an OMPM for the one-year baseline. Treatment patterns were evaluated at 6-,12-, and 24-months post-index date and stratified by the class of the index OMPM. Discontinuation was defined as a gap of ≥30 days without OMPM assuming patients take medication as prescribed.

Results: Of 9,868 new OMPM users, 85.6% had EM. The discontinuation rate in all migraine patients for the index OMPM was 71.1% at 6-months, 81.3% at 12-months (Figure:1a) and 88.7% at 24-months post-index date. Of the 28.1% (n=2773) who initiated a second OMPM class 67.3% discontinued it and of the 6.4% (n=631) who initiated a third OMPM class and 57.2% discontinued it, within 12 months of index OMPM initiation (Figure:1b). Similar patterns were observed at 6- and 24-months post-index as well as among the patients with EM.

Conclusions: Discontinuation rate among migraine patients initiating OMPMs are high and increase with follow-up. Switching or adding another OMPM class is not very common and the discontinuation rates with subsequent therapies are also high.

**Fig. 1 (abstract P0112).**
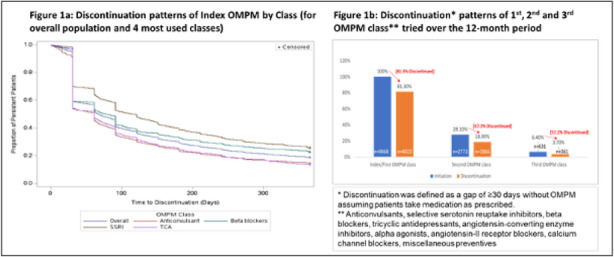
See text for description

## P0113 Exercised Brain in Pain: Quantification of Exercise in Migraine Patients Seen at a Large Tertiary Headache Center

### M. Dyess^1^, A. Cuneo^1^, G. O'Fallon^2^, H. Haley^3^, A. Narula^4^, N. Murinova^1^, D. Krashin^5^, M. Chan-Goh^1^, M. Budica^1^, S. Belaskova^6^

#### ^1^University of Washington Medical Center, Neurology, Seattle, WA, United States; ^2^Case Western University, Research Volunteer, Seattle, WA, United States; ^3^Boston University, Research Volunteer, Boston, MA, United States; ^4^University of Washington, Research Volunteer, Seattle, WA, United Kingdom; ^5^Puget Sound VA, Seattle, WA, United States; ^6^St. Anne's University Hospital, International Clinical Research Centre, Brno, Czech Republic

##### **Correspondence:** M. Dyess

Objective: Quantify the amount of exercise in patients diagnosed with migraine in a tertiary headache clinic at the University of Washington. Analyze migraine characteristics and comorbidities as they relate to exercise.

Design/Methods: All new patients referred to our headache clinic complete a detailed patient intake questionnaire. This questionnaire asks about amount of exercise per week, headache characteristics, sleep, depression, anxiety, and stress. Data are analyzed by headache providers who diagnose patients using the ICHD-3 criteria.

Results: In our analysis, n=4879 unique patients were diagnosed with migraine. 74.7% (n=3644) with chronic, and 25.3% (n=1235) with episodic migraine. Exercise related questions were completed by 95% (n=4647) of patients. 27% (n=1270) of those who exercised reported achieving 150+ minutes of moderate to vigorous exercise weekly, the minimum level recommended by the World Health Organization (WHO). Our analysis suggests that exercise level below the recommended level by WHO is correlated with an increased rate of depression, anxiety, and sleep problems.

Conclusions: We identified that most patients with a migraine diagnosis do not get the minimum level of exercise recommended by the WHO. For patients achieving 150 minutes or more of moderate exercise per week, rates of depression, anxiety, and sleep problems are lower. We recommend raising awareness that exercise can have a significant impact on the headache itself and comorbidities.

## P0114 Clinical characteristics of headache after vaccination against COVID-19 (Coronavirus SARS-CoV-2) with the COVID-19 mRNA-1273 Moderna vaccine: a prospective multicentre observational cohort study

### C. Göbel^1,2^, A. Heinze^2^, S. Karstedt^1,2^, M. Morscheck^2^, L. Tashiro^2^, A. Cirkel^1,2^, Q. Hamid^3^, R. Halwani^3^, M. H. Temsah^4^, M. Ziemann^5^, S. Görg^5^, T. Münte^1^, H. Göbel^2^

#### ^1^University Hospital Schleswig-Holstein, Department of Neurology, Lübeck, Germany; ^2^Kiel Headache and Pain Centre, Kiel, Germany; ^3^University of Sharjah, College of Medicine, Sharjah, United Arab Emirates; ^4^King Saud University, College of Medicine, Riyadh, Saudi Arabia; ^5^University Hospital Schleswig-Holstein, Institute of Transfusion Medicine, Lübeck, Germany

##### **Correspondence:** C. Göbel

Background: The aim of the study is to examine in a real live situation in detail the phenotype of headaches occurring after vaccination against Covid-19 with the COVID-19 mRNA-1273 Moderna vaccine.

Methods: The study is a continuous prospective multicenter observational cohort study taking place during the Covid-19 vaccination campaign. With a publicly available online questionnaire, specific aspects of the headache phenotype and related variables are collected globally. Attention was drawn to the study via websites and social media.

Findings: In this interim analysis a total of 583 participants reported headaches after vaccination with the COVID-19 Vaccine mRNA-1273 Moderna. The mean age of the participants was 42.9 ± 12.6 years. 93.3% stated that they had not experienced any headaches with any other vaccination. Headaches occur an average of 16.8 ± 28.1 hours after vaccination and last an average duration of 17.0 ± 23.2 hours. In 72.4% of the participants headache occurs as a single episode. 77.3% of participants indicate a bilateral location. This is most often found on the forehead (38.9%), temples (32.1%) and occipital area (26.9%). 48.0% indicate a pressing and 39.3% a dull pain character. The pain intensity is most often moderate (41.9%), severe (37.1%) or very severe (10.4%).

Interpretation: Headaches after Covid-19 vaccination with the COVID-19 mRNA-1273 Moderna vaccine show a characteristic headache phenotype with numerous inflammatory accompanying symptoms.

## P0115 A Survey of the Most Common Drugs Causing Headaches in FDA Adverse Event Reporting System

### B. Musialowicz, B. Kamitaki, P. Zhang

#### ^1^Rutgers Robert Wood Johnson Medical School, New Brunswick, NJ, United States

##### **Correspondence:** B. Musialowicz; P. Zhang

Background: This project seeks to identify classes of medications most likely to cause drug-induced headaches in the FDA Event Reporting System (FAERS).

Methods: We extracted case ID, adverse events, and attributed medications for entries in the FAERS database from July 2018 to March 2020. Each entry occupied a line in our data. We removed duplicate words in each line. We separated entries into two files based on whether each contained the word "headache(s)". We counted the occurrences for unique words in each file. Using this result, we calculated the reporting odds ratios (ROR) and 95% confidence interval for unique words in the "headache(s)" database. We then excluded all English words from the list and ranked the resultant list of drug names by ROR.

Results: We extracted 2,673,081 entries of which 86,086 contain the word "headache(s)". Medications with highest 50 ROR and ROR lower bounds include nitrates, contraceptives, antihistamines, sedatives, antifungals/antibiotics, anti-neoplastics, pulmonary hypertension directed vasodilators, and immunosuppressants. A number of headache medications were also included: NSAIDs, opioids, antidepressants, and beta-blockers.

Conclusion: Our study offers a potential list of the medication classes most likely to cause iatrogenic induced headaches and may offer insights into headache pathophysiology. The inclusion of headache medications in our results may be due to indication bias, reflecting the inherit limitations of our method.

## P0117 Migraine Management with Telemedicine Visits Only: Can Patients Achieve Clinically Meaningful Improvement?

### B. Torphy, M. Smith, A. Calvillo, A. Kovlari

#### Chicago Headache Center & Research Institute, Chicago, IL, United States

##### **Correspondence:** B. Torphy

Objective: There is a paucity of research about telemedicine in the treatment of migraine, and that which does exist involves telemedicine as a follow-up strategy after an initial in-office visit. The purpose of this study was to determine if clinically meaningful improvement in migraine can be achieved with the use of synchronous telemedicine visits only.

Methods: In a retrospective chart review we assessed Headache Impact Test – 6 (HIT-6) scores for new patients at the initial visit, which was conducted via synchronous telemedicine. HIT-6 scores were also assessed at follow-up visits. Patient visits from 3 March 2020 – 18 March 2021 were included. Patients who had an in-office initial or follow-up visit or did not complete HIT-6 test were excluded from the study.

Results: At follow up 80% of patients who met screening criteria (n=73) had an improvement in HIT-6 score, and 60% of those patients with improvement had a reduction of > 6 points, which is a threshold previously identified as clinically meaningful in patients with chronic migraine. Mean improvement in HIT-6 scores at > 1 month follow-up was 5 points, and mean improvement in HIT-6 scores at follow-up visits > 3 months was > 6 points. Data was analyzed with SPSS version 27.

Conclusions: Clinically meaningful improvement in migraine can be achieved with the exclusive use of telemedicine visits for migraine care. Further research is needed to compare this improvement with that which is seen using in-office visits.

**Fig. 1 (abstract P0117).**
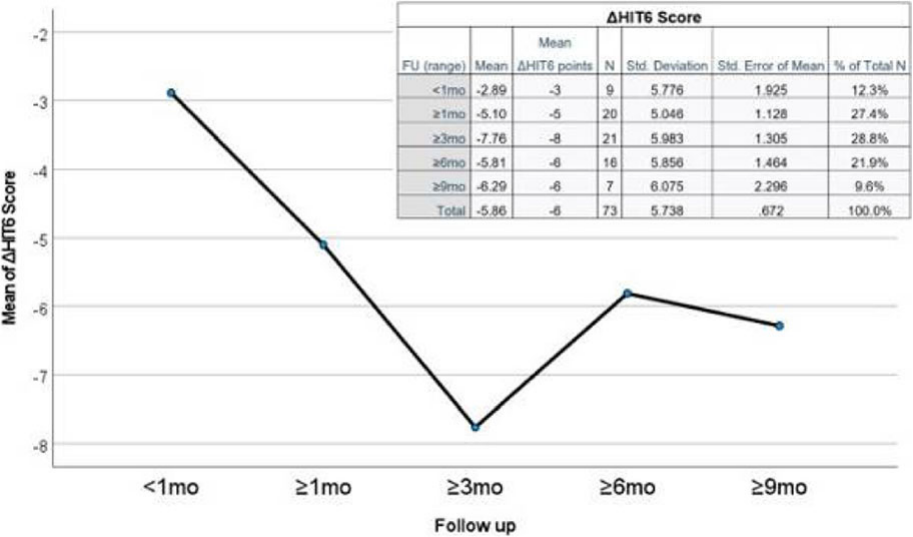
See text for description

**Fig. 2 (abstract P0117).**
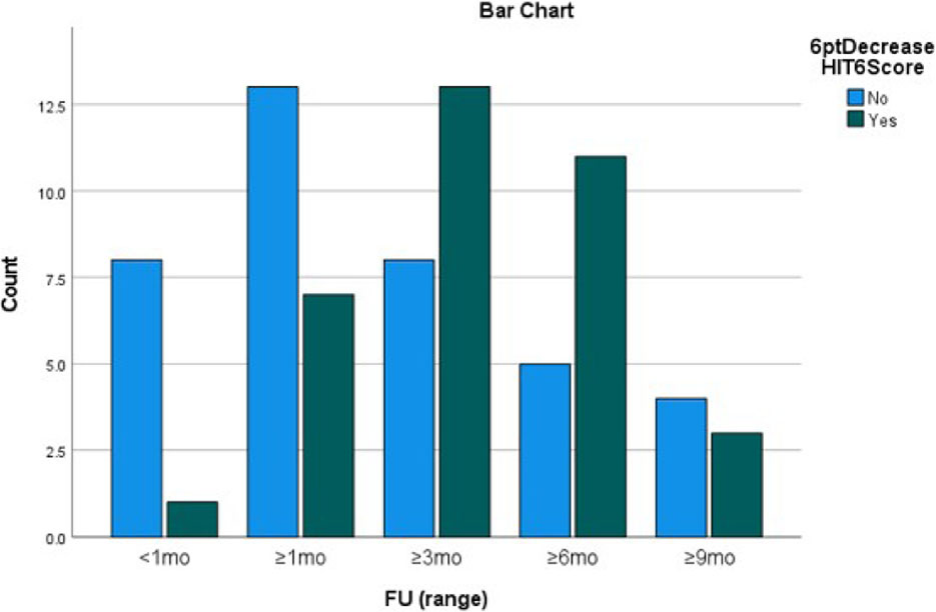
See text for description

## P0118 How much weight loss is required to reduce intracranial pressure in idiopathic intracranial hypertension?

### S. P. Mollan^1^, J. Mitchell^2^, A. Yiangou^2^, R. Ottridge^3^, Z. Alimajstorovic^2^, D. Cartwright^4^, S. Hickman^5^, R. Singhal^6^, A. Tahrani^4^, E. Frew^7^, K. Brock^8^, A. J. Sinclair^2^

#### ^1^University Hospitals Birmingham, Neuro-ophthalmology, Birmingham, United Kingdom; ^2^University of Birmingham, Metabolic neurology, Birmingham, United Kingdom; ^3^University of Birmingham, Birmingham Clinical Trials Unit,, Birmingham, United Kingdom; ^4^University of Birmingham, Institute of Metabolism and Systems Research, Birmingham, United Kingdom; ^5^Royal Hallamshire Hospital, Department of neurology, Sheffield, United Kingdom; ^6^University Hospitals Birmingham, Upper GI Unit and Minimally Invasive Unit, Birmingham, United Kingdom; ^7^University of Birmingham, Institute of Applied Health Research, Birmingham, United Kingdom; ^8^University of Birmingham, Institute of Cancer and Genomic Sciences, Birmingham, United Kingdom

##### **Correspondence:** S. P. Mollan

Background: The amount of weight loss required in idiopathic intracranial hypertension to reduce intracranial pressure (ICP) has not been established.

Methods: Using the IIH:weight trial data from 66 active patients randomised to bariatric surgery or community weight management intervention (CWI) (1:1). The expected ICP values are predicted by a linear hierarchical regression model fit to the trial outcomes, adjusted for time, treatment arm and weight.

Results: Modelling the trial outcomes demonstrated that greater reduction in ICP was predicted with greater weight loss, with 24% weight loss resulting in normalisation of ICP in this population. The effect on ICP further improves between 12 to 24 months as the participants continue to lose weight. For expected ICP values to cross the threshold for normal, at 25cmCSF within 2 years, it is generally required that the patient would be allocated to the bariatric surgery arm and achieve a weight of 110kg. Those with a higher starting weight needed to lose more weight to meaningfully reduce ICP. This model also demonstrated that in the CWI arm if no or little weight loss was achieved in those with a high baseline weight an increase in ICP would be expected.

Conclusions: There should be care when exposing women with IIH and BMI≥35kg/m2 to repeated cycles of lifestyle interventions that fail to achieve adequate weight loss, as this approach is unlikely to achieve sustained remission of disease.

## P0119 Bariatric surgery versus community weight management intervention for the treatment of idiopathic intracranial hypertension (IIH:WT): A randomized controlled trial

### S. P. Mollan^1^, J. Mitchell^2^, R. Ottridge^3^, M. Aguiar^4^, A. Yiangou^2^, Z. Alimajstorovic^2^, D. Cartwright^5^, O. Grech^2^, G. G. Lavery^5^, C. S. J. Westgate^2^, V. Vijay^2^, W. Scotton^2^, B. R. Wakerley^5^, T. Matthews^1^, A. Ansons^6^, S. Hickman^7^, J. Benzimra^8^, C. Rick^9^, R. Singhal^10^, A. Tahrani^5^, K. Brock^11^, E. Frew^4^, A. J. Sinclair^2^

#### ^1^University Hospitals Birmingham, Neuro-ophthalmology, Birmingham, United Kingdom; ^2^University of Birmingham, Metabolic neurology, Birmingham, United Kingdom; ^3^University of Birmingham, Birmingham Clinical Trials Unit,, Birmingham, United Kingdom; ^4^University of Birmingham, Institute of Applied Health Research, Birmingham, United Kingdom; ^5^University of Birmingham, Institute of Metabolism and Systems Research, Birmingham, United Kingdom; ^6^Manchester University NHS Foundation Trust, Manchester, United Kingdom; ^7^Royal Hallamshire Hospital, Department of neurology, Sheffield, United Kingdom; ^8^Royal Devon and Exeter NHS Foundation Trust, Department of Ophthalmology, Exeter, United Kingdom; ^9^University of Nottingham, Nottingham Clinical Trials Unit, Nottingham, United Kingdom; ^10^University Hospitals Birmingham, Upper GI Unit and Minimally Invasive Unit, Birmingham, United Kingdom; ^11^University of Birmingham, Institute of Cancer and Genomic Sciences, Birmingham, United Kingdom

##### **Correspondence:** S. P. Mollan

Objective: The IIH weight trial (IIH:WT) aimed to compare the efficacy of bariatric surgery with a community weight management intervention (CWI) in active IIH.

Methods: This was a five-year randomized control trial which enrolled participants between March 1, 2014 and May 25, 2017 at five hospitals in the United Kingdom. Participants with active IIH and body mass index (BMI) ≥35kg/m^2^ were screened. The primary outcome was change in intracranial pressure (ICP) measured by lumbar puncture (LP) opening pressure (OP) at 12 months.

Results: Sixty-six women were randomised (mean age, 32 years). ICP was significantly lower in the bariatric surgery arm at 12 months (adjusted mean difference -6.00cm cerebrospinal fluid [CSF] 95% confidence interval [CI] -9.5 to -2.4]; p= .001) and at 24 months (adjusted mean difference -8.2cmCSF [95% CI, -12.2 to -4.2]; p< .001) compared with the CWI arm. Weight was significantly lower in the bariatric surgery arm at 12 months (adjusted mean difference -21.4Kg 95% CI, -32.1 to -10.7]; p< .001) and at 24 months (adjusted mean difference -26.6kg [95% CI, -37.5 to -15.7]; p< .001) compared with the CWI arm. Quality of life (SF36, physical component score) improved significantly at 12 and 24 months (adjusted mean difference p=.043; p=.006, respectively).

Conclusions: Bariatric surgery was superior to a CWI in lowering ICP in IIH women with a BMI ≥35kg/m^2^. Continued improvement at two years demonstrated the impact on sustained disease remission.

## P0120 Intracranial pressure determines headache morbidity in idiopathic intracranial hypertension

### S. P. Mollan^1^, B. R. Wakerley^2^, Z. Alimajstorovic^3^, J. Mitchell^3^, R. Ottridge^4^, A. Yiangou^3^, M. Thaller^3^, O. Grech^3^, G. G. Lavery^2^, K. Brock^5^, A. J. Sinclair^3^

#### ^1^University Hospitals Birmingham, Neuro-ophthalmology, Birmingham, United Kingdom; ^2^University of Birmingham, Institute of Metabolism and Systems Research, Birmingham, United Kingdom; ^3^University of Birmingham, Metabolic neurology, Birmingham, United Kingdom; ^4^University of Birmingham, Birmingham Clinical Trials Unit, Birmingham, United Kingdom; ^5^University of Birmingham, Institute of Cancer and Genomic Sciences, Birmingham, United Kingdom

##### **Correspondence:** S. P. Mollan

Objective: The aim was to characterise headache and investigate the association with intracranial pressure (ICP) in Idiopathic Intracranial Hypertension (IIH).

Methods: IIH:WT was a randomised controlled trial investigating weight management methods in IIH. Active IIH participants (evidenced by papilloedema) and a body mass index (BMI) ≥35kg/m^2^ were recruited. At baseline, 12 months and 24 months headache characteristics and quality of life outcome measures were collected and lumbar puncture measures were performed.

Results: Sixty-six women were included (mean age 32.0 years (SD ± 7.8)), and mean body mass index of 43.9 ± 7.0 kg/m^2^. The headache phenotype was migraine-like in 86%. Headache severity correlated with ICP) at baseline (r=0.285; p=0.024); change in headache severity and monthly headache days correlated with change in ICP at 12 months (r=0.454, p=0.001 and r=0.419, p=0.002 respectively). Cutaneous allodynia was significantly correlated with ICP at 12 months. (r=0.479, p<0.001). Boot strap analysis noted a positive association between ICP at 12 and 24 months and enabled prediction of change in headache severity and monthly headache days. ICP was associated with significant improvements in quality of life (SF-36).

Conclusions: We demonstrate a positive relationship between ICP and headache and cutaneous allodynia, which has not been previously reported. Those with the greatest reduction in ICP had the greatest reduction in headache frequency and severity.

## P0121 Safety of Select Headache Medications in Patients with Cerebral and Spinal Cavernous Malformations

### C. C. Chiang^1^, R. Brown^1^, G. Lanzino^2^, K. Flemming^1^

#### ^1^Mayo Clinic, Neurology, Rochester, MN, United States; ^2^Mayo Clinic, Neurosurgery, Rochester, MN, United States

##### **Correspondence:** C. C. Chiang

Background: Patients with cavernous malformations (CM) and a primary headache disorder are often limited in medication options due to concern for bleeding risk.

Methods: From a prospective cohort of patients at Mayo Clinic with CM between 2015 and February 2021, demographics, clinical presentation, and radiographic lesion location data were collected. Medical record reviews and written surveys were used for patient follow-ups. We studied medications used from the time of diagnosis of the CM to a censor date of first prospective symptomatic hemorrhage, complete surgical excision of sporadic form CM, or death. Using logistic regression, the influence of non-aspirin NSAID (NA-NSAID), triptan, or OnabotulinumtoxinA on prospective hemorrhage risk was assessed.

Results: 329 patients with spinal or cerebral CM (58% female; 20.1% familial; 42.2% presentation to medical attention from hemorrhage; 27.4% brainstem) were included. During a follow-up of 1799.9 patient-years, 92 prospective hemorrhages occurred. The use of NA-NSAIDs, triptans, and OnabotulinumtoxinA after the diagnosis of CM was not associated with an increased risk of prospective hemorrhage. NSAID and triptan users were more commonly women and less commonly had a history of hemorrhage at diagnosis.

Conclusions: The use of triptans and NA-NSAIDs in patients with CM studied, does not precipitate hemorrhage. Similarly, we did not find that OnabotulinumtoxinA (≤200 units per session) precipitated CM hemorrhage.

**Table 1 (abstract P0121).**
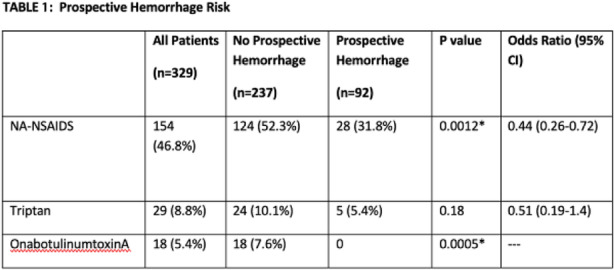
See text for description

## P0122 Neuroimaging in the Diagnosis of Idiopathic Intracranial Hypertension – Is It Useful?

### J. Juhl Korsbaek^1^, D. Beier^2^, L. Hoegedal^3^, R. H. Jensen^1^

#### ^1^Danish Headache Center, Department of Neurology, Rigshospitalet-Glostrup, Glostrup, Denmark; ^2^Odense University Hospital, Department of Neurology, Odense, Denmark; ^3^Odense University Hospital, Department of Radiology, Odense, Denmark

##### **Correspondence:** J. Juhl Korsbaek

Objectives

Although debated, diagnostic criteria for Idiopathic Intracranial Hypertension (IIH) include neuroimaging signs of elevated intracranial pressure (ICP). Presence of ≥ 3 signs have shown high specificity for IIH in case-control studies. We present the first large, prospective field study of the diagnostic criteria.

Methods: We prospectively included patients with suspected IIH and did a standardized diagnostic work-up (interview, neuro-ophthalmological and neurological exam, lumbar puncture, neuroimaging). Exclusion criteria were pregnancy, previous IIH, secondary ICP elevation and missing data. Neuroimaging (MRI and CT/MRI venography) was evaluated by a blinded neuro-radiologist for pituitary morphology, distension of the optic nerves, flattening of the globe and sinus venous stenoses.

Results: We included 157 patients, and found IIH in 56.1 %, probable IIH in 1.9 %, suggested IIH without papilledema in 0.6 % and non-IIH in 41.4 %. Optic nerve distension, flattening of the globe, sinus venous stenoses and partial empty sellae were more common in IIH than in non-IIH (p < 0.01, < 0.0001, < 0.0001 and 0.03). The specificity of ≥ 3 signs was 93 % and the sensitivity was 63.4 %.

Conclusion: We present sufficient prospective evidence of the high specificity of neuroimaging in a large, well-defined population. Based on this we suggest an update to the diagnostic criteria increasing the use of neuroimaging.

## P0123 Thunderclap Headache: A primary symptom of a steroid responsive encephalopathy with autoimmune thyroiditis

### N. Zala^1^, L. Wirth^1^, T. Rizos^1^, B. Jordan^1^, H. Meredig^2^

#### ^1^University Hospital Heidelberg, Neurology, Frankfurt am Main, Germany; ^2^University Hospital Heidelberg, Neuroradiology, Heidelberg, Germany

##### **Correspondence:** N. Zala

Objective: Thunderclap headache is frequently associated with intracranial vascular disorders and is a frequent cause for emergency department admission. A correlation of thunderclap headache with autoimmune disorders, such as steroid responsive encephalopathy with autoimmune thyroiditis (SREAT), is highly unusual.

Method: A 79-year-old female presented with a sudden onset of high-intensity bifrontal headache without other neurological manifestations. CSF analysis revealed moderate lymphocytic pleocytosis without evidence of infectious, neoplastic or metabolic causes. Brain MRI showed diffuse white matter signal abnormality and hyperperfusion of leptomeningeal arteries.

Result: The medical history revealed an episode of aseptic meningoencephalitis which responded to steroids. On further analysis increased levels of serum anti-TPO antibodies were identified and against the background of a previous steroid responsive aseptic meningoencephalitis, diagnosis of SREAT was considered highly probable. Steroid therapy was initiated, which resulted in a full recovery.

Conclusion: In particular because SREAT responds well to steroids, our case underlines the importance of considering SREAT during assessment of a sudden high-intensity headache associated with mild to moderate neuropsychiatric symptoms.

**Fig. 1 (abstract P0123).**
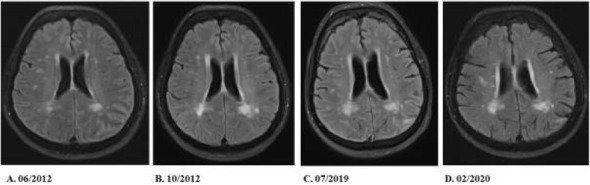
progressive diffuse signal abnormalities of white matter over 7-year period (A-C) with gradual resolving after steroids (D)

**Fig. 2 (abstract P0123).**
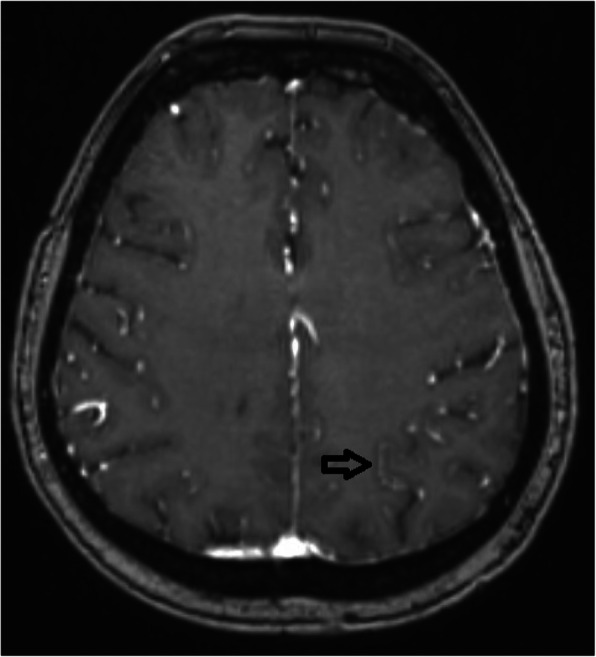
Hyperperfusion of leptomeningeal arteries

## P0124 Fertility in Idiopathic Intracranial Hypertension

### M. Thaller^1,2^, J. Mytton^3^, A. Yiangou^1,2^, J. Mitchell^1,2^, O. Grech^1^, Z. Alimajstorovic^1^, B. R. Wakerley^2^, S. P. Mollan^4^, A. J. Sinclair^1,2^

#### ^1^University of Birmingham, Birmingham, United Kingdom; ^2^Queen Elizabeth Hospital, Neurology, Birmingham, United Kingdom; ^3^Queen Elizabeth Hospital, Health Informatics, Birmingham, United Kingdom; ^4^Queen Elizabeth Hospital, Birmingham Neuro-Ophthalmology, Birmingham, United Kingdom

##### **Correspondence:** M. Thaller

Background and Objective: Idiopathic intracranial hypertension (IIH) is associated with hyperandrogenism and affects women of reproductive age with obesity, however the impact on fertility is not known. In this UK observational study, we quantified the impact of IIH on fertility and pregnancy outcome.

Methods: Data was extracted from the Hospital Episodes Statistics (HES) database for all females, aged between 18 and 45, admitted to hospitals in England between 1st April 2002 and 31st March 2019 with a diagnosis of IIH. This was compared to 2 groups of Polycystic ovary syndrome (PCOS) and general population (neither IIH nor PCOS) patients.

Results**:** Data was collected from 17,587 IIH, 199,633 PCOS, and 10,947,012 general population patients. The live birth rate was significantly lower amongst women with IIH (54.1%) compared to PCOS (67.9%), p<0.0001 and compared to general population (57.7%), p<0.0001. Following diagnosis of IIH, pregnancy rate decreased by 42% from 0.65 to 0.38 live births/female. Post IIH diagnosis elective caesarean sections were more than twice that of the general population (OR 2.4, 95%CI 2.3-2.5) compared to pre-diagnosis (OR 1.1, 1.0-1.2).

Conclusion: Women with IIH had lower pregnancy rate than the general population. Following IIH diagnosis, pregnancy rate almost halved, and elective caesarean sections more than doubled.

## P0125 Changes in Intracranial Pressure Waveform in Patients with Idiopathic Intracranial Hypertension

### R. Buckman^1,2,3^, J. Mitchell^1,2,3^, A. Yiangou^1,2,3^, M. Thaller^1,2,3^, O. Grech^1^, Z. Alimajstorovic^1^, B. R. Wakerley^3^, S. P. Mollan^4^, A. J. Sinclair^1,2,3^

#### ^1^University of Birmingham, Institute of Metabolism and Systems Research, Birmingham, United Kingdom; ^2^Birmingham Health Partners, Centre for Endocrinology, Diabetes and Metabolism, Birmingham, United Kingdom; ^3^University Hospitals Birmingham NHS Foundation Trust, Department of Neurology, Birmingham, United Kingdom; ^4^University Hospitals Birmingham NHS Foundation Trust, Birmingham Neuro-Opthalmology, Opthalmology Department, Birmingham, United Kingdom

##### **Correspondence:** R. Buckman; J. Mitchell

Introduction: Telemetric intracranial pressure (ICP) monitoring is increasingly utilised to manage complex cerebrospinal fluid disorders. Changes in ICP waveforms are poorly understood. We aimed to evaluate the changes in ICP waveforms due to alterations in posture.

Methods: Telemetric ICP monitors (RauMedic p-Tel, Hembrechts, Germany) were inserted in patients with active IIH (papilloedema, ICP > 25cmCSF) at least one week prior to baseline assessment. ICP was recorded over 60 minutes in supine and standing positions. For each ICP recording average peak pressure, trough pressure and waveform amplitude were determined using LabChart 7 peak analysis software.

Results**:** ICP waveforms were recorded in 16 females (one withdrew and one had recording error). At enrolment ICP was 24.8 (SD 4.1) mmHg (equivalent to 33.7cmCSF), mean age 28±9 yrs and body mass index 38.1±6.2 kg/m2. In supine position mean ICP was 24.0±4.8 mmHg and fell to 12.9±3.4 mmHg in standing position (change mean -11.1±4.7 mmHg, p<0.0001). Changing from supine to standing lead to a significant fall in peak pressure (-11.1± 6.6 mmHg, p<0.0001), and trough pressure (-11.9±4.3 mmHg, p<0.0001), and significant rise in amplitude (+2.0±0.9 mmHg, p<0.0001).

Conclusions: Moving from supine to standing decreased ICP by 50% and altered the waveform parameters. Extending ICP analysis to interpreting waveforms is likely to lead to greater understanding of cerebral compliance and perturbations by disease.

## P0127 Obstructive sleep apnoea in idiopathic intracranial hypertension: findings from the Idiopathic Intracranial Hypertension Weight Trial (IIH:WT)

### A. Yiangou^1,2,3^, J. Mitchell^1,2,3^, M. Nicholls^1,4^, Y. C. Chong^5^, V. Vijay^1,2,3^, B. R. Wakerley^3^, G. G. Lavery^1,2,3^, A. Tahrani^1,4,6^, S. P. Mollan^5^, A. J. Sinclair^1,2,3^

#### ^1^University of Birmingham, Metabolic Neurology, Institute of Metabolism and Systems Research, Birmingham, United Kingdom; ^2^Birmingham Health Partners, Centre for Endocrinology, Diabetes and Metabolism (CEDAM), Birmingham, United Kingdom; ^3^University Hospitals Birmingham NHS Foundation Trust, Department of Neurology, Birmingham, United Kingdom; ^4^Birmingham Health Partners, Centre of Endocrinology, Diabetes and Metabolism (CEDAM), Birmingham, United Kingdom; ^5^University Hospitals Birmingham NHS Foundation Trust, Birmingham Neuro-Ophthalmology Unit, Ophthalmology Department, Birmingham, United Kingdom; ^6^University Hospitals Birmingham NHS Foundation Trust, Department of Endocrinology, Birmingham, United Kingdom

##### **Correspondence:** A. Yiangou

Objective: Obesity is a risk factor for idiopathic intracranial hypertension (IIH) and obstructive sleep apnoea (OSA). The aim was to determine the prevalence of OSA in IIH, and the association between OSA and papilloedema.

Methods: The IIH:WT was a multicentre, randomised controlled trial that evaluated bariatric surgery vs. community weight management intervention (CWI) on intracranial pressure (ICP). In this planned substudy, OSA was measured (two consecutive nights) using home polygraphy measuring the apnoea-hypopnoea index (AHI) at baseline and 12 months.

Results: Analysis included 40 women with active IIH. OSA prevalence was 47% (n=25) (American Academy of Sleep Medicine criteria). Questionnaire screening for OSA had greatest sensitivity with STOP-BANG (84%) compared to Berlin (68%) and the Epworth Sleepiness Scale (69%). Bariatric surgery improved OSA severity compared to CWI (median[95%CI] AHI reduction of -2.8[-11.9, 0.7], p=0.017). The reduction in the AHI over 12 months correlated with reduction in papilloedema (optic nerve head volume) (r=0.543, p=0.045), which remained significant after adjustment for changes in body mass index (BMI) (R2=0.522, p=0.017).

Conclusion: OSA is common in IIH, STOP-BANG was the most sensitive screening tool and bariatric surgery improved OSA in IIH. Importantly, improvement in OSA was associated with reduction in papilloedema independent of changes in BMI. Treating OSA in IIH may improve papilloedema and needs further investigation.

## P0128 Changes in the intensity of pain and quality of life in patients with low-grade gliomas after surgical treatment

### M. Kurnukhina, V. Cherebillo

#### First Pavlov State Medical University of St. Petersburg, Neurosurgery, St. Petersburg, Russian Federation

##### **Correspondence:** M. Kurnukhina

Background: Glial tumors make up the majority of primary tumors of the CNS in adults and include a whole range of tumors with different levels of cellular differentiation and malignancy. Headaches are one of the most common complaints of patients with low-grade gliomas (LGG).

Materials and methods: A clinical study of 80 patients with LGG was conducted. The analysis of the pain and quality of life was carried out before the operation, as well as for 5 years after the surgery. Age of the patients: from 18 to 72 years (median 47,5 years). To assess intensity of pain and the quality of life we selected VAS and special questionnaire EORTC QLQ-C30.

Results and discussion: In the early postoperative period, patients report an increase in the severity of pain(VAS 8), but 3-6 months after surgery-a decrease in pain to VAS 4. In the first year of the study marked a significant improvement in the quality of life in patients with LGG for the functional scales, cognitive functioning, pain syndrome (p<0,05). Statistically significant influence on the period of transformation LGG to HGG, physical, social, emotional functioning, pain intensity, factors such as patient age, the size of the formation, morphological variation of the LGG, the presence of mutations IDH, BRAF, TERT, Vim (p<0,05).

Conclusion: Surgical treatment has a positive effect in the pain syndrome and on the quality of life of LGG patients in the late postoperative syndrome.

## P0129 Minimally invasive surgery for spinal CSF-leaks in spontaneous intracranial hypotension

### J. Beck, U. Hubbe, J. H. Klingler, R. Rölz, L. M. Kraus, H. Urbach, C. Fung

#### University of Freiburg, Germany, Neurosurgery, Freiburg, Germany

##### **Correspondence:** J. Beck

Background and objective: To describe minimal invasive surgical treatment of spinal cerebrospinal fluid (CSF) leaks in patients with spontaneous intracranial hypotension (SIH).

Methods: Between 4-2019 and 12-2020 we included all consecutive patients with SIH undergoing surgery for a spinal CSF-leak. CSF leaks were diagnosed by dynamic myelography. Surgery was performed under general anesthesia in the prone position via a 2.5 cm dorsal midline incision using 20 mm tubular retractors. Primary outcome was the minimal invasive closure of the CSF leak, secondary outcome was the occurrence of complications.

Results: We included 58 patients, median age 46 (IQR 36-55), 38 female (65.5%) with the diagnosis of SIH. We performed 62 surgical procedures. We diagnosed 38 ventral leaks (65.5%), 17 lateral leaks (29.3%) and 2 CSF-venous fistulas (3.4%). In all but one patient (98%) the leak could be approached, identified and closed via the tubular retractor. 1 patient had two surgeries due to wrong level. Immediately after surgery in 76% of patients symptoms of SIH subsided completely or even transformed to high pressure headache. Overall revision rate was 8.6% due to seroma, suture insufficiency and 2 recurrent leaks. There was 1 patient with a mild permanent weakness of the thumb (1.7%).

Conclusion: Minimally invasive surgery using tubular retractors can be safely and effectively performed for closure of ventral and lateral spinal CSF leaks as well as CSF-Venous fistulas.

## P0130 Spinal CSF leaks and superficial siderosis: Closely linked diseases?

### C. Fung^1^, B. Haupt^1^, D. Cipriani^1^, L. Häni^2^, A. Raabe^2^, . M. Kraus^1^, K. Argiti^1^, A. El-Rahal^1^, J. Beck^1^

#### ^1^Medical Center, University of Freiburg, Department of Neurosurgery, Freiburg, Germany; ^2^Inselspital, Department of Neurosurgery, Bern, Switzerland

##### **Correspondence:** C. Fung

Objective: Superficial siderosis (SS) is a rare condition characterized by hemosiderin deposition in the subpial layers of the brain and spinal cord. Spinal CSF leaks in the setting of spontaneous intracranial hypotension might be one pathophysiological condition that causes SS. Here we present the largest series of patients with spinal CSF leaks and SS and display possible associations between SIH and SS.

Methods: We included all consecutive patients with a surgically confirmed spinal CSF leak as well as clinical or radiological SS. Demographic, clinical, and imaging data were extracted from patients" medical records and databases.

Results: We identified 12 patients with both a leak and SS; mean age of 59 and equal distribution between sex. 3 time dependent patterns could be identified: i) patients with SIH symptoms without symptoms related to SS yet clear imaging findings of SS, ii) patients with SIH symptoms and symptoms and imaging findings related to SS iii) and patients without SIH symptoms but with symptoms related to SS. After closure of all spinal CSF leaks all SIH related symptoms resolved and 70% of patients improved with respect to their SS related symptoms, especially if treated early.

Conclusion: Based on this cohort we propose that spinal CSF leaks causes SS. The process most likely takes several years. Radiological signs of SS precede the clinical manifestation. Diagnosis and timely closure of a CSF leak seems to stop progression and improve symptoms.

## P0131 Persistent headache attributed to past cervicocephalic artery dissection: clinical characterization and predictors of headache persistence

### B. Martins, I. Mesquita, P. Abreu, A. Costa

#### Faculdade de Medicina da Universidade do Porto, Porto, Portugal

##### **Correspondence:** B. Martins

Objective: To evaluate clinical characteristics and predictors of headache persistence after cervicocephalic artery dissection (PHPAD).

Methods: Retrospective cohort study of patients with cervicocephalic artery dissection (CCAD) between 2015-2020. Demographics and clinical data were obtained via clinical records, while persistent headache characterization was obtained via telephonic questionnaire.

Results: We identified 90 patients with CCAD; 22 (24%) had persistent headache. Comparing patients with PHPAD and no-PHPAD, there were no differences concerning gender, age, and cardiovascular risk factors. There were statistically significant differences regarding previous history of headache (64% *vs*. 5%); delay from symptoms onset to diagnosis (3.6 days vs. 1.9); and headache/cervical pain in the acute event (82% *vs.* 43%). A logistic regression model depicted previous headache history, posterior circulation dissection and lower initial NIHSS as predictive factors of PHPAD.

Conclusion: Few studies characterized patients with PHPAD and even less addressed its predictors. In our study, about a quarter of patients with a history of dissection had PHPAD. Previous headache history, posterior circulation dissection and less severe disease were identified as predictors of headache persistence.

## P0133 New diagnostic criteria for acute headache attributed to ischemic stroke

### E. R. Lebedeva^1^, A. V. Ushenin^1^, N. M. Gurary^2^, D. V. Gilev^3^, J. Olesen^4^

#### ^1^the Ural State Medical University, International Headache Center "Europe-Asia", Yekaterinburg, Russian Federation; ^2^MU "New Hospital", Neurology, Yekaterinburg, Russian Federation; ^3^the Ural State Medical University, International Headache Center "Europe-Asia", Department of Economics, Yekaterinburg, Russian Federation; ^4^Danish Headache Center, Copenhagen, Denmark

##### **Correspondence:** E. R. Lebedeva

Background: The International Classification of Headache Disorders (ICHD) diagnostic criteria for acute headache attributed to ischemic stroke are based primarily on the opinion of experts. The aim of this study was to field test, for the first time, the diagnostic criteria for these headaches of the ICHD-3.

Methods: The study population consisted of 550 patients (mean age 63,1, 54% males) with first-ever ischemic stroke, and 192 control patients (mean age 58.7, 36% males) admitted to the emergency room without any acute neurological deficits or serious disorders. All data were collected prospectively, using a standardized case-report form during face-to-face interviews by neurologists.

Results: Headache at onset of ischemic stroke was present in 82 (14.9%) of 550 patients with stroke. A new type of headache occurred in 46 (56%) of patients with stroke and in no controls, a previous headache with altered characteristics was found in 30 of the 82 patients with stroke (36%) and two control patients (p<0.009). Six patients had a usual headache. Only 30% of the headaches at stroke onset fulfilled the diagnostic criteria of ICHD-3. We propose new criteria fulfilled by 85% of the headaches. Specificity remained excellent as only two controls had a headache fulfilling the proposed criteria.

Conclusions**:** Existing diagnostic criteria for acute headache attributed to stroke of the ICHD-3 are too insensitive. We suggest new criteria with high sensitivity and preserved specificity.

## P0134 Sex Differences in Spontaneous Intracranial Hypotension

### S. J. Wang^1,2,3^, P. T. Lin^1,2^, Y. F. Wang^1,2,3^, J. W. Wu^2,4^, J. F. Lirng^2,3,5^, S. S. Hseu^6^, S. P. Chen^1,2,3^

#### ^1^Taipei Veterans General Hospital, Department of Neurology, Taipei City, Taiwan; ^2^National Yang Ming Chiao Tung University, School of Medicine, Taipei City, Taiwan; ^3^National Yang Ming Chiao Tung University, Brain Research Center, Taipei City, Taiwan; ^4^Taipei Veterans General Hospital, Department of Medical Education, Taipei City, Taiwan; ^5^Taipei Veterans General Hospital, Department of Radiology, Taipei City, Taiwan; ^6^Taipei Veterans General Hospital, Department of Anesthesiology, Taipei City, Taiwan

##### **Correspondence:** S. J. Wang

Objectives: To determine sex differences in clinical profiles and treatment outcomes following epidural blood patch (EBP) in patients with spontaneous intracranial hypotension (SIH).

Methods: We retrospectively reviewed the medical records of patients with SIH at a tertiary medical center. Demographics, histories, and imaging were collected and compared between sexes. The primary outcome measure was the treatment response to the first EBP. Multivariate logistic regression modeling was performed to predict the first EBP response.

Results**:** Overall, 437 patients with SIH (163 men/ 274 women, mean age 40.3 ± 9.9 years) were identified, and 80 patients (18.3%) had subdural hematoma (SDH). In total, 368 patients (84.2%) received EBP, and 198 (53.8%) responded to the first EBP. Women were less likely to have SDH (11.3% vs. 30.1%, *p* < 0.001), and were more likely to respond to the first EBP (60.9% vs. 41.4%, *p* < 0.001), despite that less blood volume of EBP was injected (27.0 mL (interquartile range (IQR) 23.0-35.0) vs. 31.5 mL (IQR 25.0-37.3), *p* = 0.027) when compared with men. Women (odds ratio (OR) = 2.31) and a higher injected blood volume (OR = 1.03) predicted response to the first EBP by multivariate logistic regression model.

Conclusions: Women with SIH had lower risks for SDH and responded better to the first EBP. Further studies are warranted to understand the underlying pathophysiology.

## P0135 Semiology & outcome of headache in Chiari-malformation type 1

### D. Thunstedt^1^, M. Schmutzer^2^, M. Fabritius^3^, J. Thorsteinsdottir^2^, M. Kunz^2^, R. Ruscheweyh^1^, A. Straube^1^

#### ^1^University Hospital, Ludwig Maximilians University, Department of Neurology, Munich, Germany; ^2^University Hospital, Ludwig Maximilians University, Department of Neurosurgery, Munich, Germany; ^3^University Hospital, Ludwig Maximilians University, Department of Radiology, Munich, Germany

##### **Correspondence:** D. Thunstedt

Background and objective: Congenital anomalies are infrequent causes of symptomatic headache. These include Chiari malformation type 1 (CM1), which usually presents as cough headache but may also mimic other primary headache disorders such as migraine. Previous literature on semiology of headache associated with CM1 is sparse. The aim is to describe headache patterns and to identify factors that influence the course of headache.

Methods: 89 patients diagnosed with CM1 from 2010 until 2021 will be analysed retro - and prospectively. Age, sex, comorbidities, semiology according to ICHD-3 as well as radiological and neurosurgical data will be assessed. Specifically, individual aspects such as side of tonsillectomy, if present, and tonsillar descent are analysed. In the case of syrinx, longitudinal extent is analysed. All of the above data will be evaluated pre - and postoperatively.

Results: Data acquisition is ongoing. Out of 89 patients (58 female, 31 male, age 45.8 years ±17.7 years), 61 patients (68.5%) underwent foramen-magnum decompression (FMD). 52 patients (61.0%) presented with headache. Of these, 10 patients (19.2%) met migraine criteria, whereas 42 patients (80.8%) experienced cough headache. Primary endpoints are the analysis of frequency, intensity and headache-type. Secondary endpoint is to examine factors for headache improvement.

Conclusion: This analysis aims at improving the description of headache semiology and factors that may influence headache in CM1.

## P0136 The effectiveness of blockade of the occipital nerve in the complex treatment of medication-overuse headache

### D. Sotnikov, O. Potapov

#### Sumy State University, Neurosurgery and Neurology, Sumy, Ukraine

##### **Correspondence:** D. Sotnikov

Most treatment-resistant headaches are medication-overuse that require complex treatment.

Objective: to study the effectiveness of great occipital nerve (GON) block in the combined treatment of medication-overuse headache (MOH).

Methods: The study involved 47 patients suffering from MOH, aged 26 to 55 (mean 44,8±6,3) years. The majority of patients were women – 40 (85,1%) persons. The first group (22 persons) received only amitriptyline (average daily dose was 43,8±9,4 mg). Patients in the second group (25 persons) were additionally treated with a bilateral GON blockade with the injection of lidocaine and betamethasone once a week in the first month after withdrawal.

Results: Fourty-one patients completed the study. According to the study, number of days with headache in the first group decreased by 34.5%, in the second group – by 44.3%. The intensity of cephalgia reduced by 22.6% and 33.4% accordingly. We noticed that addition GON blockades were more efficient in patients with allodynia (12 persons) – reducing number of days with headache by 48.7% and intensity of pain by 43.3% against patients without allodynia (9 persons) – number of days with headache decreased by 39.8% and intensity of pain by 23.5%.

Conclusion: Bilateral GON blockade could be recommended for MOH treatment in the first month after withdrawal, especially in patients with allodynia.

## P0137 Altered Speech Patterns in Patients with Post-Traumatic Headache

### C. Chong^1,2^, J. Zhang^2^, J. Li^3^, T. Wu^2^, G. Dumkrieger^1^, S. Nikolova^1^, K. Ross^4^, G. Stegmann^2^, T. J. Schwedt^1^, S. Jayasurija^2^, V. Berisha^2^

#### ^1^Mayo Clinic, Neurology, Phoenix, AZ, United States; ^2^Arizona State University, Phoenix, AZ, United States; ^3^Georgia Tech, School of Industrial and Systems Engineering, Atlanta, GA, United States; ^4^Phoenix VA Health Care System, Phoenix, AZ, United States

##### **Correspondence:** C. Chong

Objective: To interrogate whether speech deficits are detected in patients with Post-Traumatic Headache (PTH) attributed to mild Traumatic Brain Injury and whether there are changes in speech during headache compared to the headache-free state.

Methods: PTH patients and Healthy Controls (HC) provided speech samples using a mobile app over a 30-day period. Vowel and consonant pronunciation, pitch, sentence reading speed, and pause rate measures were extracted from sentence reading and spontaneous speech tasks. Analyses were conducted using a mixed-effect model design.

Results: 1,122 speech samples were collected from 19 PTH (mean age=42.5, SD=13.7; time post-mTBI at enrollment= 14 days; and 31 HC subjects (mean age=38.1, SD=12.2). Regardless of headache presence or absence, PTH patients had longer pause rates (p=.049) and alterations in vowel (p=.037) and consonant (p=.0062) pronunciation relative to HC. During headaches there were longer pause rates (p=.018), slower sentence reading rates (p=.037), and less precise vowel (p=.049) and consonant (p=.018) pronunciation compared to the speech of HC. During headache, PTH patients had slower sentence reading rates (p=.0071) and less accurate vowel pronunciation (p=.0034) compared to when they were headache-free.

Conclusions: Speech features are altered in PTH during and between headaches. Speech features might be useful proxy measures for prognosticating PTH recovery.

**Fig. 1 (abstract P0137).**
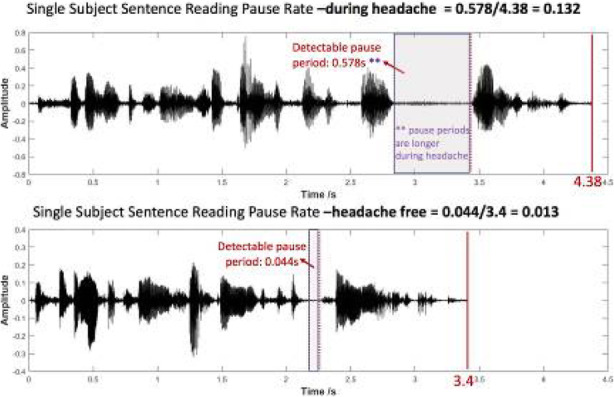
See text for description

## P0138 Psychological changes in adolescents with a tension-type headache after heart rate variability biofeedback training

### K. Stepanchenko, V. Marchenko

#### Kharkiv Medical Academy of Postgraduate Education, Kharkiv, Ukraine

##### **Correspondence:** K. Stepanchenko

Background and objective: Some evidence suggests that heart rate variability biofeedback-based training (HRV-BBT) might be an effective way to treat headaches and psychological symptoms. The aim to examine the effect of HRV-BBT on anxiety and depression in adolescents with tension-type headache (TTH).

Methods: 118 adolescents were examined. We formed four groups of adolescents with episodic (ETTH) and chronic TTH (CTTH) who received only drug therapy and only HRV-BBT and 5th group – adolescents with CTTH who received a combination of drug and HRV-BBT. The intensity of the pain (VAS); the level of reactive and personal anxiety (self-esteem scale Spielberger-Hanin); the level of depression (scale V.A. Zhmurova) were performed.

Results: We observed a decrease in the level of anxiety in adolescents with TTH after HRV-BBT. However, only the reduction in reactive anxiety was significant (ETTH: before and after treatment – 42,6±7,5 and 33,5±5,4, р<0,05; CTTH: 37,7±6,8 and 29,2±6,8, р<0,05). The level of depression was significantly reduced after HRV-BBT in adolescents of all groups (ETTH: before and after treatment – 20,2±4,7 and 14,4±3,9, р<0,05; CTTH: 24,9±5,3 and 9,4±3,8, р<0,05), and the use of pharmacotherapy had a positive effect only in the group with CTTH who more often received amitriptyline.

Conclusions: Our findings support the beneficial impact of HRV-BBT on anxiety and depression for adolescents with TTH with higher effectiveness in adolescents with episodic forms.

## P0139 Psychological predictors of real-life experience with Erenumab in chronic migraine with or without medication overuse: data from a 1-year follow-up

### S. Bottiroli^1,2^, R. Deicco^1,3^, G. Vaghi^1,3^, G. Fiammingo^1,3^, E. Guaschino^1^, M. Allena^1^, N. Ghiotto^1^, C. Tassorelli^1,3^, G. Sances^1^

#### ^1^IRCCS Mondino Foundation, Headache Science and Neurorehabilitation Centre, Pavia, Italy; ^2^Giustino Fortunato University, Faculty of Law, Benevento, Italy; ^3^University of Pavia, Department of Brain and Behavioral Sciences, Pavia, Italy

##### **Correspondence:** S. Bottiroli

Background/objective: To evaluate the psychological predictors of the outcome of real-life experience with the anti-CGRP monoclonal antibody Erenumab in a 1-year follow-up in chronic migraine (CM).

Methods: Seventy-one CM (ICHD-III criteria) patients (age:49.1±9.5) with or without medication overuse who had already failed at least 3 preventive therapies received Erenumab (70 or 140-mg dose s.c.). At T0 patients received a psychological evaluation comprising mood, anxiety, and personality disorders, alexithymia, childhood traumas and current stressors.

Results: At the 1-year follow-up, 50 patients (age:49.0±9.5) reported a reduction of at least 50% in migraine days/month (Responders, R); whereas 21 (age:49.3±9.7) did not (non Responders, NR). When compared to R, NR were characterized by a higher prevalence of anxiety (90% vs 60%, *p*=.012) and Cluster C (avoidant, dependent, and obsessive-compulsive) personality disorders (87% vs 38%, *p*=.002). They also showed more alexithymic traits (53.2±12.9 vs 43.7±14.2, *p*=.03) and a higher number of stressors (1.2±2.5 vs 0.3±0.7, *p*=.012). The two groups were similar for mood disorders and childhood traumas.

Conclusions: Erenumab is an effective option for patients with difficult-to-treat migraine. Our findings show a further distinction within these patients, highlighting the impact of current stressors, anxiety and an "anxious-fearful" personality in those CM patients being refractory to many preventive treatments, including Erenumab.

## P0140 Subjective cognitive impairment in patients with transformed migraine and the associated psychological and sleep disturbances

### M. ELSherif^1^, M. Abdelsalam^1^, A. Shoukri^2^, A. Esmael^1^

#### ^1^Mansoura University faculty of Medicine, Neurology, Mansoura, Egypt; ^2^Ain Shams University, Chest, Cairo, Egypt

##### **Correspondence:** M. ELSherif

This study aimed to evaluate the association of subjective cognitive impairment (SCI) with depression, anxiety, and modalities of sleep in those who have transformed migraines (TM). Subjects and methods The study was conducted on 120 participants with TM and 41 control group participants. The subjective cognitive decline questionnaire classified the participants as SCI and non-SCI. The Headache Impact Test-6, MigraineDisability Assessment, Montreal Cognitive Assessment, Mini-Mental State Examination, Patient Health Questionnaire-9, Pittsburgh Sleep Quality Index, Epworth Sleepiness Scale, Full Polysomnography, and Beck"s Anxiety and the Depression Inventories were used and analyzed between patients with SCI and non-SCI. Results Patients with TM who had SCI represented 34% with severe headache effects, disability, pain severity, increased depression and increased anxiety. They showed shorter sleep duration during weekdays, lower sleep quality, less sleep time, lower efficiency, and less REM sleep along with greater sleep latency, periodic limb movements, a higher arousal index, snore index, and percent of NREM3. There was a positive correlation between certain polysomnography parameters like percent NREM3, sleep period, sleep index, sleep latency, sleep arousal index, and periodic limb movements, and an inverse correlation with the percent of REM sleep, total sleep time, and sleep efficiency. Conclusion Subjective cognitive complaints are common in patients with transformed migraine affecting about 34% of cases.TM patients with SCI had more sleep and psychological disturbances.

**Fig. 1 (abstract P0140).**
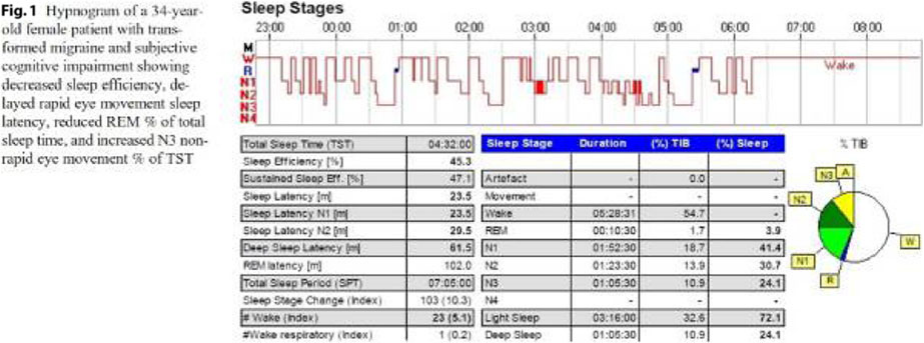
See text for description

## P0141 Clinical features of medication overuse headache following overuse of different acute symptomatic headache drugs

### S. Y. Oh^1,2^, J. J. Kang^1,2^, H. K. Park^3^, S. J. Cho^4^, M. K. Chu^5^

#### ^1^Jeonbuk National University Hospital, Neurology, Jeonju, South Korea; ^2^Jeonbuk National University Hospital, Research Institute of Clinical Medicine, Jeonju, South Korea; ^3^Inje University Ilsan Paik Hospital, Inje University College of Medicine, Neurology, Seoul, South Korea; ^4^Dongtan Sacred Heart Hospital, Hallym University College of Medicine, Neurology, Hwaseong, South Korea; ^5^Severance Hospital, Yonsei University College of Medicine, Neurology, Seoul, South Korea

##### **Correspondence:** J. J. Kang

Background: Medication overuse headache is a growing problem worldwide, although some recent clinical studies gave insights that clinical features seem to vary significantly depending on the type of overused drug, however, there is still controversy.

Objective: To investigate and compare the clinical characteristics of patients with MOH following overuse of different acute headache drugs.

Methods: This cross-sectional observation study prospectively collected demographic and clinical questionnaire data from 114 consecutive patients with MOH according to IHS criteria between May 2020 and January 2021.

Results: A total of 105 MOH patients were included in this study. The patients are associated with the overuse of triptans (29.5%), ergot alkaloids (7.6%), simple or combination-analgesics (35.2%), opioids (0.9%) and combination of them (26.7%). The MDMOH was significantly longer for analgesics (10.6 years) than other drugs for triptans (4.3 years) or ergots (4.1 years) (*p*=0.011). The MMFSH was lowest for triptans (7.4 days per month), higher for ergots (8.9 days), and highest for analgesics (14.4 days) (*p*=0.005). The MMFMedS for visiting headache clinic was highest for combination of multiple drugs than simply triptans or analgesics (*p* = 0.008). The MCMIF was most frequent in the combination of multiple drugs group (25 days per month) and the lowest in the triptans (18.1 days) (*p*=0.007).

Conclusion*:* Clinical characteristics of MOH are linked to acute medication class ingested.

## P0142 Association between migraine and psychological and behavioural factors

### G. D'Aurizio, G. Saporito, F. Pistoia, R. Ornello, V. Caponnetto, F. Bruno, A. Splendiani, S. Sacco

#### University of L'Aquila, Department of Applied Clinical Sciences and Biotechnology, L'Aquila, Italy

##### **Correspondence:** G. Saporito

Background: Migraine is a neurological disorder that influences the patient's well-being, often leading to stress and discomfort. A relationship between migraine and sleep quality has been reported, as well as a frequent association of migraine with anxiety and mood alterations.

Method: Sixty-five patients with Episodic Migraine (EM;65F;43.9±7.2), 65 with Chronic Migraine (CM;65F;47.8±8.5), and 65 Healthy Controls (HC;65F;43.7±9.3) were assessed using the Pittsburgh Sleep Quality Index(PSQI), the Insomnia Severity Index(ISI), the Epworth Sleepiness Scale (ESS), the Intolerance of Uncertainty Inventory (IUI-10), the Intolerance of Uncertainty Scale-12(IUIS12), the URS Scale, the IA Questionnaire, the Eysenck Personality Questionnaire(EPQ-R), the State-Trait Anxiety Inventory (STAI-2), the Anxiety Sensitivity Index-3 (ASI-3), the Brief-Temps, the General Decision Making Style (GDMS), the Pain Catastrophizing Scale (PCS).

Results: A statistically significant difference among the three groups has been found in the score obtained: PSQI (p<.001), ISI (p=.002), EPQ-R/P (p=.045), STAI-2 (p=.002), ASI-3-PH (p=.01); ASI-3-ME (p<.001), PCS TOT(p<.001), TEMPS TOT (p<.001).

Conclusions: It is plausible to hypothesize that CM shows a reduced quality and quantity of sleep as compared to the other groups. Moreover, CM seems to show an increase in arousal in response to environmental stimuli combined with mood instability, with an ensuing tendency to accentuate the severity and perception of pain.

## P0143 Impaired decision-making under ambiguity but not under risk in patients with medication overuse headache

### C. I. Lau^1,2^, W. H. Chen^1^, V. Walsh^2^

#### ^1^Shin-Kong Wu Ho-Su Memorial Hospital, Dementia Center, Department of Neurology, Taipei City, Taiwan; ^2^University College London, Institute of Cognitive Neuroscience, London, United Kingdom

##### **Correspondence:** C. I. Lau

Background and Objective: The loss of control over analgesics and high relapse rates after withdrawal in patients with medication overuse headache (MOH) may indicate a dependency-like behaviour. Whether patients with MOH exhibit similar decision-making impairment as substance use disorders is still controversial. Decision-making depends upon the degree of uncertainty: 1) under ambiguity where probability of outcome is unknown and 2) under risk where probabilities are known. The present study is the first one to examine both types of decision-making in MOH.

Methods: We investigated 25 patients with MOH, 25 patients with chronic migraine (CM) and 25 matched healthy controls (HC) with two different decision-making tasks. Decision-making under ambiguity was assessed with the Iowa Gambling Task (IGT) that involves emotional feedback processing, whereas decision-making under risk was assessed with the Cambridge Gambling task (CGT) that involves executive supervision.

Results**:** In comparison to CM and HC, MOH showed significantly more disadvantageous decisions under ambiguity in the IGT (figure 1), whereas all three groups performed similarly in the CGT (figure 2).

Conclusions: Our behavioural data suggest that MOH presents with decision-making deficits in situations under ambiguity, but not under risk. This dissociation indicates disrupted emotional feedback processing rather than executive dysfunction which may contribute to the pathogenesis of MOH.

**Fig. 1 (abstract P0143).**
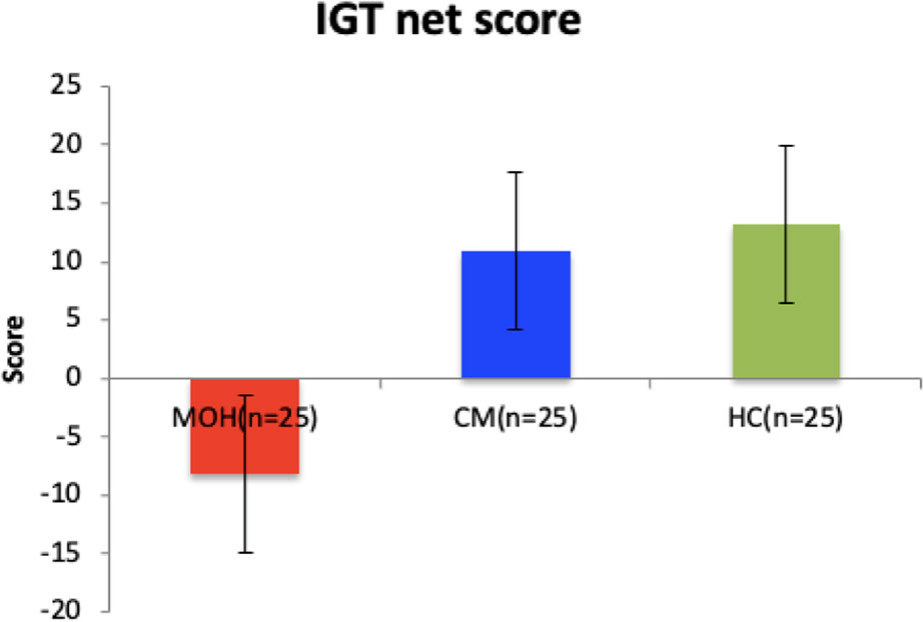
See text for description

**Fig. 2 (abstract P0143).**
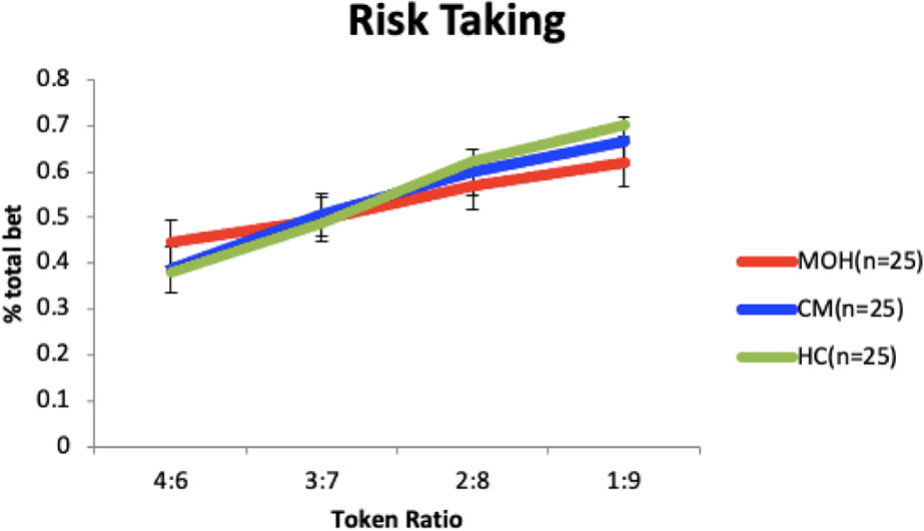
See text for description

## P0144 Efficacy of cognitive-behavioral therapy for the prophylaxis of migraine in adults: a randomized controlled trial

### T. Klan^1^, C. Gaul^2^, E. Liesering-Latta^2^, B. Both^1^, I. Held^1^, M. Witthöft^1^

#### ^1^University of Mainz, Department of Psychology, Mainz, Germany; ^2^Migraine and Headache Clinic, Koenigstein im Taunus, Germany

##### **Correspondence:** T. Klan

Objective: Efficacy of a newly developed migraine-specific cognitive behavioral therapy (CBT) program combining several approaches (education and counselling, coping with fear of attacks, trigger management) was evaluated.

Methods: N=121 adults with migraine were randomized to either CBT, or relaxation training (RLX), or a waiting-list control-group (WLC). The outpatient group therapy (CBT or RLX) comprised seven sessions each 90 minutes. Participants who completed the WLC-group were subsequently randomized to CBT or RLX. Baseline was compared to post-treatment, and followed by assessments 4- and 12-months post-treatment. Main outcomes are headache days, disability by the Headache Disability Inventory (HDI), and self-efficacy by the Headache Management Self-Efficacy Scale (HMSE-G-SF).

Results: N=97 participants completed the pre-post assessment. The pre-post analyses showed higher self-efficacy (HMSE-G-SF) in both treatments (CBT: p=0.021; RLX: p=0.006) compared to the WLC. The follow-up analyses yielded reductions from pre after 12-months (N=77 completer) in headache days (–1.84 days, p<0.001) and disability by HDI (–11.70 points, p<0.001) for the completer (CBT and RLX), whereas there was no significant difference between both treatment groups.

Conclusion: Migraine-specific CBT and RLX have similar, moderate long-term effects in migraine-prophylaxis. Thus, CBT may be a promising alternative for patients who are demanding for a more tailored behavioral intervention.

## P0145 Visual Quality of Life in Patients with Visual Snow Syndrome

### K. S. Hu^1^, C. Martindale^2^, M. M. Cortez^2^, K. B. Digre^1^

#### ^1^John A. Moran Eye Center, University of Utah, Ophthalmology, Salt Lake City, UT, United States; ^2^University of Utah, Neurology, Salt Lake City, UT, United States

##### **Correspondence:** K. S. Hu

Background and Objective: Visual snow is a syndrome of unremitting positive visual phenomena that involve the entire visual field. It is often co-morbid with headache, depression, and anxiety. Visual quality of life has not been evaluated in this population.

Methods: An electronic survey was created with questions about visual snow symptoms and previously validated questionnaires including the Headache Impact Test (HIT-6), Visual Function Questionnaire-25 (VFQ-25), and Utah Photophobia Symptom Impact Scale-12 (UPSIS-12). Patients were identified via electronic health record starting from July 2015. Inclusion criteria included age >18. Exclusion criteria included concurrent ophthalmic disease other than refractive error or dry eye.

Results: Response rate was 65% (32/49 of invited subjects). 72% were female; mean age was 35. 69% of patients carried a prior headache diagnosis, while 75% reported having tinnitus. Median composite VFQ-25 scores were lower than published population-based values (Hirneiss et al. 2010), and were inversely correlated with HIT-6 (r=-0.44, p=0.012) and UPSIS-12 (r=-0.67, p<0.001) scores. Correlations with VFQ-25 and PHQ-9 and GAD-7 were not statistically significant.

Conclusions: Visual snow is associated with reduced visual quality of life. Tinnitus and headache are co-morbid conditions prevalent in patients with visual snow. Visual quality of life worsened with increased headache and light sensitivity; there was no correlation with affective symptoms.

## P0146 CASE REPORT: Galcanezumab for a chronic headache in idiopathic intracranial hypertension

### T. Lima, M. Peres

#### Hospital Israelita Albert Einstein, Neurologia, São Paulo, Brazil

##### **Correspondence:** T. Lima

Despite the significant headache morbidity in Idiopathic intracranial hypertension (IIH), there is no evidence-based treatment for long-term headache management. We report a case in which a patient with HII persisted with daily headache, even ocular remission (resolved papilledema), and presented a good response to galcanezumab.

44 years old, female, with episodic migraine, presents a change in headache pattern, with an important increase in intensity and poor response to simple analgesics. Concomitant to the new headache pattern, there was deterioration of visual acuity. After conducting an investigation for secondary headache, she was diagnosed with idiopathic intracranial hypertension. After pharmacological treatment with Topiramate and acetazolamide, there was an important improvement in visual acuity, but no improvement in headache. Headache persisted, with daily frequency, even after optimization of drug treatment for three months. In this context, galcanezumab 240mg dose attack was initiated. After 1 month of starting the medication, the patient returns referring to only 1 day of headache during the 30-day period, no side effects reported.

A prospective open-label study of erenumab in IIH patients with persistent headaches in whom their papilledema has resolved, demonstrates significant efficacy to reduce headaches. There is no study with galcanezumab. Further studies are needed to assess the effectiveness of monoclonal antibodies in HII.

## P0147 Theory of mind: A new perspective on cluster headache

### S. Ballesta-Martínez, M. P. Navarro-Pérez, E. Bellosta-Diago, J. Rodríguez Montolio, E. Jiménez Jara, J. Espinosa-Rueda, S. Santos-Lasaosa

#### HCU Lozano Blesa, Neurology, Zaragoza, Spain

##### **Correspondence:** S. Ballesta-Martínez

Objective: Theory of mind (ToM) is the ability to attribute mental states of self and others, such as beliefs (cognitive ToM) and feelings (affective ToM). Based on the role of hypothalamus in pain and social cognition, our aim is to determine whether ToM is impaired in patients with episodic cluster headache (ECH).

Methods: We conducted a case-control study in which 17 patients and 11 matched controls carried out social cognition tasks (Reading the Mind in the Eyes test -RMET- to evaluate affective ToM and Hinting task for cognitive ToM) as well as the Symbol Digit Modalities Test (SDMT) to assess cognitive performance and Hospital Anxiety and Depression Scale (HADS). Demographic and clinical characteristics were also recorded. Statistical analysis was performed using SPSS package.

Results: All participants were male; mean age was 48.2±8 in the control group and 50.2±10.9 in the patient group. Patients had had an attack free period of at least 1 month (mean attack free period 9.5±12.9 months). We found no differences in RMET (p=0.152), HADS Anxiety score (p=0.107) nor HADS Depression score (p=0.530). We found differences in Hinting task (p=0.006) and SDMT (p=0.001).

Conclusion: Our results suggest that ECH patients can perceive other people"s or one"s own feelings (affective ToM) but have difficulties at recognizing beliefs (cognitive ToM). These deficits are not apparently attributable to depression or anxiety states yet are in accordance with worst cognitive performance.

## P0148 Psychosocial variables and healthcare resources in patients with cluster headache and in patients with migraine

### E. Calandre^1^, J. Garcia-Leiva^1^, J. Ordoñez-Carrasco^2^, L. Guapacha-Borrero^1^, M. A. de Pascual^3^

#### ^1^Universidad de Granada, Instituto de Neurociencias, Armilla, Spain; ^2^Universidad de Almeria, Department of Psychology, Almeria, Spain; ^3^Asociacion de Cefalea en Racimos y Primarias, Madrid, Spain

##### **Correspondence:** E. Calandre

Background & objective: To compare the burden caused by cluster headache (CH) and migraine (M), by assessing different psychosocial variables and the use of healthcare resources.

Methods**:** An online survey was uploaded in the website of the Spanish Association of Cluster Headache and other Primary Headaches. It included sociodemographic data, the Patients Health Questionnaire-9, the Insomnia Severity Index, the EuroQOL-5D-5L, and a questionnaire evaluating the use of different healthcare resources (family doctor visits, specialists visits, emergency room visits, medical analyses, hospitalization, and surgical interventions) during the past six months. Patients experiencing other associated headaches or central sensitization syndromes were excluded.

Results**:** Thirty-nine CH patients (25-45 years, 88.9% male) and 27 M patients (20-52 years, 61.5% females) were evaluated. Mean scores for depression and insomnia were clinically relevant in both groups, but significantly higher among CH patients, as was the percentage of subjects reporting suicidal ideation. EQ-5D-5L and EQ-5D-5L VAS scores were lower than the reported mean population values but did not differ between both patients groups. CH patients reported significantly more visits to the family physician and surgical interventions than M patients.

Conclusion: Although both CH and M had a relevant impact on patients wellbeing, the burden of CH was greater than M. CH patients also required greater medical attention.

## P0149 Cluster headache: comorbidity with migraine and/or fibromyalgia and psychosocial burden

### E. Calandre^1^, J. Garcia-Leiva^1^, J. Ordoñez-Carrasco^2^, L. Guapacha-Borrero^1^, M. A. de Pascual^3^

#### ^1^Universidad de Granada, Instituto de Neurociencias, Armilla, Spain; ^2^Universidad de Almeria, Department of Psychology, Almeria, Spain; ^3^Asociacion de Cefalea en Racimos y Primarias, Madrid, Spain

##### **Correspondence:** E. Calandre

Background & objective**:** Cluster headache (CH), migraine (M) and fibromyalgia (FM) can coexist. We aimed to evaluate the comorbidity of CH with M and/or FM and the impact of each disease groups.

Methods**:** An online survey was uploaded in the website of the Spanish Association of Cluster Headache and other Primary Headaches. It included sociodemographic data, the Patients Health Questionnaire-9, the Insomnia Severity Index, the EuroQOL-5D-5L, and a questionnaire evaluating the use of different healthcare resources (family doctor visits, specialists visits, emergency room visits, medical analyses, hospitalization, and surgical interventions) during the past six months.

Results**:** Of 91 CH patients 39 (42.8%) had only CH, 15 (16.5%) had CH+M, 10 (10.9%), had CH+FM and 27 (29.7%) had CH+M+FM. In contrast with non-comorbid CH, female sex predominated in comorbid CH. Medical comorbidities were significantly more frequent among CH+FM, and CH+M+FM than in non-comorbid CH. Depression and suicidal ideation were frequent in all groups without differences among them. Insomnia, also common to all groups, was significantly higher in CH+M+FM group. EQ-5D-5L and EQ-5D-5L VAS scores were low in all groups but significantly lower in CH+M+FM. Medical analyses were more frequent in CH+FM and CH+M+FM.

Conclusion**:** Comorbidity with M or FM was frequent among CH patients, being female sex a risk factor for comorbid M or FM. Patients with CH+M+FM had the worse ratings in insomnia and quality of life.

## P0150 CGRP monoclonal antibodies off-label use in patients with hemicrania continua and chronic cluster headache

### N. Vashchenko^1,2^, K. Skorobogatykh^2^, J. Azimova^2^

#### ^1^Sechenov University, Neurological department, Moscow, Russian Federation; ^2^University Headache Clinic, Moscow, Russian Federation

##### **Correspondence:** N. Vashchenko

Background and objectives: Monoclonal antibodies that target calcitonin gene-related peptide (CGRP) were recently approved for migraine prevention. Erenumab and Fremanezumab were registered in Russia in August 2020 and are currently available for purchase in pharmacies. We want to report the results of their off-label use in patients with hemicrania continua and chronic cluster headache (CCH).

Methods**:** Two men (30 and 38 years old) with CCH, both using 960mg of Verapamil with mild effects, and two women (21 and 33 years old) with hemicrania continua with mild Indomethacin effect were consulted in the headache clinic. Two patients (1 with CCH and 1 with hemicrania continua) were prescribed off-label Erenumab 70 mg and two others Fremanezumab 225 mg, monthly injections, considering their safety and efficacy in clinical trials.

Results: All patients had a significant improvement after the first injection.CCH patients had a meaningful reduction in attack frequency (from 1-2 attacks per day to up to 10 days pain-free episodes) and better medication response (100mg of oral Sumatriptan or even external trigeminal nerve stimulation alone can stop the attack). Both patients with hemicrania continua have only 2-3 headache days per month (comparing to 30 days before the injections).

Conclusions: The use of CGRP monoclonal antibodies may be effective in patients with hemicrania continua and chronic cluster headache. More clinical trials are needed to prove efficacy on a large group of patients.

## P0151 Erenumab for chronic refractory cluster headache – case report

### A. R. Gonçalo Pinheiro, Â. Abreu, E. Parreira

#### Hospital Professor Doutor Fernando Fonseca, Neurology, Lisbon, Portugal

##### **Correspondence:** A. R. Gonçalo Pinheiro

Introduction: CGRP is released after trigeminal-autonomic reflex activation during a cluster headache attack. Galcanezumab has shown positive results in episodic cluster headache. Erenumab has also been described as effective in cluster headache and comorbid migraine. We present a case of off-label use of Erenumab for chronic cluster headache treatment, refractory to all treatments, except corticosteroids.

Case: A 63-year-old male developed in 2015 a chronic cluster headache: he presented daily headache, ranging from one attack per night in the first year, to several nocturnal and diurnal attacks in the following two years. He was medicated with verapamil, melatonin, lithium, topiramate and occipital nerve block, without success. He then started oral corticosteroids with efficacy but become dependent of this medication. Valproate and botulinum toxin were also tried. In 2020 he started Erenumab 140mg, after informed consent. After one-week, complete resolution of the attacks occurred, and it lasted 10 weeks, when he was able to stop corticosteroids. After 9 months of treatment, he shows a significant reduction in frequency and intensity of the attacks.

Conclusion: In this case, Erenumab allowed control of refractory cluster headache and suspension of corticosteroids. We emphasize that, because CGRP is involved in the pathophysiology of the disease, anti-CGRP therapies may improve its treatment.

## P0152 Absence of structural correlatable findings in Cluster Headache patients fulfilling IHS Criteria: Experience in three different Hospitals in Spain

### S. Pérez-Pereda^1^, V. González-Quintanilla^2^, M. Drake^3^, C. Serrano^4^, C. N. Marzal^4^, S. Cusó^4^, C. Aguilella^4^, M. Fernández Recio^5^, G. Velamazán^5^, J. Pascual^1^

#### ^1^University Hospital Marqués de Valdecilla and University of Cantabria, Neurology, Santander, Spain; ^2^University Hospital Marqués de Valdecilla and IDIVAL, Neurology, Santander, Spain; ^3^University Hospital Marqués de Valdecilla and IDIVAL, Radiology, Santander, Spain; ^4^Fundació Hospital Sant Joan de Déu de Martorell, Neurology, Martorell, Spain; ^5^University Hospital Virgen de Valme, Neurology, Sevilla, Spain

##### **Correspondence:** S. Pérez-Pereda

Background and objective: In contrast to migraine, brain MRI is recommended for the initial diagnosis of Cluster Headache (CH) to exclude other conditions that could mimic its symptoms. Our aim was to analyze the true value of MRI in CH.

Methods: We analysed the brain MRIs of consecutive patients diagnosed with CH according to current IHS criteria in 3 Headache Units in Spain and exhaustively reviewed their clinical history.

Results: 134 patients were included. 49 (37%) showed some abnormal finding. 43 were male; mean age at diagnosis 42+/-14y. 6 were chronic and 43 episodic CH. MRI findings were: 18 white matter lesions, 12 sinus inflammatory changes, 5 small arachnoid cysts, 5 chronic ischemic lesions, 3 empty sella turca, 2 tumors (trigeminal schwannoma and craneopharyngioma), 2 diffuse cortico-subcortical atrophy and 5 other unspecific findings. All of them were considered non symptomatic based on the neuroimaging characteristics, the clinical course and/or the response to conventional treatment. Patients who showed tumors presented atypical features (facial hypoesthesia on examination and episodes of prolonged duration that progressed to continuous refractory pain without specific pattern, respectively) and they did not fulfill, retrospectively, IHS CH criteria.

Conclusions: Brain MRI in patients who meet the IHS CH criteria, with no atypical features, does not show any correlatable findings, suggesting that these criteria are highly predictive of its primary origin.

**Fig. 1 (abstract P0152).**
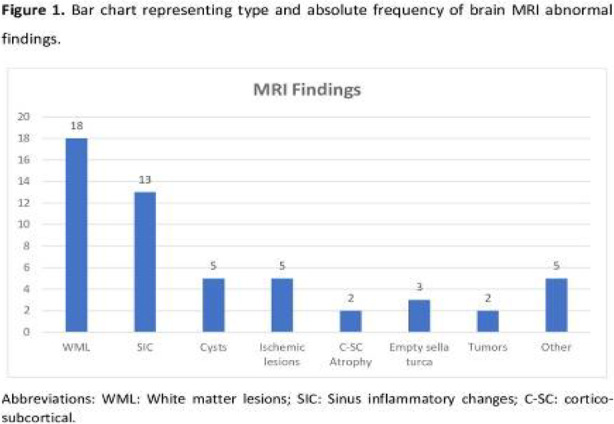
See text for description

**Table 2 (abstract P0152).**
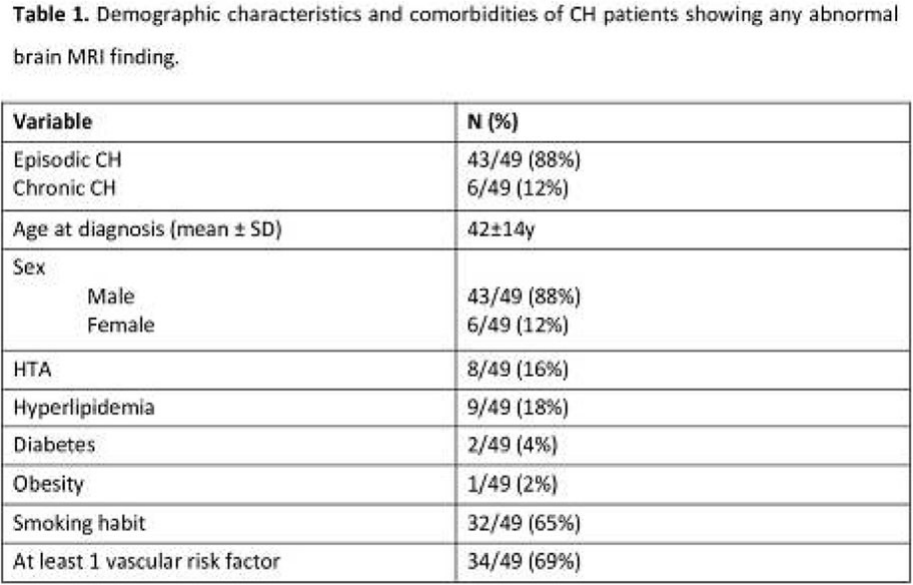
See text for description

## P0153 Chronic Cluster Headache in Woman: A Case Report

### D. A. Sudibyo, H. B. Hidayati

#### Medical Faculty Airlangga University/ Dr. Soetomo General Hospital Surabaya, Indonesia, Neurology, Surabaya, Indonesia

##### **Correspondence:** D. A. Sudibyo

Background and objective: Cluster headache is the most frequent trigeminal autonomic headache syndromes, with high morbidity due to its pain severity. Chronic cluster headache, comprised of 10-20% patients, can be more difficult to control, mandates for efficient prophylactic therapy for the patient. We present a woman with chronic cluster headache with successful verapamil prophylactic treatment.

Case report: A 53 years old woman, admitted in neurology outpatient clinic, complained of 2 years severe left periorbital pain in temporal region with Numeric Pain Rating Scale (NPRS) 10, accompanied by autonomic symptoms (conjunctival injection, tearing, nausea, hyperhidrosis), lasting 45 minutes – 2 hours (if untreated), twice a day especially at night, improved with oxygen therapy during acute attack. Neurological examination and head computed tomography (CT) scan with contrast were normal. Due to her worsening periodicity for the past 2 weeks, prophylactic treatment with verapamil 80 mg twice daily was commenced which give an excellent remission of symptoms and reduced pain intensity within 14 days (NPRS was reduced to 0 with no cluster attack for one month follow up).

Conclusions: Verapamil 80 mg twice daily can be used as prophylactic treatment for chronic cluster headache with good result and less side effect. It gives significant reduction on pain intensity followed by no cluster attack.

Keywords: Cluster headache, Verapamil, Prophylactic treatment

**Fig. 1 (abstract P0153).**
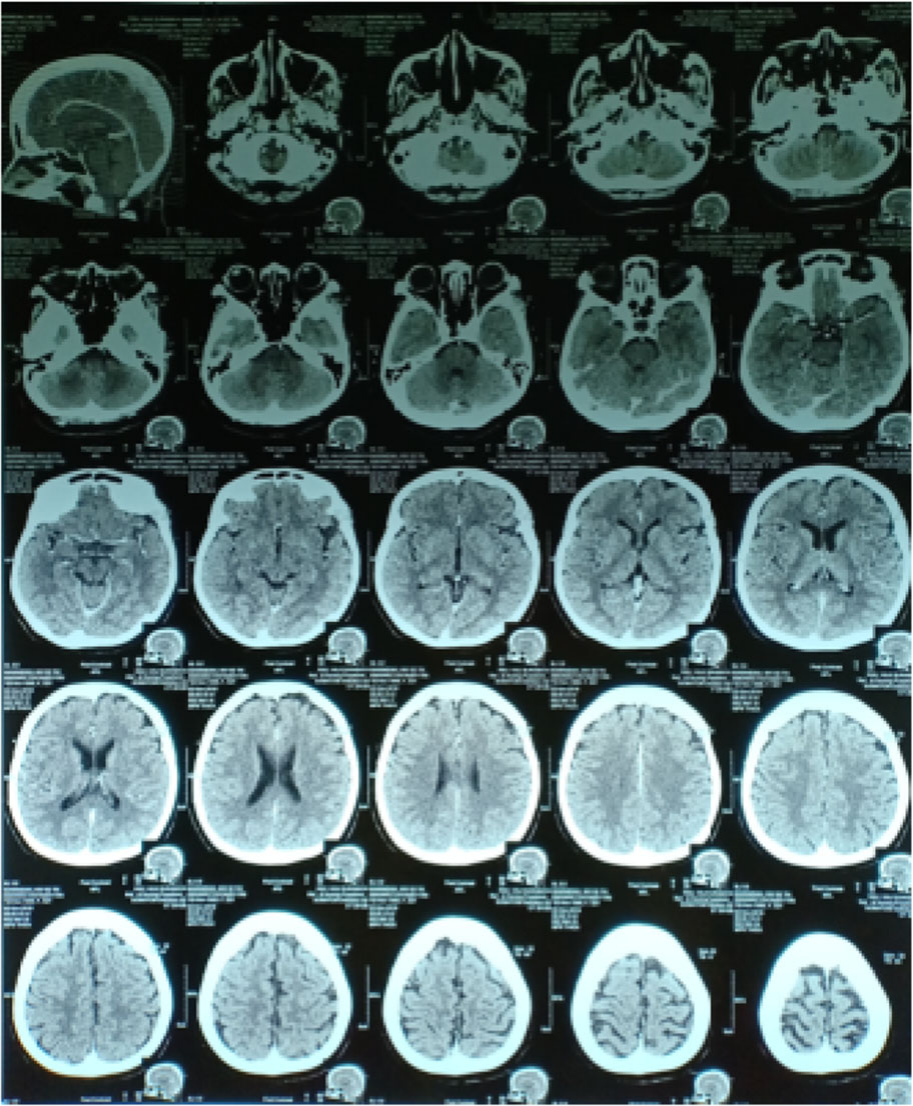
See text for description

## P0154 Intranasal ketamine for acute Cluster headache attacks - Results from a proof-of-concept open label trial

### A. S. Petersen^1^, A. Snoer^1^, A. S. Pedersen^1^, M. Barloese^1,2^, P. Holm^3^, O. Pedersen^3^, R. H. Jensen^1^

#### ^1^Danish Headache Center, Departement of Neurology, Glostrup, Denmark; ^2^Hvidovre Hospital, Department of Functional and Diagnostic Imaging, Section for Clinical Physiology and Nuclear Medicine, Hvidovre, Denmark; ^3^Lionheart Pharmaceuticals ApS, Vanløse, Denmark

##### **Correspondence:** A. S. Petersen

Background and objective: Acute treatment options for Cluster headache patients who have an insufficient response to oxygen and triptans are limited. Intranasal ketamine has anecdotally been successful in treating a Cluster headache attack but never systematically tested.

Methods: We conducted an open-label pilot study in which 20 chronic Cluster headache patients according to International Classification of Headache Disorders 3rd were treated during one cluster headache attack with intranasal ketamine. Under in-hospital observation patients received 15 mg ketamine by intranasal spray every six minutes a maximum of five times. The primary endpoint was a 50% reduction of pain intensity within 15 minutes after initiating treatment.

Results: The primary endpoint was not met. However, 30 minutes after first application the pain intensity was reduced by 59% from 7.25±1.24 to 2.94±3.40 on a 11 points numeric rating scale (mean, SD, p=0.0002) and 11 out of 16 (69%) scored four or below on the numeric rating scale. Exactly, half the patients preferred ketamine to oxygen and/or sumatriptan injection and complete relief was self-reported by 8 out of 20 patients (40%). No serious advense events were identified during the trial.

Conclusion: Intranasal ketamine may be an effective acute treatment of cluster headache within 30 minutes but should be tested in a larger controlled design. Patients and physicians should be conscious of the abuse potential of ketamine.

**Fig. 1 (abstract P0154).**
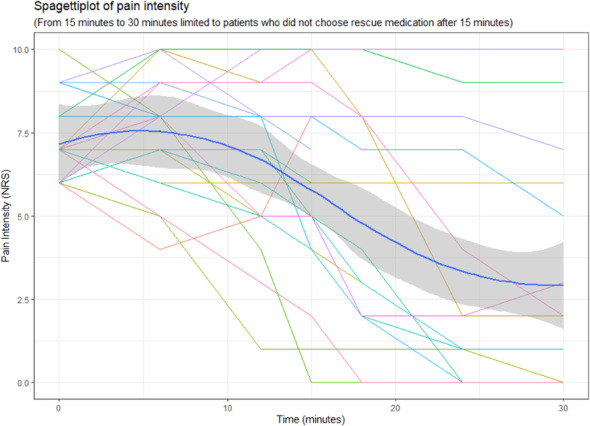
See text for description

**Fig. 2 (abstract P0154).**
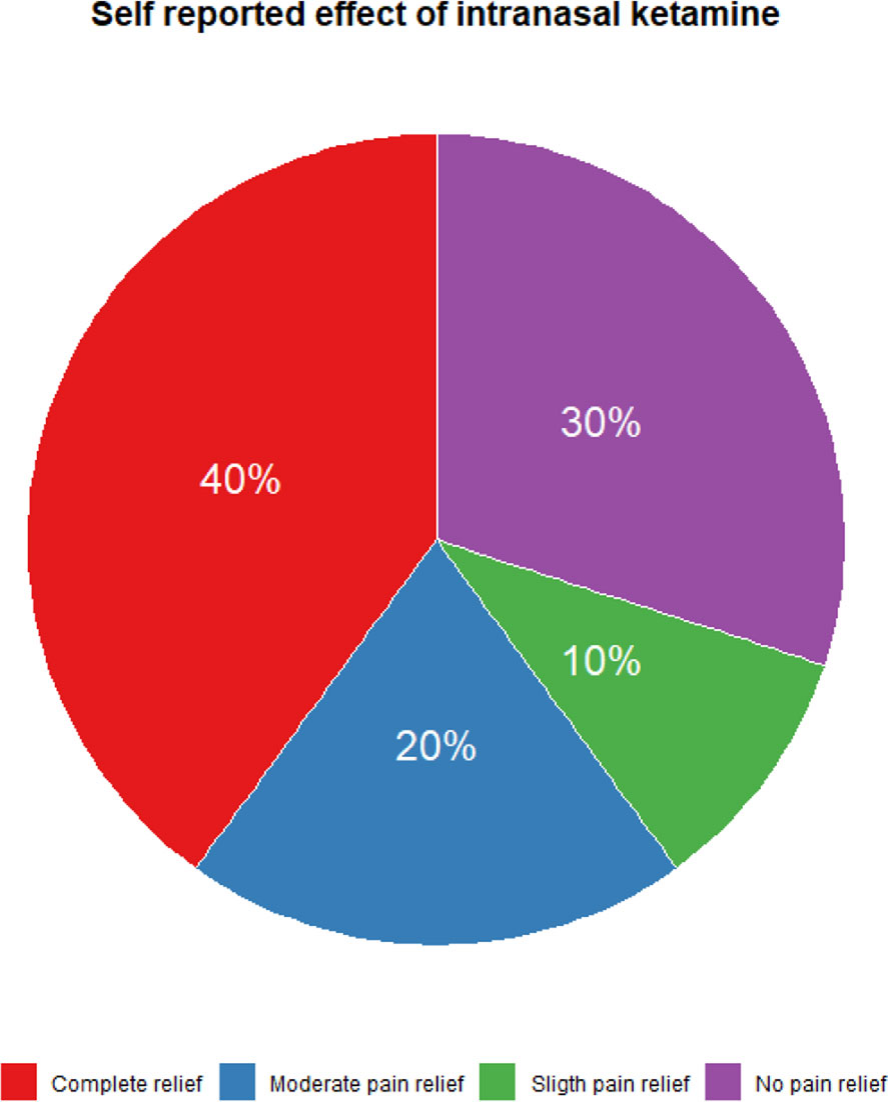
See text for description

## P0155 Greater Occipital Nerve blocks for treatment in cluster headache: an observational prospective study

### G. M. Asioli^1^, G. Urbinati^1^, V. Favoni^2^, G. Pierangeli^1,2^, S. Cevoli^2^

#### ^1^University of Bologna, Department of Biomedical and Neuromotor Sciences, Bologna, Italy; ^2^IRCCS Istituto delle Scienze Neurologiche di Bologna, UOC Clinica Neurologica Rete Metropolitana NEUROMET, Bologna, Italy

##### **Correspondence:** G. M. Asioli

Background and objective: Greater occipital nerve blockade (GONB) can be used for transitional treatment in cluster headache (CH). A wide range of GONB protocols are described, and it is uncertain which leads to better results. In this observational prospectivestudy we aim to evaluate the effectiveness and safety of GONB with methylprednisolone (MP) and lidocaine in CH.

Methods: We consecutively recruited patients accessed to our Headache Centre for episodic (ECH) or chronic CH (CCH). Patients underwent to GONB with slow-release MP 80 mg and Lidocaine 40 mg. Primary outcome was the absence of CH attacks at one month. Secondary outcome was the reduction of at least 50% of daily attacks.

Results: A total of 32 patients were recruited: 23 with ECH and 9 with ECH. Ten patients (31%) were attacks free at one month, while a total amount of 19 patients (59%) show a reduction of at least 50% of daily attacks. In non-attack-free patients, daily frequency of attacks decreased from a median of 2 (IQR:1-3) to 0.5 (IQR:0.4-0.6) (p<0.05) and the intensity of pain decreased from a median of 8 (IQR: 7-9.5) to 6.3 (IQR:3.9-7.5) (p<0.05). Eleven patients needed further therapies and were considered non-responders.No serious adverse events were reported.

Conclusion: At one month after GONB, 31% of patients were attacks free and 59% showed a reduction of at least 50% of daily attacks. Our findings confirm that GONB with MP and lidocaine may have an important role as transitional CH management.

## P0156 Phenotype of Cluster Headache: clinical variability, persisting pain between attacks, and comorbidities - a observational cohort study in 825 patients

### C. Göbel^1,2^, S. Karstedt^1,2^, A. Heinze^2^, B. Koch^2^, H. Göbel^2^

#### ^1^University Hospital Schleswig-Holstein, Department of Neurology, Lübeck, Germany; ^2^Kiel Headache and Pain Centre, Kiel, Germany

##### **Correspondence:** C. Göbel

Background and objective: Cluster headaches can occur with considerable clinical variability. The aim of the study was to analyze the severity and extent of the clinical symptoms of episodic and chronic cluster headaches with regard to their variability and to compare them with the requirements of the ICHD-3 diagnostic criteria.

Methods: The study was carried out as a cross-sectional analysis of 825 patients who had been diagnosed with cluster headaches by their physician. Using an online questionnaire, standardized questions on sociodemographic variables, clinical features of the cluster headache according to ICHD-3 and accompanying clinical symptoms were recorded.

Results: The majority of patients with cluster headaches have clinical features that are mapped by the diagnostic criteria of ICHD-3. However there is a significant proportion of clinical phenotypes that are not captured by the ICHD-3 criteria for cluster headaches. In addition, sequential change in the side of the pain, pain location as well as persisting pain between the attacks is not addressed in the ICHD-3 criteria.

Conclusion: The variability of the phenotype of cluster headaches can preclude some patients from receiving an appropriate diagnosis and effective therapy if the diagnostic criteria applied are too strict. The occurrence of persisting pain between attacks should also be diagnostically evaluated due to its high prevalence and severity as well as psychological strain.

## P0157 Patient satisfaction and adverse response from prevention with 240mg galcanezumab of episodic cluster headache

### H. Mo^1^, B. K. Kim^2^, H. S. Moon^3^, S. J. Cho^1^

#### ^1^Hallym University Dongtan Sacred Heart Hospital, Neurology, Hwaseong-si, Gyeonggi-do, South Korea; ^2^Nowon Eulji Medical Center, Eulji University, Neurology, Seoul, South Korea; ^3^Kangbuk Samsung Hospital, Neurology, Seoul, South Korea

##### **Correspondence:** S. J. Cho

Background: A prefilled syringe of a dose of 300 mg of galcanezumab has not been available in most countries including Korea. We investigated the role of two doses of 120 mg of galcanezumab for episodic cluster headache in clinical practices.

Methods: Among 33 patients with episodic cluster headache who received at least one dose of 240 mg of galcanezumab since February 2020 to January 2021. Global impression of improvement and adverse drug responses were collected based on the headache diary or history taking or telephone interviews.

Results: Twenty-eight men and 5 women were enrolled, mean age was 38.4 ± 8.9 years, mean body weight was 72.3 ± 10.8 kg, and 9 patients had comorbid migraine. Twenty-five patients received concomitant preventive medications and 8 patients received only 240 mg of galcanezumab as their preventives. Global impressions of improvement were marked in 17 (51.5%), moderated in 8 (24.2%), mild improvement in 6 (18.1%), and no changed in 2 (6.1%). Among 8 patients treated with galcanezumab only, global impressions of improvement were marked in 5 (62.5%), moderated in 2 (25%), mild improvement in 1 (12.5%), and no changed in 1 (12.5%). There were no serious adverse events.

Conclusion: A dose of 240 mg of galcanezumab can be administered for patients with episodic cluster headache with favorable impression of improvement from patients in daily practices.

## P0158 Association between migraine-related disability and negative thought content, metacognition and emotional distress in adult patients with migraine

### B. Yavuz, E. Acar, B. Sancak, I. E. Sayin, P. Yalınay Dikmen, E. Ilgaz Aydinlar

#### Acibadem University School of Medicine, Istanbul, Turkey

##### **Correspondence:** E. Ilgaz Aydinlar

Objective: This study investigates the relationship between negative cognitive content and the severity of disability associated with migraine in adult populations.

Method: Eighty-one patients, age between 18-65, diagnosed as having migraine according to IHS criteria were asked to fulfill the sociodemographic form, Migraine Disability Assessment Scale (MIDAS), Depression Anxiety Stress Scale (DASS), Automatic Thoughts Questionnaire revised (ATQ-R), and Metacognition Questionnaire-30 (MTQ-30). Bivariate correlations and linear regression analysis were performed to investigate the association between MIDAS, DASS, ATQ-R, and MTQ-30 scores. A probability level of p<0.05 was used to indicate statistical significance.

Results: Pearson correlation analysis yielded positively significant association between MIDAS scores and DASS depression, anxiety, stress subscale scores and ATQ-R scores (r=0.496, p<0.001; r=0.450, p<0.001; r=0.348, p=0.02; r=0.376, p=0.01, respectively). In the linear regression model, DASS depression subscale (p=0.047) and Metacognition Questionnaire-30 postive beliefs about worry dimension (p=0.027) were significant predictors when MIDAS score was a dependent.

Conclusion: Our findings show that higher depression, anxiety, stress levels, and negative thought content are associated with increased migraine-related disability. This study also indicates that depression and positive beliefs about worry predict more migraine-related disability.

## P0159 Dependent-like behaviour and relapse in medication overuse headache one to 17 years after inpatient detoxification

### S. Salhofer-Polanyi^1^, K. Zebenholzer^2^, T. Berndl^2^, K. Kastrati^2^, S. Raab^2^, P. Schweitzer^2^, T. Stria^2^, P. Topic^2^, C. Wöber^2^

#### ^1^Krankenhaus Hietzing mit Neurologischem Zentrum Rosenhügel, Neurology, Vienna, Austria; ^2^Medical University of Vienna, Neurology, Vienna, Austria

##### **Correspondence:** S. Salhofer-Polanyi

Objective: To evaluate whether a dependent-like behavior at follow-up after inpatient detoxification is associated with a relapse into MOH.

Methods: We included MOH patients treated from January 1, 2000 to December 31, 2015. Follow-up information was obtained by a semi structured telephone interview comprising the Beck Depression Inventory (BDI-II), The Beck Anxiety Inventory (BAI), the Migraine Disability Assessment Test (MIDAS), and the Severity of Dependence Scale (SDS). A possible selection bias was excluded by a matched case control analysis between the sample and drop out group.

Results: Ninety out of 493 patients (20.5%) completed the telephone interview. Baseline data of participants and dropouts showed no statistically significant differences. At follow-up dependent-like behavior (i.e. an SDS score >5) was found in 64.8% of the patients and one third experienced primary treatment failure or relapse into MOH. In these non-responders, SDS scores were higher than in patients with sustained absence of MOH (8.5±4.3 vs. 5.7±3.9, p=0.003). Univariate ANOVA showed that improvement was shortest in the group with highest SDS scores, (p= 0.027). SDS, BDI-II, and BAI scores were statistically significantly correlated (r=0.4, p<0.001). Non-responders were significantly more common among dependent patients with than without psychiatric co-morbidity (p=0.05).

Conclusion: After an average of 9.1 years after inpatient detoxification for MOH, dependent-like behavior is present in almost two thirds of the patients. Poor outcome is associated with higher SDS scores and prognosis is worse in patients with dependent-like behavior and comorbid affective disorders.

## P0160 Temporomandibular Disorders in Migraine and Tension-Type Headache Patients: A Systematic Review with Meta-Analysis of Observational Studies

### P. Bizzarri^1^, D. Manfredini^2^, M. Koutris^3^, M. Bartolini^4^, L. Buzzatti^1^, C. Bagnoli^5^, A. Balercia^6^, A. Scafoglieri^1^

#### ^1^Vrije Universiteit Brussel (VUB), Department of Physiotherapy, Human Physiology and Anatomy (KIMA), Faculty of Physical Education & Physiotherapy, Bruxelles, Brussels, Belgium; ^2^University of Siena, School of Dentistry, Siena, Italy; ^3^Academic Centre for Dentistry Amsterdam - ACTA, Dpt of Orofacial Pain and Dysfunction, Amsterdam, Netherlands; ^4^Polytechnic university of marche, Ancona, Italy; ^5^University of Rome Tor Vergata, Department of Clinical Science and Translational Medicine, Rome, Italy; ^6^SOD Chirurgia Maxillofacciale, Ospedali Riuniti Ancona, Ancona, Italy

##### **Correspondence:** P. Bizzarri

Background and objective: Headache is a common painful comorbidity in patients suffering from temporomandibular disorders (TMD). However, the prevalence of TMD in primary headaches patients is not well-defined. The co-occurrence of both primary headaches and TMD can be critical in determining the best clinical management of patients. We aimed to search possible evidence regarding the presence of TMDs in primary headaches patients.

Methods: Observational studies comparing the presence of TMD, arthrogenous, myogenous, or combined, in adults with migraine or tension-type headache (TTH) to ones without headache were included. Two reviewers independently screened articles electronic databases, assessed for risk of bias, and extracted data. Meta-analysis was conducted using a random effect model, Mantel-Haenszel statistical method and Odds Ratio (OR) as effect measure.

Results: 1405 articles were identified. 13 cross sectional studies were finally included. Pulled risk of TMD was higher in TTH and migraine patients than controls (13 studies; OR:4.25[2.84-6.35]), such as risks of myogenous (5 studies;OR:2.01[1.62-2.50]), combined

(5 studies;OR:2.81[1.77-4.46]) and painful

TMD(8 studies;OR:5.31[2.96-9.54]).

Headache patients didn't show risk of arthrogenous TMD

(4 studies;OR:0.96[0.54-1.71]) and non-painful TMD(2 studies;OR:1.10[0.28-4.26]).

Conclusions: Migraine and TTH appear to increase the risk of myogenous, combined or painful TMDs, but not of arthrogenous or non-painful TMD

**Fig. 1 (abstract P0160).**
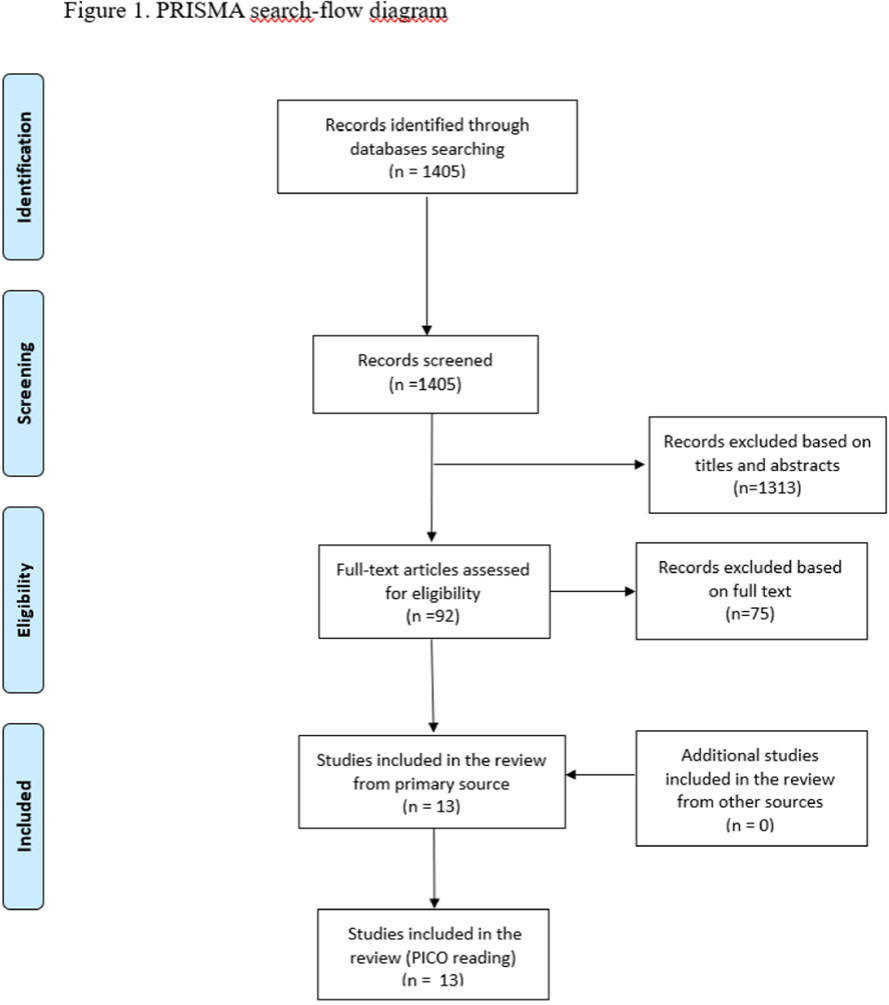
See text for description

**Fig. 2 (abstract P0160).**
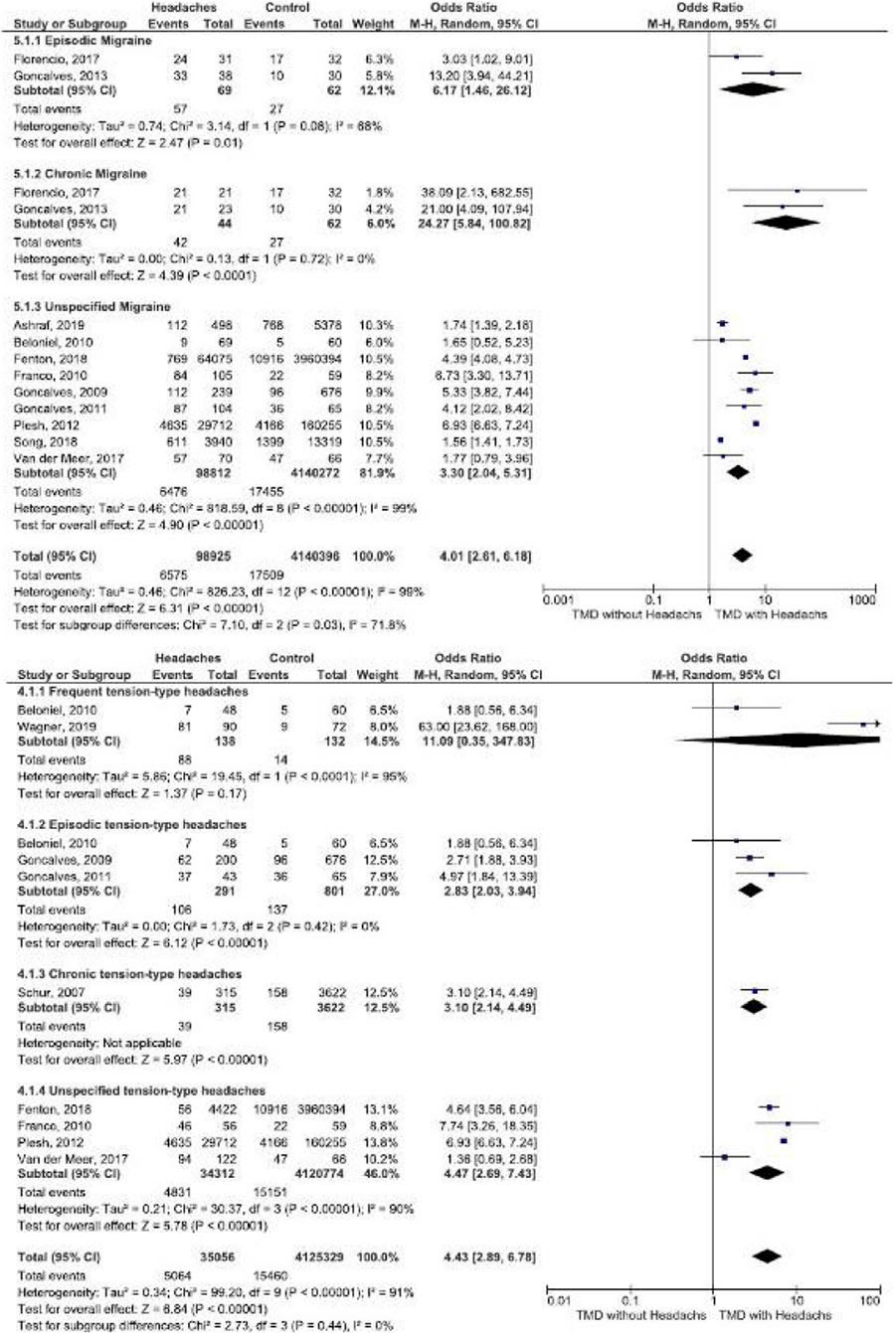
See text for description

## P0161 Fruit and Vegetable Intake, Migraine, and Comorbidities in New Patients at a University Headache Clinic

### A. Narula^1,2^, D. Krashin^3^, M. Chan-Goh^1^, M. Bigal^1^, M. Dyess^1^, N. Murinova^1^

#### ^1^University of Washington, Neurology, Seattle, WA, United States; ^2^University of Washington, Research Volunteer, Neurology, Seattle, WA, United States; ^3^Puget Sound VA, Pain and Psychiatry, Seattle, WA, United States

##### **Correspondence:** N. Murinova

Background: Fruit and vegetable consumption may beneficially influence migraine. This study aims to quantify the amount of servings of fruits and vegetables per week in association with migraine chronification and comorbidities.

Design/Methods: All new patients referred to our headache clinic complete a detailed patient questionnaire. Our intake form asks about fruit and vegetable servings per day, headache characteristics, sleep, depression, and anxiety, headache diagnoses using the ICHD-3 criteria are added.

Results: Questions about fruit and vegetable servings were completed by 4408 patients diagnosed with migraine. 42% of these patients reported eating less than three servings daily. Males have fewer servings of fruits and vegetables per day compared to females. Patients in the upper quintile of consumption have significantly lower PHQ-4 scores and are more likely to exercise. Patients with sleep difficulties consumed significantly fewer servings of fruits and vegetables. There was no significant difference in consumption between episodic and chronic migraine patients.

Conclusions: Close to 80% of migraine patients do not meet minimum recommendations for fruit and vegetable consumption, especially male migraine patients. High consumption correlates with lesser anxiety, and more exercise. We recommend that migraine patients should be counseled about dietary recommendations for fruit and vegetable servings as this may be helpful to them.

## P0162 Psychiatric Conditions in Pregnant Migraine Patients Seen at a University Headache Clinic

### D. Krashin^1^, A. Cuneo^2^, M. Chan-Goh^2^, M. Bigal^2^, N. Murinova^2^

#### ^1^Puget Sound VA, Pain and Psychiatry, Seattle, WA, United States; ^2^University of Washington, Neurology, Seattle, WA, United States

##### **Correspondence:** D. Krashin; N. Murinova

Background: Typically, women with migraine improve during pregnancy. This may not apply to chronic migraine and medication overuse headache (MOH) patients. We wanted to identify if women whose migraine headaches remained severe or worsened during pregnancy were more likely to have psychiatric comorbidity.

Design/Methods: All patients referred to our headache clinic complete a detailed questionnaire prior to their first visit, including questions regarding current pregnancy, headache characteristics, depression and anxiety symptoms and perceived stress. This is analyzed along with headache diagnosis.

Results: 38 patients were pregnant and 37 patients had migraine. Of those, 29 had chronic migraine, and 22 were identified with medication overuse headache. Of these, 17 (44%) had anxiety and 14 (36%) patients had depression, 4 patients had post-traumatic stress disorder and 2 had bipolar disorder. In 31(81%), the patients" headache impaired their work.

Conclusions: Our results show high incidence of psychiatric comorbidities in pregnant women presenting to the headache clinic. ormonal fluctuations in connection with pregnancy can influence attack frequency. Previous studies, including analyses of patient data at our headache clinic, suggest that psychiatric comorbidities are more common in chronic migraine. These patients warrant a greater degree of attention in headache clinic, as they may have more severe health issues while having limited treatment options due to pregnancy.

## P0164 Assessment of the condition neck muscles proprioreception as predictor of migraine chronization

### M. Mozheiko, S. Likhachev, I. Maryenko

#### Republican research and clinical centre of neurology and neurosurgery, Neurological, Minsk, Belarus

##### **Correspondence:** M. Mozheiko

Introduction: Due to the tendency to increase the prevalence of primary headaches, attention is increasing to the problem of chronic migraine (MG), the influence of comorbid factors on its.

Objective: To evaluate effect of proprioception neck muscles according to stabilography data on muscle-fascial function (MFF) in patients with MG.

Materials and methods: 33 patients (w) with MG examined (MGKB-3 beta, 2013), mean age 38±9.4. To assess statokinetic stability (SS) computer stability analyzer used, the "Head turn" test (quality balance function (QBF%), speed change of statokinesiogram area (SCSA mm/s). Assessment of MFF by testing active movements in cervical spine (CS), presence of trigger muscle-fascial zones (MFZ) in the trapezius muscles (TM).

Results: Significant decrease in QBF 91[84.2;94.2]% to 74.3[67.3;71.8]% (T=70;p<0.05) in test "Head turned" right, and significant increase in SCSA 5.4[3.6;12]mm/s to 16[9.08;23]mm/s (T=49;p<0.05) in "Head turned" left. Significantly, restriction of lateroflexia is often noted in testing active movements of CS (χ2=4.36, p=0.03) and trigger MFZ (χ2=5.28,p=0.02) in TM.

Conclusions: Signs of established myofascial dysfunction (MD) detected in 58%, what cause the formation of statokinetic instability in MG patients. To improve tactics of pathogenetic therapy for MG, it"s advisable to use manual-muscle testing in order to identify MD as comorbid factor contributing to the chronization of MG, and to develop drug-free modalities for its correction**.**

## P0165 Relative frequency and subtypes of constipation by migraine status in a healthcare population sample: Results of the Migraine Signature Study

### R. B. Lipton^1^, D. C. Buse^1^, S. Vaidya^2^, A. Scott^2^, A. Jacobson^2^, M. Goodreau^2^, W. Stewart^3^, A. Khodavirdi^4^, M. Navetta^4^, M. Chehrenama^4^, R. Urman^4^, K. Gill^4^, A. Pressman^2^

#### ^1^Albert Einstein College of Medicine, Neurology, Bronx, NY, United States; ^2^Sutter Health, Sacramento, CA, United States; ^3^Medcurio Inc., Oakland, CA, United States; ^4^Amgen Inc, Thousand Oaks, CA, United States

##### **Correspondence:** D. C. Buse

Background and objective: To determine relative frequency and subtypes of constipation among patients with migraine (M) and non-migraine headache controls (HC) in a health system sample.

Methods: A sample of adult (≥18 years of age) primary care patients from a California healthcare system completed a survey which included the validated AMS/AMPP diagnostic migraine questionnaire, common comorbidities, the validated ROME IV Diagnostic Questionnaire (for constipation and subtypes), and a constipation management questionnaire. M and HC groups were identified via survey and HC status was confirmed via EHR.

Results: The overall survey response rate was 1,297/2,558 (50.7%); excluding those with missing data left 807 M and 349 HC patients. Compared with HC patients, M patients were more likely to be female (M: N=654 (81.0%) vs. HC: N=211 (60.5%)), and younger with more in age 30-44 group (M: N=291 (36.1%) vs. HC: N=80 (22.9%)). Respondents with M were more likely than HC to meet ROME IV criteria for constipation (M: N=213 (26.4%) vs. HC: N=31 (8.9%)). Subtype distributions were: Functional Constipation (M=15.0% vs. HC=5.2%), Irritable Bowel Syndrome with Constipation (M=11.4% vs. HC=2.9%) and Opioid Induced Constipation (M=1.2% vs. HC=0.6%).

Conclusions: Constipation overall and by subtypes was more common in patients with migraine than in headache controls, which could be influenced by responder bias. Additional work will explore constipation management and comorbidities.

## P0166 Napping and headache outcomes in adults with episodic migraine: a six-week prospective cohort study in Boston, Massachusetts, USA

### A. Vgontzas^1^, E. Mostofsky^2^, K. Hagan^2^, M. Mittleman^2^, S. Bertisch^3^

#### ^1^Harvard Medical School, Brigham and Women's Hospital, Neurology, Boston, MA, United States; ^2^Harvard University T.H. Chan School of Public Health, Epidemiology, Boston, MA, United States; ^3^Harvard Medical School, Division of Sleep and Circadian Disorders, Department of Medicine, Boston, MA, United States

##### **Correspondence:** A. Vgontzas

Objective: To prospectively examine and quantify the associations of napping with headache frequency, duration, and pain intensity.

Methods: 98 adults with physician-confirmed ICHD-3 episodic migraine reported on headaches and sleep behaviors on twice-daily electronic diaries and wore wrist actigraphs for six weeks. Naps were identified by self-report and confirmed by actigraphy. We used linear regression to examine whether napping was associated with headache outcomes.

Results: Over 4,406 study days, participants reported 1,081 headache days. Over 80% of the sample napped at least once during the study, with naps on 117/1,081 (10.8%) of headache days and 285/3,325 (8.5%) of non-headache days. In age/sex-adjusted models, napping during the study was associated with an additional 1.2 (95%CI -1.1, 3.5) headache days/month. There was no association between napping and average maximum headache pain (1-10 scale) (-0.6 95%CI -7.1, 8.4) or headache duration (0.4 95%CI -5.1, 5.9 hours). Effect estimates were similar after additional adjustment for employment, alcohol and caffeine intake, medication use, disability (HIT-6 score), and sleep quality.

Conclusions: In a primarily employed cohort of patients with episodic migraine in the US, napping was prospectively recorded with a modestly higher prevalence on headache (10.8%) than non-headache days (8.5%). Napping at least once during the study was associated with 1.2 more headache days per month, though estimates were imprecise.

## P0167 Headache in women attending a menopause clinic: an unmet need?

### A. MacGregor, J. Pundir

#### Barts Health NHS Trust, Centre for Reproductive Medicine, London, United Kingdom

##### **Correspondence:** A. MacGregor

Objectives: to assess the prevalence of headache and migraine, headache related disability, and symptomatic migraine treatment in women attending a menopause clinic.

Methods: Women attending the weekly menopause outpatient clinic from October 2019 were asked to complete a simple questionnaire regarding headache and disability. The questionnaire included validated questionnaires to diagnose migraine (ID-migraine to diagnose migraine without aura, and the Visual Rating Scale (VARS) to diagnose migraine aura. HIT-6 was used to assess headache related disability. Patients were also asked to record drugs used for acute treatment.

Results: Data collection was terminated at the end of February 2020 due to the pandemic. Of 117 women completing the questionnaire, 68 reported headache (58%) of which 48 were diagnosed with migraine (41%) and 20 (17%) had non-migraine headache. Of women with migraine, 35/48 had attacks only of migraine without aura and 13/48 had attacks of migraine with aura. Headache associated disability was very severe (HIT-6 60+) or substantial (HIT-6 ≥56≤59) in 51/68 of all women with headache and in 23/48 women with migraine. Of women with migraine, 11/48 treated attacks with triptans, 6/48 took codeine containing medication, and 31/48 used paracetamol only.

Conclusion: Disabling headache affected a substantial number of women and inappropriate treatment was common. There is an unmet need for effective diagnosis and management of migraine in menopause.

## P0168 Headache in COVID‐19: Headache Isolated Symptom

### S. Zamanian, E. Pourakbar

#### Social security organization, Mashhad, Iran

##### **Correspondence:** S. Zamanian

Headache was reported in up to one‐third of the hospitalized patients; yet, the clinical characteristics of headache associated with coronavirus disease 2019 (COVID‐19) have not been defined. This observational case study included patients who were consulted to headache unit due to headache and had COVID‐19 illness. Headache features in 13 PCR‐confirmed COVID‐19 patients with mild symptoms were reported. Headache was the isolated symptom of the COVID‐19 in 3 patients and emerged as an early symptom during the disease course in all patients. Patients specified severe, rapid onset, unrelenting headache with migraine‐like features, as well as unusual sensory symptoms such as anosmia, and gastrointestinal symptoms such as diarrhea and loss of appetite and weight. Headache lasted up to 3 days in 70% of the patients and resolved in all patients within 2 weeks. Despite the fact that most of the patients were female and headache characteristics were suggestive of migraine, majority of patients were not suffering from primary headaches. It was concluded that headache could be an isolated symptom of COVID‐19, which might possibly be ignored in asymptomatic patients. Headaches associated with COVID‐19 included features resembling migraine and/or atypical symptoms including anosmia and diarrhea.

## P0169 Migraine management in a sample of patients during Covid-19 Pandemic in Egypt

### M. Nada, S. Hamdy, M. Abdel-Naseer, A. Hassan, H. Shehata, N. Shalaby

#### Cairo University, Neurology, Cairo, Egypt

##### **Correspondence:** M. Nada

Background: Migraine is a disabling disease with probable risk to be affected during pandemic.

Aim: Explore the effect of Covid-19 pandemic on migraine management in Egypt

Methods: A self-reported questionnaire survey was used for a sample of migraine patients (n=250 patients) in Egypt in the period between May 2020 till January, 2021.

Results: A total of 210 patients (84%) completed this questionnaire survey. The age ranged from 18 to 45 years, with 175 (83.3%) were females. Regarding migraine analysis, 85.7% had episodic migraine (n=180); while 14.2% (n=30) had chronic migraine. 78.57% (n=165) were compliant to prophylactic treatment. Increased migraine frequency was reported in 73.3% (n= 154) while severity increased in 57.2% (n=120) compared to pre-pandemic headaches. Over-use of analgesics was reported in 52.4% (n=110) of respondents. 6 patients were on onabotulinumtoxin A injection but due to lockdown, they didn"t receive their injections with reported increase in their headache frequency and severity. Due to lockdown, telemedicine was initiated in headache clinic but only 28.5% (n= 60) of respondents used it. Seventy of the respondents (33.3 %) had Covid-19 infection during this study with 45 of them (64.3%) reported worsening of their headache severity during and after Covid-19 infection 23 patient reported new headache.

Conclusion: The Covid-19 pandemic has affected both health care provided to migraine patients as well as migraine disease itself.

## P0170 COVID-19 and pediatric headaches: are admissions increasing in Emergency Department?

### A. Marino^1^, C. Galati^1^, C. Gliubizzi^1^, F. Guccione^1^, M. L. Manzo^1^, R. Nardello^1^, S. Mangano^1^, A. Santangelo^2^, F. Brighina^3^, F. D'Aiuto^4^, E. Piro^5^, D. Buffa^6^, V. Raieli^6^

#### ^1^Child Neuropsychiatry Unit Dept. Pro.M.I.S.E. “G D’Alessandro”, University of Palermo, Italy, Palermo, Italy; ^2^Pediatric Clinic, Dept. of Clinical and Experimental Medicine, University of Pisa, Italy, Pisa, Italy; ^3^Dept. of Experimental Biomedicine and Clinical Neurosciences - University of Palermo, Italy, Palermo, Italy; ^4^Emergency Dept.- P.O. Cristina Hospital- ARNAS Civico, Italy, Palermo, Italy; ^5^Neonatal Intensive Care Unit –Dept. for Health Promotion, Maternal Infant Care “G. D’Alessandro”, University of Palermo, Italy, Palermo, Italy; ^6^Child Neuropsychiatry Dept., P.O. Di Cristina - ARNAS Civico Palermo, Italy, Palermo, Italy

#### **Correspondence:** A. Marino

Background and objectives**:** Recent studies have showed that in emergency department (ED) pediatric admissions for headache are increasing in the last years. However Covid-19 pandemic may have changed the use of health services for several reasons. Aim of this study is to analyze the rates of admission for pediatric headaches in ED before and during Covid-19 Pandemic.

Methods**:** we have collected retrospectively the records of children (range of age 5-14) admitted on ED in 2012, 2019 and 2020. We selected the records including Headache and Headache associated to other symptoms (vomit, fever, dizziness, etc.), collecting further the use of computed tomography (CT) and neurological consultation.

Results**:** In 2012, 2019 and 2020 the cephalalgic children admitted to ED were respectively 313/18806 (1.66%), 407/15605 (2.61%) and 234/9630 (2.43%). The admission rates for headaches shows highly significant differences between 2012 and biennial 2019/2020. There are no differences in use of CT and neurological consultations. The only difference in access was the initial drop in the first months of lockdown (2020/80% vs 2019/50%).

Conclusions**:** Our data support the increase of admission for headache to the pediatric ED in the last ten years. However the Covid-19 pandemic has not increased the admission rate compared to 2019 neither the use of CT or neurological consultations. The fear of using EDs was no changed for headache compared to other pediatric alarm symptoms.

## P0171 A Review on Headaches due to covid-19 infection

### S. M. Hashemi^1^, N. Yamani^2^, M. Togha^3^

#### ^1^Tehran University of Medical Sciences, Medicine, Tehran, Iran; ^2^Zanjan University of Medical Sciences, Neurology, Zanjan, Iran; ^3^Tehran University of Medical Sciences, Iranian Center of Neurological Research, Tehran, Iran

##### **Correspondence:** S. M. Hashemi

Since December 2019, numerous review studies have been published on COVID-19 and its neuroinvasion. Although a number of hypotheses have been proposed regarding the association between headache and the coronavirus, no solid evidence has been presented for the mechanism and features of headache in COVID-19. In this review, the headaches reported in previous studies are classified and their possible pathogenic mechanisms are outlined. To accomplish this objective, various types of headache are classified and their patterns are discussed according to ICHD-3 diagnostic criteria, including, headache attributed to systemic viral infection, viral meningitis or encephalitis, non-infectious inflammatory intracranial disease, hypoxia and/or hypercapnia, cranial or cervical vascular disorder, increased cerebrospinal fluid (CSF) pressure, refractive error, external-compression headache, and cough headache. Then, their pathogeneses are categorized into three main categories including, direct trigeminal involvement, vascular invasion, and inflammatory mediators. Furthermore, persistent headache after recovery and the predictors of intensity are further investigated. Apart from the headache in association with Covid-19 infection, there are an increased number of headache sufferers in the Covid-19 era due to the changing of lifestyle and prolonged use of electronic devices. This review offers a practical approach to the classification, diagnosis, and management of COVID-19-attributed headache.

## P0172 Online Mindfulness Improves Emotional Health during COVID-19 for Patients with Migraine, Healthcare Providers, and the General Population

### S. R. Farris^1^, L. Grazzi^2^, M. Holley^1^, A. Dorsett^1^, K. Xing^1^, C. R. Pierce^1^, P. Estave^1^, N. O’Connell^1^, R. E. Wells^1^

#### ^1^Wake Forest Baptist, Winston Salem, NC, United States; ^2^IRCCS Foundation "Carlo Besta" Neurological Institute via Celoria, Milan, Italy

##### **Correspondence:** R. E. Wells

Objective: To evaluation online mindfulness during the COVID-19 pandemic in patients with migraine, healthcare providers, and the general population.

Methods: 233 participants (203 U.S.; 20 international; 10 unknown) participated in this prospective, single-arm, non-randomized clinical trial of a single online mindfulness meditation session with pre- and post-surveys. 45% participants had migraine and 24% were healthcare providers. A web-based review of online resources was also conducted.

Results: Most participants felt the online mindfulness session was helpful and the electronic platform effective for practicing mindfulness (89%, 95% CI: [82 to 93%]), with decreased momentary anxiety (76%; 95% CI: [69 to 83%]), stress (80%; [72 to 86%]), and COVID-19 concern (55%; [46 to 63%]), (p<0.001 for each measure). No differences were seen between groups (patients with migraine or healthcare providers). Participants helped others during the pandemic through 1) following public health guidelines, 2) conducting acts of service and connection, and 3) self-care. "Mindfulness + COVID" search results increased by 52% from May to August 2020 (63.5 to 96.4 Million).

Conclusions: Virtual mindfulness is an increasingly accessible intervention available world-wide that may improve emotional health for patients with migraine, healthcare providers, and the general population during this isolating public health crisis. Kindness and altruism are being demonstrated during the pandemic.

## P0173 Pathophysiology, clinical characteristics and neurological changes in headache in COVID-19

### A. J. Volante^1,2^, M. O. Pelizaro^1,2^, B. B. Bossa^1,2^, A. V. D. Silva^1,2^, V. A. Bello^1,2^, R. C. Frederico^1,2^

#### ^1^PUC PR Londrina, Londrina, Brazil; ^2^University, medicine, Londrina, Brazil

##### **Correspondence:** A. J. Volante; M. O. Pelizaro

Objective: To review neurological manifestations, including headache, associated with a different COVID-19 infection in the literature so far.

Methods: The databases selected for this review were: SciELO (Scientific Electronic Library Online) and PubMed from January 2020 to March 2021. Original observational studies were included.

Results: Nineteen articles with cross- selectional studies, cohort and case report were summarized in Table 1. The main neurological symptoms reported were: headache (8 to 74.6%), dizziness (13%), anosmia and ageusia (33,9 to 68%). Headache was the symptom most observed in the patients in the studies, varying from 8 to 74,6% of the cases. It was reported as pain of moderate to severe intensity, holocranial location, with a focus on the frontal and temporal areas bilaterally, lasting more than 72 hours, predominance in males, associated with anosmia and ageusia.

Conclusion: From this, it is concluded that headache is obtained as the most common neurological manifestation, and may or may not be associated with other symptoms.

Keywords: "headache"; "COVID-19"; "neurological disorders".

**Table 1 (abstract P0173).**
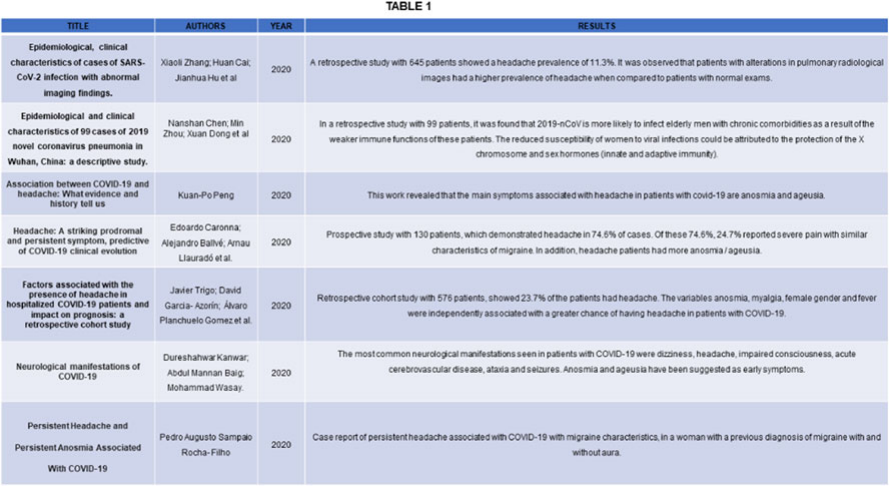
See text for description

**Table 2 (abstract P0173).**
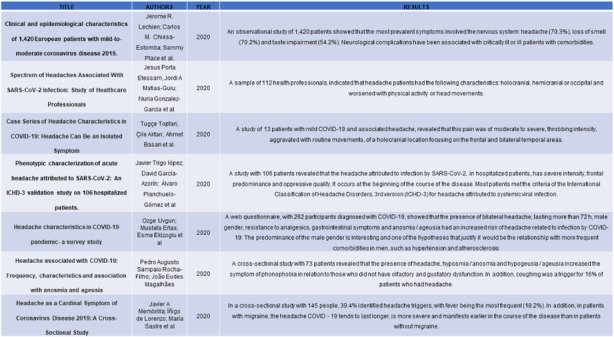
See text for description

## P0175 Evaluation of Amitriptyline as a potential treatment for COVID-19 persistent headache

### A. Gonzalez-Martinez^1^, Á. L. Guerrero Peral^2,3,4^, A. B. Gago Veiga^1^, Á. Sierra Mencía^2^, D. García-Azorín^2,4^

#### ^1^Hospital Universitario de La Princesa & Instituto de Investigación Sanitaria La Princesa, Headache Unit, Neurology, Madrid, Spain; ^2^Hospital Clínico Universitario de Valladolid, Headache Unit, Neurology Department, Valladolid, Spain; ^3^Universidad de Valladolid, Department of Medicine, Valladolid, Spain; ^4^Institute for Biomedical Research (IBSAL), Salamanca, Spain

##### **Correspondence:** A. Gonzalez-Martinez

Objective: Headache is a frequent symptom of COVID-19. Two distinct headache phenotypes have been described in relation with SARS-CoV-2 infection, one associating migraine symptoms and another including tension-type headache (TTH) symptoms. Amitriptyline is a preventive treatment widely employed for both migraine and TTH. We aim to describe COVID-19 persistent headache response to amitriptyline in a series of patients with COVID-19 persistent headache.

Methods: We performed a retrospective cohort study including patients prospectively collected with COVID-19 persistent headache followed-up at two Headache Units and were treated with amitriptyline between March 2020 and October 2020. We gathered the demographic characteristics, COVID-19 headache phenotype as well as amitriptyline response (reduction of 50% in the number of days with headache).

Results: We included 11 patients with COVID-19 persistent headache, 72% (9/11) females, median age 43 (IQR:21) years old. 27% (3/11) had prior diagnosis of migraine and 18% (2/11) had prior history of TTH. TTH COVID-19 phenotype was found in 82% (9/11). Median time follow up was 12 (IQR:6) months. We found that 63% (7/11) improved in either intensity or headache days, all of which presented a TTH COVID-19 phenotype, while none of migraine phenotype did.

Conclusion: Amitriptyline may be an effective preventive treatment for COVID-19 persistent TTH phenotype but not for migraine phenotype, although further larger studies are necessary.

## P0176 Development of patients with migraine in SARS-CoV-2 Pandemic

### A. M. Pochert^1^, S. Morgenstern^1^, G. Goßrau^1^, A. Klimova^2^

#### ^1^Headache Clinic, University Pain Center, University Hospital, TU Dresden, Germany, Dresden, Germany; ^2^Institute of Medical Informatics and Biometrics, Dresden, Germany

##### **Correspondence:** A. M. Pochert

Objective: The SARS-CoV-2 Pandemic resulted since March 2020 in massive restrictions of everyday life. The study investigates quality of life, symptoms of depression, anxiety and stress, headache impact and frequency in patients with migraine in the pandemic.

Methods: In a prospective study, 76 patients with episodic or chronic migraine with and without aura were analysed using patient reported questionnaires: headache diary, HIT- 6 for headache impact on daily life, DASS for depression, anxiety and stress and MSQ v2.1 for migraine-specific quality of life. First data collection was carried out in March 2020 (T0). 3(T1), 6(T2), 9(T3) and 12(T4) months later data was collected again.

Results: We report first results of our ongoing data collection. Overall, headache impact on daily life remained relatively stable over time (HIT-6 median T0:62,T1:61,T2:61,T3: 62). The median DASS score started in March 2020 with 17,5, reduced in June and September to 15 and 13,5 and increased in December to 20 points. Most relevant changes were seen in the depression scale, starting with median 5, decreasing to 4 and 3 in the summer months and increasing in December to 7. The median MSQ was 59.29 at the first survey in March 2020. It increased to 65 after 6 months and 63.57 after 9 months. T4 is actually collected.

Conclusion: Our data show a dynamic development in migraine specific quality of life and self reported symptoms of depression in patients with migraine between March and December 2020.

## P0177 Clinical course of migraine during the COVID-19 Lockdown

### A. Gonzalez-Martinez^1,2^, Á. Planchuelo-Gómez^3^, Á. L. Guerrero Peral^4,5,6^, D. García-Azorín^4,5^, S. Santos-Lasaosa^7^, M. P. Navarro-Pérez^7^, P. Odriozola-González^8^, M. J. Irurtia^8^, S. Quintas^1,2^, R. de Luis-García^3^, A. B. Gago Veiga^1,2^

#### ^1^Hospital Universitario de La Princesa & Instituto de Investigación Sanitaria La Princesa, Headache Unit, Neurology Department, Madrid, Spain; ^2^Universidad Autónoma de Madrid, Departamento de Neurología, Madrid, Spain; ^3^Universidad de Valladolid, Imaging Processing Laboratory, Valladolid, Spain; ^4^Hospital Clínico Universitario de Valladolid, Headache Unit, Neurology Department, Valladolid, Spain; ^5^Institute for Biomedical Research (IBSAL), Salamanca, Spain; ^6^Universidad de Valladolid, Department of Medicine, Valladolid, Spain; ^7^Hospital Clínico Universitario Lozano Blesa & Aragon IIS, Headache Unit, Department of Neurology,, Zaragoza, Spain; ^8^Universidad de Valladolid, Department of Psychology, Valladolid, Spain

##### **Correspondence:** A. Gonzalez-Martinez

Objective: Previous studies have demonstrated that migraine can worsen due to stress, changes in lifestyle habits or infections. We hypothesize that changes during coronavirus disease 2019 (COVID-19) lockdown might have worsened the clinical course of migraine.

Methods: Retrospective survey study collecting demographic data, clinical variables related to headache (frequency), migraine (subjective worsening, frequency, and intensity), lockdown, and symptoms of post-traumatic stress from migraine patients followed-up at three Headache Units between June-July 2020.

Results: 222 subjects were included. Among them, 201/222 (90.5%) were women, aged 42.5 ± 12.0 (mean±SD). Subjective improvement of migraine was reported in 31/222 participants (14.0%), while worsening in 105/222 (47.3%) and was associated with changes in migraine triggers such as stress related to going outdoors and intake of specific foods/drinks. Intensity of attacks increased in 67/222 patients (30.2%), and it was associated with the subjective worsening, female sex, recent insomnia, and use of acute medication during a headache. An increase in monthly days with any headache was observed in 105/222 patients (47.3%) and was related to symptoms of post-traumatic stress, older age and living with five or more people.

Conclusion: Approximately half the migraine patients reported worsening of their usual pain during the lockdown; worsening was related to changes in triggers and the emotional impact of the lockdown.

## P0178 COVID-19 lockdown: a survey on lifestyle changes and migraine

### A. Granato, L. D'Acunto, A. Buoite Stella, G. Furlanis, S. Olivo, P. Manganotti

#### University Hospital and Health Services of Trieste - ASUGI, University of Trieste, Clinical Unit of Neurology, Headache Centre, Department of Medicine, Surgery and Health Sciences, Trieste, Italy

##### **Correspondence:** L. D'Acunto

Objective: COVID-19 lockdown modified lifestyle, behaviours, physical activity (PA) and working habits. Aim of the study is to assess the impact of lockdown on migraine according to behavioural changes.

Methods: Migraineurs who attended the Headache Centre in 2019 were interviewed. All were prophylaxis free or with the same prophylaxis from at least 3 months. Demographics, working routine, lifestyle, migraine characteristics and disability (HIT-6) were compared between the first month of the lockdown and January 2020.

Results: Thirty-seven patients were analysed as migraine without aura (MwoA) (n=26, 45 y [31-53]) and migraine with aura (MwA) plus migraine with and without aura (MwA/MwoA) (n=11, 38 y [26-47]). No changes were reported for food/fluid/alcohol intake, smoke and sleep, while PA decreased (65% vs 31%; p=0.012). Time spent working outside the habitation reduced (MwoA, p=0.001; MwA plus MwA/MwoA, p=0.005) with an increase of remote working (MwoA, p=0.011; MwA plus MwA/MwoA, p=0.039). MwoA reported mean headache duration [3h, (2-12) vs 2h (1-8); p=0.041] and HIT score [59 (51-63) vs 50 (44-57); p=0.001]. MwoA living in urban area had a higher HIT score than those living in rural area [53 (46-57) vs 42 (36-49); p=0.033]. Severity of the attack and symptomatic drug intake didn"t change.

Conclusion: Pain duration and disability improved in MwoA during lockdown, probably due to possibility to rest during attack. Living in rural area might have a protective role.

## P0179 Analysis of headaches urgent care during lockdown due to COVID-19

### J. Espinosa-Rueda, M. P. Navarro-Pérez, S. Ballesta-Martínez

#### HCU Lozano Blesa, Neurology, Zaragoza, Spain

##### **Correspondence:** J. Espinosa-Rueda; M. P. Navarro-Pérez

Objective: Due to the COVID-19 pandemic Spanish government imposed a nationwide lockdown with strict confinement measures from March 15 to May 10, 2020. During this period, there was a decrease in the number of patients who attended the Emergency Department (ED). Our aim is to evaluate the number of patients with primary headaches who visited the ED during lockdown and their management in the ED.

Methods: We retrospectively reviewed patients who visited the ED with diagnosis at discharge of primary headache from 15th March to 10th May 2020 and during the same period of 2019. Demographics, number of admissions, headache duration prior to ED visit, and length of stay in the ED were compared between the two periods.

Results: We found a significant decrease in the number of patients who visited the ED for neurological reasons (396 vs 168) during lockdown in 2020, especially for primary headaches (42 vs 8; p = 0.028) as well as in the length of stay in the ED during the lockdown (198 min vs 444 min; p = 0.002). In addition, headache duration prior to ED visit was longer during the lockdown (245 h vs 119 h), however this difference was not statistically significant (p = 0.114). There were no differences regarding sex, hospital admissions and previous assistance by the Primary Care Physician.

Conclusion: There was a significant decrease in the number of patients attending the ED for primary headache and in the length of stay in the ED during the COVID-19 lockdown.

## P0181 Evaluation of changes in migraine headache patterns and characteristics before and after the pandemic of Covid-19 disease

### M. Togha, F. Martami, E. Jafari, S. Ebadi

#### Tehran University of Medical Sciences, Tehran, Iran

##### **Correspondence:** M. Togha

Objective: We aimed to investigate the impact of COVID-19 pandemic on migraine characteristics.

Methods**:** 400 migraine patients were enrolled in the current cross-sectional study. We administered a self-reported survey that included demographic, migraine-related and lifestyle factors in regard to the period before and after the pandemic of Covid-19.

Results: The frequency, duration and the severity of attacks were found to be significantly higher after the pandemic of Covid-19 than before (10.7 ± 9.9 vs. 9.6 ± 10.4 P= 0.005; 14.3 ± 17.5 vs. 13 ± 15.6 P= 0.001; 7.1 ± 2.1 vs 6.7 ± 2.2 P= 0.001, respectively). After classifying the participants based on the trends of migraine frequency into three groups (decrease, stable and increase) it was found that in the group that the frequency of headache attacks increased, the percentage of patients who using N-95 and N-99 masks was significantly higher than the other two groups. Decreased sleep hours, fast food intake, irregular diet, caffeinated beverage consumption, decreased neck exercise and physical activity and working hours with electronic devices were significantly more reported by patients whose number of headache attacks increased during the pandemic period.

Conclusion: These findings suggest that COVID-19 pandemic had an overall negative impact on migraineurs. Practical strategies should be implemented for patients with migraine, with emphasis on appropriate lifestyle modifications.

## P0182 Case report - Hyposmia after covid-19 in patients with migraine

### B. Höfer, A. Hübler, M. Richter, A. Klimova, A. Hähner, G. Goßrau

#### University Hospital and Faculty of Medicine Carl Gustav Carus TU Dresden, University Pain Centre, Dresden, Germany

##### **Correspondence:** B. Höfer

Background: Headache, loss of smell and taste are among the initial symptoms of Covid-19, and hyposmia often persists even after infection is resolved. We investigate the clinical course of patients with pre-existing migraine after Covid-19 and in particular the response of hyposmia to a structured 3month olfactory training.

Methods: 3patients with pre-existing chronic migraine (cM) and episodic migraine without aura (eM) reported anosmia after infection with SARS-Cov19. We present data on headache frequency and intensity, headache days, days of work disability and headache-related impairment of daily life, as well as olfactory threshold, discrimination and identification, and trigeminal sensitivity. Clinical and history data, headache diary, Midas and the Sniffin Stick Test are collected.

Results: Covid-19 lead to a change in headache type and headache frequency in the patients shown here

**Table:** 

	Aug	Sep	Oct	Nov	Dec	Jan	Feb
cM17	14M, 6H	14M, 16H	10M, 10H	10M,1H	10M, 2H*	14M, 2H	9M,10TTH
cM24	6M,17H	6H	22H*	27H	1M,8H	1M, 1H	2TTH,2H
eM49	4M	2M	2M	4M *	1M	6M	6M

(M=Migraine, TTH=TensionTypeHeadache, H=Headache, *=Covid-19)

*Patients performed oT for 3 months.*

Conclusion: The effects of Covid-19 on patients with migraine have not yet been studied. We report on the course of 3 patients with hyposmia after Covid-19 and the use of a structured olfactory training.

## P0183 Headache prevalence in COVID-19 ambulatory patients

### N. NInashvili^1,2^, M. Shavdia^1^

#### ^1^Tbilisi State Medical University, Tbilisi, Georgia; ^2^Natonal Center for Disease Control and Public Health, Tbilisi, Georgia

##### **Correspondence:** N. NInashvili

COVID-19 is characterized with multiple symptoms. It is well- known that high temperature – one of the leading symptoms of COVID-19 is often accompanied with headache. The aim of the study was to determine prevalence of headache and high temperature in COVID-19 patients being under the online supervision of family doctors.

Methods: Descriptive epidemiological study was conducted in November-December 2020. Patients were selected non-randomly. The study inclusion criteria were a single symptom of respiratory infection and positive PCR test on SARS-CoV-2. Semi-structured questionnaire was developed and disseminated in the study subjects. Descriptive statistics were applied to the results.

Results: Patients age ranged from 10 to 66. The majority of them were females. Although acute pain such as sore throat, back pain, headache, heart pain and muscle pain prevailed the leading symptoms were high temperature(75.0%) and fatigue(62,5%), sore throat ranked third(51.0%) in the complains. Prevalence of headache composed 25.0%. Though headache was not a frequent symptom and rarely accompanied with running temperature, it was severe (by numeric pain scale) and long-lasting in contrast to the other symptoms. Patients suffering with headache were of relatively younger ages (14-21 years old) than those with other types of pain.

Conclusion: Headache was not distinguished with high prevalence in COVID-19 patients, however when existed was severe and long-lasting.

## P0184 Case report: COVID-19 and benign intracranial hypertension Introduction: Neurological complications are not rare in patients who survived COVID-19. On the other hand, ophthalmologists say that ocular manifestations should not be neglected

### I. Zekja, M. Xhelili, J. Kruja

#### Neurology Service, UHC “Mother Teresa” Faculty of Medicine, UM Tirana, Albania, Neurology, Tirana, Albania

##### **Correspondence:** I. Zekja

Case report**:** We report the case of a 45- year-old male patient COVID-19 positive one month ago, without any other comorbidities, who presents in the Emergency Room in a stuporous state and bilateral midriasis after a tonic bilateral epileptic seizure. Two hours later he was lucid and oriented, without any focal neurological deficit but bilateral midriasis persisted. The patient complained severe, holocranial throbbing headache with dizziness, nausea and significant visual blurring. Ophthalmological examination reveals bilateral optic disc oedema, peripapillary hemorrhagic petechiae and venous tortuosity. Brain MRI , Angio- MRI and EEG resulted normal. The patient is treated with a high-dose of corticosteroids for three days and acetazolamide. After treatment he has no other complaints and the headache is less severe. We scheduled a follow-up with fundoscopy, after being treated with acetazolamide for 10 days.

Discussion: Headache is one of the frequent neurological symptoms associated with COVID-19. In the absence of evidence of infectious or vascular disease, pseudotumor cerebri should be considered. Several studies suggest that patients with COVID-19 have vascular retinal lesions ,including flame shaped haemorrhages, peripapillary petechie and acute retinal ischaemia.

Conclusion**:** Further research is needed for COVID-19 and the possible neurological or ocular complications. It is important to consider pseudotumor cerebri in a patient with severe headache after COVID-19 and to perform a fundoscopy if indicated.

## P0185 Headache in COVID-19

### A. Kacprzak

#### Bielański Hospital, Neurology, Warsaw, Poland

Background: Many studies confirmed headache as one of themost common COVID-19-related neurological symptom. Our aim was to recognize and characterize features of headache accompanying this disease.

Methods: Research based on questionnaire study gathered 100 randomly chosen medical healthcare employees who experienced symptoms associated with COVID-19 disease, 96 of them with confirmed COVID-19 (positive SARS-CoV-2 PCR Laboratory Test or positive Rapid COVID-19 Antigen Test). A headache specialist designed a questionnaire containing the main semiological aspects of headache. The questionnaire included questions of headache features such as location (bilateral, unilateral, partial), quality (tension, pulsation), duration of pain (permanent, episodic, duration and frequency of singular headache episodes), associated symptoms like nausea, vomiting, hypersensitivity to light and sounds, headache connection with physical effort. Participants were also asked about information of the COVID-19 not headache-related symptoms, sex and age, presence of fever during disease, headache treatment response and medications they used.

Conclusion: Headaches are one of the most common symptoms of COVID-19. Mostly bilateral, tension type, intensity from middle to severe, sometimes lasting many days. They are often escalating by physical effort or coughing and start frequently at the same time with other COVID-19 symptoms. They do not fulfill migraine and tension-type headache criteria.

**Fig. 1 (abstract P0185).**
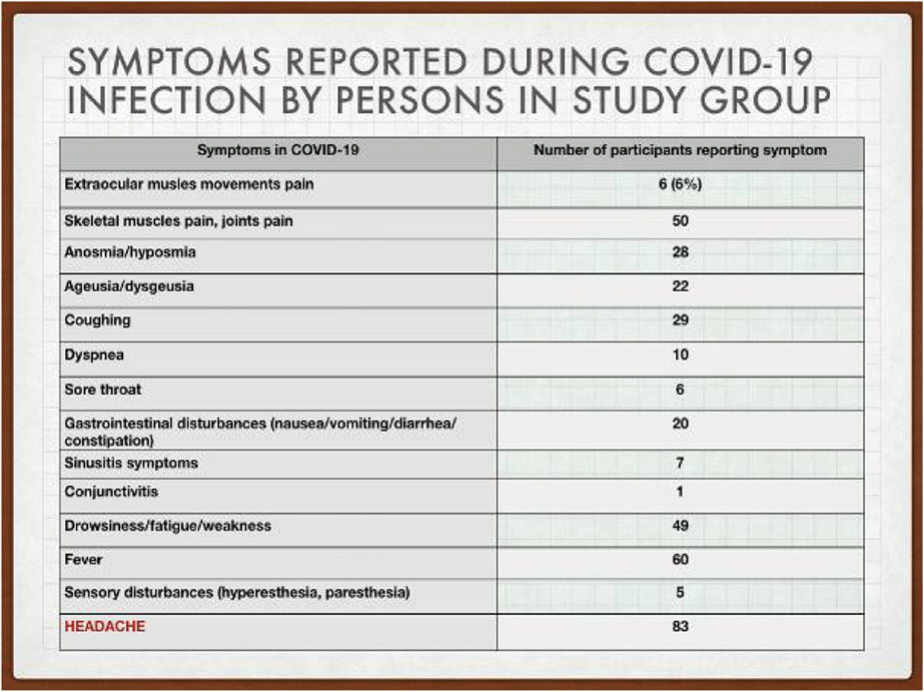
See text for description

**Fig. 2 (abstract P0185).**
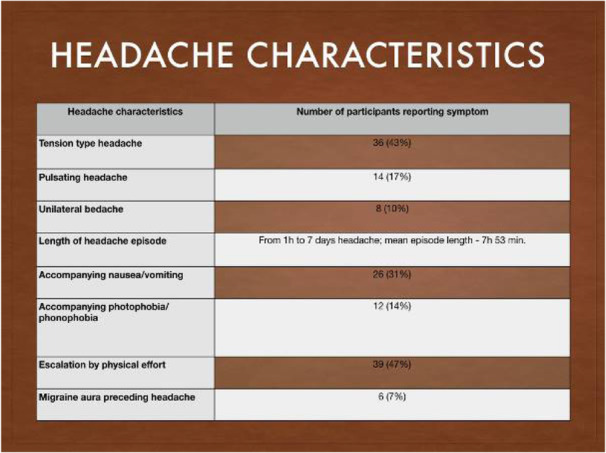
See text for description

## P0186 Impact of COVID-19 in headache disorders: A populational-based study

### M. Peres^1^, T. Lima^2^, A. Oliveira^3,4^

#### ^1^IPq-HCFMUSP, São Paulo, Brazil; ^2^Hospital Albert Einstein, São Paulo, Brazil; ^3^ABRACES, São Paulo, Brazil; ^4^Universidade de Sao Paulo, Center for Clinical and Epidemiological Research, São Paulo, Brazil

##### **Correspondence:** M. Peres

Background: Covid-19 pandemia affected significantly global economy, health care systems and individuals worldwide. Primary headache disorders clinical course may be affected by the social circunstances and the infection itself. Individuals without history of primary headaches may develop headache as symptom of covid-19 infection. Limited information is available regarding the impact of the new coronavirus in Brazil. Objective. We aimed in this study to access the impact of covid-19 pandemia and infection in primary headaches in the general population. Methods. A sample representative of the general population according to the last Brazilian Census through a panel of 1000 respondents was studied. Lifetime prevalence and 1-year prevalence were ascertained. Headache course during pandemia, as covid-19 diagnosis and symptoms were asked. Results. 3.6% of the total population reported they never had a headache before and started to have headaches after the pandemia, 17.7% reported their headache worsened during the pandemia, 74.7% had no change, 2.3% reported headache improvement; 12.5% had confirmed covid-19 diagnosis (5.3% had new headaches), 22% had symptoms without confirmed diagnosis (4.5%). Covid diagnosis or symptoms increased 2.5 times the likelyhood of worsening primary headaches during pandemia (34.8% vs 13.9%). Conclusion. Covid-19 infection and pandemia affected significantly primary headaches patients as increased new headaches in the Brazilian general population.

## P0187 Headache characteristics in COVID-19 pandemic in southern region of Iran

### A. Ghabeli Juibary^1^, A. Sobhani^2^

#### ^1^Shiraz University of Medical sciences, Marvdasht, Iran; ^2^Shiraz University of Medical sciences, Neiriz, Iran

##### **Correspondence:** A. Ghabeli Juibary

Background: Headache is the most important and frequent symptom of COVID-19 patients. We tried to explore this symptom in different settings.

Methods: Our team created paper-based questionnaire screening the characteristics and course of headaches besides clinical COVID-19 features in patients admitted or presented in outpatient clinics. The features COVID-19 related headache and their relations with other clinical features were recorded in the southern region of Iranhospitals/clinics.

Results: A total of 258 COVID-19 participants (150 females;58.13%,) with a mean age of 38.21 ± 10.2 years participated in this study. COVID-19 related headaches were more related with anosmia/ageusia and gastrointestinal disturbances (Nausea/Vomiting abdominal discomfort) (p < 0.000 and p < 0.000),

Headache demonstrated different characteristics like pulsating, pressing. Our analyses showed that bilateral headache, male sex, duration over 3 days, refractory to simple analgesics were significant in COVID-19 positive patients.

Conclusion: long-lasting headaches often bilateral, resistance to simple analgesics and having male gender was more prevalent with COVID-19. gastrointestinal disturbance and anosmia were frequent during headache episodes. The diagnosis of COVID-19 infection may be easier regarding detecting these characteristics in patients.

Keywords: COVID-19 infection; Headache; Migraine.

## P0188 Clinical characteristics of headache after vaccination against COVID-19 (Coronavirus SARS-CoV-2) with the COVID-19 Vaccine BBIBP-CorV: a prospective multicentre observational cohort study

### C. Göbel^1,2^, A. Heinze^2^, S. Karstedt^1,2^, M. Morscheck^2^, L. Tashiro^2^, A. Cirkel^1,2^, Q. Hamid^3^, R. Halwani^3^, M. H. Temsah^4^, M. Ziemann^5^, S. Görg^5^, T. Münte^1^, H. Göbel^2^

#### ^1^University Hospital Schleswig-Holstein, Department of Neurology, Lübeck, Germany; ^2^Kiel Headache and Pain Centre, Kiel, Germany; ^3^University of Sharjah, College of Medicine, Sharjah, United Arab Emirates; ^4^King Saud University, College of Medicine, Riyadh, Saudi Arabia; ^5^University Hospital Schleswig-Holstein, Institute of Transfusion Medicine, Lübeck, Germany

##### **Correspondence:** C. Göbel

Background: The aim of the study is to examine in a real live situation in detail the phenotype of headaches occurring after vaccination against Covid-19 with the Vaccine BBIBP-CorV (Sinopharm).

Methods: The study is a continuous prospective multicenter observational cohort study taking place during the Covid-19 vaccination campaign. With a publicly available online questionnaire, specific aspects of the headache phenotype and related variables are collected. The departments responsible for organizing the vaccinations at university hospitals in Germany and the United Arab Emirates were contacted. They were asked to inform about the study as part of the ongoing vaccination campaign. Furthermore, attention was drawn to the study via the institutes" websites and social media.

Findings: A total of 252 participants reported headaches after vaccination with the COVID-19 Vaccine BBIBP-CorV. The mean age of the participants was 42,5 ± 9,0 years. 84.4% stated that they had not experienced any headaches with any other vaccination. Headaches occur an average of 20,4 ± 29,6 hours after vaccination and last an average duration of 12,3 ± 19,9 hours. In 45.4% of the participants headache occurs as a single episode.

Interpretation: Headaches after Covid-19 vaccination with the COVID-19 Vaccine BBIBP-CorV show a characteristic headache phenotype with relatively mild headache symptoms and with few accompanying symptoms.

## P0189 Microvascular decompression in trigeminal neuralgia - a prospective study of 115 patients

### A. S. S. Andersen^1^, T. B. Heinskou^1^, P. Rochat^2^, J. B. Springborg^2^, N. Noory^1^, E. A. Smilkov^3^, L. Bendtsen^1^, S. Maarbjerg^1^

#### ^1^Danish Headache Center, Department of Neurology, Rigshospitalet - Glostrup, Glostrup, Denmark; ^2^Department of Neurosurgery, Dep. of Neurosurgery, Rigshospitalet - Blegdamsvej, Copenhagen, Denmark; ^3^Department of Diagnostic Radiology, Dep. of Diagnostic Radiology, Rigshospitalet – Glostrup, Glostrup, Denmark

##### **Correspondence:** A. S. S. Andersen

Objective: Primary trigeminal neuralgia (TN) is a neuropathic pain disorder with shock-like touch-evoked pain paroxysms in the trigeminal territory. Microvascular decompression (MVD) is first choice surgical treatment. This is the first high-quality prospective study using independent assessors of outcome and complications of MVD.

Methods: We recorded clinical characteristics, outcome and complications in consecutive patients with TN who underwent MVD. Patients were assessed by a neurologist before and 3, 6, 12 and 24 months after MVD. Neurovascular contact (NVC) was evaluated by 3.0 Tesla MRI with the radiologist blinded to symptomatic side.

Results: We included 115 patients. Ninety-nine (86%) patients had a clinically significant effect, whereof eighty (70%) patients had an excellent outcome. There was a significant association between an excellent surgical outcome and the male sex (4.9 (CI 1.9-12.8), p = 0.001) and NVC with morphological changes (2.5 (CI 1.1 – 6.0), p = 0.036), respectively. Thirty-three (29%) patients had major and 64 (56%) had minor complications. At 24-months follow-up 81 (70%) patients did not have any complications. The most frequent major complication was permanent hearing impairment (10%). The most frequent minor complication was transient hearing impairment (15%).

Conclusions: MVD is effective for TN with a high chance of long-lasting effect. Surgical complications are relatively frequent warranting thorough patient information preoperatively.

## P0190 Effects of two programs with aerobic exercise in headache attributed to temporomandibular disorder

### P. Moleirinho-Alves^1,2^, A. Almeida^2,3^, P. Cebola^2,3^, R. Oliveira^4^, P. Pezarat-Correia^4^

#### ^1^Faculty of Human Kinetics, University of Lisbon, Oeiras, Portugal; ^2^Cuf Tejo Hospital, Physiotherapy, Lisbon, Portugal; ^3^Higher Institute of Health Science, Dental Medicine, Monte da Caparica, Portugal; ^4^Faculty of Human Kinetics, University of Lisbon, CIPER Neuromuscular Research Lab, Oeiras, Portugal

##### **Correspondence:** P. Moleirinho-Alves

Objective: Assess the effects of two 8-week aerobic exercise programs on frequency, intensity, and impact of headaches attributed to temporomandibular disorder (TMD).

Methods: Thirty patients diagnosed with headache attributed to TMD were divided into two groups of 15 participants: an aerobic and therapeutic exercise program (G1, mean age:26.0±4.4 years), and an aerobic exercise program (G2, mean age:24.9±3.4 years). Headache frequency and intensity were evaluated using a headache diary, intensity was reported using a numerical pain rating scale (NRS), and headache impact was evaluated using a Headache Impact Test (HIT-6). These parameters were evaluated twice at baseline (A01/A02), at the end of the 8-week intervention period (A1), and 8–12 weeks after the end of the intervention (A2).

Results: None of G1 participants reported having headaches, and in G2, ten participants still reported headache, at A1. Scores for headache intensity (0.3[95%(0.0 [95%CI:-0.683,0.683]), (2.3[95%CI:1.650,3.017]), significantly decreased in G1/G2 at A1. Score of HIT-6 (37.2[95%CI:33.600,40.733]),(49.3 [95%CI:45.767,52.900]), significantly decreased in G1 at A1. Effects obtained immediately after programs completion were maintained until the final follow-up in both groups.

Conclusion: G1 program had the best results with total relieve of frequency of headache and score decrease of HIT-6 at A1, which remained unchanged at A2. Interventions to reduce headache attributed to TMD should be multimodal.

## P0191 Painful ophthalmoplegia due to involvement of cavernous sinus region by malignant neoplasm: report of three cases

### G. Barros^1^, D. Gulhote^1^, J. Silva^1^, B. Franchito^1^, M. Sukessada^1^, A. Piffer^1^, D. Ueno^2^, P. Neves^2^, H. Mariano da Silva Junior^1,2^

#### ^1^PUC-Campinas, Campinas, Brazil; ^2^Hospital Municipal Dr. Mario Gatti, Neurosurgery, Campinas, Brazil

##### **Correspondence:** G. Barros

Objective: We report the cases to increase the visibility of metastases to the cavernous sinus (CS) resulting in ophthalmoplegia.

Methods: Data disclosure was authorized by the patients through an Informed Consent Form.

Results: A 47-year-old man presented right retro-orbital pain and progressive ophthalmoplegia 5 months after resection of laryngeal spinocellular carcinoma and local radiotherapy. Imaging exam showed involvement of CS (Figure 1). A 44-year-old man, 9 months after excision of spinocellular carcinoma of the larynx and radiotherapy, presented severe pain and paralysis of the left cranial nerve VI. Brain CT was performed (Figure 2). A 67-year-old woman with an adenocarcinoma on the left parotid gland presented a frontal and right temporal headache, more intense in the retro-orbital region. After a month, she developed complete CS syndrome. MRI revealed a T1 hyperintense and T2 hypointense lesion with peripheral enhancement in the CS. All patients died despite treatment.

Conclusion: The most common diagnostic hypotheses to painful ophthalmoplegia are diabetic neuropathy and Tolosa-Hunt syndrome. CS involvement may be the first evidence of a distant head and neck disease. Despite the poor prognosis, palliative care should be considered. Disclosure of Interest: None Declared.

**Fig. 1 (abstract P0191).**
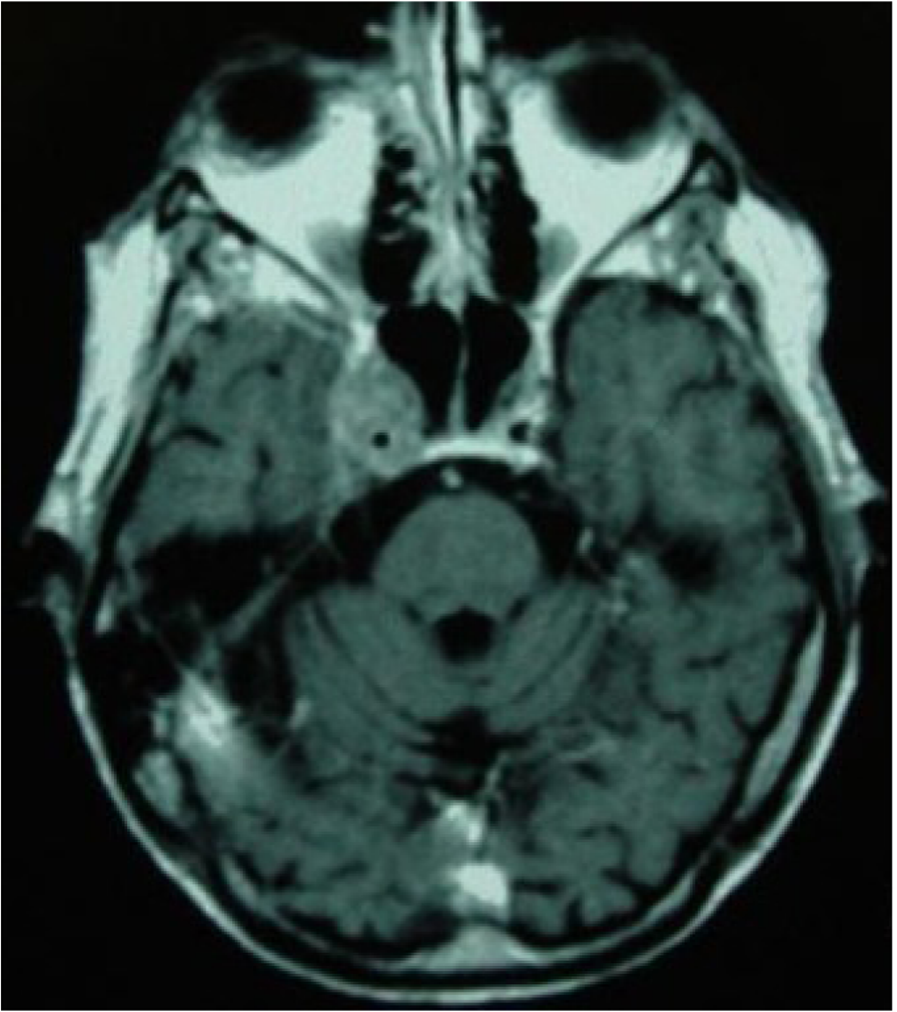
Expansive lesion (2,4x1,7x1,7 cm) in the right CS

**Fig. 2 (abstract P0191).**
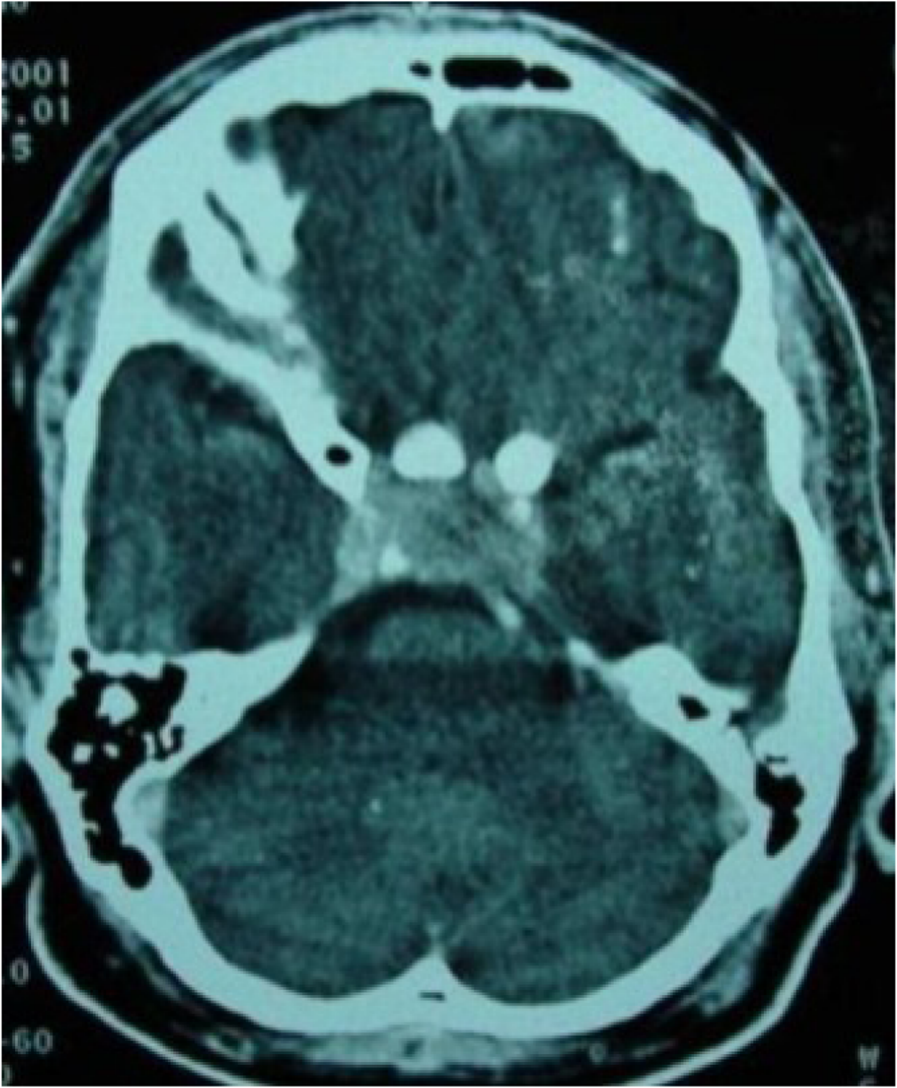
Brain CT showing a hyperdense expansive lesion at the sella turcica's topography

## P0192 Management of trigeminal neuralgia presenting as intraoral pain using Onabotulinum toxin A injections: a case series

### A. Virk, R. Merrill, J. Cohen

#### ^1^UCLA School of Dentistry, Orofacial pain, Los Angeles, CA, United States

##### **Correspondence:** A. Virk

Introduction: Trigeminal neuralgia (TN) presents as tooth or non-tooth pain in the trigeminal distribution. Patients with TN often seek dental care without benefit and risk misdiagnosis and inappropriate treatment. Treatment modalities include antiseizure medications, surgery and Onabotulinum toxin A. We present a series of 7 TN cases presenting as only intraoral pain that were managed successfully with Onabotulinum toxin A injections.

Methods: A comprehensive history and physical exam including dental, head/neck, and neurologic exam was done. Cone beam computed tomography and magnetic resonance imaging were obtained to rule out any secondary causes of pain. Sensory testing revealed areas of intraoral allodynia in each patient. A working diagnosis of trigeminal neuralgia was made per ICHD-3 classification, different treatment options were discussed with patients, and intraoral injections of Onabotulinum toxin A (10-25 units) were done in and around the trigger zones.

Results: Two, 4- and 12-week patient follow-ups found decreased pain and normalized sensory testing. Noted adverse events included temporary drooping of the lip and 1 patient reported a transient increase in pain for 24-48 hours. Most patients reported complete pain relief 10-14 days post injection.

Conclusion: Onabotulinum toxin A can provide extended pain relief without significant adverse effects and provides an additional minimally invasive option for management TN.

## P0193 Sex differences in primary trigeminal neuralgia

### S. Maarbjerg^1^, E. A. Smilkov^2^, N. Noory^1^, T. B. Heinskou^1^, A. S. S. Andersen^1^, J. B. Springborg^3^, P. Rochat^3^, D. M. Kristensen^1,4^, L. Bendtsen^1^

#### ^1^Rigshospitalet-Glostrup, Department of Neurology, Danish Headache Center, Glostrup, Denmark; ^2^Rigshospitalet-Glostrup, Radiology, Glostrup, Denmark; ^3^Rigshospitalet-Glostrup, Neurosurgery, Copenhagen, Denmark; ^4^Université de Rennes, Inserm, EHESP, Irset, Rennes, France

##### **Correspondence:** S. Maarbjerg

Objective: Previous studies have pointed to sex differences in primary trigeminal neuralgia (TN) in various sub-analyses. This is the first study aiming to evaluate sex differences in TN aetiology, demographics, clinical characteristics and medical treatment response.

Methods: We systematically and prospectively collected data in consecutive patients diagnosed with TN by experts in headache and facial pain using semi-structured questionnaires and patient-directed questionnaires. Patients were scanned using 3.0 Tesla MRI. A blinded neuroradiologist evaluated the presence and degree of neurovascular contact.

Results: We included 516 patients with TN out of whom 333 (65%) were women and 183 (35%) were men (p < 0.001). The age at disease onset was 4 years younger in women compared to men (52.9 vs. 57.7 years, p < 0.001). There were no differences in pain characteristics except concomitant persistent pain was more prevalent in women (193 (56%) vs. 87 (48%), p = 0.023). Response to medical treatment and medication dosages at 2-year follow-up was equal. The association (OR) between a neurovascular contact with morphological changes of the trigeminal nerve and the symptomatic side was higher in men (18.5 (6.9-69.7) p < 0.001) compared to women (6.9 (3.5-13.9), p < 0.001).

Conclusions: Based on a unique, large and prospective dataset, we demonstrate that there are significant sex differences in TN pointing to sex-specific distinct TN aetiologies.

## P0194 Radiofrequency thermal ablation as one of the effective methods of trigeminal neuralgia's treatment

### M. Kurnukhina, A. Gusev, V. Cherebillo

#### First Pavlov State Medical University of St. Petersburg, Neurosurgery, St. Petersburg, Russian Federation

##### **Correspondence:** M. Kurnukhina

Background**:** In connection with the progression of the trigeminal neuralgia and the marked resistance to the applied pharmacotherapy, physiotherapy and reflexology, surgical interventions are reasonably used, including minimally invasive puncture techniques of radiofrequency exposure to the peripheral branches of the nerve.

Purpose**:** Evaluation of the effect of radiofrequency thermal ablation on the quality of life of patients with trigeminal neuralgia.

Materials and methods**:** A study of 30 patients with trigeminal branch V3 neuralgia was performed. Against the background of taking medication, the patient noted a side effect, in the form of pronounced dizziness. After the surgical intervention – radiofrequency thermal ablation of the V3 branch of the trigeminal nerve, the intensity of the pain syndrome and the quality of life are monitored using the McGill and SF-36 questionnaires.

Results**:** Positive dynamics in the late postoperative period in the form of a significant reduction in sensory, affective, evaluative scales of the questionnaire by McGiIl(p<0,05), revealed a positive for all scales of the SF-36: physical, role, social and emotional functioning, reduction in pain intensity (p<0,05). We found regression of pain syndrome and absence of complications in the postoperative period in all the studied patients.

Conclusions**:** Radiofrequency thermal ablation of the branches of the trigeminal nerve is one of the most effective methods of treating patients with trigeminal neuralgia.

## P0195 Safety and efficacy of injections of botulinum toxin A for the treatment of trigeminal neuralgia

### O. Tsurkalenko, L. Dzyak

#### State Institution "Dnipropetrovsk medical academy of Ministry of Health of Ukraine", Neurology and Neurosurgery, Dnipro, Ukraine

##### **Correspondence:** O. Tsurkalenko

Background and objective: Pharmacological agents are the first choise for trigeminal neuralgia (TN) treatment. But local intradermal and/or submucosal injections of Botulinum Toxin Type A (BTX-A) could be use in the cases of insufficient effectiveness or intolerance of pharmacological treatment.

Methods: Twenty-five patients with classical TN were treated with adopted local multi-point injection of 150 U of BTX-A. Follow-up visits were conducted every week to observe the pain severity, efficacy and adverse reactions. The primary outcome was the efficacy of BTX-A.

Results: The visual analogue scale scores reduced significantly as early as week 1, and sustained until week 8 throughout the study. Evaluation of the Patient Global Impression of Change demonstrated that 71.6% of the patients reported that their pain symptoms were "much improved" or "very much improved". All adverse reactions were graded as mild or moderate.

Conclusions: BTX-A injection in TN is safe and efficient. It is a useful treatment for refractory TN.

## P0196 A Case of Idiopathic Intracranial Hypertension Presenting with Trigeminal Pain

### A. Janah, S. Markowitz

#### Shaare Zedek Medical Center, Jerusalem, Israel

##### **Correspondence:** A. Janah; S. Markowitz

Introduction: Idiopathic intracranial hypertension (IIH) usually presents with generalized headache, visual obscurations and papilledema.

To our knowledge, this is the first case reported of unilateral trigeminal symptoms related to IIH in a patient with a skull base abnormality.

Case report: A 44-year-old overweight woman presented with intermittent shock like pains and paresthesia on the right side of her face in the distribution of V1-V3.

Neurological exam was normal including fundoscopy and facial sensation.

A contrast enhanced brain MRI showed a hypoplastic Meckel's cave on the right (figure 1), and a small area of hyperintensity and enhancement along the adjacent dura.

MRI was repeated twice and the finding was determined to be venous plexus and not a region of inflammation or a space occupying lesion.

A CT of the brain done to further investigate bony structures of the skull base showed hypoplasia of the right skull base (figure 2), and a partially empty sella -suggestive of raised intracranial pressure.

LP was performed and opening pressure was slightly elevated at 21 cm H2O, there was no pleocytosis or elevated protein.

She had relief of her pain after drainage of CSF which was sustained by treatment with acetazolamide.

Conclusion: We hypothesize that the hypoplastic Meckel"s cave increased the trigeminal nerve susceptibility to irritation from the small elevation in intracranial pressure. Therefore, our patient presented solely with trigeminal pain.

**Fig. 1 (abstract P0196).**
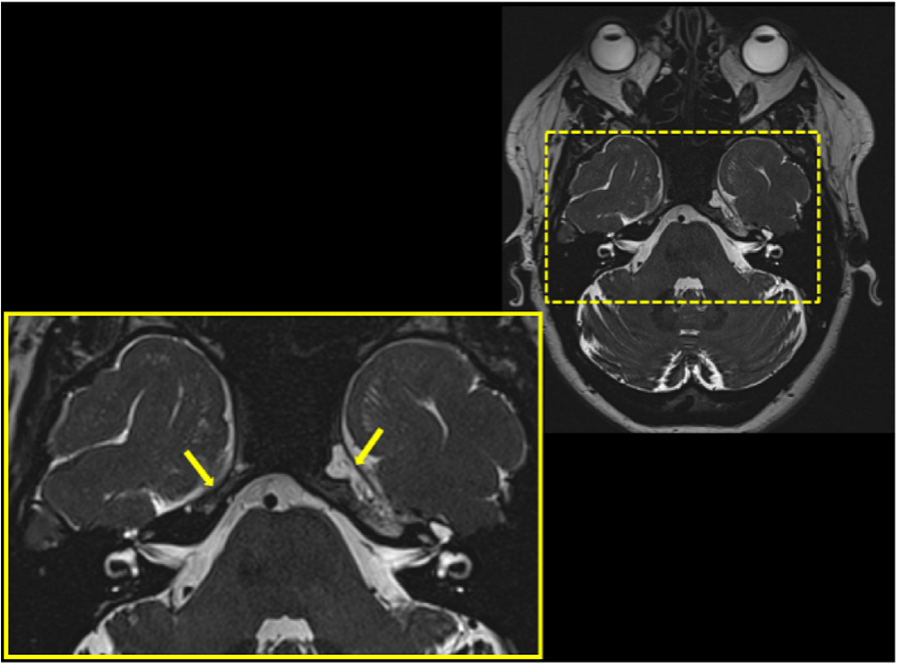
See text for description

**Fig. 2 (abstract P0196).**
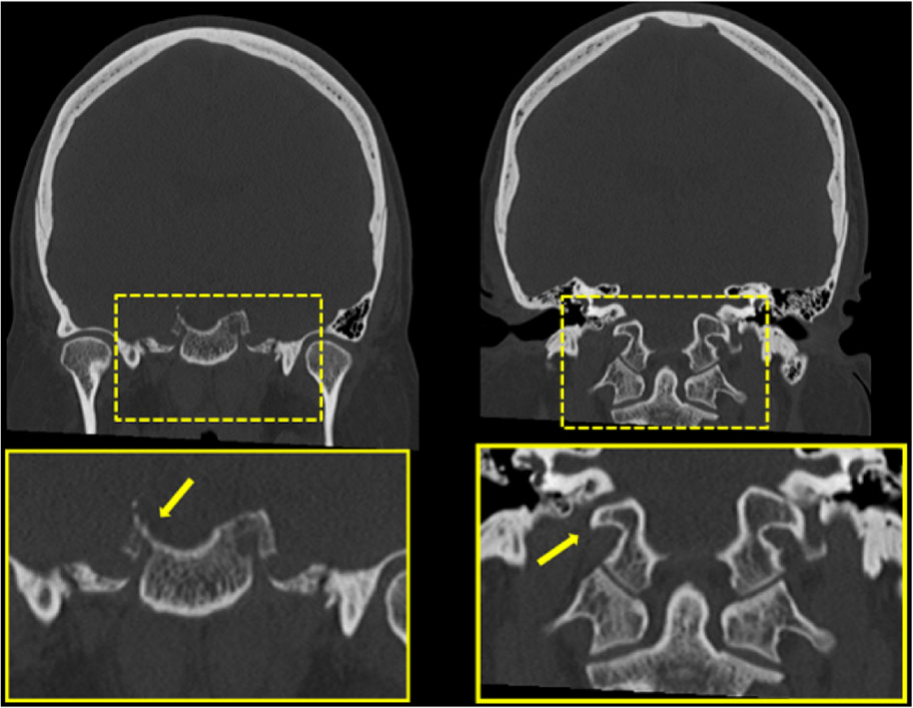
See text for description

## P0198 Migraine premonitory phase prospective study (ProdromaBot study). Preliminary data

### K. Skorobogatykh^1^, J. Azimova^1,2^, D. Korobkova^1^, N. Vashchenko^1^

#### ^1^University Headache Clinic, Moscow, Russian Federation; ^2^The Institute of General Pathology and Pathophysiology., Moscow, Russian Federation

##### **Correspondence:** K. Skorobogatykh

Premonitory phase of migraine attack is hard to study as the symptoms are very unspecific and hard to recall after the attack. The objective of our study was to evaluate the symptoms of the premonitory phase prospectively and to find predictors of the migraine attack. Here we report results of the first part of the study.

Methods**:** We used Migrebot headache diary database to select subjects with migrainosus features and headache frequency 3-8 days per month. After that selected subjects proceeded to complete a specially designed version of the diary (ProdromaBot) to assess the interictal symptoms and migraine attacks for at least 30 days. Participants were required to complete three time points (TP) daily (9am,15pm,21pm). At each TP, participants answered 51 questions about well being, potential triggers, premonitory symptoms, as well as the presence of a headache and its characteristics.

Results**:** 98 subjects entered the study. 71 subjects completed a 30 days period with at least 80% compliance. 59 subjects visited the headache clinic to confirm migraine. From these 59 subjects we collected for further analysis: diary days- 3602; total # of TPs- 10644; total # of answers- 542844; TPs with headache (new or ongoing)- 1783; TPs with episodes of new headache- 960.

Results**:** The design of ProdromaBot diary has proved its reliability for data collection over 30 consecutive days with three time points a day with 51 questions at each TP. Most subjects were at least 80% diary compliant.

**Fig. 1 (abstract P0198).**
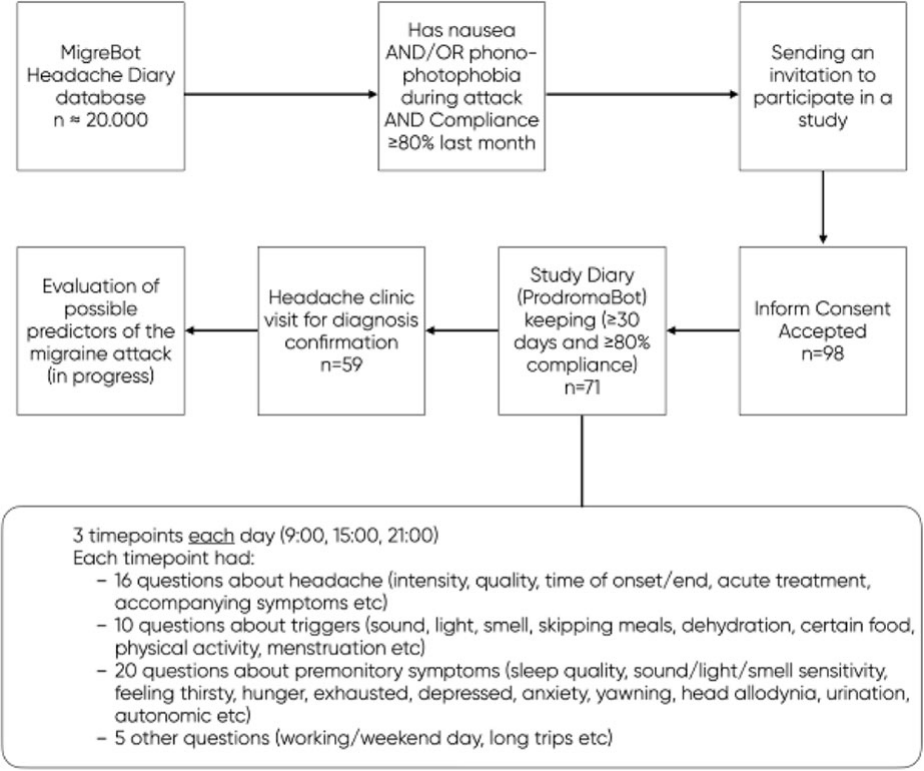
See text for description

## P0200 The *NOTCH3* cysteine-altering variant, p.R544C, does not increase the risk of migraine with or without aura

### Y. F. Wang^1,2,3^, Y. C. Liao^1,2,3^, Y. S. Tzeng^1^, S. P. Chen^1,2,4,3^, J. F. Lirng^2,5^, J. L. Fuh^1,2,3^, W. T. Chen^1,2,3^, K. L. Lai^1,2,3^, Y. C. Lee^1,2,3^, S. J. Wang^1,2,3^

#### ^1^Taipei Veterans General Hospital, Department of Neurology, Taipei City, Taiwan; ^2^National Yang Ming Chiao Tung University, School of Medicine, Taipei City, Taiwan; ^3^National Yang Ming Chiao Tung University, Brain Research Center, Taipei City, Taiwan; ^4^Taipei Veterans General Hospital, Department of Medical Research, Taipei City, Taiwan; ^5^Taipei Veterans General Hospital, Department of Radiology, Taipei City, Taiwan

##### **Correspondence:** Y. F. Wang

Objectives: To determine the prevalence and clinical correlates of *NOTCH3* p.R544C variant, which is associated with cerebral autosomal dominant arteriopathy with subcortical infarcts and leukoencephalopathy, in migraine patients.

Methods: Migraine patients were prospectively enrolled, with the diagnosis made according to the ICHD criteria by headache specialists. DNA samples of 3,502 population controls free of stroke, dementia, and headache were obtained from the Taiwan Biobank. Genotyping of p.R544C was carried out by TaqMan genotyping assay or Axiom Genome-Wide TWB 2.0 Array.

Results**:** The study recruited 2,884 migraine patients (2,279F/605M, mean age 38.8±11.7 years), including 324 (11.2%) with migraine with aura (MA). 32 patients (1.1%) harbored the p.R544C variant, and the percentage was comparable to that in population controls (36/3,502; 1.0%) (p=0.846). Overall, migraine patients with and without p.R544C had similar headache profiles. However, those carrying the p.R544C variant had less pulsatile headache (50.0% vs. 68.2%, p=0.028), and a trend toward a higher percentage of moderate to severe white matter hyperintensities in the anterior temporal lobe (9.1% vs. 0%, p=0.091).

Conclusion: The prevalence of the p.R544C variant was comparable between migraine patients and non-headache controls. The clinical presentations in migraine patients with and without the p.R544C variant were similar. Further studies are needed to clarify the role of *NOTCH3* variants in migraine.

## P0201 Migraine and Tension-Type Headache among Children and Adolescents: Application of International Headache Society Criteria in a Clinical Setting

### J. Genizi, N. Kerem

#### Bani Zion Medical Center, Hafia, Israel

##### **Correspondence:** J. Genizi

Introduction**:** The International Headache Criteria were written in order to help physicians to establishing headache diagnosis. However, sometimes children with headache do not fulfill any diagnosis. The purpose of our study was to assess the clinical application of the criteria in a clinical setting.

Methods**:** Medical records of children referred for primary headache to the pediatric neurology clinic at Bnai Zion Medical Center from 2008 to 2017 were assessed.

Results**:** 989 patients (range 6–18 years; 53% females) were assessed at our neurology clinic. 24% (N=241) were diagnosed with TTH, 26% (N=256) with migraine, 4.5% (45) had mixed headaches and in 41.5% (410) we were unable to reach a specific diagnosis. Patients diagnosed with TTH reported having more emotional difficulties (p = 0.001). No significant differences were found in headache characteristics, frequency or intensity between the younger children and the adolescents within either group, TTH or migraine.

Conclusions**:** Retrospective application of International Headache Society Criteria in a large cohort of children with headaches failed to diagnose a specific type of headache in 41.5% of children. Migraine and TTH were equally prevalent, and both constituted a major burden on our patients' everyday lives. We found no major differences in frequency, intensity, and characteristics of pain between younger children and adolescents.

**Fig. 1 (abstract P0201).**
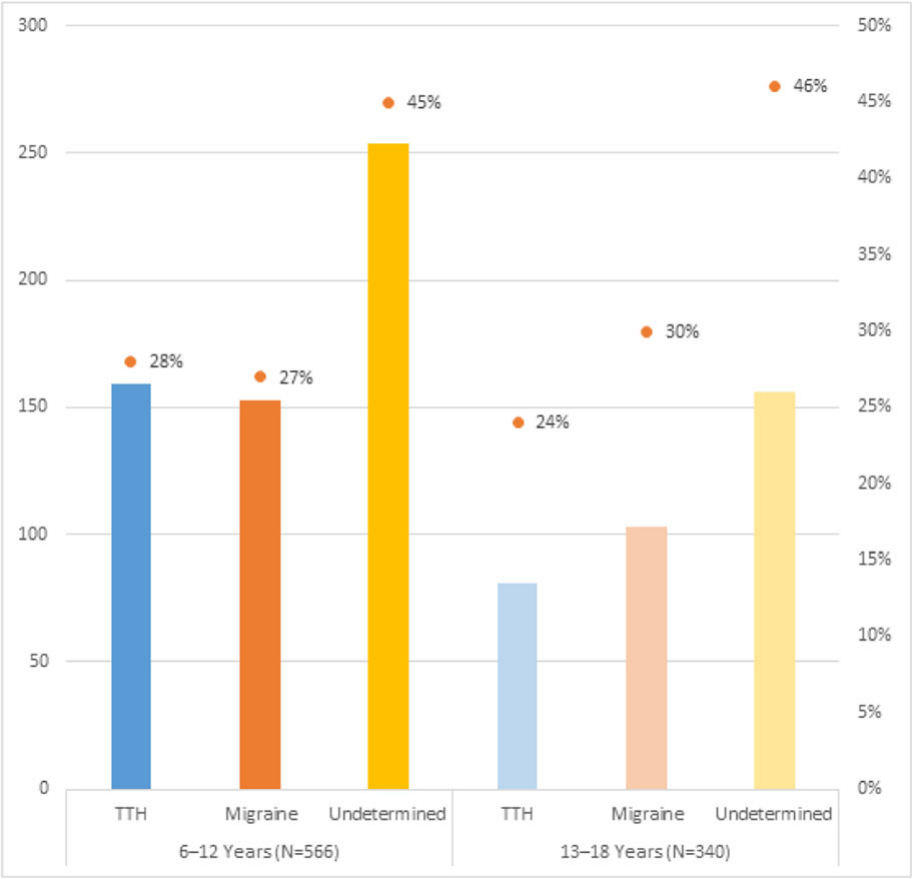
See text for description

## P0202 Systematic review and meta-analysis on physical differences between migraine, cervicogenic headache and healthy controls

### E. Anarte-Lazo^1^, G. F. Carvalho^2^, A. Schwarz^2^, K. Luedtke^2^, D. Falla^1^

#### ^1^School of Sport, Exercise and Rehabilitation Sciences, University of Birmingham, Centre of Precision Rehabilitation for Spinal Pain, Birmingham, United Kingdom; ^2^Institute of Health Sciences, University of Luebeck, Lübeck, Germany

##### **Correspondence:** E. Anarte-Lazo

Objective: Identification of physical differences between patients with migraine and cervicogenic headache (CGH) versus healthy controls (HC)

Methods: A systematic search until January 2020 was performed. The Down"s and Black Scale was used for the risk of bias assessment and the agreement was calculated. A meta-analysis with random effect models was performed, when possible. Two independent reviewers performed all steps of the review.

Results**:** We identified 60 studies and 40 were included in the meta-analysis. Compared to HC, migraineurs exhibited reduced a) range of motion (ROM) on flexion (-2.85, 95%CI -5.12 to -0.58), lateral flexion (-2.17, 95%CI -3.75, -0.59) and the flexion-rotation test (-8.96, 95%CI -13.22 to -4.69), b) cervical lordosis angle (-0.89, 95%CI -1.72 to -0.07), c) pressure pain thresholds (PPT) over the cranio-cervical region, d) neck extension strength (-11.13, 95%CI -16.66 to -5.6), and increased activity of the trapezius (6.18, 95%CI 2.65 to 9.71) and anterior scalene muscles (2.87, 95%CI, 0.81 to 4.94) during performance of the cranio-cervical flexion test (CCFT); besides, CGH patients exhibited decreased neck flexion (-33.70, 95%CI -47.23 to -20.16) and extension (-55.78, 95%CI -77.56 to -34.00) strength.

Conclusion: People with migraine present with a reduction of ROM, strength and PPT, postural changes and altered performance of CCFT when compared to controls but not CGH, while people with CGH present with reduced neck strength compared to HC

## P0203 Differences in physical testing between migraine and cervicogenic headache: A systematic review and meta-analysis

### A. Schwarz^1^, E. Anarte-Lazo^2^, G. F. Carvalho^1^, K. Luedtke^1^, D. Falla^2^

#### ^1^Institute of Health Scienc/ University of Luebeck, Lübeck, Germany; ^2^University of Birmingham, Birmingham, United Kingdom

##### **Correspondence:** A. Schwarz

Objective: Identification of physical differences between patients with migraine and cervicogenic headache (CGH).

Methods: A systematic search of electronic databases published until January 2020 was performed. Down"s and Black Scale was used to assess the risk of bias and the agreement was calculated with Cohen"s Kappa. Data extraction was performed by one reviewer and checked by a second. A narrative synthesis was conducted, data was combined and a meta-analysis with random effect models was performed, when possible. All steps were performed by two independent reviewers, followed the recommendations of the Cochrane Handbook and were reported according to PRISMA. The review was registered at PROSPERO and a study-protocol was published.

Results**:** Seven publications were eligible and six of them were included in the meta-analysis. The results showed decreased range of motion (ROM) on the flexion-rotation test (FRT) (17.67, 95%CI 13.69 to 21.65) and reduced neck flexion strength (23.81 95%CI 8.78 to 38.85) in patients with CGH compared to those with migraine. Further studies, not included in the meta-analysis, suggested an increased percentage of cervical dysfunction, poorer performance on the cranio-cervical flexion test, reduced pressure pain thresholds and increased mechano-sensitivity of neural tissue in patients with CGH compared to migraine patients.

Conclusion: Differential diagnosis of CGH from migraine could be strengthened by use of the FRT and neck flexion strength.

## P0204 Chronic migraine, clinical and genetic considerations

### M. A. Chalmer, J. Olesen, T. F. Hansen

#### Rigshospitalet-Glostrup, Danish Headache Center, Glostrup, Denmark

##### **Correspondence:** M. A. Chalmer

Background & objectives: The transition from episodic migraine (EM) to chronic migraine (CM) is a gradual process, and patients oscillate between EM and CM. The current diagnostic criteria for CM include a mixture of migraine and tension-type-like headaches, suggesting that the current definition of CM is questionable. Patients with migraine on 8 or more days but not 15 days with headache a month are as disabled as patients with ICHD-3 defined CM. Thus, we have suggested new proposed diagnostic criteria for CM (pCM). Patients who meet criteria for migraine with- or without aura ≥ 8 days/month for more than 3 months fulfill the criteria regardless of the frequency of headache. Given the oscillating nature of CM, we assessed if genetic risk factors contribute to migraine chronification.

Methods: We applied whole-genome sequencing and genotype data to assess if common or rare variants give rise to CM or pCM in a cohort of n>2200 migraine patients, clinically assessed by semi-structured interview.

Results: No aggregation of CM nor pCM in families with a clustering of migraine. No rare variants nor a high polygenic risk score give rise to migraine chronification. Migraine chronification is not associated with allelic associations with an odds ratio above 2.65.

Conclusion: No specific genetic variants explain the difference between EM and CM. The present data question the logics of dividing migraine in EM and CM because they most likely are a continuum and not two different states.

## P0205 Exploratory study on the application of digital natural language processing techniques for the classification of headache disorders

### N. Vandenbussche^1,2^, K. Plevoets^3,4^, K. Paemeleire^1,2^

#### ^1^Ghent University Hospital, Department of Neurology, Ghent, Belgium; ^2^Ghent University, Department of Basic and Applied Medical Sciences, Faculty of Medicine and Health Sciences, Ghent, Belgium; ^3^Ghent University, Faculty of Arts and Philosophy, Ghent, Belgium; ^4^Ghent University, Faculty of Sciences, Ghent, Belgium

##### **Correspondence:** N. Vandenbussche

Background and objective: To investigate natural language processing (NLP) to classify between migraine and cluster headache based on patient-written text.

Methods: Outpatient migraine and cluster headache patients were recruited to provide a written, digital, Dutch narrative text about their headache disorder. We analysed texts through manual annotation for themes, lexical and sentiment analysis. Machine learning (ML) models aimed to classify between both disorders based on attack description. The study was approved by the ethical committee of Ghent University Hospital.

Results: One hundred twenty-one patients, 81 migraine patients (79% female, mean age 43) and 40 cluster headache patients (20% female, mean age 49), participated. Themes with the highest coverages were medical history, attack description, treatment and burden of disease. Word keyness in texts was significant for "hoofdpijn" (*headache*) in migraine and "pijn" (*pain*) in cluster headache (both p < 0.001). A trend towards higher negative emotional tonality in attack descriptions by cluster headache patients was found. ML models showed best results for naive Bayes classifiers (average [avg] accuracy [acc] 0.90, avg F1-score 0.85), compared to support vector machines (avg acc 0.80, avg F1-score 0.69) and logistic regression (avg acc 0.81, avg F1-score 0.63).

Conclusions: NLP and ML applications have a high potential to classify between migraine and cluster headache based on patient-written attack descriptions.

## P0206 Explicit Diagnostic Criteria for Transient Ischemic Attacks Used in the Emergency Department Are Highly Sensitive and Specific

### C. Göbel^1,2^, S. Karstedt^1,2^, T. Münte^1^, H. Göbel^2^, S. Wolfrum^3^, E. R. Lebedeva^4^, J. Olesen^5^, G. Royl^1^

#### ^1^University Hospital Schleswig-Holstein, Department of Neurology, Lübeck, Germany; ^2^Kiel Headache and Pain Centre, Kiel, Germany; ^3^University Hospital Schleswig-Holstein, Interdisciplinary Emergency Department, Lübeck, Germany; ^4^Ural State Medical University, Department of Neurology, Yekaterinburg, Russian Federation; ^5^Danish Headache Center, Glostrup Hospital, University of Copenhagen, Copenhagen, Denmark

##### **Correspondence:** C. Göbel

Background: Making a correct diagnosis of a transient ischemic attack (TIA) is prone to errors because numerous TIA mimics exist and there is a shortage of evidence-based diagnostic criteria. In this study, we applied for the first time the recently proposed explicit diagnostic criteria for TIAs (EDCT) to a group of patients presenting to the emergency department of a large German tertiary care hospital with a suspected TIA. The aim was to determine the sensitivity and specificity of the EDCT in its clinical application.

Methods: A total of 128 patients consecutively presenting to the emergency department of the University Hospital of Lübeck, Germany, under the suspicion of a TIA were prospectively interviewed about their clinical symptoms at the time of presentation. The diagnosis resulting from applying the EDCT was compared to the diagnosis made independently by the senior physicians performing the usual diagnostic work-up, allowing calculation of sensitivity and specificity of the EDCT.

Results: EDCT achieved a sensitivity of 96% and a specificity of 88%. When adding the additional criterion F ("the symptoms may not be better explained by another medical or mental disorder"), specificity significantly increased to 98%.

Conclusions: The data show that the EDCT in its modified version are a highly useful tool for clinicians. They display a high sensitivity and specificity to accurately diagnose TIAs in patients referred to the emergency department with a suspected TIA.

## P0207 Familial hemiplegic migraine: a preliminary clinical and follow-up study in a pediatric sample

### M. Capizzi^1^, G. Quatrosi^1^, G. M. Nocera^1^, F. Guccione^1^, A. Santangelo^2^, L. Vetri^3^, R. Nardello^1^, M. Elia^3^, S. Mangano^1^, F. Brighina^4^, G. Santangelo^5^, V. Raieli^5^

#### ^1^Department of Health Promotions Maternal Infant Care, Internal Medicine and Medical Specialities “G. D’Alessandro” Child Neuropsychiatry School, University Hospital “P. Giaccone”, Child Neuropsychiatric Unit, Palermo, Italy; ^2^Pediatric Clinic, Dept. of Clinical and Experimental Medicine, University of Pisa, Italy, Clinical and Experimental Medicine, Pisa, Italy; ^3^Oasi Research Institute-IRCCS., Child Neuropsychiatric Unit, Troina, Italy; ^4^University of Palermo, Department of Experimental Biomedicine and Clinical Neurosciences, Palermo, Italy; ^5^P.O. Di Cristina - ARNAS Civico Palermo, Child Neuropsychiatry Dept, Palermo, Italy

##### **Correspondence:** M. Capizzi

Objective: Familial Hemiplegic Migraine (FHM) is a rare clinical condition. Follow-up studies are even rarer and there is the need to increase the observations of pediatric population affected to clarify the prognosis and possible treatment. Aim of our study was to carry out a follow-up activity in a group of 7 children affected by FHM.

Methods: A multi-center study was conducted retrospectively to select all genetically proven cases of FHM, collecting data based on clinical and genetic documentation. The selected subjects were interviewed on clinical course of hemiplegic migraine, possible other types of headache and clinical disorders.

Results: Our children were 5 males and 2 females (age media onset: 7ys 8m, range age 3,3-15,2; age media follow-up 13 ys 6 m; follow-up duration 5 ys 9 m., range 3ys4m -9ys). We found a CACNA1A mutation in 3 children and a ATP1A2 in 4. At the follow-up time they had complained 1.86 attacks for year. Clinically 57% presented speech disorders, 28,57% sensory disorders, 14,28% visual disorders and 57,14% an impairment of consciousness. Otherwise only 1 child presented a diagnosis of epilepsy and intellectual disability. 3 children showed recurrent attacks of migraine with and without aura.

Conclusions: Our data supports the recent data of other Italian multicentric studies on 14 subjects showing a low frequency of hemiplegic attacks. Further our cases were rarely associated to other disorders and had a good prognosis to short-term follow-up.

## P0208 Coping strategies, psychological symptoms and migraine features in adolescents: which relationship with maternal stress?

### S. Tarantino^1^, M. Proietti Checchi^1^, L. Papetti^1^, M. A. N. Ferilli^1^, F. Ursitti^1^, V. Messina^1^, R. Moavero^1,2^, G. Sforza^1^, F. Vigevano^1^, T. Grimaldi Capitello^1^, M. Valeriani^1,3^

#### ^1^Ospedale Pediatrico Bambino Gesù, Neuroscience, Rome, Italy; ^2^Tor Vergata University of Rome, Child Neurology and Psychiatry Unit, Rome, Italy; ^3^Aalborg University, Center for Sensory-Motor Interaction, Aalborg, Denmark

##### **Correspondence:** S. Tarantino

Objective: We aimed to explore: 1) the coping responses to stressful events and their possible association with migraine severity in adolescents 2) the role of maternal stress on their children’s coping strategies, psychological profile and headache.

Methods: We studied 33 adolescents (m.a. 13.8±1.3 years; 9 M and 24 F). They were divided in “high” and “low” frequency of attacks and “mild” and “severe” pain intensity. To evaluate patients’ anxiety, depression and coping strategies we used respectivelly SAFA-A, SAFA-D and CRI-Y questionnaires. Maternal stress was analyzed by PSI-SF.

Results: We found a significant higher score in “Cognitive avoidance” compared with “Seeking guidance and support” (p= 0.01), “Seeking alternative rewards” (p=0.00) and “Emotional discharge” (p= 0.01) scales. Total SAFA A and D showed a negative correlation with “Positive reappraisal” (respectivelly, p= 0.05 and p= 0.00) and a positive correlation with “Resignation or Acceptance” (p= 0.03 and p= 0.00). A negative correlation between mothers’ PSI Total score and “Positive reappraisal” (p= 0.05) was found. No relationship between CRI-Y and PSI-SF and adolescents’ migraine frequency/intensity was found.

Conclusions: Adolescents with migraine tend to use cognitive strategies of coping, with an avoidance response. Coping response to stressful events and maternal stress show a relationship with adolescents’ psychological symptoms, which in turn, may have a negative influence on migraine severity.

## P0209 Migraine in adolescents: how eating disorders are associated to the frequency of attacks

### S. Tarantino^1^, M. Proietti Checchi^1^, L. Papetti^1^, M. A. N. Ferilli^1^, F. Ursitti^1^, V. Messina^1^, R. Moavero^1,2^, G. Sforza^1^, F. Vigevano^1^, T. Grimaldi Capitello^1^, M. Valeriani^1,3^

#### ^1^Ospedale Pediatrico Bambino Gesù, Neuroscience, Rome, Italy; ^2^Tor Vergata University of Rome, Child Neurology and Psychiatry Unit, Rome, Italy; ^3^Aalborg University, Center for Sensory-Motor Interaction, Aalborg, Denmark

##### **Correspondence:** S. Tarantino

Objective: Data on the possibile association between anxiety, depression, eating disorders and migraine severity in pediatric migraine are sparse. We aimed to analyze: 1) the prevalence of eating disorders symptoms in adolescents with migraine; 2) the possible relationship between anxiety, depression, eating disorders symptoms and migraine frequency.

Methods: We studied 35 adolescent girls with migraine (m.a. 13.9±1.5 years). Due to their low frequencies, we excluded male patients from our analysis. According to the frequency of migraine, patients were classified in “high” and “low” frequency. Anxiety, depression and eating disorders symptoms were assessed by SAFA battery.

Results: Among our patients, 71.5% reported symptoms of anorexic (42.9%) and bulimic (28.6%) behaviour. We found significant higher scores in “School related anxiety” (p= 0.03) and “Perfectionism” (p= 0.01) subscales in patients with high frequency of attacks. In the “high frequency” patients, bulimic symptoms showed a positive and significant correlation with school anxiety (p= 0.03), depressed mood (p= 0.00) and sense of desperation (p= 0.00).

Conclusions: Symptoms of eating disorders may be common among adolescent girls with migraine. Our data suggest that anxiety and depression may mediate the association between bulimic behaviour and migraine frequency. We suppose that school anxiety and depressive symptoms may lead to bulimic behaviour; these symptoms may, in turn, influence the frequency of migraine.

## P0210 Does symptomatic treatment help children and adolescents with chronic migraine?

### M. A. N. Ferilli^1^, L. Papetti^1^, F. Ursitti^1^, R. Moavero^2,1^, S. Tarantino^1^, G. Sforza^1^, F. Vigevano^1^, M. Valeriani^1,3^

#### ^1^Bambino Gesù Children's Hospital, Neurology, Rome, Italy; ^2^Tor Vergata University of Rome, Child Neurology and Psychiatry Unit, Rome, Italy; ^3^Aalborg University, Center for Sensory Motor Interaction, Aalborg, Denmark

##### **Correspondence:** M. A. N. Ferilli

Background and objective**:** Chronic migraine (CM) is defined in the third edition of the International Classification of Headache Disorders (ICHD-3) as the presence of headaches on 15 days or more in a month, at least 8 days showing the migraine phenotype, for more than 3 months. CM affects from 0.6% to 1.8% of children and adolescents and determines a decrease of the quality of life. Aim of this study is to analyze the type of symptomatic drugs used and their efficacy for the treatment of acute migraine attacks in pediatric patients with CM.

Methods**:** We conducted a retrospective and prospective study by selecting pediatric patients diagnosed with CM in our Department. We administered a questionnaire to the parents of all our pediatric patients with CM according to ICHD-3; questions were focused on symptomatic drugs used for acute migraine attacks and their effectiveness.

Results**:** For the final analysis we considered 91 patients with CM. Only two patients responded to the initial therapy with acetaminophen and only 31 % improved with ibuprofen. Fiftythree % of patients had relief with second-line NSAIDs drugs like ketoprofen, indomethacin, naproxen. Fifty one % of patients did not respond to more than three drugs and 16 % were resistant to all acute treatments. All patients underwent prophylaxis therapy.

Conclusions: In our study we have shown that the drugs for acute attack are not very effective in patients with CM and that some patients do not respond to any acute treatment.

## P0211 Vestibular migraine in children: clinical characterization of a cohort

### A. Silva^1,2^, M. Amorim^3^, I. Luzeiro^1^, F. Palavra^2,4,5^

#### ^1^Centro Hospitalar e Universitário de Coimbra, Neurology, Coimbra, Portugal; ^2^Hospital Pediátrico, Centro Hospitalar e Universitário de Coimbra, Child Development Center, Neurology Department, Coimbra, Portugal; ^3^Centro Hospitalar e Universitário de Coimbra, Otorhinolaryngology Department, Coimbra, Portugal; ^4^Instituto de Investigação Clínica e Biomédica de Coimbra (iCBR), Faculdade de Medicina, Universidade de Coimbra, Coimbra, Portugal; ^5^Centro Clínico e Académico de Coimbra, Coimbra, Portugal

##### **Correspondence:** A. Silva

Objective: Characterize demographically and clinically a population of pediatric patients diagnosed with VM

Methods: A retrospective analysis was performed, including patients under 18 years old, observed in our center during 5 years. Electronic records were consulted to obtain demographic and clinical data.

Results: 23 patients were identified, 56.5% being female. The mean age of headache onset was at 10.3 years old (SD=3.6) and vertigo onset at 10.1 years old (SD=4.1). Migraine presented without aura in 87.0% of the cases and was associated with vertigo in 52.2% of the patients. The most frequent vertiginous symptoms were rotation (47.8%), and imbalance (21.7%). In 65.2% vertigo lasted for seconds and occurred once a month (34.8%). Emotional stress was the main trigger for crisis (34.8%), which resolved spontaneously in 47.8% of patients. 69.6% of patients did not need to undergo prophylactic therapy.

Conclusion: In children with vertigo, VM is an etiology to be considered. Clinical history is essential to establish the diagnosis, ascertaining the existence of migraine manifestations, as well as family history. Physical examination allows to exclude vestibular pathology. Treatment consists of preventing crisis initially with non-pharmacological measures and drugs can be started if there is significant impairment in day-to-day activities. Further studies are needed to validate the diagnostic criteria for pediatric VM and to establish the most effective long-term therapy.

## P0212 Pain comorbidity of primary headaches in adolescents in the Republic of Moldova

### T. Lozan^1^, S. Odobescu^2^, I. Moldovanu^2^

#### ^1^ICS Health Forever International SRL, pediatric neurology, Chisinau, Moldova; ^2^Institute of Neurology and Neurosurgery, National Center of Headache, Chisinau, Moldova

##### **Correspondence:** T. Lozan

Background: Pain with various localizations are frequently associated with primary headaches.

Objective: The aim of this study was to evaluate the pain comorbidity of migraine (MG) and tension-type headache (TTH) in adolescents in the Republic of Moldova.

Method: In total there were 1486 adolescents diagnosed with primary headache (10–19 y.o.) recruited from urban and rural area of the country. Diagnosis was based at ICHD-3 (2018) criteria.

Results: Pain comorbidity was more frequently in adolescents diagnosed with MG compared to those with TTH (65.9% vs. 58.3%, p <0.001). Depending on the gender, we highlight a higher intensity of the phenomenon among girls than in boys (66.3% vs 43.8%, p <0.001). In adolescents with MG the prevalence of pain with extracephalic location is higher in girls than in boys (72.8% vs. 52.8%, p <0.001). In the case of TTH boys suffer less often than girls (49.9% vs. 68.6%, p<0.001). Adolescents with MG reported more often abdominal pain (73.1%), unspecified back pain (72.8%) and low back pain (72%). Adolescents with TTH - neck pain (36.6 %), face pain (32%) and chest pain (31.7%).

Conclusion: According to the present study, adolescents suffering from primary headaches reported more frequently abdominal pain (73.1%), low back pain (72%) and cervical pain, with a difference in the degree of their manifestation depending on gender and type of primary headaches.

Key words: migraine, tension-type headache, adolescents, pain comorbidity.

**Fig. 1 (abstract P0212).**
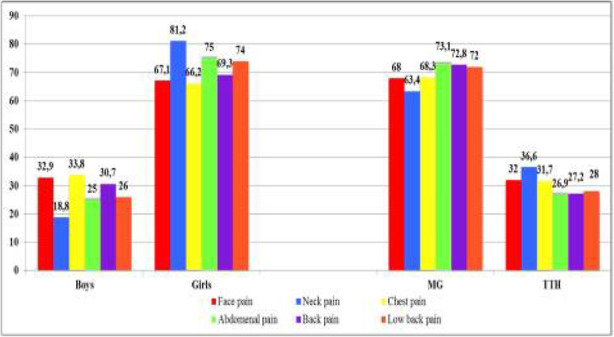
See text for description

## P0213 Prevalence and burden of primary headache disorders among school-aged children in Addis Ababa, Ethiopia

### Y. Zewde, M. Zebenigus, H. Belay

#### Addis Ababa University, Neurology, Addis Ababa, Ethiopia

##### **Correspondence:** Y. Zewde

Background and objective: Headache disorders are the most common pain complaint for seeking medical attention among children. Despite their prevalence, epidemiological data on childhood headache disorders is scarce in sub-Saharan Africa countries, including Ethiopia. Hence, the aim of this study was to assess the prevalence and burden of primary headache disorders among school-aged children in the Ethiopian capital, Addis Ababa.

Methods**:** A cross-sectional survey was conducted among children aged 6-15 years in a private school in Addis Ababa, Ethiopia. Participants were selected by systematic random sampling. A self-administered structured questionnaire used in prior studies was used for data collection. Headache diagnosis was made based ICHD-3 beta version.

Results**:** Of the 359 study participants, 51% were males with a mean age of 10.08 (±2.13) years. The 1-year prevalence of primary headaches was 86.1%: tension-type headache (TTH) (39.1%), migraine (28%), headache on ≥ 15 days/month (1.3%), and probable MOH (1.2%). The overall burden of headache disorders in terms of impaired focus, fear of another attack, and feeling sad were 46.3%. This was significantly higher among migraineurs (29.7% vs 23.8%) as compared to TTH (*X2<0.005).*

Conclusions**:** The prevalence and burden of primary headache were significantly higher among Ethiopian school-aged children. This requires an urgent public health intervention to mitigate the negative effects of headaches on the growing brain.

## P0214 Sleep disorders in Mexican children with headache

### A. Marfil, L. Fernandez, N. Nava, J. De la O, B. Chavez

#### Autonomous University of Nuevo Leon, Neurology Service, Monterrey, Mexico

##### **Correspondence:** A. Marfil; L. Fernandez

Introduction: Migraine is the commonest form of primary headache in pediatric population. Sleep disorders represent a frequently associated comorbidity, observing a complex relationship between these two conditions. The aim is to evaluate the quality of sleep in pediatric patients with a headache disorder.

Methods: A prospective, cross-sectional cohort study was conducted from February-November 2018 at the headache clinic of the Neurology Service of the University Hospital "Dr. José E. González". The clinical history addresses headache characteristics and sleep quality.

Results: Of 52 patients, 65% were female and a mean age of 10.8 yo. 11% of patients had a tension-type headache and 68% had a migraine. Among the latter, 36% were without aura, 10% with aura, and 21% probable migraine. Within sleep disorders, non-restorative sleep was found in 40% of patients and daytime sleepiness in 36%. In addition, 36% of the patients presented snoring and 13% bruxism. In patients with migraine, snoring occurred in 31% and daytime sleepiness in 34%; in patients with tension-type headache, 16% had at least one symptom related to sleep disorders.

Conclusion: In our study, 75% of the patients had at least one symptom of sleep disorder, in agreement with that reported internationally. Non-restorative sleep, daytime sleepiness, and snoring are the most associated sleep disorders. No patient used preventive treatment suggesting a bidirectional causal effect between headache and sleep disorder.

## P0216 The Prognosis of Migraine and Tension-Type Headache in Children and Adolescents

### J. Genizi, N. Kerem

#### Bani Zion Medical Center, Hafia, Israel

##### **Correspondence:** J. Genizi

Backgound**:** Migraine and tension-type headache (TTH) are common among children and adolescents, yet their long-term prognosis is not well understood.

Objective**:** To evaluate the long-term outcomes of pediatric migraine and TTH.

Methods**:** Pediatric patients who visited the pediatric neurology clinic due to diagnoses of migraine or TTH were contacted by phone 8–10 years after their initial diagnosis and interviewed about their outcomes.

Results**:** Of 120 patients, 59 were seen initially due to migraine and 61 due to TTH. For the migraine patients, headaches improved in 48 and worsened in 4. Regarding diagnosis at follow-up, 59% still had migraine, 17% had TTH, and 23% were headache-free. Aura and photophobia were significantly associated with persistence of a migraine diagnosis. For the TTH patients, headaches improved in 49 and worsened in 9. Regarding diagnosis at follow-up, 36.7% still had TTH, 18.3% had migraine, and 45% were headache-free. TTH patients became headache-free at twice the rate of migraine patients. 36.7% of the patients with TTH retained their initial diagnosis compared to 59.3% among the migraine patients.

Conclusions: Most pediatric patients presenting with migraine or TTH will experience a favorable outcome over 10 years, with TTH patients having twice the chance of complete resolution.

**Fig. 1 (abstract P0216).**
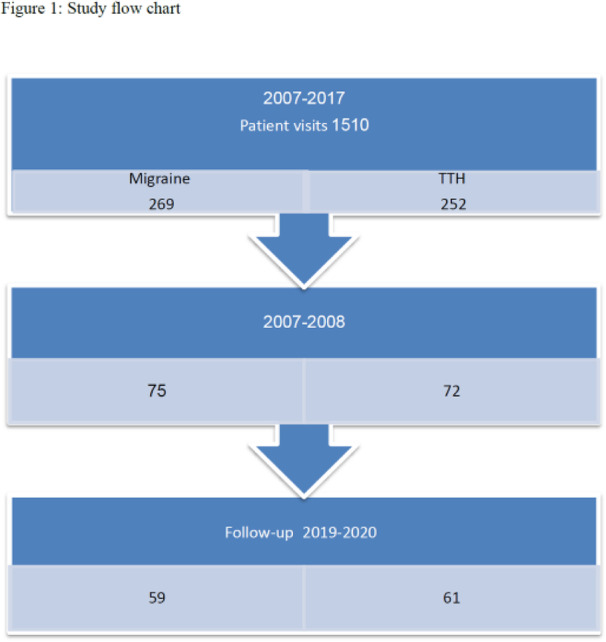
See text for description

## P0217 Odours that trigger migraine attacks and differences in the frequency of migraine attacks induced by odour according to clinical characteristics

### N. Imai^1^, A. Sawai^1^, A. Moriya^1^, E. Kitamura^2^

#### ^1^Japanese Red Cross Shizuoka Hospital, Department of Neurology, Shizuoka, Japan; ^2^Kitasato University, Department of Neurology, Sagamihara, Japan

##### **Correspondence:** N. Imai

Objectives: Our objective was to specifically determine the odours that trigger migraine attacks and the frequency of migraine attacks induced by odour according to clinical characteristics.

Methods: In total, 101 patients were included in our study. A questionnaire was used to determine the types of odour that triggered migraine attacks and the differences in the frequency of migraine attacks induced by specific odours according to their clinical characteristics.

Results: Odours that triggered migraine attacks included the following: perfume (56%), tobacco (48%), fabric softener (33%), body odour (33%), kitchen refuse (25%), hairdressing and hairdresser-related odours (23%), and automobile-related odours (23%). Patients whose migraine attacks were triggered by tobacco, soap or hairdressing and hairdresser-related odours were significantly younger than those whose migraine attacks were not triggered by these odours. Male migraineurs were significantly triggered by moth repellent than female migraineurs. Migraine attacks in patients with chronic migraine were significantly more frequently triggered by excrement, animals, socks, sweat, fabric softener, coffee, soap, kitchen refuse, cheese and vomit than in patients with episodic migraine.

Conclusion: We found that migraine attacks were more frequently triggered by odours among younger patients and patients with chronic migraine, and the triggering odours differed between each group.

## P0218 Adherence and Persistence to Preventive Migraine Treatments over 12 Months Follow-Up for Patients with Migraine: Calcitonin Gene-Related Peptide Monoclonal Antibodies versus Other Preventive Treatments

### O. Varnado^1^, J. Manjelievskaia^2^, J. Ford^1^, W. Ye^1^, A. Perry^2^, K. Schuh^1^, R. Wenzel^1^

#### ^1^Eli Lilly and Company, Indianapolis, IN, United States; ^2^IBM Watson Health, Cambridge, MA, United States

##### **Correspondence:** O. Varnado

Background: Calcitonin gene-related peptide (CGRP) monoclonal antibodies (mAb) were first FDA approved in 2018 for prevention of migraine in adults. Here, adherence and persistence to CGRP mAb versus other preventive migraine treatments (non-CGRP mAb) are compared over 12 months (mo).

Methods: This retrospective, observational study was conducted using MarketScan® Databases. Adults with ≥1 claim (first claim=index) for CGRP mAb (erenumab, fremanezumab, or galcanezumab) or non-CGRP mAb (e.g., antidepressants, anticonvulsants) from 01 May 2018 to 30 Jun 2019 with continuous enrollment for ≥12mo pre- and post-index (follow-up) were included. Adherence was assessed as proportion of days covered (PDC) during 12-mo follow-up. Persistence was defined as days of continuous therapy (gap ≤60 days) from index date to end of follow-up. Descriptive, chi-square (categorical variables), and *t*-test (continuous variables) analyses were conducted.

Results: Overall, 4528 patients (pts) on CGRP mAb and 10,897 pts on non-CGRP mAb were included (Table 1). Mean 12-mo PDC was higher for CGRP mAb versus non-CGRP mAb (55.2% vs 37.8%, P<.001). More pts on CGRP mAb were adherent (PDC ≥80%) versus pts on non-CGRP mAb (P<.001) at 12mo. At end of follow-up, mean persistence was greater for CGRP mAb versus non-CGRP mAb (212.8 vs 142.9 days, P<.001).

Conclusion: At 12-mo follow-up, pts on CGRP mAb had higher medication adherence and persistence compared with pts on non-CGRP mAb.

Sponsor: Eli Lilly and Company.

**Table 1 (abstract P0218).**
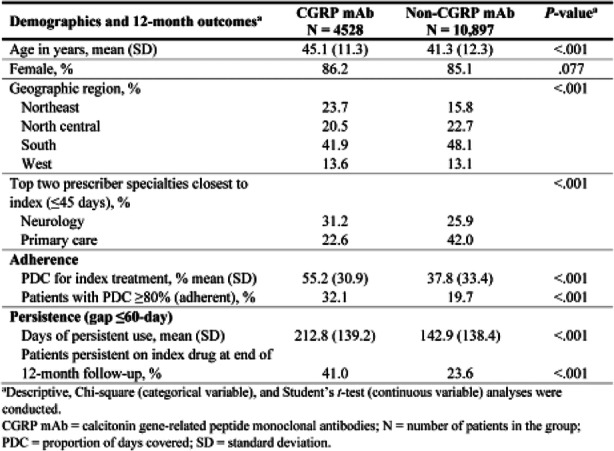
See text for description

## P0219 Treatment Use and Satisfaction in a High Frequency Sample of People Self-Identifying with Migraine: Results of the Coalition for Headache and Migraine Patients (CHAMP) Headache Disease Patient Access Survey

### B. Morton^1^, C. Brooks^2^, K. Lenaburg^1^, M. Buzby^1^, W. Young^3^, R. B. Lipton^4^, D. C. Buse^5^

#### ^1^Coalition for Headache and Migraine Patients, Jericho, VT, United States; ^2^Indiana University, Department of Sociology, Bloomington, IN, United States; ^3^Thomas Jefferson University, Jefferson Headache Center, Philadelphia, PA, United States; ^4^Montefiore Headache Center, Albert Einstein College of Medicine, Department of Neurology, New York, NY, United States; ^5^Albert Einstein College of Medicine, Department of Neurology, New York, NY, United States

##### **Correspondence:** B. Morton

Objectives: To assess treatment use and satisfaction among survey respondents with a self-reported medical diagnosis (SR-MD) of migraine.

Methods: We recruited US adults through CHAMP via email and social media. Treatment satisfaction was measured on a 5-point scale from very dissatisfied (1) to very satisfied (5).

Results: Of 1,770 eligible respondents with a SR-MD of migraine, 92.6% were female, 90.8% white, and 55.5% <50 years old; most (60.8%) reported chronic migraine. For headache, 87.3% had used prescription preventive treatment, 84.8% had used prescription acute treatment, and 55.1% had used ≥10 pharmacological treatments. Nearly all had tried complementary/alternative therapies (96.2%) and 69.7% used biobehavioral treatments. Just 19.1% used devices for headache. Only 37.3% were satisfied with their overall headache treatment plan (Figure). Those seeing headache specialists were most satisfied. Predictors of low satisfaction were higher monthly headache days, more pharmacological treatments tried, and lower levels of education and income. Those employed full time were more satisfied and those "occupationally disabled" less satisfied (Table). Monthly headache days was inversely correlated with satisfaction (*r*_*s*_ = -0.34, *p* < 0.001).

Conclusions: Despite widespread treatment use in this sample with high frequency migraine, satisfaction was low and varied by respondent characteristics; particularly those with more severe and impactful disease.

**Table 1 (abstract P0219).**
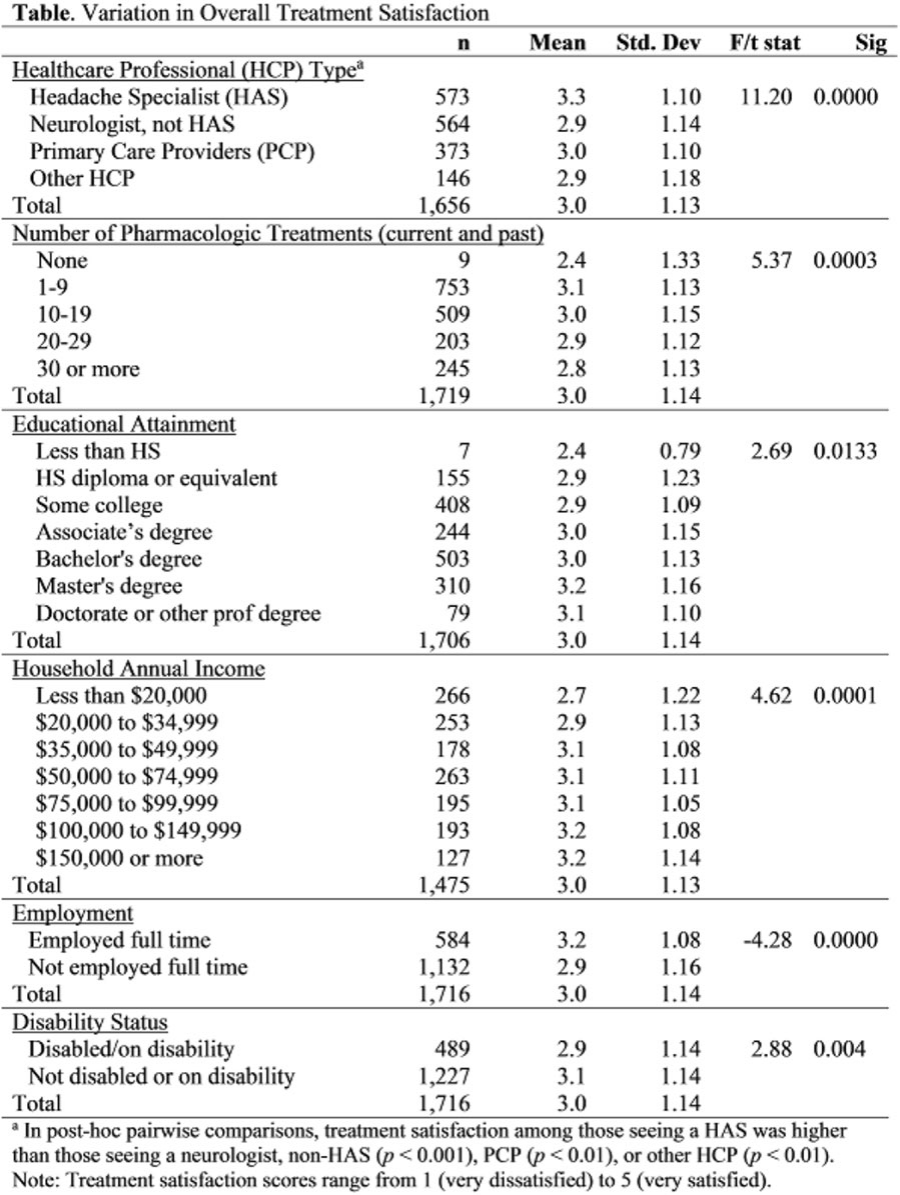
See text for description

**Fig. 1 (abstract P0219).**
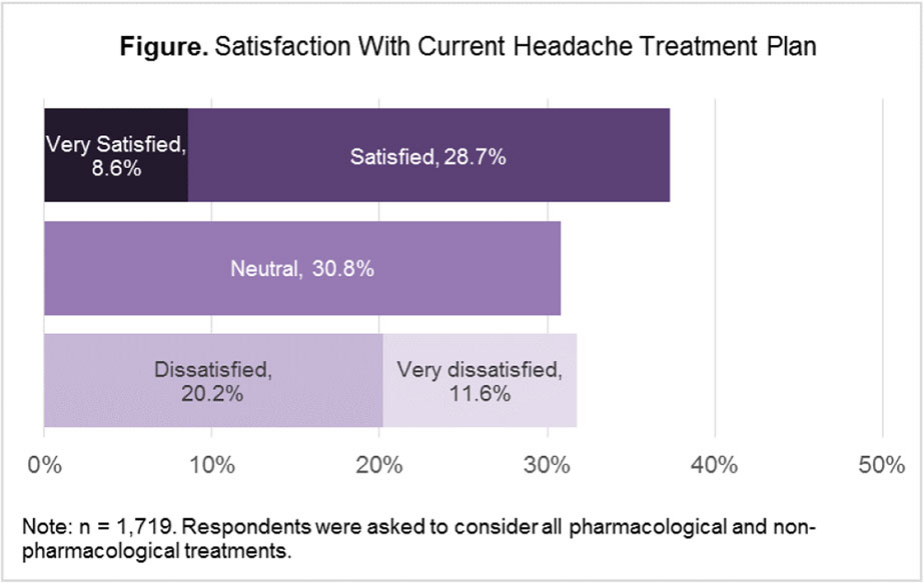
See text for description

## P0220 Impact of migraine on work productivity and healthcare costs in Malaysia: a retrospective, cross-sectional analysis using self-reported data from the Migraine-Buddy© application

### K. J. Goh^1^, S. Kalra^2^, P. Nair Ramadasan^3^

#### ^1^University of Malaya, Department of Medicine, Faculty of Medicine, Kuala Lumpur, Malaysia; ^2^Novartis Corporation, Senior Medical Advisor, Petaling Jaya, Malaysia; ^3^Novartis Corporation, Field Medical Lead, Petaling Jaya, Malaysia

##### **Correspondence:** K. J. Goh

Objective: To evaluate the impact of migraine on work productivity and healthcare costs using the self-reporting Migraine-Buddy© application in Malaysia.

Methods: In this retrospective analysis, the most recent 28-day data captured from adult, self-diagnosed individuals with migraine from registration date on the application (in the 12-month study period; Feb 2020–Jan 2021) were analysed. Patients were stratified by frequency of monthly migraine days (MMD) as episodic migraine (EM; 4–7 MMD), high frequency EM (HFEM; 8–14 MMD) and chronic migraine (CM; ≥15 MMD). Primary endpoints (absenteeism and presenteeism) and secondary endpoints (healthcare cost, demographic characteristics, incidence of anxiety/depression, pain intensity, migraine attack duration) were summarised descriptively.

Results: Of 986 records, 362 patient"s data (EM=348, HFEM=10, CM=4) was analyzed. The mean days of absenteeism and presenteeism/year were 9.8 and 31.7days, respectively, and the mean days increased with increase in MMD (Fig 1). Results of secondary endpoints are presented in Table 1. The burden of healthcare costs for most patients was in the range of RM100–RM299 for physician visits, over-the-counter and alternative medications. About a quarter of patients reported anxiety (23.5%) and depression (26.2%). Majority of the users reported migraine attack duration of 8h–<1day.

Conclusion: Migraine can considerably affect productivity of employed patients and increase the burden of healthcare cost.

**Table 1 (abstract P0220).**
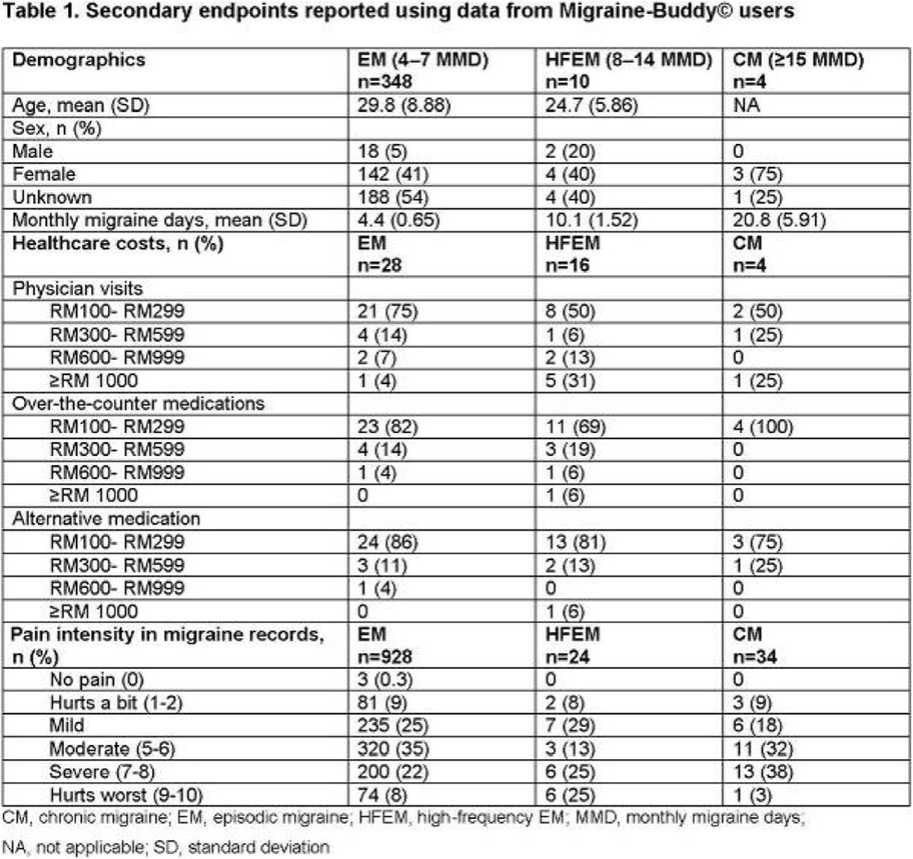
See text for description

**Fig. 1 (abstract P0220).**
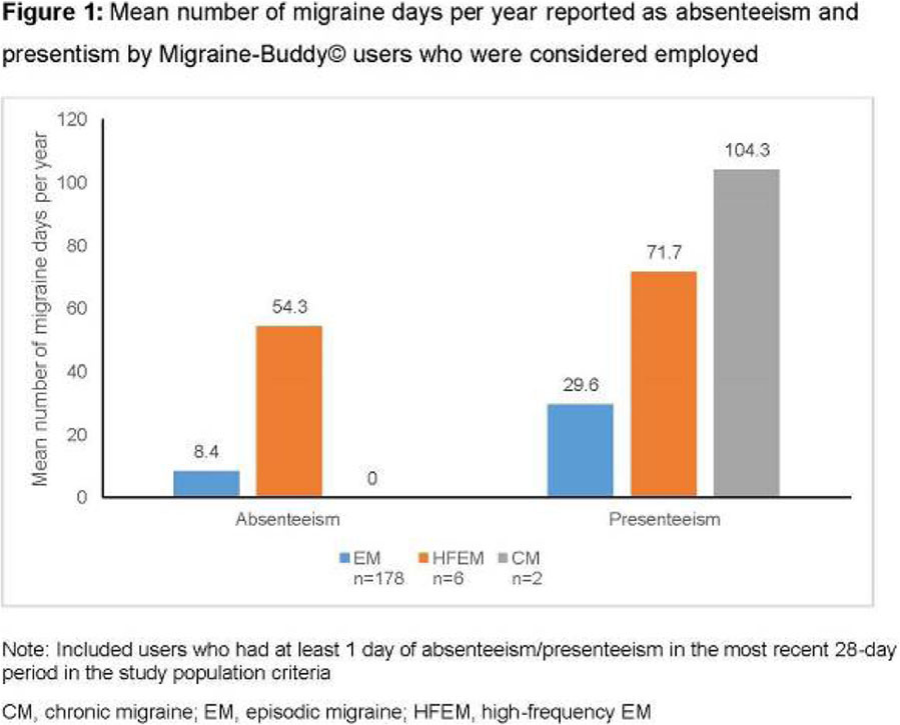
See text for description

## P0221 Migraine evolution over 6 month among Migrebot headache diary users

### K. Skorobogatykh^1^, J. Azimova^1,2^

#### ^1^University Headache Clinic, Moscow, Russian Federation; ^2^The Institute of General Pathology and Pathophysiology., Moscow, Russian Federation

##### **Correspondence:** K. Skorobogatykh

Background: Migraine could be episodic or chronic over a one person lifetime. Migrebot is an interactive chat-based headache diary.

Aim: In this study we analyzed the evolution of migraine frequency among the Migrebot users.

Methods: From more than 20000 Migrebot users we selected those who have had at least one day with migraine. Migraine day was defined as: day with headache with photo- AND phonophobia AND/OR nausea AND/OR any triptan was taken. From these users we selected those, who completed the diary for more than 6 months. We analysed the 1st and the 6th month in these cohorts. We excluded those subjects who completed the diary less than 25 days per month in these months.

Results. We got 1161 subjects and divided them into 4 groups based on the number of headache days during the 1st month. Group A:1-4 days (20,6%); group B:5-8 days (34,1%); group C:9-14 days (29,9%); group D:15-30 days (15,4%).

After 6 month: in group A 76,2% users remained in group A, 19,2% moved to group B, 4,2% moved to groupC, 0,4% moved to group D; in group D 28,5% users remained in group D, 13,4% moved to group A, 24,6% moved to group B, 33,5% moved to group C.

Conclusion: Over 6 month: most of the users with infrequent episodic migraine tend to stay in this group; the majority of users with chronic migraine (71,5%) have improved.

**Table 1 (abstract P0221).**
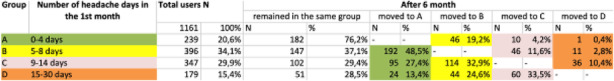
See text for description

## P0222 Online survey revealing patients' journey throughout the Brazilian healthcare system

### M. N. Souza^1,2^, G. Kubota^1^, C. Simioni^1^, M. Calderaro^2^, B. Mattos^3^, L. Chaves^3^, B. Costa^1,2^, F. R. Santos^1,2^, V. Passarelli^1,2^, I. Fortini^1^

#### ^1^University of São Paulo, Neurology / Headache, São Paulo, Brazil; ^2^Hospital Samaritano, Neurology, São Paulo, Brazil; ^3^HSR, São Paulo, Brazil

##### **Correspondence:** M. N. Souza

Objective: investigate the journey of migraine patients.

Methods: cross-sectional web-based survey through social media platforms. We included subjects who reported recurrent headaches and fulfilled ICHD-3 criteria for migraine. We describe clinical aspects and compare those who sought medical attention for their headache with those who did not.

Results: from 541 respondents, 329 fulfilled ICHD-3 criteria for migraine, 261 sought medical attention, and had a higher frequency of headache (11.59 vs 6.89). Use of triptans (29% vs 0, p<0.001), opioids (20% vs 0, p<0.001), and ergotamine (32% vs 11%, p<0.001) were more frequent among patients who sought medical attention. In this group, 25% went only to Emergency Department (ED), 16% only to outpatient care, and 59% sought went to both settings. Regarding acute treatment in the ED, 35% reported significant or complete improvement after 2 hours, 29% mild improvement, and 36% minor or no improvement; 86% reported being informed to have the diagnosis of migraine by the ED physician. Ancillary tests were reported by 57%, 48% reported receiving any information about their condition, and 2% reported recommendation to seek medical follow-up after ED withdrawal.

Conclusion: in this sample, 20% of respondents fulfilling migraine criteria never sought medical help. Among those who sought medical attention we found a high rate of opioid use and ancillary tests, in contrast with a low rate of longitudinal follow-up recommendation.

## P0223 Profile of migraine investigation in the emergency department of a tertiary hospital in Brazil: the exceeding use of Brain CT

### M. N. Souza^1,2^, G. Kubota^1,2^, A. P. Fonseca^2^, M. Calderaro^2^, G. Kuster^2^, M. Jordão^2^, R. Carvalho^2^, A. P. Oliveira^2^, R. Pincerato^2^

#### ^1^University of São Paulo, Neurology / Headache, São Paulo, Brazil; ^2^Hospital Samaritano, Neurology, São Paulo, Brazil

##### **Correspondence:** M. N. Souza

Objective: evaluate the use of brain CT for migraine patients in the Emergency Department (ED).

Methods: a retrospective evaluation of all 814 consecutive Brain CT performed in migraine patients during ED visit with the final diagnosis of migraine, from January 2028 to December 2019. Two independent neuroradiologists evaluated all neuroimaging studies and classified them according to the relevance with the headache diagnosis as no abnormal findings, irrelevant findings, or potentially relevant findings.

Results: among 814 images reviewed, 62,6% were completely normal, 33% presented irrelevant findings, and 4.2% presented potentially relevant findings. Sinusopathy was the most common potentially relevant finding and amounted to 94% of cases. One patient presented an unruptured aneurism, and one patient presented with a meningioma. Unrelevant findings included microangiopathy, cortical volume reduction, arachnoid cists, among others.

Conclusion: our data reveals a low rate of relevant findings among neuroimaging studies performed in patients with migraine diagnosis during ED visits. Considering the recommendation against the use of neuroimaging for patients with migraine diagnosis, and the costs of healthcare resource utilization with no substantial value to patients, our data points to the need to improve decision making for clinicians evaluating migraine patients in the ED.

## P0224 Educational Level of Migraine Patients and Clinical Features Seen at a University Headache Clinic

### N. Murinova^1^, M. Dyess^1^, M. Chan-Goh^1^, M. Bigal^1^, A. Cuneo^1^, D. Krashin^2^

#### ^1^University of Washington, Neurology, Seattle, WA, United States; ^2^Puget Sound VA, Pain and Psychiatry, Seattle, WA, United States

##### **Correspondence:** N. Murinova

Background: Education level effects migraine, lower education correlates with risk of migraine chronification. This study aims to quantify the level of education in patients referred to our headache clinic, evaluate whether lassociated with an increased risk of chronification, and relates to other common migraine comorbidities.

Method: New patients referred to our headache clinic complete a patient intake questionnaire that asks about the highest level of education completed, as well as about headache characteristics, sleep, depression, anxiety, and stress. Data was analyzed with patients' diagnoses.

Results: In our analysis, 4408 unique patients were diagnosed with migraine, 75% with chronic migraine. 5.72% had a doctorate or higher, 16.88% had a masters, 39.58% completed at least college, 34.32% completed at least high school. 63% of patients seen in the clinic have college or higher education. Statistical analysis shows that having a higher education correlates with less chronification. This effect is robust when correcting for gender and age.

Conclusions: It is notable that most patients seen in our headache clinic have college or higher education and yet suffer from chronic migraine. However, we show that patients with college or higher education have less headache days per month and less severe headaches compared to patients with less than college education. Educational attainment may be protective due to greater functional brain reserve.

## P0225 Twenty-five years of Triptans - A Nationwide Population Study

### O. Davidsson^1^, I. Olofson^2^, L. J. A. Kogelman^2^, M. Andersen^1^, K. Rostgaard^1^, H. Hjalgrim^1^, J. Olesen^2^, T. Hansen^2^

#### ^1^Statens Serum Institut, Copenhagen, Denmark; ^2^Danish Headache Center, Glostrup, Denmark

##### **Correspondence:** O. Davidsson

Background**:** The efficacy of triptans as the main acute treatment strategy for migraine headache at the population wide level needs to be understood to inform clinical decision-making. We summarize key trends in triptan use using more than 25 years of Danish nationwide data.

Methods**:** We conduct a nationwide register-based cohort study based on all Danish residents with access to public healthcare between Jan 1st, 1994 and Oct. 31st, 2019 and summarize informative trends of all purchases of triptans in Denmark in the same period.

Results**:** Over a 25-year period, triptan use almost tripled and the yearly prevalence of triptan use increased from 5.17 to 14.57 per 1,000 inhabitants. Between 2014 and 2019, 12.3% of the Danish migraine population purchased a triptan. After an initial purchase, 43% of patients had not repurchased triptans within 5 years. At most 10% of patients indicating triptan discontinuation tried more than one triptan. The prevalence of triptan overuse increased in parallel with the prevalence of triptan use, prevalent in 56 of every 1,000 triptan users every year between 2014 and 2019.

Conclusion**:** In a cohort with access to free clinical consultations and low medication costs, we observed low rates of triptan adherence, likely due to disappointing efficacy and/or unpleasant side effects rather than economic considerations. Triptan success continues to be hindered by poor implementation of clinical guidelines and high rates of treatment discontinuance.

## P0226 The Migraine-Specific Quality of Life Questionnaire, Role Function Restrictive Domain: Defining Clinically Meaningful Categories of Functional Impairment Severity

### R. Speck^1^, D. Kudrow^2^, S. Christie^3^, D. Ayer^4^, J. Ford^4^, D. Bushnell^5^

#### ^1^Critical Path Institute, Tucson, AZ, United States; ^2^California Medical Clinic for Headache, Santa Monica, CA, United States; ^3^University of Ottawa, Ottawa, Canada; ^4^Eli Lilly and Company, Indianapolis, IN, United States; ^5^Evidera, Bethesda, MD, United States

##### **Correspondence:** R. Speck

Objectives**:** Determine meaningful score categories of the Migraine-Specific Quality of Life Questionnaire (MSQ) Role Function-Restrictive (RFR) domain to aid interpretation.

Methods**:** Two neurologists with clinical expertise in migraine were consulted and provided recommendations during this study. MSQ data from two episodic and one chronic clinical trial were pooled for analyses. The Patient Global Impression of Severity (PGI-S) was selected as the main anchor, response categories within each measure were used to plot histograms of baseline and Month 3 RFR scores and to evaluate responsiveness.

Results**:** Baseline RFR scores ranged from 0-100 with no floor or ceiling effects. The RFR distinguished change over time which was demonstrated with significant differences in mean change scores from baseline to Month 3. Review of the descriptive statistics, histograms, and responsiveness results informed the proposed categories (85-100, 75-84, 55-74, 40-54, and <40). At Month 3 the greatest proportions of patients that self-rated on the PGI-S as "Normal, not at all ill," or "Borderline ill" (51.0%) fell into the 85-100 category, "Mildly impaired" (36.9%) in the 75-84 category, "Moderately ill" (42.3%) in the 55-74 category, "Markedly ill" (39.9%) in the 40-54 category, and "Severely ill," and "Extremely ill" (46.1%) in the <40 category.

Conclusions**:** The proposed MSQ RFR score categories provide cut-offs to define disease severity and functional impairment, aiding score interpretation.

## P0227 Timely diagnosis of migraine – A prospective observational study assessing the potential to prevent unnecessary emergency department visits for headache

### H. Drangova^1^, N. Kofmel^2^, M. Branca^3^, D. Gloor^3^, B. Lehmann^2^, A. Exadaktylos^2^, S. Jung^1^, U. Fischer^1^, C. Schankin^1^

#### ^1^Inselspital, Bern University Hospital, Neurology, Bern, Switzerland; ^2^Inselspital, Bern University Hospital, Emergency Medicine, Bern, Switzerland; ^3^University of Bern, Bern, Switzerland

##### **Correspondence:** H. Drangova

Objective: Headache is one of the most common causes for a presentation at the emergency department (ED). Since migraine is the main etiology of headache in the ED, specific treatment might prevent ED consultations, if the diagnosis of migraine had been given earlier. The aim of this study is to assess the magnitude of missed diagnosis of migraine prior to ED visits.

Methods: This is a single-centre prospective study. Inclusion criterion was the presentation for acute headache at the ED. The treating physician assessed if patients had prior headache attacks fulfilling the ICHD-III criteria of migraine, and if they already had the diagnosis of migraine prior to the ED visit. Data was correlated with the discharge diagnosis.

Results: 214 patients were included of which 96 (45% of 214) received the diagnosis of migraine at discharge. Of those, the current ED visit was the first manifestation of migraine in 22. 43 already had a prior diagnosis of migraine, and 31 (i.e. 42% of 74) previously had fulfilled the criteria of migraine but had not been given the diagnosis.

Conclusion: About 2/5 of patients with previous migraine headaches who presented at the ED for acute attacks could have been given the diagnosis earlier. Potentially, specific acute treatment might have prevented the presentation at the ED. This study demonstrates the need for better recognition of migraine by pre-hospital healthcare providers including pharmacists, primary care physicians, and neurologists.

## P0228 Lockdown quality of life of migraine patients followed in a tertiary care headache outpatient clinics in Spain

### C. Treviño-Peinado, C. Sanchez

#### Hospital Universitario Severo Ochoa, Neurology Department, Leganés, Spain

##### **Correspondence:** C. Treviño-Peinado

Objective: To assess the impact of migraine on the patient quality of life (QoL) and their family relationships, taking into account the different treatments prescribed.

Methods: Cross-sectional descriptive monocentric study. Patients with diagnosis of episodic/chronic migraine according to the ICHD-3 treated during the first semester of 2020 were included. Demographic variables, pain characteristics, abortive and preventive treatment were collected. The MIDAS, HIT-6, MSQ v.2.1 QoL scales and questions related to the family environment were assessed.

Results: We included 55 patients, 94.5% women, with a mean age of 49 years. Triptans were taken by 74.5%. As preventive treatment they used OnabotulinumtoxinA 60%, anesthetic blockade 58.2%, Erenumab 4 patients and oral treatment combined or not with other techniques (78.2%). Average VAS 7.31. They had 12 days of migraine/month. The mean score of MIDAS scale was 43.13 (median 34, SD-38), HIT-6 62.84 (median 64, SD-7) and in the MSQv2.1 was 67 (median 67, DE-17). The Preventive Role of the MSQV2.1 was the most affected with a median of 75. The items most affected in family questions were "not being able to make noise at home and not being able to make plans".

Conclusion: People who needed follow-up in tertiary care headache outpatient clinics had a severe impact on their personal, social and work-life. It is necessary to validate specific questionnaires about the impact of migraineurs on the quality of life in their personal environment.

## P0229 Patient Perception of Migraine Impact and Burden: Survey Results From 10 European Countries

### U. Reuter^1^, J. M. Cohen^2^, L. Lyras^2^, M. Dusselier^3^, M. Geens^3^, P. Pozo-Rosich^4^

#### ^1^Charité University Hospital Berlin, Berlin, Germany; ^2^Teva Branded Pharmaceutical Products R&D, Inc., West Chester, PA, United States; ^3^InSites Consulting, Ghent, Belgium; ^4^Headache Unit, Neurology Department, Vall d’Hebron University Hospital, Barcelona, and Headache and Neurological Pain Research Group, Vall d’Hebron Institute of Research (VHIR), Universitat Autònoma de Barcelona, Barcelona, Spain

##### **Correspondence:** U. Reuter

Objective: Migraine is associated with reduced quality of life (QoL) and negative effects on the lives of patients, family, and friends. This survey evaluated patient perceptions of migraine diagnosis and treatment, stigma, and awareness and support in Europe.

Methods: Across 10 European countries, adult patients (≥18 years [yrs]) with self-reported ≥4 migraine days per month completed a 12-minute digital survey (between 19 November–6 December 2019).

Results: Of the 7,521 patients surveyed (25-54 yrs, 70%; female, 73%), 47% reported ≥3-year delay in diagnosis after initial symptoms; 31% reported ≥3-year delay in treatment after diagnosis. Overall, 58% of patients were satisfied with their prescription treatment and 61% with their physician. Patients frequently reported that migraine impacted their health/wellbeing (65%), social life (61%), and work/career (54%), and also affected their partner (69%) or children (57%). Overall, 44% of patients reported hiding their migraine, most commonly from their employer (63%). For migraine information, patients consulted doctors (66%), search engines (39%), medical websites (37%), or pharmacists (35%). A total of 41% of patients surveyed said the healthcare community is most responsible for addressing the impact of migraine and supporting patients.

Conclusions: These survey results confirm the unresolved impact and burden of migraine for those who suffer and the need to develop strategies and actions to minimize this impact and burden.

## P0230 Migraine Burden and Impact: Survey Results From 6 Countries in South America, Asia, and Australia

### M. Nattan Portes Souza^1^, J. M. Cohen^2^, T. Lengil^2^, M. Dusselier^3^, M. Geens^3^

#### ^1^Neurology Department, Hospital das Clínicas, Universidade de São Paulo, São Paulo, Brazil; ^2^Teva Branded Pharmaceutical Products R&D, Inc., West Chester, PA, United States; ^3^InSites Consulting, Ghent, Belgium

##### **Correspondence:** M. Nattan Portes Souza

Objective: Migraine is a common and disabling neurological disease that negatively affects the daily lives, careers, and relationships of patients. This survey assessed patient perceptions of migraine burden, diagnosis, treatment, stigma, awareness, and support across 6 countries in South America, Asia, and Australia.

Methods: Patient perceptions were evaluated in a digital 12-minute survey of adults (≥18 years [yrs]) with self-reported diagnosis of migraine (≥4 days/month) between 19 November–8 December 2019.

Results: Of 5,024 patients surveyed (25-44 yrs, 65%; female, 72%), 71% reported migraine symptom progression over time. Roughly half were satisfied with their preventive (51%) and acute (58%) migraine medications, and 60% were satisfied with their treating healthcare provider (HCP). Most patients (75%) felt understood by their HCP, but 55% felt having HCPs better educated about migraine would be beneficial. Patients commonly reported that migraine impacted their health/wellbeing (73%), work/career (59%), and familial relationships (44%). A total of 49% of patients reported hiding their migraine, most commonly from their employers (62%). Overall, 47% of patients surveyed felt HCPs are primarily responsible for addressing the impact of migraine and supporting patients.

Conclusions: These survey results highlight patient perceptions of the burden and impact of migraine, as well as a need to improve patient support across 6 countries in Asia, South America, and Australia.

## P0231 Project for the establishment of the italian migraine registry(i-graine-new)

### L. Fofi^1^, L. Hollander^2^, C. Aurilia^1^, G. Egeo^1^, G. Fiorentini^3^, N. Vanacore^4^, S. Bonassi^5^, C. Tomino^6^, P. Barbanti^1^

#### ^1^IRCCS San Raffaele, Headache and Pain Unit, Rome, Italy; ^2^CD Pharma Group, Scientific Direction, Milan, Italy; ^3^San Raffaele University, Rome, Italy; ^4^National Institute of Health, National Center for Disease Prevention and Health Promotion, Rome, Italy; ^5^RCCS San Raffaele, Unit of Clinical and Molecular Epidemiology; San Raffaele University, Rome, Italy; ^6^IRCCS San Raffaele, Scientific Direction; San Raffaele University, Rome, Italy

##### **Correspondence:** L. Fofi

Objectives: I-GRAINE-NEW aims to follow a large representative sample of patients with migraine with the following principal specific objectives:

1. provide information on migraine natural history and its evolution over time;
2. provide epidemiological, social and sanitary resource use data
3. identify the impact of patient management on prognosis

Methods: Data will be acquired through an observational, prospective study including 41 headache centers.

I-GRAINE-NEW will enroll a representative sample of 10% of adult patients with episodic or chronic migraine and will last at least 5 years. Patients will be evaluated by face-to-face interviews using a detailed semi-structured questionnaire. A subgroup of 6000 patients referred for a first outpatient visit, will be considered for a retrospective-prospective sub-study that will collect more in-depth information using the clinical interview, a daily headache diary and a series of PROMs.

Data will be stored in the electronic case report forms. All procedures will by compliant with GDPR 2016/697.

Results and conclusions: The I-GRAINE registry is expected to shed light on migraine unmet needs, define the endophenotypes, and improve clinical management, resulting in increased disease awareness, better healthcare resource allocation, and reduced economic burden.

## P0232 Functional REstoration with rEyvow (FREE): a US-based Cross-sectional Survey in Patients Taking Lasmiditan

### T. J. Schwedt^1^, L. Lombard^2^, E. Doty^2^, M. Vincent^2^, K. M Mills^2^, D. W Ayer^2^, D. S Mackie^3^, M. DeCongelio^3^, P. Hauck^2^, C. Dougherty^4^

#### ^1^Mayo Clinic, Phoenix, Phoenix, AZ, United States; ^2^Eli Lilly and Company, Indiana, IN, United States; ^3^Kantar Health, New York, NY, United States; ^4^MedStar Georgetown University Hospital, Washington, DC, United States

##### **Correspondence:** C. Dougherty

Objective: Assess respondents' ability to return to their usual activities and level of impairment of those activities after migraine acute treatment with lasmiditan.

Methods: A 15-min web-based survey was conducted on adult respondents who had enrolled in the US patient support program, redeemed a savings card, and treated at least 1 migraine attack with lasmiditan within the prior month. Symptoms/outcomes/ability to engage in various activities after recent lasmiditan-treated migraine attack were assessed using descriptive statistics.

Results: 78 respondents completed the survey (mean age 48 years/93.6% female/16.9 mean headache days/month). Untreated/unsuccessfully treated migraine attacks prior to ever taking lasmiditan resulted in inability/ severely impaired ability to perform various activities (Table). At the time of lasmiditan dosing (most recent attack), 49% had severe and 45% had moderate pain. By 2-hours post-dose, 94% respondents had some/complete pain improvement. After lasmiditan treatment, 45-75% respondents returned to their current/planned activities, except for planned activities outside home (22%). Extent of ability to perform current/planned activities varied by activity (Table). 77% respondents were satisfied with lasmiditan; 62% were satisfied with its ability to return them to their usual activities.

Conclusion: With lasmiditan, majority respondents were satisfied and able to return to their usual activities with no/some degree of impairment.

## P0233 The route of patients to the diagnosis of hemiplegic migraine and the peculiarities of this migraine type

### M. Bozhenko^1^, N. Bozhenko^1^

#### ^1^Danylo Halytsky Lviv National Medical University, Neurology department, Lviv, Ukraine

##### **Correspondence:** M. Bozhenko

Objective: Hemiplegic migraine(HM) is considered a rare type of migraine with an aura. Due to the brightness of neurological manifestations and lack of awareness about this form of migraine, these patients are often misdiagnosed.

Methods: A review and analysis of HM clinical cases and their route to diagnosis among outpatients of Lviv regional clinical hospital during 2019-2020.

Results: During 2019-2020 years 6 patients with HM were identified. 4-female, 2-male. With the age range from 16 to 32 years old. None of them previously had been diagnosed with HM. Only one had a familial form. The onset of a migraine was in the age 14-17 years. Time from attacks onset to diagnosis of HM was from 2 to 16 years. These attacks were previously diagnosed as panic attacks, TIA, and epilepsy. Misdiagnosing led to a lack of adequate migraine treatment. In the case of a female with 16 years of hemiplegic migraine attacks history, it led to migrainous infarction after the last attack. In 2 more patients foci of gliosis on MRI were described. Also, we found typical weakness spreading from the distal parts to the proximal.

Conclusion: Lack of awareness among general practitioners and pediatricians leads to the fact that hemiplegic migraine attacks are often perceived by doctors as other paroxysmal conditions, which leads to a lack of adequate treatment of these patients, which significantly affects their quality of life and can sometimes lead to complications such as migrainous infarction.

## P0234 Neck symptoms as a risk factor for headache: a scoping systematic review

### T. McPartland^1^, N. McCool^1^, L. Best^2^, R. Forbes^2,1^

#### ^1^Queens University Belfast, Medical School, Belfast, United Kingdom; ^2^Southern HSC Trust, Neurology Centre, Portadown, United Kingdom

##### **Correspondence:** R. Forbes

Background and Objective: People with migraine report neck pain before and during headache. People with neck disorders experience headache, although cervicogenic headache remains a contentious clinical diagnosis. To quantify the relationship between neck symptoms and headaches we sought studies describing headache frequency in unselected populations with neck pain and/or stiffness.

Methods**:** structured MeSH search of MEDLINE 1969-Dec 2020. We included publications in adult or paediatric populations which enabled calculation of odds ratios for neck symptoms as a risk factor for Unspecified Headache (UH), Migraine (M), Tension-Type Headache (TTH) or Chronic Tension-Type Headache (CTTH).

Results**:** We found 1868 articles, reviewed 164 full-text and included 19 studies with 502,744 subjects. Headache risk with neck symptoms are Adults: UH 4.41 (95%CI 4.32 - 4.51), M 3.50 (3.31-3.70), TTH 7.18 (3.86-13.38), CTTH 1.72 (0.52-5.85); Paediatric: UH 2.27 (2.02-2.50), M 3.16 (2.46 - 4.07), TTH 1.37 (0.92-2.03), CTTH 2.27 (1.42-3.63).

Conclusions**:** Neck symptoms are a significant risk factor for Unspecified Headaches and Migraine but the relationship with Tension-Type Headache and Chronic Tension-Type Headache is different for adults and paediatric groups. If neck symptoms are a risk factor for disabling headache then a risk-factor reduction approach may simplify headache treatment and eliminate controversy surrounding cervicogenic headache.

## P0235 Impact of Headache on Quality of Life in United States Veterans: a Qualitative Study

### I. Gosnell^1,2^, T. Damush^3,4,2^, H. Lindsey^2,5^, R. Goldman^2,6,7^, A. Grinberg^1,2^, S. Riley^4^, L. Burrone^2^, S. Baird^4^, B. Fenton^2,5^, J. J. Sico^2,5^, E. K. Seng^1,8,2,9^

#### ^1^Yeshiva University, Ferkauf Graduate School of Psychology, Bronx, NY, United States; ^2^VA Connecticut Healthcare System, Headache Centers of Excellence, Research & Evaluation Center, West Haven, UT, United States; ^3^Indiana University, School of Medicine, Indianapolis, IN, United States; ^4^Roudebush VAMC, Veterans Health Administration Health Services Research and Development (HSR&D) Center for Health Information and Communication (CHIC) and Quality Enhancement Research Initiative Expanding expertise Through E-health Network Development (EXTEND) QUERI Centers, Indianapolis, IN, United States; ^5^Yale University, School of Medicine, New Haven, CT, United States; ^6^Warren Alpert Medical School of Brown University, Department of Family Medicine, Providence, RI, United States; ^7^Harvard University T.H. Chan School of Public Health, Boston, MA, United States; ^8^Albert Einstein College of Medicine, Bronx, NY, United States, ^9^Montefiore Medical Center, Bronx, NY, United States

##### **Correspondence:** I. Gosnell

Objective: Headache is a common, chronic and disabling disease with episodic exacerbations that impact quality of life. We evaluated health-related quality of life (HRQoL) in veterans living with headache and receiving care in the Veterans Health Administration (VHA) Headache Center of Excellence program.

Methods: We conducted semi-structured qualitative interviews with a purposeful sample of 20 veterans across VHA. Patients were asked about headache characteristics, management, and healthcare. We qualitatively coded all NVivo files using an apriori/emergent codebook. We conducted a comparative case analysis to identify emergent domains of HRQoL.

Results: The 20 participants (16 men, 4 women) had a mean age of 54 years (SD=13.77) and headache diagnosis of migraine (n=15), other (n=7), tension-type (n=3), cluster (n=1), medication overuse (n=1), and/or post-traumatic headache (n=1). Participants were white (n=15), Black (n=4), Asian (n=1), and Hispanic (n=1). Headache impacted patients" HRQoL via: 1) hopelessness around lack of control; 2) frustration that pain occurred at random; 3) regardless of treatment, relief did not last; 4) headache attacks led to social withdrawal; and 5) headache attacks prevented participation in life activities.

Conclusions: Chronic headache pain and unpredictable symptom occurrence contribute to reduction in HRQoL in people living with headache. Further research is needed into how to maintain or improve quality of life in veterans with headache.

## P0236 Headache As A Warning Sign Of Acute Stroke: Prehospital Services Study In Bishkek, Kyrgyzstan

### I. Lutsenko^1^, G. Abdumanapova^2^

#### ^1^Kyrgyz State Medical Academy, Advanced Training Department, Bishkek, Kyrgyzstan; ^2^Novosibirsk State University, Neurlogy, Novosibirsk, Russian Federation

##### **Correspondence:** I. Lutsenko

Background: Although headache is a noticeable symptom and can follow stroke manifestation, it is often underestimated in the acute period of stroke in Kyrgyzstan.

Aim: We aimed to analyze the prevalence of the headache as the symptom in the acute stroke patients in Bishkek.

Methods: In an observational study we studied logistics, onset symptoms and behavior of 477 acute stroke patients, examined by the emergency medical team and hospitalized in stroke units in a period from November, 2019 till March, 2020.

Results: Headache was presented as a reason for call to emergency services in 22% of cases of all strokes, while 32% of patients mentioned headache in the stroke onset in the interview with the emergency team. 69% of patients had acute headache and in 48% it followed with the weakness in limbs and speech disturbances. Headache was expressed in high hypertension with systolic blood pressure higher than 180 (OR=4, 95% CI, p = 0.001;) was described as bilateral, dull, continuous and localised in temples and occipital part (78%) and was more associated with a lacunar stroke. Patients used hypotensive medications (captopril), paracetamol+aspirin to abort the headache and green tea as a remedy.

Conclusion: Headache was a frequent symptom in a stroke onset and is associated with the sudden rise of a blood pressure in a stroke onset. Patients encouraged medical personnel to include headache as a symptom in stroke recognition algorithms which exist in Kyrgyzstan.

## P0237 Primary Headache Disorder Among Ukrainian Students

### O. Tsurkalenko, L. Dzyak, A. Tsurkalenko

#### State Institution "Dnipropetrovsk medical academy of Ministry of Health of Ukraine", Neurology and Neurosurgery, Dnipro, Ukraine

##### **Correspondence:** A. Tsurkalenko

Background and Objectives: Primary headaches are remarkably prevalent worldwide. We examined the prevalence of primary headache disorders among students of higher education institutions.

Methods: We conducted study that included 1381 students of higher education institutions in the 2019/2020 academic year. Prevalence and attributable burden of headaches, definite and probable migraines, definite and probable tension-type headaches, chronic headaches, and medication-overuse headaches were assessed using the Headache-Attributed Restriction, Disability, Social Handicap, and Impaired Participation (HARDSHIP) questionnaire.

Results: Of 1381 questionnaires that were distributed, 1,101 students completed the questionnaire. The study population consisted of 36% man and 64%woman with a mean age of 18,5±1.1 years. The 1-year prevalence of primary headache disorders was 41.5%, with more middle secondary-year than thirst-year students (51.7 vs. 29,8%; *p*<0.02). When stratified according to diagnostic criteria, migraine headaches were the most frequently reported (22,3%), followed by tension type headaches (19,1%), chronic headaches (2,8%), and probable medication-overuse headaches (2,4%).

Conclusions: Primary headaches are remarkably common in Ukrainian students, with migraine headaches being the most frequently reported type. These findings necessitate the direction of health services such as lifestyle modification training to prevent primary headache in this population.

## P0238 Migraine Prevalence and Impact among Medical Students of the University of Calabar, Southern Nigeria

### S. Ozomma^1^, S. Oparah^1^, E. Olose^2^, U. Asibong^2^

#### ^1^University of Calabar Teaching Hospital, Department of Internal Medicine, Calabar, Nigeria; ^2^University of Calabar, Calabar, Nigeria

##### **Correspondence:** S. Ozomma

Background: Migraine among medical students further compounds the demanding nature of medical training.

Objective**:** This study aimed to determine migraine prevalence, associated absenteeism and headache-related health-seeking roles among undergraduate medical students of the University of Calabar, Nigeria.

Methods: In this cross-sectional descriptive study, we used a structured questionnaire incorporating the International Headache Society criteria for migraine to identify migraine among the aforementioned students, besides obtaining data on headache-related absenteeism and health-seeking behavior. Two hundred and twenty participants, comprising 62.3% males and 37.7% females, completed the study.

Results: Overall, 5.9% of them had migraine headaches, with gender-specific prevalence values of 4.4% and 8.4% for males and females, respectively. 53.8% of the affected persons had migraine with aura. The age at migraine onset ranged from 11 to 16 years, with a mean (standard deviation) and median ages of 13.6 (1.92) years and 13.5 years, respectively. All the students diagnosed with migraine reported being absent from scheduled activity because of headaches. More than half of those with migraine relied on self-medication; whereas, only a quarter had consulted a physician for their migraine attacks.

Conclusion: Migraine was common among this set of medical students, with frequent headache-induced absenteeism. There was poor utilization of available healthcare resources, for migraine treatment, even among the medical students with access to tertiary health care.

**Fig. 1 (abstract P0238).**
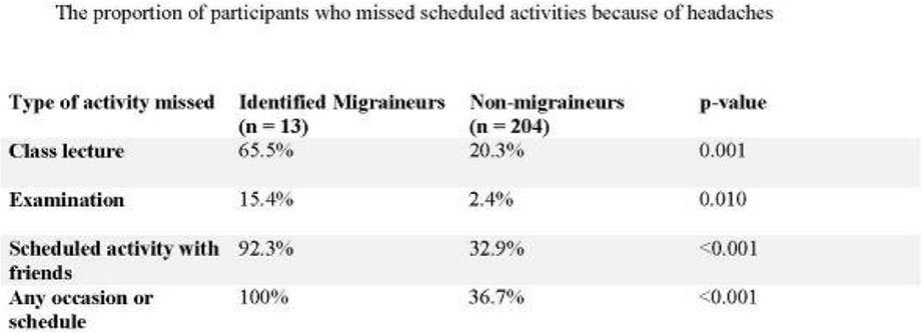
See text for description

**Fig. 2 (abstract P0238).**
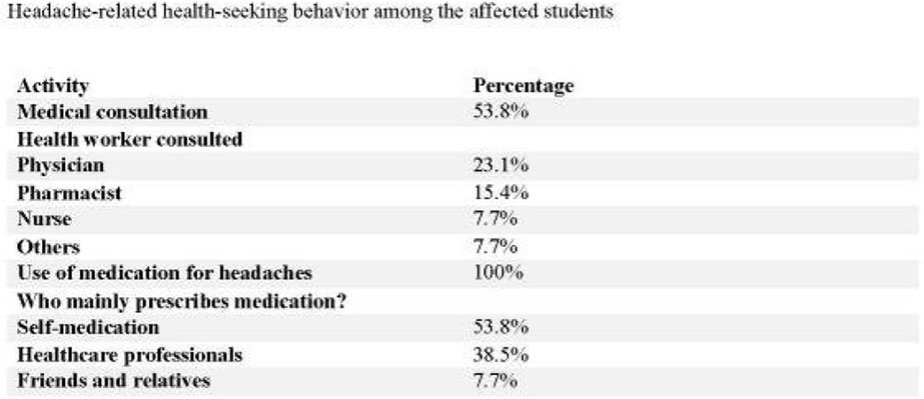
See text for description

## P0239 Factors Affecting Migraine during Fasting States

### A. Abdou, M. Hamdy, E. Hamdy

#### Alexandria University Faculty of Medicine, Department of Neurology, Alexandria, Egypt

##### **Correspondence:** A. Abdou

Background and objective: Fasting is adopted by many individuals worldwide either for health optimization or for religious reasons. Fasting, however, exacerbate migraine. This work aimed at studying factors that may contribute to migraine exacerbation during long fasting among Egyptian migraineurs who fast for religious reasons 16 hours daily for one full month (*Ramadan*).

Methods: This was a cross-sectional study conducted on 30 migraine patients. Patients filled a diary about dietary consumption, fluid intake, and sleep habits during *Ramadan*. A comparative analysis was made between days with and days without migraine.

Results: Of 222 days recorded, 48 days were with migraine and 74 days were without. On regression analysis, initial insomnia (OR 5.1, CI 2.3-10.9, p<0.001), fried food (OR 2.9, CI 1.19-6.79, p=0.018), coffee (OR 2.3, CI 1.18-4.88, p=0.015), citrus fruits (OR 2.3, CI 0.97-19.5, p=0.037), watermelon (OR 12.7, CI 1.68-96.5, p=0.002), dairy products (OR 2.14, CI 1.17-3.91, p=0.012) were found to increase the odds of migraine occurrence. Factors associated with low odds of migraine occurrence were fluid intake (OR 0.88, CI 0.77-0.88, p=0.034), frequent meals (OR 0.44, CI 0.26-4.89, p=0.004), and dessert consumption (OR 0.49, CI 0.26-0.94, p=0.025).

Conclusion: Sleep habits, fluid intake, coffee consumption and dietary habits contribute to migraine occurrence during long fasting states and should be considered for adequate control of the disease during fasting.

## P0240 Locating Organizations and Their Methods in Registrations of Clinical Migraine Trials: Analysis of ClinicalTrials.gov

### P. Zhang^1^, T. Do^2^

#### ^1^Rutgers Robert Wood Johnson Medical School, New Brunswick, NJ, United States; ^2^Rigshospitalet Glostrup, Neurology, Copenhagen, Denmark

##### **Correspondence:** P. Zhang

Introduction: ClinicalTrials.gov is a centralized venue for monitoring clinical research and allows access to information on publicly and privately funded studies.

Objective: To identify major organizations conducting clinical migraine trials and the frequency of different study designs.

Methods: Utilizing ClinicalTrials.gov application programming interface, we extracted studies including individuals with migraine from February 29, 2000 to July 28, 2020 for the following: (1) host organization; (2) study type; (3) primary purpose; (4) intervention model; (5) allocation.

Results: We included 921 entries encompassing 423 organizations. The top 32 (3%) organizations each produced ≥5 entries totaling 40.0% of entries. Approximately 86% were interventional studies; 13.6% were observational studies. Randomized design allocation is the most frequent. The most frequent primary purpose is treatment (62.4%) followed by prevention (13.0%). There were 56.9% parallel assignment, 15.2% single group assignment, and 12.4% crossover assignment models.

Discussion: A minority of organizations contribute to a significant number of registrations of clinical migraine trials. The most common study is interventional, randomized, with parallel assignment for treatment purpose. Organizations should aim to improve pre-registrations to increase transparency and to reduce introduction of bias in clinical studies.

## P0241 Monosodium glutamate (MSG) induces headache-like behaviors in rats

### T. Benbow^1^, M. Ranjbar Ekbatan^1^, G. H. Y. Wang^1^, F. Teja^1^, F. G. Exposto^2^, P. Svensson^2^, B. Cairns^1^

#### ^1^University of British Columbia, Pharmaceutical Sciences, Vancouver, Canada; ^2^Aarhus University, Dentistry and Oral Health, Aarhus, Denmark

##### **Correspondence:** T. Benbow

Objective: Oral ingestion of MSG results in reports of headache and craniofacial tenderness in healthy humans. We examined whether systemic administration of MSG could produce evidence of headache in rats.

Methods: The behavior of Sprague Dawley rats (6 male, 6 female) was video recorded before and after intraperitoneal injections of either MSG (1-1000 mg/kg), nitroglycerin (GTN, 10 mg/kg) or normal saline in a randomized order by a blinded experimenter. Behaviors (grimace score, head shakes, rearing, head scratches, facial grooming, temporalis muscle mechanical withdrawal threshold (MT)) were evaluated from the recordings by two blinded assessors. Plasma glutamate and a-CGRP concentrations after administration of 1000 mg/kg MSG were measured in anesthetized rats as a terminal experiment. Significant differences were assessed with two-way repeated measures ANOVA.

Results: Compared with GTN and saline, MSG (500-1000 mg/kg) significantly increased grimace scores and headshakes, and significantly decreased rearing, head scratches, and facial grooming for 20-30 minutes post administration. MT was unchanged. Plasma glutamate and a-CGRP concentrations increased from 30 to 3800 mM and 2 to 10 pg/ml, respectively, 30 min post injection.

Conclusion: MSG induces headache-like behaviors in a dose-related manner associated with increased plasma glutamate and CGRP concentrations. These findings suggest that, like humans, systemic administration of MSG in rodents may induce headache.

## P0243 Evidence that monosodium glutamate (MSG) administration induces nausea-like behavior in rats

### T. Benbow^1^, M. Ranjbar Ekbatan^1^, F. Teja^1^, F. G. Exposto^2^, P. Svensson^2^, B. Cairns^1^

#### ^1^University of British Columbia, Pharmaceutical Sciences, Vancouver, Canada; ^2^Aarhus University, Dentistry and Oral Health, Aarhus, Denmark

##### **Correspondence:** T. Benbow; B. Cairns

Objective: Oral ingestion of MSG results in reports of headache and nausea in healthy humans. We examined whether systemic administration of MSG could evoke a nausea-like state in rats.

Methods: The behavior of Sprague Dawley rats (6 male, 6 female) was video recorded before and after intraperitoneal injections of either MSG (500-1000 mg/kg), nitroglycerin (GTN, 10 mg/kg) or normal saline. Treatments were given in a randomized order by a blinded experimenter. The duration of lying-on-belly (LOB) nausea-like behavior was evaluated by two blinded assessors. Cutaneous temperature of the nose was measured before and every 10 minutes after intraperitoneal injections via infrared thermography. Significant differences were assessed with two-way repeated measures ANOVA. Correlation between LOB and facial cutaneous temperature was determined with Pearson"s correlation analysis.

Results: Compared with GTN and saline, MSG (1000 mg/kg) significantly increased LOB behavior between 20 and 30 minutes post administration. Nose cutaneous temperature was significantly decreased compared to GTN and saline from 10-30 minutes post MSG (1000 mg/kg) administration. A significant inverse correlation between LOB behavior duration and nose cutaneous temperature was found.

Conclusion: MSG induces nausea-like behavior in rats that consists of increased LOB duration and facial cutaneous hypothermia. This data suggests that, like humans, systemic administration of MSG to rats may induce nausea.

## P0245 Musculoskeletal impairment of the cervical spine in the 4 phases of the migraine cycle

### S. Di Antonio^1^, M. Castaldo^1^, M. Ponzano^2^, F. Bovis^2^, P. Torelli^3^, C. Finocchi^4^, L. Arendt-Nielsen^1^

#### ^1^School of Medicine, Aalborg University, Denmark., Department of Health Science and Technology, Center for Pain and Neuroplasticity (CNAP), Aalborg, Denmark; ^2^University of Genoa, Department of Health Sciences (DISSAL), Section of Biostatistics, Genova, Italy; ^3^University of Parma, Headache Centre, Department of Medicine and Surgery, Parma, Italy; ^4^Ospedale Policlinico San Martino, Headache Centre, IRCCS, Genova, Italy

##### **Correspondence:** S. Di Antonio

Objective**:** Assess if patients with episodic migraine (EM) have increase musculoskeletal impairments of the cervical spine compared to healthy controls independently by the phases of the migraine cycle and the presence of neck pain

Methods: In this multicenter cross-sectional, observational study, EM patients and healthy controls (age 18-65) were included. Cervical active range of movement (AROM), craniocervical flexion test (CCFT), flexion rotation test (FRT), and pressure pain threshold (PPT) over the neck were assessed. A linear regression model using the variable group to predict the results was performed. Healthy controls were used as reference groups adjusting the model for age, sex, and disability due to neck pain

Results: 42 Control, 32 interictal EM, 34 Preictal EM, 25 Ictal EM, and 23 postictal EM were included. The AROM was lower only in Ictal EM compared to healthy controls (p=0.033), with no other differences (p>0.111). Healthy controls had higher CCFT (p<0.001), lower FRT (p<0.001) compared to EM patients in all phases with no differences in neck PPT (p>0.096)

Conclusion: EM patients in all phases of the migraine cycle have reduced functionality of deep cervical flexors muscles and restricted passive range of motion of the upper cervical spine. No neck hyperalgesia was observed in any of the phases. The active range of motion was impaired only in the ictal phase, suggesting that acute headache could cause a reduction in functionality of the neck movement.

**Fig. 1 (abstract P0245).**
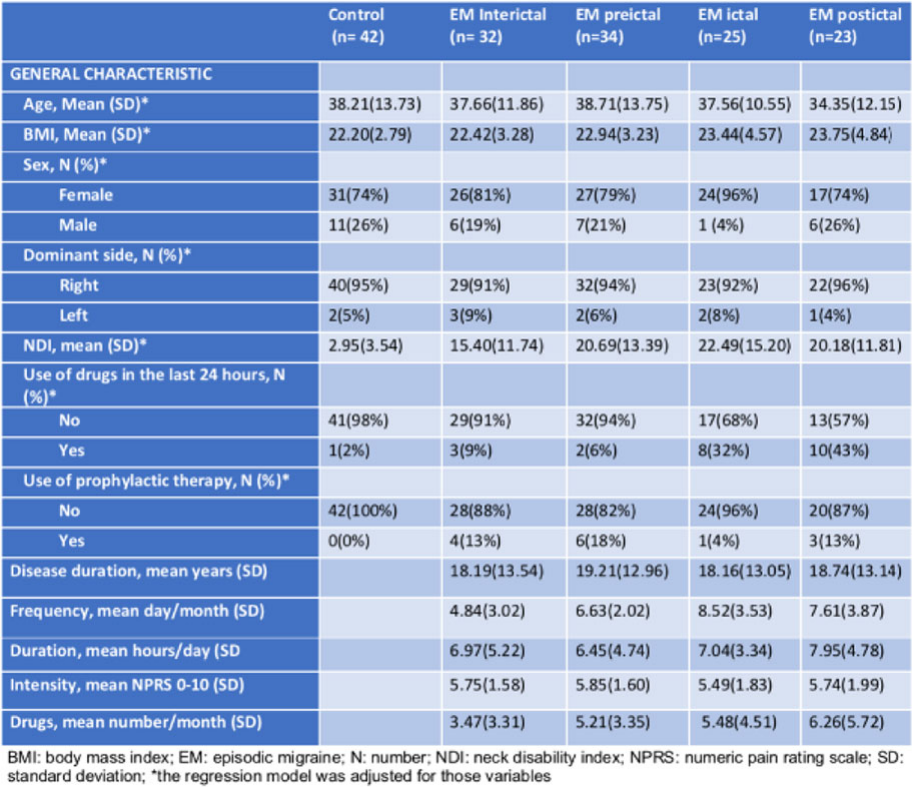
See text for description

**Fig. 2 (abstract P0245).**
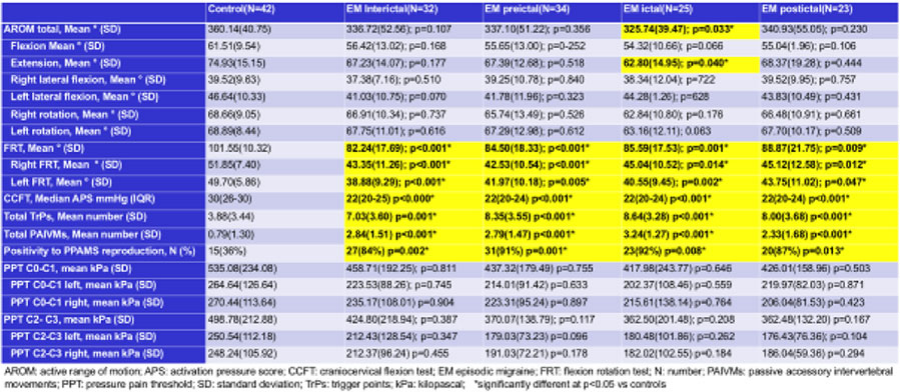
See text for description

## P0246 Part II:Diet-induced obesity induces cutaneous allodynia in female rats

### C. S. J. Westgate, I. Israelsen, R. H. Jensen, S. Eftekhari

#### Danish Headache Center, Copenhagen, Denmark

##### **Correspondence:** C. S. J. Westgate

Objectives: Headache is a predominant co-morbidity in raised intracranial pressure (ICP) disorders. Obesity is associated with an increased risk of idiopathic intracranial hypertension and migraine. We assessed the effect of diet-induced obesity (DIO) on cephalic sensitivity in a non-traumatic raised ICP model.

Methods: Female Sprague-Dawley rats received high fat diet (60% fat) or matched control diet. Blinded periorbital and hind paw pain thresholds were measured throughout the diet by a von frey anaesthesiometer. Prior to implantation with an ICP telemetric device, body composition was assessed via dual energy x-ray absorptiometry (DEXA). Expression of headache relevant genes was studied in the trigeminal ganglia (TG).

Results: Over the course of a 15-17 week diet DIO rats demonstrated cyclical periorbital allodynia (P=0.003) but normal hind paw thresholds. On the day of DEXA, DIO rats were 17.5% heavier (P<0.0001) with a higher abdominal (Abd) fat percentage (P<0.0001) with raised ICP (P=0.005). DIO rats had periorbital allodynia (P<0.0001) with an inverse correlation with Abd fat percentage (r=-0.65, P=0.0005). Hind paw thresholds were normal. In support, DIO TG had increased expression of *Trpv1* (P<0.01) and *Cgrpa* (CGRPα) (P<0.01). Pro-inflammatory gene expression was unaltered.

Conclusion: DIO rats demonstrated cephalic cutaneous allodynia likely involving the CGRP pathway, in the context of raised ICP. Further investigation into the mechanisms could lead to novel therapeutics.

## P0247 Altered kynurenine pathway in episodic migraine patients: potential peripheral biomarkers during the interictal period

### B. Tuka^1,2^, A. Nyári^3^, E. K. Cseh^3^, T. Körtési^1,4^, D. Veréb^5^, F. Tömösi^6^, G. Kecskeméti^6^, T. Janáky^6^, J. Tajti^3^, L. Vécsei^1,2^

#### ^1^Faculty of Medicine, University of Szeged, Department of Neurology, MTA-SZTE Neuroscience Research Group, Szeged, Hungary; ^2^Faculty of Medicine, University of Szeged, Department of Neurology, Interdisciplinary Excellence Centre, Szeged, Hungary; ^3^Faculty of Medicine, University of Szeged, Department of Neurology, Szeged, Hungary; ^4^Faculty of Health Sciences and Social Studies, University of Szeged, Szeged, Hungary; ^5^Faculty of Medicine, University of Szeged, Department of Radiology, Szeged, Hungary; ^6^Faculty of Medicine, University of Szeged, Department of Medical Chemistry, Interdisciplinary Excellence Centre, Szeged, Hungary

##### **Correspondence:** B. Tuka; A. Nyári; T. Körtési

Background and objective: Several members of kynurenine pathway (KP) are able to influence the glutamatergic neurotransmission, subsequently the pathomechanism of migraine. Our aim was to reveal the complete peripheral tryptophan (Trp) catabolism, which comprises the KP in episodic migraineurs and its relationship with clinical characteristics of patients.

Methods: Female migraineurs (n=50) and healthy subjects (n=34) aged between 25-50 years were enrolled. Peripheral blood samples were collected from subjects (during both the interictal/ictal periods in patients). 12 metabolites were determined by neurochemical measurements.

Results: Significantly decreased plasma concentrations of Trp (p<0.025), L-kynurenine (p<0.001), kynurenic acid (KYNA) (p<0.016), anthranilic acid (ANA) (p<0.007), picolinic acid (PICA) (p<0.03), 5-hydroxy-indoleaceticacid (5-HIAA) (p<0.025) and melatonin (MELA) (p<0.023) were detected in the interictal period of migraine without aura patients compared to controls, while elevated ANA, 5-HIAA and MELA levels were found during attacks. Correlations were identified between the followings: xanthurenic acid, MELA–attack frequency, KYNA–menstruation cycle-related headache, PICA–last attack before ictal sampling.

Conclusions: Metabolic imbalance is assumed during the attack free period, which can manifest in depressed peripheral KP contributing glutamate excess, neurotoxicity and generalised hyperexcitability. KP may have clinical relevance in migraine.

## P0250 Neurotransmitter exchange in cephalalgia in women with gynecological pathology, depending on the stability of the menstrual cycle

### Y. Karakulova, N. Selyanina, P. Khasanova, A. Ivanova

#### E.A.Vagner Perm State Medical University, Neurology, Perm, Russian Federation

##### **Correspondence:** Y. Karakulova

Objective: to assess the role of serotonin in the occurrence of cephalalgia in women with gynecological pathology.

Materials and methods**:** clinical, visual analog scale (VAS), the Hospital Anxiety and Depression Scale (HADS), Spielberger-Hanin test, serotonin in the blood (by ELISA), Statistica 10.

Results: We examined 61 patients with headaches and gynecological pathology with a stable (41 women) and unstable (20 women) menstrual cycle at the age of 15 to 41 years. 43 of them had tension headaches (TH), 10 patients had migraines (M). The amount of serum serotonin of patients with TH was significantly lower (116.7±60.3 ng/ml, p=0,009) than of patients with M (276.67±94.5 ng/ml) and in the control group (256.8±24.38 ng/ml). In women with a stable menstrual cycle, the VAS was 7.14±2.32 points, the blood serotonin level was 194.64±34.8 ng/ml, and with an unstable one, it is 9.05±4.63 points and 132.8±53.4 ng/ml, respectively (p=0.041). In patients with a stable cycle, depression is not observed, and when the cycle is disrupted, is determined severe depression (13.9±9.1 points, p=0.000810). The correlation analysis revealed a relationship between the indicators of headache intensity and personal anxiety (РА) (r=0.3552; p=0.0050), depression and blood serotonin levels (r=-0.4218; p=0.0013).

Conclusions: In women with gynecological pathology, TH is more common than M. Women with an unstable cycle are more likely to suffer with depression and have low serum serotonin levels.

## P0251 The EEG pattern to Hyperventilation in patients of different ages with various neurological disorders

### I. Khachidze, M. Gugushvili

#### Beritashvili Centre of Experimental Biomedicine, Ceorgia National University (SEU), Tbilisi, Georgia

##### **Correspondence:** I. Khachidze

Introduction: HPT (Hyperventilation provocation tests) is useful for the study of electroencephalography (EEG). The research aimed classified pathological EEG responses to HPT according to different parameters:time of manifestation and age of patients with neurological disorder headaches, fatigue etc.

Methods: The outpatient applied to the Beritashvili Centre of experimental Biomedicine. The control group consisted of 1201 participants whose EEG response to hyperventilation was within normal range. The three types of pathological EEG responses to hyperventilation (PERH) were detected in 985 outpatients PERHI corresponds to disorganization of basic rhythm. PERHII-paroxysmal discharges without epileptic elements. PERHIII-epileptic activity. The patients were divided by PERH into the following age groups:3-6, 7-12, 13-18, 19-30, 31-50, 51higher.

Results**:** In all age revealed disorganization of basic EEG rhythm in the first, second and third minutes of HPT. In the first minute of HPT the three types of PERH were revealed in all age, which was not observed in the second and third minutes.

Conclusion: 3 main types of PERH detected in all age-groups of patients might be informative for correct diagnosis, monitoring treatment plans and functional outcomes. The EEG reaction to hyperventilation undergoes permanent changes during brain maturation and development. Point out that children, adults, and elderly have different individual sensitivity to hypocapnia developed during hyperventilation.

## P0252 Antagonism of CGRP receptor: central and peripheral effects in animal models of migraine

### R. Greco^1^, C. Demartini^1^, M. Francavilla^1^, A. M. Zanaboni^1,2^, D. Martinelli^1^, G. Castellazzi^1^, C. Tassorelli^1,2^

#### ^1^IRCCS Mondino Foundation, Pavia, Italy; ^2^University of Pavia, Department of Brain and Behavioral Sciences, Pavia, Italy

##### **Correspondence:** C. Demartini

CGRP is a key component of migraine pathophysiology at peripheral and, probably, also at central sites.

Objective: To evaluate the effect of CGRP blockade in peripheral and central areas of the nervous system in two animal models of migraine.

Methods: Male Sprague-Dawley rats were exposed to nitroglycerin (NTG) or vehicle and treated with the CGRP antagonist olcegepant or vehicle 1h before undergoing the orofacial formalin test. In another group of rats we applied the inflammatory soup (IS) on the dura mater to induce neurogenic inflammation model and 10 min later treated them with olcegepant or vehicle. All animals were sacrificed at the end of the experimental session and gene expression of CGRP and pro-inflammatory cytokines were evaluated in the trigeminal ganglion, meninges and medulla-pons.

Results: Olcegepant significantly attenuated NTG-induced trigeminal hyperalgesia in the orofacial formalin test, while decreasing pro-inflammatory cytokines and CGRP mRNA levels in all areas. Similar effects were also observed in the neurogenic inflammation model.

Conclusions: The findings show that the antagonism of CGRP receptor induces changes in molecules that are relevant for migraine pathophysiology in both peripheral and central nervous system areas. This observation may be clinically relevant, as migraine patients not responding to monoclonal antibodies targeting CGRP, whose effect is mostly peripheral, may still benefit from the treatment with a CGRP antagonist.

## P0253 T2-Space protocol MRI-- A Safe, Minimally Invasive Screening Tool for Spinal CSF Leak causing Spontaneous Intracranial Hypotension

### B. Daripa, S. Lucchese

#### University of Missouri Health Care, Neurology, Columbia, SC, United States

##### **Correspondence:** B. Daripa; S. Lucchese

Objective: Spontaneous Intracranial Hypotension due to spinal CSF leak is a secondary cause of headache with potentially devastating consequences for patients who suffer from it. Diagnosis is complicated by the lack of a reasonable, minimally invasive screening test. This results in many patients going undiagnosed for years after headache onset. Current testing approaches are either overly invasive, such as CSF infusion protocols, or both invasive and insensitive, such as lumbar puncture with opening pressure or CT myelogram as it is commonly used—both require access to the thecal space and lack sensitivity. CT Myelogram will not see a leak if it is intermittent, or very slow, and in the setting of spinal CSF leak, opening pressure on LP may be high, low, or normal. A potential remedy for this state is T2 Space Protocol spinal MR Myelogram.

Methods: Chart review of patients who have had T2 space MRI and Myelogram to assess if findings are consistent between the study types.

Results: We have few patients who have had both studies at our facility; we did find 2 who had, and these show clear indications of CSF leak on both myelogram and T2 space protocol MRI.

Conclusions: The presence of CSF leak-evidence on T2 space MRI corroborated by Myelogram demonstrates the potential value of this protocol as a screening tool. It is highly sensitive for spinal pathology and minimally invasive, making it an excellent choice for screening patients suspected of spinal CSF leak.

**Fig. 1 (abstract P0253).**
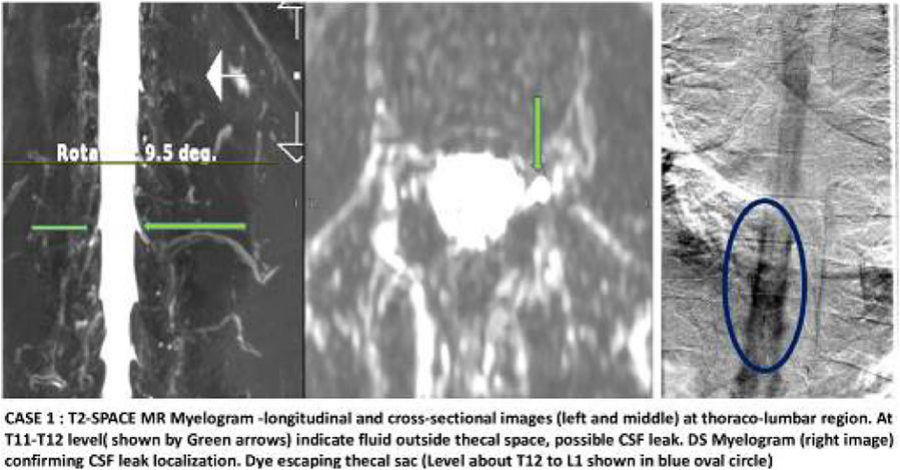
See text for description

**Fig. 2 (abstract P0253).**
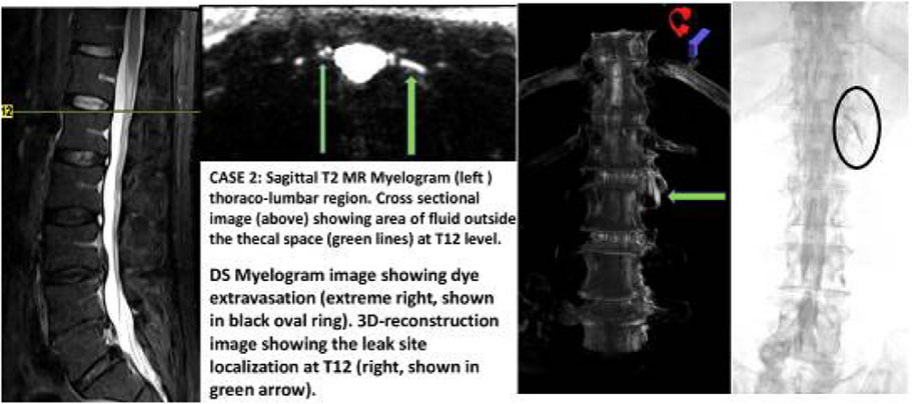
See text for description

## P0255 Cortical spreading depression alters transcriptomic profile in meninges and associated vasculature of rats

### M. Wang, L. Jiang, K. Sun

#### Xi'an Jiaotong-Liverpool University, Department of Biological Sciences, Suzhou, China

##### **Correspondence:** M. Wang

Objectives: Cortical spreading depression (CSD) induces activation of the meninges and associated vasculature (MAV), a key process leading to trigeminal nerve activation and migraine pain. However, how CSD mediates these phenomena in migraine is poorly understood. The aim of this study is to examine CSD-mediated transcriptomic profile of the MAV.

Methods**:** CSD was recorded using electrophysiology in rats. RNA-seq analysis and qPCR were applied for gene expression analysis.

Results: RNA-seq analysis showed that multiple CSD rapidly induced profound changes in gene expression profile in the ipsilateral MAV of rats. CSD induced a total of 1126 genes with altered expression levels, of which 953 CSD-induced DEGs were upregulated and 173 CSD-induced DEGs were downregulated in the rat ipsilateral MAV. All these genes were, for the first time, identified to be altered by CSD in the MAV. These transcriptomic changes accounts to 4.8 % of genes identified in the MAV of rats. Furthermore, these changes of transcriptomic profile were largely associated with altered pathways in synaptic transmission, ion transport and neuroinflamma-tion.

Conclusions: These data implied that MAV activation may be attributed to changes in its transcriptomic profile which are markedly induced by CSD.

The authors declare that there is no conflict of interests.

## P0256 TRPA1/SFK signaling in trigeminal ganglion contributes to migraine pathophysiology

### L. Nie^1,2^, J. Quinn^2^, M. Wang^1,2^

#### ^1^Xi'an Jiaotong-Liverpool University, Department of Biological Sciences, Suzhou, China; ^2^University of Liverpool, Department of Pharmacology and Therapeutics, Liverpool, United Kingdom

##### **Correspondence:** L. Nie

Background and objective: TRPA1 is a promising therapeutic target in migraine by responding to migraine triggers and regulating migraine pathogenesis. However, TRPA1-invovled signaling events in migraine are poorly understood. In this study, we explored the potential role of Src family kinases (SFK) in TRPA1-mediated migraine pathophysiology in trigeminal ganglion (TG), the key anatomical region for migraine pain transmission from periphery to brain.

Methods: A mouse trigeminal ganglia (TG) tissue culture model was applied. The level of SFK activation was detected using Western Blot; calcitonin gene-related peptide (CGRP) release was detected using ELSIA and IL-1β gene expression was detected using qPCR.

Results: The results showed that activation of TRPA1 by umbellulone increased the level of phosphorylated SFK at Y416 in TG, which was reduced by inhibition of protein kinase A by PKI (14-22) amide. Moreover, inhibition of SFK activity by saracatinib reduced umbellulone-enhanced CGRP release and IL-1β gene expression in TG.

Conclusions: These findings suggest that SFK participate in TRPA1 signaling in TG to mediate neuropeptide release and neuroinflammation, leading to peripheral sensitization and the development of migraine.

**Fig. 1 (abstract P0256).**
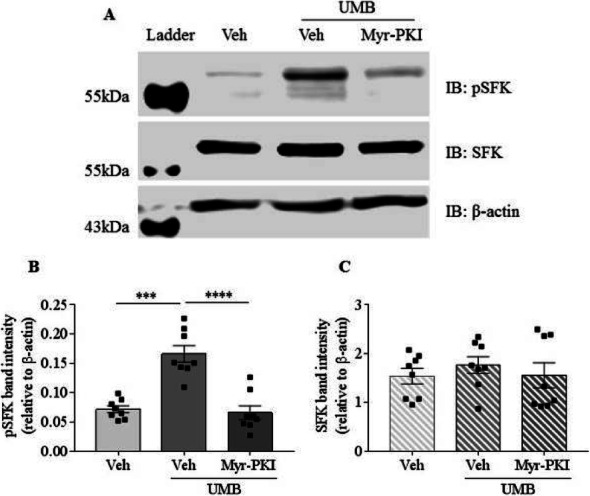
See text for description

**Fig. 2 (abstract P0256).**
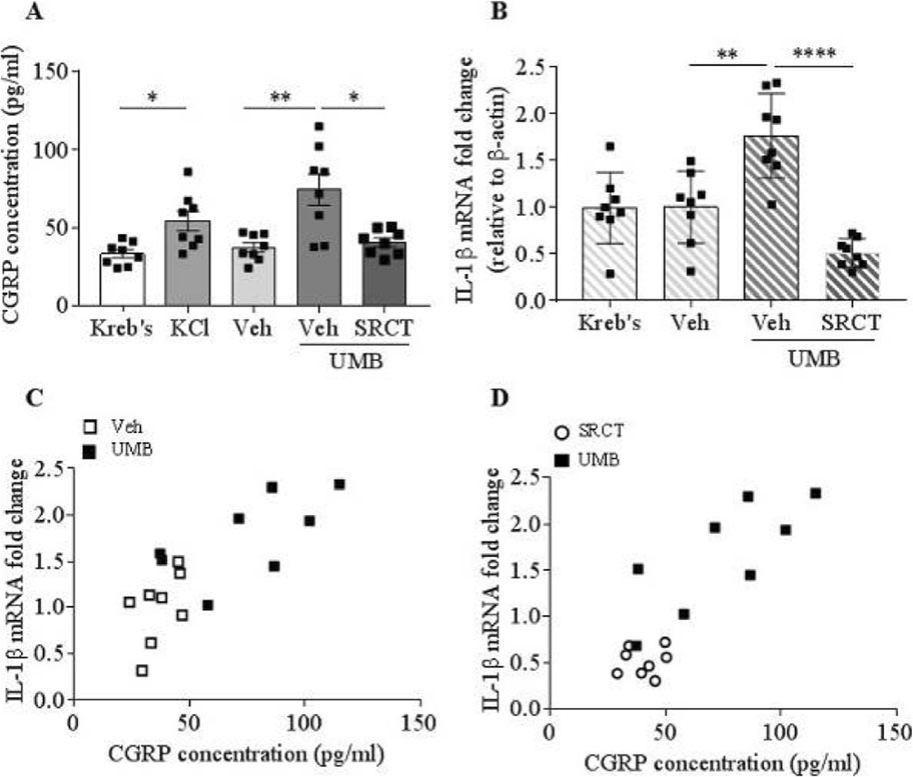
See text for description

## P0257 Is there a link between pain chronification, and allodynia and vitamin D deficiency in headache?

### C. Baiata^1,2^, V. Rebecchi^2^, L. Princiotta Cariddi^2,3^, D. Gallo^4,5^, M. Mauri^2,6^, M. Versino^2,5^

#### ^1^University of Milano Bicocca, Neurology and Stroke Unit, Monza, Italy; ^2^ASST Sette Laghi di Varese - Ospedale di Circolo Fondazione Macchi, Neurology and Stroke Unit, Varese, Italy; ^3^University of Insubria, Clinical and Experimental Medicine and Medical Humanities, Center of Research in Medical Pharmacology, Varese, Italy; ^4^University of Insubria, Endocrine Unit, Varese, Italy; ^5^University of Insubria, Medicine and Surgery, Varese, Italy; ^6^University of Insubria, Department of Biotechnologies and Life Sciences, Varese, Italy

##### **Correspondence:** C. Baiata

Objective**:** The vitamin D deficit has been associated to pain chronification, and chronic migraine is frequently associated with allodynia. The aim of this study was to assess the potential role of VitD in pain chronification and its relation to the occurrence of allodynia.

Methods: We recruited 76 consecutive patients: 32 belonged to the episodic migraine (EM), 34 to the chronic migraine and medication overuse (CM-MOH) groups and 10 to the tension-type headache (TTH) group. All patients underwent neurological and physical examination and anamnestic data collection including allodynia and serum calcifediol (25(OH)D) assessment.

Results**:** The occurrence of patients with vit D deficit was significantly higher in the CM-MOH (46%), than in the EM groups (25.7%) and in the TTH group (11.4%). The Vit D deficit was not significantly associated with any of the other variables. Allodynia also was more frequent in CM-MOH (66.7%) than in the EM (29.2%) and TTH groups (6.7%). On the contrary the occurrence of allodynia was independent from the vit D deficit (allodynia occurred in 42.4% of patients with and 57.6% without vit D deficit)

Conclusion: Prevalence of VitD deficiency and allodynia were significantly higher in patients suffering from CM-MOH, however the co-occurrence of allodynia and VitD deficiency were not correlated, thus suggesting that chronification and allodynia do not stem from the same pathophysiological mechanism.

## P0258 COmbining UbRogepAnt and preventives for miGrainE (COURAGE) study using the Migraine Buddy application: A novel, entirely remote design for collecting real-world evidence

### R. B. Lipton^1^, J. Contreras-De Lama^2^, D. Serrano^3^, E. Engstrom^3^, W. Poh^4^, F. Cadiou^4^, A. Adams^2^

#### ^1^Albert Einstein College of Medicine, Bronx, NY, United States; ^2^AbbVie, Irvine, CA, United States; ^3^Pharmerit International, Boston, MA, United States; ^4^Healtint Pte. Ltd, Singapore, Singapore

##### **Correspondence:** R. B. Lipton

To describe COURAGE, a novel, mobile (Migraine Buddy) app-based, study evaluating the real-world effectiveness of ubrogepant for the acute treatment of migraine when used with an approved preventive.

Eligible adults (≥3 ubrogepant-treated attacks, ≥3 migraine attacks in last 30 days) used ubrogepant with onabotulinumtoxinA (ub+obA), or with anti-CGRP monoclonal antibody (ub+mAb), or with both medication (ub+both). Over 30 days, participants reported treatment outcomes at <1, 1-2, 2-4, or >4 hrs post-ubrogepant. Interim marginal odds of achieving meaningful pain relief (MPR) for the 1st ubrogepant-treated attack by 2 and 4 hrs post dose were modeled via logistic regression. Covariates were age, MIDAS, ubrogepant dose.

As of 01/2021, 492 respondents consented, with 461 screened and then 354 enrolled; users with baseline treated ≥1 attack with ubrogepant) were ub+obtA, n=88 (83); ub+mAb, n=206 (175); ub+both, n=60 (51). 237 completed the study and 177 logged ≥3 ubrogepant-treated attacks. Interim data suggests many patients achieving MPR by 2 hrs post-treatment with a larger majority achieving MPR at 4 hrs. Adjusted odds were significant (p<0.001) for ub+obA and ub+mAb.

COURAGE has successfully assessed treatment value and usage patterns remotely to keep patients and HCP safe during the COVID-19 pandemic. Interim findings suggest that ubrogepant is effective when used with approved migraine preventives; final data may inform clinicians how best to optimize treatment.

## P0259 Real-World Efficacy, Tolerability and Safety of Ubrogepant

### C. C. Chiang^1^, K. Arca^2^, R. Dunn^2^, M. Girardo^3^, J. Quillen^3^, D. W. Dodick^2^, A. Starling^2^

#### ^1^Mayo Clinic, Neurology, Rochester, MN, United States; ^2^Mayo Clinic, Neurology, Scottsdale, AZ, United States; ^3^Mayo Clinic, Biostatistics, Scottsdale, AZ, United States

##### **Correspondence:** C. C. Chiang

Objective: To assess the real-world efficacy, tolerability, and safety of ubrogepant in a tertiary headache center.

Method: This was a cohort study conducted at Mayo Clinic Arizona. All patients prescribed ubrogepant were tracked and contacted 1-3 months after the prescription to answer a list of standardized questions.

Results: We obtained eligible responses from 106 patients; 86.8% had chronic migraine. Complete headache freedom, and headache relief for ≥75% of all treated attacks at 2 hours after taking ubrogepant was achieved in 19.0% and 47.6% patients, respectively. 31.1% patients were being "very satisfied" with ubrogepant. Adverse events were reported in 39.6% patients, including fatigue 27.4%, dry mouth 7.5%, nausea 6.6%, constipation 4.7%, dizziness 2.8%, and others 6.6%. Predictive factors for being a "good responder" to ubrogepant included migraine with aura, episodic migraine, <5 prior unsuccessful preventive or acute treatments, successful responses to a CGRP monoclonal antibody and onabotulinumtoxinA. For the 62 (58.5%) patients concurrently using a CGRP monoclonal antibody, there was no difference in the "good responder" rate or adverse event rate compared to those who were not on a CGRP monoclonal antibody, though the rate of moderate adverse events was higher.

Conclusion: Our study confirms and extends the efficacy profile and tolerability of ubrogepant in a real-world tertiary headache clinic and identifies factors that may predict efficacy.

**Table 1 (abstract P0259).**
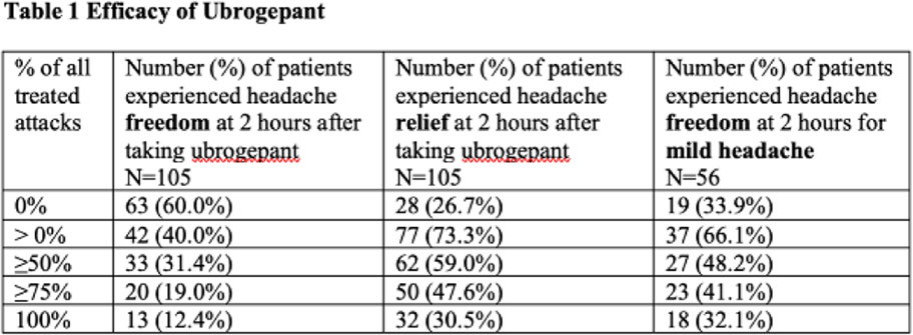
See text for description

**Table 2 (abstract P0259).**
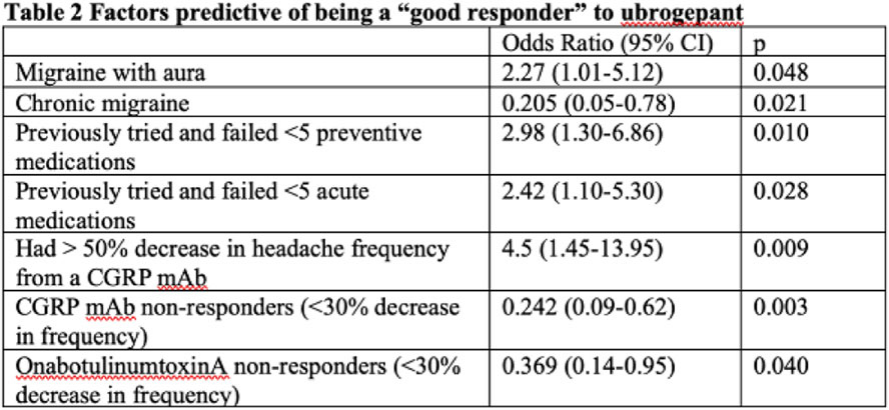
See text for description

## P0260 Adding sodium bicarbonate to bupivacaine in occipital nerve blocks improves injection related pain

### M. Sastre-Real^1^, J. Díaz de Terán Velasco^1^, C. Treviño-Peinado^2^

#### ^1^La Paz University Hospital, Neurology, Madrid, Spain; ^2^Severo Ochoa University Hospital, Neurology, Leganés, Spain

##### **Correspondence:** M. Sastre-Real

Objective: Our aim was to analyze if addition of sodium bicarbonate (SB) to bupivacaine for occipital nerve block (ONB) relieves injection related pain.

Methods: We included patients with a previous diagnosis of chronic migraine who received OnabutulinumtoxinA and ONB. We compared data from two University Hospitals where different compounds were injected according to their usual clinical practice: at site1 SB was added to bupivacaine and at site2 bupivacaine alone was used. The technique was otherwise similar. Pain during injection was assessed by a 1-10 analog scale.

Results: 51 patients were included (35 at site1 and 16 at site2). Patients from site1 suffered from more monthly migraine days (MMD) (11.4±7.4 vs 17.7±9.8; p=0.01) and less days from last migraine attack (1.7±4.3 vs 4.1±4.3, p=0.001). Patients in site1 reported less injection related pain in both sides (4.4±2.7 vs 6.5士1.3, p=0.005), in symptomatic side (4.2±2.9 vs 6.5±1.5, p=0.013) and in non-symptomatic side (3.3±2.4 vs 6.0±1.8, p=0.002). Tenderness to palpation was related to less painful injections only in right side. MMD, days from last attack and the presence of allodynia did not correlate with injection pain scores. We found no differences in patient reported improvement or number of headache days 1 week after injection between site1 and site 2.

Conclusion: The addition of SB to bupivacaine resulted in less painful injections in ONB. Well-designed studies are needed to confirm this finding.

## P0261 Impact of Prior Monthly Headache Days on Migraine-Related Quality of Life: Results From the CaMEO Study

### R. B. Lipton^1^, P. Pozo-Rosich^2,3^, S. L. Orr^4,5^, M. L. Reed^6^, K. M. Fanning^6^, B. Dabruzzo^7^, D. C. Buse^1^

#### ^1^Albert Einstein College of Medicine, Bronx, NY, United States; ^2^Vall d’Hebron University Hospital, Headache Unit, Neurology Department, Barcelona, Spain; ^3^Universitat Autonoma of Barcelona, Headache Research Group, VHIR, Barcelona, Spain; ^4^Alberta Children’s Hospital, University of Calgary, Calgary, AB, Canada; ^5^Cumming School of Medicine, University of Calgary, Calgary, AB, Canada; ^6^Vedanta Research, Chapel Hill, NC, United States; ^7^AbbVie, Madison, NJ, United States

##### **Correspondence:** R. B. Lipton

Objective: To examine the relationship between monthly headache days (MHDs) at cross-section and 3 months earlier on current Migraine-Specific Quality of Life Questionnaire (MSQ v2.1) scores.

Methods: CaMEO is a web-based, longitudinal study that identified US individuals who met migraine criteria consistent with the *International Classification of Headache Disorders-3*. Groups defined by MHD frequency at 3 and 6 months were established and mean MSQ Role Function–Restrictive (MSQ-RFR) scores calculated. MSQ-RFR scores at 6 months were modeled as the outcome in nested linear regression models examining 6- and 3-month MHDs.

Results: Among 16,789 migraine respondents, 6509 (38.8%) had valid MHD and MSQ-RFR data. At 6 months, 4640 (71.3%) respondents reported 0–3 MHDs, 896 (13.8%) reported 4–7 MHDs, 510 (7.8%) reported 8–14 MHDs, and 463 (7.1%) reported ≥15 MHDs. Across 6-month MHD categories, mean MSQ-RFR scores were 82.5, 61.5, 56.8, and 47.5 among those reporting 0–3, 4–7, 8–14, and ≥15 MHDs, respectively. Within each 6-month MHD group, mean 6-month MSQ-RFR showed a trend towards lower MSQ-RFR with higher 3-month (prior) MHD category. Linear regression showed that MSQ-RFR at 6 months was significantly associated with MHD frequency at 6 and 3 months (P=0.001 for each).

Conclusion: Our results demonstrate an inverse relationship between MSQ-RFR score at 6 months and MHD frequency at both 6 months and 3 months. Improvements in MSQ-RFR scores may lag behind improvements in MHDs.

## P0262 A Novel Approach to Defining Success in the Acute Treatment of Migraine: Pooled Results From the ACHIEVE I and ACHIEVE II Trials

### C. Iaconangelo^1^, D. Serrano^2^, A. Manack Adams^3^, J. M. Trugman^4^, R. B. Lipton^5^

#### ^1^Pharmerit International, New York, NY, United States; ^2^Pharmerit International, Bethesda, MD, United States; ^3^AbbVie, Irvine, CA, United States; ^4^AbbVie, Madison, NJ, United States; ^5^Albert Einstein College of Medicine, Bronx, NY, United States

##### **Correspondence:** C. Iaconangelo

Objective: To evaluate an alternative method of characterizing success in clinical trials for the acute treatment of migraine.

Methods: Pooled data for placebo and ubrogepant 50 mg (ACHIEVE I and ACHIEVE II trials) and data for ubrogepant 100 mg (ACHIEVE I) were used for this analysis. To define treatment success, we used confirmatory latent class modeling (LCM) that included inputs at baseline and 2 hours for pain severity and functional disability, and binary measures of nausea, photophobia, and phonophobia. Treatment success rates and predictive validity (using satisfaction with study medications [SWSM] 24 hours post-dose) with LCM were compared with 2-hour pain freedom (2hPF).

Results: LCM-based treatment success rates were 53.2% for ubrogepant 50 mg, 54.9% for ubrogepant 100 mg, and 39.0% for placebo, yielding a placebo-corrected difference of 14.2% (*P*<0.001) for the 50 mg dose and 15.9% (*P*<0.001) for the 100 mg dose. The LCM approach estimated higher rates of treatment success and larger placebo-corrected differences than with 2hPF. Using SWSM as the gold standard, sensitivity (0.72 vs 0.31) and Youden"s index (0.44 vs 0.28) were higher with LCM than for 2hPF.

Conclusion: The LCM approach more sensitively predicted treatment satisfaction at 24 hours and better aligned with our clinical understanding of migraine as a symptom complex. In contrast, 2hPF failed to capture a substantial proportion of patients satisfied with treatment.

## P0263 A Novel Approach to Defining Success in the Acute Treatment of Migraine: Demonstrating Therapeutic Benefit at 1 Hour Post-dose in the Pooled ACHIEVE I and ACHIEVE II Trials

### C. Iaconangelo^1^, D. Serrano^2^, A. Manack Adams^3^, J. M. Trugman^4^, R. B. Lipton^5^

#### ^1^Pharmerit International, New York, NY, United States; ^2^Pharmerit International, Bethesda, MD, United States; ^3^AbbVie, Irvine, CA, United States; ^4^AbbVie, Madison, NJ, United States; ^5^Albert Einstein College of Medicine, Bronx, NY, United States

##### **Correspondence:** C. Iaconangelo

Objective: To evaluate an alternative method of characterizing early treatment success (1-hour post-dose) in clinical trials for the acute treatment of migraine.

Methods: Pooled data for placebo and ubrogepant 50mg (ACHIEVE I and ACHIEVE II trials) and data for ubrogepant 100mg (ACHIEVE I) were used for this analysis. To define treatment success, we used confirmatory latent class modeling (LCM) that included inputs at baseline and 1 hour for pain severity and functional disability, and binary measures of nausea, photophobia, and phonophobia. Treatment success rates and predictive validity (using satisfaction with study medications [SWSM] 24 hours post-dose) with LCM were compared with 1-hour pain freedom (1hPF).

Results: Treatment success rates based on LCM were 34.3% for ubrogepant 50mg, 34.2% for ubrogepant 100mg, and 25.9% for placebo, yielding a placebo-corrected difference of 8.4% (*P*<0.001) for the 50mg dose and 8.3% (*P*<0.001) for the 100mg dose. In comparison, the 1hPF endpoint estimated very low rates with no differences between active treatment and placebo. Using SWSM as the gold standard, sensitivity (0.46 vs 0.07) and Youden"s index (0.26 vs 0.06) were higher for LCM than for 1hPF.

Conclusion: The LCM approach was a more sensitive predictor of treatment satisfaction at 24 hours and better aligned with our clinical understanding of migraine as a symptom complex. Compared with LCM, 1hPF failed to capture a substantial proportion of people satisfied with treatment.

## P0264 DHE Pharmacology revisited: Does a broad receptor profile molecule treat the whole migraine?

### S. Aurora^1^, S. RAY^1^, K. Satterly^2^, L. McConnachie^2^, S. Shrewsbury^1^, J. Hoekman^3^, P. J. Goadsby^4^

#### ^1^Impel NeuroPharma, Medical Affairs, Seattle, WA, United States; ^2^Impel NeuroPharma, Analytical and Translational Sciences, Seattle, WA, United States; ^3^Impel NeuroPharma, Research & Development, Seattle, WA, United States; ^4^University of California, Los Angeles, Neurology, Los Angeles, CA, United States

##### **Correspondence:** S. Aurora

Objectives: Migraine is a complex neurological disorder, however, therapeutics have focused on targeting a relatively narrow set of receptors i.e. 5HT1B/1D/F or CGRP. Comparative receptor pharmacology of various acute therapies for migraine were examined.

Methods: Following a literature review, additional, functional receptor activity of DHE was screened against 170 G-protein coupled receptors.

Results: DHE mesylate (10 μM) exhibited *agonist* activity at: Adrenoceptor α2B, CXCR7, Dopamine D2, D5, 5HT1A/1B/2A/2C/5A, binding with high affinity to the 5HT1B, Adrenoreceptor α2B, Dopamine D2receptors and exhibited *antagonist* activity at: Adrenoceptor a1B, a2A, a2C, CALCR-RAMP2, Dopamine D1, D3, D4, D5 and 5HT1F. Further work showed DHE did not bind to the 5HT3 receptor and did so in a limited capacity to the 5HT4E receptor, at concentrations up to 300 nM. Comparative receptor binding of migraine specific therapies is presented in tabular format. A model was created to show where in migraine progression each acute migraine specific therapeutic acts to address migraine symptoms.

Conclusion: DHE interacts with several different receptor subtypes. Unlike other migraine therapeutics, it may exert a wider influence over the pathophysiology of the migraine. Moreover, the slow dissociation of DHE from target receptors is thought to sustain its anti-migraine effects, extending duration of benefit, reducing headache recurrence rates and, perhaps, medication overuse headache.

## P0265 Efficacy of Lasmiditan for the Acute Treatment of Perimenstrual Migraine

### A. MacGregor^1^, M. Komori^2^, J. Krege^3^, Y. Dong^3^, M. Vincent^3^, P. Hauck^3^, H. Igarashi^4^

#### ^1^Centre for Reproductive Medicine, St. Bartholomew’s Hospital, London, United Kingdom; ^2^Eli Lilly and Company, Kobe, Japan; ^3^Eli Lilly and Company, Indiana, IN, United States; ^4^Department of Internal Medicine, Headache Care Unit, Fujitsu Clinic, Kawasaki, Japan

##### **Correspondence:** A. MacGregor

Objective: Evaluating lasmiditan's (LTN's) efficacy in perimenstrual migraine in women.

Methods: Data from randomized, double-blind, placebo-controlled Phase 2 MONONOFU (N=78) and Phase 3 CENTURION (N=225) LTN trials were pooled. Attacks were treated within 4 hours(h) of pain onset provided the headache severity was moderate/severe. Perimenstrual attack was defined as attack that was treated at any time from Day −2 to Day +3 of menstruation. Data were from each patient's 1st treated perimenstrual migraine. Logistic regression model with treatment group and region as covariates was used to evaluate efficacy. Patients with missing outcome data were imputed as nonresponders.

Results: 303 perimenstrual migraine attacks were treated (50mg [N=24]/100mg [N=90]/200mg [N=110]/placebo [N=79]). A greater proportion of patients achieved head pain freedom with LTN 200mg vs placebo at all time points assessed (Fig.) with significance starting at 1h [10.9%,p=0.04] vs placebo-treated patients [2.5%]. At 2h, 33.6% of patients in 200mg group (p<0.001); 16.7% of both 100mg (p=0.11) and 50mg (p=0.19) groups were pain free vs 7.6% with placebo. More patients treated with LTN100mg/200mg experienced no interference with normal activities at/after 2h and freedom from most bothersome migraine-associated symptoms at all time points vs placebo.

Conclusion: LTN resulted in freedom from perimenstrual migraine-related head pain, most bothersome symptoms and interference with normal activities.

**Fig. 1 (abstract P0265).**
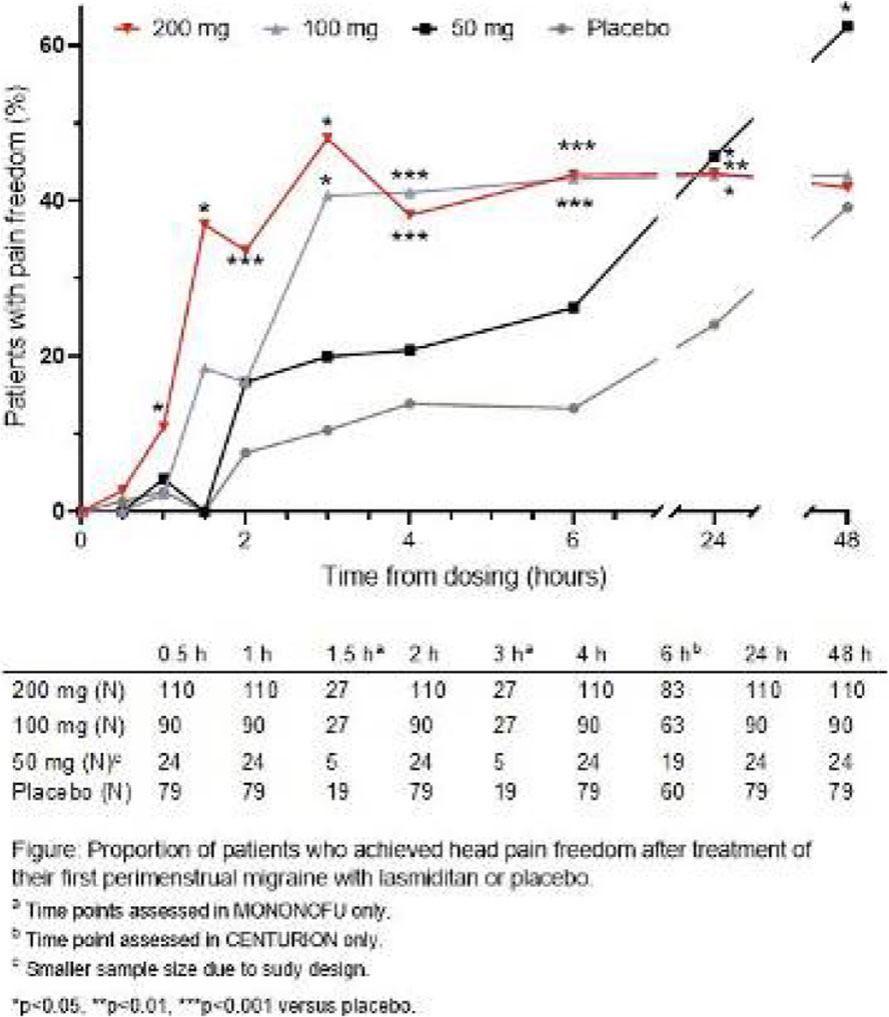
See text for description

## P0266 Oral Rimegepant 75 mg is Safe and Well Tolerated in Adults With Migraine and Cardiovascular Risk Factors: Results of a Multicenter, Long-Term, Open-Label Safety Study

### S. Hutchinson^1^, J. D. Schim^2^, R. B. Lipton^3^, R. Croop^4^, C. M. Jensen^4^, A. Thiry^4^, E. G. Stock^4^, C. M. Conway^4^, M. Lovegren^4^, V. Coric^4^, M. Hanna^4^

#### ^1^Orange County Migraine & Headache Center, Irvine, CA, United States; ^2^Headache Center of Southern California, Carlsbad, CA, United States; ^3^Albert Einstein College of Medicine, Bronx, NY, United States; ^4^Biohaven Pharmaceuticals, New Haven, CT, United States

##### **Correspondence:** S. Hutchinson

Objective: Evaluate the safety and tolerability of rimegepant in adults with cardiovascular (CV) risk factors.

Methods: Multicenter, long-term, open-label safety study (NCT03266588) in adults with a history of 2-14 monthly migraine attacks of moderate to severe pain intensity. Subjects used rimegepant 75 mg up to once daily for up to 52 weeks. For this analysis, subjects were organized into subgroups by number of baseline CV risk factors (0, 1, ≥2) and Framingham 10-year risk of developing a CV condition (low = <10%, moderate to high = ≥10%).

Results: Of the 1800 rimegepant-treated subjects, 735 (40.8%) had CV risk factors (518 [28.8%] had 1 and 217 [12.1%] had ≥2]) and 126 (7.0%) had a moderate to high risk 10-year CV risk. The most common adverse events (AEs) regardless of relationship to treatment were upper respiratory tract infection (8.8%), nasopharyngitis (6.8%), and sinusitis (5.1%), and the proportion of subjects reporting ≥1 AE was similar across all subgroups (Table 1). No serious AEs were considered by the investigator to be related to rimegepant. Only 1 subject out of 1800, a 53 year-old male with a history of CV disease (angina pectoris), experienced an ischemic Cardiac Disorder SOC AE (angina pectoris) deemed by the investigator to be not related to rimegepant.

Conclusion: Rimegepant dosed up to once daily for up to 1 year showed favorable safety and tolerability in adults with migraine with CV risk factors, including adults with moderate to high CV risk.

**Table 1 (abstract P0266).**
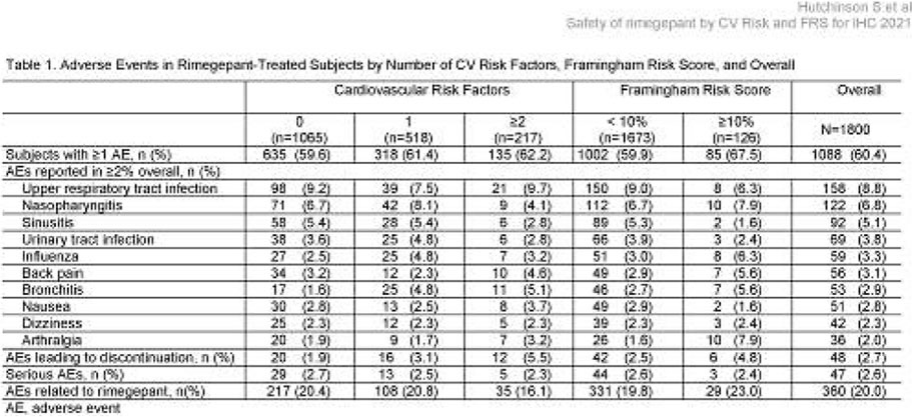
See text for description

## P0267 Intranasal Zavegepant is Effective and Well Tolerated for the Acute Treatment of Migraine: A Phase 2/3 Dose-Ranging Clinical Trial

### R. B. Lipton^1^, J. Madonia^2^, C. M. Conway^2^, A. Thiry^2^, M. Forshaw^2^, A. Murphy^2^, C. M. Jensen^2^, G. Dubowchik^2^, V. Coric^2^, R. Croop^2^

#### ^1^Albert Einstein College of Medicine, Bronx, NY, United States; ^2^Biohaven Pharmaceuticals, New Haven, CT, United States

##### **Correspondence:** R. B. Lipton

Objective: Evaluate the efficacy, safety, and tolerability of intranasal zavegepant — a third-generation, high-affinity, selective and structurally unique, small molecule CGRP receptor antagonist — in the acute treatment of migraine.

Methods: In this randomized, dose-ranging, placebo-controlled, Phase 2/3 trial (NCT03872453), adults with migraine treated 1 attack of moderate to severe pain intensity with intranasal zavegepant 5, 10, 20 mg, or placebo. Coprimary efficacy endpoints were pain freedom and freedom from the most bothersome symptom (MBS; ie, photophobia, phonophobia, or nausea) at 2 hours postdose. Endpoints were tested hierarchically at an alpha level of 0.0167.

Results: In total 1581 subjects (median age 40 years, 85.5% female, ~14% taking preventive migraine medication) were in the modified intention-to-treat population [zavegepant 5 mg (n=387), 10 mg (n=391), 20 mg (n=402), placebo (n=401)]. On the coprimary endpoints (Table 1), zavegepant 10 mg and 20 mg were superior to placebo. Figure 1 shows pain relief rates through 2 hours postdose for all zavegepant dose strengths. The most common (>5%) adverse events (AEs) with zavegepant were dysgeusia (13.5%-16.1% vs 3.5% with placebo) and nasal discomfort (1.3%-5.2% vs 0.2% with placebo). The majority of AEs were mild or moderate. There was no signal of hepatoxicity.

Conclusion: Intranasal zavegepant 10 mg and 20 mg were effective for the acute treatment of migraine, with a favorable safety profile.

**Table 1 (abstract P0267).**
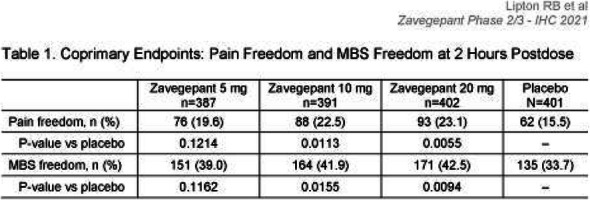
See text for description

**Fig. 1 (abstract P0267).**
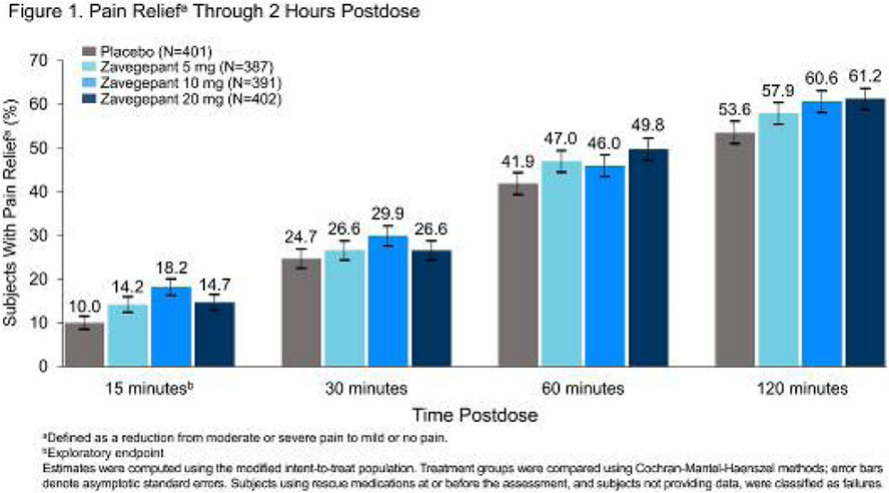
See text for description

## P0268 Rimegepant for the Acute Treatment of Migraine: Subgroup Analyses From 3 Phase 3 Clinical Trials by Number of Triptans Previously Tried and Failed

### C. M. Jensen^1^, R. B. Lipton^2^, A. M. Blumenfeld^3^, R. Croop^1^, A. Thiry^1^, G. L'Italien^1^, B. Morris^1^, V. Coric^1^, P. J. Goadsby^4,5^

#### ^1^Biohaven Pharmaceuticals, New Haven, CT, United States; ^2^Albert Einstein College of Medicine, Bronx, NY, United States; ^3^Headache Center of Southern California, Carlsbad, CA, United States; ^4^NIHR-Wellcome Trust King’s Clinical Research Facility, King’s College Hospital/SLaM Biomedical Research Centre, King’s College, London, Germany; ^5^University of California, Los Angeles, Neurology, Los Angeles, CA, United States

##### **Correspondence:** C. M. Jensen

Objective: Assess the efficacy of rimegepant — an oral small molecule calcitonin gene-related peptide receptor antagonist — for the acute treatment of migraine in subjects with and without a history of triptan treatment failure.

Methods: Three double-blind, placebo-controlled trials of similar design randomized adults with migraine to rimegepant 75 mg tablet (NCT03235479, NCT03237845) or ODT (NCT03461757) or placebo to treat 1 migraine attack of moderate to severe pain intensity. Subgroups with a history of treatment failure with 1 or ≥2 triptans and those without a history of triptan failure, including triptan-naïve and current triptan users, were analyzed. Triptan treatment failure was defined as self-reporting a history of discontinuing ≥1 triptan due to inadequate efficacy and/or poor tolerability. The coprimary endpoints were 2-hour freedom from pain and the most bothersome symptom (MBS).

Results: In the pooled population (N=3507: rimegepant n=1749, placebo n=1758), 2272 (64.8%) subjects had no history of triptan treatment failure and 1235 (35.2%) had a history of treatment failure with ≥1 triptan. Results for the coprimary endpoints in each triptan subgroup are shown in Figure 1. No differences in coprimary endpoints were found in pairwise comparisons of triptan subgroups in rimegepant-treated subjects (Table 1).

Conclusions: Rimegepant was effective for the acute treatment of migraine in subjects with and without a history of triptan treatment failure.

**Fig. 1 (abstract P0268).**
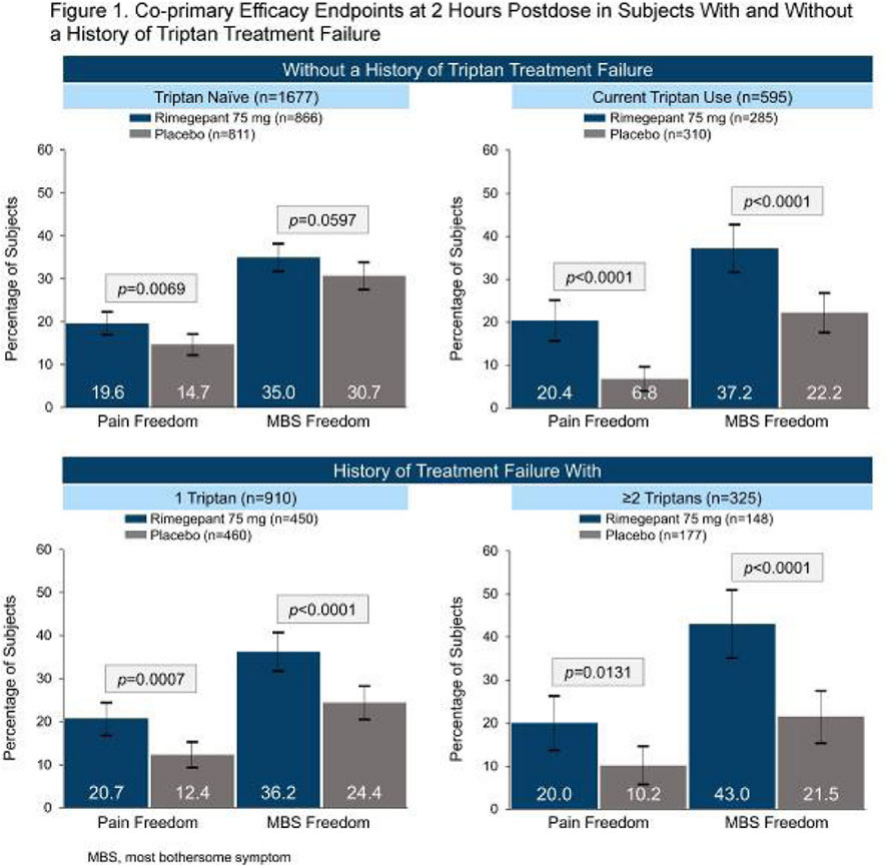
See text for description

**Table 1 (abstract P0268).**
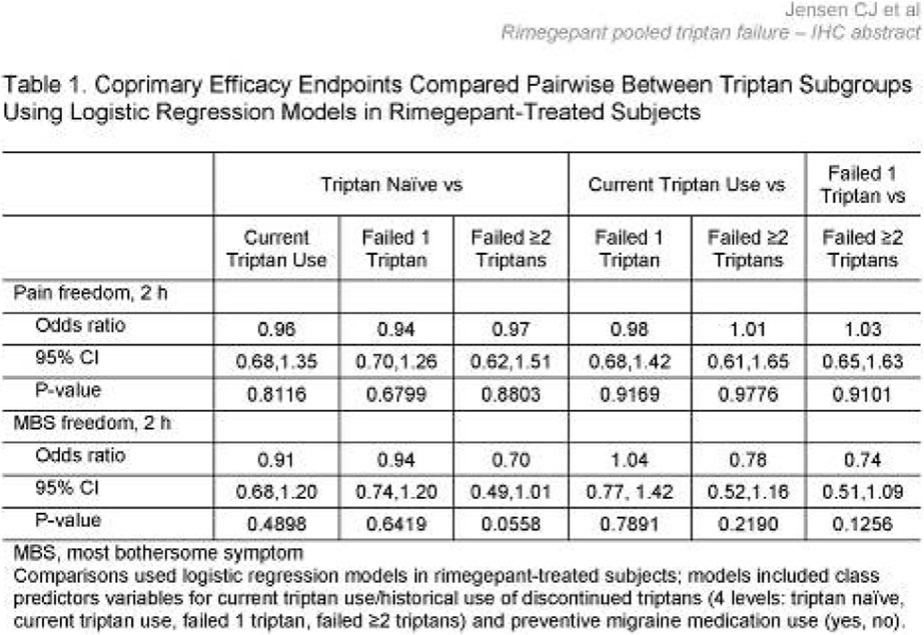
See text for description

## P0269 Acute Treatment with Oral Rimegepant 75 mg Reduces Migraine-Related Disability in Adults With and Without a History of Triptan Treatment Failure: Results from a One Year, Open-Label Safety Study

### R. B. Lipton^1^, C. M. Jensen^2^, A. Thiry^2^, E. Kim^2^, M. Lovegren^2^, R. Croop^2^, V. Coric^2^, G. L'Italien^2^

#### ^1^Albert Einstein College of Medicine, Bronx, NY, United States; ^2^Biohaven Pharmaceuticals, New Haven, CT, United States

##### **Correspondence:** R. B. Lipton; C. M. Jensen

Objective: Assess the effects of rimegepant, an oral small molecule CGRP receptor antagonist, on migraine-related disability in adults with and without a history of triptan treatment failure.

Methods: Long-term, open-label safety study (NCT03266588) of adults with a history of 2-14 moderate to severe monthly migraine attacks. Rimegepant was dosed as needed (PRN) for 52 weeks or every other day plus PRN on nonscheduled dosing days for 12 weeks (EOD+PRN). The Migraine Disability Assessment (MIDAS) was given at baseline and Weeks 12, 24, 36, and 52; disability was scored as 0-5 (little or no), 6-10 (mild), 11-20 (moderate), and ≥21 (severe). Subgroups with no history of triptan failure (including triptan naive and current triptan users) and a history of failure with 1 or ≥2 triptans were assessed. Triptan failure was defined as having a history of discontinuing ≥1 triptan due to inadequate efficacy and/or poor tolerability.

Results: Of the 1800 subjects, 546 (30.3%) had 1 triptan failure, and 246 (13.7%) had ≥2 triptan failures. Baseline mean (SD) MIDAS total scores showed severe disability: 0 triptan failures 32.8 (33.1); 1 triptan failure 34.5 (31.8); and ≥2 triptan failures 36.9 (32.0). Changes from baseline in MIDAS total scores exceeded the clinically important difference threshold at all time points for all 3 subgroups (Figure 1).

Conclusions: Rimegepant 75 mg reduced migraine-related disability versus baseline regardless of prior history of triptan treatment failure.

**Fig. 1 (abstract P0269).**
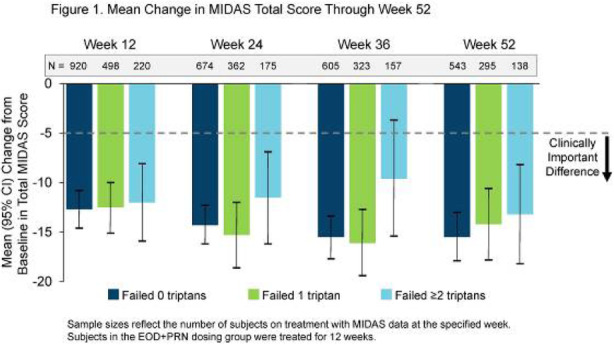
See text for description

## P0271 Long-term Use of Rimegepant 75 mg for the Acute Treatment of Migraine Reduces Use of Analgesics and Antiemetics

### D. Kudrow^1^, K. Mullin^2^, G. Berman^3^, R. B. Lipton^4^, A. Thiry^5^, R. Croop^5^, M. Lovegren^5^, V. Coric^5^, C. M. Jensen^5^

#### ^1^California Medical Clinic for Headache, Santa Monica, CA, United States; ^2^New England Institute for Neurology and Headache, Stamford, CT, United States; ^3^Clinical Research Institute, Minneapolis, MN, United States; ^4^Albert Einstein College of Medicine, Bronx, NY, United States; ^5^Biohaven Pharmaceuticals, New Haven, CT, United States

##### **Correspondence:** D. Kudrow

Objective: Determine if rimegepant treatment over time reduces the use of analgesics and antiemetics in adults with migraine.

Methods: This long-term, open-label safety study (NCT03266588) included adults with a history of 2-14 moderate-severe monthly migraine attacks who took rimegepant 75 mg (1) up to once daily as needed (PRN) for 52 weeks to treat attacks of any pain intensity or (2) every other day plus PRN (QOD+PRN) for 12 weeks. Subjects could take standard of care analgesic and antiemetic medications for migraine if needed. Use of analgesics and antiemetics was analyzed during the 30-day observation period and during rimegepant long-term treatment.

Results: Of the 1800 subjects treated (PRN [n=1514], QOD+PRN [n=286]), 89.4% were female, and mean age was 43 years. The most commonly used analgesics and antiemetics are shown in Table 1. The percentage of subjects with a 100% reduction in select analgesic and antiemetic use consistently increased during Weeks 1-4, Weeks 5-8, and Weeks 9-12 of rimegepant PRN and QOD+PRN treatment (Figure 1). During Weeks 49-52 of PRN treatment, 61.3% (95% CI: 57.8, 64.6) of subjects had a 100% reduction in select analgesic and antiemetic use.

Conclusions: As needed dosing and scheduled every other day dosing of oral rimegepant 75 mg was associated with significant reductions in analgesic and antiemetic use in adults with migraine.

**Table 1 (abstract P0271).**
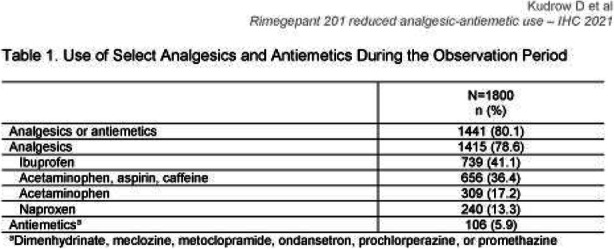
See text for description

**Fig. 1 (abstract P0271).**
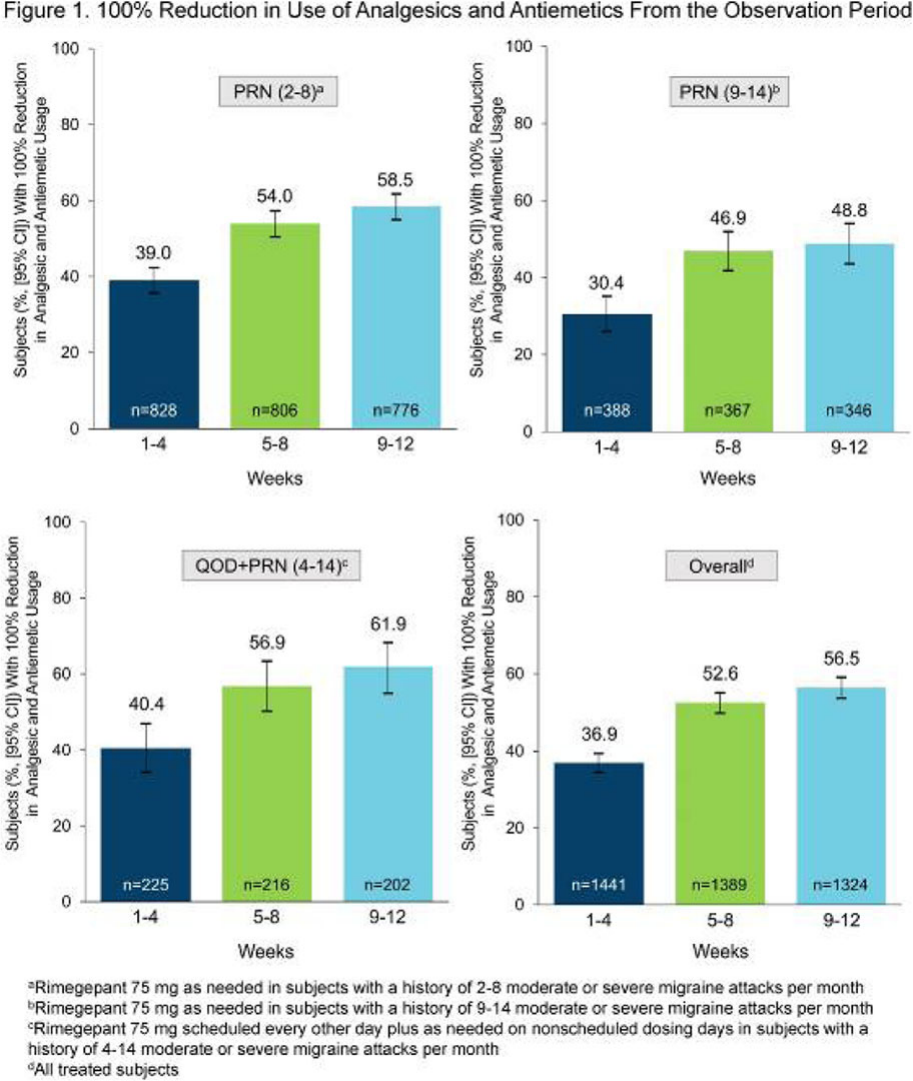
See text for description

## P0272 Acute Treatment of Migraine With Rimegepant Improves Health Related Quality of Life in Adults With a History of Triptan Treatment Failure: Results from a Long-Term, Open-Label Safety Study

### I. Turner^1^, P. Winner^2^, R. B. Lipton^3^, C. M. Jensen^4^, G. L'Italien^4^, A. Thiry^4^, M. Lovegren^4^, C. M. Conway^4^, V. Coric^4^, R. Croop^4^

#### ^1^Island Neurological Associates, Plainview, NY, United States; ^2^Palm Beach Neurology, West Palm Beach FL, United States; ^3^Albert Einstein College of Medicine, Bronx, NY, United States; ^4^Biohaven Pharmaceuticals, New Haven, CT, United States

##### **Correspondence:** I. Turner

Objective: Assess the effects of rimegepant on migraine-specific quality of life (MSQoL) in adults with migraine with a history of triptan treatment failure.

Methods: In a long-term, open-label safety study (NCT03266588), adults with a history of 2-14 moderate-severe monthly migraine attacks used rimegepant 75 mg up to once daily for up to 52 weeks. The migraine-specific quality of life questionnaire (MSQv2) was administered at baseline and weeks 12, 24, 36, and 52; domains include Role-Restrictive (RR), Role Preventative (RP), and Emotional Function (EF). Raw total scores were rescaled from 0-100; higher scores indicate better quality of life. This post-hoc analysis assessed MSQv2 in subgroups with a history of discontinuing 1 or ≥2 triptans due to inadequate efficacy or poor tolerability (i.e., treatment failure).

Results: Respective mean baseline MSQv2 scores for RR, RP, and EF were 53.6, 69.4, and 62.5 among subjects with a history of 1 triptan treatment failure (n=546) and 51.5, 66.7, and 56.2 among subjects with ≥2 triptan treatment failures (n=246). Both subgroups showed positive mean (95% CI) changes from baseline on all MSQv2 domains beginning at week 12 and continuing through week 52 (Figure 1). The effect of selective attrition cannot be assessed.

Conclusions: Long-term treatment with rimegepant was associated with improved MSQoL among adults with migraine and a history of triptan treatment failure, regardless of the number of triptans previously tried and failed.

**Fig. 1 (abstract P0272).**
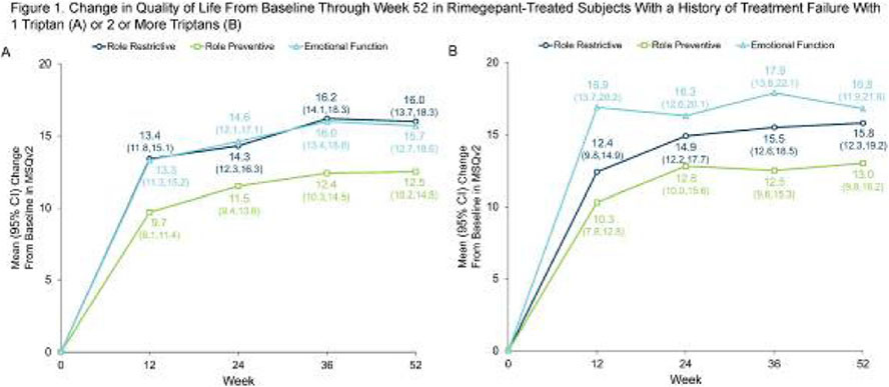
See text for description

## P0273 Effect of established vestibular dysfunction on migraine transformation

### I. Maryenko, S. Likhachev, M. Mozheiko

#### Republican research and clinical centre of neurology and neurosurgery, Neurological, Minsk, Belarus

##### **Correspondence:** I. Maryenko; M. Mozheiko

Introduction: Combination of migraine (MG) and vertigo (VG) is quite common in the population, many patients with MG may show signs of impaired vestibular function.

Objective: To evaluate the effects of established vestibular dysfunction (VD) on the course of MG.

Materials and methods: 40 patients (34 w and 6 m, mean age 37.5±18.2) with MG (ICHD, 2013) examined. Pain syndrome assessed by "PainDETECT" questionnaire, VG type determined according to the anamnesis. Spontaneous and provocative nystagmus recorded by electronistagmography.

Results: Sudden systemic VG paroxysms 1(3.3%), paroxysms of systemic VG in turning head -10(33.3%), non-systemic VG 7(28.0%), vestibulovegetative complaints by the type of motion sickness 18(60%) cases and paroxysms of systemic positional VG 15(50.0%) established significantly more often (p<0.05). Spontaneous nystagmus not registered in group. Provocative nystagmus detected in the Dix-Hallpike test in 25(62.5%) cases. The "PainDETECT" revealed in 28(70%) patients high probability of developing neuropathic pain component (19-41 points) (χ2=27.25, p=0.00001), in 4(10%) patients possible presence of neuropathic pain component established.

Conclusions: VD in MG increases pain perception found. It increases risk of transition from episodic to chronic migraine with the formation of neuropathic pain syndrome. Early treatment of VD will effectively affect VG syndrome, accelerate the recovery of impaired functions, reduce risk of episodic migraine becoming chronic.

## P0275 Patient Preference, Satisfaction, and Improved Clinical Global Impression of Change with Rimegepant 75 mg for the Acute Treatment of Migraine: Results from a Long-Term Open-Label Safety Study

### I. Turner^1^, J. M. Pavlovic^2^, R. B. Lipton^2^, R. Croop^3^, E. G. Stock^3^, A. Thiry^3^, C. M. Conway^3^, M. Lovegren^3^, V. Coric^3^, G. L'Italien^3^, C. M. Jensen^3^

#### ^1^Island Neurological Associates, Plainview, NY, United States; ^2^Albert Einstein College of Medicine, Bronx, NY, United States; ^3^Biohaven Pharmaceuticals, New Haven, CT, United States

##### **Correspondence:** I. Turner

Objectives: Assess preference of medication, satisfaction with medication, and Clinical Global Impression of Change (CGI-C) in participants using oral rimegepant, a small molecule CGRP receptor antagonist with demonstrated efficacy in the acute and preventive treatment of migraine.

Methods: Multicenter, long-term, open-label safety study (NCT03266588) enrolled adults with a history of 2-14 monthly migraine attacks of moderate to severe pain intensity. Subjects used rimegepant 75 mg as needed up to once daily to treat attacks of any pain intensity for up to 52 weeks. Preference of medication compared with previous acute treatments for migraine and satisfaction with medication were recorded by subjects via electronic diary; CGI-C was administered by clinicians.

Results: Overall, 1514 participants began the 52-week treatment period. At Week 24, 78.7% of subjects preferred rimegepant over their previous migraine medications, 89.4% of subjects were satisfied with rimegepant, and 88.8% of subjects were considered improved since study entry on the CGI-C scale. The percentages (95% CIs) of subjects at Week 52 who preferred rimegepant, were satisfied with rimegepant, and were considered improved since study entry are shown in Figure 1.

Among individuals using rimegepant as an acute treatment for 1 year, 4 in 5 preferred rimegepant to their previous migraine medications, 7 in 10 were satisfied withrimegepant, and 9 in 10 experienced clinical improvement relative to baseline.

**Fig. 1 (abstract P0275).**
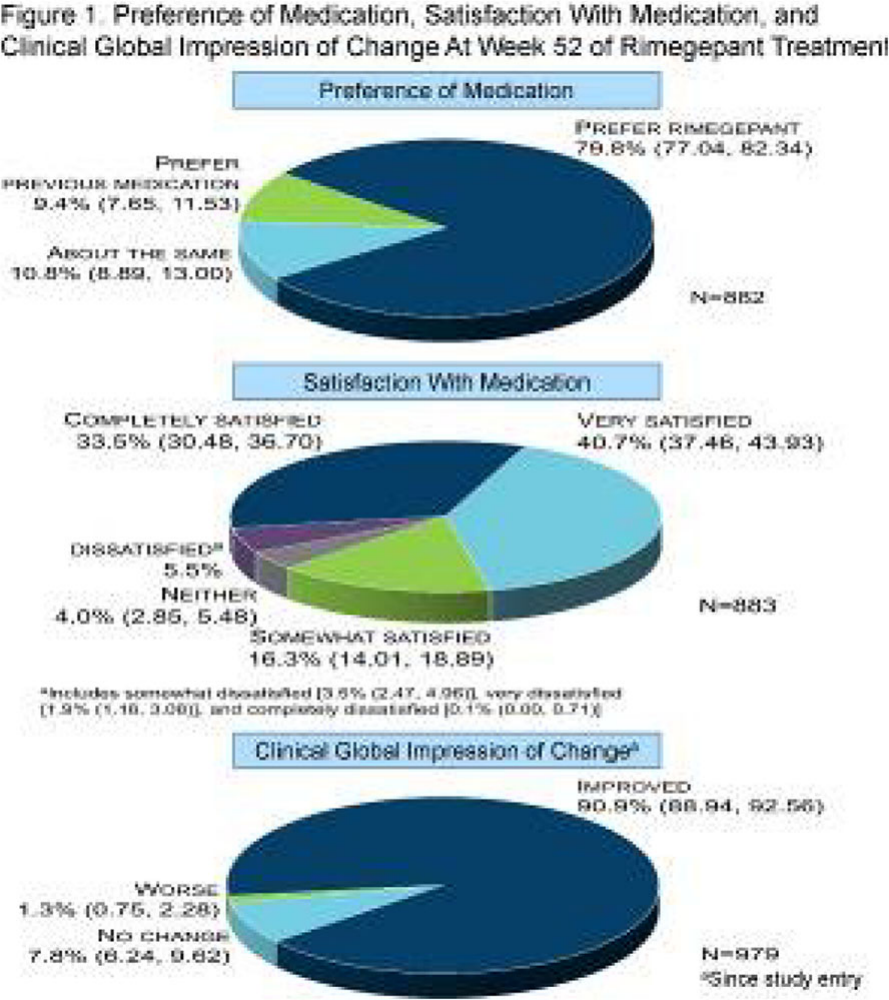
See text for description

## P0276 Efficacy and Safety of AXS-07 (MoSEIC Meloxicam-Rizatriptan) for the Acute Treatment of Migraine: Results from the INTERCEPT Phase 3, Randomized, Double-blind, Placebo-controlled Trial

### A. Jones^1^, C. O'Gorman^1^, R. B. Lipton^2^, S. J. Tepper^3^, H. Tabuteau^1^

#### ^1^Axsome Therapeutics, New York, NY, United States; ^2^Albert Einstein College of Medicine, New York, NY, United States; ^3^Geisel School of Medicine at Dartmouth, Hanover, NH, United States

##### **Correspondence:** A. Jones; C. O'Gorman

Background and objective: AXS-07 (MoSEIC™ Meloxicam-Rizatriptan) is a novel, oral, rapidly absorbed, multi-mechanistic investigational drug for the acute treatment of migraine. Meloxicam is a new molecular entity for migraine enabled by MoSEIC technology, resulting in rapid absorption while maintaining a long half-life. INTERCEPT, a Phase 3, randomized, double-blind, placebo (pbo)-controlled study, assessed the efficacy and safety of AXS-07 in the early acute treatment of migraine.

Methods: 302 patients were randomized (1:1) to take a single dose of AXS-07 or pbo at the earliest sign of pain, while mild.

Results: AXS-07 met the two co-primary endpoints: a statistically significantly greater percentage of patients vs. pbo achieved freedom from pain (32.6% vs. 16.3%, p=0.002) and most bothersome symptom (43.9% vs. 26.7%, p=0.003), 2 hours after dosing. AXS-07 rapidly eliminated migraine pain compared to pbo, with numerical separation by 30 mins, and statistical significance at 90 mins (p=0.003) and all timepoints thereafter. AXS-07 significantly prevented pain progression in 73.5% of AXS-07 patients compared to 47.4% for pbo (p<0.001) and significantly reduced rescue med use through 24hrs (15.3% of AXS-07 patients compared to 42.2% of pbo patients, p<0.001). 73.5% of AXS-07 patients returned to normal functioning at 24hrs vs. 47.4% for pbo (p<0.001).

Conclusions: Treatment with AXS-07 substantially and significantly eliminated pain and prevented pain progression vs. placebo.

## P0278 Rimegepant 75 mg for the Acute Treatment of Migraine in Adults With Frequent Migraine: Long-Term Safety and Clinical Improvement Versus Baseline

### K. Mullin^1^, S. Hutchinson^2^, T. Smith^3^, C. M. Jensen^4^, C. Leroue^4^, A. Thiry^4^, M. Lovegren^4^, C. M. Conway^4^, V. Coric^4^, R. Croop^4^

#### ^1^New England Institute for Neurology and Headache, Stamford, CT, United States; ^2^Orange County Migraine & Headache Center, Irvine, CA, United States; ^3^StudyMetrix Research, St Louis, MO, United States; ^4^Biohaven Pharmaceuticals, New Haven, CT, United States

##### **Correspondence:** K. Mullin

Objective: Assess long-term safety and clinical improvement with rimegepant – an oral small molecule CGRP receptor antagonist with demonstrated efficacy in acute and preventive treatment of migraine – in adults with frequent migraine attacks.

Methods: Open-label safety study of adults with 2-14 monthly migraine attacks of moderate-severe intensity. Subjects with chronic migraine were allowed; there were no limitations on the number of monthly migraine or non-migraine headache days. A 30-day observation period (OP) was followed by long-term treatment with rimegepant 75 mg orally up to once daily for up to 52 weeks. Migraine days were captured via electronic diary. This post-hoc analysis assessed safety and clinical global impression of change (CGI-C) in subjects experiencing ≥15 migraine days per 30 days in the OP.

Results: In total, 13.7% (246/1800) of subjects had ≥15 migraine days per 30 days in the OP. In this subgroup, the most common adverse events (AEs) were nasopharyngitis (8.5%), sinusitis (6.1%), and upper respiratory tract infection (5.3%); 4.9% of subjects discontinued due to an AE; and 3.7% had a serious AE, none of which were related to rimegepant. Percentages (95% CI) of subjects who were improved since study entry on the CGI-C scale, were 89.2% (83.3, 93.2) and 87.5% (80.0, 92.5) at week 24 (n=157) and week 52 (n=112), respectively (Figure 1).

Conclusion: Rimegepant was well-tolerated and associated with clinical improvement in adults with frequent migraine.

**Fig. 1 (abstract P0278).**
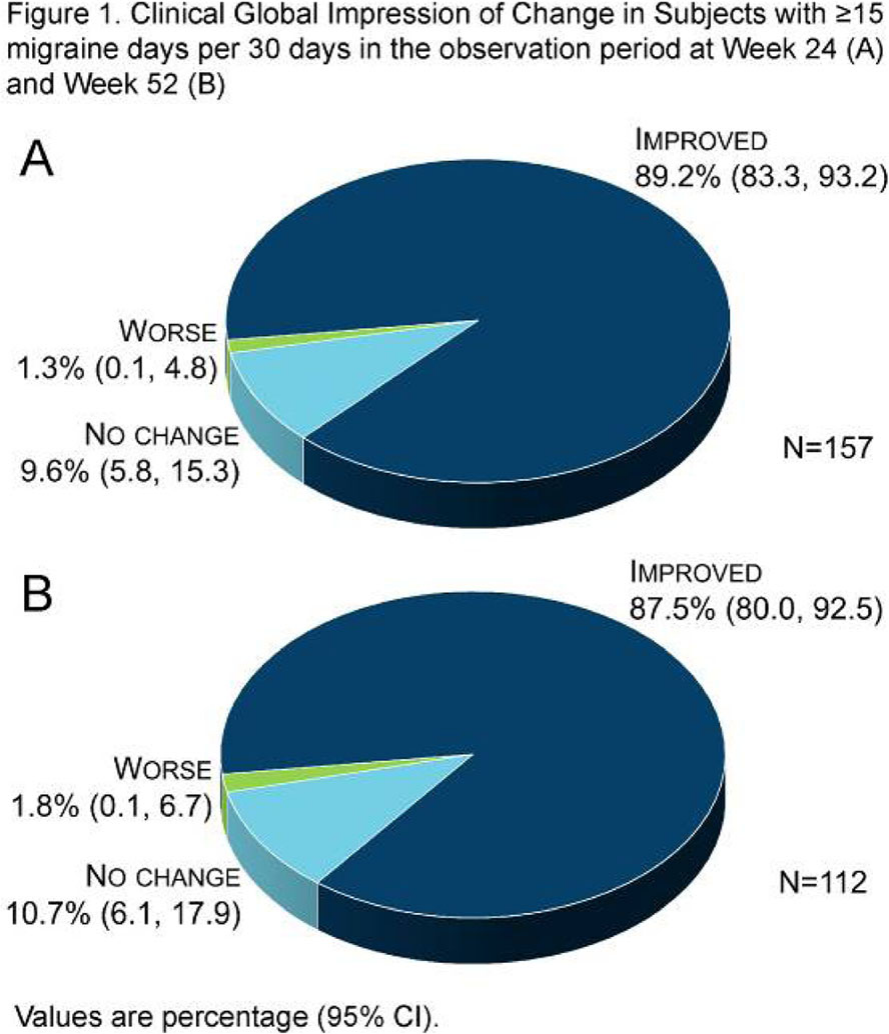
See text for description

## P0279 Long-Term Efficacy and Safety of AXS-07 (MoSEIC Meloxicam-Rizatriptan) for the Acute Treatment of Migrane: Results from the MOVEMENT Phase 3 Trial

### A. Jones, C. O'Gorman, H. Tabuteau

#### Axsome Therapeutics, New York, NY, United States

##### **Correspondence:** A. Jones; C. O'Gorman

Background and objective: AXS-07 (MoSEIC™ meloxicam-rizatriptan) is a novel, oral, rapidly-absorbed, multimechanistic investigational medicine.

The MOVEMENT trial aimed to evaluate the long-term efficacy & safety of AXS-07 for the acute treatment of migraine.

Methods: MOVEMENT was a Phase 3, long-term, open-label study that enrolled patients who had completed the previous pivotal trials of AXS-07: MOMENTUM and INTERCEPT. Patients could treat up to 10 migraine attacks per month over the up to 12-month period, with 1 dose of AXS-07 (20 mg MoSEIC meloxicam/10 mg rizatriptan) for each migraine.

Results: 706 patients were enrolled. AXS-07 rapidly and substantially relieved pain and most bothersome symptoms (MBS). Pain relief (PR) was achieved by 39% and 68% of patients within 1 and 2hrs post dose, respectively. Pain freedom (PF) and absence of MBS was achieved by 38% and 47% of patients, respectively 2hrs post dose. PR with AXS-07 was durable. Sustained PR from 2-24 and 2-48hrs was achieved by 60% and 59% of patients, respectively. Sustained PF from 2-24 and 2-48hrs was achieved by 33% and 32% of patients, respectively. 85% of patients remained free of rescue medication use through 24hrs, and 83% through 48hrs, after a single dose. AXS-07 was generally safe and well tolerated. The most commonly reported adverse events (≥3%) over the 12-month treatment period were nausea, dizziness, and vomiting.

Conclusion: AXS-07 rapidly, substantially, and durably relieved migraine pain and MBS.

## P0281 Ubrogepant Was Safe and Well Tolerated in the Acute Treatment of Perimenstrual Migraine

### J. M. Pavlovic^1,2^, J. Ailani^3^, S. Hutchinson^4^, H. Lai^5^, B. Dabruzzo^5^, S. Y. Yu^5^, J. M. Trugman^5^, A. MacGregor^6,7^

#### ^1^Albert Einstein College of Medicine, Bronx, NY, United States; ^2^Montefiore Headache Center, Bronx, NY, United States; ^3^MedStar Georgetown University Hospital, Washington, DC, United States; ^4^Orange County Migraine and Headache Center, Irvine, CA, United States; ^5^AbbVie, Madison, NJ, United States; ^6^Barts and the London School of Medicine and Dentistry, Centre for Neuroscience, Surgery and Trauma, Blizard Institute of Cell and Molecular Science, London, United Kingdom; ^7^St. Bartholomew’s Hospital, Centre for Reproductive Medicine, London, United Kingdom

##### **Correspondence:** J. M. Pavlovic

Objective: To determine the efficacy and safety of ubrogepant in the acute treatment of perimenstrual migraine (pmM) attacks.

Methods: Phase 3, randomized, open-label, 52-week extension trial of adults with migraine randomized to usual care, ubrogepant 50 mg, or 100 mg and treated up to 8 migraine attacks (any pain severity) per 4-week interval. In this post hoc analysis of female participants, a migraine attack was considered perimenstrual (pmM) if it started on or between 2 days before and 3 days after the start of menstrual bleeding. Efficacy was assessed via the proportion of treated attacks achieving pain freedom and pain relief at 2 hours.

Results: The trial included 734 female participants overall and 354 participants who reported ≥1 menstrual cycle start date; 1329 pmM attacks and 16,145 non-pmM attacks were treated with ubrogepant. In the 50 mg dose group, pain freedom at 2 hours was achieved in 28.7% of ubrogepant-treated pmM attacks compared with 22.1% of non-pmM attacks (*P*=0.054). In the 100 mg dose group, pain freedom at 2 hours was achieved in 29.7% of ubrogepant-treated pmM attacks compared with 25.3% of non-pmM attacks (*P*=0.757). Pain relief at 2 hours was achieved in 64.8% of pmM attacks vs 65.2% of non-pmM attacks in the 50 mg dose group (*P*=0.683) and 67.1% vs 68.4% in the 100 mg dose group (*P*=0.273).

Conclusion: In this randomized 52-week extension trial, the efficacy of ubrogepant for the treatment of pmM was comparable to that observed for non-pmM.

## P0282 Monthly Migraine Days, Tablet Utilization, and Quality of Life Associated with Rimegepant – Post Hoc Results from an Open Label Safety Study (BHV3000-201)

### K. Johnston^1^, L. Harris^2^, L. Powell^1^, E. Popoff^1^, V. Coric^2^, G. L'Italien^2^, C. P. Schreiber^3^

#### ^1^Broadstreet Health Economics & Outcomes Research, Vancouver, Canada; ^2^Biohaven Pharmaceuticals, New Haven, CT, United States; ^3^CMH Neurology and Headache Center, Bolivar, MO, United States

##### **Correspondence:** K. Johnston

Objective: The objective was to describe patterns in monthly migraine days (MMD), tablet utilization and estimate health-related quality of life (HRQoL) measures in patients treated with rimegepant 75 mg.

Methods: Eligible subjects were a subset of the BHV3000-201 trial: adults with ≥1 year migraine history and 6-14 MMD at baseline, treated with rimegepant 75 mg up to once daily as-needed (PRN) for up to 52 weeks. MMDs, tablets taken and tablet-to-MMD ratio were calculated every 4 weeks. An economic evaluation by the Institute for Clinical and Evaluative Review (ICER) was used to characterize HRQoL impact of rimegepant versus usual care, as well as migraine free periods. This was combined with MMD data to estimate accumulated quality-adjusted life years (QALYs).

Results: Among 1,114 subjects MMDs were 10.4 at baseline, decreasing to 7.7 by week 52. Tablet use also decreased (Figure 1), from 7.7 tablets in weeks 4-8, to 7.2 tablets in weeks 48-52. This trajectory was associated with an estimated 0.922 QALYs over one year (of a maximum 1.0). If patients remained at baseline of 10.4 MMD, QALYs of 0.898 were estimated.

Conclusion: Ongoing acute treatment with rimegepant 75 mg PRN over one year was associated with reduced MMDs and corresponding monthly tablet utilization reduction. These data suggest that repeated rimegepant PRN did not lead to a medication overuse headache trend. MMD reductions and rimegepant for acute migraine episodes jointly resulted in improved HRQoL estimates.

**Fig. 1 (abstract P0282).**
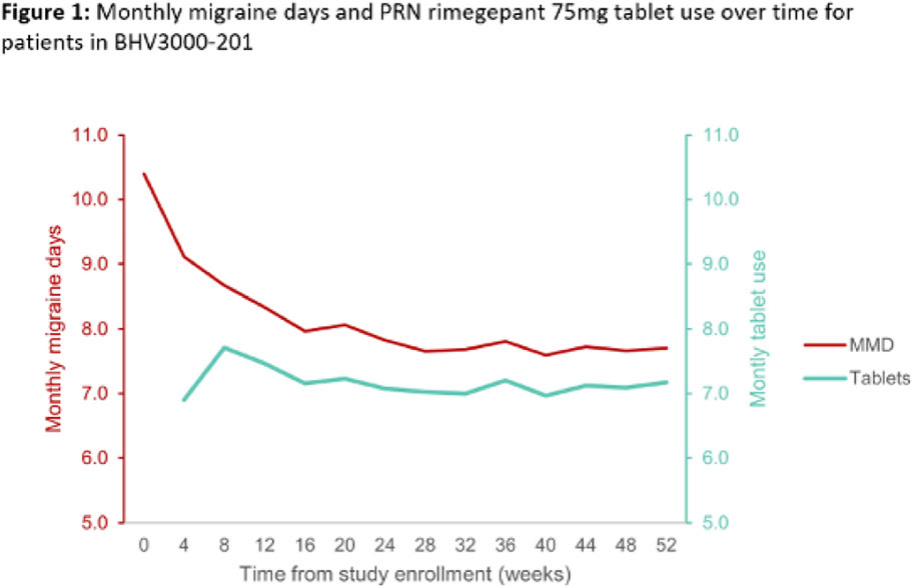
See text for description

## P0283 Assessment of changes in the severity of photofobia of migraine patients after great occipital nerve block

### J. A. Membrilla, I. de Lorenzo, L. Sánchez-Casado, M. Sastre-Real, J. Diaz-de-Terán

#### La Paz University Hospital, Neurology, Madrid, Spain

##### **Correspondence:** J. A. Membrilla

Background and objective: To study the effect of greater occipital nerve (GON) block over photophobia in migraine.

Methods**:** Observational prospective case-control study of migraine patients with photophobia attending the Headache Unit of a third-level hospital. Cases were defined as patients receiving GON block, which was performed at visit 1 (V1). All patients were evaluated with the Hospital Anxiety and Depression Scale, the Migraine Specific Quality of Life Questionnaire, the Utah Photophobia Symptom Impact Scale (UPSIS-12) and the Korean Photophobia Questionnaire (KUMC-8); both in V1 and one week after (V2).

Results**:** 41 patients were recruited; 28 cases and 13 controls. At V1, there were not significant differences in UPSIS (mean±SD): cases 29.4±8.3 vs controls 27.8±8.1, p=0.558) and KUMC-8 (cases 6.7±1.2 vs controls 6.2±1.7, p=0.323. At V2, cases experimented a significant improvement in photophobia impact scales compared to controls (UPSIS-12: reduction of 6.0±6.5 points, p<0.001; KUMC-8: reduction of 1.2±1.8 points, p=0.002). The other used scales did not show significant variation. Lesser improvement was seen in migraine with aura, but this was not statistically significant (reduction of 4.4±4.1 vs 8.5±8.7, p=0.101).

Conclusion**:** GON block has a beneficial effect over photophobia in migraine patients, measured with UPSIS-12 and KUMC-8. Patients without aura may have a greater improvement. GON block could be a useful therapeutic technique for photophobia in migraine.

## P0284 Quickest Way to Less Headache Days: an operational research model and its implementation for Chronic Migraine

### P. Zhang^1^, I. Lo^2^

#### ^1^Rutgers Robert Wood Johnson Medical School, New Brunswick, NJ, United States; ^2^Stanford University, Stanford, A, United States

##### **Correspondence:** P. Zhang

Background: Choosing migraine prevention medications often involves trial and error. Operations research (OR) allow us to derive a mathematically optimum way to conduct such trial and error processes.

Objective: Given probability of success and adverse events as a function of time, we seek to develop and solve an OR model, applicable to any arbitrary patient, minimizing time until discovery of an effective migraine prevention medication. We then seek to apply our model to real life data for chronic migraine prevention.

Design: An OR model is developed and then solved for the optimum solution, taking into account the likelihood of reaching 50% headache day reduction as a function of time. We then estimate key variables using FORWARD study as well as erenumab data published by Barbanti et al. at IHC 2019.

Results: The solution for our model is to order the medications in decreasing order by probability of efficacy per unit time. This result can be generalized through calculation of Gittins index. In chronic migraine the optimum sequence of prevention medication trial is erenumab for 12 weeks, followed Botox for 32 weeks, followed by topiramate for 32 weeks.

Conclusions: We propose an optimal sequence for preventive medication trial for patients with chronic migraine. Since our model makes limited assumptions on the characteristics of disease, our model can be applied to other scenarios so long as probability of success/adverse event as a function of time can be estimated.

## P0285 Real-World Evidence for Control of Patients With Chronic Migraine Who Received Calcitonin Gene–Related Peptide Monoclonal Antibody Therapy Added to OnabotulinumtoxinA Treatment

### A. M. Blumenfeld^1^, B. M. Frishberg^1^, J. D. Schim^1^, O. Hughes^2^, A. Manack Adams^3^

#### ^1^Headache Center of Southern California, Carlsbad, CA, United States; ^2^ICON plc, Boston, MA, United States; ^3^Allergan, an AbbVie Company, Irvine, CA, United States

##### **Correspondence:** A. M. Blumenfeld

Objective: Collect real-world data and improve our understanding of the potential benefits of adding a CGRP mAb to onabotulinumtoxinA (onabotA) in CM.

Methods: This chart review included adults with CM treated at 1 clinic (10/2018–11/2019) with ≥2 consecutive onabotA injections before ≥1 month of onabotA plus erenumab, fremanezumab, or galcanezumab. Charts at time of first CGRP mAb prescription (baseline) and up to 4 visits over ≤12 months were reviewed for adverse events (AEs), discontinuations, monthly headache days (MHDs), and migraine-related disability (MIDAS). Outcomes were also evaluated in patients who completed ~12 months of onabotA treatment (4 visits) after starting CGRP mAb.

Results: Of 300 charts reviewed, 257 met criteria for the primary cohort; 103 (40%) were completers. The CGRP mAbs were erenumab (primary: 78%; completers: 84%), galcanezumab (16%; 11%), and fremanezumab (6%; 5%). Patients discontinued CGRP mAb more than onabotA (23% vs 3%). The most common AE was constipation (9%). Mean MHDs were 21.5 (primary) and 22.4 (completers) before initiating onabotA, and 12.1 before adding CGRP mAb in both cohorts. Following the initiation of combination treatment, mean MHDs significantly decreased at all visits (month 12: 4.0 [95% CI: −5.4,−2.6]), and 44.8% had a ≥5-point decrease in MIDAS at ~12 months. Similar results were observed for completers.

Conclusion: This real-world study demonstrated benefits with onabotA alone and additive benefits with CGRP mAb.

## P0286 Consecutive Headache-Free Days With OnabotulinumtoxinA Treatment in Patients With Chronic Migraine: A Pooled PREEMPT Analysis

### H. C. Diener^1^, D. W. Dodick^2^, R. B. Lipton^3^, K. Sommer^4^, S. D. Silberstein^5^

#### ^1^University of Duisburg-Essen, Essen, Germany; ^2^Mayo Clinic, Phoenix, AZ, United States; ^3^Albert Einstein College of Medicine, Bronx, NY, United States; ^4^Allergan, an AbbVie Company, Marlow, United Kingdom; ^5^Thomas Jefferson University, Jefferson Headache Center, Philadelphia, PA, United States

##### **Correspondence:** H. C. Diener

Objective: Evaluate the impact of onabotulinumtoxinA (onabotA) versus placebo on the number of consecutive headache-free days (HFDs) and days without moderate/severe headache in chronic migraine (CM).

Methods: This was a post hoc analysis of the phase 3, 24-week, randomized, double-blind PREEMPT trials (NCT00156910, NCT00168428). A headache day was defined as a day with ≥4 continuous headache hours. Participants recorded headache severity as mild, moderate, or severe. Percentages of participants who experienced ≥7, ≥14, and ≥21 consecutive days without headache or without a moderate/severe headache requiring acute medication were compared.

Results: A total of 1384 participants were randomized to onabotA (n=688) or placebo (n=696). During the 28-day screening phase, the mean number of headache days was 19.9 for onabotA and 19.8 for placebo. During double-blind treatment, significantly more participants treated with onabotA than placebo experienced ≥7 (70% vs 64%; *P*=0.039), ≥14 (40% vs 31%; *P*<0.001), and ≥21 (26% vs 18%; *P*<0.001) consecutive HFDs without acute medication use. Significant differences favoring onabotA remained when the analysis was restricted to participants who experienced ≥7 (74% vs 68%; *P*=0.018), ≥14 (42% vs 34%; *P*=0.003), and ≥21 (28% vs 21%; *P*=0.003) consecutive moderate/severe HFDs without acute medication use.

Conclusion: OnabotA treatment resulted in significantly more consecutive HFDs and moderate/severe HFDs than placebo in CM patients.

## P0287 Migraine evolution after discontinuation of preventive treatment with CGPR-(receptor) antibodies: a prospective, longitudinal study

### B. Raffaelli, M. Terhart, L. H. Overeem, J. Mecklenburg, L. Neeb, U. Reuter

#### Charité University Hospital Berlin, Department of Neurology, Berlin, Germany

##### **Correspondence:** B. Raffaelli

Objective: To evaluate the course of migraine after discontinuation of migraine preventive treatment with CGRP-(receptor) monoclonal antibodies (mAb) following EHF guidelines.

Methods: This longitudinal cohort study included patients with migraine who received a CGRP-(receptor) mAb for ≥8 months before treatment discontinuation. We collected headache data during the four weeks prior to mAb treatment initiation (baseline), in the month before the last treatment injection, in weeks 1-4 and weeks 9-12 after treatment completion (i.e. weeks 5-8 and 13-16 after the last injection). Primary outcome of the study was the number of monthly migraine days (MMD) at baseline and at every study intervall. Secondary outcomes were the number of monthly headache days (MHD) and monthly days with acute medication use (AMD).

Results: We included n=62 patients in the study, n=31 treated with erenumab and n=31 treated with galcanezumab or fremanezumab. Patients reported 13.27 ±6.40 MMD at baseline, which decreased to 8.24 ±6.59 in the last active treatement period (p<0.001). In the first month of the drug holiday, MMD increased to 10.32 ±6.85 but remained significantly lower than baseline (p=0.033). In the third month, MMD returned to baseline levels (12.47 ±6.64, p>0.999). MHD and AMD also showed a gradual worsening starting with the first month after interruption.

Conclusion: The discontinuation of migraine prevention with CGRP-(receptor) mAbs led to a progressive worsening of migraine over time.

## P0288 Changes in quality of life after discontinuation of migraine preventative treatment with CGRP(-receptor)-antibodies

### M. Tehart, B. Raffaelli, L. H. Overeem, J. Mecklenburg, L. Neeb, U. Reuter

#### Chaité University Hospital Berlin, Department of Neurology, Berlin, Germany

##### **Correspondence:** M. Tehart

Objective: To evaluate patients" quality of life after discontinuation of migraine prophylaxis with monoclonal antibodies (mAb) targeting calcitonin gene-related peptide (CGRP) or its receptor (CGRP-R).

Methods: We included migraine patients after 8-12 months of therapy with a CGRP(-R)mAb and before a planned discontinuation attempt. Quality of life was measured by the Headache Impact Test (HIT-6), the Short-Form (SF-12) with its Physical Component Score (PCS-12) and Mental Component Score (MCS-12), the Depression Anxiety and Stress Scale (DASS-21), the Euroquol form EQ-5D-5L and the Patient-Reported Outcomes Measurement Information System (PROMIS) during the last treatment month, weeks 5-8 and 13-16 after the last mAb injection.

Results: Complete data were available from 58 patients, 27 patients received the CGRP-R-mAb erenumab, and 31 the CGRP mAbs galcanezumab or fremanezumab. During weeks 13-16 after the last mAb injection, HIT-6 scores deteriorated from 59.3±7.0 to 63.5±6.2 (p<0.001). The PCS-12 and the MCS-12 worsened by 4.2±8.0 points (p=0.010) and 2.9±9.3 points (p=0.002) respectively. The EQ-5D-5L and DASS-21 also declined by 0.07±0.2 points (p=0.042) and 2.7±7.9 points (p=0.042). The changes in HIT-6, PCS-12, and EQ-5D-5L were already significant in the first month of the drug holiday. PROMIS scores did not change significantly.

Conclusion: Our results show a significant decline in the quality of life of migraine patients after treatment discontinuation of a CGRP(-R)mAb.

## P0289 Eptinezumab for Migraine Prevention in Patients 50 Years or Older: A Subgroup Analysis of PROMISE-1 and PROMISE-2

### V. Martin^1^, C. Tassorelli^2^, A. Ettrup^3^, J. Hirman^4^, R. Cady^5^

#### ^1^UC Headache and Facial Pain Center, Cincinnati, OH, United States; ^2^IRCCS C. Mondino Foundation, Pavia, Italy; ^3^H. Lundbeck A/S, Copenhagen, Denmark; ^4^Pacific Northwest Statistical Consulting, Inc., Woodinville, WA, United States; ^5^Lundbeck La Jolla Research Center, San Diego, CA, United States

##### **Correspondence:** A. Ettrup

Objective: To evaluate the efficacy and safety of eptinezumab for the migraine prevention in patients ≥50 years of age, a subpopulation that may pose unique challenges to preventive treatment due to the greater prevalence of comorbidities and use of concomitant medication.

Methods: Patients with episodic migraine (EM; PROMISE-1) or chronic migraine (CM; PROMISE-2) and ≥50 years of age at baseline were included. PROMISE-1 included adults ≤75 years old; PROMISE-2 included adults ≤65 years old. In both studies, the primary efficacy outcome was the reduction in monthly migraine days (MMDs) over weeks 1–12.

Results: Across studies, 385 patients were ≥50 years old (100 mg, n=132; 300 mg, n=127; placebo, n=126). More patients had CM (n=242 [63%]) than EM (n=143 [37%]). In CM patients ≥50 years old, mean changes from baseline in MMDs over weeks 1–12 were –7.7 (100 mg) and –8.6 (300 mg) with eptinezumab vs –6.0 with placebo. In EM patients ≥50 years old, mean changes were –3.8 (100 mg) and –4.4 (300 mg) with eptinezumab vs –2.6 with placebo. These changes were comparable to the total PROMISE-1 and PROMISE-2 populations. A similar percentage of patients experienced TEAEs across treatment groups (100 mg, 46.6%; 300 mg, 53.5%; placebo, 52.4%).

Conclusion: In this post hoc subgroup analysis, the efficacy of eptinezumab in patients with migraine ≥50 years old was comparable to that in the overall clinical trial populations, with an equally favorable safety and tolerability profile.

## P0290 70 or 140 mg to start? Predictive factors of response after 6 months to 70 mg of erenumab as a starting dose in patients with chronic migraine

### N. Morollón^1^, R. Belvís^1^, A. De Dios^2^, N. Pagès^2^, M. Massip^2^

#### ^1^Hospital de la Santa Creu i Sant Pau, Neurology, Barcelona, Spain; ^2^Hospital de la Santa Creu i Sant Pau, Pharmacy, Barcelona, Spain

##### **Correspondence:** N. Morollón

Introduction: There is a lack of data to help us know which patients will respond to the 70 mg/month dose of erenumab and which will need twice the dose.

Methods: We compared demographic and clinical variables of the patients who had a response to 70 mg / month and those who required an increase to a dose of 140 mg/28 days.

Results: We included 48 patients on erenumab treatment for at least 3 months. 22 patients responded to doses of 70 mg/28 days, 25 patients required an increase in dose to 140 mg/28 days, in 1 patient erenumab was withdrawn due to side effects. No statistically significant differences were found between both groups in terms of mean age (50.7 vs. 47.87 years, p = 0.21), years of evolution of migraine (31 vs. 33.5, p = 0, 53), years of evolution of chronic migraine (9.15 vs. 7.12, p = 0.3), headache days per month (27.3 vs. 26.3, p = 0.51), days of migraine per month (14 vs. 13.9, p = 0.9) or in the number of proven preventive treatments (4.6 vs. 5.12, p = 0.3). Significant differences were found in terms of medication abuse (77.2% of patients with 70 mg vs. 84% in patients with 140 mg, p = 0.008) and regarding the absence of response to botulinum toxin (55% in patients with 40 mg vs. 64% in those with 140 mg, p = 0.03).

Conclusion: The excess consumption of analgesic medication and the absence of response to botulinum toxin appear as predictive factors that patients will require treatment with erenumab at a dose of 140 mg/28 days.

## P0291 What if a monoclonal antibody doesn't work as a migraine preventive treatment? Description of the experience in switching between monoclonals antibodies in a Headache Unit

### N. Morollón^1^, R. Belvís^1^, A. De Dios^2^, N. Pagès^2^, M. Massip^2^

#### ^1^Hospital de la Santa Creu i Sant Pau, Neurology, Barcelona, Spain; ^2^Hospital de la Santa Creu i Sant Pau, Pharmacy, Barcelona, Spain

##### **Correspondence:** N. Morollón

Introduction: There is a lack of data to help us know when and how to switch from one ineffective monoclonal antibody to another in migraine patients.

Methods: We describe how the switch from an ineffective monoclonal antibody to another has been made, the result and the safety of the change. We considered ineffective treatment when the patient experienced less than 50% reduction in migraine days per month after 12 weeks of treatment. We chose the antibody to make the switch based on the target of the previous antibody (erenumab acts against the receptor of the peptide related to the calcitonin gene, while galcanezumab and fremanezumab act by blocking the ligand). The switch was made directly, without waiting longer than the half-life of the previous antibody.

Results: We included 19 patients with chronic migraine that had not responded to the first monoclonal antibody. 89,4% were women and the average age was 47,7 years old. A total of 21 changes were made, 5 changes from erenumab to galcanezumab, 10 changes from erenumab to fremanezumab, 4 changes from galcanezumab to erenumab, and 2 patients, as a last chance, from galcanezumab to fremanezumab.. 33.3% of patients got to be responders after switching from one antibody to another. No patient had adverse effects in the context of switching from one to the other

Conclusion: If a first monoclonal antibody is ineffective as a preventive treatment for migraine, it may be a good option to change to another with a different target because a third of the patients will be able to achieve improvement. The direct switch from one monoclonal antibody to another is safe.

## P0292 Effects of Atogepant as Evaluated by the Activity Impairment in Migraine-Diary (AIM-D) and Headache Impact Test (HIT-6) in a 12-Week, Double-blind, Randomized Phase 3 (ADVANCE) Trial for Preventive Treatment of Migraine

### R. B. Lipton^1^, P. Pozo-Rosich^2,3^, A. M. Blumenfeld^4^, Y. Li^5^, L. Severt^5^, J. Stokes^5^, P. Gandhi^5^, D. W. Dodick^6^

#### ^1^Albert Einstein College of Medicine, Bronx, NY, United States; ^2^Vall d’Hebron University Hospital, Headache Unit, Neurology Department, Barcelona, Spain; ^3^Universitat Autonoma of Barcelona, Headache Research Group, VHIR, Barcelona, Spain; ^4^Headache Center of Southern California, Carlsbad, CA, United States; ^5^AbbVie, Madison, NJ, United States; ^6^Mayo Clinic, Phoenix, AZ, United States

##### **Correspondence:** R. B. Lipton

Objective: To evaluate the effect of atogepant, an oral CGRP receptor antagonist for migraine prevention, on 2 patient-reported outcome measures: AIM-D and HIT-6.

Methods: Phase 3, randomized, double-blind, placebo (PBO)-controlled trial (ADVANCE; NCT03777059). Participants with 4−14 migraine d/mo received oral atogepant 10, 30, or 60mg, or PBO once daily for 12 wks. The AIM-D, collected daily by e-diary, has 2 domains (0–100 scale; lowest–greatest impact): Performance of Daily Activities (PDA) and Physical Impairment (PI). Changes from baseline in mean monthly AIM-D PDA or PI scores across 12 wks were alpha-controlled secondary endpoints. HIT-6 (completed monthly) was exploratory.

Results: Of 910 participants randomized, 902 were treated (mean age 42 y; 89% female), including 873 in modified intent-to-treat population (10mg n=214; 30mg n=223; 60mg n=222; PBO n=214). All atogepant groups had functional improvement vs PBO based on AIM-D PDA and PI scores across 12 wks. Differences were statistically significant for the 60 and 30mg doses (least-squares mean difference vs PBO: PDA 60mg: −3.32, 30mg: –2.54; PI 60mg: −2.46, 30mg: −1.99), but not for 10mg (PDA −1.19; PI −1.08). All atogepant groups had significant improvement in HIT-6 total score and HIT-6 responder rates vs PBO at wks 4, 8, and 12, except responder rate for the 30mg dose at wk 4.

Conclusion: Atogepant significantly reduced impairment in PDA, PI, and headache impact relative to placebo.

## P0293 Atogepant Improved Patient-Reported Migraine-Specific Quality of Life in a 12-Week Phase 3 (ADVANCE) Trial for Preventive Treatment of Migraine

### R. B. Lipton^1^, P. Pozo-Rosich^2,3^, A. M. Blumenfeld^4^, Y. Li^5^, L. Severt^5^, J. Stokes^5^, P. Gandhi^5^, D. W. Dodick^6^

#### ^1^Albert Einstein College of Medicine, Bronx, NY, United States; ^2^Vall d’Hebron University Hospital, Headache Unit, Neurology Department, Barcelona, Spain; ^3^Universitat Autonoma of Barcelona, Headache Research Group, VHIR, Barcelona, Spain; ^4^Headache Center of Southern California, Carlsbad, CA, United States; ^5^AbbVie, Madison, NJ, United States; ^6^Mayo Clinic, Phoenix, AZ, United States

##### **Correspondence:** R. B. Lipton

Objective: Evaluate effects of atogepant, an oral CGRP receptor antagonist in development for migraine prevention, on Migraine-Specific Quality of Life Questionnaire v2.1 (MSQ).

Methods: Phase 3, randomized, double-blind, placebo (PBO)-controlled trial (ADVANCE; NCT03777059). Participants (4−14 migraine d/mo) received oral atogepant 10, 30, 60mg, or PBO once daily for 12 wks. Change from baseline in MSQ Role Function–Restrictive (RFR) domain at wk 12 was an alpha-controlled secondary endpoint. Least-squares mean differences (95% CI) vs PBO for change from baseline at wks 4, 8, and 12 in MSQ RFR, Role Function–Preventive (RFP), and Emotional Function (EF) domains are reported.

Results: Of 910 participants randomized, 902 were treated (mean age 42 y; 89% female) and 873 were included in the modified intent-to-treat population (10mg n=214; 30mg n=223; 60mg n=222; PBO n=214). At wk 12, all atogepant groups had statistically significant improvements vs PBO in RFR (10mg, 9.9 [5.5–14.4]; 30mg, 10.1 [5.7–14.5], 60mg, 10.8 [6.4–15.2]), RFP (10mg, 5.8 [1.9–9.6]; 30mg, 6.9 [3.1–10.7]; 60mg, 7.1 [3.3–10.9]), and EF (10mg, 8.3 [3.4–13.1]; 30mg, 9.7 [4.9–14.4]; 60mg, 10.5 [5.8–15.3]). For RFR, significant differences vs PBO occurred at the earliest assessment (wk 4). All atogepant groups achieved within-group minimally important difference in each domain at wks 4, 8 and 12.

Conclusion: Atogepant produced significant and clinically meaningfully improved migraine-specific quality of life.

## P0294 Oral Daily Atogepant for the Preventive Treatment of Migraine Increases Responder Rates for Reduction in Mean Monthly Migraine Days

### R. B. Lipton^1^, P. Pozo-Rosich^2,3^, A. M. Blumenfeld^4^, D. W. Dodick^5^, P. McAllister^6^, Y. Li^7^, K. Lu^7^, B. Dabruzzo^7^, R. Miceli^7^, L. Severt^7^, M. Finnegan^7^, J. M. Trugman^7^

#### ^1^Albert Einstein College of Medicine, Bronx, NY, United States; ^2^Vall d’Hebron University Hospital, Headache Unit, Neurology Department, Barcelona, Spain; ^3^Universitat Autonoma of Barcelona, Headache Research Group, VHIR, Barcelona, Spain; ^4^Headache Center of Southern California, Carlsbad, CA, United States; ^5^Mayo Clinic, Phoenix, AZ, United States; ^6^New England Institute for Neurology and Headache, Stamford, CT, United States; ^7^AbbVie, Madison, NJ, United States

##### **Correspondence:** R. B. Lipton

Objective: To assess responder rates at various times after initiating atogepant treatment.

Methods: A 12-week phase 3 trial evaluated the safety, efficacy, and tolerability of atogepant for the preventive treatment of migraine (ADVANCE; NCT03777059). Participants (18–80 years) with a ≥1-year history of migraine (with/without aura) consistent with *International Classification of Headache Disorders* (*3rd ed*) criteria, experiencing 4–14 migraine days/month, were randomized to receive oral atogepant 10, 30, or 60mg or placebo once daily. These analyses evaluated ≥25%, ≥50%, ≥75%, and 100% reductions in mean monthly migraine days (MMDs) across 12 weeks and each 4-week interval. Adverse events (AEs) in ≥5% of participants are reported.

Results: The efficacy analysis population included 873 participants: placebo: n=214; atogepant: 10mg: n=214; 30mg: n=223; 60mg: n=222. Atogepant-treated participants were more likely to experience a ≥50% reduction in the 3-month mean MMDs (56–61% vs 29% with placebo; *P*<0.0001). The proportion of participants experiencing ≥25%, ≥50%, ≥75%, and 100% reductions in mean MMDs significantly increased during each 4-week interval (≥50% reduction: 48–71% vs 27–47% with placebo). The most common AEs for atogepant were constipation (6.9–7.7%) and nausea (4.4–6.1%).

Conclusion: The use of oral, once-daily atogepant 10, 30, and 60mg significantly increased responder rates at all thresholds with approximately 60% achieving a ≥50% reduction in mean MMDs at 12 weeks.

## P0295 Daily Atogepant Provides a Rapid Onset and Sustained Benefit in the Preventive Treatment of Migraine

### T. J. Schwedt^1^, R. B. Lipton^2^, J. Ailani^3^, S. D. Silberstein^4^, C. Tassorelli^5^, H. Guo^6^, K. Lu^6^, B. Dabruzzo^6^, R. Miceli^6^, L. Severt^6^, M. Finnegan^6^, J. M. Trugman^6^

#### ^1^Mayo Clinic, Phoenix, AZ, United States; ^2^Albert Einstein College of Medicine, Bronx, NY, United States; ^3^MedStar Georgetown University Hospital, Washington, DC, United States; ^4^Thomas Jefferson University, Jefferson Headache Center, Philadelphia, PA, United States; ^5^University of Pavia, Department of Neurology, Pavia, Italy; ^6^AbbVie, Madison, NJ, United States

##### **Correspondence:** T. J. Schwedt

Objective: To evaluate the time course of efficacy with atogepant during the ADVANCE trial.

Methods: In this 12-week, randomized, double-blind, placebo-controlled, phase 3 trial, participants (18–80 y) with 4–14 migraine days/month were randomized to placebo, atogepant 10 mg, atogepant 30 mg, or atogepant 60 mg tablets once daily for 12 weeks. Efficacy outcomes included change from baseline in mean monthly migraine days (MMDs), change in weekly migraine days, and proportion of participants with a migraine on each day.

Results: During weeks 1–4, mean change from baseline in MMDs was -3.1 for atogepant 10 mg, -3.4 for atogepant 30 mg, -3.9 for atogepant 60 mg vs -1.6 for placebo (*P<*0.0001 all dose groups). This difference between all atogepant doses and placebo was maintained during weeks 5–8 (*P*≤0.012) and weeks 9–12 (*P*≤0.0002). Mean change from baseline in weekly migraine days during the first week was -0.77 atogepant 10 mg, -0.94 atogepant 30 mg, -1.03 atogepant 60 mg vs -0.29 placebo (*P*<0.0001 all doses). On the first full day after dose administration, the proportion of participants who reported a migraine day was 14.1% for atogepant 10 mg, 10.8% for atogepant 30 mg, and 12.3% for atogepant 60 mg vs 25.2% in the placebo group (*P*≤0.0071 all doses).

Conclusion: Atogepant provided a rapid and sustained reduction in migraine days including statistically significant reductions as early as the first full day after dosing.

## P0296 Atogepant Significantly Reduces Mean Monthly Migraine Days in the Phase 3 Trial (ADVANCE) for the Prevention of Migraine

### J. Ailani^1^, R. B. Lipton^2^, P. J. Goadsby^3,4^, H. Guo^5^, R. Miceli^5^, L. Severt^5^, M. Finnegan^5^, J. M. Trugman^5^

#### ^1^MedStar Georgetown University Hospital, Washington, DC, United States; ^2^Albert Einstein College of Medicine, Bronx, NY, United States; ^3^NIHR-Wellcome Trust King’s Clinical Research Facility, King’s College London, London, United Kingdom; ^4^University of California, Los Angeles, CA, United States; ^5^AbbVie, Madison, NJ, United States

##### **Correspondence:** J. Ailani

Objective: To evaluate the efficacy, safety, and tolerability of oral atogepant for the preventive treatment of migraine.

Methods: This was a phase 3, multicenter, randomized, double-blind, placebo (PBO)-controlled trial (NCT03777059) of adults (18–80 years) with ≥1-year history of migraine (with/without aura). Participants with 4–14 migraine days/month were randomized 1:1:1:1 to atogepant 10, 30, or 60 mg or PBO once daily for 12 weeks. The primary endpoint was a change from baseline in mean monthly migraine days (MMDs) across the 12-week treatment period. Adverse events (AEs) were collected.

Results: The trial randomized 910 participants, including 902 and 873 in the safety and modified intent-to-treat populations; 805 participants completed the study. Mean change from baseline in MMDs were -3.69, -3.86, and -4.20 for atogepant 10, 30, and 60 mg vs -2.48 for PBO (*P<*0.0001). The percentage of participants who achieved a ≥50% reduction in MMDs across 12 weeks were 56%, 59%, and 61% for atogepant 10, 30, and 60 mg vs 29% for PBO (*P<*0.0001). AEs were reported by 52%–54% of participants for atogepant and 57% for PBO. The most common AEs were constipation (7%–8% vs 0.5% PBO) and nausea (4%–6% vs 2% PBO); none were considered serious. Discontinuations due to AEs were 2%–4% for atogepant and 3% for PBO.

Conclusion: Atogepant produced statistically significant and clinically meaningful reductions in MMDs and was safe and well-tolerated.

## P0297 Anti-CGRP antibodies are effective in patients with a dual diagnosis of migraine and Medication Overuse Headache

### M. Sastre-Real, L. Sánchez-Casado, C. Corral-Quereda, M. J. Ruiz Castrillo, J. Díaz de Terán Velasco

#### La Paz University Hospital, Neurology, Madrid, Spain

##### **Correspondence:** M. Sastre-Real

Objective: Our aim was to evaluate the effectiveness of monoclonal antibodies against CGRP (galcanezumab) or its receptor (erenumab) in a series of patients with migraine and medication overuse headache (MOH).

Methods**:** We analyzed patients with a previous diagnosis of migraine and MOH who received anti-CGRP antibodies between January 2020 and January 2021 in a specialized Headache Clinic.

Results: 93 patients were included. 90.2% were female. Mean age was 49.1±9.9 (mean±SD). 67 (70.5%) received erenumab and 28 (29.5%) galcanezumab. 47.4% had depression and 50.5% had anxiety. All of them had failed to 3 or more preventive treatments including OnabotulinumtoxinA and 55.9% to at least 6 prior preventatives. 70.8% were on concomitant oral preventatives at first injection. MMD decreased from 24±12 to 10±14 and 12±14 at 3 and 6 months (median±IQR) (p<0.0001) with a 50% response rate of 54.4% and a 75% response rate of 21.1% at 6 months. Number of analgesics and number of triptans per month decreased from 19±41 and 17±22 to 8±15 and 6±8 at 6 months (median±IQR) (p<0.0001), leading to a MOH prevalence of 25% at 6 months. Patients also experimented a significant improvement in disability scales (MIDAS and HIT6).

Conclusion: Anti-CGRP antibodies are effective in patients with migraine and comorbid MOH in terms of reduction of MMD, acute medication consumption and disability improvement. 54.4% of patients reduced their MMD to ≥50%.

**Fig. 1 (abstract P0297).**
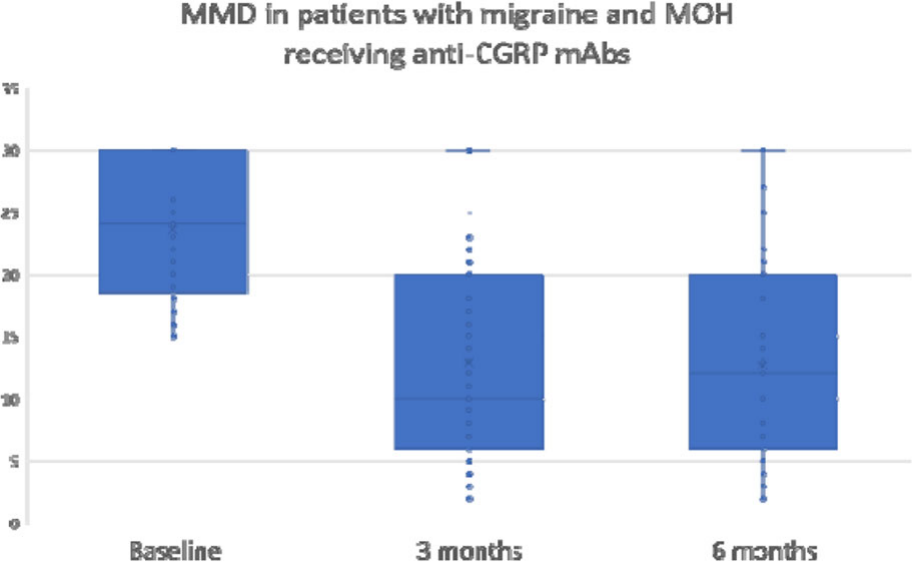
See text for description

## P0298 Effect of erenumab in converting chronic migraine to episodic migraine in a Botulinum toxin-refractory chronic migraine population: Real-world data from a UK secondary care headache clinic

### F. Cheng, J. Murphy, J. Truman, C. Weerasinghe

#### York Teaching Hospitals NHS Trust, Department of Neurology, York, United Kingdom

##### **Correspondence:** F. Cheng

Objective: To investigate real-world erenumab efficacy in converting highly-refractory chronic migraine (CM) to episodic migraine (EM). Methods: 21 chronic migraineurs refractory to OnabotulinumtoxinA and ≥4 oral preventatives were treated with erenumab in a prospective case series in a United Kingdom secondary care headache clinic. Each patient failed a mean of 5.5 oral preventatives and 6.0 cycles of OnabotulinumtoxinA. We measured monthly headache days (MHD), migraine days (MMD) and headache-free days (HFD). Mean MHD<15 and mean MMD<8 days/month responses assessed conversion to EM.

Results**:** Baseline MHD and MMD were 27.3 and 20.0 days. 61.9% had no baseline headache-freedom. 52.4% achieved MHD<15 in any month, first doing so in 2.2±1.7 months (range: 1–6 months); 85.7% achieved MMD<8 in any month, first doing so in 3.4±2.4 months (range: 1–9 months); and 52.4% patients achieved both MHD<15 and MMD<8 in any month, first doing so in 3.1±1.8 months (range: 1–6 months). All patients who achieved MHD<15 in any month also achieved MMD<8 in any month, whilst 33.3% achieved MMD<8 in any month without achieving MHD<15 in any month.

Conclusion**:** In a highly-refractory CM patient cohort, 52.4% achieved both MHD<15 and MMD<8 in any month, thereby achieving conversion to EM. Additionally, 33.3% achieved MMD<8 in any month without achieving MHD<15 in any month. Responder patients first achieved MHD<15 by 6 months and MMD<8 by 9 months.

**Fig. 1 (abstract P0298).**
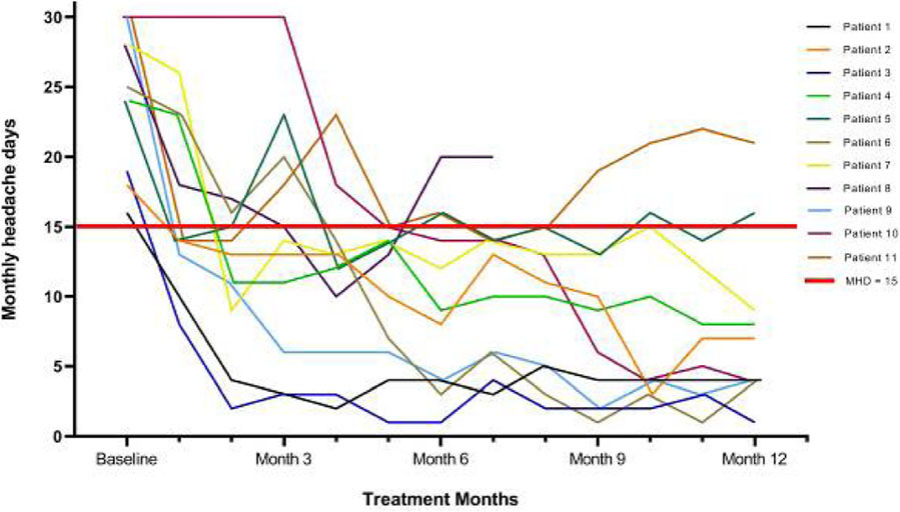
See text for description

**Fig. 2 (abstract P0298).**
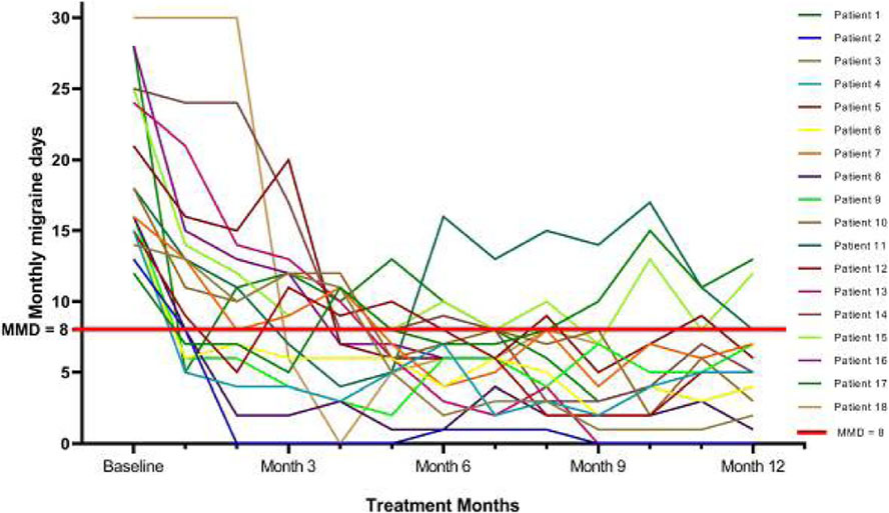
See text for description

## P0299 Daily Dosing of Atogepant for Preventive Treatment of Migraine Improved Patient-Reported Outcomes Measures of Activity Impairment in Migraine-Diary, Migraine-Specific Quality of Life, and Headache Impact Test in a 52-Week Trial

### R. B. Lipton^1^, R. Halker Singh^2^, L. Mechtler^3^, J. McVige^3^, S. Zhao^4^, L. Severt^4^, J. Stokes^4^, P. Gandhi^4^, M. Ashina^5^

#### ^1^Albert Einstein College of Medicine, Bronx, NY, United States; ^2^Mayo Clinic, Scottsdale, AZ, United States; ^3^Dent Neurologic Institute, Buffalo, NY, United States; ^4^AbbVie, Madison, NJ, United States; ^5^Danish Headache Center, University of Copenhagen, Glostrup, Denmark

##### **Correspondence:** R. B. Lipton

To present the patient-reported outcomes (PROs) following 52-weeks of once-daily oral atogepant (ato) 60 mg, a CGRP antagonist being developed for migraine prevention.

This multicenter, open-label trial (NCT03700320) enrolled and randomized adults (4-10 migraine days/month) 5:2 to ato or oral standard of care migraine prevention (SOC). Activity Impairment in Migraine-Diary (AIM-D) Performance of Daily Activities (PDA) and Physical Impairment (PI) domains, Migraine-Specific Quality of Life Questionnaire v2.1 (MSQ v2.1) Role Function-Restrictive (RFR) domain, and HIT-6 total scores were analyzed on the modified intent-to-treat population (mITT; only ato pts).

744 participants were randomized and 521 comprised the mITT (mean age: 42.5 years, 88.3% were female, 76.8% were White). AIM-D PDA and PI domain score changes for first and last timepoints (**Fig. 1**). MSQv2.1 RFR least squares (LS) mean (standard error [SE]) at baseline was 48.1 (20.26) and score changes (**Fig. 2)**. HIT-6 scores followed a similar trend to AIM-D and MSQv2.1 scores. As indicated by confidence intervals not including 0, significant improvements were observed for all PROs at the earliest timepoint assessed and appeared to increase with treatment duration. At Week 52, 80.1% of participants were HIT-6 responders (≥5 points decrease from baseline).

Long-term ato use was associated with reduced impairment due to migraine, improvements in migraine-specific quality of life, and reductions in the impact of headache.

**Fig. 1 (abstract P0299).**
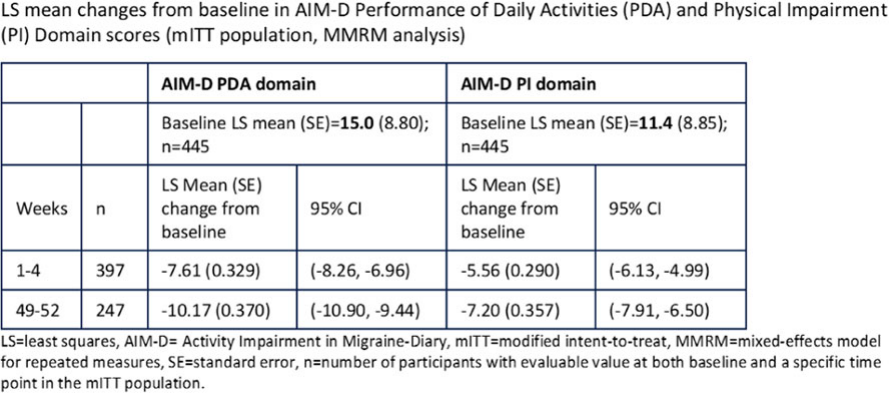
See text for description

**Fig. 2 (abstract P0299).**
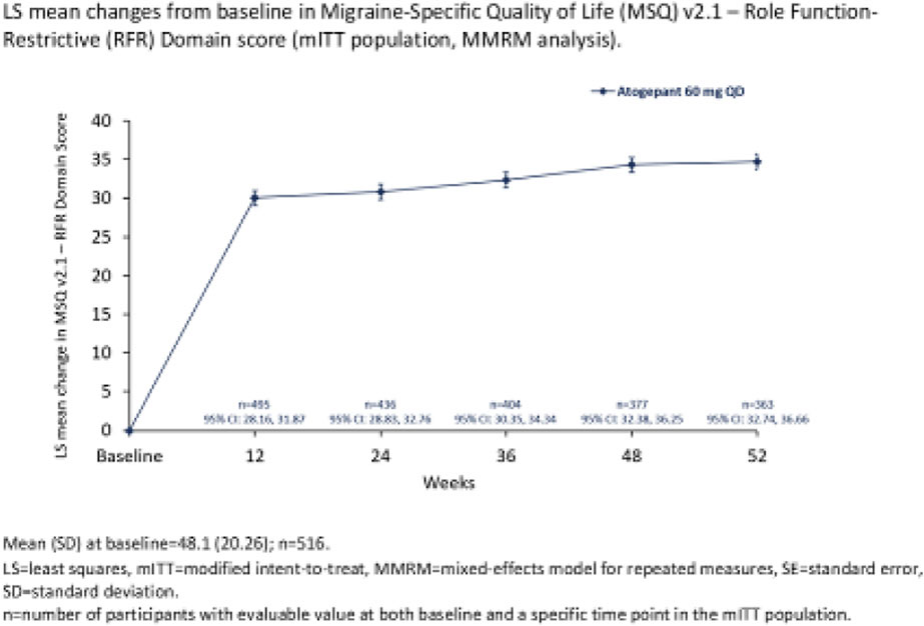
See text for description

## P0300 Real life experience of one year treatment with galcanezumab in chronic migraine with and without medication overuse headache

### G. Vaghi^1,2^, V. Bitetto^1,2^, R. De Icco^1,2^, E. Guaschino^1^, M. Allena^1^, C. Tassorelli^1,2^, G. Sances^1^

#### ^1^IRCCS Mondino Foundation, Headache Science & Neurorehabilitation Center, Pavia, Italy; ^2^University of Pavia, Department of Brain and Behavioral Sciences, Pavia, Italy

##### **Correspondence:** G. Vaghi

Objective: Evaluating one-year effectiveness of galcanezumab in patients with chronic migraine (CM) with and without medication overuse headache.

Methods: We analyzed 26 patients (F22, M4, mean age: 53yrs, migraine history: 38yrs) who failed at least 3 preventive therapies. Galcanezumab was administered monthly for 12 treatments (T1 through T12) with a loading dose of 240 mg and maintenance dose of 120mg. Two patients interrupted treatment for inefficacy at T7 and T9. We collected clinical data on headache features (diary), disability and allodynia (standardized questionnaires) at baseline and quarterly.

Results: Patients with a pattern reversal from chronic to episodic migraine (i. e.>50% responders) were 42% at T1, rising to 62% at T12. Super-responders (i. e.>75% responders) were 8% at T1, 21% at T12. A significant improvement in headache was detected already at T1 and persisted over one-year treatment-Fig. 1. An improvement in MIDAS and HIT-6 scores was detected from T3(p<0.001), while allodynia intensity decreased significantly from T12 (p=0.03)-Fig. 2. Mild side effects were reported by 33% of patients (constipation, cutaneous reaction and fatigue).

Conclusion: Galcanezumab is related to high percentage of pattern reversal in difficult-to-treat patients and to improvement in clinical features already during the 1st month of treatment and in headache-related disability after a few months. Efficacy is maintained over the long-term showing a positive tolerability profile.

**Fig. 1 (abstract P0300).**
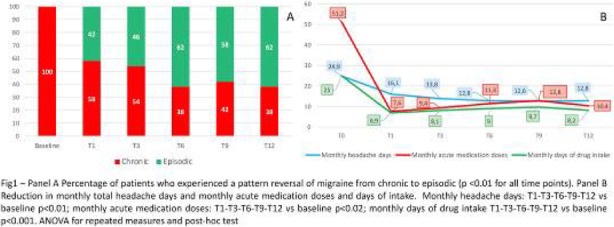
See text for description

**Fig. 2 (abstract P0299).**
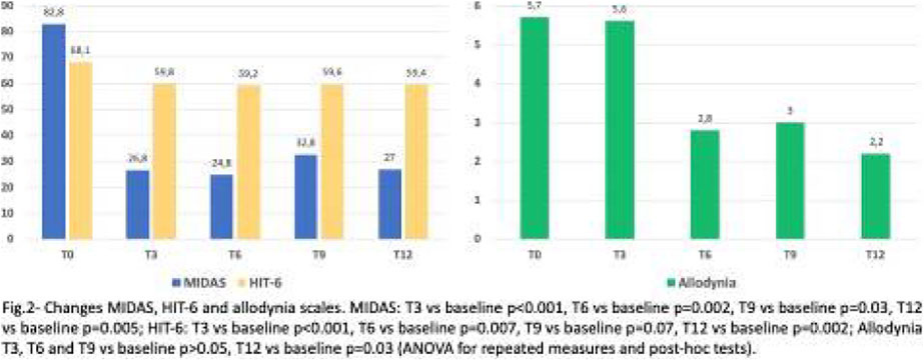
See text for description

## P0301 Atogepant 60 mg Once-Daily Shows Efficacy for the Preventive Treatment of Migraine: Results From a 52-Week Open-Label Extension Trial

### M. Ashina^1^, S. J. Tepper^2^, U. Reuter^3^, A. M. Blumenfeld^4^, S. Hutchinson^5^, J. Xia^6^, R. Miceli^6^, L. Severt^6^, M. Finnegan^6^, J. M. Trugman^6^

#### ^1^Danish Headache Center, University of Copenhagen, Glostrup, Denmark; ^2^Geisel School of Medicine at Dartmouth, Hanover, NH, United States; ^3^Charité University Hospital Berlin, Berlin, Germany; ^4^Headache Center of Southern California, Carlsbad, CA, United States; ^5^Orange County Migraine and Headache Center, Irvine, CA, United States; ^6^AbbVie, Madison, NJ, United States

##### **Correspondence:** M. Ashina

Here we present the 52-week efficacy results for atogepant, an oral, calcitonin gene-related peptide (CGRP) receptor antagonist in development for the preventive treatment of migraine.

Multicenter, open-label trial (NCT03700320) randomized adults with migraine 5:2 to atogepant 60 mg QD (ato) or oral standard of care (SOC) migraine preventive. Efficacy measures (not collected for the SOC arm) were analyzed using a mixed-effects model for repeated measures on the modified intent-to-treat population (mITT).

744 participants (pts) were randomized (ato, n=546); 521 ato pts were in the mITT population (mean age of 42.5 years, 88.3% were female, and 76.8% were White). Baseline mean (SE) monthly migraine days (MMDs) were 7.30 (2.62); least-squares (LS) mean change from baseline at weeks (wks) 1-4 and 49-52 was -3.84 and -5.19. Baseline mean (SE) monthly acute medication use days were 6.63 (3.26); LS mean change at wks 1-4 and 49-52 was -4.04 and -4.93. All 95% confidence intervals were non-zero. The proportions of responders based on reductions in MMDs are shown in the **Figure**; proportions of responders increased throughout the trial.

Atogepant 60 mg once-daily reduced MMDs, acute medication use days, and improved response; response was observed early, sustained over 1 year, and appeared to increase with treatment duration. Results support the use of atogepant as a long-term, preventive treatment of migraine.

**Fig. 1 (abstract P0301).**
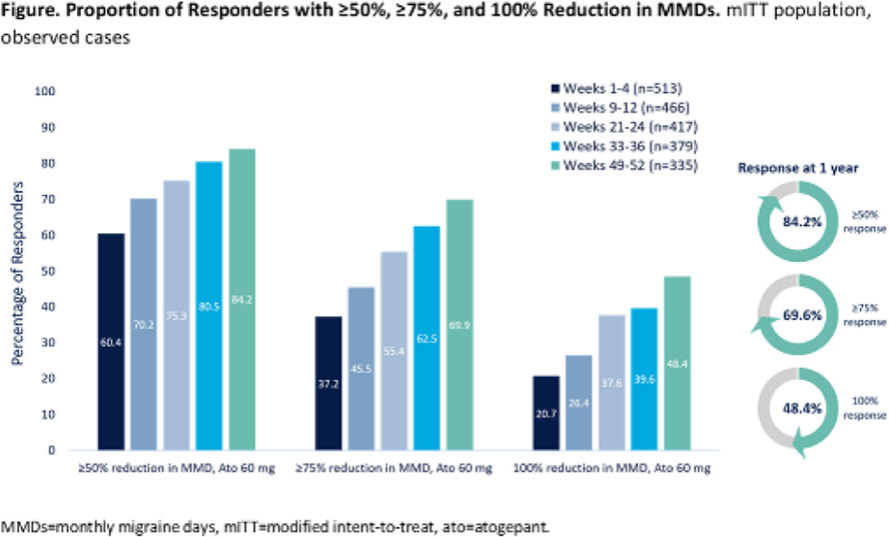
See text for description

## P0302 Does erenumab suspension affect chronic migraine course? A real life experience

### G. Vaghi^1,2^, V. Bitetto^1,2^, E. Guaschino^1^, R. De Icco^1,2^, N. Ghiotto^1^, C. Tassorelli^1,2^, G. Sances^1^

#### ^1^IRCCS Mondino Foundation, Headache Science & Neurorehabilitation Center, Pavia, Italy; ^2^University of Pavia, Department of Brain and Behavioral Sciences, Pavia, Italy

##### **Correspondence:** G. Vaghi

Backgound&Objective: Local regulations require a 3-month interruption of CRGP antibodies treatment in migraine after a 12-month course. Limited data are available on the persistence of their effect after interruption. We report migraine pattern during suspension period in chronic migraine patients (CM)

Methods: We analyzed 65CM patients: F45, M20, mean age: 49.2+9.3, treated with erenumab for over a year (mean duration 17±5.6months) before the mandatory suspension. We evaluated changes in monthly headache days (MHD), related disability (MIDAS), monthly medication doses (MMD) and days of drug intake (DDI) at baseline (T_0_), end of treatment (T_end_) and during suspension.

Results: MHD significantly improved at T_end_ compared to baseline (T_0_23.6+5.5, T_end_10.1+7), as did MDD (T_0_30.1+25.3, T_end_8.2+5.8) and DDI (T_0_19.7+7.7, T_end_7.1+4.3), p<0.001. All parameters significantly worsened already in the 1^st^month of suspension when compared to T_end_, p<0.01(Fig. 1) and maintained a worsening pattern over the subsequent 2months. Though significantly worse, the clinical condition observed in the last month of suspension was still better than T_0_ values. MIDAS worsened accordingly, p<0.001 (Fig. 2; T_end_18.9+26.1, after stop 45.3+37.2)

Conclusions**:** Erenumab suspension was associated with an early and progressive worsening of headache-related parameters and disability. Regulators should consider the possibility to allow prolonged treatment in migraine subjects resistant to other preventive therapies.

**Fig. 1 (abstract P0302).**
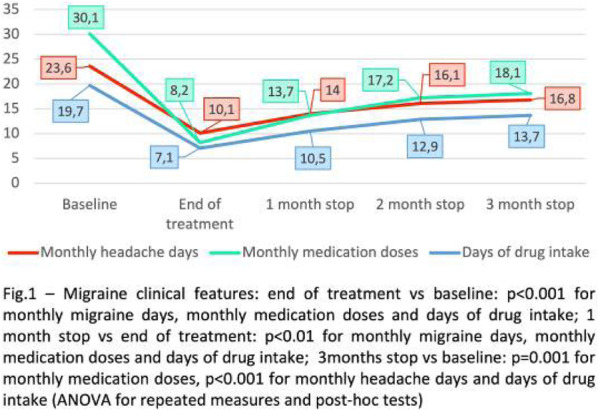
See text for description

**Fig. 2 (abstract P0302).**
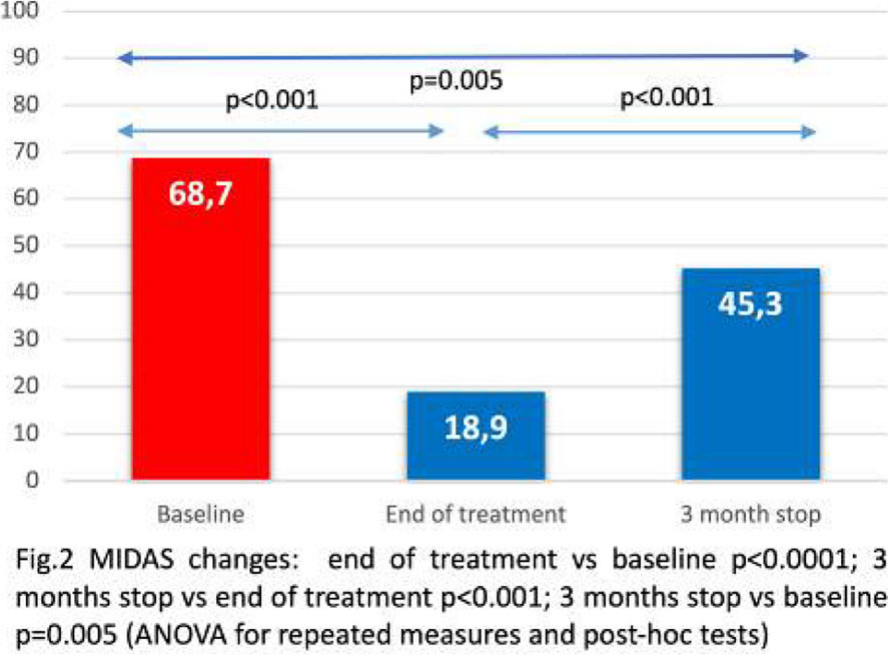
See text for description

## P0303 Empirically derived dietary patterns and their association with clinical symptoms of migraine headache

### F. Khorvash^1^, G. Askari^2^, A. Arab^2^

#### ^1^Isfahan University Of Medical Sciences, Neurology, Isfahan, Iran; ^2^Isfahan University of Medical Sciences, Community Nutrition, Isfahan, Iran

##### **Correspondence:** F. Khorvash

Objective: There has been attention in determining what factors may trigger migraine attacks, including dietary factors.The present study aimed to investigate the association between major dietary patterns and clinical symptoms of migraine.

Methods: In this cross-sectional study, 262 patients (20-50 years old) with migraine were enrolled using a simple random sampling method. The patients" dietary intake over the previous year was assessed via a validated 168-item, semi-quantitative food frequency questionnaire (FFQ). Headache diaries regarding clinical characteristics of migraine headache during the preceding month were obtained.

Results: We identified three major dietary patterns including "traditional", "western", and "healthy". After adjustment for potential confounders, greater adherence to the western dietary pattern was associated with higher headache frequency (β=6.18; 95% CI: -0.46, 12.82; P=0.068) and lower headache duration (β=-0.48; 95% CI: -0.94, -0.03; P=0.036) among patients with chronic migraine. Moreover, healthy dietary pattern tended to have decreased severity of headache (β=-0.051; 95% CI: -1.10, 0.07; P=0.089) among subjects with episodic migraine.

Conclusion: We found that a lower intake of sweets, processed meat, refined grains, and condiments, as well as a higher intake of vegetables, fruits, whole grains, and low-fat dairy products, might be associated with better clinical symptoms of migraine headache.

## P0304 Effect of synbiotic supplementation on migraine characteristics, gut permeability and inflammatory biomarker in women with migraine: Results of a randomised controlled trial

### F. Khorvash^1^, G. Askari^2^, A. Ghavami^3^, Z. Heidari^4^

#### ^1^Isfahan University Of Medical Sciences, Neurology, Isfahan, Iran; ^2^Isfahan University of Medical Sciences, Community Nutrition, Isfahan, Iran; ^3^Isfahan University of Medical Sciences, Clinical Nutrition, Isfahan, Iran; ^4^Isfahan University of Medical Sciences, Biostatistics and Epidemiology, Isfahan, Iran

##### **Correspondence:** F. Khorvash

Background: Previous studies have shown a role of gut-brain axis in migraine headaches pathogenesis. We aimed to examine the effect of synbiotic supplementation on the characteristics of migraine attacks, gut permeability and inflammation in women with migraine.

Methods: Sixty-nine migraine patients who completed this randomized double-blind controlled trial received two capsules of synbiotic or placebo. The migraine severity, frequency and duration of attacks, gastrointestinal problems, serum Hs-CRP and zonulin levels were measured at baseline and the end of the intervention.

Results: After a 12-week intervention, among participants the mean frequency of migraine attacks significantly reduced in the synbiotic group (mean change: -1.02 per month, P=0.011). Also, a non-significant reduction was also evident in the migraine severity (mean decrease: -0.17; P=0.168) and duration (mean decrease: -3.97 hours; P= 0.327) in the synbiotic group. Patients who received the synbiotic also showed significant reduction of gastrointestinal problems (P= 0.032). In contrast to the placebo, synbiotic supplementation significantly decreased the zonulin levels as gut permeability (mean change: -4.12 vs 0.85 respectively, P=0.034) and Hs-CRP levels as inflammation (mean change: -0.43 vs -0.09 respectively, P=0.022) in migraine patients.

Conclusion: The results of this study showed that the synbiotic supplementation could be an effective supplement to improve migraine headache.

## P0305 Self-reported Effect of Exercise in Patients with Migraine

### Y. E. Gil^1^, S. Cho^2^, C. S. Chung^1^, M. J. Lee^1^

#### ^1^Samsung Medical Center, Sungkyunkwan University School of Medicine, Neurology, Seoul, South Korea; ^2^Uijeongbu Eulji Medical Center, Neurology, Uijeongbu, South Korea

##### **Correspondence:** Y. E. Gil

Background and objective: Regular exercise is often recommended to patients with migraine, while it can trigger a migraine attack. We investigated self-reported effects of exercise on headache in patients with migraine.

Methods: We retrospectively reviewed a prospective headache registry of single tertiary hospital where all patients were asked about their experience regarding effects of exercise on headache. First-visit patients with migraine were included in this study and classified based on their response: headache worsening, relief, and no influence. Factors associated with headache worsening were analyzed.

Results: A total of 1828 patients were included in this study. Regarding the effect of exercise on their headache, 373 (20.4%), 571 (31.2%), and 884 (48.4%) patients reported worsening, relief, and no influence, respectively. The proportion of patients who reported worsening by exercise increased from 14.0% in low-frequency episodic migraine (EM), 21.5% in high-frequency EM, to 27.3% in chronic migraine (P for trend <0.001). Relief by exercise was not associated with migraine frequency changes (P for trend=0.318). Younger age, high frequency episodic migraine, chronic migraine, and the presence of allodynia were independently associated with headache worsening by exercise.

Conclusions: Effect of exercise on headache differs among patients. Regular exercise should be recommended not routinely but individually based on clinical features of the patient.

## P0306 1-2 Year Real World Prospective Quality of Life Data in Patients with an Abrupt Onset New Daily Persistent Headache and Chronic Migraine Phenotype Treated with Erenumab in Ireland

### A. Buture^1^, E. Tomkins^2^, A. A. Shrukalla^2^, E. Troy^2^, K. Conaty^2^, E. Macken^1^, R. Lonergan^1^, J. Melling^1^, N. Long^2^, E. Shaikh^2^, M. Ruttledge^2^

#### ^1^Mater Misericordiae University Hospital, Neurology, Dublin, Ireland; ^2^Beaumont Hospital, Neurology, Dublin, Ireland

##### **Correspondence:** A. Buture

Background: Abrupt onset of unremitting daily headache is generally refractory to conventional migraine prophylactic treatments. CGRP monoclonal antibodies have been shown to be effective in clinical trials in migraine but we are not aware of any real-world data involving the CGRP treatment in this patient group.

Objective: To prospectively assess the benefit of Erenumab on Quality of Life (QOL) in a group of patients with abrupt onset daily persistent headache who have failed multiple preventative migraine therapies.

Methods: 52 patients who received either 70mg or 140mg Erenumab every 28 days by subcutaneous injection. Patients were asked to complete migraine specific QOL questionnaires before starting treatment, and at 3-6 months intervals, up to two years after starting treatment. The migraine specific QOL questionnaires included: the Headache Impact Test-6 (HIT-6), Migraine Associated Disability Assessment (MIDAS) test and Migraine-Specific Quality-of-Life Questionnaire (MSQ).

Results: 52 patients started treatment between December 2018 and October 2019. 30 stopped treatment during the first year due to lack of benefit and/or side effects. Most patients stopped because of lack of efficacy. 22 patients had improvement in QOL measurements and stayed on treatment, between one and two years.

Conclusion: Clinically significant improvements in QOL were experienced in approximately 40% of our cohort of refractory chronic headache patients treated with Erenumab.

## P0307 Raynaud's phenomenon secondary to erenumab in a patient with chronic migraine

### A. H. Manickam^1^, A. Buture^2^, E. Tomkins^3^, M. Ruttledge^3^

#### ^1^Bharathiar University, Department of Human Genetics and Molecular Biology, Tamilnadu, India; ^2^Mater Misericordiae University Hospital, Neurology, Dublin, Ireland; ^3^Beaumont Hospital, Neurology, Dublin, Ireland

##### **Correspondence:** A. H. Manickam

Background: Calcitonin gene related peptide (CGRP) monoclonal antibodies have revolutionised the management of migraine. Although this class of drugs is generally well tolerated, new data regarding their side effects is emerging.

Case: We present the case of a 45 year old female with a long standing history of chronic migraine who failed four oral migraine prophylactic drugs (including propranolol, amitriptyline, topiramate and venlafaxine) before she was treated with erenumab 70 mg subcutaneous injection. The patient developed Raynaud's phenomenon (RP) two weeks after the second dose of erenumab, but did not initially wish to discontinue treatment as she found it very beneficial for her migraine, reporting 40% improvement in headache severity. Unfortunately, the patient discontinued treatment after eight months due to the side effect of RP.

Conclusion: This is a case report of a patient with chronic migraine, who developed RP secondary to erenumab treatment. RP secondary to CGRP monoclonal antibodies has been rarely reported in the literature previously, and it is a side effect with relevant clinical implications that could influence the initiation or cessation of these class of drugs in patients with migraine.

## P0308 Characterisation of patient and treatment profiles of migraine patients treated with erenumab in routine clinicl practice: Interim results from the SPECTRE study

### C. Gal^1^, M. Koch^2^, C. Baufeld^3^

#### ^1^Migraine and Headache Clinic Koenigstein, Koenigstein im Taunus, Germany; ^2^Novartis Pharma AG, Basel, Switzerland; ^3^Novartis Pharma GmbH, Nuremberg, Germany

##### **Correspondence:** C. Gal

Erenumab, a CGRP receptor antagonist, was the first monoclonal antibody approved for preventive migraine treatment. The SPECTRE study (characteriSation of Prescription patterns in Episodic and Chronic migraine patients starting Treatment in a Reallife setting with Erenumab in Germany) aims to better understand patient profiles and treatment patterns for erenumab in Germany based on migraine characteristics and comorbidities.

This non-interventional study is conducted at 139 centers in Germany and enrolled 572 adult migraine patients. Apart from a daily headache diary, the patient-reported-outcome (PRO) questionnaires HIT-6 and TSQM are documented at 3-month intervals.

Previous baseline analysis of a small proportion of patients showed that the majority of erenumab patients in this subgroup were women with chronic migraine, with a high proportion of psychiatric comorbidities. Here we expand this analysis to about 400 erenumab patients. Baseline characteristics, including monthly migraine days, prophylactic pretreatments and comorbidities, and 6-month follow-up data, including information from an app-based migraine diary and PRO data, will be presented.

SPECTRE will provide valuable insights into the use of erenumab in clinical practice in Germany, help characterize prescription patterns, patient profiles and analyse the respective therapy response. This will possibly allow for development of individual treatment strategies for each patient.

## P0309 Wearing-off effect of onabotulinumtoxinA: A prospective real-world study

### M. P. Navarro-Pérez^1,2^, R. Marín-Labanda^1^, S. Ballesta-Martínez^1,2^, J. Espinosa-Rueda^1,2^,E. Bellosta-Diago^1,2^, S. Santos-Lasaosa^1,2^

#### ^1^Hospital Clínico Universitario Lozano Blesa, Neurology, Zaragoza, Spain; ^2^Aragon Institute for Health Research (IIS Aragon), Zaragoza, Spain

##### **Correspondence:** M. P. Navarro-Pérez

Objective: Our aim is to assess the frequency of onabotulinumtoxinA (onabotA) wearing-off effect (WOE) in patients with chronic migraine (CM) during the first and second treatment cycles and its possible associated factors.

Methods**:** We prospectively evaluated WOE during the first two treatment cycles in patients with CM who started treatment with onabotA between January and March 2020. To assess the presence of WOE we asked patients if they had experienced a worsening of headache or migraine (frequency, intensity, duration, non-painful associated symptoms) at week 10 or week 12.

Results: 59 patients completed the study, 93.2% were women, mean age 44 years (SD 12). 45/59 (76.3%) presented medication overuse (MO) at baseline. Most patients received 195U onabotA dose [50/59 (84.7%) from treatment onset and 100% at second cycle]. WOE was present in 24/59 patients (40.6%), in 13 patients occurred after one treatment cycle and in 11 patients after the two treatment cycles. After the first treatment cycle 21/59 patients (35.6%) reported WOE, 7/59 (11.9%) at week 10 and 14/59 (23.7%) at week 12. After the second treatment cycle WOE was reported by 14/59 patients (23.8%), 7/59 (11.9%) at week 10 and 7/59 (11.9%) at week 12. Age, sex, MO, psychiatric comorbidity, onabotA dose and baseline headache characteristics did not differ between the WOE and no WOE groups.

Conclusion: WOE of onabotA is common in patients with CM in clinical practice even with 195U dose from the treatment onset.

## P0310 Role of targeting CGRP for Migraine Prevention and Challenges with Oral Therapies: A Pilot survey on knowledge, attitude & practice (KAP) in migraine prevention among Indian Neurologists

### S. Thakur^1^, A. Thorat^1^, J. Desai^2^

#### ^1^Novartis India Limited, Medical Affairs, Mumbai, India; ^2^Jaslok Hospital, Mumbai, India

##### **Correspondence:** S. Thakur

Objective: To understand Indian neurologist"s knowledge, awareness and perception regarding the challenges with oral prophylactic agents; the role of CGRP in migraine pathophysiology and possible role of anti-CGRP mAbs in migraine prophylaxis

Methodology: A nationwide cross-sectional questionnaire-based online survey was conducted among registered Indian Neurologists. The questionnaire was validated by a group of neurologists participating in the advisory board and was rolled out to a random sample of 140 neurologists and their response was anonymized. Aggregate data was summarized by percentage graphs.

Results: 47 neurologists voluntarily participated. Participating neurologists believe that most patients (61%) have ≤3months adherence to current oral prophylactics citing adverse events (33%) as common cause. Most participants believe that adverse events (76.3%), limited efficacy (73.7%) and lack of target specificity (55.3%) are the most common challenges with oral prophylactics. Most participants were aware on the role of CGRP in migraine pathophysiology (94%) and most participants perceived antagonizing CGRP would be a promising option for migraine prophylaxis (97%)

Conclusion: Results highlight that there is an unmet need with currently used oral prophylactics among neurologists who believe targeting CGRP may be a promising option. Hence anti-CGRP mAbs could be an attractive option if they can address unmet needs with current oral prophylactics. This was a pilot survey with a small sample size. Further research on clinical utility in terms of ideal patient profile, duration of therapy, etc with anti-CGRP drugs is warranted.

**Fig. 1 (abstract P0310).**
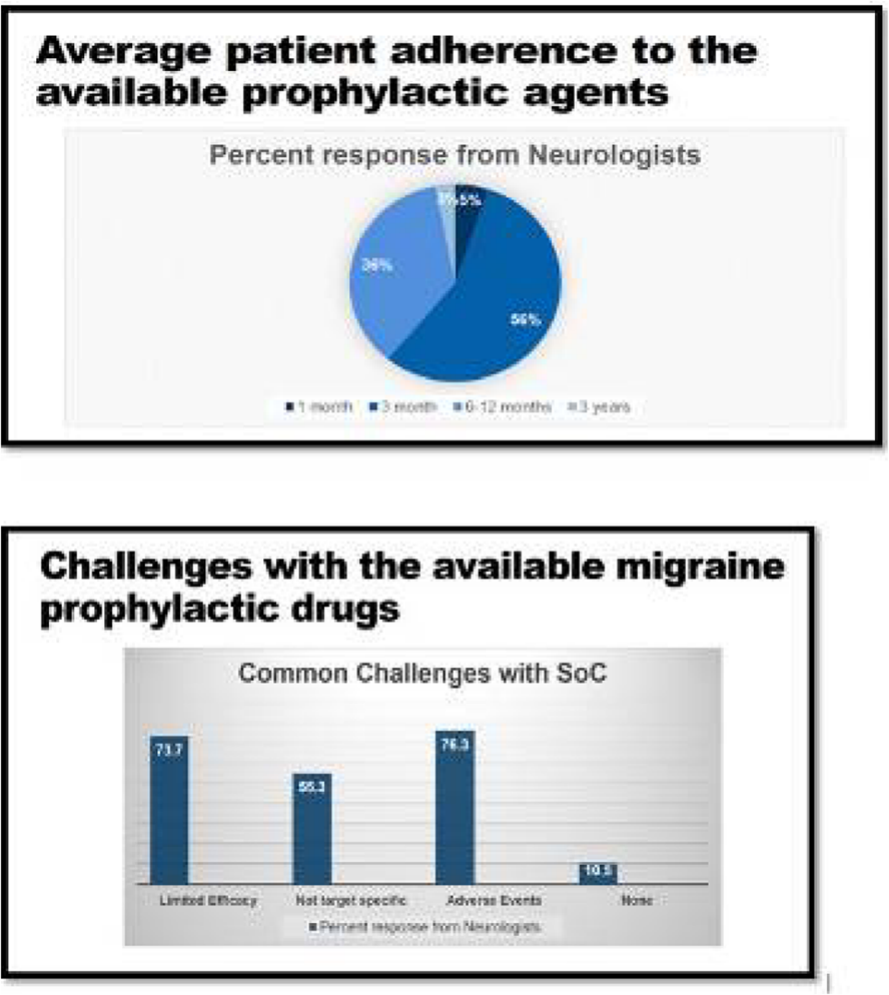
See text for description

**Fig. 2 (abstract P0310).**
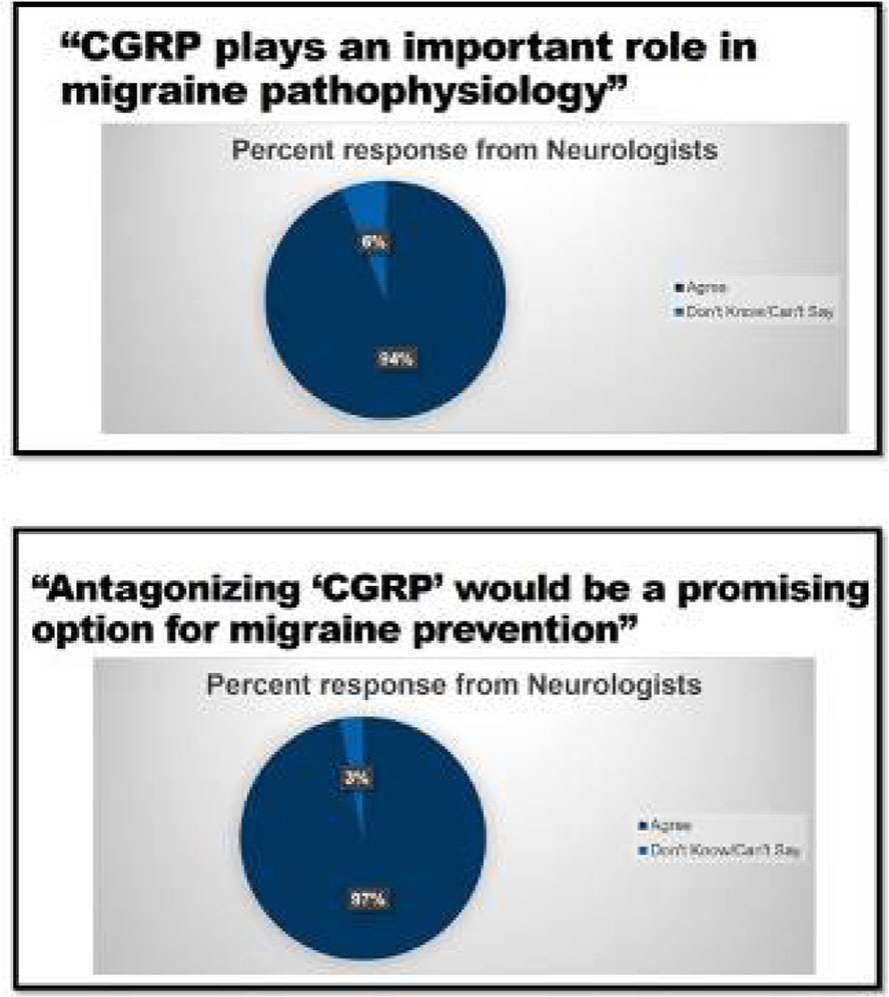
See text for description

## P0311 MIDAS score reduction in the decision-making process of erenmab treatment: pearls and pitfalls

### R. De Icco^1,2^, G. Vaghi^1,2^, M. Allena^1^, E. Guaschino^1^, V. Bitetto^1^, D. Martinelli^1,2^, A. Putortì^1,2^, S. Bottiroli^1,3^, N. Ghiotto^1^, G. Sances^1^, C. Tassorelli^1,2^

#### ^1^IRCCS Mondino Foundation, Headache Science and Neurorehabilitation Center, Pavia, Italy; ^2^University of Pavia, Department of Brain and Behavioral Sciences, Pavia, Italy; ^3^Giustino Fortunato University, Faculty of Law, Benevento, Italy

##### **Correspondence:** R. De Icco

Objective: The Italian Medicines Agency requires discontinuation of anti-CGRP monoclonal antibodies in migraine patients not achieving a 50% reduction in MIDAS score (improvement) at 3 (3M) and 6 months (6M). This decision-making step is based on expert opinion. Our aim is to evaluate the predictive role of MIDAS on long-term erenumab efficacy.

Methods: Seventy-five patients with chronic migraine and medication overuse (49.5±9.4 years, 53 females, 22.7±5.9 monthly migraine days - MMD) received erenumab treatment for one year (140 mg s.c. – 13 administrations, 28 days apart) without interruptions. MIDAS improvement at 3M and 6M was tested as a predictor of erenumab response in terms of: i) MMD, and ii) rate of 50% Responders.

Results: Better results in average MMD reduction at the end of treatment were observed in patients with MIDAS improvement at 3M (9.6 - C.I. 7.6-11.5 vs. 13.4 - C.I. 11.2-15.6, p=0.012), but not at 6M (10.0 - C.I. 8.1-11.9 vs. 13.0 – C.I. 10.7-15.4, p=0.055). MIDAS improvement at 3M was associated with a higher percentage of 50% Responders at the end of treatment (76.2% vs. 53.2%, p=0.026), but the same was not true for MIDAS improvement at 6M (p=0.342).

Conclusion**:** A 50% reduction in MIDAS score represents a good predictor of clinical outcome after 1-year treatment with erenumab when evaluated at M3, but not at M6. Patients discontinued from erenumab at M6 due to a lacking 50% reduction in MIDAS score may still benefit from erenumab administration.

## P0312 Patients With Migraine Who Achieved a ≥75% Reduction in Monthly Migraine Days With Eptinezumab Treatment: Subgroup Analysis of PROMISE-1 and PROMISE-2

### R. B. Lipton^1^, L. Charleston IV^2^, C. Tassorelli^3^, T. Brevig^4^, J. Hirman^5^, R. Cady^6^

#### ^1^Department of Neurology, Albert Einstein College of Medicine, Bronx, NY, United States; ^2^Department of Neurology and Ophthalmology, Michigan State University, East Lansing, MI, United States; ^3^IRCCS C. Mondino Foundation, Pavia, Italy; ^4^H. Lundbeck A/S, Copenhagen, Denmark; ^5^Pacific Northwest Statistical Consulting, Inc., Woodinville, WA, United States; ^6^Lundbeck La Jolla Research Center, San Diego, CA, United States

##### **Correspondence:** R. B. Lipton

Objective**:** To evaluate patients with episodic (EM) or chronic migraine (CM) achieving a ≥75% migraine responder rate (MRR) over weeks 1–12 (Wk1-12) with eptinezumab vs placebo (pbo), the consistency of that response, and its impact on patient-reported outcomes (PROs).

Methods: Patients with EM (PROMISE-1) or CM (PROMISE-2) treated with eptinezumab 100mg, 300mg, or pbo and experiencing ≥75% MRR over Wk1-12 were included. PROs (from PROMISE-2) included 6-item Headache Impact Test (HIT-6), patient-identified most bothersome symptom (PI-MBS), and Patient Global Impression of Change (PGIC).

Results: PROMISE-1 ≥75% MRRs over Wk1-12 were 22.2% (100mg, *P*=0.1126), 29.7% (300mg, *P*=0.0007), and 16.2% (pbo); PROMISE-2 ≥75% MRRs were 26.7% (*P*=0.0001), 33.1% (*P*<0.0001), and 15.0%. Once ≥75% MRR over Wk1-12 was achieved, >70% of EM and >80% of CM patients maintained ≥75% MRR over subsequent doses across groups. In CM patients with ≥75% MRR over Wk1-12, mean change in Wk12 HIT-6 total score with eptinezumab (pooled) was –11.7, with 64.4% reporting little to no/some life impact and >80% reporting much/very much improved on PI-MBS and PGIC.

Conclusion: More eptinezumab-treated patients achieved ≥75% MRR over Wk1-12 vs pbo across patients with migraine, with response primarily consistent across the 24-week treatment period. For CM patients achieving ≥75 MRR, PRO results indicated substantial improvements in headache-related impact and symptoms.

## P0313 Reductions in Migraine Frequency With Fremanezumab Treatment in Individuals With Chronic and Episodic Migraine

### S. J. Nahas^1^, X. Ning^2^, J. M. Cohen^2^, S. Barash^2^, V. Ramirez Campos^2^, S. D. Silberstein^1^

#### ^1^Thomas Jefferson University, Philadelphia, PA, United States; ^2^Teva Branded Pharmaceutical Products R&D, Inc., West Chester, PA, United States

##### **Correspondence:** S. J. Nahas

Objective: People with migraine who have more frequent attacks may have greater disease burden. This pooled analysis assessed the shift in migraine frequency category for participants (pts) treated with fremanezumab from three phase 3, double-blind, placebo (PBO)-controlled trials (HALO CM, HALO EM, and FOCUS).

Methods: In all 3 studies, pts with chronic or episodic migraine (CM/EM) were randomized 1:1:1 to quarterly (QTY) fremanezumab, monthly (MLY) fremanezumab, or matched PBO. The percentages of pts with a shift of ≥1 category down during 12 weeks of treatment were evaluated by baseline (BL) frequency category (high-frequency CM [HFCM; ≥19 monthly migraine days (MMD)]; low-frequency CM [LFCM; 15-18 MMD]; high-frequency EM [HFEM; 10-14 MMD]; moderate-frequency [MFEM; 4-9 MMD]).

Results: At BL, 659 pts had LFEM, 515 had HFEM, 511 had LFCM, and 500 had HFCM. Higher proportions of pts with MFEM receiving QTY (53%) and MLY (52%) fremanezumab experienced a shift of 1 category down to LFEM (<4 MMD) versus PBO (29%). Higher proportions of pts receiving QTY and MLY fremanezumab versus PBO experienced a shift of ≥1 category down in the BL HFEM subgroup to MFEM or LFEM (QTY, 77%; MLY, 75%; PBO, 58%), the BL LFCM subgroup to HFEM, MFEM, or LFEM (QTY, 73%; MLY, 76%; PBO, 57%), or the BL HFCM subgroup to LFCM, HFEM, MFEM, or LFEM (QTY, 57%; MLY, 59%; PBO, 44%).

Conclusion: Both QTY and MLY fremanezumab resulted in favorable migraine frequency category shifts to a greater extent than PBO.

## P0314 Impact of Fremanezumab Treatment on Clinical Outcomes Among Migraine Patients With Comorbid Depression, Anxiety or Hypertension in a Real-World Setting

### K. Tangirala^1^, V. Pedarla^2^, M. Driessen^3^, L. J. Krasenbaum^1^, S. F. Thompson^1^, K. Maughn^2^, M. J. Seminerio^1^, J. M. Cohen^1^

#### ^1^Teva Branded Pharmaceutical Products R&D, Inc., West Chester, PA, United States; ^2^STATinMED Research, Plano, TX, United States; ^3^Teva Pharmaceuticals, Amsterdam, Netherlands

##### **Correspondence:** K. Tangirala

Objective: To evaluate real-world impact of fremanezumab in patients (pts) with migraine and comorbid depression (DEP), anxiety (ANX), or hypertension (HTN).

Methods: Data for adults with ≥1 migraine diagnosis and a medication record for fremanezumab were obtained from the Veradigm Health Insights Database (study period, 1/1/2014–6/30/2019; identification period, 9/1–12/31/2018). Changes in mean antidepressant prescription (AD) and anxiolytic prescription (AX) use from the 6-month baseline period to 6 months after fremanezumab initiation were assessed by comorbidity. For the comorbid HTN subgroup, changes in systolic and diastolic blood pressure (SBP/DBP) were analyzed.

Results: For the DEP (n=172) subgroup, proportion of pts with AD (−12.2%; P=0.003) and number of AD used (−0.2; *P*=0.008) were statistically significantly reduced with fremanezumab treatment. For the ANX subgroup (n=180), fremanezumab treatment resulted in significant reductions in proportion of pts with AX (−7.8%; *P*=0.037) and nonsignificant reductions in number of AX used (−0.1; *P*=0.182). Among those with HTN (n=142), fremanezumab treatment resulted in nonsignificant reductions in SBP (–0.34mmHg) and DBP (–0.59mmHg; both *P*>0.05).

Conclusions: Preventive fremanezumab treatment in migraine pts with comorbid DEP or ANX resulted in significant decreases in proportions of pts with AD and AX use, suggesting possible improvement in these comorbidities. Among pts with HTN, nonsignificant reductions in SBP and DBP were observed.

## P0315 Long-term Efficacy of Fremanezumab in Patients With Chronic or Episodic Migraine and Documented Prior Inadequate Response to 2-4 Classes of Migraine Preventive Medications

### S. Nägel^1^, J. M. Cohen^2^, V. Ramirez Campos^2^, S. Barash^2^, X. Ning^2^, D. Kudrow^3^

#### ^1^Department of Neurology, Martin Luther University Halle, University Hospital Halle (Saale), Halle, Germany; ^2^Teva Branded Pharmaceutical Products R&D, Inc., West Chester, PA, United States; ^3^California Medical Clinic for Headache, Santa Monica, CA, United States

##### **Correspondence:** S. Nägel

Objective: To evaluate long-term efficacy of fremanezumab in patients (pts) with chronic or episodic migraine (CM/EM) and prior inadequate response to 2-4 preventive classes during 12-week double-blind period (DBP) and open-label extension (OLE) of phase 3b FOCUS study.

Methods: After 28-day baseline period, pts were randomized (1:1:1) to quarterly (QTY) or monthly (MLY) fremanezumab or placebo (PBO) for DBP. Pts completing DBP entered OLE. Pts on MLY fremanezumab continued MLY dosing; pts on QTY or PBO switched to MLY. Outcomes are summarized by DBP group.

Results: During DBP, least squares mean changes in monthly migraine days (MMD) in PBO, QTY, and MLY fremanezumab groups, respectively, were –0.8, –3.9, and –4.5 for CM pts (n=509) and –0.6, –3.7, and –3.8 for EM pts (n=328; *P*<0.0001 vs PBO); during OLE, mean MMD changes were –5.3, –5.1, and –5.8 for CM pts (n=493) and –3.9, –5.1, and –5.1 for EM pts (n=313). Reductions were also observed for CM and EM pts in monthly headache days of at least moderate severity during DBP and increased during OLE. In PBO, QTY, and MLY fremanezumab groups, respectively, for DBP, ≥50% reduction in MMD were 8%, 27%, and 29% for CM pts and 10%, 47%, and 43% for EM pts (*P*<0.0001 vs PBO) and for OLE, were 31%, 35%, and 40% for CM pts and 49%, 63%, and 56% for EM pts.

Conclusions: Fremanezumab demonstrated long-term efficacy in CM and EM pts with prior inadequate response to multiple preventive classes, including those switching from QTY to MLY fremanezumab.

## P0316 Responder Rates for Reductions in Nausea or Vomiting and Photophobia and Phonophobia in Patients Treated With Fremanezumab in the HALO CM, HALO EM, HALO LTS, and FOCUS Studies

### P. McAllister^1^, J. M. Cohen^2^, X. Ning^2^, V. Ramirez Campos^2^, L. Janka^2^

#### ^1^New England Institute for Neurology and Headache, Stamford, CT, United States; ^2^Teva Branded Pharmaceutical Products R&D, Inc., West Chester, PA, United States

##### **Correspondence:** P. McAllister

Objective: This post hoc analysis evaluated ≥50% reductions in days with nausea or vomiting (N/V) and photophobia and phonophobia (P/P) using data from HALO (chronic migraine [CM], episodic migraine [EM], and long-term safety [LTS]) and FOCUS studies.

Methods: Patients (pts) were randomized 1:1:1 to quarterly (QTY) fremanezumab, monthly (MLY) fremanezumab, or placebo (PBO) over 3 months (mo) in HALO CM, HALO EM, and FOCUS. Pts continued or were randomized 1:1 to QTY or MLY fremanezumab over 12 mo in HALO LTS. Proportions of pts with ≥50% reductions in monthly average days with N/V and P/P over 3 mo in HALO CM, EM, and FOCUS and at mo 12 in HALO LTS (≥50% N/V and P/P response) were evaluated.

Results: In HALO CM (N=1,121), significantly higher proportions of pts achieved ≥50% N/V response with fremanezumab (QTY, 45%; MLY, 43%) versus PBO (27%; *P*<0.0001) and ≥50% P/P response (QTY, 34%; MLY, 35% vs PBO, 26%; *P*≤0.0143). With QTY and MLY fremanezumab, respectively, vs PBO, significantly higher proportions of pts achieved ≥50% N/V response (45% and 47% vs 32%; *P*<0.0007) and ≥50% P/P response (43% and 41% vs 31%; *P*<0.0115) in HALO EM (N=865). Similar ≥50% N/V and P/P response rates were observed in FOCUS (N=837), and response rates increased over 12 mo of fremanezumab treatment in HALO LTS (N=1,103).

Conclusions: With both fremanezumab regimens, many patients achieved clinically meaningful reductions in days with nausea/vomiting and photophobia/phonophobia over up to 12 months.

## P0317 Design and population characteristics of APOLLON

### S. Ortler^1^, H. Göbel^2^, M. Maier-Peuschel^1^, M. Koch^3^

#### ^1^Novartis Pharma GmbH, Nürnberg, Germany; ^2^Schmerzklinik Kiel, Migräne- und Kopfschmerzzentrum, Kiel, Germany; ^3^Novartis Pharma AG, Basel, Switzerland

##### **Correspondence:** S. Ortler

Erenumab is the first EMA and FDA approved monoclonal antibody targeting the CGRP-receptor specifically developed for preventive migraine treatment. Recently, 5-year data from an open-label treatment phase confirmed the long-term safety profile of erenumab in an international cohort. However, long-term data on safety and efficacy of erenumab is still limited for the German population. Further, the impact and relevance of a drug holiday in the erenumab treatment should be investigated.

APOLLON is a 128-week open-label study of erenumab treatment, assessing long-term safety and tolerability in migraine patients in Germany who previously participated in a head-to-head trial of erenumab and topiramate (HER-MES, NCT03828539). The treating physician can change the erenumab dose according to the approved label or initiate a drug holiday. Thereby, impact of treatment discontinuation on monthly migraine days is assessed prior to, during and after the medication-free epoch.

Detailed study design and results of the first interim analysis describing the baseline characteristics of the total study population of 701 enrolled patients will be presented.

This analysis will provide insights into the patient population enrolled in the APOLLON study to assess long-term safety and tolerability of erenumab. Common treatment algorithms will be elucidated by this trial investigating the impact of drug holidays during preventive migraine treatment in the participating 80 headache centers.

## P0318 Fremanezumab in the prevention of high-frequency episodic and chronic migraine: FRIEND (FRemanezumab In rEal world stuDy), the first Italian multicenter, prospective real-life study

### L. Fofi^1^, G. Egeo^1^, C. Aurilia^1^, C. M. Costa^2^, C. Altamura^2^, F. Vernieri^2^, M. Albanese^3^, F. D'Onofrio^4^, L. Di Clemente^5^, M. Zucco^5^, P. Di Fiore^6^, F. Frediani^6^, R. Messina^7^, B. Colombo^7^, M. Filippi^7^, F. Bono^8^, L. Manzo^8^, A. Carnevale^9^, P. Barbanti^1^, S. Proietti^10^, S. Bonassi^10^

#### ^1^IRCCS San Raffaele, Headache and Pain Unit, Rome, Italy; ^2^Campus-Bio Medico University, Neurology Unit, Rome, Italy; ^3^University of Rome Tor Vergata, Neurology Unit, Rome, Italy; ^4^San G. Moscati Hospital, Institute of Neurology, Avellino, Italy; ^5^San Camillo-Forlanini Hospital, Department of Neurosciences, Rome, Italy; ^6^San Carlo Borromeo, ASST Santi Paolo e Carlo, Headache center, Neurology & Stroke Unit, Milan, Italy; ^7^Vita-Salute San Raffaele University-Ospedale San Raffaele, Department of Neurology, Milan, Italy; ^8^Magna Graecia University of Catanzaro, Neurology Unit, Department of Medical and Surgical Sciences, Catanzaro, Italy; ^9^San Filippo Neri Hospital, Department of Neurology, Rome, Italy; ^10^IRCCS San Raffaele Pisana, Unit of Clinical and Molecular Epidemiology; San Raffaele University, Rome, Italy

##### **Correspondence:** L. Fofi

Objective**:** We assessed fremanezumab effectiveness, safety and tolerability in high-frequency EM (HFEM) and CM in a real-life population.

Methods: This is a 24-week, multicenter (n=9), longitudinal, cohort, real life study performed from 28/01/020 to 15/03/2021. We considered all consecutive patients with HFEM or CM aged 18-65 years. Change in monthly migraine days (MMD) at weeks 21-24 compared to baseline was the primary efficacy endpoint. Secondary endpoints encompassed variation in monthly analgesic intake and change in VAS, HIT-6 and MIDAS scores during the same time interval.

Results**:** 47 patients received >1 fremanezumab dose (225 mg monthly, n=38; 625 mg quarterly, n=9). Thirty-one patients were treated for 24 weeks and considered for effectiveness analysis. From baseline to weeks 21-24, fremanezumab treatment induced a significant reduction in MMD (-8.9±5.3,p<0.001), analgesic intake

(-12.7±9.8,p<0.001), and VAS (-3.0±2.6,p<0.001),

HIT-6 (-13.8±9.5,p<0.001) and MIDAS scores

(-69.7±57.5,p<0.001). 4 patients (8.5%) presented adverse events: injection site erythema (2), orticaroid reaction (2), abdominal colic (1). Only 1 patient discontinued for ineffectiveness.

Conclusions**:** 24-week fremanezumab treatment provides effectiveness, safety and tolerability in real-life patients with HFEM or CM with >3 prior preventive treatment failures. Our data need to be confirmed in larger studies.

## P0319 Efficacy and Safety of Erenumab in Patients with Episodic Migraine in East Asia: Taiwan and Korea subpopulation analysis of the EMPOwER study

### S. J. Wang^1,2^, B. K. Kim^3^, G. Paiva Da Silva Lima^4^, S. Pandhi^5^, S. Wen^6^, S. Mondal^7^, P. Hours-Zesiger^5^

#### ^1^Taipei Veterans General Hospital, Taipei City, Taiwan; ^2^National Yang-Ming University, Taipei City, Taiwan; ^3^Eulji University, Seoul, South Korea; ^4^Amgen Inc, Thousand Oaks, CA, United States; ^5^Novartis Pharma AG, Basel, Switzerland; ^6^Novartis Pharmaceuticals Corporation, East Hanover, NJ, United States; ^7^Novartis Healthcare Pvt. Ltd., Hyderabad, India

##### **Correspondence:** S. J. Wang

EMPOwER (NCT03333109), a 3-month, double-blind, randomised study, evaluated the efficacy and safety of erenumab in adult patients with episodic migraine (EM) from Asia, the Middle East, and Latin America. The results from the subpopulation analysis of Taiwan and Korea are reported here.

Overall, 249 randomised patients received placebo (PBO), erenumab 70mg or 140mg (3:3:2) for 3 months. The primary endpoint was change from baseline in monthly migraine days (MMD). Secondary endpoints assessed were achievement of ≥50% reduction in MMD, change in monthly acute migraine-specific medication treatment days (MSMD), Headache impact test (HIT-6™) scores and safety. Assessments were done over the last month (Month 3) of the double-blind treatment period.

At baseline, mean (standard deviation) age was 40.4 (10.3) years, 79.1% of patients were female and the mean MMD was 7.94 (2.39). At Month 3, a statistically significant reduction from baseline in mean MMD (**Figure**) was observed with erenumab compared with PBO; similarly, a higher proportion of patients achieved ≥50% reduction in MMD and greater reductions in MSMD and HIT-6™ score were reported with erenumab versus PBO (**Table**). The safety profile of erenumab was in line with that of the global population with no newly-emergent safety signals.

The EMPOwER study confirms the efficacy and safety of erenumab 70mg and 140mg in adult patients with EM from Taiwan and Korea, consistent with results from the global population.

**Fig. 1 (abstract P0319).**
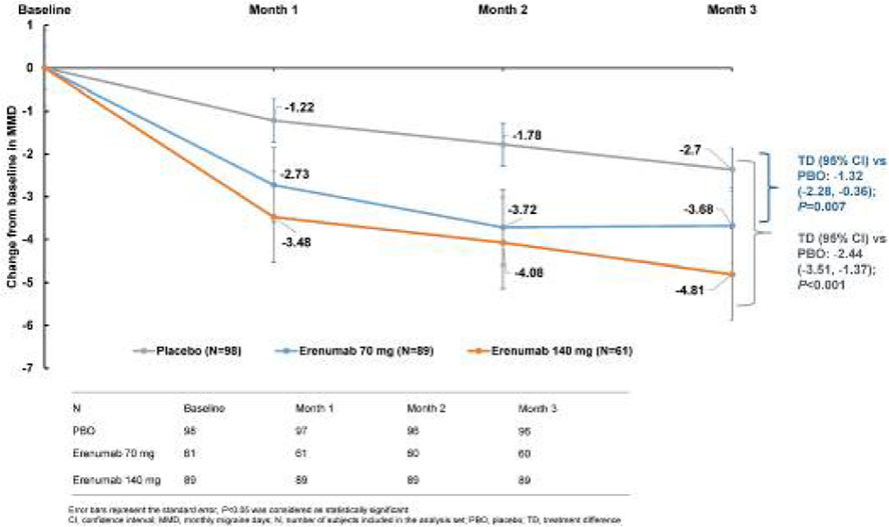
See text for description

**Fig. 2 (abstract P0319).**
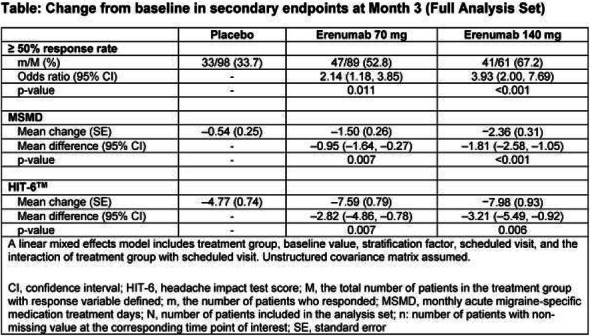
See text for description

## P0320 Pooled Analysis of the Effectiveness of Fremanezumab in Patients With Lower- and Higher-frequency Chronic Migraine

### P. McAllister^1^, J. M. Cohen^2^, X. Ning^2^, V. Ramirez Campos^2^, S. Barash^2^, M. Ashina^3^

#### ^1^New England Institute for Neurology and Headache, Stamford, CT, United States; ^2^Teva Branded Pharmaceutical Products R&D, Inc., West Chester, PA, United States; ^3^Danish Headache Center, Department of Neurology, Rigshospitalet, Glostrup, Denmark

##### **Correspondence:** P. McAllister

Objective: As chronic migraine (CM) becomes more burdensome with increasing frequency of migraine attacks, the efficacy of fremanezumab, a fully-humanized monoclonal antibody (IgG2Δa) that selectively targets calcitonin gene-related peptide (CGRP), was evaluated in patients with higher-frequency CM (HFCM; ≥19 migraine days/month) and lower-frequency CM (LFCM; 15-18 days/month).

Methods: Data were pooled from 2 double-blind phase 3 trials in CM patients (HALO CM and FOCUS). Patients were randomized (1:1:1) to quarterly fremanezumab, monthly fremanezumab, or placebo. Change from baseline (BL) in average monthly migraine days (MMDs) and monthly headache days (MHDs) of at least moderate severity were evaluated, along with the proportion of patients with ≥50% reduction in MMDs.

Results: In the quarterly or monthly fremanezumab groups, respectively, versus placebo for patients with LFCM (n=511) and HFCM (n=500), significantly greater least-squares mean reductions from BL were observed in MMDs (LFCM: −5.9 and −5.9 vs −3.2; HFCM: −4.6 and −5.5 vs −2.9; *P*≤0.0078) and MHDs (LFCM: −4.9 and −5.3 vs −2.2; HFCM, −4.6 and −5.4 vs −2.5; *P*≤0.0007). In the LFCM and HFCM subgroups, respectively, the proportion of patients with ≥50% reduction in MMDs was significantly greater with quarterly (34% and 18%) and monthly fremanezumab (31% and 23%) versus placebo (16% and 10%; *P*≤0.0464).

Conclusions: These results demonstrate fremanezumab to be effective for CM, regardless of baseline migraine frequency.

## P0321 Pooled Analysis of the Efficacy of Fremanezumab in Persons With Moderate- and Higher-frequency Episodic Migraine

### S. J. Nahas^1^, X. Ning^2^, J. M. Cohen^2^, S. Barash^2^, V. Ramirez Campos^2^, S. D. Silberstein^1^

#### ^1^Thomas Jefferson University, Jefferson Headache Center, Philadelphia, PA, United States; ^2^Teva Branded Pharmaceutical Products R&D, Inc., West Chester, PA, United States

##### **Correspondence:** S. J. Nahas

Objective: As persons with episodic migraine (EM) experience greater disease burden with more frequent attacks, the efficacy of fremanezumab, a fully-humanized monoclonal antibody (IgG2Δa) that selectively targets calcitonin gene-related peptide, was evaluated in persons with higher-frequency EM (HFEM; 10-14 migraine days/month) and moderate-frequency EM (MFEM; 4-9 days/month).

Methods: In 2 double-blind phase 3 studies (HALO EM and FOCUS) included in this analysis, participants were randomized (1:1:1) to quarterly fremanezumab, monthly fremanezumab, or placebo. Change from baseline (BL) in average monthly migraine days (MMDs) and monthly headache days (MHDs) of at least moderate severity were evaluated, along with the proportion of participants with ≥50% reduction in MMDs.

Results: In MFEM (n=659) and HFEM (n=515) subgroups, significantly greater least-squares mean reductions from BL were seen with quarterly and monthly fremanezumab, respectively, versus placebo in MMDs (MFEM: −3.5 and −3.4 vs −1.5; HFEM: −4.2 and −4.7 vs −2.8; *P*≤0.0009) and MHDs (MFEM: −3.0 and −2.8 vs −0.8; HFEM: −3.5 and −3.7 vs −1.8; *P*<0.0001). In MFEM and HFEM subgroups, respectively, the proportion of participants with ≥50% reduction in MMDs was also significantly higher with quarterly (51% and 38%) and monthly (50% and 42%) fremanezumab versus placebo (25% and 21%; *P*≤0.0005).

Conclusions: These results demonstrate fremanezumab to be effective for EM, regardless of baseline migraine frequency.

## P0322 Changes in cerebral blood flow after erenumab treatment – a prospective study of migraine patients

### M. Nowaczewska^1^, M. Straburzyński^2^, G. Meder^3^, W. Kaźmierczak^1^

#### ^1^L.Rydygier Collegium Medicum Nicolaus Copernicus University, otolaryngology, Bydgoszcz, Poland; ^2^Headache Clinic—Terapia Neurologiczna Samodzielni, Warsaw, Poland; ^3^Jan Biziel University Hospital No. 2, Department of Interventional Radiology, Bydgoszcz, Poland

##### **Correspondence:** M. Nowaczewska

Objective: The aim of this study was to check if erenumab treatment induce cerebral blood flow( CBF) changes reflected by transcranial Doppler (TCD) and whether there is a correlation between migraine, TCD parameters and treatment outcome.Methods: This prospective study involved migraineurs qualified to erenumab treatment. Patients underwent clinical and TCD evaluations before first erenumab injection and during the 6^th^ week of treatment. Data on migraine type, monthly migraine days (MMD), medication overuse headache (MOH), mean blood flow velocity (Vm) and pulsatility index (PI) in cerebral arteries were collected before and after the treatment.Patients reporting ≥ 50% reduction in MMD were defined as good responders. Results:Thirty woman were enrolled,mean age was 40.53 years,twenty with chronic migraine, fourteen with MOH.19 patients were good responders.Pretreatment Vm value in right cerebral arteries and basilar artery were significantly lower in good responders as compared with non responders.Vm values in all arteries significantly increased after the treatment as compared with baseline values,but only in a good responders,while PI remained unchanged.A significant negative correlation was observed between baseline Vm in right cerebral arteries and treatment effectiveness. Conclusion: Good response to erenumab is associated with a significant increase of Vm in brain arteries, which may reflect CBF increase. Lower baseline Vm in right cerebral arteries predict erenumab efficacy.

## P0323 Time Gained With Long-term Fremanezumab Treatment in Patients With Chronic and Episodic Migraine

### M. Marmura^1^, J. M. Cohen^2^, X. Ning^2^, V. Ramirez Campos^2^, S. Barash^2^, P. J. Goadsby^3^

#### ^1^Thomas Jefferson University, Jefferson Headache Center, Philadelphia, PA, United States; ^2^Teva Branded Pharmaceutical Products R&D, Inc., West Chester, PA, United States; ^3^King's College Hospital, London, United Kingdom and University of California, Los Angeles, CA, United States

##### **Correspondence:** M. Marmura

Objective: This posthoc analysis assessed headache-free days (HFD) and migraine-free days (MFD) gained in patients using fremanezumab from a 1-year (yr) extension study (HALO LTS).

Methods: Patients continued or were randomized 1:1 to quarterly (QTY) or monthly (MLY) fremanezumab. Expected number of headache days of any severity (HD; duration ≥4 consecutive hours [hr] or acute migraine-specific medication use) and migraine days (MD; ≥4 consecutive hr [chronic migraine (CM)] or ≥2 consecutive hr [episodic migraine (EM)] meeting criteria for migraine/probable migraine or acute migraine-specific medication use) over 1 yr were calculated using baseline numbers (extrapolated to 1 yr). Values were compared with actual MD/HD numbers observed with 1 yr of fremanezumab treatment in HALO LTS. No statistical testing was performed.

Results: For CM patients, mean expected/actual MD over 1 yr were 214/134 (80 MFD gain) in QTY fremanezumab group and 214/124 (91 MFD gain) in MLY fremanezumab group; mean expected/actual HD were 211/134 (78 HFD gain) and 212/126 (86 HFD gain), respectively. For EM patients, mean expected/actual MD were 120/55 (65 MFD gain) in QTY group and 119/57 (62 MFD gain) in MLY group; mean expected/actual HD were 112/53 (58 HFD gain) and 111/57 (54 HFD gain), respectively.

Conclusion: Over 1 year of treatment, CM patients receiving fremanezumab may gain 2.5-3 months of migraine-free/headache-free days and EM patients may gain 1.5-2 months, reducing overall migraine burden.

## P0325 FINESSE: Fremanezumab for Preventive Treatment in Migraine

### A. Straube^1^, G. Broessner^2^, C. Gaul^3^, X. Hamann^4^, T. Kraya^5^, I. Schauerte^6^, L. Neeb^7^

#### ^1^Ludwig Maximilians University, Neurology, Munich, Germany; ^2^Medizinische Universität Innsbruck, Neurology, Innsbruck, Austria; ^3^Migräne- und Kopfschmerz-Klinik Königstein, Koenigstein im Taunus, Germany; ^4^Teva GmbH, Ulm, Germany; ^5^Klinikum St. Georg, Klinik und Poliklinik für Neurologie, Neurology, Leipzig, Germany; ^6^Institut Dr. Schauerte, Munich, Germany; ^7^Charité University Hospital Berlin, Neurology, Berlin, Germany

##### **Correspondence:** A. Straube

Objective: Effectiveness and side effects of fremanezumab, a monoclonal antibody that selectively binds calcitonin gene-related peptide (CGRP) and prevents its binding to the CGRP receptor, as preventive treatment of episodic and chronic migraine (EM, CM) during 6 months after first dose in a real-world setting.

Methods**:** Prospective, non-interventional study in adults with EM or CM in routine clinical practice. Primary endpoint: Proportion of patients reaching ≥ 50% reduction in average MDM (Migraine Days per Month) during 6 months after first dose. Relevant secondary endpoints include changes from baseline in: (1) Monthly average number of migraine days; (2) Disability scores (Migraine Disability Assessment questionnaire/MIDAS; six-item Headache Impact Test/HIT-6); (3) Days of concomitant acute migraine medication.

Results: 567 patients were included (88.0% female, 45.8 ± 12.4 years of age); 54.7% had EM, 41.6% CM. 216 had completed the 6-month visit. Table 1 shows effectiveness data for month 6, whereby more patients with EM (54.0%) than with CM (42.4%) achieved the primary endpoint. Concomitant acute migraine medication showed no relevant change.

Conclusion: 49.1% of the patients achieved ≥ 50% MDM reduction over 6 months. 38.7% reported improved MIDAS, and 36.3% improved HIT-6 scores. The study is still recruiting and a later interim analysis will reveal further information.

**Fig. 1 (abstract P0325).**
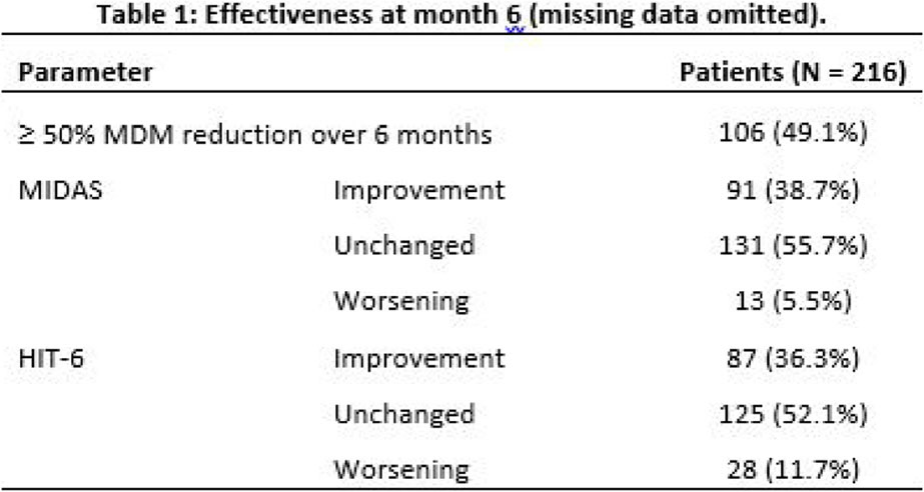
See text for description

## P0326 Occipital nerve blockade for preventive migraine treatment in pregnancy

### Â. Abreu, A. Rêgo, E. Parreira

#### Hospital Prof. Dr. Fernando Fonseca, Amadora, Portugal

##### **Correspondence:** Â. Abreu

Objective: Therapeutic options for preventive treatment of migraine during pregnancy are very limited. Although not specifically assessed in pregnancy, a safe alternative is anesthetic blockade of the great occipital nerve (GON). We present a small series of pregnant women who have underwent anesthetic blockade of GON for the treatment of migraine.

Methods: Retrospective analysis of a series of pregnant women with migraine treated with anesthetic blockade of both GON with 2% lidocaine. The assessment was based on the headache calendar, the opinion of the patients and the result of impact scales.

Results: 4 pregnant women were included, all treated in the second and third trimester. Only one had chronic migraine and analgesic abuse and the rest had episodic migraine.

There was an excellent response in 3 patients (in one with complete response after the first treatment and in the others with a significant improvement in the frequency and intensity); only one patients had no response to the first treatment and lost follow-up. In all, the procedure was well tolerated and without side effects. The blockade was effective a few days after the injection, lasting from 2 weeks to several months.

Conclusion: The blockage of the GON was safe, technically easy and efficacious in this small series. It seemed to have an effect not only as an acute treatment but also as a preventive. It should therefore be offered to all women whose migraine needs treatment during pregnancy.

## P0327 Long-term Tolerability and Improvements in Disability and Quality of Life With Fremanezumab in Patients With Chronic or Episodic Migraine and Documented Inadequate Response to 2-4 Prior Classes of Migraine Preventive Medications

### D. C. Buse^1^, J. M. Cohen^2^, V. Ramirez Campos^2^, X. Ning^2^, L. Janka^2^, L. Mechtler^3^

#### ^1^Albert Einstein College of Medicine, Bronx, NY, United States; ^2^Teva Branded Pharmaceutical Products R&D, Inc., West Chester, PA, United States; ^3^Dent Neurologic Institute, Buffalo, NY, United States

##### **Correspondence:** D. C. Buse

Objective: To evaluate tolerability, disability, and health-related quality of life (HRQoL) with fremanezumab in the 12-week double-blind period (DBP) and 12-week open-label extension (OLE) of the phase 3b FOCUS study in patients (pts) with chronic/episodic migraine (CM/EM) and inadequate response to 2-4 prior migraine preventive medication classes.

Methods: Pts (N=838) were randomized (1:1:1) to quarterly (QTY) or monthly (MLY) fremanezumab or placebo (PBO) for the DBP. Pts completing the DBP entered the OLE. Pts on MLY fremanezumab continued MLY dosing; pts on QTY fremanezumab or PBO switched to MLY dosing. Outcomes are summarized by double-blind (DB) randomization group.

Results: Across all DB randomization groups, adverse events (AEs) were reported for 40% to 56% of CM or EM pts in DBP and 49% to 60% of CM or EM pts in OLE. Serious AEs were reported for ≤2% of CM or EM pts in DBP and ≤4% of CM or EM pts in OLE (**Table 1**). During the DBP in CM or EM pts, significant improvements in disability (Migraine Disability Assessment and 6-item Headache Impact Test scores) and HRQoL (Migraine-Specific Quality of Life scores) were observed with fremanezumab during DBP. These improvements were maintained or increased during OLE (**Table 2**).

Conclusion: Fremanezumab demonstrated long-term tolerability, decreased disability, and improved HRQoL in CM and EM patients with inadequate response to 2-4 prior preventive medication classes, including those switching from QTY to MLY fremanezumab.

**Fig. 1 (abstract P0327).**
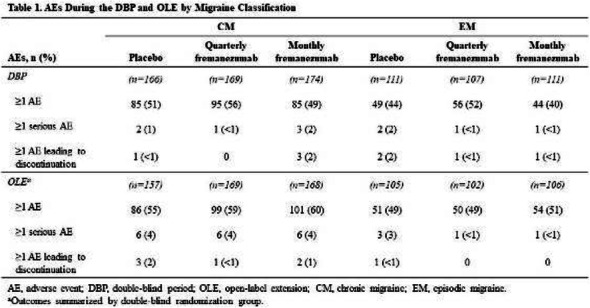
See text for description

**Fig. 2 (abstract P0327).**
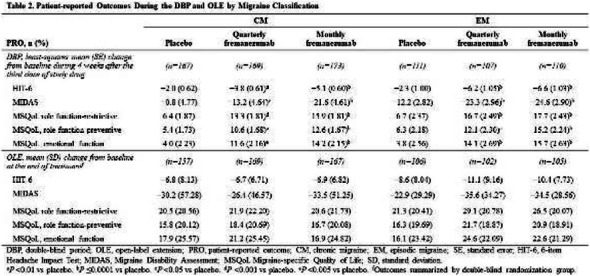
See text for description

## P0328 Effect of antibody switch in non-responders to a CGRP receptor antibody treatment in migraine: A multi-center retrospective cohort study

### L. H. Overeem^1^, A. Peikert^2^, M. D. Hofacker^1^, K. Kamm^3^, R. Ruscheweyh^3^, A. Gendolla^4^, B. Raffaelli^1^, U. Reuter^1^, L. Neeb^1^

#### ^1^Charité University Hospital Berlin, Neurology, Berlin, Germany; ^2^Neurologicum Bremen, Bremen, Germany; ^3^Ludwig Maximilians University, Neurology, Munich, Germany; ^4^Praxis Gendolla, Essen, Germany

##### **Correspondence:** L. H. Overeem

Objective: Not all patients respond to a CGRP monoclonal antibody (mAb). Non-responders to one CGRP mAb class (receptor/ligand) may benefit from a switch to the other class, but there are no efficacy data so far. We aimed to assess treatment response to a CGRP-mAb in patients that have previously failed the CGRP-receptor-mAb in a real-world analysis.

Methods: We performed a retrospective cohort study from November 2018 to May 2020 in patients who switched from erenumab to either galcanezumab or fremanezumab. Only non-responders (<30% reduction of monthly headache days (MHD) after 3 treatment cycles) were included in the analysis. MHD and acute medication days (AMD) were extracted from patient headache diaries. Endpoints were the ≥30% and ≥50% reduction of MHD, and the reduction of MHD and AMD in month 3 compared to baseline after switch. The Friedman test for repeated measures with the Dunn"s post-hoc test and Bonferroni correction were used to evaluate treatment effects.

Results: Of the 25 patients, 8 (32%) patients achieved a ≥30% reduction in MHD of which 3 (12%) achieved a ≥50% reduction after switching from the CGRP-R-mAb to a CGRP-mAb at month 3. Monthly headache days were substantially reduced in month 3 compared to baseline (20.8±7.1 to 17.8±9.1; p=0.009).

Conclusion: Our findings provide evidence that one out of three erenumab non-responders benefits from a switch to a CGRP-mAb. Switching of CGRP-mAb classes seems to be a promising treatment option in these patients.

## P0329 A novel scoring approach to identify responders to erenumab in clinical practice

### R. Messina^1^, I. Cetta^1^, B. Colombo^2^, A. Meani^3^, S. Tronci^2^, M. Filippi^1^

#### ^1^San Raffaele Scientific Institute, Neuroimaging Research Unit and Neurology Unit, Milan, Italy; ^2^San Raffaele Scientific Institute, Neurology Unit, Milan, Italy; ^3^San Raffaele Scientific Institute, Neuroimaging Research Unit, Milan, Italy

##### **Correspondence:** R. Messina

Objective: A preventive migraine drug is usually considered successful if it reduces migraine days by at least 50%. Here, we aimed to develop a multidimensional composite score that combines measures that are clinically relevant to establish migraine patients" response to erenumab.

Methods: The primary outcome of the study was erenumab efficacy, established following standard clinical evaluation. A composite treatment response score was calculated as a linear combination of response criteria evaluating significant changes in migraine frequency, headache frequency, severity of the migraine attack and migraine-related disability. Logistic regression models were run to assess the association of the composite response score, as well as different response criteria, with the primary efficacy outcome. The Brier Score and receiver-operating characteristic (ROC) analyses were performed to assess model discriminative ability.

Results: Fifty-three percent, 68% and 73% of patients achieved the primary efficacy outcome after 3, 6 and 12 months of erenumab. The composite response score achieved the lowest Brier scores at each time point, suggesting a higher predictive accuracy. Compared to the other response criteria, the composite response score had the highest AUC values at each time point.

Conclusion: Here, we proposed a simple and exhaustive multidimensional score that may facilitate patients" management in clinical practice and may expand patients" access to effective therapies.

## P0330 BoNT-A efficacy in high frequency migraine: an open label, single arm, exploratory study applying the PREEMPT paradigm

### D. Martinelli^1^, S. Arceri^2^, R. De Icco^1^, M. Allena^3^, E. Guaschino^3^, N. Ghiotto^3^, G. Castellazzi^3^, G. Cosentino^1^, G. Sances^3^, C. Tassorelli^1^

#### ^1^IRCCS Mondino Foundation, Headache Science and Rehabilitation Center / Dept of Brain and Behavioural Sciences at Pavia Univeristy, Pavia, Italy; ^2^IRCCS Fondazione Mondino, Pavia University, Dept. of brain and behavioral science, Pavia, Italy; ^3^IRCCS Mondino Foundation, Headache Science and Rehabilitation Center, Pavia, Italy

##### **Correspondence:** D. Martinelli

Background: OnabotulinumtoxinA (BoNT-A) proved effective in the prevention of chronic migraine.

Objective: In this exploratory, open label, single-arm trial (NCT04578782) we evaluated the efficacy and safety of BoNT-A (Allergan-AbbVie) in the prevention of high-frequency episodic migraine (HFEM, 8-14 migraine days/month).

Methods: We enrolled 32 HFEM subjects (age 44.8±11.9 yrs, 11.0±2.2 migraine days,11.5±2.1 headache days, 7 females). After a 28-day baseline period, subjects underwent 4 subsequent BoNT-A treatments according to the PREEMPT paradigm, every 12 weeks. The primary outcome was the monthly migraine days (MMD) reduction in the 12-week period after the last BoNT-A treatment as compared to baseline.

Results: BoNT-A reduced the number of MMD by 3.68 days (-33.1%, p<0.01). 39 % of the patients experienced a >50% reduction in MMD. BoNT-A significantly reduced also the number of headache days (-33.9%, p<0.01) and the acute medications intake (-22.9%, p=0.03). Disability and QoL scores improved markedly (MIDAS -41.7%, p<0.01 and MSQ -31.7%, p<0.01). Adverse events were transient and mild-to-moderate in severity. One patient discontinued the study due to a cutaneous adverse reaction.

Conclusions: BoNT-A administered according to the PREMPT paradigm proved effective in the prevention of HFEM paving the way to important clinical implications since HFEM subjects are at high risk of chronification.

This is an investigator-initiated trial partially supported by Allergan–Abbvie

**Fig. 1 (abstract P0330).**
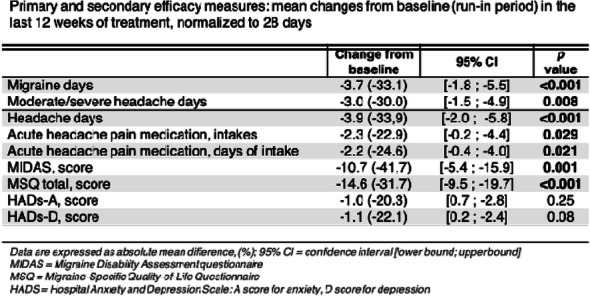
See text for description

## P0331 Patient-reported outcomes among migraine patients treated with cgrp-monoclonal antibodies in clinical practice

### L. Sánchez-Casado, C. Corral-Quereda, J. Díaz de Terán Velasco, M. Sastre-Real

#### Hospital Universitario La Paz, Neurology, Madrid, Spain

##### **Correspondence:** L. Sánchez-Casado

Objective: Monoclonal antibodies targeting calcitonin gene-related peptide (CGRP) or its receptor (anti-CGRP mAbs) have been shown to improve disability among migraineur patients. Nevertheless, studies assessing patient-reported outcomes (PROs) in the more complex population of a real‐world clinical setting are scarce. Our aim was to evaluate changes on headache-related disability in a series of patients with migraine treated with anti-CGRP mAbs.

Methods: This was a single-center prospective cohort study that includes patients with chronic migraine (CM) or high frequency episodic migraine (HFEM) and multiple preventive treatment failures who received anti-CGRP mAbs (erenumab, galcanezumab or fremanezumab). Migraine Disability Assessment Test (MIDAS) and Headache Impact Test-6 (HIT-6) scores were collected at baseline, 3 months and 6 months.

Results: 134 patients were prescribed anti-CGRP mAbs from January 2020 to January 2021. 107 (79.9%) had CM and 27 (20.1%) HFEM. Mean age was 49.5±10.5 years (mean±SD), 88% were female. 55.6% had failed to ≥6 prior preventive treatments. MIDAS score was significantly reduced from 55.0±72 at baseline to 22.0±65 at 3 months and to 22.5±50 at 6 months (median±IQR, p<0.0001). HIT-6 score reduction was also significant, from 67.5±6 to 60.0±10 at 3 months and to 65.5±12 at 6 months (median±IQR, p<0.0001).

Conclusion**:** Our results show that CGRP-mAbs improve headache-related disability in patients with CM and HFEM.

## P0332 BoNT-A efficacy in high frequency migraine: an exploratory study applying machine learning to predict therapy responsiveness

### D. Martinelli^1^, G. Castellazzi^2^, R. De Icco^1^, S. Arceri^3^, M. Allena^2^, A. Putortì^1^, L. Ahmad^1^, G. Cosentino^1^, G. Sances^2^, C. Tassorelli^1^

#### ^1^IRCCS Mondino Foundation, Headache Science and Rehabilitation Center / Dept of Brain and Behavioural Sciences at Pavia Univeristy, Pavia, Italy; ^2^IRCCS Mondino Foundation, Headache Science and Rehabilitation Center, Pavia, Italy; ^3^IRCCS Fondazione Mondino, Pavia University, Dept. of brain and behavioral science, Pavia, Italy

##### **Correspondence:** D. Martinelli

Background: Recently we reported OnabotulinumtoxinA (BoNT-A) efficacy in the prevention of high-frequency episodic migraine (HFEM) in an exploratory, open label, single-arm trial (Martinelli et al., submitted).

Objective: In this sub-analysis we sought to identify predicting elements of responsiveness to BoNT-A in HFEM.Methods: We enrolled 32 subjects with HFEM (8-14 migraine days/month) and thoroughly profiled them from a clinical/anamnestic perspective. After a 28-day baseline period, subjects underwent 4 BoNT-A treatments according to the PREEMPT paradigm, every 12 weeks. Subjects filled in a headache diary the number of monthly migraine days (MMD) was used to divide them into 2 groups according to the response to BoNT-A treatment in the last 12-weeks compared to baseline: responders (>50% migraine days reduction vs baseline). Collected data were used as input features to run a machine learning Random Forest (RF) algorithm.

Results: RF discriminated responders from non responders with a high classification accuracy of 85.71% (AUC=90.91%) using 4 baseline features: migraine onset age, opioid use, hospital anxiety and depression score (HADS) and Migraine Disability Assessment (MIDAS) score. High responsiveness positively correlates with migraine onset age and HADS-A score, and negatively correlates with ongoing opioid use and MIDAS score.

Conclusions: These findings identify a 4-feature panel of easy-to-obtain parameters predictive of BoNT-A therapy responsiveness in HFEM.

**Fig. 1 (abstract P0332).**
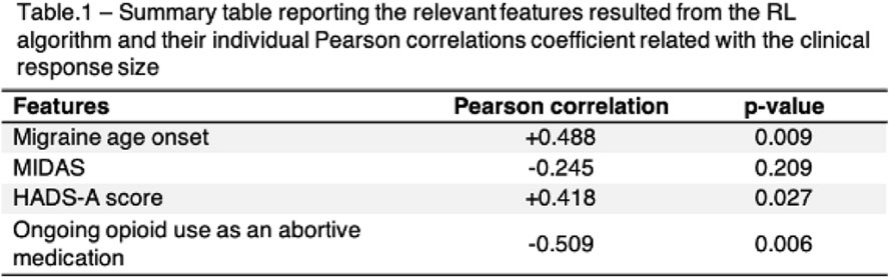
See text for description

**Fig. 2 (abstract P0332).**
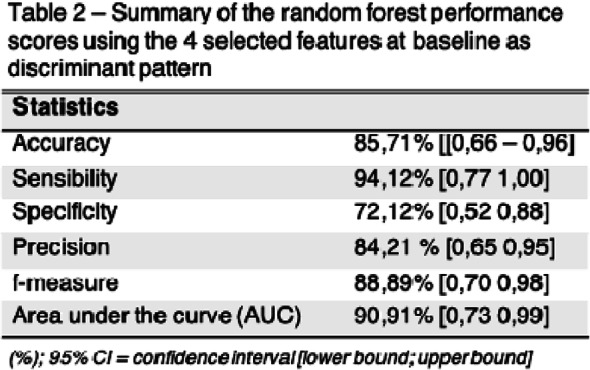
See text for description

## P0333 Has onabotulinumtoxinA follow-up delay during COVID-19 lockdown affected the migraine course?

### A. Gonzalez-Martinez^1,2^, Á. Planchuelo-Gómez^3^, Á. L. Guerrero Peral^4,5,6^, D. García-Azorín^4,5^, S. Santos-Lasaosa^7^, M. P. Navarro-Pérez^7^, P. Odriozola-González^8^, M. J. Irurtia^8^, S. Quintas^1,2^, R. de Luis-García^3^, A. B. Gago Veiga^1,2^

#### ^1^Universidad Autónoma de Madrid, Departamento de Neurología, Madrid, Spain; ^2^Hospital Universitario de La Princesa & Instituto de Investigación Sanitaria La Princesa, Headache Unit, Neurology, Madrid, Spain; ^3^Universidad de Valladolid, Imaging Processing Laboratory, Valladolid, Spain; ^4^Hospital Clínico Universitario de Valladolid, Headache Unit, Neurology Department, Valladolid, Spain; ^5^Institute for Biomedical Research (IBSAL), Salamanca, Spain; ^6^Universidad de Valladolid, Department of Medicine, Valladolid, Spain; ^7^Hospital Clínico Universitario Lozano Blesa & Aragon IIS, Headache Unit, Department of Neurology,, Zaragoza, Spain; ^8^Universidad de Valladolid, Department of Psychology, Valladolid, Spain

##### **Correspondence:** A. Gonzalez-Martinez

Objective: During the COVID-19 pandemic face-to-face procedures have been postponed. We aim to evaluate the impact of onabotulinumtoxinA follow-up delay in migraine during COVID-19 pandemic.

Methods: Subjective worsening, intensity of migraine attacks and frequency of headache and migraine were retrospectively compared between patients with unmodified and interrupted onabotulinumtoxinA follow-up in Headache Units.

Results: We included 67 patients with chronic migraine or high-frequency episodic migraine under onabotulinumtoxinA treatment, 65 (97.0%) female, 44.5±12.1 years old. Treatment administration was voluntarily delayed in 14 (20.9%) patients and nine (13.4%) were unable to continue follow-up. Patients with uninterrupted follow-up during lockdown presented 8.4 and 8.1 less monthly days with headache (adjusted p=0.011) and migraine attacks (adjusted p=0.009) compared to patients whose follow-up was interrupted, respectively.

Conclusion: Involuntary delay of onabotulinumtoxinA follow-up in patients with migraine due to COVID-19 pandemic was associated with a higher frequency of headache and migraine attacks. Safe administration of onabotulinumtoxinA during lockdown should be promoted.

## P0334 Safety and tolerability of anti-CGRP monoclonal antibodies: a real-world study

### C. Corral-Quereda, L. Sánchez-Casado, J. Diaz-de-Terán, M. Sastre-Real

#### Hospital Universitario La Paz, Neurología, Madrid, Spain

##### **Correspondence:** C. Corral-Quereda

Objective**:** Our aim was to assess the adverse events (AE) reported by patients with migraine treated with monoclonal antibodies against CGRP or its receptor (anti-CGRP mAbs) in daily clinical practice.

Methods: Data were prospectively collected after three months of treatment with galcanezumab, erenumab and fremanezumab at the Headache Clinic of a tertiary University Hospital, between January 2020 and January 2021. The variables included were: sex, age, presence and type of , concomitant preventive therapies for migraine and migraine characteristics.

Results: A total of 134 patients were included. AE were reported in 69 patients (51.5%). Constipation was the most frequent AE, reported by 45 patients (33.6%). Less frequent AE were dizziness (6 patients, 4.5%), myalgia (5 patients, 3.7%) and asthenia (5 patients, 3.7%). No severe AE occurred, however, constipation led to treatment discontinuation in one patient (<0.7%). We found no association between any AE (including constipation) and the anti-CGRP mAb used, epidemiological variables, concomitant treatment or migraine characteristics.

Conclusions: In patients with migraine anti-CGRP mAbs were well tolerated. Approximately half of patients suffered from AE in our series, but most of them were mild. The most frequent AE was constipation. There were no differences in AE between the different anti-CGRP mAbs.

## P0335 Psychometric Validation of a Novel Patient-reported Outcome Tool to Assess Impact of Migraine Across All Phases: the Migraine Symptom and Impact Questionnaire

### J. VanderPluym^1^, V. Ramirez Campos^2^, A. Cortez^2^, J. M. Cohen^2^, S. Gandhi^2^, S. Lewis^3^, C. Sweeney^3^, V. Williams^3^, J. Ma^3^, S. Yarr^3^, C. Romano^3^

#### ^1^Mayo Clinic, Phoenix, AZ, United States; ^2^Teva Branded Pharmaceutical Products R&D, Inc., West Chester, PA, United States; ^3^RTI Health Solutions, Research Triangle Park, NC, United States

#### **Correspondence:** J. VanderPluym

Objective: To evaluate psychometric properties of the Migraine Symptom and Impact Questionnaire (Mi-SAIQ), a novel, patient-reported outcome (PRO) tool designed to fully assess migraine impact across all 4 phases of a migraine attack and interictal phase between attacks.

Methods: Psychometric properties of Mi-SAIQ, including key elements of validity and reliability, were evaluated using data from a prospective, longitudinal, noninterventional study in adult migraine patients (n=126). Item-level analyses investigated behavior of Mi-SAIQ items and questionnaire structure to optimize scoring rules; subsequent composite-level analyses examined reliability, validity, and responsiveness of candidate Mi-SAIQ composite scores.

Results: Item-level frequency distributions supported the appropriateness of Mi-SAIQ response options. Confirmatory factor model fit indices were acceptable for 1- and 4-factor models, supporting the Mi-SAIQ composite scoring. For candidate composite scores, Cronbach's alphas (≥0.80) and test-retest intraclass correlation coefficients (≥0.75) were satisfactory. Correlations with validated PRO scores supported Mi-SAIQ composite score construct validity and known-group analyses supported their discriminating ability.

Conclusions: Overall findings support Mi-SAIQ as a useful tool for clinical practice/trial settings to describe the full migraine experience. Results suggest a short-form version could be useful, providing a potential avenue for future study.

## P0336 Which patient related outcome best reflects the willing of a patient to continue with an anti-CGRP monoclonal preventive treatment? A sub-analysis of a prospective study

### C. Nieves Castellanos, M. Losada López, M. I. Fabrich Marín, J. Pérez García, S. Díaz Insa

#### Hospital Universitari i Politécnic la Fe de Valencia, Valencia, Spain

##### **Correspondence:** C. Nieves Castellanos

Objetive: Refractory migraine is one of the most disabling pathologies. We present this study to analyze which indicator is the best correlation with the continuation of galcanezumab and erenumab.

Methods: We have carried out a real-life study collecting the patients with refractory migraine with treatment with galcanezumab or erenumab since January 2020. We present a sub-analysis of the data (migraine days, headache days) and scales (HIT-6, MIDAS, MSQ, pain catastrophizing scale (PCS)) and correlate them with the continuation of treatment at 3 months.

Results: Collected data from 220 patients, 81,55% women, 111 with erenumab and 109 with galcanezumab. 158 patients (71,82%) are satisfied with the treatment and continue after three months. The migraine frequency was reduced from 20,57 days to 16,78 (18,42%), use of triptans was reduced from 17,13 days per month to 10,26 (40,11%). The results of the scales are: EVA was reduced from 8,65 points of average to 6,85 points (20,81%), MIDAS was reduced from 93 points to 56,38 points (39,37%), HIT-6 was reduced from 68,86 points to 60,74 (24,71%), PCS changed from 32,74 to 22,28 (31,95%) and MSQ was increased from 29,2 of average to 47,51 (62,71%).

Conclusion: Quality of life measured with the MSQ scale correlates with the percentage of patients in a better way than migraine days. PROs which have in account the patient"s perception could be a good way to evaluate the willing of the patient to continue with a treatment.

## P0337 Study protocol of a randomised controlled trial of the efficacy of a smartphone-based therapy of migraine (SMARTGEM)

### A. S. Oliveira Gonçalves^1^, I. Laumeier^2^, B. Raffaelli^2^, M. Dahlem^3^, P. Burow^4^, F. Rimmele^5^, S. Nägel^4^, T. P. Jürgens^5^, T. Kurth^1^, U. Reuter^2^, L. Neeb^2^

#### ^1^Charité University Hospital Berlin, Berlin, Germany; ^2^Charité University Hospital Berlin, Berlin, Germany; ^3^Newsenselab GmbH, Berlin, Germany; ^4^Neurologie, Universitätsklinikum Halle, Halle, Germany; ^5^Kopfschmerzzentrum Nord-Ost, Universitätsmedizin Rostock, Rostock, Germany

##### **Correspondence:** A. S. Oliveira Gonçalves

Objective: Digitalisation offers new treatment approaches for people with migraine. Smartphone applications (apps) for migraine patients include a wide variety of functions, ranging from digital headache calendars to app-based treatments. Further possibilities arise by using electronic communication tools. However, there is currently insufficient evidence on the benefits of digital tools for patients. SMARTGEM aims to fill in this research gap.

Methods: SMARTGEM is a randomised controlled trial assessing whether the provision of a new digital form of care leads to a reduction in migraine frequency, improves quality of life, reduces medical costs and work absenteeism in people with migraine. It consists of M-sense (a medical app) and a communication platform with online consultations and a patient forum moderated by headache specialists (DRKSID: DRKS00016328).

Results: Adult patients with ≥ 5 migraine days/month at baseline were recruited from outpatient headache centres over 23 months and are being followed up for a year. Patients" baseline characteristics will be presented. First results are expected in 2022.

Conclusion: SMARTGEM constitutes a new integrated approach for migraine treatment. Its protocol offers an example of how to evaluate therapies using digital tools. Results will provide insightful information on the efficacy of electronic health tools in improving the quality of life of patients suffering from migraine while reducing healthcare resource consumption.

## P0338 Two Year Real World Prospective Quality of Life Data in Treatment Refractory Chronic Migraine Patients Treated with Erenumab in Ireland

### E. Troy^1^, A. A. Shrukalla^1^, A. Buture^2^, K. Conaty^1^, E. Macken^2^, R. Lonergan^2^, J. Melling^2^, N. Long^1^, E. Shaikh^1^, E. Tomkins^1^, M. Ruttledge^1^

#### ^1^Beaumont Hospital, Neurology, Dublin, Ireland; ^2^Mater Misericordiae University Hospital, Neurology, Dublin, Ireland

##### **Correspondence:** E. Troy

Background: CGRP monoclonal antibodies have been shown to be effective in patients with chronic migraine. Erenumab is a fully-human anti-CGRP monoclonal antibody which targets the CGRP receptor. Quality of Life (QOL) questionnaires are one of the only tools that we have to measure response to treatment in episodic and chronic migraine.

Objective: To prospectively determine the efficacy of Erenumab on QOL in chronic migraine patients who have been refractory to at least 4-5 prior preventative migraine therapies.

Methods: 148 consecutive chronic migraine patients were given either 70mg or 140mg Erenumab every 28 days by subcutaneous injection. Patients completed migraine specific QOL questionnaires before starting treatment with Erenumab, and at 3-6 months intervals, continuing for up to two years after starting treatment. The migraine specific QOL questionnaires were: the Headache Impact Test-6 (HIT-6), Migraine Associated Disability Assessment (MIDAS) test and Migraine-Specific Quality-of-Life Questionnaire (MSQ).

Results: 148 chronic patients started Erenumab between December 2018 and October 2019. Approximately 45% of these patients stopped treatment during the first year due to lack of efficacy and/or side effects. Up to 55% of patients had clinically significant improvement after treatment for 6-12 months, and wished to stay on treatment.

Conclusion: Clinically significant improvements in QOL were experienced by approximately 55% of treatment refractory chronic migraine patients treated with Erenumab over a 1-2 year period.

## P0339 Rimegepant Versus Atogepant and Monoclonal Antibody Treatments for the Prevention of Migraine: A Systematic Literature Review and Network Meta-analysis

### E. Popoff^1^, L. Powell^1^, T. Rahim^1^, K. Johnston^1^, L. Harris^2^, A. Thiry^2^, V. Coric^2^, G. L'Italien^2^

#### ^1^Broadstreet Health Economics & Outcomes Research, Vancouver, Canada; ^2^Biohaven Pharmaceuticals, New Haven, CT, United States

##### **Correspondence:** E. Popoff

Objective: The objective was to evaluate the relative efficacy of rimegepant, atogepant, and monoclonal antibodies (mAbs) for migraine prevention.

Methods: A comprehensive systematic literature review (SLR) was conducted. Efficacy was compared via network meta-analyses (NMAs). Evidence consisted of placebo-controlled trials of: rimegepant 75mg, atogepant 10mg, 30mg, 60mg, erenumab 70mg, 140mg, galcanezumab 120mg, 240mg, eptinezumab 30mg, 100mg, 300mg, and fremanezumab 225mg, 675mg. The rimegepant study included episodic migraine (77%) and chronic migraine (23%) patients; others had 100% episodic migraine patients. Efficacy outcomes included change from baseline in monthly migraine days (MMDs) and number achieving a 50% reduction in MMDs.

Results: Significantly favourable differences for change in MMDs and number achieving a 50% reduction in MMDs were seen with active treatments versus (vs.) placebo (Figure 1). Change in MMDs vs. placebo ranged from eptinezumab 100mg (mean difference -0.70 MMDs [95% credible interval (Crl) -1.26, -0.14]) to galcanezumab 240mg (-1.76 [-2.21, -1.31]); -0.90 (-1.47, -0.33) for rimegepant. For number achieving a 50% reduction in MMDs, estimates vs. placebo ranged from atogepant 60mg once daily (risk difference 11.13% [95% Crl 1.07%, 21.78%]) to galcanezumab 120mg (23.40% [17.60%, 29.06%]); 12.90% (5.37%, 20.53%) for rimegepant.

Conclusions: Rimegepant, atogepant, and mAbs showed similar efficacy in migraine prevention when compared to placebo.

**Fig. 1 (abstract P0339).**
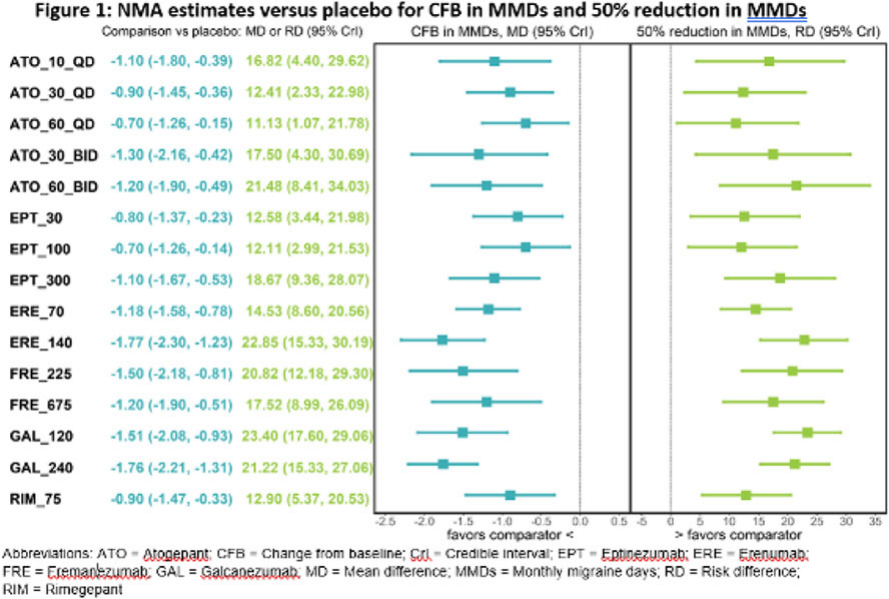
See text for description

## P0340 Economic modeling of migraine prevention therapies: Considerations for current and emerging therapies

### K. Johnston^1^, L. Harris^2^, L. Powell^1^, E. Popoff^1^, V. Coric^2^, G. L'Italien^2^

#### ^1^Broadstreet Health Economics & Outcomes Research, Vancouver, Canada; ^2^Biohaven Pharmaceuticals, New Haven, CT, United States

##### **Correspondence:** K. Johnston

Objective: The objective was to review guidelines for modeling migraine prevention therapies and features of novel therapies to identify further recommendations.

Methods: A targeted literature review was undertaken for recent/emerging migraine prevention therapies and economic modeling considerations, focusing on therapy features anticipated to impact modeling.

Results: Monoclonal antibodies (mAbs) and gepants were identified as migraine prevention therapies of interest. Rimegepant bears consideration for modeling because of its efficacy as both an acute and preventive therapy. Migraine prevention models focusing on monthly migraine days (MMDs) should encompass interactions between acute and preventive therapies, and potential variation in acute therapy efficacy. The mAbs are administered via periodic injections and may have health-related quality of life (HRQoL) implications beyond those associated with MMDs and/or toxicity, such as patient treatment preferences, waning of effectiveness throughout an injection cycle, and cyclical patterns of migraine. Thus, economic evaluations of rimegepant should encompass both acute and preventive treatment with respect to MMDs, while economic evaluations of mAbs should include comprehensive HRQoL impacts.

Conclusion: Migraine prevention models should account for features of gepants and mAbs, including interactions between acute and preventive therapy and implications beyond MMD reduction.

## P0341 Clinical characteristics of patients who preferred migraine prevention treatment with CGRP monoclonal antibodies

### N. Vashchenko^1,2^, K. Skorobogatykh^2^, J. Azimova^2^, D. Korobkova^2^

#### ^1^Sechenov University, Neurological department, Moscow, Russian Federation; ^2^University Headache Clinic, Moscow, Russian Federation

##### **Correspondence:** N. Vashchenko

Background and objectives: CGRP monoclonal antibodies (mAbs) were approved for migraine prevention in Russia in August 2020. They are not covered by insurance but available for purchase in pharmacies. We want to present the characteristics of patients who were prescribed CGRP mAbs in our headache clinic.

Methods: We evaluated all patients, with whom CGRP mAbs were used as the medication of choice. To assess migraine's severity, we used Migraine Disability Assessment Test (MIDAS) and Work Productivity and Activity Impairment Questionnaire (WPAI). We also used Hospital Anxiety and Depression Scale (HADS).

Results: From October 2020 till March 2021, 70 patients started monthly injections of CGRP mAbs. 65 women, 10 men, mean age 37,2±6,3. From them,27 patients had episodic migraine,43–chronic. Prior preventive therapy experience ranged from 0 in 13 patients, to 3 or more medication groups.13 patients had clinically significant anxiety (mean 5,8±4 points),9 – depression (mean 3,7±3,5). The mean MIDAS score was 47,6. Out of 44 patients working for pay, 14 reported missing their work due to their migraine attack. 38 people (86%) said that migraine interferes with their work productivity intensity with 5 or more out of 10.

Conclusions: Patients who prefer CGRP mAbs therapy usually have severe disabling migraine, sometimes resistant or refractory. However, patients with episodic migraine with no previous preventive treatment also choose monthly injections instead of daily intake of pills.

## P0342 Crowdsourcing post-marketing safety surveillance for migraine preventives: Self-reported adverse events associated with calcitonin gene-related peptide (CGRP) therapeutics on a social media forum

### P. Zhang^1^, B. Kamitaki^1^, T. Do^2^

#### ^1^Rutgers Robert Wood Johnson Medical School, New Brunswick, NJ, United States; ^2^Rigshospitalet Glostrup, Neurology, Copenhagen, Denmark

##### **Correspondence:** P. Zhang

Objectives: Real-world observational data, such as those contained within social media platforms, can summarize diverse patient experiences and detect drug-related safety signals. We characterized adverse events related to calcitonin gene-related peptide therapeutics on Reddit, an anonymous online discussion forum.

Methods: We examined differences in word frequencies from posts extracted from Reddit subforum r/Migraine from 2010 to 2020 using computational linguistics. In the validation phase, we compared propranolol versus topiramate, as well as propranolol and topiramate each against randomly selected posts. In the application phase, we examined posts discussing the CGRP therapeutics erenumab and fremanezumab to determine frequently discussed side effects.

Results: From 22,467 Reddit r/Migraine posts, we extracted 402 propranolol posts, 1423 topiramate posts, 468 erenumab posts, and 73 fremanezumab posts. Comparing topiramate against propranolol identified a number of expected side effects. Erenumab compared against a random selection of terms identified "constipation" as a recurring key word. Erenumab against fremanezumab identified "constipation," "depression," "vomiting," and "muscle." No adverse events were identified for fremanezumab.

Conclusions: Computational linguistics applied to social media can identify potential adverse events of interest for migraine preventives. Further studies are needed to explore side effects and safety of the novel CGRP medications.

## P0343 The real-life early and continuative response to Galcanezumab in chronic migraine: 3-month analysis of the multicenter prospective cohort GARLIT study

### C. Altamura^1^, N. Brunelli^1^, C. M. Costa^1^, C. Aurilia^2^, G. Egeo^2^, L. Fofi^2^, V. Favoni^3^, G. Pierangeli^3^, R. Messina^4^, M. Filippi^4^, B. Colombo^4^, C. Lovati^5^, M. Aguggia^6^, D. Bertuzzo^6^, F. D'Onofrio^7^, A. Doretti^8^, P. Di Fiore^9^, F. Frediani^9^, C. Finocchi^10^, R. Rao^11^, F. Schiano Di Cola^11^, F. Bono^12^, A. Ranieri^13^, M. Albanese^14^, S. Cevoli^3^, P. Barbanti^2^, F. Vernieri^1^

#### ^1^Campus Bio-Medico University of Rome, Neurology, Rome, Italy; ^2^Headache and Pain Unit, IRCCS San Raffaele Pisana, Rome, Italy; ^3^IRCCS Istituto delle Scienze Neurologiche di Bologna, Bologna, Italy; ^4^IRCCS San Raffaele Scientific Institute, Vita-Salute San Raffaele University, Neurology Unit, Milan, Italy; ^5^University Hospital L. Sacco, Neurology Unit, Milan, Italy; ^6^Asti Hospital, Neurology and Stroke Unit, Asti, Italy; ^7^Giuseppe Moscati Hospital, Neurology Unit, Avellino, Italy; ^8^Istituto Auxologico Italiano, IRCCS, Neurology, Stroke Unit and Laboratory of Neuroscience, Milan, Italy; ^9^S. Carlo Borromeo Hospital, ASST Santi Paolo e Carlo Milan,, Headache Center, Neurology and Stroke Unit, Milan, Italy; ^10^IRCCS Ospedale Policlinico San Martino, Genova, Italy; ^11^University of Brescia, Neurology Unit, Department of Clinical and Experimental Sciences, Brescia, Italy; ^12^A.O.U. Mater Domini, Neurology Unit, Catanzaro, Italy; ^13^“A. Cardarelli” Hospital, Headache Centre, Neurology and Stroke Unit, Naples, Italy; ^14^University Hospital of Rome “Tor Vergata”, Department of Systems Medicine, Rome, Italy

##### **Correspondence:** C. Altamura

Objective**:** This prospective, observational, multicenter study investigated the early and sustained effectiveness of therapy with galcanezumab in real-life patients with chronic migraine (CM).

Methods: All consecutive adult patients with CM having clinical indication (3 failed preventives) for Galcanezumab were considered. We collected during one run-in month period (baseline) and during the first three months of therapy: headache days of at least moderate intensity (MHDs), monthly painkillers intake, migraine features and clinical profiles.

Results: 156 patients (82.4% female, 47.3±12.3 years) were enrolled. Patients with a 3-month ≥50% responder rate were 65 (41.7%) and presented a lower body mass index (BMI; p=.004), more frequently unilateral migraine pain (p=.002, OR 5.365 95%CI [1.824 -15.776]), and good response to triptans (p=.003, OR 2.932 95%CI [1.466 -5.863]). Sustained conversion from CM to EM (55.8% of cases) was more frequently observed in those with good response to triptans (p=.003; OR 2.824 95%CI [1.379-5.782]) and unilateral pain (p=.046; OR 2.727 95%CI [1.068-6.964]). Continuative MO discontinuation (61.8% of subjects with baseline MO) was more frequently observed in patients with good response to triptans (p=.002, OR 3.824 95%CI [1.587-9.210]).

Conclusions: Unilateral pain, good response to triptans and normal weight may be associated with a positive sustained response in the first three months of therapy with Galcanezumab in chronic migraine.

## P0344 The efficacy of re-using medicines for preventive treatment of migraine

### D. Sotnikov, O. Potapov

#### Sumy State University, Neurosurgery and Neurology, Sumy, Ukraine

##### **Correspondence:** D. Sotnikov

Objective**:** to determine the duration of the therapeutic effect of preventive treatment in therapeutic groups and the effectiveness of their reuse.

Material and methods**:** The study included 76 persons who had a 50% or more reduction in migraine attacks after three-month treatment with a combination of amitriptyline (average daily dose of amitriptyline was 37.0±2.4 mg and propranolol 80.0±4.7 mg) – 29, lamotrigine (111.1±7.2 mg) – 23, gabapentin (864.0±46.9 mg) – 24 patients. The duration of the therapeutic effect was estimated after 6 months.

Results:

**Table:** 

Therapeutic groups	The return of attacks is less than 50%	The return of attacks by 51–80%	The return of attacks by 81–100%
Amitriptyline + propranolol	10 (34,5%)	12 (41,4%)	7 (24,1%)
Lamotrigine	7 (30,4%)	9 (39,2%)	7 (30,4%)
Gabapentin	5 (20,8%)	10 (41,7%)	9 (37,5%)

Thus, 65.5% patients using of propranolol with amitriptyline, 69,6% patients using of lamotrigine, 79.2% patients using of gabapentin had returning 50-100% of the previous number attacks and required repeated courses. Reduction in the frequency of attacks by half or more in re-prophylaxis using of propranolol with amitriptyline was in 78.9%. Re-using of lamotrigine was effective in 62.5%, gabapentin – in 52.6%.

Conclusions: It"s necessary to study the optimal duration of preventive treatment and the choice of reuse of the drug should be individually given the probable reduction in their effectiveness.

**Fig. 1 (abstract P0344).**
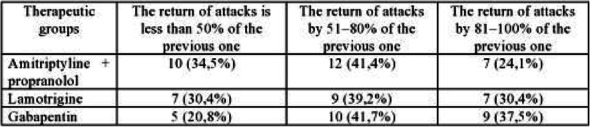
See text for description

## P0345 Over 65 years old erenumab experience

### J. S. Rodriguez-Vico, A. Jaimes-Sanchez, A. García-Gómez

#### HospitalUniversitario Fundación Jiménez Díaz, Headache Unit, Madrid, Spain

##### **Correspondence:** J. S. Rodriguez-Vico

Objetive: People over 65 years old were excluded in randomized trials with Erenumab. We describe the efficacy of Erenumab with real-world evidence in people with or over 65 years-old.

Methods: Retrospective study in the Headache Unit of a University Hospital. Charts of patients receiving Erenumab (70 /140mg) from December 2019 to March 2021 were reviewed. Demographic variables were collected and efficacy analysis included change from baseline in monthly headache days (MHD), and responses greater than 50% and 75% at 3 months.

Results: We reviewed the charts of 240 patients. Twenty-one subjects older than 64 years and who had received at least three injections of Erenumab were included. The mean age was 69,62 (range 65-86). Nineteen patients (90.5%) had Chronic Migraine, and 2 (9,5%) had high frequency episodic migraine. As usual in Migraine, most of them were women 16 (76%). Mean baseline MHD was 19,4 (SD 7.4). After 3 months with Erenumab, mean MHD was 14,2 (SD 9.8). Mean improvement was -5,2 days per month (SD 6.1) with a 31,4% reduction of MHD. Seven patients (33%) had an improvement greater than 50% and 2 (9%) greater than 75%. No significant differences in terms of efficacy were found between the 70 and 140 milligram doses.

Conclusion: Erenumab is effective in people older than 65 years old. We are looking to collect data at 6 months where we would probably find more 50% responses. More studies need to be done to confirm our suspect.

## P0346 Two years of guideline-oriented migraine therapy with Erenumab under the regulatory conditions of the healthcare system in Germany

### A. Peikert^1^, E. Stühler^2^, V. Tozzi^2^, M. Köchling^1^, H. Israel-Willner^1^, H. Dikow^1^, F. Rossnagel^1^, S. Braune^1^, A. Bergmann^1^

#### ^1^NeuroTransData (NTD) network, Neuburg, Germany; ^2^PricewaterhouseCoopers AG, Zurich, Switzerland

##### **Correspondence:** A. Peikert

Background/Objective: German regulatory guidelines demanded 5 (EM) or 6 (CM) failed/contraindicated first-line prophylactics before covering the costs of CGRP-mABs, Valproate meanwhile was omitted. Medical guidelines recommend a significant (50%) response as a prerequisite for sustained prescription. We aimed to describe results and implications of E. treatment in neurological practices under these conditions.

Methods: The headache registry of NeuroTransData network of neurologists captures demographics, headache characteristics, comorbidities, symptom load and the use and effect of acute and preventive medication via standardized webbased data entry and smartphone app.

Results: Currently (01.01.21) 5121 pts. fulfilled the ICHD-3-criteria for migraine. 435 (8,5 %) received E., 431 could be evaluated. 140 (32,5 %) had CM. 14 pts. (3,2 %) stopped therapy due to side effects, 76 (17,6 %) to lacking efficacy, 43 (9,9 %) to other reasons. The responder-rate (at least 50% reduction of migraine days) rose from 40 % after 3 up to 65% after 24 injections, 25 % of the patients had a less than 25 % response after 2 years.

Conclusions: Treatment with Erenumab under the regulatory conditions in Germany was mostly well tolerated and effective. A considerable proportion of patients was treated for up to 2 years without reaching 50 % response. This indicates a good ratio between tolerability and effectiveness in the evaluated sample of therapy resistant migraine patients.

## P0347 Long-term (>48 weeks) safety and tolerability of erenumab in real-life

### C. Aurilia^1^, S. Cevoli^2^, G. Egeo^1^, L. Fofi^1^, R. Messina^3^, A. Salerno^4^, P. Torelli^5^, M. Albanese^6^, A. Carnevale^7^, F. Bono^8^, D. D'Amico^9^, M. Filippi^3^, C. Altamura^10^, F. Vernieri^10^, B. Colombo^3^, F. Frediani^11^, B. Mercuri^4^, F. D'Onofrio^12^, L. Grazzi^9^, M. Aguggia^13^, V. Favoni^2^, C. Finocchi^14^, P. Di Fiore^11^, C. M. Costa^10^, N. Brunelli^10^, A. Fallacara^10^, D. Bertuzzo^13^, M. Zucco^15^, L. Di Clemente^15^, M. Trimboli^16^, A. Pascarella^8^, L. Manzo^8^, P. Barbanti^1^

#### ^1^IRCCS San Raffaele, Headache and Pain Unit, Rome, Italy; ^2^IRCCS Istituto delle Scienze Neurologiche, Bologna, Italy; ^3^IRCCS San Raffaele Scientific Institute, Vita-Salute San Raffaele University, Neurology Unit, Milan, Italy; ^4^San Giovanni Addolorata Hospital, Rome, Italy; ^5^University of Parma, Department of Medicine and Surgery, Headache Center, Unit of Neurology, Parma, Italy; ^6^University Hospital of Rome "Tor Vergata",, Neurophysiopathology, Rome, Italy; ^7^San Filippo Neri Hospital, Rome, Italy; ^8^A.O.U. Mater Domini, Center for Headache and Intracranial Pressure Disorders, Neurology Unit, Catanzaro, Italy; ^9^Neurological Institute C. Besta IRCCS Foundation, Headache and Neuroalgology Unit, Milan, Italy; ^10^Headache and Neurosonology Unit, Policlinico Universitario Campus Bio- Medico, Rome, Italy; ^11^Headache Center, ASST Santi Paolo Carlo, Milan, Italy; ^12^AOSG Moscati, Avellino, Italy; ^13^Cardinal Massaia Hospital, Asti, Italy; ^14^San Martino Hospital, Genova, Italy; ^15^San Camillo Hospital, Rome, Italy; ^16^San Carlo Hospital, Potenza, Italy

##### **Correspondence:** C. Aurilia

Background**:** Erenumab proved to be safe and well tolerated in a 5-year continuation of a 1-year double-blind, placebo-controlled study.

Aim**:** to assess >48-week erenumab tolerability and safety in a real-world setting

Methods**:** In this long term (>48-week), multicenter (n=15), longitudinal cohort real life study, we monitored all the adverse events emerged in consecutive adult patients with high-frequency episodic migraine (HFEM) or chronic migraine (CM) treated with monthly erenumab 70 mg or 140 mg from 20 December 2018 to 15 December 2020.

Results**:** 442 patients (HFEM: 115; CM: 327) were treated with erenumab for >48 weeks: 209 (47.3%) patients were treated for 49-60 weeks, 132 (29.9%) for 61-72 weeks; 73 (16.5%) for 73-84 weeks; 21 (4.7%) for 85-100 weeks. Overall, >1 treatment emergent adverse event (TEAE) was reported by 136 (30.8%) [HFEM: 43 (37.4%); CM: 93 (28.4%)]. Most common TEAE were constipation (n =66; 14.9 %), injection site erythema (n =15; 3.4%), and influenza (n =7; 1.6%). Serious adverse events (SAE) were reported by 8 patients (1.8%) and led to treatment discontinuation: severe constipation (n=3), abdominal pain (n=1), NSTEMI (n=3), Covid-19 infection (n=1). Only severe constipation was considered treatment-related SAE (0.45%).

Conclusion**:** Erenumab is safe and well tolerated also in long-term treatment (>48 weeks) in real life.

## P0348 Initial Efficacy Evidence of Migraine Preventive Treatment Using External Combined Occipital and Trigeminal Nerve Stimulation

### R. Sharon^1,2^, S. J. Tepper^3,4^

#### ^1^Sheba Medical Center, Ramat-Gan, Israel; ^2^Tel-Aviv University, Tel-Aviv, Israel; ^3^Geisel School of Medicine at Dartmouth, Hanover, NH, United States; ^4^Dartmouth Headache Center, Lebanon, NH, United States

##### **Correspondence:** R. Sharon

Objective: Combined occipital and trigeminal nerve stimulation (COT-NS) has shown marked results in the abortive treatment of migraines. Until recently, COT-NS was only available with implanted systems. A new noninvasive COT-NS system (Relivion®) was recently approved by the FDA for acute migraine, following successful results of a pivotal clinical trial. The current retrospective study was designed to collect real-world data regarding the safety and efficacy of the COT-NS system in the preventive treatment of migraines.

Methods: Seventeen patients with high-frequency episodic migraine or chronic migraine self-administered daily 20-minute treatments with the COT-NS system and electronically reported migraine characteristics for a duration of 3-6 months. The primary efficacy measure was change (%) in monthly migraine days in the final treatment month compared to baseline. Responder-rate (patients with ≥50% reduction in monthly migraine days) was additionally measured. A physician remotely monitored treatment progress. Reports of adverse events were collected as well.

Results: Average migraine days frequency decreased by 63%, from 14.6 days per month at baseline to 5.4 days at the final treatment month (p<0.0001). Patient responder rate was 76%. No serious adverse events were reported.

Conclusions: These initial results indicate for the first time that COT-NS may serve as a highly effective preventive treatment to reduce headache frequency in episodic and chronic migraine patients.

## P0349 Erenumab in patients failing Onabotulinum toxin A for the treatment of refractory chronic migraine

### Â. Abreu, R. Pinheiro, E. Parreira

#### Hospital Prof. Dr. Fernando Fonseca, Amadora, Portugal

##### **Correspondence:** Â. Abreu

Objectives: Migraine is a disabling disease but with the development of calcitonin gene-related peptide receptor antagonists there is a new therapeutic option for migraine prophylaxis.

We intended to evaluate the therapeutic response with Erenumab in patients who have previously failed treatments with Onabotulinum toxin A (BoNT-A).

Methodology: Prospective analysis of patients with refractory migraine who failed previous treatment with BoNT-A (January 2016 to January 2021) and that started Erenumab. Demographic data, frequency and intensity of crises and side effects were analyzed.

Results: Twelve patients (11 women and 1 man) with an average age of 45.7 years (23-70) were included. Six patients were diagnosed with frequent episodic migraine and the remaining chronic migraine.

Most patients discontinued BoNT-A treatment because of therapeutic inefficiency (n=10) or adverse reaction (n=1).

Overall, there was an improvement in both the intensity and frequency of headache with an average reduction of 4.8 days per month with headache and an average reduction of 4.2 days per month with moderate or severe headache.

Four patients with chronic migraine interrupted treatments due to lack of effectiveness (n=3) or pregnancy plans (n=1).

Conclusion: There was an improvement in patients treated with Erenumab previously submitted to treatments with BoNT-A with an excellent safety profile.

For patients refractory to Erenumab and BoNT-A, alternatives will be needed.

## P0350 Satisfaction with galcanezumab as a medication: a cross sectional study in migraine patients

### A. López-Bravo^1^, A. Oliveros-Cid^2^, L. Sevillano-Orte^2^

#### ^1^Aragon Institute for Health Research (IIS Aragón), Zaragoza, Spain; ^2^Hospital Reina Sofía, Neurology, Zaragoza, Spain

##### **Correspondence:** A. López-Bravo

Background and objective: Treatment satisfaction is of utmost importance for ensuring adherence. Galcanezumab is a new therapy for the treatment of migraine. The objective was to evaluate treatment satisfaction with galcanezumab and to identify factors that may influence patients" satisfaction.

Methods: Patient perspectives on satisfaction were evaluated with the Spanish versin of the Treatment Satisfaction Questionnaire for Medication version 1.4(TSQM 1.4). The TSQM 1.4 domain scores range from 0 to 100.

Results: Study participants consisted of thirty migraine patients, of which 76.67% had chronic migraine and 80% were women. TSQM scores at 12 weeks were as follows: graded effectiveness 80.6%, graded side effects 100%, graded convenience 83.3% and global satisfaction (GS) 78.6%. At 24 weeks: effectiveness 66.7%, side effects 100%, convenience 83.3% and GS 85.7%. Compared to previous preventive treatment, higher median scores were observed in the dimensions"side effects", "convenience" and "effectiveness"(93.5 ± 14.8 Vs 73.1 ± 21.0 and 64.8 ± 20.6, respectively). Regression analysis showed that the change in monthly migraine days(MMDs), Headache Impact Test scores(HIT) and Migraine Disability Assessment(MIDAS) scores were significantly associated with global satisfaction at 12 weeks.

Conclusion: The results of this study revealed that the majority of migraine patients were highly satisfied with galcanezumab in terms of efficacy, safety, convenience, and global satisfaction.

## P0351 Efficacy and sustainability of greater occipital nerve blocks in vestibular migraine prevention: a retrospective analysis

### E. Rykova^1^, L. Murdin^2^, S. Y. Ahmad^1^, E. Filips^1^, G. Lambru^3^

#### ^1^King's College London, London, United Kingdom; ^2^Guy's and St Thomas' Hospital NHS Foundation Trust, Audio-vestibular Medicine, ENT, London, United Kingdom; ^3^Guy's and St Thomas' Hospital NHS Foundation Trust, Pain and Neurology, London, United Kingdom

##### **Correspondence:** E. Rykova

Background and Objective**:** Only sparse data on treatments for vestibular migraine (VM) are published. Greater occipital nerve blocks (GONBs) are widely used for the prevention of primary and secondary headache disorders. However no data evaluating the effectiveness in VM is available. We present a retrospective analysis on the effect of GONBs in adults with VM coming from a single specialist Headache clinic.

Methods**:** Consecutive patients with VM diagnosed between 2019-2020 who underwent at least one GONB were included. Responders were defined as patients that obtained at least 50% reduction in headache days.

Results**:** Twenty adults were identified. After the first GONB, 15 patients (75%) were considered responders: six obtained a 100% improvement in headache symptoms and nine obtained at least a 50% headache improvement. Four patients did not respond. One patient experienced a transient worsening. The mean duration of improvement in responders was 68 days (SD ±78.6, range 21-304 days). 13/15 responders to the first GONB had a second treatment three months later. Nine of them (69%) continued to respond. Of these, seven patients (78%) reported a sustained response to the third GONB. There were no adverse events.

Conclusion**:** GONBs are a safe, possibly effective with sustained benefit overt time for the headache symptoms of VM. Larger studies with an accurate evaluation of the effect of GONBs on the vestibular component of VM would clarify the role of this treatment for VM.

## P0352 The efficacy of repetitive tran cranial magnetic stimulation in treating patients with chronic daily headache

### A. Hanafy, I. Fahmy, A. Farouk, A. Labib

#### Kasr Alaini, Clinical neurophysilogy, Cairo, Egypt

##### **Correspondence:** A. Hanafy

Abstract Background: Headache is the most common pain disorder, affecting around 66% of the global population. This study aimed to investigate the efficacy of high-frequency repetitive transcranial magnetic stimulation (rTMS) in treating patients with primary chronic daily headaches (chronic tension-type headache and chronic migraine). Methods: Twenty-seven patients participated in the study, divided into 2 groups: a study group (16 patients) and a control group (11 patients). Treatment consisted of 12 high-frequency (5 Hz) real rTMS sessions, delivered over the left dorsolateral prefrontal cortex (DLPFC), whereas sham rTMS was used for the control group.

Results: Patients of the study group, after real rTMS stimulation, showed a high statistically significant reduction of the measured headache parameters compared to the control group (P value < 0.001), and the percentage of improvement was 94.5%. No significant reduction of headache parameters, after sham rTMS stimulation, was observed in the control group (P value > 0.05) and the percentage of improvement was 7.9%.

Conclusion: High-frequency rTMS is effective in reducing chronic tension headaches and chronic migraines. This finding runs with the approval of the suggested role of DLPFC in pain control. This might open opinions for new treatment strategies in tension-type headache and migraine prevention.

Keywords: Repetitive trans-cranial magnetic stimulation, Chronic daily headache, Tension headache, Migraine

## P0353 Noninvasive Vagus Nerve Stimulation for Prevention of Episodic Migraine: a Proof-of-Concept Study

### J. Ailani^1^, N. Hindiyeh^2^, K. Knievel^3^, T. Smith^4^, S. Nadkarni^5^, J. Wolfe^6^, S. J. Nahas^7^, E. L. H. Spierings^8^

#### ^1^Georgetown University Hospital, Department of Neurology, Washington, DC, United States; ^2^Stanford University, Neurology & Neurological Sciences, Palo Alto, CA, United States; ^3^Barrow Neurological Institute, Phoenix, AZ, United States; ^4^StudyMetrix Research, St. Peters, MO, United States; ^5^Downtown L.A. Research Center, Los Angeles, CA, United States; ^6^Allergy & Asthma Associates of Santa Clara Valley Research Center, San Jose, CA, United States; ^7^Thomas Jefferson University, Jefferson Headache Center, Philadelphia, PA, Germany; ^8^Boston Headache Institute, Boston PainCare, Waltham, MA, United States

##### **Correspondence:** J. Ailani

Objective**:** To evaluate the efficacy and safety of noninvasive auricular vagus nerve stimulation (aVNS) in the prevention of episodic migraine.

Methods**:** This study enrolled participants 18-65 years of age, with a >1-year history of migraine and 4-14 migraine headache days/month. Following a 1-month baseline period, eligible participants received purpose-designed earbuds that transcutaneously deliver electrical stimulation to the auricular vagus nerve. Treatment was administered daily for 6 months. The primary outcome was the overall mean change from baseline in the number migraine days/month. Secondary measures included the proportion of subjects with ≥50%, ≥75%, and 100% reduction in migraine days/month and the incidence of treatment-emergent adverse events.

Results**:** Forty-five subjects were enrolled; 35 were included in the modified ITT population. The mean (SD) number of migraine days/month decreased from 8.3 (2.6) at baseline by an average of -4.04 (3.42) (p<0.05) at Months 4 to 6. At Months 4 to 6, 45%, 27%, and 17% of subjects had a ≥50%, ≥75%, or 100% decrease, respectively, in the number of migraine days/month. Sixteen device-related events were reported; all were mild or moderate and were resolved without intervention or further sequalae.

Conclusions**:** This mode of aVNS, used for 6 months, provided clinical benefits to participants with episodic migraine with minimal side effects. Further evaluation in larger, well-controlled studies is needed.

## P0354 Effect of transcranial direct current stimulation compared to sham in episodic migraine: systematic review and meta-analysis of randomized controlled trials

### F. Haghdoost^1^, D. Padala^2^, A. Salam^3^, C. Delcourt^1^, A. Rodgers^1^

#### ^1^University of New South Wales, The George Institute for Global Health, Sydney, Australia; ^2^The George Institute for Global Health, Hyderabad, India; ^3^University of New South Wales, The George Institute for Global Health, Hyderabad, India

##### **Correspondence:** F. Haghdoost

Objective: To do a meta-analysis to evaluate the effect of transcranial direct current stimulation (tDCS) on episodic migraine.

Methods: MEDLINE and EMBASE were searched until October 2020. Randomized clinical trials that compared the effect of tDCS to sham in adults with episodic migraine and reported migraine frequency per month were included. Two researchers, independently in duplicate, screened the studies, and collected data from included studies. Meta-analysis for mean difference in headache frequency per month was conducted using random effects model by Comprehensive Meta-Analysis (CMA) software.

Results: Overall, five studies (185 participants) were included. Studies were grouped based on the lead placement. Figure 1 illustrates the results. Headache frequency per month reduced significantly in studies with active anodal stimulation (excitatory) over occipital cortex [mean difference=1.8 (95% CI: 0.4, 3.1)] and motor cortex [mean difference=1 (95% CI: 0.4, 1.6)] but not in studies with cathodal stimulation (inhibitory) over occipital area [mean difference=0.2 (95% CI: -0.5, 0.8)] compared to sham. Among trials with benefits, there was evidence that these benefits continued after the tDCS treatment period.

Conclusion: This meta-analysis indicates that at least for two montages in tDCS there is encouraging evidence of benefit in episodic migraine patients compare to sham. However, there is ongoing uncertainty on the size of benefits, optimal placement and duration of therapy.

**Fig. 1 (abstract P0354).**
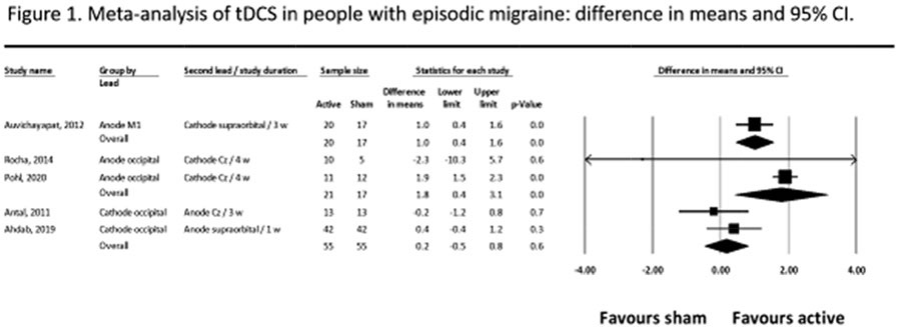
See text for description

## P0355 Effectiveness of tDCS in patients with chronic migraine: a randomized controlled trial

### M. D. Rahimi^1^, J. Salehi Fadardi^2^, E. Azizi^3^, F. Alidoosti^4^, N. Karimi^2^

#### ^1^Ferdowsi University of Mashhad, Faculty of Education and Psychology, Herat, Afghanistan; ^2^Ferdowsi University of Mashhad, Mashhad, Iran; ^3^Shahid Beheshti University, Tehran, Iran; ^4^Khayyam University, Mashhad, Iran

##### **Correspondence:** M. D. Rahimi

Background: treating migraine, using a non-invasive, multifunctional, and alternate monotherapy, is an interesting challenge regarding many patients with migraine, the complicated pathophysiology of migraine, the unknown or varied mechanisms of action of migraine-related, available monotherapies or add-on therapies, and their varied adverse effect profile. Objectives and methods: a five-group, single-blind, and randomized design with pre-post-test and 6-month assessments was used to test the effectiveness of tDCS in the patients with chronic migraine. Using ICHD-3, patients were randomly assigned to one of the study groups to receive 11 consecutive weeks (i.e., 24 sessions; each session = two montages; each montage = 20-min /2mA) of tDCS with four protocols and eight bipolar montages: (1) protocol [F8 (anode)-FC5 (Cathode) plus C4 (anode)-FCz (cathode)] (n = 30); (2) protocol [F8 (cathode)-FC5 (anode) ) plus C4 (Cathode)-FCz (anode) )] (n = 30); (3) protocol [O1 (cathode)-O2 (anode) ) plus C3 (anode)-FCz (cathode) )] (n = 30); (4) protocol [O1 (anode)-O2 (cathode) ) plus C3 (cathode)-FCz (anode) )] (n = 30); or (5) receiving sham-tDCS (n = 30) group.

Results: The results of a series of MANCOVA showed significant reduction in the frequency, duration, and intensity of pain in the experimental groups compared to the sham-tDCS group.

Conclusion: The results suggest that tDCS can be used as an alternate monotherapy in the treatment of patients with chronic migraine.

## P0356 Polypharmacy in cervicogenic headache - A case report

### K. Shimohata^1^, T. Shimohata^2^

#### ^1^Kameda-Daiichi hospital, Department of Anesthesiology, Niigata, Japan; ^2^Gifu University Graduate School of Medicie, Department of Neurology, Gifu, Japan

##### **Correspondence:** K. Shimohata

Objective**:** Cervicogenic headache (CH), which is caused by cervical spinal diseases, is reportedly resistant to pharmacological treatment. The objective was to describe a case of CH in an elderly patient with polypharmacy.

Methods: A case report.

Results: A 90-year-old man had experienced dull, continuous, left occipital pain and neck pain with restriction of range of motion 4 years earlier. Although he was diagnosed with occipital neuralgia and tension-type headache and treated with acetaminophen, celecoxib, tizanidine, etizolam, pregabalin, lomerizine and tofisopam, these were ineffective. He was diagnosed with cervical spondylosis and radiculopathy (left C2) by a spine surgeon and was referred to our outpatient pain clinic. We diagnosed him with CH according to ICHD3 and performed a great occipital nerve (GON) block. He had complete headache relief without adverse effects. 2 years later, he had a chronic daily headache and visited our clinic again. Lightheadedness and hypotension were also observed. As adverse effects of polypharmacy were thought, we discontinued all his medications except pregabalin. After the symptoms had resolved, we performed a GON block and achieved complete pain relief. He was subjected to GON block on a weekly basis for 3 weeks, and his headache improved significantly.

Conclusion: CH has a risk of polypharmacy because of its resistance to pharmacological therapy. GON block could be a safe and effective alternative therapy for elderly patients with CH.

## P0357 Withdrawn

## P0358 Bilateral Subdural Hematoma due to intracranial hypotension- A case report of cervical spinal CSF leak

### M. Papajani^1^, A. Rroji^1^, S. Grabova^1^, B. Qazimllari^1^, A. Xhumari^2,1^, J. Kruja^2,1^

#### ^1^UHC Mother Teresa, Tirana, Albania; ^2^Faculty of Medicine, University of Medicine, Tirana, Neurology, Tirana, Albania

##### **Correspondence:** M. Papajani

Headache is a common complaint but intracranial hypotension (IH) is a rare cause often misdiagnosed. We present a case of low CSF pressure headache resulting from a CSF leak.

A middle-aged woman with a 2-week history of persistent frontotemporal headache associated with photophonophobia, nausea, vertigo, bilateral tinnitus, following an episode of neck trauma. A head CT revealed blood at Sylvian fissure and SAH was suspected. She had a normal neurologic examination on admission. CTA showed a left subdural hematoma (SDH) without vassal abnormality. She had a minor head trauma 2 months ago on the left parietal side and untreated hypertension. She left the hospital in good condition.

The patient was readmitted to hospital 8 days later with intensive headache persisting in the lying position and enhanced after the slightest changes of head position. A control head CT showed bilateral SDH. Laboratory and blood coagulation workup was unremarkable. Brain MRI revealed signs of intracranial hypotension and cervico-thoracal MRI show extradural liquor collection at the C1-C2 level. We concluded that SDH was a complication of IH due to CSF leakage. As the conservative treatment was not effective, she underwent blood patch therapy with excellent outcomes.

Reviewing the literature, we emphasize that IH should be highly suspected in all patients presenting with bilateral or recurrent SDH, as well as in middle-aged patients with new-onset, daily persistent headaches.

**Fig. 1 (abstract P0358).**
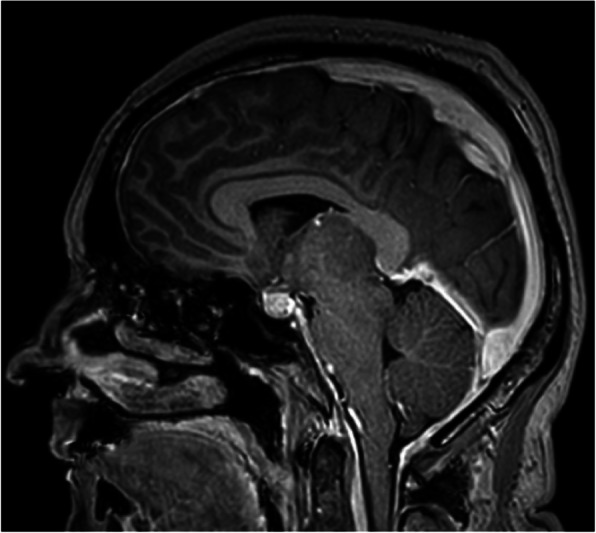
See text for description

**Fig. 2 (abstract P0358).**
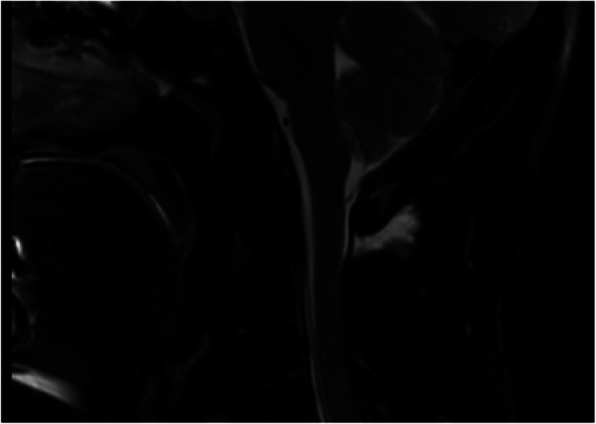
See text for description

## P0359 Steroids response of headache in multiple cranial neuropathies in Mexican population

### R. J. Garcia-Bermudez, J. A. Hernandez-Dominguez

#### Instituto Mexicano del Seguro Social, Neurology, Mexico City, Mexico

##### **Correspondence:** R. J. Garcia-Bermudez

Background and Objective: Headache is one of the main reasons for consultation in neurology^1^. Multiple cranial neuropathies (MCN) is the dysfunction of homologous or different nerves in the same or contralateral side. Steroids could be used because of lack of studies about this pathology, with responses depending on the etiology^2,3^. There are no studies about steroids response in MCN in Mexican population, so we want to establish if headache as initial manifestation of MCN has a good steroids response.

Methods: We carried out an observational, case series, analytical, and retrospective study in Neurology service at Centro Medico Nacional Siglo XXI, in Mexico City. Information was collected from medical records of patients with MCN from January 2015 to December 2020, obtaining the proportion of patients with headache and steroids response, performing Fisher exact method.

Results: 25 patients were included (Table 1). 11 (44%) had headache as initial manifestation, with remission of headache in 10 (90.9%) after steroids; in the other 14 (56%), only 7 (50%) had. OR 10, 95%CI 0.99-100.4 (p 0.04).

Conclusions: Headache as initial manifestation of MCN has a better response than headache after other symptoms in Mexican population, which could lead to a treatment algorithm in this pathology. Further studies with a larger sample are required in order to prove these results.

References

^1^ Postgrad Med 1995;98:197-208.

^2^ Semin Neurol 2009;29:53-65.

^3^ Arch Neurol 2005;62:1714-1717.

**Fig. 1 (abstract P0359).**
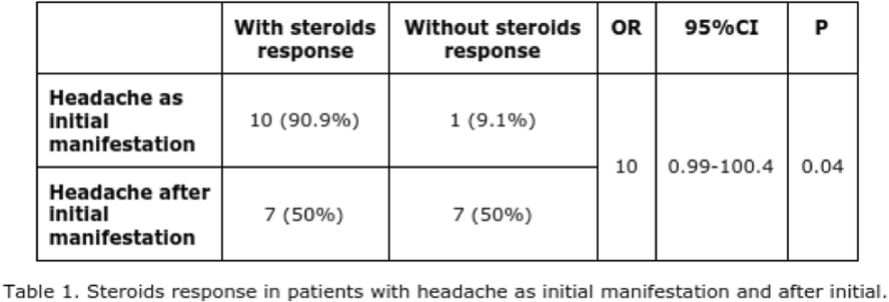
See text for description

## P0360 Idiopathic intracranial hypertension associated with iron-deficiency anemia: A case report

### I. Spanou, E. Chalkiadaki, D. Matsaridis

#### 251 Air Force General Hospital, Neurology, Athens, Greece

##### **Correspondence:** I. Spanou

Objective: Although idiopathic intracranial hypertension (IIH) and iron deficiency anemia (IDA) are both common conditions in women of childbearing age, there is growing evidence suggesting more than an incidental association.

Methods: We present a case of severe IDA presenting as IIH.

Results: A 44-year-old female presented with recent onset headache, bilateral blurred vision and pulsatile tinnitus. She complained about a daily headache of moderate intensity that awaken her from sleep and improved immediately when standing up. Past medical history was remarkable for obesity and chronic menorrhagia. Ophthalmologic examination revealed bilateral decreased visual acuity and papilledema while rest neurological examination was unremarkable. Brain magnetic resonance imaging indicated an empty sella, prominent subarachnoid space around optic nerves and vertical tortuosity of them. Venous sinuses were normal. Cerebrospinal fluid analysis was normal with an opening pressure of 25cmH_2_O. Blood tests revealed iron deficiency anemia with hemoglobin 6.8 g/dl and hematocrit 22.8%. She received a single unit of blood transfusion, intravenous iron supplementation and acetazolamide. Within 1 month disk edema had completely resolved. Acetazolamide was discontinued 4 months later that patient"s body mass index returned within normal limits.

Conclusion: Physicians must be aware of the rare association of IDA with IIH, as the rapid correction of anemia may be vision-saving, preventing disease recurrence.

## P0361 Chiari 1 malformation-related headache in pre-school-aged children

### F. Ursitti^1^, L. Papetti^1^, M. A. N. Ferilli^1^, G. Sforza^1^, R. Moavero^1,2^, S. Tarantino^1^, C. Ruscitto^1,2^, F. Vigevano^1^, M. Valeriani^1,3^

#### ^1^Ospedale Pediatrico Bambino Gesù, Neurologia, Rome, Italy; ^2^Tor Vergata University, Child Neurology and Psychiatry Unit, Rome, Italy; ^3^Aalborg University, Center for Sensory-Motor Interaction, Neurology Unit, Aalborg, Denmark

##### **Correspondence:** F. Ursitti

Background and objective: Chiari I malformation (CM1) is common incidental finding. In few cases it causes symptoms. Surgery is recommended in symptomatic CM1.

Methods: female,2 years/6 months, had headache in neck, daily frequency, during running and laughter. No response to paracetamol. Brain MRI showed CM1(13 mm). She continued follow up with clinical examination. A female,2 years/8 months, had headache in occipital region. Frequency was 5 times month. Coughs triggered headaches. Paracetamol no efficacy. Brain MRI showed CM1(17 mm); spinal cord MRI showed cervical alteration. She was undergoing surgery with improvement. A male,4 years/10 months, had headache in occipital region with pallor, vomiting and photophobia. Mother suffered of migraine. MRI showed CM1(9 mm). For elevated frequency and increased of cerebrospinal fluid in optic nerve he was undergoing surgery, but headache was reappearing.

Results: treatment of CM1-related headaches is difficult because pain in occipital region or coughing headache suggests symptomatic CM1, but children may suffer migraine or tension-type headache. In our cases age of onset, clinical characteristic and triggers of headache are principal factors that could be considerate to perform neuroimaging.

Conclusions**:** onset of headache at an early age, occipital localization during cough and absence of familial history lead to suppose secondary causes, as CM1.This can be treated surgically, although not in all cases headache resolves after surgery

## P0362 Neurological presentation of spontaneous skull base defects: Retrospective study

### Y. Orlova^1^, G. Kalamangalam^1^, S. Chen^2^, J. Poynter^2^, J. Justice^2^, B. Lobo^2^

#### ^1^University of Florida, Neurology, Gainesville, FL, United States; ^2^University of Florida, Otolaryngology, Gainesville, FL, United States

##### **Correspondence:** Y. Orlova

Background: The relationship between spontaneous defects of the skull base, encephalocele and idiopathic intracranial hypertension (IIH) remains poorly understood.

Methods: We performed a retrospective chart review of patients with spontaneous skull base defects and encephalocele at our institution during 2010-2019.

Results: In a pilot analysis of 43 patients (37 women, 9 men), 37 patients (79.1%) presented with craniofacial pain, 21 hearing loss (48.8%) and 20 with middle ear effusion (46.5%). 9 patients (20.1%) had a history of meningitis. 34 had surgical repair of skull base defects, but only 28 had overt clinical signs of CSF leak.

Of 16 patients evaluated by neurologists, 10 had headache disorder (migraine in 5, secondary headache in 4, unspecified in 2, trigeminal neuralgia in 1). IIH was diagnosed in 7, but only 2 met formal diagnostic criteria, see details in Table 1. Three patients had epilepsy. CSF opening pressure was documented in 7 patients and was normal in 4 (11-18 cm H2O) and elevated in 3 (26-47 cm H2O). Most radiological studies did not comment on imaging signs of raised intracranial pressure.

Conclusion: Spontaneous encephalocele can lead to a variety of neurological presentations. Only a minority of patients appear to meet diagnostic criteria for IIH. A further analysis of our entire cohort is in progress.

**Fig. 1 (abstract P0362).**
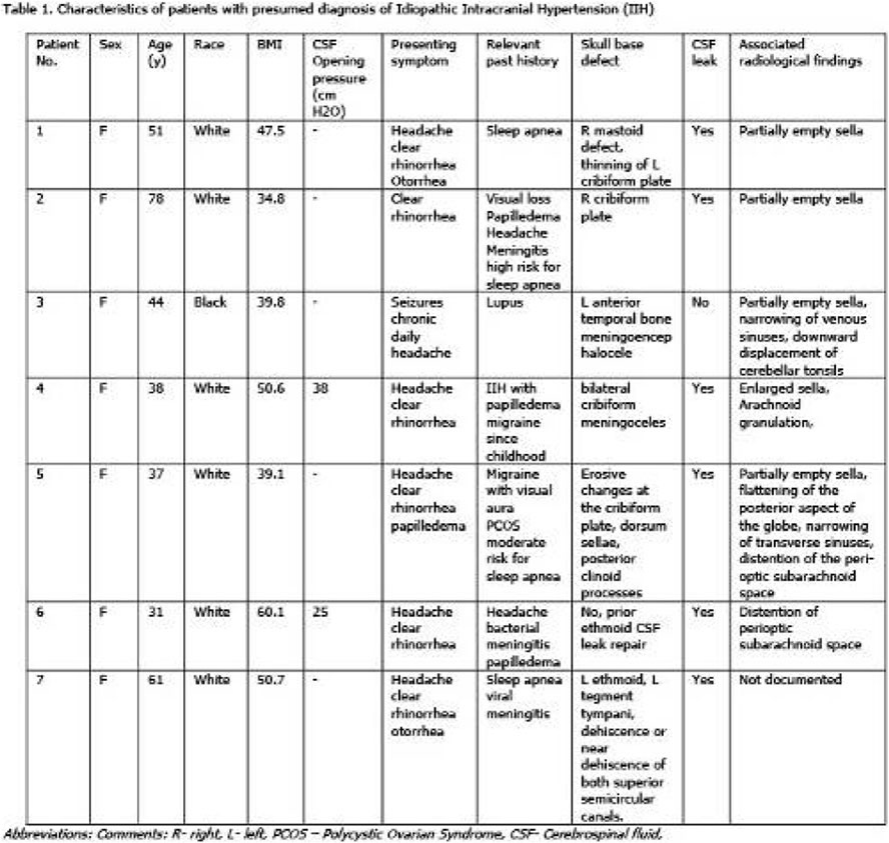
See text for description

## P0363 Spontaneous Intracranial Hypotension secondary to a dual mechanism of cerebrospinal fluid leak: a case report

### F. R. Santos, D. Menendez, C. Guariglia, R. Valeriano, M. Shibuya, D. Setuguti, G. Kuster, M. Calderaro, M. N. Souza, F. Tescari, M. Jordão, R. Pincerato, C. Damasceno

#### Hospital Samaritano Higienópolis, São Paulo, Brazil

##### **Correspondence:** F. R. Santos

Objective: To report a case of headache secondary to a cerebrospinal fluid (CSF) venous fistula with an associated dural tear.

Methods: case description and presentation of neuroimaging findings.

Results: A 47-years-old female presented with continuous orthostatic headache, bilateral, predominating in occipital, irradiating to vertex, photophobia, phonophobia, and nausea. The neurological examination was normal. Brain MRI revealed indirect signs of spontaneous intracranial hypotension – brain sagging, pituitary enlargement, and hyperemia, flattening of the anterior pons, venous sinus dilatation, and enhancement of the pachymeninges. A blind L1-L2 blood patch was performed with a transient improvement followed by worsening. Dynamic CT myelography was conducted and showed contrast in a paravertebral vein at T09-T10 level. Digital subtraction myelography (DSM) confirmed the CSF venous fistula. During surgery, a network of engorged epidural veins was treated with bipolar coagulation and clipping. Another intraoperative finding was a dural tear surrounding the T9 nerve root, which was obliterated with a muscle patch and fibrin glue. The patient became symptom-free one week after the procedure.

Conclusions: CSF-venous fistula is a treatable cause of Spontaneous Intracranial Hypotension with increasing recognition with invasive neuroimaging technique improvements. To our knowledge, we describe the first case of a dual mechanism of CSF loss (CSF venous fistula plus dural tear).

## P0364 Headache related to endovascular thrombectomy: a prospective study

### D. Gallo, L. Manrique, M. Polanco, E. Torres, E. Palacio, J. L. Vázquez, S. Pérez-Pereda, V. González-Quintanilla, J. Pascual

#### University Hospital Marqués de Valdecilla and University of Cantabria, Neurology, Santander, Spain

##### **Correspondence:** J. Pascual

Objective: To study headache in thrombectomy.

Method: We prospectively evaluated clinical features of headache after endovascular thrombectomy using an ad hoc questionnaire in consecutive patients who underwent endovascular thrombectomy during one year.

Results: 117 thrombectomies were performed (mean age 68 years; 55% female). Most patients had anterior circulation strokes (105; 89,74%). Mean NIHSS score pre and post-procedure was 13 and 6, respectively. 93 patients (79,5%) received general anaesthesia (average 45.6 minutes) and 62.4% required stent or aspiration thrombectomy. 33 (28,2%) had headache related to the procedure. There was a higher prevalence of previous primary headache (24,3% vs 7,1%), female sex (60% vs 40%) and posterior circulation strokes in the headache group. No differences were observed in the ASPECTS and NIHSS scores or in the procedure complexity. Headache locations concurred with the affected artery territory and were usually ipsilateral, although headache was bilateral in 34% of cases, mostly oppressive, with a mean duration of 2-3 days and moderate-severe intensity.

Conclusions: One-third of patients who underwent endovascular thrombectomy had procedural headache. Female sex, history of primary headache and posterior circulation stroke were associated with headache occurrence. Procedural complexity was not associated with headache. Headache after endovascular thrombectomy meets the ICHD-3 criteria for headache caused by endovascular procedures.

## P0365 Headache features in multiple sclerosis patients

### M. Bozhenko, T. Nehrych, N. Bozhenko

#### Danylo Halytsky Lviv National Medical University, Neurology department, Lviv, Ukraine

##### **Correspondence:** M. Bozhenko

Objective: Headache is one of the most common complaints among patients with MS. But the relationship between headache and MS still not clearly understood. It is very difficult to determine whether this headache is primary or secondary to MS.

Methods: 120 MS patients with a median disease duration of 6[2,75; 12] years were examined. 70,83% of patients were females. Each patient headache was diagnosed according to the ICHD-3 criteria based on the detailed analysis. Quality of life was assessed with the SF-36 questionnaire and the neuropathic component of pain with Pain Detect.

Results: 51,7% of examined patients had headaches during the last month. 24,17% had a headache that meets the criteria of tension-type headache (for 41,4% it was the most disturbing symptom (MDS) of MS), 6,7%-migraine (for 62,5% MDS of MS), in 20,83% of patient"s headache didn"t meet the criteria of primary headache. 35,6% of patients with headaches had characteristics of neuropathic pain with burning sensation and hyperesthesia. 19,2% of patients consider headache as their first manifestation of MS. Also, MS patients with headaches had lower mental health component of quality of life.

Conclusion: Headache can be the first and the most disturbing symptom of MS. A detailed history of MS and headache are important to determine the type of their relationship. In some patients, its characteristics do not meet the criteria of primary headache and may have the characteristics of neuropathic pain.

## P0366 Pain characteristic of Cervicogenic headache in Tertiary Referral Hospital

### D. A. Sudibyo, M. H. Machfoed, I. Suharjanti

#### Medical Faculty Airlangga University/ Dr. Soetomo General Hospital Surabaya, Indonesia, Neurology, Surabaya, Indonesia

##### **Correspondence:** D. A. Sudibyo

Background and objective: Cervicogenic headache is defined by the International Classification of Headache Disorders (ICHD-3) as headache caused by a disorder of the cervical spine and its component bony, disc and/or soft tissue elements, usually but not invariably accompanied by neck pain. Pain arising from cervicogenic headache comes from nociceptive, neuropathic or both components. We want to evaluate pain characteristic of cervicogenic headache using painDETECT questionnaire.

Methods: Preliminary study was held from June to August 2020 during pandemic covid-19 at Neurology Outpatient Clinic, Dr. Soetomo General Hospital (a Tertiary Referral Hospital) Surabaya, Indonesia. Pain type examination was measured with painDETECT questionnaire. A score of 12 indicates that pain is unlikely to have a neuropathic component (< 15%), while a score of 19 suggests that pain is likely to have a neuropathic component (> 90%). A score between these values indicates that the result is uncertain and required more detailed examination.

Results: There were 30 subjects with cervicogenic headache, 20% male and 80% female, age ranging from 20 to 70 years old. We found 13.33% cervicogenic headache patient with neuropathic pain component, 36.67% with mixed pain component and 50% with nociceptive pain component measured with painDETECT questionnaire.

Conclusions: Most of cervicogenic headache patient comes with nociceptive pain component.

Keywords:

cervicogenic headache, neuropathic, nociceptive

## P0367 Specific characteristics of cerebral arteriovenous malformations manifested with headache

### O. Tsurkalenko, L. Dzyak

#### State Institution "Dnipropetrovsk medical academy of Ministry of Health of Ukraine", Neurology and Neurosurgery, Dnipro, Ukraine

##### **Correspondence:** O. Tsurkalenko

Background and objective: It has been widely described that a large percentage of people with cerebral arteriovenous malformations (cAVM) have headache. However, the specific characteristics of cAVM, assotiated with headache and the mechanisms of it occurrence are still poorly described. The purpose of this study was to identify the specific characteristics of cerebral arteriovenous malformations (cAVM) manifistated with headache.

Methods: A comprehensive clinical, neuropsychological and neuroimaging examination of 398 patients with cAVM were done between 2010 and 2020 years.

Results: Among the studied patients headache was found in 68%, in 49% it was the first symptom of the disease. Clinical and radiological characteristics of cAVM showed that headache occurred significantly more frequently among larger AVM (with vs. without headache, 13.4 vs. 4.9 ml, p<0,002), diffuse AVM (76.3 vs. 21.1%, p<0.002), AVM with transdural arterial communication (87.2 vs. 23.5%, p<0.001), occipital AVM (70.1 vs. 26.8%, p<0.001), older patients (44.7, p<0.037). CT-perfusion of patients with headache showed reduced flow through structurally normal brain region remote from cAVM. These changes were accompanied by cognitive impairment.

Conclusions: The pathogenesis of headache in cAVM patients may involve several mechanisms, including steel-fenomen, cortical spread depression, increased intracranial pressure and demonstrate general lesion of the brain vessels caused by arteriovenous shunting.

## P0368 Clinical findings of headaches in patients with ischemic stroke. First Mexican series

### A. Marfil, F. Gongora, L. Fernandez, M. Cristobal, A. Garcia

#### Autonomous University of Nuevo Leon, Neurology Service, Monterrey, Mexico

##### **Correspondence:** A. Marfil

Introduction: Headache in ischemic stroke (IS) has an incidence of 8-34%. The clinical description varies between series. It is generally accepted that IS in the posterior circulation present with headaches more frequently and the characteristics are more similar to a tension-type headache. As far as we know, no series of IS in Mexico has studied, ad hoc, headaches. The aim is to report the clinical characteristics of headaches as part of IS in a Mexican population.

Methods: iReNe (i-Registro Neurovascular) is a database that gathers information on IS in our institution. In September 2018 we began to collect information on headaches, as well as to perform a paraclinical neurovascular evaluation.

Results: Of 282 patients, 45(16%) had a headache. Headache as the initial manifestation occurred in 21(47%). The pain was oppressive and stabbing in 13 cases each (28%). Immediate zenith occurred in 16(35%) and 6 minutes-4 hours in 9(20%). Bilateral location in 29(64%). Presence of accompanying symptoms in 32(71%), with nausea in 21(46%). Anterior topography was in 33 and posterior in 12. There was no correlation between headache and IS topography.

Conclusion: This is the first series of its kind in Mexico. Most of the headaches started with the other manifestations. No characteristic clinical profile or topographic correlation with IS was found. We conclude that headache is not unusual but, in our series, it does not add localization value.

## P0369 Headache due to late onset hydrocephalus

### M. Bralic, V. Vuletic

#### Clinical Hospital Center Rijeka, Dept. of Neurology, Rijeka, Croatia

##### **Correspondence:** M. Bralic

Tuberculous meningitis (TBM) represents the most serious form of tuberculosis with significant morbidity and mortality. Hydrocephalus, a known complication, can occur either early or late in the clinical course. It has been reported in 87%–90% of children with TBM, whereas it is seen in about 12% of adults. Hydrocephalus could be either of the communicating type or the obstructive type with the former being more frequently seen.

We present an interesting case of patient with worsening headache due to late onset hydrocephalus. A 21-year-old man complains of holocephalic headache that worsened over the period of several weeks and was followed with dizziness, memory and concentration disturbance. According to his medical history he was diagnosed and treated of TBM in the age 14. A cranial CT revealed internal hydrocephalus. Thoracic X-rays taken in the supine position were normal. The cranial MRI displayed restricted third ventricle with widening of the ventral horns of the lateral ventricles as a sign of aqueductal stenosis. Patient was transferred to neurosurgical department where he underwent shunt surgery.

In our case, the patient developed late onset hydrocephalus, thus, it can be hypothesized that subclinical relapse of the disease was cause of it. Our case proves that in the case of a secondary headache unusual causes, but according to the patient's medical history possible headache causes deserve to be taken into consideration.

## P0370 Perceived stress and pain severity in individuals with chronic migraine: A longitudinal cohort study using daily prospective diary data

### M. Vives-Mestres^1,2^, A. Casanova^1^, A. Hershey^3,4^, S. L. Orr^5,6^

#### ^1^Curelator, Inc, Cambridge, MA, United States; ^2^Universitat de Girona, Girona, Spain; ^3^Cincinnati Children's Hospital, Neurology, Cincinnati, OH, United States; ^4^University of Cincinnati, Department of Pediatrics, Cincinnati, OH, United States; ^5^Alberta Children's Hospital, Neurology, Calgary, AB, Canada; ^6^University of Calgary, Departments of Pediatrics, Community Health Sciences, and Clinical Neuroscience, Calgary, AB, Canada

##### **Correspondence:** S. L. Orr

Background**:** To describe patterns of peak pain severity from day-to-day, and in relation to perceived stress, in individuals with chronic migraine (CM).

Methods**:** This was a prospective longitudinal cohort study among adults with CM. Daily data about headache, symptoms, and lifestyle factors were collected using the N1-Headache™ digital health platform for 90 days. Days were classified as "migraine days" according to ICHD criteria. Perceived stress was measured on a 0-10 rating scale. On "migraine days", peak pain severity was recorded on a 4-point categorical scale. A logit ordinal model with random effects for intercept and slope was used to assess the relationship between peak severity and stress, adjusting for gender, age, continuous headache, menstrual bleeding, day of the week, and disability.

Findings**:** Data on 136 participants with 8,216 migraine days were analyzed. Sixty-nine percent of participants (94/136) reported the same peak severity on the majority (>50%) of their migraine days. For every unit increase in stress, the odds of reporting a higher peak severity were 10% higher (OR[95%CI]=1.10[1.07-1.14]). The inclusion of random effects for the intercept and slope improved the model and showed that there were large differences in individuals' reporting of peak severity and in its relationship to stress.

Interpretation: While overall higher perceived stress was associated with higher peak severity, there is a substantial amount of variation between individuals.

**Fig. 1 (abstract P0370).**
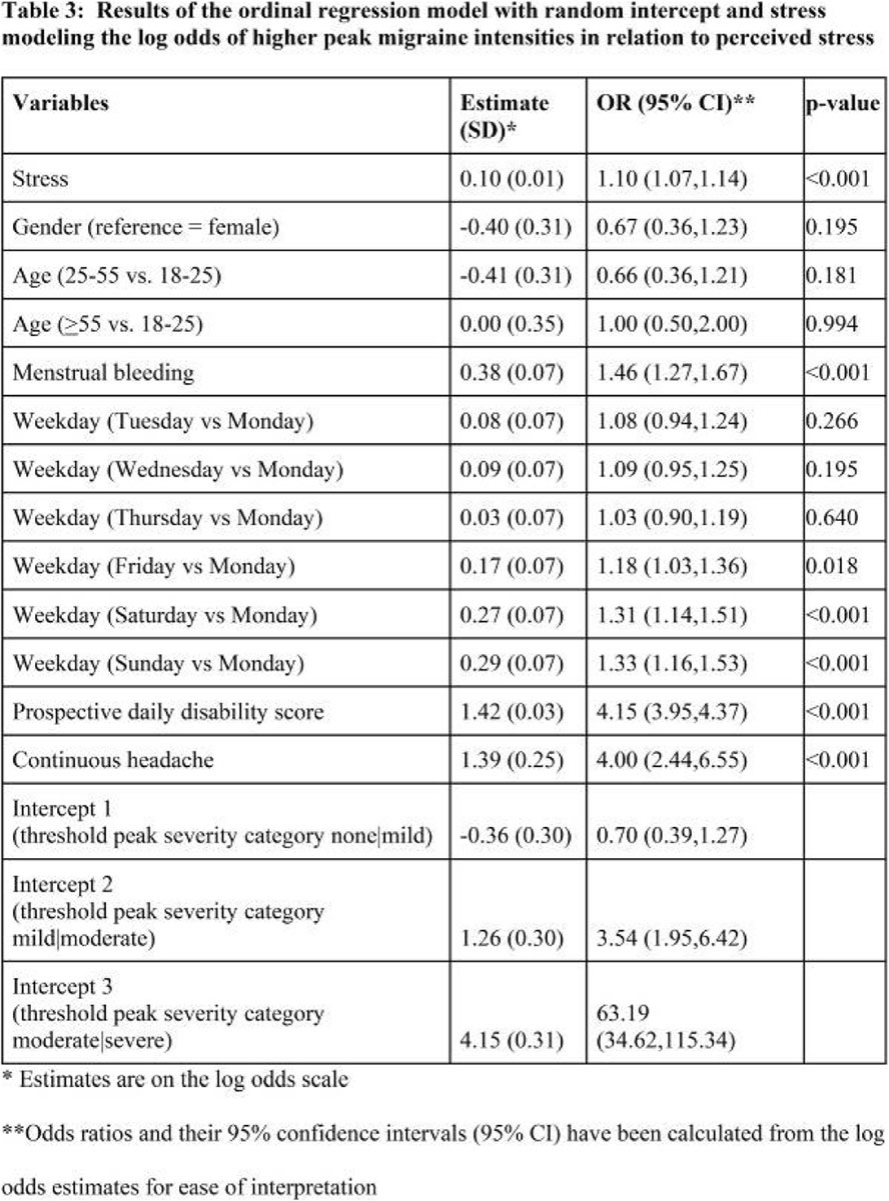
See text for description

**Fig. 2 (abstract P0370).**
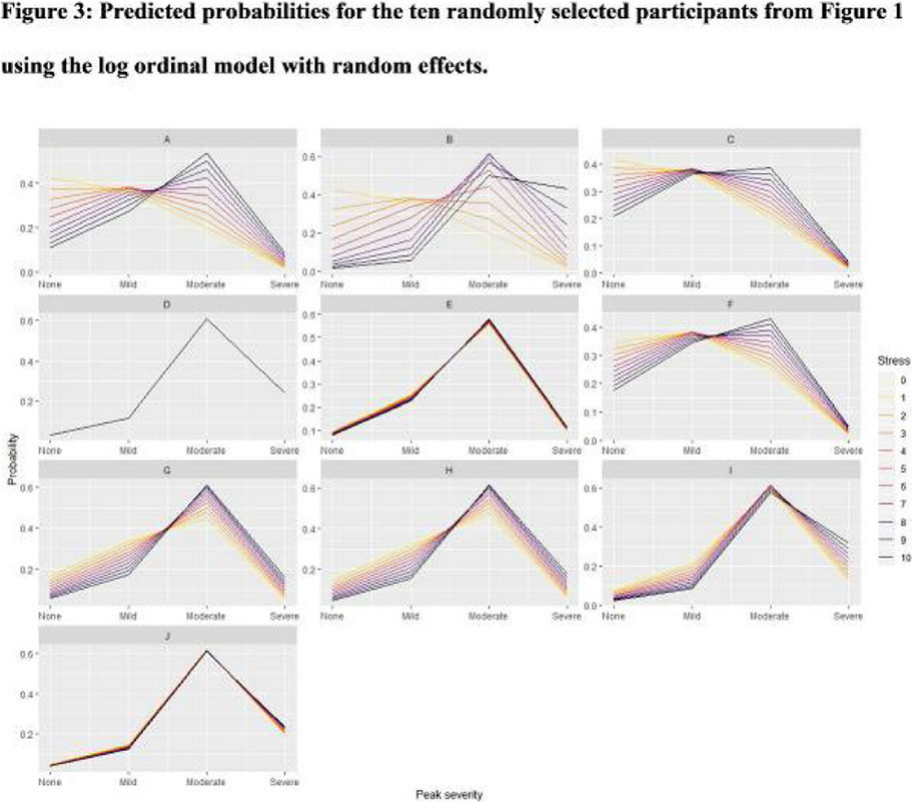
See text for description

## P0371 The influence of coping-strategies and anxiety on clinical course of migraine

### N. Starikova, Y. Karakulova

#### E.A. Vagner Perm State Medical University, Neurology, Perm, Russian Federation

##### **Correspondence:** N. Starikova

Objective: To study the association of pain coping strategies (CS) and anxiety levels of patients with clinical course of migraine.

Patients and methods: 130 migraine patients of the tertiary headache center aged 18-56 and 15 healthy controls of matching age were included in open-label cross-section study. 100-point VAS, Vanderbielt"s CS Questionnaire, Spielberger"s and Beck"s Inventories, MIDAS, M-ACT questionnaire, Migraine-specific QoL questionnaire QVM, Gotheborg QoL Inventory (GQI) were used. Statistical analysis of the data was performed with STATISTICA software.

Results: Migraine patients chose passive CS more frequently than controls (p=0,000). Passive CS of patients significantly correlated with attacks" duration (R=0,221, p=0,028), pain intensity (R=0,222, p=0,027), treatment efficacy (R=-0,250, p=0,019), MIDAS score (R=0,312, p=0,002). The choice of passive coping-strategies significantly (p=0,003-0,000) correlated with poor QoL according to both QoL questionnaires. The Me scores of trait anxiety in migraine patients were elevated (42,00; CI 41,12-44,38) compared with controls (38,00 (CI 32,97-42,62), р=0,046). Trait anxiety scores positively correlated with MIDAS score (R=0,312; p=0,0006) and analgesics intake (R=0,203; p=0,026), and negatively – with the efficiency of analgesia depending on M-ACT questionnaire (R=-0,264; p=0,005).

Conclusion**:** Coping strategies of patients and anxiety scores significantly correlate with clinical course of migraine.

## P0372 Caffeine Use, Migraine Symptoms, and Comorbidities in Migraine Patients at a University Headache Clinic

### N. Murinova^1^, M. Dyess^1^, M. Chan-Goh^1^, M. Bigal^1^, A. Cuneo^1^, D. Krashin^2^

#### ^1^University of Washington, Neurology, Seattle, WA, United States; ^2^Puget Sound VA, Pain and Psychiatry, Seattle, WA, United States

##### **Correspondence:** N. Murinova

Background:

Most people worldwide consume at least some caffeine. Caffeine can be both a headache trigger and a pain reliever. We examined whether caffeine consumption has a clearly defined relationship to migraine symptoms and comorbidity in our patient population, in a region famous for coffee consumption.

Methods: All new patients referred to the Headache Clinic at the University of Washington complete a detailed patient intake questionnaire that includes questions regarding caffeine use, headache characteristics, sleep, depression, anxiety, and stress. These were analyzed along with headache diagnosis.

Results: Of 5677 unique patients with migraine, 74 % of these had chronic migraine. Caffeine use was identified in 82% of patients. 70% consumed one one or less serving of caffeine per day, while 4% consumed three or more. In chronic migraine, higher caffeine use correlates with increased number of headache days per month. We found no correlation of caffeine use and any migraine comorbidities such as difficulty with sleep, perception of stress and depression measures.

Conclusions: Most of our patients with migraine consume caffeine. We found a correlation with headache days in chronic, but not episodic migraine patients, and no correlation with comorbidities in any patients, which was surprising. These findings may mean that caffeine consumption is not a significant migraine trigger, or else that patients limit their caffeine consumption to avoid this.

## P0373 Alexithymia and psychological distress in chronic migraine and fibromyalgia: A comparative study

### S. Bottiroli^1,2^, A. Ghiggia^3,4^, F. Galli^5^, L. Castelli^3,4^, G. Sances^1^, E. Guaschino^1^, M. Allena^1^, C. Tassorelli^1,6^

#### ^1^IRCCS Mondino Foundation, Headache Science and Neurorehabilitation Centre, Pavia, Italy; ^2^Giustino Fortunato University, Faculty of Law, Benevento, Italy; ^3^University of Turin, Department of Psychology, Torino, Italy; ^4^AOU Città della Salute e della Scienza di Torino, Clinical Psychology Unit, Torino, Italy; ^5^Sapienza University of Rome, Department of Dynamic and Clinical Psychology, and Health Studies-Faculty of Medicine and Psychology, Rome, Italy; ^6^University of Pavia, Department of Brain and Behavioral Sciences,, Pavia, Italy

##### **Correspondence:** S. Bottiroli

Background and objective: Alexithymia is a personality trait characterized by the inability to identify and express emotions. Several studies evidenced a positive association between alexithymia and psychological distress in patients with chronic migraine (CM) and fibromyalgia (FM). Here we evaluated the prevalence of alexithymia and distress in FM and CM, compared to healthy controls (HC).

Methods: Two-hundred and fifty women with CM (age: 46.1±11.5, disease duration: 7.9±7.3 yrs) and 250 FM (age: 51.2±10.5, disease duration: 7.9±7.8 yrs) were assessed by the Toronto Alexithymia Scale (TAS-20), and the Hospital Anxiety and Depression Scale (HADS). A HC group (n=280; age: 51.8±9.0) was also enrolled and assessed by TAS-20 and HADS.

Results: FM had significantly higher levels of alexithymia (p < .001) and psychological distress (p < .001) than CM and HC. CM patients reported higher levels compared to HC group in the total score (p < .001) and in the Difficulty Identifying Feeling subscale of the TAS-20 (p < .001). A moderation analysis was performed to examine the moderation effect of the group (CM vs. FM) on the relationship between alexithymia and psychological distress. Besides a strong relationship between alexithymia and distress, the group variable was not a significant moderator.

Conclusions: These findings suggest a common psychological dysregulation in patients suffering from CM and FM, which manifests into a different expression of the physical symptom.

## P0374 Associations between physical activity, quality of life and headache in people with Idiopathic Intracranial Hypertension

### A. Denton^1^, H. Gunn^2^, K. Hemmings^3^

#### ^1^Idiopathic Intracranial Hypertension UK, Exeter, United Kingdom; ^2^University of Plymouth, Plymouth, United Kingdom; ^3^University of Derby, Derby, United Kingdom

##### **Correspondence:** A. Denton

Background and objective**:** Physical activity is reduced in people with headache conditions such as migraine, this has not been explored in people with Idiopathic Intracranial Hypertension (pwIIH). This survey aimed to quantify physical activity and explore relationships between physical activity, health related quality of life (HRQoL), headache impact and other clinical characteristics in pwIIH.

Methods**:** An online questionnaire via IIH UK. Primary measures were physical activity (PASIPD) and HRQoL(SF-36®) with secondary outcome measures of headache impact (HIT-6™), Body Mass Index (BMI) and age.

Results: 164 pwIIH completed the questionnaire. PASIPD measures showed that pwIIH have low levels of physical activity (10.38 (IQR ±17.6) MET hr/day) and a low level of engagement with exercise and muscle strengthening programmes, similar to people with physical disabilities and other headache disorders. Significant moderate correlations were found between PASIPD total score, headache impact (HIT-6™) and HRQoL (Physical component score and sub-categories of SF-36® physical functioning, physical role, general health, vitality and social role) (p<0.05) however there were no significant correlations between PASIPD and Mental component score, age or current BMI(p>0.05).

Conclusion: The results suggest that future research should explore the barriers to physical activity and exercise in pwIIH and find ways of increasing physical activity and engagement with exercise.

## P0375 Neuropsychological changes in children with sickle cell disease and their correlation to the imaging studies. N.B.This abstract belongs to original research article that has been published in "Journal of advances in Medicine and Medical Research"

### M. Badr^1^, M. El-Shanshory^2^

#### ^1^Faculty of Medicine, Tanta University, Neurology, Tanta, Egypt; ^2^Faculty of Medicine, Tanta University, Pediatric, Tanta, Egypt

##### **Correspondence:** M. Badr

Objective**:** To assess neurological and psychological disorders in children with sickle celldisease (SCD) using multimodal approach through clinical, laboratory, neuroimaging and neurophysiological studies in a trial to detect etiological risk factors.

Method**:** This study was conducted on 50 children (27 male and 23 female; age range 2-18years) with SCD and 25 healthy children matched age and sex in Department of Pediatric (Hematology Unit) and Department of Neurology, Tanta University Hospital Egypt, between April 2016 and April 2018. All subjects were subjected to fullhistory taking, neurologic examination using pediatric neurological sheet, laboratory investigations,neuroimaging including: CT and /or MRI, MRA and/or CT angiography, also MRV, TCCD, EEG and Stanford-Binet Intelligence scales-Fifth Edition.

Results**:** Most of patients presented with headache 66%, cognitive decline 48%, seizures 28%, and visual affection 24%. Less common presentations were, ischemic and hemorrhagic stroke 6%and 4% respectively. SCD children showed many abnormalities on neurological examination andon different modalities of MR imaging on the brain with positive correlation (X2=7.641, p-value<0.001*, r=0.248) with many risk factors. Prophylactic blood transfusion in SCD patients with abnormal TCD had a role in reducing the incidence of stroke.

Conclusion**:** Children with SCD were presented with variable neuropsychological disturbances that correlated with the brain imaging.

## P0376 Peripartum Cerebrovascular events from imaging perspective

### M. Maghbooli

#### Zanjan University of Medical Sciences, Neurology, Zanjan, Iran

The average age of pregnancy has increased from 24.6 to 27.2 in the past 30 years, increasing pregnancy-associated complications. As neurological diseases contribute to approximately 20% of maternal deaths, it is important to identify these at risk population. Cerebrovascular complications are classified into ischemic infarctions, subarachnoid hemorrhage, eclamptic encephalopathy, postpartum cerebral angiopathy, and cerebral venous thrombosis.

Some are easily recognized by obstetricians and are managed without significant neurological input unless seizures develop. Others are relatively benign, but should be recognized by neurohospitalists as they are often reasons for consults. Some diseases initially present with nonspecific symptoms such as headache. However, headache is a common complaint in pregnant women and distinguishing the benign headache from one that is a sign of serious disease is often not considered until serious neurological complications develop.

A CT study should be avoided due to a significant increase of the X-ray exposure and to the necessity of administering intravenous contrast unless the information is critical to guide therapy. There is no evidence of adverse fetal effects in humans to the magnetic field exposure for magnetic resonance imaging (MRI).

This review highlight on pearls in neuroimaging findings of main cerebrovascular events in peripartum period.

## P0377 Spontaneous intracranial hypotension, findings, misdiagnosis: a systematic review

### S. Shaafi, E. Sadeghi

#### Tabriz University of medical science, Neurology, Tabriz, Iran

##### **Correspondence:** S. Shaafi

Background: Spontaneous intracranial hypotension (SIH) is a pathology characterized by orthostatic headaches, diffuse pachymeningeal enhancement on magnetic resonance imaging (MRI), and low to normal cerebrospinal fluid pressures. SIH results from a CSF leak secondary to structural weakness in the dura, either at the cervicothoracic junction or the thoracic spine. Patients may develop subdural hematomas or hygromas. Misdiagnosis may delay appropriate treatment and expose the patient to risks of therapeutic interventions for headache mimics. The pathognomonic findings on contrasted MRI brain are diffuse, smooth, pachymeningeal gadolinium enhancement, and brain sagging.

Methods: We have reviewed current literature including published original, review articles, and case reports or case series in PubMed/MEDLINE and other databases using the keywords; Spontaneous, intracranial hypotension, findings, misdiagnosis.

Results: In most of reports emphasis is on that spontaneous intracranial hypotension is an important cause of "new daily persistent headaches". Patients with spontaneous intracranial hypotension are commonly misdiagnosed, causing a significant delay in the initiation of effective treatments.

Conclusion: Many radiologic and clinical findings may mimic these classic findings, and conversely, secondary changes from SIH can give rise to symptoms that imitate other conditions. Because SIH is a curable condition, it is important for physicians to recognize its nonclassic presentations and be familiar with the differential diagnoses of its radiologic and clinical findings.

## P0378 Lasmiditan Over Four Migraine Attacks in Chinese Population: Findings from CENTURION Study

### L. He^1^, T. Yu^2^, X. Yang^3^, Q. Hu^4^, B. Zhang^4^, S. Zhong^4^, S. Yu^5^

#### ^1^West China Hospital of Sichuan University, Department of Neurology, Chengdu, China; ^2^Second Hospital, Jilin University, Department of Neurology, Changchun, China; ^3^Xiangya Hospital, Central South University, Department of Neurology, Changsha, China; ^4^Eli Lilly and Company, LCDDMAC, Shanghai, China; ^5^The General Hospital of People’s Liberation Army (301 hospital), Department of Neurology, Beijing, China

##### **Correspondence:** S. Yu

Objective**:** We present findings in Chinese population from the multicenter, placebo (PBO)-controlled, double-blind Phase 3 study, CENTURION, designed to assess the efficacy, including consistency of response, of lasmiditan (LTN) over 4 migraine attacks.

Methods**:** Patients were randomized 1:1:1 to LTN 200 mg; LTN 100 mg; or a control group receiving PBO for 3 attacks and LTN 50 mg for either the 3^rd^ or 4^th^ attack (1:1). The primary endpoints were pain freedom at 2h (1^st^ attack) and pain freedom at 2h in ≥2/3 attacks.

Results**:** 275 patients (mean age 37.6 years; 72.4% female; MIDAS mean score 36.6) were treated ≥1 migraine attack with study drug (control, n=92; LTN 100 mg, n=91, LTN 200 mg, n=92). Pain freedom rates at 2h for 1^st^ attack were significantly higher in LTN 200 mg (32%, OR [95% CI]=3.1 [1.4, 6.7]) and numerically higher in LTN 100 mg (25%, OR [95% CI]=2.2 [0.98, 4.9]) than PBO (13%), with separation from placebo beginning at 1 hour. Pain freedom rates at 2h for ≥2/3 attacks were significantly higher in LTN 200 mg (25%, OR [95% CI]=3.5 [1.3, 9.5]) and LTN 100 mg (23%, OR [95% CI]=3.2 [1.2, 8.9]) than PBO (9%). The most frequent treatment-emergent adverse events with LTN were dizziness, asthenia, muscular weakness, and somnolence.

Conclusions**:** In Chinese population, LTN was significantly better than PBO for both primary endpoints with an acceptable safety profile. These findings were generally consistent with that observed in CENTURION primary cohort population.

## P0379 Disability and psychosocial features in patients with acute whiplash associated disorders with and without headache: a case-control study

### E. Anarte-Lazo^1^, C. Bernal-Utrera^1^, D. Falla^2^, C. Rodriguez-Blanco^1^

#### ^1^University of Seville, Department of Physiotherapy, Seville, Spain; ^2^University of Birmingham, School of Sports, Exercise and Rehabilitation Sciences, Birmingham, United Kingdom

##### **Correspondence:** E. Anarte-Lazo

Objective: To assess whether there are differences in psychosocial features and disability between people with acute whiplash associated disorders (WAD) who develop headache versus those who do not.

Methods: A case-control study was conducted from September 2020 to February 2021 in a Traumatology Clinic in Madrid, Spain. Among 49 consecutively recruited patients who were assessed, 41 patients were included in this study, 22 and 19 with and without headache, respectively. Visual Analogue Scale (VAS) of neck pain intensity, Neck-Disability Index (NDI), Tampa-Scale Kinesiophobia-11 (TSK-11) and Pain Catastrophizing Scale (PCS) were evaluated.

Results: Baseline differences between groups were found in relation to sex; there were more women in the group with headache (73.7% vs 50% in the non-headache group). No baseline differences were found in age, height, weight and days from the accident to the evaluation. Significant mean differences were found between groups for VAS (23.47 [14.09-32.86]), NDI (10.99 [7.05-14.93]), TSK-11 (9.80 [5.66-13.95]) and PCS (12.64 [7.55-17.53]) revealing greater psychosocial features and greater pain and disability in those with headache.

Conclusion: Our findings showed that psychosocial features and disability were greater in patients with headache following a whiplash injury when compared to those patients with acute WAD who did not develop headache. These findings should be considered and integrated within the management of these patients.

## P0381 Post-traumatic headache: A comprehensive review

### M. Togha, M. Babaei

#### Sina Hospital, School of Medicine, Tehran University of Medical Sciences; Headache department, Irainian Center of Neurological Researches, Institute of Neuroscience, Tehran University of Medical Sciences., Tehran, Iran

##### **Correspondence:** M. Babaei

Headache is a sequel of traumatic head and neck injuries. It has a prevalence of 33-92%, mostly occurring during the first week post-injury, and improves within 3-6 months. It mostly resembles primary headaches including migraine, tension-type headache, trigeminal autonomic cephalgias, and cervicogenic headaches. Several risk factors and underlying mechanisms have been addressed for this type of headache.

Comprehensive patient evaluation and exclusion of serious underlying causes and associated disorders is needed for its management. Red flags, including altered mental status, focal neurological deficit, progressively worsening headache, intractable headache, and headaches caused by Valsalva maneuver or changing position indicate serious underlying causes, requiring appropriate work-up.

When a diagnosis was made, a multidisciplinary therapeutic approach should be performed. Management of comorbidities and proper patient education is also required. In case of headache persistence for 3-6 months after proper therapeutic plans, patients should be referred to a tertiary headache clinic for further evaluations.

## P0382 Ubrogepant Treatment When Pain is Mild Increases the Likelihood of Achieving Pain Freedom in Participants Who Treated Migraine Attacks of Mild and Moderate or Severe Pain

### R. B. Lipton^1^, D. W. Dodick^2^, P. J. Goadsby^3,4^, R. Burstein^5^, A. Adams^6^, J. Lai^7^, S. Y. Yu^7^, M. Finnegan^7^, J. M. Trugman^7^

#### ^1^Albert Einstein College of Medicine, Bronx, NY, United States; ^2^Mayo Clinic, Phoenix, AZ, United States; ^3^NIHR-Wellcome Trust King’s Clinical Research Facility, King’s College, London, United Kingdom; ^4^University of California, Los Angeles, Neurology, Los Angeles, CA, United States; ^5^Harvard Medical School, Beth Israel Deaconess Medical Center, Boston, MA, United States; ^6^AbbVie, Irvine, CA, United States; ^7^AbbVie, Madison, NJ, United States

##### **Correspondence:** R. B. Lipton

Objective: Efficacy of ubrogepant in treating migraine with moderate/severe pain was demonstrated in 2 phase-3 trials. Clinical guidance recommends treatment when pain is mild, a strategy examined here in participants treating ≥1 attack with mild pain and ≥1 attack with moderate/severe pain.

Methods: Phase-3, 52-week trial (NCT02873221). Adults randomized 1:1:1 to usual care, or ubrogepant 50mg or 100mg, treated ≤8 migraine attacks every 4 weeks. Efficacy measures were collected only for ubrogepant.

Results: 459 of 808 participants treated ≥1 attack with mild pain and ≥1 attack with moderate/severe pain and were eligible for this analysis. A higher proportion of attacks with mild pain vs moderate/severe achieved 2-hr pain freedom (50mg: 57.1% vs 30.9%; 100mg: 51.1% vs 27.2%); absence of nausea, photophobia and phonophobia and restoration of normal function all occurred in significantly higher proportions for attacks treated with mild pain (P

Conclusions: Among persons treating both mild and moderate/severe attacks, outcomes were better for attacks treated when pain is mild. Findings in this subgroup extend prior results and further support the recommendation to treat early when pain is mild.

**Fig. 1 (abstract P0382).**
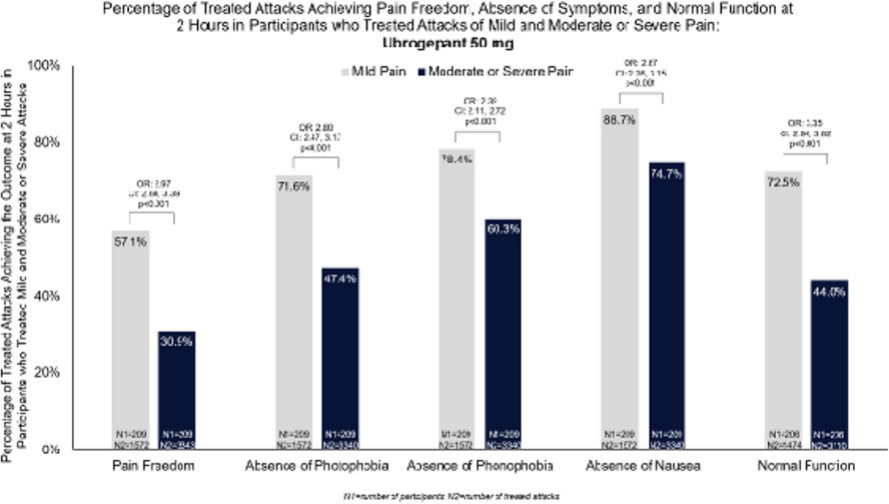
See text for description

**Fig. 2 (abstract P0382).**
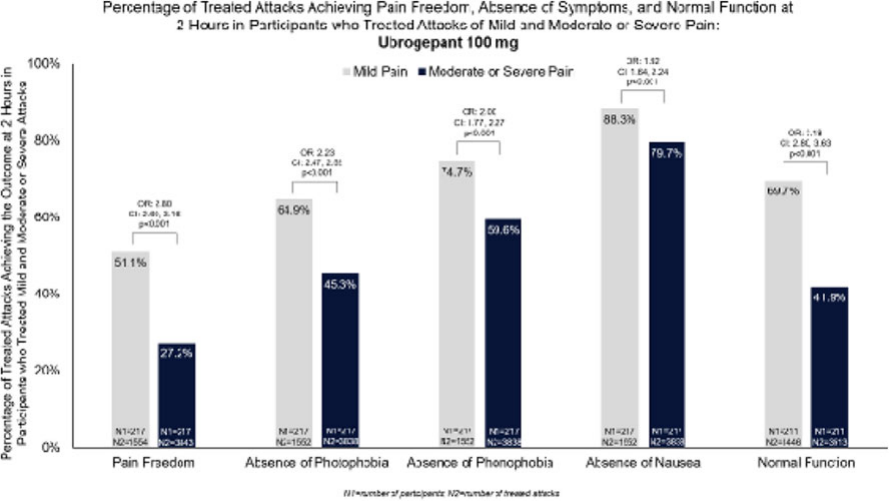
See text for description

## P0383 Prevalence, clinical characteristics of headache in medical students at Alzaiem Alazhari university in 2020, Khartoum, Sudan

### R. Tofaha Alhusseini^1^, M. Ibrahim^2^, M. Alfatih^2^, A. Ahmed^2^

#### ^1^Alzaeim Alazhari University, Medicine, Khartoum, Sudan; ^2^Alzaeim Alazhari University, Medicine, Khartoum, Sudan

##### **Correspondence:** R. Tofaha Alhusseini

Introduction: Headache is one of the most common disorders of the nervous system. Headache means pain in the head. The (WHO) reports that almost half of the adults worldwide will experience headache in any time at any given year.

Objectives: To determine the prevalence rate and clinical characteristics of headache among medical students in Alzaiem Alazhari university in Khartoum state, Sudan in 2020

Methodology: A descriptive cross-sectional study using a 41 items questionnaire was introduced to 71 medical students from Alzaeim Alazhari university in the period from January 1st to 15th of February.

Results: Out of the 71 respondents 35 (49.3%) were Male and 36 (50.7%) were female while most of them were in the (21-24) age group by (69.01%). Most of the participants responded that they headaches (74.65%) with (32.31%) of them having continuous and (67.69%) of them with no continuous headache. 32 (45.07%) of them had headaches, half of them lasted for 1-2 hours and the other half lasted more than 10 hours per day. The most common location for the headache was both sides (23.02%) followed by the fore head (22.22%). The most common characteristic of headache was pulsating (48.48%) followed by pressure like (37.88%).

Conclusion: There is a high prevalence rate of headache among medical students with migraine as the most common cause of headache

## P0384 Migrainous infraction: a case report of a rare and overlooked phenomenon

### P. Gklinos, C. Zachariadi, D. Siakavella, E. Papathanasiou, D. A. Lagka, E. Ieridou, A. Toumasis, M. Nianiarou, E. Makri, G. Aposporos, M. Terzoudi, A. Agathonikou

#### KAT General Hospital of Attica, Department of Neurology, Kifisia, Greece

##### **Correspondence:** P. Gklinos

Background & objective: The objective of this study is to present a case of a patient with migrainous infraction during the course of a typical migraine attack with aura and discuss the clinical presentation, radiological findings, and differential diagnosis of this rare migraine complication.

Methods: A 31-year-old, right-handed male patient with a history of migraine with aura and epilepsy of unknown etiology was admitted to our hospital due to an episode of right-sided numbness involving his right arm and face, as well as expressive aphasia, lasting for 30 minutes, followed by a typical migraine headache. Minutes after presentation at the emergency room (ER), the patient developed two additional transient episodes of Broca"s aphasia that completely resolved within 10 minutes.

Results: Brain computerized tomography (CT) scan, electroencephalography (EEG), and blood tests were unremarkable while the MRI FLAIR sequence revealed a hyperintense signal in the left frontoparietal cortex. The diffusion-weighted image (DWI) sequence demonstrated high signal intensity in the same cortical region with corresponding low value on apparent diffusion coefficient mapping (ADC), which indicated restricted diffusion. The clinical diagnosis was migrainous infraction, and the patient was treated with antiplatelet therapy.

Conclusion: Migrainous infraction is a rare complication of migraine. Prompt diagnosis may improve the outcome of patients and avoid inappropriate management of symptoms.

**Fig. 1 (abstract P0384).**
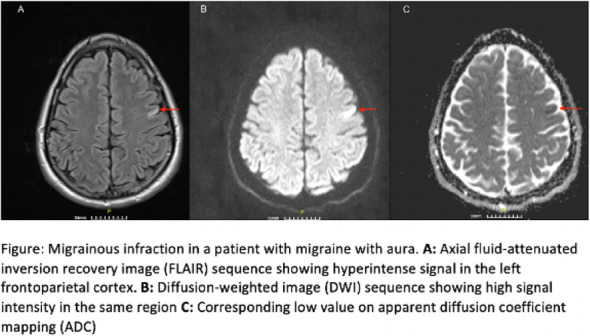
See text for description

## P0385 Primary stabbing headache in children and adolescents: is it a migraine precursor?

### F. Ursitti^1^, L. Papetti^1^, M. A. N. Ferilli^1^, R. Moavero^1,2^, S. Tarantino^1^, G. Sforza^1^, C. Ruscitto^1,2^, F. Vigevano^1^, M. Valeriani^1,3^

#### ^1^Ospedale Pediatrico Bambino Gesù, Neurologia, Rome, Italy; ^2^Tor Vergata University, Child Neurology and Psychiatry Unit, Rome, Italy; ^3^Aalborg University, Center for Sensory-Motor Interaction, Neurology Unit, Aalborg, Denmark

##### **Correspondence:** F. Ursitti

Background and objective: The aims of our study were to describe the clinical features of the pediatric PSH and to investigate whether PSH is related to more common primary headaches.

Methods: Nineteen patients with PSH were recruited (13 girls and 6 boys, aged from 4 to 16 years).

Results: In our patients, pain had usually involved bilateral fronto-temporal region. Four patients failed to identify a precise pain location. Stabs were usually lasting less than 1 minute. In one patient, each attack included several stabs and lasted around 20 minutes. Pain intensity was usually mild to moderate. Strong pain intensity was referred by 2 patients. Eight patients presented with associated symptoms, as photophobia (5), phonophobia (6), and nausea (3). Migraine was associated with PSH in 5 patients and tension-type headache (TTH) in one. Episodic syndromes which may be associated with migraine, as infantile colic, motion sickness, limb pain, recurrent abdominal pain, and vertigos, were referred by 13 patients.

Discussion: In our pediatric case series, PSH clinical features were very similar to those described in adulthood. While in adults PSH is frequently associated with migraine and TTH, only 32% of our patients referred another primary headache. It is noteworthy that 70% of our PSH patients had a history of episodic syndromes.

Conclusion: in pediatric age PSH could represent an age-related phenotype of the migrainous syndrome which will turn later into a more typical migraine.

## P0386 Assessing Readiness for Headache Services in District of Kolar

### A. Dixit^1^, G. N Rao^1^, G. Baburao Kulkarni^2^

#### ^1^National institute of Mental health and Neuro Sciences, Department of epidemiology, Bengaluru, India; ^2^National institute of Mental health and Neuro Sciences, Department of Neurology, Bengaluru, India

##### **Correspondence:** A. Dixit

Background: Headache disorders a major cause of public ill-health, require trained resources for appropriate management and hence, an acute need to institute systematic initiatives and organise services.

Objective: Levels of knowledge and practice regarding the management of Primary headache disorders among Health personnel in Government sector within a district were assessed. The readiness for headache services in the district with respect to planning, training, personnel and support services including availability of drugs was documented.

Methodology: A mixed methodology strategy included eliciting response to Case vignettes along with Key informant Interviews.

Results: 80% of medical officers had correct knowledge for provisional diagnosis in dealing cases of organic conditions; 60% had correctly given provisional diagnosis of Migraine. Non-medical health personnel did not have the desired knowledge and required expertise regarding correct treatment and referral for headache disorders. Discrepancies were observed related to management of individual headache disorders. Further, KIIs indicated that there have been no plans or discussions for introducing headache services. Headache services did not feature in the priority at the district or state level.

Conclusion**:** Despite a proven burden, headache services at the district level is poorly organised. However, opportunities exist aplenty for making headache services at district and sub-district levels more systematic.

**Fig. 1 (abstract P0386).**
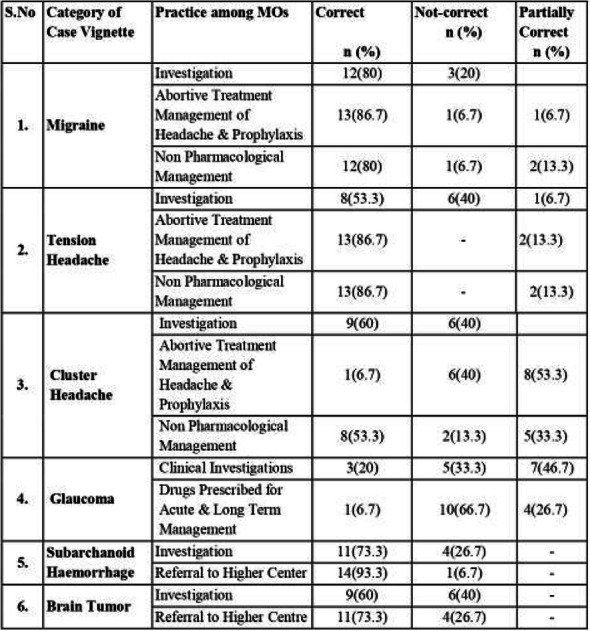
See text for description

## P0387 Lasmiditan is Effective in the Acute Treatment of Migraine in Patients with Insufficient Response to Triptans: Findings from the Modified-parallel, Placebo-controlled, Double-blind, Phase 3 Consistency CENTURION Study

### U. Reuter^1^, L. Lombard^2^, J. Krege^2^, E. Gomez Valderas^2^, J. Krikke-Workel^2^, G. Dell Agnello^2^, S. Dowsett^2^, D. C. Buse^3^

#### ^1^Charité University Hospital Berlin, Department of Neurology, Berlin, Germany; ^2^Eli Lilly and Company, Indiana, IN, United States; ^3^Albert Einstein College of Medicine, New York, NY, United States

##### **Correspondence:** D. C. Buse

Objective: Assessing lasmiditan's efficacy in triptan-insufficient responders (TIR).

Methods: CENTURION randomized patients with migraine with/without aura to lasmiditan (LTN) 200mg/LTN100 for 4 attacks or placebo and LTN50 for 3 and 1 attack. TIR were: subset with inconsistent response to their most recent triptan; taking triptan; had poor/very poor migraine Treatment Optimization Questionnaire(mTOQ-6) score; had discontinued their most recent triptan due to efficacy/tolerability issues/contraindications. Gated secondary endpoint: pain freedom at 2hours (h). Results are for first attack through 2h postdose, sustained effects through 48h and response consistency, defined as reaching outcome at 2h in ≥2/3 attacks.

Results: During first attack, both lasmiditan doses showed statistically significant benefit over placebo for pain freedom and relief starting at 1h and 0.5h(LTN200) or 1h (LTN100)(p<0.05) and consistency of effect across attacks for pain freedom and relief at 2h. Results for pain freedom at 2h(gated): placebo, 8.8%; LTN100, 24%(odds ratio[OR] 3.3[1.8-6.0]; LTN200, 25.6%(OR 3.6[2.0-6.4];p<0.001). Significant differences from placebo were evident for 1/both lasmiditan doses for migraine-related disability freedom at 2h, much/very much better on Patient Global Impression of Change at 2h, most bothersome symptom freedom at 2h, rescue medication need and sustained pain freedom at 24 and 48h(p<0.05).

Conclusions: Lasmiditan was beneficial across various clinical endpoints in TIR.

## P0388 Red ear syndrome and headache: a systematic reviw of literature

### J. Baleki Borri, H. Mariano da Silva Junior, C. Alberto Bordini

#### PUC- Campinas, Medicine, Campinas, Brazil

##### **Correspondence:** J. Baleki Borri; H. Mariano da Silva Junior

Objective: The Red Ear Syndrome (RES) is an enigmatic disorder with approximately 100 published cases in literature. It is characterized by attacks of burning pain and erythema on the ear. RES is classified in idiopathic and secondary forms, often associated to primary headaches and upper cervical disorders respectively. The aim of this paper is to provide an overview of studies which reports this poorly understood condition.

Methods: We review all previously described cases and 53 articles were selected following the Preferred Reporting Items for Systematic Review and Meta-Analysis (PRISMA) protocol. All the 94 patients collected from case reports were placed into idiopathic or secondary RES groups.

Results: In both groups there are a female to male predilection ratio, unilateral attacks are more common, the duration of attacks can range seconds to hours and occur daily. In the idiopathic RES, 48,2% of patients the attacks were associated with primary headaches and 34,4% had isolated attacks, the most common trigger was tactile stimuli. On the other hand, in secondary RES the most common trigger was head movement. Furthermore, in 63,1% of the cases the pain extends to other regions beyond the ear mostly in secondary cases. Patients can also experience autonomic and vestibulocochlear symptoms.

Conclusion: Our systematic review showed important clinical differences between primary and secondary RES. These results could shed light on the knowledge of this peculiar condition.

## P0390 Decreased Neuropeptide Action Associated with Cardiovascular Risk in Migraine Patients with Polycystic Ovary Syndrome (PCOS)

### L. Al-Hassany^1^, B. K. T. Zick^1,2^, K. M. Linstra^1,2^, C. Meun^3^, K. Van der Ham^3^, J. Van den Berg^1^, E. H. Boersma^4^, A. H. J. Danser^1^, B. C. J. Fauser^5^, J. S. Laven^3^, M. J. H. Wermer^2^, G. M. Terwindt^2^, A. Maassen van den Brink^1^

#### ^1^Erasmus University Medical Center, Division of Vascular Medicine and Pharmacology, Department of Internal Medicine, Rotterdam, Netherlands; ^2^Leiden University Medical Center, Department of Neurology, Leiden, Netherlands; ^3^Erasmus University Medical Center, Division of Reproductive Endocrinology and Infertility, Department of Obstetrics and Gynaecology, Rotterdam, Netherlands; ^4^Erasmus University Medical Center, Department of Cardiology, Rotterdam, Netherlands; ^5^University Medical Center Utrecht, Department of Reproductive Medicine & Gynecology, Utrecht, Netherlands

##### **Correspondence:** L. Al-Hassany

Objective: Migraine is an underappreciated cardiovascular (CV) risk factor. We compared endothelial microvascular dysfunction, the contribution of neuropeptide release, and the CV risk in middle-aged women suffering from PCOS with and without migraine.

Methods: Peripheral microvascular function was assessed cross-sectionally using Local Thermal Hyperemia (LTH) of the forearm under control conditions, after inhibition of neuronal axon reflex by EMLA cream application, and inhibition of nitric oxide (NO) formation by L-NMMA. The dermal blood flow (DBF) response to LTH is characterized by a first peak mediated by neuropeptide release, followed by a plateau phase mainly involving NO.

Results: We have included 49 women with PCOS, of which 23 suffered from migraine (mean age 50.8±2.9). Baseline characteristics of both groups were comparable, including their CV risk as traditionally determined by the Framingham Risk Score. EMLA cream application resulted in significantly lower inhibition of the total DBF response in migraine patients – expressed as the Area Under the Curve (AUC) – relative to control conditions (95% CI of the difference [4.04–33.47]; p=0.014). Also, EMLA cream caused a significantly lower inhibition in both the height and the AUC of the plateau phase relative to control conditions in migraine patients.

Conclusions: Neuropeptide action was significantly decreased in migraine patients in the interictal period, which may contribute to the increased CV risk in migraine.

## P0391 The hidden diagnoses in Emergency Department: a study on not otherwise specified headache

### L. D'Acunto, A. Granato, M. E. Morelli, G. Bellavita, P. Manganotti

#### University Hospital and Health Services of Trieste - ASUGI, University of Trieste, Clinical Unit of Neurology, Headache Centre, Department of Medicine, Surgery and Health Sciences, Trieste, Italy

##### **Correspondence:** L. D'Acunto

Objective**:** Aim of the study was to evaluate specialist visits carried out in the patients discharged from emergency department (ED) with diagnosis of Not Otherwise Specified (NOS) headache in order to evidence discrepancies between specialist and ED diagnosis at discharge.

Methods: All patients discharge from the ED of the tertiary-care University Hospital of Trieste with NOS headache as final diagnosis were retrospectively (1.6.2018- 31.12.2018) analyzed. We included who underwent to one specialist examination at least. Demographic data, specialist and ED diagnosis were analyzed.

Results: We analyzed 124 patients (93 F, 31 M, 44 y ±15). 71.8% of patients were examined only by a neurologist, 12.9% by non-neurologists, 15.3% by both neurologist and non-neurologist. Only 37% received a precise diagnosis, slightly more frequently by neurologist than the other consultants (40.5% vs 37.5%). Neurologists diagnosed primary headaches, headaches secondary to neurological diseases, and facial neuralgia; non-neurologists detected headaches secondary to non-neurological diseases. Primary headaches were diagnosed in 25.7% of cases, migraine being the most frequent. Physicians did not report any specialist diagnoses in the ED discharge sheet.

Conclusions: Specialist consultants made specific diagnoses in one-third of patients that were not reported as final in the discharge records by the ED physician. This leads to a loss of diagnoses and to an overestimation of NOS headache.

## P0392 Changes in pain syndrome and quality of life in patients with pituitary adenoma after surgical treatment

### M. Kurnukhina, V. Cherebillo

#### First Pavlov State Medical University of St. Petersburg, Neurosurgery, St. Petersburg, Russian Federation

##### **Correspondence:** M. Kurnukhina

Background: Headache is one of the most frequent complaints of patients with pituitary adenoma. In this regard,the study on the impact of pain on the quality of life in these patients is extremely relevant at the present time.Previously, studies on this topic have not been described in the literature.

Purpose: Assessment of the impact of pain syndrome on the quality of life of patients with pituitary adenoma in the pre-and postoperative period.

Materials and methods**:** A clinical study of 45 patients with pituitary adenomas was conducted.The analysis of the quality of life was carried out in the preoperative period and in the early postoperative period.The subjects were aged from 22 to 63 years(median 45 years).We used a special EORTC QLQ-C 30 questionnaire.

Results**:** 68,9% of patients reported diffuse headaches, 4.4%-headache of a certain localization. Before surgery patients with more pronounced pain syndrome more often indicated a deterioration in cognitive,social functioning,increased fatigue, decreased appetite and general health (p<0,05).After surgical treatment, the severity of headaches decreased in patients (before surgery-28.51±29,41; after surgery-16.31±25,02). After the removal of the formation, the patients, as well as before the operation, noted increased fatigue, decreased appetite and physical functioning with severe headaches (p<0,05).

Conclusion: We found a decrease in the severity of the pain syndrome, against which there was an improvement in the quality of life

## P0393 Relationship between sleep disorders and life-style in patients with chronic tension-type headache

### C. Rusamova, M. Yakubova, N. Ibrohimova, D. Po'latova

#### Tashkent Medical Academy, Neurology, Tashkent, Uzbekistan

##### **Correspondence:** C. Rusamova

Objectives**:** to assess the prevalence and impact of sleep disturbances and its associations with life-style including work, social functioning and well-being in tension-type headache(TTH)patients.

Materials and methods: We examined 80 patients (men-36, (45%) women - 44, (55%) aged from 40 to 78 years, in TTH patients at the Tashkent Medical Academy in neurology department.The Pittsburgh questionnaire was used for determining the quality of sleep index (PSQI).Headache related life-style was measured using the Disability Days/Impairment Ratings, The Hassles Scale Short Form.

Results: On the Pittsburgh scale, various sleep disorders were observed in 70 patients (88%)100% of cases from 1 to 5 times a week) in relation to the duration of sleep, daytime dysfunction, and subjective sleep quality (p<0,005). Poor sleep quality (higher PSQI components (19-29 points) were observed in patients with TTH.Headache-related disability days were reported by 84% of patients (p<0.0001), work or social functioning was severely impaired in 58% of patients (p<0.005).

Conclusion: The presence of association of headache and sleep disorders was found and it impacts negatively on patient"s life-style. A close attention to sleep problems among patients with headache may have implication for the choice of treatment and the prognosis, possibly preventing chronification of the headache and worse quality of life.

## P0394 **Comorbidities of patients with dementia and migraine**

### M. Dyess^1^, N. Murinova^1^, D. Krashin^2^

#### ^1^University of Washington Medical Center, Neurology, Seattle, WA, United States; ^2^Puget Sound VA, Seattle, WA, United States

##### **Correspondence:** M. Dyess

Objective: Describe characteristics of patients diagnosed with dementia and migraine at University of Washington Medical System.

Design/Method: The Leaf research database was used to obtain retrospective data for all patients with ICD-10 code diagnosis for migraine (using ICD-10 G43.001-G43.D1) and dementia (using ICD-10 F03.90-F03.91) at the University of Washington.

Results: Migraine was identified in n=33,549 patients, and dementia was identified in n=8,668 patients. N=149 patients had both migraine and dementia. 46% (n=69) of those with dementia and migraine also had anxiety (ICD10: F41.0-41.9) versus 20% (n=1740) of those with dementia alone. 36% (n=53) of patients with dementia and migraine were diagnosed with insomnia (ICD10: G47.00-G47.09) versus 14% (n=1242) in dementia alone. Hypertension (ICD10: I10) was noted in 72% (n=107) of those with dementia and migraine. Hypertension was present in 59% (n=5114) of those with dementia alone and 21% (n=7132) in migraine alone.

Conclusion**:** Migraine is an infrequent concurrent diagnosis in those with dementia and represents 1.7% of patients in this cohort. Rates of anxiety, insomnia, and hypertension were higher in those with migraine and dementia over those with dementia alone. Clinical experience shows that we rarely see migraine concurrently diagnosed in dementia patients, and this is difficult to explain with current scientific understanding.

## P0395 New master's degree program "Master of Migraine and Headache Medicine" at the University of Kiel

### H. Göbel^1^, M. Illert^2^

#### ^1^Kiel Headache and Pain Centre, Kiel, Germany; ^2^University Hospital Schleswig-Holstein, Center for Scientific Continuing Education at the Christian Albrecht University of Kiel, Kiel, Germany

##### **Correspondence:** H. Göbel

At the University of Kiel, Germany, a new academic master's course "Master of Migraine and Headache Medicine" (MMHM) of four academic semesters is planned to start in the winter term of 2021/2022. The concept was positively assessed by a national and international expert board.

The Master's degree is divided into four terms, with the first three terms being allocated to teaching sessions, the fourth term being allocated to the master's thesis.

In the first term (Foundation, Organization, Clinical pathways), the foundation of headache disorders and the clinical processes in a hospital specialized in the treatment of headache disorders are presented. The focus hereby lies on epidemiology, classification, pathophysiology of headache disorders, medical ethics and organization / leading of teams. Three days are reserved for clinical practice.

The second term (Diagnostic pathways and therapies) focuses on the methods and criteria for diagnosing different headache disorders. At the forefront lie interdisciplinary approaches using various evidence-based methods and clinical practice.

In the focus of the third term (Organization/Structure of headache treatment and prevention, perspectives for the coming decade) lies the concrete interdisciplinary teamwork in headache centres, which is presented as part of hospital days.

Teaching will consist of a mixture of face-to-face teaching as well as clinical on-site training.

## P0396 Why patients do not comply with headache diaries?

### C. Borbinha^1^, I. Pavão Martins^2^

#### ^1^Hospital do Espírito Santo de Évora, Neurology Department, Évora, Portugal; ^2^Hospital Universitário de Sta Maria, CHLN, Lisboa, Faculdade de Medicina da Universidade de Lisboa, Headache Unit, Neurology, Lisbon, Portugal

##### **Correspondence:** C. Borbinha

Background: Headache calendars are part of good clinical practice in headache clinics. However, patients" compliance is rather variable. We aim to identify factors associated with poor compliance.

Methods: Consecutive patients observed in follow-up visits of a tertiary headache center were divided into two groups; with a fullfilled calendar (Calendar compliers, CC) and without calendar (Calendar noncompliers, CNC). Incomplete /forgotten records were excluded. Demographic and clinical variables were compared, and CNC were asked the reasons for not filling the calendar.

Results: From 93 patients (45.6±13.3 years, on average; 83 females), the majority with migraine (96.8%), 61.3% were CC. CNC were more likely to have medication overuse (34.5% vs. 12.3%, p=0.01) and had a tendency to be paid workers (79.3% vs. 52.6%, p=0.05) compared to CC.Most CC considered calendars useful to improve doctors and patients knowledge about headaches.

Conclusions: Although these results need to be evaluated in other contexts, they suggest that patients with medication overuse have a more denial attitute towards headache records and may need additional reinforcement.

## P0397 Migraine screening in oncohematologic patients with ID Migraine questionnaire

### I. Skiba^1^, A. Polushin^1^, M. Ratanov^2^, M. Vladovskaya^1^, A. Kulagin^1^

#### ^1^First Pavlov State Medical University of St. Peterburg, RM Gorbacheva Research Institute of Pediatric Oncology, Hematology and Transplantation, St. Petersburg, Russian Federation; ^2^Sechenov University, Moscow, Russian Federation

##### **Correspondence:** I. Skiba; M. Ratanov

Background and Objective: To assess ID Migraine questionnaire sensitivity and specificity in oncohe-matologic patients.

Methods: We conducted a retrospective observational study in RM Gorbacheva Research Institute, Pavlov University. Adult oncohematologic patients who were referred to a neurologist in 2020 were included. Patients assessed by a neurologist in the early post-transplant period (100 days following hematopoietic stem cell transplant) or the intensive care unit were excluded. We recruited 268 patients with acute leukemia (68.6%), lymphoma (15.0%), chronic myeloproliferative disorders (8.2%), multiple myeloma (4.8%), or aplastic anemia (3.4%). Patients eligibility for ID Migraine was assessed with pretest criteria. After ID Migraine test, patients were assessed by a neurologist. Headache disorders were diagnosed with ICHD-3.

Results**:** Headache was diagnosed in 58 patients (21.6%). Migraine without aura (11.6%) and Infrequent episodic tension-type headache (4.9%) were the most prevalent primary forms of headache. Post-dural puncture headache (5.9%) was the most common secondary headache. Fourteen patients had two or more types of headache (24.1% of all headache patients). Thirty-nine patients (14.5%) met the pretest criteria of ID Migraine. ID migraine sensitivity and specificity was 91.3% (95% CI, 72.0% to 98.9%) and 62.5% (95% CI, 35.4% to 84.8%), respectively.

Conclusion: In oncohematologic patients, ID migraine showed high sensitivity and low specificity.

## P0399 Clinical symptoms of androgen deficiency in men with migraine or cluster headache: a cross-sectional study

### I. Verhagen^1^, R. Brandt^1^, C. Kruitbosch^1^, A. Maassen van den Brink^2^, R. Fronczek^1^, G. M. Terwindt^1^

#### ^1^Leiden University Medical Center, Leiden, Netherlands; ^2^Erasmus University Medical Center, Rotterdam, Netherlands

##### **Correspondence:** I. Verhagen

Objective**:** To compare symptoms of clinical androgen deficiency between men with migraine, men with cluster headache and non-headache male controls.

Methods**:** We performed a cross-sectional study using two validated questionnaires to assess symptoms of androgen deficiency. Primary outcome was the mean difference in androgen deficiency scores. Generalized linear models were used adjusting for age, BMI, smoking and lifetime depression. As secondary outcome we assessed the percentage of patients reporting to score below average on sexual symptoms (beard growth, morning erections, libido and sexual potency) as these items were previously shown to differentiate androgen deficiency symptoms from anxiety and depression.

Results**:** The questionnaire was completed by n=534 men with migraine, n=437 with cluster headache and n=152 controls. Patients reported more severe symptoms of androgen deficiency compared with controls, with higher AMS scores (Aging Males Symptoms; mean difference±SE: migraine 5.44±0.90, p<0.001; CH 5.62±0.99, p<0.001) and lower qADAM scores (quantitative Androgen Deficiency in the Aging Male; migraine: -3.16±0.50, p<0.001; CH: -5.25±0.56, p<0.001). Additionally, both patient groups more often reported to suffer from any of the specific sexual symptoms compared to controls (18.4%, 20.6%, 7.2%, p=0.001).

Conclusion**:** Men with migraine and cluster headache more often suffer from symptoms consistent with clinical androgen deficiency than non-headache male controls.

**Fig. 1 (abstract P0399).**
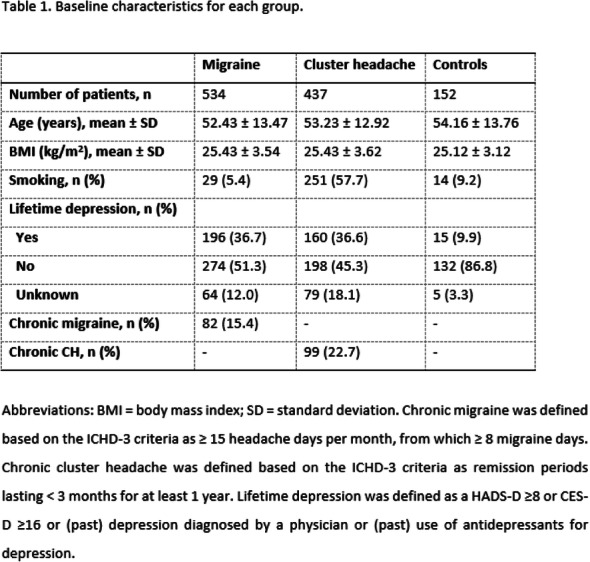
See text for description

**Fig. 2 (abstract P0399).**
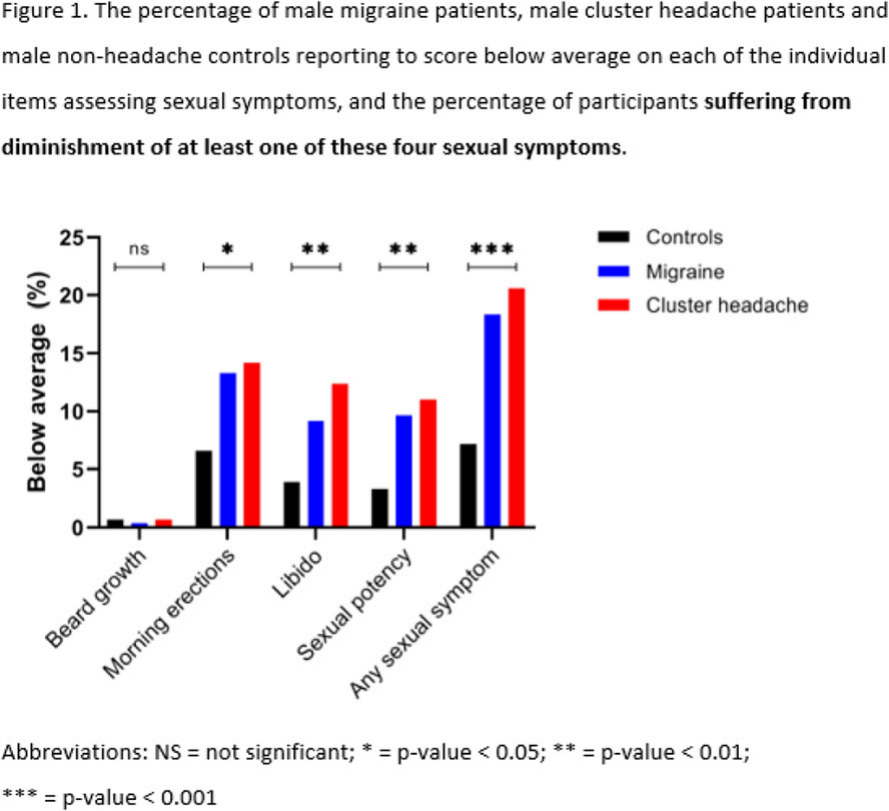
See text for description

## P0400 Pediatric tension type headache (TTH): comparative study of the preventive therapy methods effectiveness

### N. Zavadenko, Y. Nesterovskiy, E. Elena Shipilova

#### Pirogov Russian National Research Medical University, Moscow, Russian Federation

##### **Correspondence:** N. Zavadenko

Objective: to evaluate the effectiveness of monotherapy with aminophenylbutyric acid hydrochloride (APBAH), amitriptyline (A) or breathing gymnastics (BG) indicated for 2 months as preventive treatment of TTH in children and adolescents in the open randomized comparative study. 90 patients aged 8-16 years were divided into 3 groups of 30 patients each.

Methods: HIT-6 (Kosinsky et al, 2003), PedMIDAS (Hershey et al, 2001), MFI-20 (Smets et al, 1995], SCAS (Spence et al, 2003), SDSC (Bruni O et al, 1996). APBAH dosage was 15-20 mg/kg daily, A – 20-30 mg daily per os.

According to the criterion of a 50% or more reduction in the average number of headache attacks per month the improvement was achieved in group 1 (APBAH) in 56.7% of patients, group 2 (A) – in 73.3%, the group 3 (BG) – in 30%. According to a more strict criterion for headache attacks reduction by 75% or more per month, the response was observed in group 1 in 30%, group 2 – 23.3%, group 3 – 3.3% of patients. Significant differences with the group 3 for response to therapy were confirmed for groups 1 and 2. In groups 1 and 2, along with a significant decrease in the frequency, duration and intensity of TTH attacks, the significant improvement in daily activity with favorable effects on the fatigue, anxiety and sleep disorders manifestations was demonstrated. The preventive efficacy of BG was confirmed with the reduction of TTH frequency, duration, intensity and diminishing their negative impact on daily activity.

## P0401 The role of NO system in tension-type headache and arterial hypertension phenotype development

### P. Moskaleva^1^, N. Shnayder^1,2^, M. Petrova^1^, R. Nasyrova^2^

#### ^1^V. F. Voino-Yasenetsky Krasnoyarsk State Medical University, Krasnoyarsk, Russian Federation; ^2^V.M. Bekhterev National Research Medical Center for Psychiatry and Neurology, St. Petersburg, Russian Federation

##### **Correspondence:** P. Moskaleva

Background: Patients with tension-type headache (TTH) have an increased risk of developing arterial hypertension (AH), while hypertensive subjects do seem to have an increased risk of TTH.

The aim is to study the role of SNVs of the NOS1, NOS2, and NOS3 genes in the comorbidity of AH and TTH.

Methods**:** We searched for full-text English publications in databases over the past 15 years. In addition, earlier publications of historical interest were included in the review.

Results**:** In our review, we summed up the single nucleotide variants (SNVs) of Nitric Oxide Synthases (NOSs) genes involved in the development of essential AH and TTH. The results of studies we discussed in this review are contradictory. This might be due to different designs of the studies, small sample sizes in some of them, as well as different social and geographical characteristics.

Conclusions**:** The contribution of genetic and environmental factors remains understudied. This makes the issue interesting for researchers, as understanding these mechanisms can contribute to a search for new approaches to pathogenetic and disease-modifying treatment of the AH and TTH phenotype. New drugs against AH and TTH can be based on inhibition of nitric oxide (NO) production, blockade of steps in the NO-cGMP pathway, or NO scavenging. Indeed, selective neuronal NOS (n-NOS) and inducible NOS (i-NOS) inhibitors are already in early clinical development.

References: https://doi.org/10.3390/molecules26061556

## P0402 Is there any impact of cardiovascular risk factors and migraine comorbidity on Parkinson's disease severity? Preliminary data of the Moldovan Parkinson's disease cohort

### L. Rotru

#### Institute of Neurology and Neurosurgery, Functional Neurology, Chisinau, Moldova

Objectives: establishing impact of vascular risk factors, particularly migraine on severity of Parkinson's disease.

Methods: Analysis included 29 consecutive PD patients from Moldovan cohort (20.80009.8007.39), mean age 63.3±8.1 yo, mean disease duration 48.9±12.9 mo., mean disease onset age of 59.0±8.7 y. Motor assessment (UPDRS II scale, Tremor Score, Akinetic-rigid Score), quality of life impairment (PDQ39), cognitive performance (MoCA) were conducted. Presence of vascular risk factors (FRV), including migraine (Mg), QRISK3 scores and relative cardiovascular risk were determined.

Results: Twenty-five (86.2%) of total PD patients had VRF. Migraine present in 41% of them.

PD+VRF was associated with higher scores of disease severity: UPDRS II 46.73±11.75 (vs. 42.75±23.71), TrScore 0.94±0.56 (vs. 0.88±0.33), ARScore 0.82±0.53 (vs. 0.70±0.12), higher PDQ39 52.78±27, 81 (vs. 41.25±20.16) and lower cognitive MoCA scores 21.75±4.07 (vs. 22.60±3.29), not reaching statistical significance.

PD+Mg had higher PDQ39 index: 55.00±31.12 (vs. 48.88±24.35) and lower MoCA cognitive scores - 21.48±4.20 (vs. 22.09±3.90), no statistically significant difference. PD+Mg patients had significantly higher relative cardiovascular risk scores - 2.80±6.84 (vs. 1.53±0.66, p = 0.02).

Conclusions: Vascular risk factors, if present in PD patients, may predict a worse motor and cognitive performance. Migraine presence significantly increases the patients cardiovascular risk.

## P0403 Quality analysis of primary headaches diagnosis and treatment: online survey results from Russian Federation

### D. Khutorov^1^, D. Korobkova^2^, T. Makeeva^3^, N. Vashchenko^2,4^, K. Skorobogatykh^2^

#### ^1^Nikiforov Russian Center of Emergency and Radiation Medicine, Clinical Neurology Department, St. Petersburg, Russian Federation; ^2^University Headache Clinic, Moscow, Russian Federation; ^3^"Klinika "Gorod Zdorovia" Clinic, Voronezh, Russian Federation; ^4^Sechenov University, Moscow, Russian Federation

##### **Correspondence:** T. Makeeva

Background and objectives: Despite the availability of the International Classification of Headache Disorders and clinical guidelines, patients with primary headaches still face misdiagnosis, prescription of non-informative investigations and ineffective therapy. We evaluated the quality of medical care for patients with primary headaches in Russian Federation.

Methods: We created an online questionnaire for people with a diagnosed primary headache. The questionnaire consisted of 17 questions regarding diagnosis and treatment choices and patient satisfaction with the treatment results.

Results: The study included 234 participants (227 women), mean age 36.5 ± 9.2. Among them, 174 patients had a migraine, 57 tension-type headache and 3 cluster headache. Only 16% of patients received their diagnosis at the first doctor visit. In 80% of cases, doctors prescribed additional instrumental investigations (fig. 1). The mean diagnostic delay was 4.7 ± 6.5 years. Fifty-six percent of the interviewees were prescribed ineffective medications for their headaches (fig. 2). Forty-seven percent were dissatisfied with the treatment results and 58.5% wanted to find another specialist.

Conclusions: Our findings emphasize the still existing challenges of diagnosing and treating primary headaches, as well as the need for improved specialized education for physicians in this area.

**Fig. 1 (abstract P0403).**
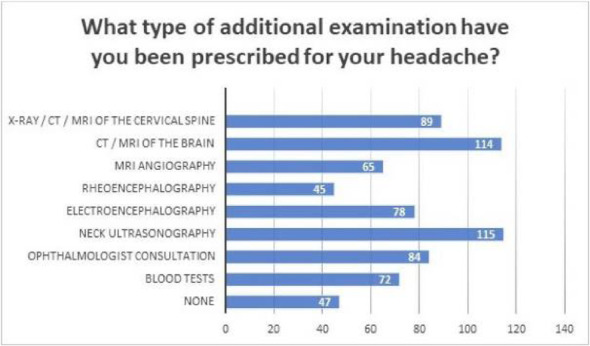
See text for description

**Fig. 2 (abstract P0403).**
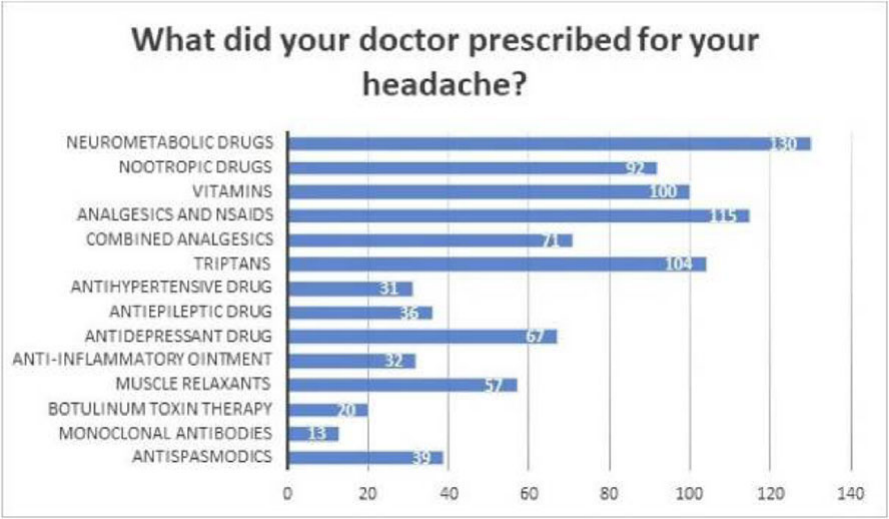
See text for description

## P0404 Impact of the Internet on the awareness and health literacy of headache patients

### D. Khutorov^1^, D. Korobkova^2^, T. Makeeva^3^, N. Vashchenko^2,4^, K. Skorobogatykh^2^

#### ^1^Nikiforov Russian Center of Emergency and Radiation Medicine, Clinical Neurology Department, St. Petersburg, Russian Federation; ^2^University Headache Clinic, Moscow, Russian Federation; ^3^"Klinika "Gorod Zdorovia" Clinic, Voronezh, Russian Federation; ^4^Sechenov University, Moscow, Russian Federation

##### **Correspondence:** T. Makeeva

Background and objectives: Outreach and health literacy are among the main objectives of professional associations and play an essential role for headache patients. With the modern Internet accessibility, web pages of medical societies and doctors in social networks increasingly attract patients and become a source of reliable information. We analyzed how medical information on the Internet affects headache patients.

Methods: We created an online survey for Russian-speaking people with headaches. The questionnaire included 12 questions regarding the availability of medical information on the Internet and its impact on headache awareness.

Results: The study included 307 participants (292 women) aged 36.6 ± 10.2. Information on the Internet helped 38.9% of respondents to ensure that the doctor diagnosed them correctly and be more susceptible to proper treatment. The medical information obtained on the Internet was helpful and valuable for 46% of interviewees. However, 51.1% were unaware of headache specialists, offices, and pain treatment centres.

Conclusions: The survey showed low patient awareness of the availability of specialized headache care and the importance of online headache education.

## P0405 An atypical case of Epicrania fugax with coronal pain radiation

### A. Cordeiro, E. Silva, M. Grunho, L. Pereira

#### Hospital Garcia de Orta, Department of Neurology, Almada, Portugal

##### **Correspondence:** A. Cordeiro

Objective: Epicrania fugax (EF) is a primary headache consisting of brief stabbing head pain, following a linear or zigzag trajectory across the scalp, through the territories of different nerves. Although rarely, some cases of coronal radiation of pain have been described.

Methods: Clinical case.

Results: A 48-year-old woman with a history of migraine without aura presented to the emergency department with new onset headache: stabbing, severe (10/10 on the visual analog scale), describing a linear trajectory on the coronal plane from the right temporal to the left temporal scalp, lasting 1-40 seconds, multiple times a day, preventing sleep. Neurological examination revealed right hemicrania hypoesthesia. Laboratory tests were unremarkable, and brain magnetic resonance imaging exhibited dilated Virchow-Robin perivascular spaces (PVS) in the left hemi-midbrain. Pregabalin 25mg twice a day was started with immediate pain relief and complete resolution by the sixth day.

Conclusion: We believe this to be a case of atypical EF with coronal radiation, raising awareness that patients can present with linear pain of different trajectories across the scalp. Despite the presence of dilated PVS, they are unlike to be causal due to their nature, lateralization and absent relation with the trigeminal nucleus and other relevant pain matrix structures. Early diagnosis of these atypical cases is essential to provide the proper treatment.

**Footnote**

**Abstract authors were asked to disclose their Conflicts of Interest. The disclosures are available**via this link.

**Index**

**A**

Ardayfio, P. A. P0110

Foster, S. A. P097

Abagnale, C. P0102, P0103, P0104

Abbasi, M. AL020

Abdel-Naseer, M. P0169

Abdelsalam, M. P0140

Abdou, A. P0239

Abdrabboh, A. AL050

Abdullahi, R. P074

Abdumanapova, G. P0236

Abreu, Â. P0151, P0324, P0326, P0349

Abreu, P. P0131

Abuukar Abdullahi, R. P074

Acar, E. P0158

Acarsoy, C. AL016

Adams, A. P0258, P0382, P099

Addesi, A. P08

Adoukonou, T. P044

Agathonikou, A. P0384

Agbetou, M. P044

Aguggia, M. AL045, AL057, P0343, P0347

Aguiar, M. P0119

Aguilella, C. P0152

Ahmad, L. P0332

Ahmad, S. Y. P0351

Ahmed, A. P0383

Ahmed, F. AL024

Ahn, A. H. AL022

Ahn, J.-Y. P010, P011

Ailani, J. P0112, P0281, P0295

P0296, P0353

Aja-Fernández, S. AL011, P031, P035

Al-Hassany, L. P0390

Al-Karagholi, M. A.-M. AL025, P076

P079, P080, P084

Al-Khazali, H. AL027

Albanese, M. AL045, AL057, P0318

P0343, P0347

Alberdi, A. P039

Alberto Bordini, C. P0388

Alfatih, M. P0383

Alidoosti, F. P0355

Alimajstorovic, Z. AL035, AL040, P0118

P0119, P0120, P0124

P0125, P061

Allena, M. P0139, P0300, P0311

P0330, P0332, P0373

Almeida, A. P0190

Alpuente, A. AL024, P073

Altamura, C. AL045, AL057, P0318

P0343, P0347

Alves-Ferreira, M. P050

Amand, C. P016

Amin, F. M. AL027, P079, P084

Amorim, M. P0211

Anarte-Lazo, E. P0202, P0203, P0379

Andersen, A. S. S. P0189, P0193, P025

Andersen, M. P0225

Andreou, A. P. AL024, P074

Andrini, G. AL046

Angermaier, A. AL042

Ansons, A. P0119

Aparecida Bello, V. P049

Aposporos, G. P0384

Arab, A. P0303

Aranyi, C. S. AL026

Arca, K. P0259

Arceri, S. P0330, P0332

Arendt-Nielsen, L. P0245, P055

Argiti, K. P0130

Argyropoulos, A. P0107

ARI, C. P02

Ariyanfar, S. P032, P047

Armand, C. IL012

Armand, C. E. P088

Arturo Larco, J. AL020

Ashina, H. AL027

Ashina, M. AL025, AL027, AL049

P0106, P0299, P0301, P0320

P076, P079, P080, P084, P087, P089

Asibong, U. P0238

Asioli, G. M. AL046, P0155

Askari, G. P0303, P0304

Askew, C. AL055

Asskour, L. P073

Attia, Z. AL02

Augimeri, A. P036

Aurilia, C. AL045, AL057, P0231, P0318

P0343, P0347

Aurora, S. P012, P0264

Ayer, D. P0226

Azimova, J. P0150, P0198, P0221, P0341

Azizi, E. P0355

**B**

Barros, A. B. P015

Babaei, M. P0381

Baburao Kulkarni, G. P0386

Badr, M. P0375

Bagdy, G. AL026

Bagnoli, C. P0160

Baiata, C. P0257

Baird, S. P0235

Baksa, D. AL026, P098

Baleki Borri, J. P0388

Balercia, A. P0160

Ballesta-Martínez, S. P0147, P0179, P0309

Ballvé, A. P040

Bangs, M. P095

Baraldi, C. AL024

Barash, S. AL043, AL051, P0108, P0313

P0315, P0320, P0321, P0323

Barbanti, P. AL045, AL057, P0231, P0318

P0343, P0347

Barch, C. IL014

Barloese, M. P014, P0154

Barros, G. P0191

Bartolini, M. P0160

Bauer, R. P066

Baufeld, C. P0308

Baykan, B. AL010, P02

Beck, J. AL039, P0129, P0130, P0132

Becker, B. P019

Beier, D. P0122

Belaskova, S. P0113, P06

Belay, H. P0213

Bellavita, G. P0391

Bello, V. A. P0173

Bellosta-Diago, E. P0147, P0309

Belopasova, A. P092

Belvís, R. P022, P0290, P0291

Benbow, T. P0241, P0243

Bendtsen, L. P0189, P0193, P025

Benseñor, I. AL017, P052, P069

Benzimra, J. P0119

Bergmann, A. P0346

Berisha, V. AL030, P0137

Berman, G. P0271

Bernal-Utrera, C. P0379

Berndl, T. P0159

Bertisch, S. P0166

Bertuzzo, D. AL045, AL057, P0343, P0347

Best, L. P0234

Bevilaqua-Grossi, D. P093

Bigal, M. P0161, P0162, P0224, P0372, P06

Bilton, E. AL035

Bitetto, V. P0300, P0302, P0311

Bizzarri, P. P0160

Bjornsdottir, G. AL04

Blanco, M. P039

Blumenfeld, A. M. P0268, P0285, P0292

P0293, P0294, P0301, P089

Boersma, E. H. P0390

Bolay, H. IL04, P02

Bonamico, L. P024

Bonassi, S. P0231, P0318

Bono, F. AL045, AL057, P0318

P0343, P0347

Borbinha, C. P0396

Bos, D. AL016

Bossa, B. B. P0173

Both, B. P0144

Bottiroli, S. P0139, P0311, P0373

Bovis, F. P0245, P055

Bozhenko, M. P0233, P0365

Bozhenko, N. P0233, P0365

Bragg, S. P0110

Bralic, M. P0369

Branca, M. P0227

Brandes, J. P095

Brandt, R. P0399, P07

Braune, S. P0346

Brevig, T. P0312

Brighina, F. P0170, P0207

Brito, J. AL018

Brock, K. AL035, AL040, P0118

P0119, P0120

Broessner, G. P0325

Brooks, C. P0219

Brown, R. P0121

Bruckers, L. P0357

Brunelli, N. AL024, AL045, AL057

P0343, P0347

Bruno, F. P0142

Bruzzone, M. G. P019, P020

Buckman, R. P0125

Budica, M. P0113

Buffa, D. P0170

Buoite Stella, A. P0178, P033

Buonvicino, D. P062

Burch, R. IL022

Burow, P. P0337

Burrone, L. P0235

Burslem, K. AL060, P0112

Burstein, R. P0382

Buse, D. C. AL041, AL048, AL052

P0165, P0219, P0261, P0327

P0387, P087, P088

Bushnell, D. P0226

Butera, C. AL024

Buture, A. P0306, P0307, P0338

Buzby, M. P0219

Buzzatti, L. P0160

**C**

Cadiou, F. AL060, P0258

Cady, R. AL048, P0106, P0289

P0312, P087

Cairns, B. P0241, P0243

Calabria, S. P08

Calandre, E. P0148, P0149

Calcerrada, I. P039

Calderaro, M. P0222, P0223, P0363, P064

Callesen, I. AL021

Calvillo, A. P0117

Calvo, D. P024

Capizzi, M. P0207

Caponnetto, V. P0142

Caramia, F. P0103

Carlo, P. P08

Carmine Belin, A. P013

Carnevale, A. AL045, P0318, P0347

Caronna, E. P040, P073

Carpay, J. A. P07

Cartwright, D. P0118, P0119, P061

Carvalho, G. F. P0202, P0203

Carvalho, R. P0223

Casanova, A. P0370

Casillo, F. P0102, P0104

Castaldo, M. P0245, P055

Castellazzi, G. P0252, P0330, P0332

Castelli, L. P0373

Cebola, P. P0190

Cecconi, E. P091

Célia Poli Frederico, R. P049

Cervellino, A. P038

Cetta, I. AL028, P0329

Cevoli, S. AL024, AL045, AL046

AL057, P0155, P0343, P0347, P08

Chalkiadaki, E. P0360

Chalmer, M. A. AL021, AL03, P0204

Chambers, D. P074

Chan, W.-K. AL06

Chan-Goh, M. P0113, P0161, P0162

P0224, P0372, P06

Chao, C.-J. AL02

Charleston IV, L. P0312

Chaves, L. P0222

Chavez, B. P0214

Cheema, S. AL034

Chehrenama, M. P0165

Chen, S.-P. AL013, AL015, P0134

P0200, P028, P051, P083

Chen, S.-T. P03

Chen, S. P0362

Chen, W.-H. P0143

Chen, W.-T. AL013, P0200, P081, P083

Cheng, F. P0298

Cheng, S. AL049

Cherebillo, V. P0128, P0194, P0392

Chernenko, A. P021

Chhabra, N. AL02

Chiang, C.-C. AL02, AL020, AL08

P0121, P0259

Chiapparini, L. P019, P020

Chiarugi, A. P062

Cho, S.-J. P010, P011, P0141, P0157

Cho, S. AL029, P010, P0305, P04

Choi, Y.-J. P011

Chong, C. AL030, IL06, P0137

Chong, Y. C. P0127

Chou, D. E. AL049

Chou, K.-H. P083

Chowdhury, A. P0111

Chowdhury, B. AL05

Chowdhury, D. AL05, P029

Christenen, S. L. T. P057

Christensen, C. AL027

Christie, S. P0226

Chu, M. K. P010, P011, P0141, P043

Chung, C.-S. AL029, P010, P011, P0305, P04

Chung, J. M. P010, P011

Chung, P.-W. P010, P011

Cipriani, D. AL039, P0130

Cirkel, A. AL012, P0114, P0188, P034, P041

Clasen, S. P0100

Cnossen, V. P07

Cohen, A. P048

Cohen, J. P0192

Cohen, J. M. AL043, AL051, AL052

P0108, P0229, P0230, P0313

P0314, P0315, P0316, P0320

P0321, P0323, P0327, P0335

Colombo, B. AL024, AL028, AL045

P0318, P0329, P0343, P0347

Cominotto, F. P033

Conaty, K. P0306, P0338

Constantin, L. P016

Contreras-De Lama, J. P0258

Conway, C. M. AL054, P0266, P0267

P0272, P0275, P0278

Coppola, G. P0102, P0103, P0104

Cordeiro, A. P0405

Coric, V. AL014, AL053, AL054, P0266

P0267, P0268, P0269, P0271, P0272

P0275, P0278, P0282, P0339, P0340

Cornejo, A. P039, P040

Corral-Quereda, C. P0297, P0331, P0334

Cortelli, P. AL046, P08

Cortez, A. P0335

Cortez, M. M. P0145

Cosentino, G. P0330, P0332

Coskun, H. P076, P079

Costa, A. P0131

Costa, B. P0222

Costa, C. M. AL045, AL057, P0318

P0343, P0347

Courraud, J. P048

Cristobal, M. P0368

Croop, R. AL014, AL053, AL054

P0266, P0267, P0268, P0269

P0271, P0272, P0275, P0278

Cseh, E. K. P0247

Cubero, M. P039

Cuneo, A. P0113, P0162, P0224, P0372, P06

Cusó, S. P0152

**D**

D'Acunto, L. P0178, P033, P0391

D'Aiuto, F. P0170

D'Amico, D. AL045, P0347

D'Aurizio, G. P0142

D'Onofrio, F. AL045, AL057, P0318

P0343, P0347

Dabruzzo, B. AL041, P0261, P0281

P0294, P0295

Dach, F. P093

Dahlem, M. P0337

Dahlgren, A. P013

Dalkara, T. AL037

Damasceno, C. P0363

Damush, T. P0235

Dankaerts, W. P0357

Danno, D. P01

Danser, A. H. J. AL023, P0390, P060

Daripa, B. P0253

Datta, D. P029

Davidsson, O. P0225

de Boer, I. AL047, P072

de Coo, I. P07

De Dios, A. P0290, P0291

De Icco, R. P0300, P0302, P0311

P0330, P0332, P079, P084, P091

De la O, J. P0214

de Lorenzo, I. P0283, P040, P085

de Luis-García, R. AL011, P0177

P031, P0333, P035

De Martino, A. P036

de Pascual, M. A. P0148, P0149

de Sousa Oliveira, A. P. P064

de Vries, T. AL023, P060

DeCongelio, M. P0232

Dedeken, P. P05

Deicco, R. P0139

Delcourt, C. P0354

Dell Agnello, G. P0387

Demartini, C. P0252, P056

Demichelis, G. P019, P020

Demonte, G. P036

Denton, A. P0374

Desai, J. P0310

Di Antonio, S. P0245, P055

Di Clemente, L. AL045, P0318, P0347

Di Fiore, P. AL045, AL057, P0318

P0343, P0347

Di Giacomo, M. P038

Di Piero, V. P0103

Di Renzo, A. P0103

Dias, A. P050

Díaz de Terán Velasco, J. P0260, P0297

P0331, P040, P063

Díaz Insa, S. P0105, P0336

Diaz-de-Terán, J. P0283, P0334, P085

Diener, H.-C. P0108, P0286

Díez-Tejedor, E. P085

Digre, K. B. P0145

Dikow, H. P0346

Dixit, A. P0386

Do, L. P095

Do, T. P0240, P0342

Dobos, D. AL026, P098

Dobrocky, T. AL039, P0132

Dobrynina, L. P092

Dodick, D. W. AL049, AL08, P0259

P0286, P0292, P0293, P0294

P0382, P087

Doesborg, P. P07

Dondi, L. P08

Dong, Y. P0265

Donmez-Demir, B. AL037

Doretti, A. AL057, P0343

Dorsett, A. P0172

dos Santos Gajardoni Farges, S. H. P049

Doty, E. P0110, P0232

Dougherty, C. P0232

Dovoedo, N. P044

Dowsett, S. P0110, P0387

Drake, M. P0152

Drangova, H. P0227

Driessen, M. P0314

Dubenko, O. P021

Dubey, P. AL05

Dubowchik, G. P0267

Duggal, A. P029

Dumkrieger, G. AL030, P0137

Dunn, R. P0259

Dusselier, M. P0229, P0230

Dyess, M. P0113, P0161, P0224, P0372

P0394, P06

Dzyak, L. P0195, P0237, P0367

**E**

Ebadi, S. P0181

Edina, S. P098

Eftekhari, S. AL031, AL032, P0246

Egeo, G. AL045, AL057, P0231, P0318

P0343, P0347

Ehrlich, M. AL058

Eitner, A. P066

Ekizoglu, E. AL010, P02

El-Rahal, A. P0130

El-Shanshory, M. P0375

Elbahi, F. A. P076, P079

Elena Shipilova, E. P0400

Elia, M. P0207

ELSherif, M. P0140

Emri, M. AL026

Engstrom, E. P0258

Ephross, S. P095

Eren-Kocak, E. AL037

Ernst, M. P048

Ertaş, M. AL010, P02

Esmael, A. P0140

Espinosa-Rueda, J. P0147, P0179, P0309

Esposito, I. P08

Estave, P. P0172, P096

Ettrup, A. P0106, P0289, P087

Evers, S. P092

Evert, L. P046

Exadaktylos, A. P0227

Exposto, F. G. P0241, P0243

**F**

Fabrich Marín, M. I. P0105, P0336

Fabritius, M. P0135

Fahmy, I. P0352

Falla, D. P0202, P0203, P0379

Fallacara, A. AL045, P0347

Fann, C. S.-J. P028

Fanning, K. M. AL041, P0261, P088

Farouk, A. P0352

Farris, S. R. P0172

Fauser, B. C. J. M. P0390

Favoni, V. AL045, AL046, AL057, P0155

P0343, P0347, P08

Fenton, B. P0235

Ferilli, M. A. N. AL07, P0208, P0209

P0210, P0361, P0385, P082, P09

Fernandez, L. P0214, P0368

Fernández Bermejo, E. M. P063

Fernández Recio, M. P0152

Ferrante, E. P038

Ferrari, M. P07

Ferraris, M. AL050

Ferraro, S. P019, P020

Fiammingo, G. P0139

Fiatova, E. AL024

Filippi, M. AL024, AL028, AL045, P0318

P0329, P0343, P0347

Filips, E. P0351

Finnegan, M. P0294, P0295, P0296

P0301, P0382, P089

Finocchi, C. AL045, AL057, P0245

P0343, P0347, P055

Fiorelli, M. P0103

Fiorentini, G. P0231

Fischer, U. P0227

Fischer-Schulte, L. IL020

Fleischmann, R. AL042

Flemming, K. P0121

Fofi, L. AL045, AL057, P0231, P0318

P0343, P0347

Fong, A. AL035

Fonseca, A. P. P0223

Forbes, R. P0234

Ford, J. P0218, P0226

Forshaw, M. P0267

Fortini, I. P0222

Fortunato, F. P036

Fourier, C. P013

Francavilla, M. P0252, P056

Franchito, B. P0191

Frederico, R. C. P. P0173

Frediani, F. AL045, AL057, P0318

P0343, P0347

Frew, E. P0118, P0119

Friedman, P. AL02

Frishberg, B. M. P0285

Fronczek, R. P0399, P07

Frunza, D. P0101

Fuh, J.-L. P0200, P028, P051

Fulton, D. P061

Fung, C. AL039, P0129, P0130, P0132

Furlanis, G. P0178

**G**

Gadzhieva, Z. P027

Gago Veiga, A. B. P0175, P0177, P0333

P040, P063

Galati, C. P0170

Galic, M. P0108

Gallardo, V. J. P040, P073

Galli, F. P0373

Gallo, D. P0364

Gallo, D. P0257

Gambardella, A. P036

Gandhi, P. P0292, P0293, P0299

Gandhi, S. P0335

Gantenbein, A. IL015

Garate, G. P030

Garcia, A. P0368

García, C. P039

Garcia, G. P022

García-Azorín, D. AL011, P0175

P0177, P031, P0333, P035

P039, P040, P063

Garcia-Bermudez, R. J. P0359

García-Gómez, A. P0345

Garcia-Leiva, J. P0148, P0149

Garelja, M. P094

Gaspar Vuolo, I. P049

Gaul, C. P0144, P0308, P0325

Gecse, K. AL026, P098

Geens, M. P0229, P0230

Gelfand, A. IL02

Gendolla, A. P0328

Genizi, J. P0201, P0216

Gezegen, H. AL010

Ghabeli Juibary, A. P0187

Ghanizada, H. AL025, AL033, P076

P078, P080, P086

Ghavami, A. P0304

Ghiggia, A. P0373

Ghiotto, N. P0139, P0302, P0311

P0330, P091

Ghorbani, Z. P047

Gianeri, R. P019

Giani, L. P019, P020

Gil, A. P039

Gil, Y.-E. P0109, P0305, P04

Gil-Gouveia, R. P015, P042

Gil-Martínez, A. P085

Gilev, D. V. P0133

Gill, K. P0165

Gimeno-Ferrer, F. P066

Giorgadze, G. AL048

Girardo, M. P0259

Gklinos, P. P0384

Glassberg, M. AL050

Gliubizzi, C. P0170

Gloor, D. P0227

Goadsby, P. J. AL049, AL053, AL054

P016, P0264, P0268, P0296

P0323; P0382, P087

Göbel, C. AL012, P0100, P0114, P0156

P0188, P0206, P034, P041

Göbel, H. AL012, P0100, P0114, P0156

P0188, P0206, P0317, P034, P0395

Goh, K.-J. P0220

Goicochea, M. T. P024

Goldman, R. P0235

Gomez Valderas, E. P0387

Gonçalo Pinheiro, A. R. P0151

Gongora, F. P0368

González García, N. P040, P063

González Martínez, A. P063

Gonzalez-Martinez, A. P0175, P0177

P0333, P039, P040

González-Quintanilla, V. P0152, P023

P030, P0364

Goodreau, M. P0165

Gorbacheva, N. P046, P065

Görg, S. AL012, P0114, P0188, P034, P041

Gosnell, I. P0235

Goßrau, G. P0176, P0182

Goulart, A. AL017, P052, P069

Grabova, S. P0358

Gralla, J. P0132

Granato, A. P0178, P033, P0391

Grandinetti, M. P024

Granitzer, M. P0357

Graves, A. P095

Grazzi, L. AL024, AL045, AL07, P0172, P0347

Grech, O. AL040, P0119, P0120, P0124

P0125, P061

Greco, R. P0252, P056

Grimaldi Capitello, T. P0208, P0209

Grinberg, A. P0235

Grunho, M. P0405

Gryc, W. P0107

Gryglas-Dworak, A. AL024

Guapacha-Borrero, L. P0148, P0149

Guariglia, C. P0363

Guarnera, A. P082

Guaschino, E. P0139, P0300, P0302, P0311

P0330, P0373, P091

Gubanova, M. P092

Guccione, F. P0170, P0207

Guerrero Peral, Á. L. AL011, P0175

P0177, P031, P0333, P035

P039, P040, P063

Guerzoni, S. AL024

Gugushvili, M. P0251

Guidetti, V. AL07

Gulhote, D. P0191

Gunn, H. P0374

Guo, H. P0295, P0296

Gurary, N. M. P0133

Gusev, A. P0194

Gutierrez, T. P024

Gyöngyi, K. P098

**H**

Ford, J. H.P097

Hagan, K. P0166

Hagen, S. AL032

Haghdoost, F. P0354

Haghighi, S. P032

Hähner, A. P0182

Hakkinen, I. AL01

Haley, H. P0113

Halker Singh, R. AL08, P0299

Halwani, R. AL012, P0114, P0188

P034, P041

Hamann, S. AL032

Hamann, X. P0325

Hamdy, E. P0239

Hamdy, M. P0239

Hamdy, S. P0169

Hamid, Q. AL012, P0114, P0188, P034, P041

Hanafy, A. P0352

Häni, L. AL039, P0130, P0132

Hanna, M. P0266

Hannibal, J. P079

Hansen, J. M. AL025

Hansen, T. P0225

Hansen, T. F. AL021, AL03, AL04, P0204, P048

Harder, A. V. E. AL04

Harris, L. AL014, P0282, P0339, P0340

Hashemi, S. M. P0171

Hassan, A. P0169

Hauck, P. P0232, P0265, P095

Haupt, B. P0130

Hautakangas, H. AL04

Hay, D. AL036, P058, P094

He, L. P0378

Heidari, Z. P0304

Heinskou, T. B. P0189, P0193, P025

Heinze, A. AL012, P0100, P0114, P0156

P0188, P034, P041

Heising, S. P061

Held, I. P0144

Hemmings, K. P0374

Hentschke, C. AL058

Hernandez-Dominguez, J. A. P0359

Hershey, A. IL03, P0370

Hessmann Gonzalez, L. P049

Hickman, S. P0118, P0119

Hidayati, H. B. P0153

Hindiyeh, N. P012, P0353

Hirman, J. AL048, P0106, P0289, P0312

Hitier, S. P016

Hjalgrim, H. P0225

Hoegedal, L. P0122

Hoekman, J. P0264

Hofacker, M. D. P0328

Höfer, B. P0182

Hollander, L. P0231

Holley, M. P0172

Holm, P. P0154

Hougaard, A. P080

Houle, T. T. P096

Hours-Zesiger, P. P0319

Houts, C. R. P087

Hseu, S.-S. P0134

Hsiao, F.-J. P081

Hsiao, Y.-T. AL06

Hsu, C.-L. P028

Hu, K. S. P0145

Hu, Q. P0378

Hubbe, U. P0129

Hübler, A. P0182

Hughes, O. P0285, P099

Hurtado, L. P039

Hutchinson, S. AL041, P0266, P0278

P0281, P0301, P089

**I**

Iaconangelo, C. P0262, P0263

Ibrahim, M. P0383

Ibrohimova, N. P0393

Ieridou, E. P0384

Igarashi, H. P0265

Ikram, M. K. AL016

Ilgaz Aydinlar, E. P0158, P02

Iljazi, A. AL027

Illert, M. P0395

Imai, N. P0217

Irurtia, M. J. P0177, P0333

Ishiyama, S. P077

Israel-Willner, H. P0346

Israelsen, I. AL031, AL032, P0246

Ivanova, A. P0250

Iyer, R. G. AL022

**J**

Jacobson, A. P0165

Jafari, E. P0181, P032

Jaimes-Sanchez, A. P0345

James Westgate, C. AL031

Janah, A. P0196

Janáky, T. P0247

Janelidze, M. AL048

Janka, L. AL052, P0316, P0327

Jansen-Olesen, I. P053, P057

Jayasurija, S. P0137

Jenkins, B. IL08

Jennysdotter Olofsgård, F. P013

Jensen, C. M. AL053, P0266, P0267

P0268, P0269, P0271, P0272

P0275, P0278

Jensen, R. H. AL031, AL032, P0122

P014, P0154, P0246

Jesse, C. M. P0132

Jiang, L. P0255

Jiménez Jara, E. P0147

Johnson, M. AL059

Johnston, K. P0282, P0339, P0340

Jones, A. AL055, P0276, P0279

Jordan, B. P0123

Jordão, M. P0223, P0363

Joshi, P. AL050

Juhász, G. AL026, P098

Juhl Korsbaek, J. P0122

Juhler, K. P053

Julia, P. G. AL044

Jull, G. AL019, P090

Jung, S. P0227

Jürgens, T. P. P0337

Justice, J. P0362

**K**

Kacprzak, A. P0185

Kalamangalam, G. P0362

Kalra, S. P0220

Kamen, L. AL053, AL054

Kamitaki, B. P0115, P0342

Kamm, K. AL024, P018, P0328

Kang, J.-J. P0141

Kaplanova, Z. P027

Karadas, O. P02

Karagiorgis, G. P017

Karakulova, Y. P0250, P0371

Karatas, H. AL037

Karimi, N. P0355

Karli, N. P02

Karstedt, S. AL012, P0100, P0114, P0156

P0188, P0206, P034, P041

Kashiwaya, Y. P01

Kasioti, E. P017

Kastrati, K. P0159

Kaytser, V. AL01

Kazemizadeh, H. P032

Kaźmierczak, W. P0322

Kecskeméti, G. P0247

Keereman, V. P05

Kelderman, T. P05

Kellier-Steele, N. P095

Kerem, N. P0201, P0216

Kessler, Y. AL043

Khachidze, I. P0251

Khamidova, Z. P027

Khasanova, P. P0250

Khodavirdi, A. P0165

Khorvash, F. P0303, P0304

Khutorov, D. P0403, P0404

Kikui, S. P01

Kim, B.-K. IL07, P010, P011, P0157, P0319

Kim, D. P010

Kim, E. P0269

Kim, J.-M. P010

Kim, S.-K. P011

Kitamura, E. P0217

Klan, T. P0144

Klehr, L. AL042

Klimova, A. P0176, P0182

Klingler, J.-H. P0129

Klærke, D. P053

Knievel, K. P0353

Kocasoy Orhan, E. AL010, P02

Koch, B. P0156

Koch, M. P0308, P0317

Köchling, M. P0346

Kocsel, N. P098

Kofmel, N. P0227

Kogelman, L. J. A. AL021, AL03, AL04

P0225, P048, P053

Kohashi, M. P01

Kökönyei, G. AL026

Komori, M. P0265

Korobkova, D. P0198, P0341, P0403, P0404

Körtési, T. P0247

Koutris, M. P0160

Kovlari, A. P0117

Kozák, L. R. P098

Krasenbaum, L. J. AL022, P0314

Krashin, D. P0107, P0113, P0161, P0162

P0224, P0372, P0394, P06

Kraus, L. M. P0129, P0130

Kraya, T. P0325

Krege, J. P0110, P0265, P0387

Krikke-Workel, J. P0387

Krishnan, A. P029

Kristensen, D. M. AL03, P0193, P025

P053, P057, P059

Kronenbuerger, M. AL042

Krotenkova, M. P092

Kruitbosch, C. P0399

Kruja, J. P0184, P0358

Kuan, A. S. AL015

Kubota, G. P0222, P0223

Kudrow, D. AL053, AL054, P0226

P0271, P0315

Kulagin, A. P0397

Kulisevsky, J. P022

Kull, P. P054

Kumar, A. AL014

Kunz, M. P0135

Kurnukhina, M. P0128, P0194, P0392

Kurth, T. P0337

Kuruvilla, D. AL059

Kuster, G. P0223, P0363, P064

Kwon, S. P0109

Kymes, S. P087

**L**

L'Italien, G. AL014, P0268, P0269, P0272

P0275, P0282, P0339, P0340

La Cour, S. H. P053

Labate, A. P036

Labib, A. P0352

Labruijere, S. P060

Lagka, D. A. P0384

Lagrata, S. IL013

Lai, H. P0281

Lai, J. P0382

Lai, K.-L. AL013, AL06, P0200, P03, P083

Lainez, J. M. IL018

Lalvani, N. AL08

Lambru, G. AL024, P0351, P074

Langa, O. P05

Lanzino, G. P0121

Lara-Lara, M. P085

Larripa, N. P024

Larsson, H. P084

Latysheva, N. AL024

Lau, C. I. P0143

Laumeier, I. P0337

Laursen, S. S. P048

LaVallee, C. AL014

Laven, J. S. P0390

Lavery, G. G. P0119, P0120, P0127, P061

Lay, C. AL08

Layos-Romero, A. P040

Le Foll, C. AL036

Lebedeva, E. R. P0133, P0206

Lee, K.-S. P011

Lee, M. J. AL029, P010, P0109, P011

P0305, P04

Lee, P.-L. P083

Lee, W. P043

Lee, Y.-C. P0200

Lehmann, B. P0227

Lehmenkühler, A. P066

Lemos, C. P050

Lenaburg, K. P0219

Lengil, T. P0230

Leone, M. P019, P020

Leroue, C. P0278

Levin, M. IL010

Lewis, S. P0335

Li, J. AL030, P0137

Li, Y. P0292, P0293, P0294

Liang, Z. AL019, P090

Liao, Y.-C. P0200

Liesering-Latta, E. P0144

Likhachev, S. P0164, P0273

Lim, E. AL02

Lim, S. AL022

Lima, T. P0146, P0186

Lima Florencio, L. P093

Lin, P.-T. P0134, P051

Lindsey, H. P0235

Ling, Y.-H. P028, P051

Linstra, K. M. P0390

Lipton, R. B. AL041, AL053, AL054, AL055

AL060, P0112, P0165, P0219, P0258

P0261, P0262, P0263, P0266, P0267

P0268, P0269, P0271, P0272, P027

P0276, P0286, P0292, P0293, P0294

P0295, P0296, P0299, P0312, P0382

P087, P088

Lirng, J.-F. P0134, P0200, P051

Liu, H.-Y. P083

Liu, Y. AL020

Llauradó, A. P040

Lo, I. P0284

Lobo, B. P0362

Loder, E. P096

Lombard, L. P0232, P0387

Lonergan, R. P0306, P0338

Long, N. P0306, P0338

Longo, D. P082

López-Bravo, A. P0350

Lopez-Lopez, C. AL025, P079

Lorenz, D. AL08

Losada López, M. P0105, P0336

Lotufo, P. AL017, P052

Lovati, C. AL057, P0343

Lovegren, M. P0266, P0269, P0271

P0272, P0275, P0278

Lozan, T. P0212

Lozano, A. G. P039

Lu, K. P0294, P0295

Lucchese, S. P0253

Luedtke, K. P0202, P0203

Lundgren, L. P. P048

Lutsenko, I. P0236

Lutz, T. AL036

Luzeiro, I. P0211

Lyons, H. AL035, AL040

Lyras, L. P0229

**M**

Mills, K. M.P0232

Ma, J. P0335

Maarbjerg, S. P0189, P0193, P025

Maassen van den Brink, A. AL023, P0390

P0399, P060

MacGregor, A. P0167, P0265, P0281

Machado, S. P042

Machfoed, M. H. P0366

Macken, E. P0306, P0338

Madera Fernández, J. P023

Madhani, S. AL020

Madonia, J. P0267

Maggioni, A. P. P08

Maghbooli, M. P0376

Magis, D. IL021

Magomedova, T. P027

Maier-Peuschel, M. AL058, P0317

Makeeva, T. P0403, P0404

Makri, E. P0384

Manack Adams, A. P0262, P0263, P0285, P088

Manfredini, D. P0160

Mangano, S. P0170, P0207

Manganotti, P. P0178, P033, P0391

Manickam, A. H. P0307

Manieri, S. P038

Manjelievskaia, J. P0218, P097

Mann, J. AL059

Manrique, L. P0364

Manthena, S. AL060

Manzo, L. AL045, P0318, P0347

Manzo, M. L. P0170

Marchenko, V. P0138

Marfil, A. P0214, P0368

María José, N. M. AL044

Mariano da Silva Junior, H. P0388

Marín-Labanda, R. P0309

Marino, A. P0170

Markey, K. AL035

Markin, K. P0101

Markowitz, S. P0196

Marmura, M. P0323

Marsico, O. P036

Martami, F. P0181

Martelletti, P. AL024

Martin, V. AL048, P0289

Martindale, C. P0145

Martinelli, D. P0252, P0311, P0330, P0332

Martínez Badillo, C. P039

Martini, N. P08

Martins, B. P0131

Maryenko, I. P0164, P0273

Marzal, C. N. P0152

Mas, N. P022

Mascarella, D. AL046

Massip, M. P0290, P0291

Matharu, M. AL034

Matsaridis, D. P0360

Matteo, E. AL046

Matthews, T. P0119

Mattos, B. P0222

Maughn, K. P0314

Mauri, M. P0257

Mavridis, T. P017

Mayda Domac, F. P02

Mazzoni, G. P048

McAllister, P. P0106, P0294, P0316, P0320

McConnachie, L. P0264

McCool, N. P0234

McGinley, J. S. P087

McPartland, T. P0234

McVige, J. P0299, P099

Meani, A. P0329

Mechtler, L. P0299, P0327, P099

Mecklenburg, J. P0287, P0288, P054

Meder, G. P0322

Medina, J. P. P019

Mehta, L. AL048

Meilof, R. P07

Melling, J. P0306, P0338

Membrilla, J. A. P0283, P040, P085

Mendes Bragatto, M. P093

Menendez, D. P0363

Mercante, J. AL017, P052, P069

Mercuri, B. AL045, P0347

Meredig, H. P0123

Merrill, R. P0192

Mesquita, I. P0131

Messina, R. AL028, AL045, P0318

P0329, P0343, P0347

Messina, V. P0208, P0209

Meun, C. P0390

Miceli, R. P0294, P0295, P0296

P0301, P089

Min, I. K. P043

Mingels, S. P0357

Mireya, L. L. AL044

Misci, P. AL028

Miscio, A. M. AL024

Mitchell, J. AL035, AL040, P0118, P0119

P0120, P0124, P0125, P0127

Mitropoulou, E. P017

Mitsikostas, D. P017

Mittleman, M. P0166

Miyahara, J. P01

Mo, H. P0157

Moavero, R. AL07, P0208, P0209, P0210

P0361, P0385, P082, P09

Moita, L. F. P015

Moldovanu, I. P0212

Moleirinho-Alves, P. P0190

Molina, M. D. C. AL017, P052

Mollan, S. P. AL035, AL040, P0118

P0119, P0120, P0124, P0125

P0127, P061

Mondal, S. P0319

Montilla, C. P039

Moon, H.-S. P010, P011, P0157

Mora, M. P039

Morelli, M. E. P0391

Morgenstern, S. P0176

Moriya, A. P0217

Moro, R. AL011, P031, P035

Morollón, N. P022, P0290, P0291

Morris, B. P0268

Morscheck, M. AL012, P0114, P0188

P034, P041

Morton, B. P0219

Moskaleva, P. P0401

Mostofsky, E. P0166

Mozheiko, M. P0164, P0273

Mullin, K. P0271, P0278

Munro, G. P057

Münte, T. AL012, P0114, P0188, P0206

P034, P041

Murdin, L. P0351

Murinova, N. P0107, P0113, P0161

P0162, P0224, P0372, P0394, P06

Murphy, A. P0267

Murphy, J. P0298

Musialowicz, B. P0115

Mytton, J. P0124

**N**

Nachev, P. AL034

Nada, M. P0169

Nadkarni, S. P0353

Nagel, M. V. P024

Nägel, S. AL043, P0315, P0337

Nagy, A. J. AL048

Nahas, S. J. AL041, AL043, P0313, P0321

P0353, P088

Nair Ramadasan, P. P0220

Napolitano, A. P082

Nardello, R. P0170, P0207

Narula, A. P0113, P0161

Nasergivehchi, S. P032

Nasyrova, R. P0401

Nattan Portes Souza, M. P0230, P064

Nava, N. P0214

Navarro-Pérez, M. P. P0147, P0177

P0179, P0309, P0333, P063

Navetta, M. P0165

Neeb, L. P0287, P0288, P0325, P0328

P0337, P054

Negro, A. AL024

Nehrych, T. P0365

Nesterovskiy, Y. P0400

Neves, P. P0191

Neves-Costa, A. P015

Newman, L. AL08

Nguyen, L. P012

Nianiarou, M. P0384

Nicholls, M. P0127

Nichols, R. P095

Nie, L. P0256

Nielsen, C. A. AL025, P080

Nieves Castellanos, C. AL044, P0105, P0336

Nigri, A. P019, P020

Nikolova, S. AL030, P0137

NInashvili, N. P0183

Ning, X. AL043, AL051, AL052, P0108

P0313, P0315, P0316, P0320

P0321, P0323, P0327

Nocera, G. M. P0207, P045

Noordam, R. AL04

Noory, M. P025

Noory, N. P0189, P0193, P025

Noseworthy, P. AL02

Novitckii, I. P065

Nowaczewska, M. P0322

Nuñez, G. P039

Nyári, A. P0247

**O**

O'Carroll, S. AL036

O'Fallon, G. P0113

O'Gorman, C. AL055, P0276, P0279

Odobescu, S. P0212

Odriozola-González, P. P0177, P0333

OGUZ AKARSU, E. P02

Oh, K. P010, P011

Oh, S.-Y. P0141

Okine, B. P074

Olesen, J. AL021, AL03, P0133, P0204

P0206, P0225, P048, P053, P057

Oliveira, A. P. P0223

Oliveira, A. AL017, AL018, P0186

P052, P069

Oliveira, R. P0190

Oliveira, R. P015, P042

Oliveira Gonçalves, A. S. P0337

Oliveri, D. P0112

Oliveros-Cid, A. P0350

Olivier, M. P024

Olivo, S. P0178, P033

Olofson, I. P0225

Olose, E. P0238

Oparah, S. P0238

Ordoñez-Carrasco, J. P0148, P0149

Orlova, Y. P0362

Ornello, R. AL024, P0142

Orr, S. L. P0261, P0370

Ortler, S. P0317

Ota, K. P01

Oterino, A. P030

Ottridge, R. P0118, P0119, P0120

Overeem, L. H. P0287, P0288, P0328, P054

Özge, A. P02

Ozguner, H. P02

Ozomma, S. P0238

O’Connell, N. P0172, P096

**P**

Paço, M. P050

Padala, D. P0354

Paemeleire, K. P0205

Pagès, N. P0290, P0291

Paiva Da Silva Lima, G. AL049, P0319

Palacio, E. P0364

Palavra, F. P0211

Palotie, A. AL04

Pan, L.-L. AL013, P028, P081, P083

Pandhi, S. P0319

Paniagua, M. P039

Panza, G. AL059

Pap, D. P098

Papajani, M. P0358

Papathanasiou, E. P0384

Papetti, L. AL07, P0208, P0209, P0210

P0361, P0385, P082, P09

Parisi, V. P0103

Park, B.-y. AL029

Park, H.-K. P0141

Park, J. W. P010, P011

Park, K.-Y. P011

Parreira, E. P0151, P0324, P0326

P0349, P042

Pascarella, A. AL045, P0347

Pascual, J. IL024, P0152, P023, P0364

Pasquin, F. P033

Passarelli, V. P0222

Pavão Martins, I. P0396

Pavlovic, J. M. P0275, P0281

Paz-Solís, J. P085

Pazdera, L. P0108

Pedarla, V. P0314

Pedersen, A. S. P014, P0154

Pedersen, O. P0154

Pedrini, A. P08

Pedroso, D. P015

Peikert, A. P0346

Peikert, A. P0328

Pelizaro, M. O. P0173

Pellesi, L. P079

Pelzer, N. AL047

Pensato, U. AL046

Penzien, D. B. P096

Pereira, L. P0405, P042

Pereira Monteiro, J. P050

Perenboom, T. P07

Peres, M. AL017, AL018, P0146, P0186

P052, P069

Perez, C. P039

Pérez García, J. P0105, P0336

Pérez-Pereda, S. P0152, P023, P030, P0364

Perry, A. P0218, P097

Petersen, A. S. P014, P0154

Petersen, S. P057

Petolicchio, B. P0103

Petrova, M. P0401

Pezarat-Correia, P. P0190

Piechowiak, E. I. AL039, P0132

Pierangeli, G. AL046, AL057, P0155

P0343, P08

Pierce, C. R. P0172, P096

Pierelli, F. P0104

Piffer, A. P0191

Pinardi, C. P019, P020

Pincerato, R. P0223, P0363

Pinheiro, R. P0324, P0349

Pinho, T. P050

Pires, R. AL018

Pirinen, M. AL04

Piro, E. P0170

Pistoia, F. P0142

Plácido, M. P042

Planchuelo-Gómez, Á. AL011, P0177, P031

P0333, P035, P063

Plevoets, K. P0205

Po'latova, D. P0393

Pochert, A.-M. P0176

Poh, W. AL060, P0258

Polanco, M. P0364

Polushin, A. P0397

Ponzano, M. P0245, P055

Popoff, E. P0282, P0339, P0340

Porta-Etessam, J. P040

Potapov, O. P0136, P0344

Pourakbar, E. P0168

Powell, L. P0282, P0339, P0340

Poynter, J. P0362

Pozo-Rosich, P. AL024, P0229, P0261

P0292, P0293, P0294, P040, P073

Pressman, A. P0165

Princiotta Cariddi, L. P0257

Proietti, S. P0318

Proietti Cecchini, A. P019, P020

Proietti Checchi, M. P0208, P0209

Pundir, J. P0167

Putortì, A. P0311, P0332

**Q**

Qazimllari, B. P0358

Quatrosi, G. P0207

Quillen, J. P0259

Quinn, J. P0256

Quintas, S. P0177, P0333, P063

**R**

Raab, S. P0159

Raabe, A. AL039, P0130, P0132

Raffaelli, B. P0287, P0288, P0328

P0337, P054

Raghava, J. P084

Rahim, T. P0339

Rahimi, M. D. P0355

Raieli, V. P0170, P0207, P045

Ramirez Campos, V. AL043, AL051, AL052

P0108, P0313, P0315, P0316

P0320, P0321, P0323, P0327, P0335

Ramos, R. P022

Ran, C. P013

Ranieri, A. AL057, P0343

Ranjbar Ekbatan, M. P0241, P0243

Rantell, K. AL034

Rao, G. N. P0386

Rao, R. AL057, P0343

Rapisarda, L. P036

Rasmussen, A. AL03

Ratanov, M. P0397

RAY, S. P0264

Razeghi Jahromi, S. P047

Rebecchi, V. P0257

Reed, M. L. AL041, AL060, P0261, P088

Rees, T. AL036

Rêgo, A. P0326

Reshef, S. AL022

Reuter, U. AL049, AL058, P0229, P0287

P0288, P0301, P0328, P0337

P0387, P054, P089

Rezaeimanesh, N. P047

Richter, F. P066

Richter, M. P0182

Rick, C. P0119

Riley, S. P0235

Rimmele, F. P0337

Ripatti, S. AL04

Rivera-Mancilla, E. AL023

Rizos, T. P0123

Roa, J. P085

Robertson, C. AL020

Rocca, M. A. AL028

Rochat, P. P0189, P0193

Rodgers, A. P0354

Rodrigues Jordao, M. P064

Rodríguez, M. AL011, P031, P035

Rodríguez Montolio, J. P0147

Rodríguez Rodríguez, E. M. P023

Rodriguez-Blanco, C. P0379

Rodriguez-Vico, J. S. P0345

Rojas, M. P039

Rölz, R. P0129

Romano, C. P0335

Ronconi, G. P08

Ross, K. AL030, P0137

Rossnagel, F. P0346

Rostgaard, K. P0225

Rotaru, L. P0402

Royl, G. P0206

Rroji, A. P0358

Rubin, A. AL022

Ruff, D. P0110

Ruiz, M. P039

Ruiz Castrillo, M. J. P0297

Ruotsalainen, S. E. AL04

Ruscheweyh, R. AL024, P0135, P018, P0328

Ruscitto, C. P0361, P0385

Russo, A. AL024

Russo, M. AL024

Rustamova, C. P0393

Ruttledge, M. P0306, P0307, P0338

Rykova, E. P0351

**S**

Mackie, D. S.P0232

Saatchi, M. P032

Sacco, S. AL024, P0142

Sadeghi, E. P0377

Sahin, A. P02

Sahin, E. P02

Saikali, N. P099

Salam, A. P0354

Salehi Fadardi, J. P0355

Salerno, A. AL045, P0347

Salhofer-Polanyi, S. P0159

Samuel, D. I. AL044

Sancak, B. P0158

Sances, G. P0139, P0300, P0302

P0311, P0330, P0332, P0373, P091

Sanchez, C. P0228

Sánchez-Casado, L. P0283, P0297

P0331, P0334

Santangelo, A. P0170, P0207

Santangelo, G. P0207

Santoro, A. AL024

Santos, F. R. P0222, P0363

Santos-Lasaosa, S. P0147, P0177, P0309

P0333, P063

Saporito, G. P0142

Sastre-Real, M. P0260, P0283, P0297

P0331, P0334, P040

Satterly, K. P0264

Savastano, L. AL020

Sawai, A. P0217

Sayin, I. E. P0158

Scafoglieri, A. P0160

Schaible, H.-G. P066

Schankin, C. P0227

Schauerte, I. P0325

Schiano Di Cola, F. AL057, P0343

Schim, J. D. P0266, P0285

Schmidt Alves Ferreira Galvão, R. P049

Schmutzer, M. P0135

Schreiber, C. P. P0282

Schreiber, J. P05

Schroeder, K. P095

Schuh, K. P0218, P097

Schwarz, A. P0202, P0203

Schwedt, T. J. AL030, AL050, P0110

P0137, P0232, P0295

Schweitzer, P. P0159

Schytz, H. AL027

Sciruicchio, V. AL07

Scott, A. P0165

Scotton, W. P0119

Sebastianelli, G. P0102, P0104

Seelam, P. AL014

Segura, T. P040

Selyanina, N. P0250

Seminerio, M. J. P0314

Seng, E. K. P0235

Sequeiros, J. P050

Sergeeva, A. P092

Serrano, C. P0152

Serrano, D. P0258, P0262, P0263

Serrao, M. P0102, P0103

Setuguti, D. P0363

Severt, L. P0292, P0293, P0294, P0295

P0296, P0299, P0301, P089

Sevillano-Orte, L. P0350

Sforza, G. AL07, P0208, P0209, P0210

P0361, P0385, P082, P09

Shaafi, S. P0377

Shabir, A. P057

Shafiyev, J. P02

Shahid, A. AL020

Shaikh, E. P0306, P0338

Shaikh, S. H. AL09

Shalaby, N. P0169

Sharon, R. P0348

Shavdia, M. P0183

Shehata, H. P0169

Shewale, A. R. AL060, P0112

Sheykhzade, M. P076

Shibata, Y. P077

Shibuya, M. P0363

Shimohata, K. P0356

Shimohata, T. P0356

Shnayder, N. P0401

Shoukri, A. P0140

Shrewsbury, S. P012, P0264

Shrukalla, A. A. P0306, P0338

Shubina, M. P046, P065

Shulman, K. J. AL022

Siakavella, D. P0384

Sico, J. J. P0235

Sieder, C. AL058

Sierra, L. P039

Sierra Mencía, Á. P0175, P039, P040, P063

Silberstein, S. D. AL049, P0286, P0295

P0313, P0321

Siliņa, M. P070

Silva, A. P0211

Silva, A. V. d. P0173

Silva, E. P0405

Silva, J. P0191

Silva Júnior, H. P0191

Silvestro, M. AL024

Simioni, C. P0222

Simons, J. AL047

Sinclair, A. J. AL035, AL040, P0118, P0119

P0120, P0124, P0125, P0127, P061

Singhal, R. P0118, P0119

Sjöstrand, C. P013

Skandarioon, C. AL025

Skiba, I. P0397

Skorobogatykh, K. P0150, P0198, P0221

P0341, P0403, P0404

Smeltere, L. P070

Smilkov, E. A. P0189, P0193

Smith, M. P0117

Smith, T. AL053, P0278, P0353

Snellman, J. AL025, P079

Snoer, A. P014, P0154

Sobhani, A. P0187

Sohn, J.-H. P010, P011

Sommer, K. P0286

Song, T.-J. P011

Sorokin, M. P0101

Sotnikov, D. P0136, P0344

Sousa, A. P050

Souza, M. N. P0222, P0223, P0363

Spanou, I. P0360

Speck, R. P0226

Spierings, E. L. H. AL051, P0353

Splendiani, A. P0142

Springborg, J. B. P0189, P0193

Starikova, N. P0371

Starling, A. AL059, P0259

Stefánsson, H. AL04

Stefansson, K. AL04

Stegmann, G. P0137

Stein, K. AL08

Steinberg, A. P013

Stepanchenko, K. P0138

Stewart, W. P0165, P088

Stock, D. A. AL054

Stock, E. G. AL054, P0266, P0275

Stokes, J. P0292, P0293, P0299

Storch, E. P054

Straburzyński, M. AL024, P0322

Straube, A. P0135, P018, P0325

Strauß, S. AL042

Stria, T. P0159

Stubberud, A. AL034

Stühler, E. P0346

Su, Y. AL047

Sudibyo, D. A. P0153, P0366

Sugiyama, H. P01

Suharjanti, I. P0366

Sukessada, M. P0191

Sun, K. P0255

Suslina, A. P092

Svensson, P. P0241, P0243

Sweeney, C. P0335

**T**

Tabuteau, H. AL055, P0276, P0279

Tagliente, M. P038

Tahrani, A. P0118, P0119, P0127

Tajti, J. P0247

Takahashi, T. P074

Takeshima, T. P01

Tangirala, K. P0314

Tarantino, S. AL07, P0208, P0209, P0210

P0361, P0385, P082, P09

Taricani Kubota, G. P064

Tarumov, D. P0101

Tashiro, L. AL012, P0114, P0188, P034, P041

Tasma, Z. P058

Tassorelli, C. P0110, P0139, P0252, P0289

P0295, P0300, P0302, P0311

P0312, P0330, P0332, P0373, P056, P091

Tchuenga Fokom, W. P044

Tedeschi Benatto, M. P093

Teja, F. P0241, P0243

Temsah, M.-H. AL012, P0114, P0188

P034, P041

Tepper, S. J. AL050, AL055, AL059, P0276

P0301, P0348, P089

Tereshchenko, S. P046, P065

Terhart, M. P0287, P0288, P054

Termine, C. AL07

Terwindt, G. M. AL047, P0390, P0399

P07, P072

Terzoudi, M. P0384

Tescari, F. P0363

Thakur, S. P0310

Thaller, M. P0120, P0124, P0125

Thiele, A. AL042

Thiry, A. AL053, P0266, P0267, P0268

P0269, P0271, P0272, P0275

P0278, P0339

Thomas, L. F. AL04

Thomas, L. AL019, P090

Thompson, J. AL050

Thompson, S. F. P0314

Thorat, A. P0310

Thorsteinsdottir, J. P0135

Thunstedt, D. P0135

Tinelli, E. P0103

Tiwari, S. AL050

Tofaha Alhusseini, R. P0383

Togha, M. IL016, P0171, P0181, P032

P0381, P047

Toldo, I. AL07

Tomino, C. P0231

Tomkins, E. P0306, P0307, P0338

Tömösi, F. P0247

Topic, P. P0159

Torelli, P. AL024, AL045, P0245, P0347, P055

Toriello, M. P030

Torphy, B. P0117

Torres, E. P0364

Torres-Ferrus, M. IL01, P073

Toumasis, A. P0384

Tozzi, E. AL07

Tozzi, V. P0346

Traut, A. P099

Treleaven, J. AL019, P090

Treviño-Peinado, C. P0228, P0260

Trigo López, J. P039, P040

Trimboli, M. AL045, P0347, P036, P038

Tronci, S. P0329

Tronvik, E. AL034

Troy, E. P0306, P0338

Trufanov, A. P0101

Trugman, J. M. P0262, P0263, P0281

P0294, P0295, P0296, P0301

P0382, P089

Truman, J. P0298

Tsanoula, S. P017

Tsermoulas, G. AL035, AL040

Tsurkalenko, A. P0237

Tsurkalenko, O. P0195, P0237, P0367

Tuka, B. P0247

Turner, D. IL023

Turner, I. P0272, P0275

Tzeng, Y.-S. P0200

**U**

Ueno, D. P0191

Ulrich, C. T. P0132

Urbach, H. P0129

Urbinati, G. P0155

Urman, R. P0165

Urru, M. P062

Ursitti, F. AL07, P0208, P0209, P0210

P0361, P0385, P082, P09

Ushenin, A. V. P0133

Uzay, B. AL037

**V**

Vaghi, G. P0139, P0300, P0302, P0311

Vaidya, S. P0165

Valeriani, M. AL07, P0208, P0209, P0210

P0361, P0385, P082, P09

Valeriano, R. P0363

Valsasina, P. AL028

Van den Berg, J. P0390

van den Bogaerdt, A. AL023, P060

van den Hoek, T. P072

van den Maagdenberg, A. AL04, IL019

Van der Ham, K. P0390

van Etten, L. P0357

Vanacore, N. P0231

Vandenbussche, N. P0205

Vandenheede, M. P05

VanderPluym, J. AL052, P0335

Varnado, O. P0218

Vashchenko, N. P0150, P0198, P0341

P0403, P0404

Vázquez, J. L. P0364

Vécsei, L. P0247

Velamazán, G. P0152

Verdecchia, P. AL07

Veréb, D. P0247

Verhagen, I. P0399, P072

Vernieri, F. AL024, AL045, AL057, P0318

P0343, P0347

Versijpt, J. P05

Versino, M. P0257

Vetri, L. P0207

Vgontzas, A. P0166

Vigevano, F. P0208, P0209, P0210, P0361

P0385, P09

Vijay, V. AL035, P0119, P0127

Villalón, C. M. AL023

Vincent, M. P0232, P0265

Virk, A. P0192

Visočnik, D. P075

Vitali da Silva, A. P049

Vives-Mestres, M. P0370

Vladovskaya, M. P0397

Vo, P. AL050

Voci, A. P09

Volante, A. J. P0173

Vuletic, V. P0369

Vuralli, D. P02

**W**

Ayer, D. W.P0232

Wakerley, B. R. AL040, P0119, P0120

P0124, P0125, P0127, P061

Waldenlind, E. P013

Walker, C. AL036, P058, P094

Walker, J. AL035, AL040

Walsh, V. P0143

Wang, G. H. Y. P0241

Wang, H. AL02

Wang, M. P0255, P0256

Wang, S.-J. AL013, AL015, AL06, IL09

P0134, P0200, P028, P03, P0319

P051, P081, P083

Wang, Y.-F. AL013, AL015, P0134, P0200

P028, P03, P051, P083

Ward, T. IL011

Weerasinghe, C. P0298

Wells, R. E. IL017, P0172, P096

Wen, S. P0319

Wenzel, R. P0218, P097

Wermer, M. J. H. P0390

Westgate, C. S. J. AL032, P0119, P0246, P025

Williams, G. P074

Williams, V. P0335

Wilms, A. AL047

Wilson, L. P0112

Winner, P. P0272

Winsvold, B. S. AL04

Wirth, C. P013

Wirth, L. P0123

Wirth, R. J. P087

Witthöft, M. P0144

Wöber, C. P0159

Wolfe, J. P0353

Wolfram, F. P084

Wolfrum, S. P0206

Wu, J.-W. P0134, P03

Wu, T. AL030, P0137

**X**

Xhelili, M. P0184

Xhumari, A. P0358

Xia, J. P0301, P089

Xing, K. P0172

Xui, F. AL049

**Y**

Yakubova, M. P0393

Yalınay Dikmen, P. AL010, P0158, P02

Yamani, N. P0171

Yang, X. P0378

Yarr, S. P0335

Yavuz, B. P0158

Ye, W. P0218, P097

Yemisci, M. AL037

Yiangou, A. AL035, AL040, P0118, P0119

P0120, P0124, P0125, P0127

Yilmaz-Ozcan, S. AL037

Young, W. P0219

Yu, C.-C. AL015

Yu, S. P0378

Yu, S. Y. P0281, P0382

Yu, T. P0378

**Z**

Zachariadi, C. P0384

Zala, N. P0123

Zaletel, M. P075

Zamanian, S. P0168

Zanaboni, A. M. P0252, P056

Zareie, H. AL019

Zarifoglu, M. P02

Zavadenko, N. P0400

Zebenholzer, K. P0159

Zebenigus, M. P0213

Zeidan, F. P096

Zekja, I. P0184

Zewde, Y. P0213

Zhang, B. P0378

Zhang, F. AL049

Zhang, J. P0137

Zhang, N. AL02

Zhang, P. AL01, P0115, P0240, P0284, P0342

Zhao, S. P0299

Zhong, S. P0378

Zick, B. K. T. P0390

Ziemann, M. AL012, P0114, P0188

P034, P041

Zsombók, T. P098

Zucco, M. AL045, P0318, P0347

Zupan, M. P075

Žvan, B. P075

Zwart, J.-A. AL04

**Ø**

Østerberg, O. P087

